# Lineage-Specific Growth Curves Document Large Differences in Response of Individual Groups of Marine Bacteria to the Top-Down and Bottom-Up Controls

**DOI:** 10.1128/mSystems.00934-21

**Published:** 2021-09-28

**Authors:** Lívia K. Fecskeová, Kasia Piwosz, Danijela Šantić, Stefanija Šestanović, Ana Vrdoljak Tomaš, Martina Hanusová, Mladen Šolić, Michal Koblížek

**Affiliations:** a Center Algatech, Institute of Microbiology of the Czech Academy of Sciences, Třeboň, Czechia; b National Marine Fisheries Research Institute, Gdynia, Poland; c Laboratory of Marine Microbiology, Institute of Oceanography and Fisheries, Split, Croatia; Max Planck Institute for Marine Microbiology

**Keywords:** aerobic anoxygenic phototrophs, amplicon sequencing, bacterioplankton, grazing, growth curves, growth rate, manipulation experiment, phosphorus limitation, top-down control

## Abstract

Marine bacterioplankton represent a diverse assembly of species differing largely in their abundance, physiology, metabolic activity, and role in microbial food webs. To analyze their sensitivity to bottom-up and top-down controls, we performed a manipulation experiment where grazers were removed, with or without the addition of phosphate. Using amplicon-reads normalization by internal standard (ARNIS), we reconstructed growth curves for almost 300 individual phylotypes. Grazer removal caused a rapid growth of most bacterial groups, which grew at rates of 0.6 to 3.5 day^−1^, with the highest rates (>4 day^−1^) recorded among *Rhodobacteraceae*, *Oceanospirillales*, *Alteromonadaceae*, and *Arcobacteraceae.* Based on their growth response, the phylotypes were divided into three basic groups. Most of the phylotypes responded positively to both grazer removal as well as phosphate addition. The second group (containing, e.g., *Rhodobacterales* and *Rhizobiales*) responded to the grazer removal but not to the phosphate addition. Finally, some clades, such as SAR11 and *Flavobacteriaceae*, responded only to phosphate amendment but not to grazer removal. Our results show large differences in bacterial responses to experimental manipulations at the phylotype level and document different life strategies of marine bacterioplankton. In addition, growth curves of 130 phylogroups of aerobic anoxygenic phototrophs were reconstructed based on changes of the functional *puf*M gene. The use of functional genes together with rRNA genes may significantly expand the scientific potential of the ARNIS technique.

**IMPORTANCE** Growth is one of the main manifestations of life. It is assumed generally that bacterial growth is constrained mostly by nutrient availability (bottom-up control) and grazing (top-down control). Since marine bacteria represent a very diverse assembly of species with different metabolic properties, their growth characteristics also largely differ accordingly. Currently, the growth of marine microorganisms is typically evaluated using microscopy in combination with fluorescence *in situ* hybridization (FISH). However, these laborious techniques are limited in their throughput and taxonomical resolution. Therefore, we combined a classical manipulation experiment with next-generation sequencing to resolve the growth dynamics of almost 300 bacterial phylogroups in the coastal Adriatic Sea. The analysis documented that most of the phylogroups responded positively to both grazer removal and phosphate addition. We observed significant differences in growth kinetics among closely related species, which could not be distinguished by the classical FISH technique.

## INTRODUCTION

Marine bacteria represent a complex assembly of species with different physiology, metabolic capacity, and substrate preferences. They play an important role in biogeochemical processes, such as the secondary production of particulate organic carbon. They also remineralize some key biogenic elements, such as nitrogen and phosphorus, that later serve as a substrate for other organisms. The ultimate measure of microbial activity is growth. The specific growth rate (μ) is defined as the relative change of microbial biomass (*B*) per unit of time (t), as follows: μ = ∂*B*/*B* × 1/∂*t*. While bacterial biomass is relatively easy to measure in laboratory batch cultures, it is challenging to determine it in natural planktonic samples. Moreover, in a dynamic marine environment, the bacterial community is being shaped constantly by predation, viral lysis, or other factors (1). Therefore, the relative change in bacterial biomass (net growth) reflects the difference between intrinsic (gross) growth rate and mortality rate.

Currently, there are two basic approaches for the determination of gross growth rates of aquatic bacteria. One approach uses specific tracer molecules, for which the rate of biosynthesis approximately equals the rate of biomass increase. Bacterial growth is usually estimated from the incorporation of radiolabeled thymidine as a proxy for bacterial DNA biosynthesis (2) or from the incorporation of radiolabeled leucine used as a proxy for bacterial biomass synthesis (3). A novel approach is the measurement of bacterial phospholipid biosynthesis rates using radiolabeled phosphate, which could be directly related to bacterial growth rates without the need of any empirical factors (4). On the other hand, this labor-intensive method can be applied only under relatively specific conditions of nutrient-limited upper oceans.

The second approach minimizes bacterial mortality by removing grazers by sample prefiltration (5–7) or dilution with water free of microorganisms (1). The growth rate is then analyzed from the increase of the cell numbers counted by microscopy or flow cytometry. In combination with fluorescence *in situ* hybridization (FISH), it is possible to determine growth rates of specific phylogenetic groups, which is a major advantage in comparison with the use of molecular tracers. However, the laborious enumeration of microscopic samples significantly limits the number of analyzed groups in one experiment.

16S rRNA amplicon sequencing has made it possible to obtain information about the composition of natural microbial communities at great depth and low cost. Unfortunately, this technique is not quantitative. Biases introduced during the library preparation, especially PCR amplification, make it impossible to relate read numbers with the absolute or relative abundance of the individual phylogenetic groups (8) or even to compare read numbers for the same phylogenetic group between different samples. To overcome some of these limitations, we recently developed a new approach called amplicon reads normalization with internal standard (ARNIS) (9). The principle of the method is the addition of a defined number of bacterial cells absent in the studied environment, which serve as an internal standard. The cells are added to the collected samples directly before their filtration. After the amplicon sequencing of the samples, the read numbers of a specific phylogroup (operational taxonomic unit [OTU]) are normalized with read numbers of the internal standard for each sample separately, creating the ARNIS ratio. This approach accounts for sample-to-sample artifacts connected to sample collection, extraction, amplification, and sequencing. Due to the PCR bias, the ARNIS ratio cannot be used for absolute quantification. However, assuming a constant PCR bias for the same phylogroup, it is possible to reconstruct growth curves from normalized read numbers for individual phylogroups between samples taken at different times. This approach was verified during a manipulation experiment of the freshwater Římov Reservoir using catalyzed reporter deposition fluorescence *in situ* hybridization (CARD)-FISH, which documented that growth curves for key freshwater bacterioplankton lineages reconstructed by ARNIS were in good agreement with those by microscopic analyses (9).

Since information about growth rates of individual phylogroups in the marine environment is scarce, we decided to apply our approach in the Adriatic Sea coastal waters close to Split, Croatia. To determine the relative importance of top-down and bottom-up control of individual phylogenetic groups of marine bacterioplankton, we performed an experimental grazer removal with or without an addition of phosphate. Changes of the bacterial community were followed using flow cytometry, microscopy, and 16S rRNA amplicon sequencing, and in combination with ARNIS, we reconstructed growth curves of individual phylogroups.

In principle, the ARNIS method is not limited to the 16S rRNA gene. It can also be applied for functional genes. Therefore, we used it for *puf*M gene (encoding the M subunit of the bacterial reaction center) amplicon sequences in an attempt to reconstruct growth curves of aerobic anoxygenic phototrophic (AAP) bacteria. AAP bacteria are a taxonomically diverse group of microorganisms, which use bacteriochlorophyll-containing reaction centers to harvest light energy (10). They are metabolically active organisms, which frequently grow faster than heterotrophic bacteria (11, 12).

## RESULTS

### Ecological context.

The experiment was conducted in early May 2019, 1 day after a strong wind mixed the upper water column. The values of main physicochemical and biological parameters are listed in Table 1. The microphytoplankton community was dominated by coccolithophores (*Coccolithophoridae* spp., Emiliania huxleyi), diatoms (*Chaetoceros* spp., Pseudonitzschia delicatissima group), and dinoflagellates (*Gymnodinium* sp., *Heterocapsa* sp.). Picophytoplankton was dominated by *Synechococcus* sp. (23.3 × 10^3^ ml^−1^ ± 0.50 × 10^3^ ml^−1^), eukaryotes (4.99 × 10^3^ ml^−1^ ± 0.25 × 10^3^ ml^−1^), and *Prochlorococcus* sp. (0.33 × 10^3^ ml^−1^ ± 0.07 × 10^3^ ml^−1^). The initial composition of the bacterial community assayed by 16S rRNA amplicon sequencing was typical for coastal waters. The most abundant phyla were *Cyanobacteria* (*Synechococcales*, 25%), *Bacteroidota* (*Flavobacteriales*, 23.5%), *Alphaproteobacteria* (*Rhodobacterales*, 14%; *Rhizobiales*, 2%; *Puniceispirillales*, 1.5%; SAR11, 1.5%), and *Gammaproteobacteria* (*Oceanospirillales*, 7.3%; *Cellvibrionales*, 6.6%; *Alteromonadales*, 5.5%; *Burkholderiales*, 1.25%; SAR86, 0.53%). Other less abundant phyla were *Planctomycetota*, *Bdellovibrionota*, *Verrucomicrobiota*, *Actinobacteriota*, *Campilobacterota*, and *Firmicutes*.

**TABLE 1:** Seawater sample characteristics

Parameter	Value
Water temp (°C)	16
Salinity (psu)	35.3
NO_3_ (μmol liter^−1^)	0.095
NO_2_ (μmol liter^−1^)	0.071
NH_4_ (μmol liter^−1^)	0.483
TIN (μmol liter ^−1^)	0.649
N_TOT_ (μmol liter^−1^)	8.286
PO_4_ (μmol liter^−1^)	0.011
P_TOT_ (μmol liter^−1^)	0.055
SiO_4_ (μmol liter^−1^)	0.669
N:P	150
Chlorophyll (μg liter^−1^)	1.07
Heterotrophic bacteria (cells liter^−1^ ±SD)	52.1 × 10^7^ ± 4.3 × 10^7^
Cyanobacteria (cells liter^−1^ ±SD)	2.87 × 10^7^ ± 0.22 × 10^7^
AAP bacteria (cells liter^−1^ ±SD)	5.5 × 10^7^ ± 0.9 × 10^7^
Primary production (μg carbon liter^−1^ day^−1^)	43.13

### Effects of manipulation on microbial community.

**FIG 1:**
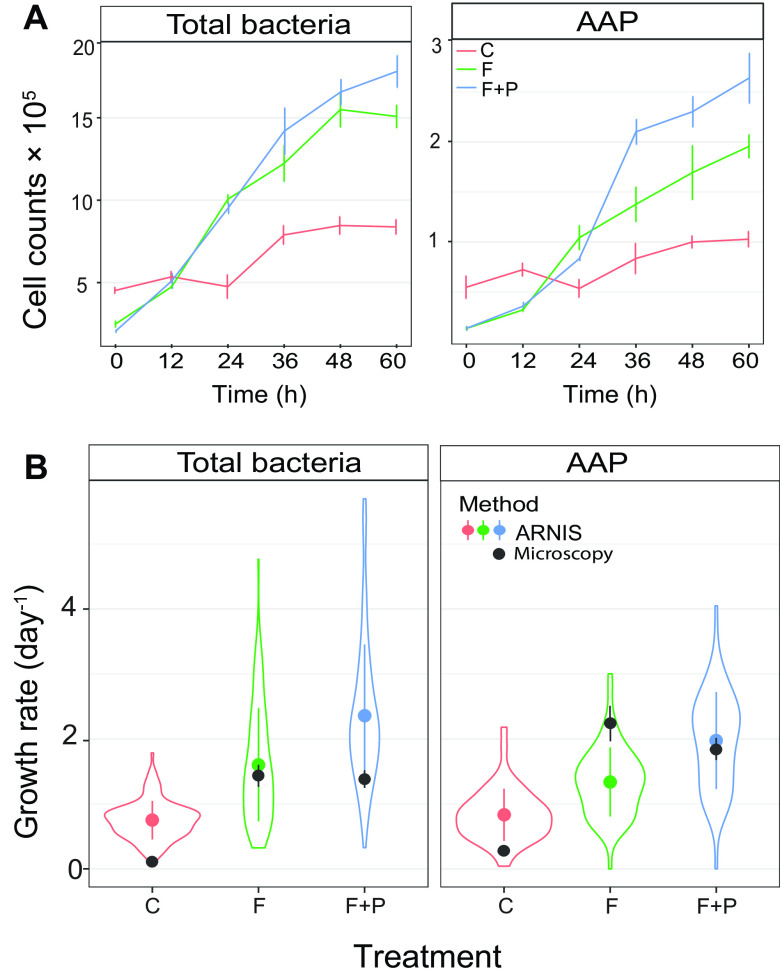
(A) Total and aerobic anoxygenic phototrophic (AAP) bacterial abundance in the control (C) and treatments (F, filtered; F+P, filtered and P-amended) based on microscopic counts. (B) Comparison of bacterial growth rates in the control and treatments based on ARNIS ratios (violin plots) and microscopy counts (black dots) in the total bacterial and AAP community. Dots with lines represent average value and standard deviation for both methods.

As verified by flow cytometry, 1.2-μm filtration removed over 95% of nanoflagellates but also removed most of the autotrophic phytoplankton and about one-half of the bacterial community (see Fig. S1 in the supplemental material). Grazer removal induced a rapid growth and resulted in a 6- and 8.5-fold increase in bacterial abundance in both the filtered (F) (from 0.26 × 10^6^ cells ml^−1^ ± 0.02 × 10^6^ cells ml^−1^ to 1.56 × 10^6^ cells ml^−1^ ± 0.11 × 10^6^ cells ml^−1^ at 48 h) and F+P (filtered and P-amended) treatment (from 0.22 × 10^6^ cells ml^−1^ ± 0.01 × 10^6^ cells ml^−1^ up to 1.79  × 10^6^ cells ml^−1^ ± 0.10 × 10^6^ cells ml^−1^ at 60 h). In contrast, a 2-fold increase was observed in the control (from 0.47 × 10^6^ to 0.85 × 10^6^ cells ml^−1^) (Fig. 1A). Initially (0 to 24 h), both grazer-free bacterial communities grew similarly by 1.41 ± 0.10 day^−1^ in the F and 1.36 ± 0.07 day^−1^ in the F+P treatment, compared with 0.14 ± 0.04 day^−1^ in the control (Fig. 1B). However, total bacteria in the P-amended treatment outpaced bacteria in the F treatment after 36 h. Proportions of high nucleic acid (HNA) cells also increased up to 80% of all bacterial cells in the first 24 h in both treatments, compared with the control where they remained around 40% throughout the experiment (Fig. S1).

10.1128/mSystems.00934-21.4 FIG S1 Changes in the microbial community in the treatments (F, filtered; F + P, filtered and P-amended; C, control) assessed by flow cytometry, as follows: total bacterial abundance (A), proportion of high nucleic acid (HNA) cells (B), abundance of *Synechococcus* (Syn) and picoeukaryotes (PE) (C), and abundance of heterotrophic nanoflagellates (D). Download FIG S1, PDF file, 0.03 MB. Copyright © 2021 Fecskeová et al. 2021 Fecskeová et al. https://creativecommons.org/licenses/by/4.0/ This content is distributed under the terms of the Creative Commons Attribution 4.0 International license.

Manipulation also resulted in major shifts in bacterial community composition. Alpha and beta diversity analyses of 16S rRNA libraries showed that community composition and diversity were comparable in the control and at the 0-h time point in the treatments (Shannon index, 4 to 5) but decreased upon manipulation (Shannon index, 1.5 to 2.5) (see Fig. S2 and S3 in the supplemental material). More than one-third of the OTUs either were removed directly by filtration or vanished from the community due to changes introduced by filtration by 24 h. These OTUs represented most of the *Cyanobacteria*, many *Bacteroidetes* orders, some *Proteobacteria* orders (*Caulobacterales*, *Sphingomonadales*, *Burkholderiales*, *Rhodobacterales*, and all *Salinisphaerales*), *Firmicutes*, *Planctomycetota*, *Verrucomicrobiota*, and *Actinobacteriota*.

10.1128/mSystems.00934-21.5 FIG S2 Alpha diversity analysis of the 16S rRNA (A) and aerobic anoxygenic phototrophs (AAP) (B) library employing the full library at ASV level, excluding singletons and the internal control. C, control; F, filtered; F + P, filtered and P-amended. Download FIG S2, PDF file, 0.5 MB. Copyright © 2021 Fecskeová et al. 2021 Fecskeová et al. https://creativecommons.org/licenses/by/4.0/ This content is distributed under the terms of the Creative Commons Attribution 4.0 International license.

10.1128/mSystems.00934-21.6 FIG S3 Bacterial community structure similarity (beta diversity) in the treatments and control assessed with a nonmetric multidimensional scaling (NMDS) analysis based on Bray-Curtis dissimilarities. C, control; F, filtered; F + P, filtered and P-amended. Download FIG S3, PDF file, 0.1 MB. Copyright © 2021 Fecskeová et al. 2021 Fecskeová et al. https://creativecommons.org/licenses/by/4.0/ This content is distributed under the terms of the Creative Commons Attribution 4.0 International license.

**FIG 2:**
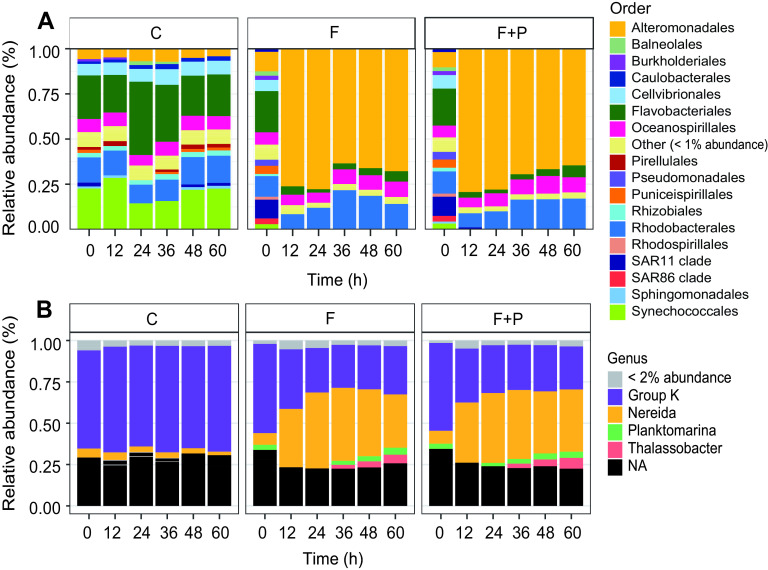
Bacterial community composition and its change in the control (C) and treatments (F, filtered; F+P, filtered and P-amended) at the order level in the 16S rRNA library (A) and genus level in the aerobic anoxygenic phototrophs (AAP) library (B), as average of three replicates in each library. NA, not assigned.

The main change after filtration was the rapid growth of members of genus *Glaciecola* (*Alteromonadaceae*), up to 80% of the community, in both treatments (Fig. 2A). *Bacteroidetes* (mainly *Flavobacteriaceae*) did not show increased growth in the F treatment (except for a few OTUs, like *Polaribacter* or NS3 marine group), but their abundance increased slightly in the P-amended treatment. Abundance of *Oceanospirillales* and *Rhodobacterales* increased gradually toward the end of the experiment. There was no significant difference in community composition between the two treatments (Fig. 2A).

### Growth curve reconstruction using the ARNIS method.

Based on amplicon read numbers normalized by an internal standard (ARNIS), we generated growth curves for 289 OTUs (see Fig. S4 in the supplemental material). Out of these OTUs, specific growth rates for >120 individual phylotypes were calculated for the period of exponential growth (0 to 24 h) (see Table S1 in the supplemental material).

10.1128/mSystems.00934-21.3 TABLE S1 Growth rates of individual OTUs in the total bacterial community (16S rRNA library) and ASVs in the AAP bacterial community (*puf*M library) calculated based on ARNIS ratios. Values represent average of the replicates. Download Table S1, XLSX file, 0.04 MB. Copyright © 2021 Fecskeová et al. 2021 Fecskeová et al. https://creativecommons.org/licenses/by/4.0/ This content is distributed under the terms of the Creative Commons Attribution 4.0 International license.

**FIG 3:**
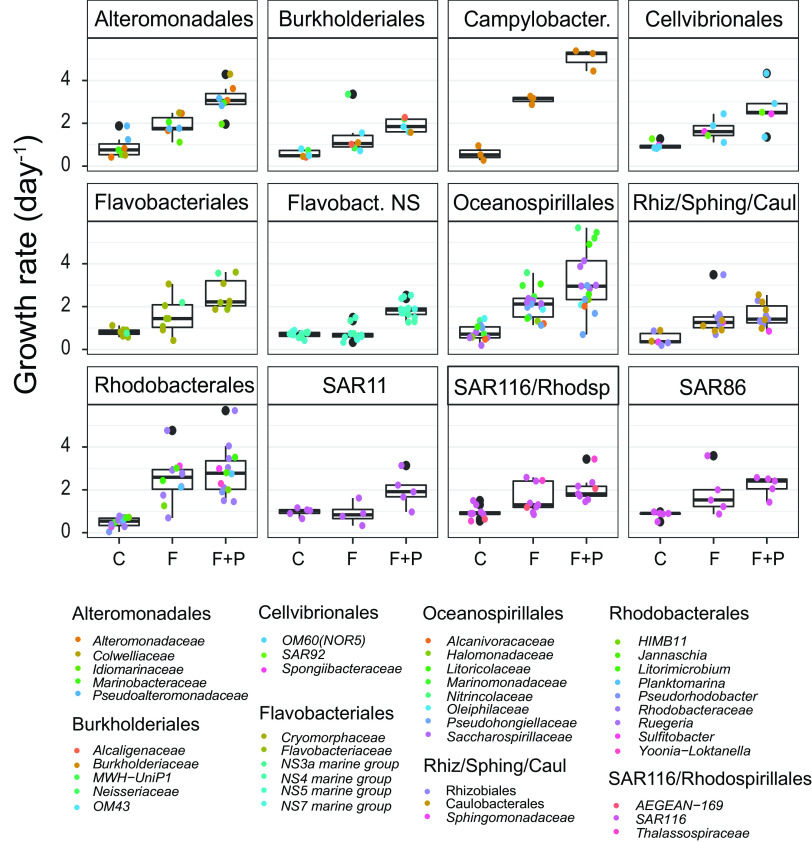
Growth rates of selected taxonomical groups calculated by ARNIS in the control (C) and treatments (F, filtered; F+P, filtered and P-amended). Dots represent individual OTUs within the given taxonomic group. Black dots represent outliers. Flavobact. NS, NS3, NS4, NS5, and NS7 marine groups of *Flavobacteriales*.

For the majority of OTUs, growth rates were substantially higher in the filtered treatments than in the control and usually the highest in the P-amended treatment. Bulk growth rates based on ARNIS were 1.60 ± 0.87 day^−1^ in the F and 2.36 ± 1.09 day^−1^ in the F+P treatment, compared with 0.71 ± 0.34 day^−1^ in the control (Fig. 1B). The distribution of individual growth rates in the treatments, especially in the P-amended treatment, was close to a normal distribution (Fig. 1B). The majority of phylotypes grew at rate between 0.6 and 3.5 day^−1^ in both treatments. The highest growth rates (>4 day^−1^ in the F treatment and >5 day^−1^ in the F+P treatment) were found among *Oceanospirillales* (*Marinomonas*, *Litoricola*, *Marinobacterium*, and *Saccharospirillaceae*), *Alteromonadales* (*Alteromonadacea* and *Colwelliaceae*), *Campylobacterales* (*Arcobacteracea*), and many *Rhodobacterales* (*Nereida*, *Litorimicrobium*, *Sulfitobacter*, and other unidentified *Rhodobacteracea*) (Fig. 3, see Fig S4 and Table S1 in the supplemental material).

**FIG 4:**
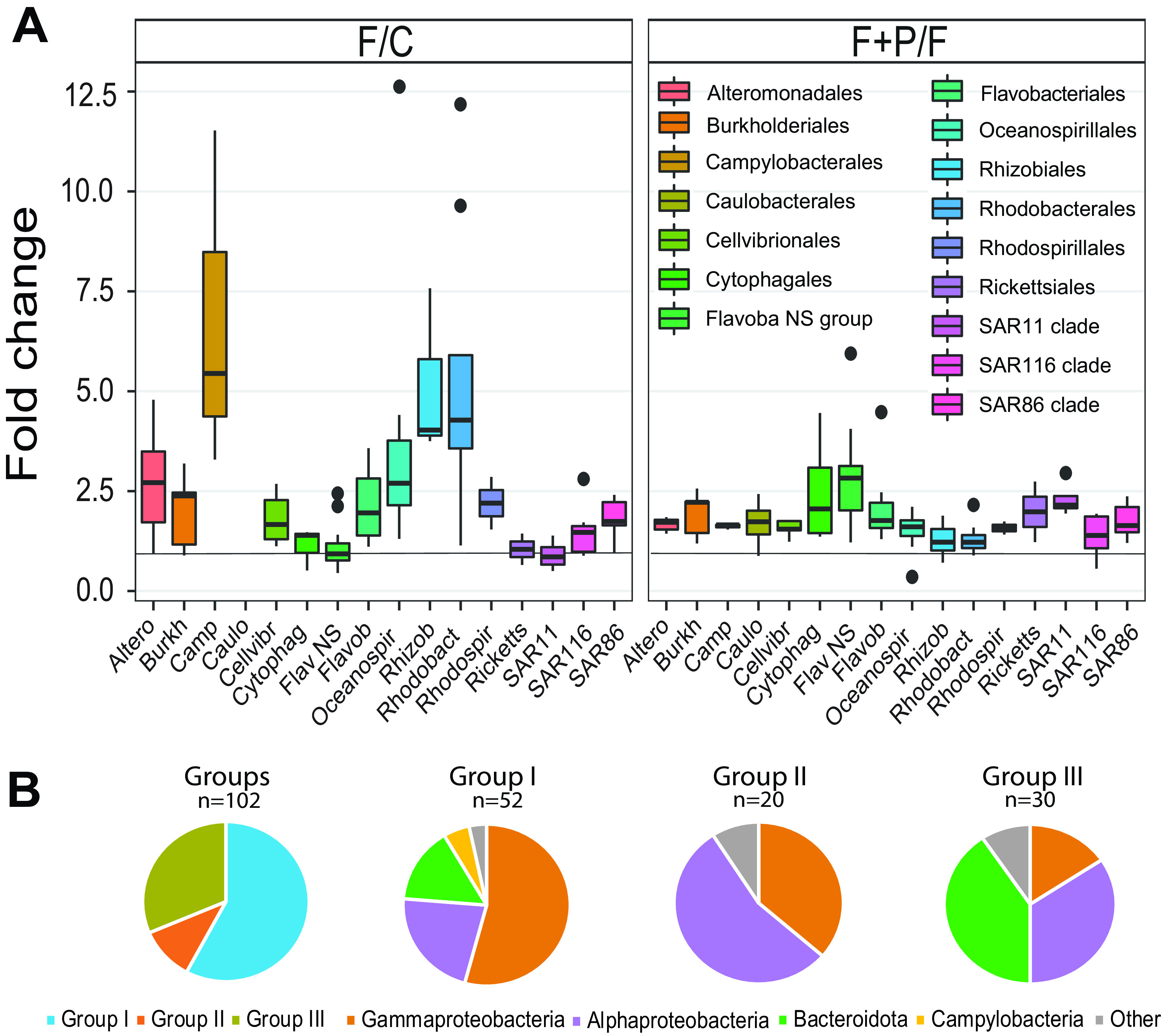
(A) Boxplot chart. Response of selected bacterial groups to manipulations, based on growth rates calculated by ARNIS, shown as the fold change of growth rates in the filtered (F) treatment versus control (F/C) and as the fold change of growth rates in the filtered and P-amended (F+P) versus F treatments (F+P/F). Vertical line indicates fold change of 1. (B) Pie charts. Response to manipulations by bacterial taxa. Groups, contribution of each group; group I, strong positive response to both filtration and P-amendment; group II, strong positive response to filtration and weak or no response to P-amendment; group III, weak or no response to filtration but strong positive response to P-amendment.

**FIG 5:**
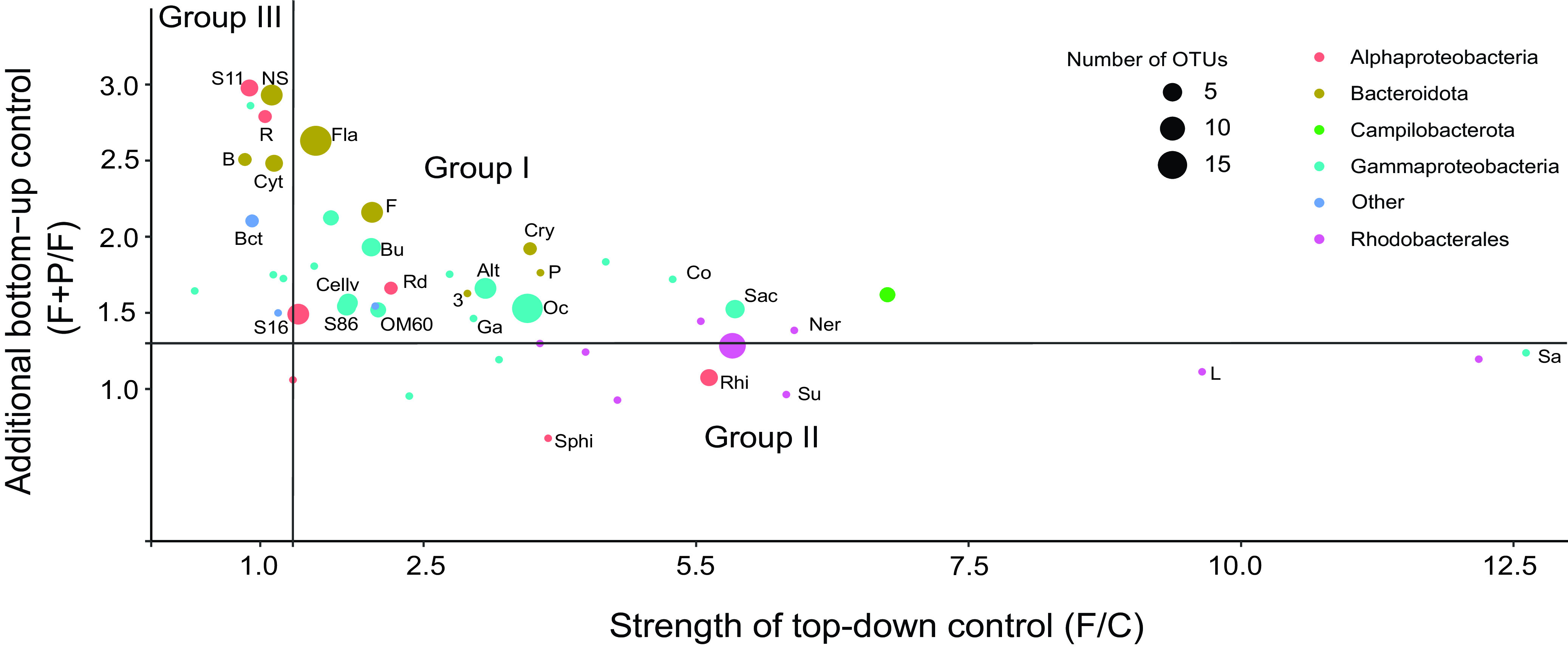
Distribution of fold change values of growth rates (F/C and F+P/F) of individual or averaged phylogroups (OTUs) mapped in the top-down (predation) versus bottom-up (phosphorus availability) framework. Alt, *Alteromonadales*; B, *Balneolales*; Bct, *Bacteriovoracales*; Bu, *Burkholderiales*; Cellv, *Cellvibrionales*; Co, *Colwellia*; Cry, *Cryomorphaceae*; Cyt, *Cytophagales*; F, *Flavobacteriales* without NS; Fla, all *Flavobacteriales*; Ga, *Glaciecola*; L, *Lentibacter*; NS, NS4, NS5, NS7 groups of *Flavobacteriales*; Ner, *Nereida*; Oc, *Oceanospirillales*; *P*, *Polaribacter*; R, *Rickettsiales*; Rhi, *Rhizobiales*; Rd, *Rhodospirillales*; Sa, *Saccharospirillum*; Sac, *Sacharospirillaceae*; Sphi, *Sphingomonadales*; Su, *Sulfitobacter*; S11, SAR11; S16, SAR116; S86, SAR86; 3, NS3 marine group (*Flavobacteria*).

Based on the response to the experimental manipulations, the individual OTUs could be divided into three groups. Group I represents OTUs that significantly responded to both grazer removal and P-amendment (OTUs affiliated mainly with *Gammaproteobacteria*, including *Alteromonadales*, *Oceanospirillales*, *Burkholderiales*, *Cellvibrionales*, and SAR86 clade; *Campilobacterales*, and some *Alphaproteobacteria*) (Fig. 4 and 5). Group II contains OTUs that responded significantly to grazer removal, but the P-amendment did not further enhance their growth or the response to P-amendment was weak (F+P/F fold change of growth rates, ≤1.3). Such a response was rare and limited to a few OTUs (*Sphingomonadales*, *Rhizobiales*, and *Rhodobacterales*) (Fig. 4 and 5). Group III encompass OTUs that exhibited only a small or no response to grazer removal (fold change of growth rates of F treatment versus control [F/C], ≤1.3) but were characterized by an increased growth rate in the P-amended treatment (*Bacteroidetes* dominated in this group, including *Cytophagales* and the NS4, NS5, and NS7 marine groups of *Flavobacteriaceae*; SAR11; *Rickettsiales*; and SAR116) (Fig. 4 and 5). The characteristics of each group with representative phylogenetic taxa and the relative strength of responses within a group are summarized in Table 2. The threshold 1.3 was identified as the 25th percentile of the distribution of fold change values (i.e., 25% of the phylotypes will be below the chosen threshold).

**TABLE 2:** Summary of groups identified by individual bacterial responses, with selected representative phylogenetic taxa belonging to each group and the strength of their relative responses to manipulations

Response to experimental manipulation	Representative phylogenetic taxa	Relative share in the total no. of OTUs by treatment at 24 h (%)			F/C	F + P/F
		C	F	F + P		
Group I: strong response to predator removal and a strong response to P-amendment	*Saccharospirillaceae*	0.05	2.98	4.45	5.36	1.52
	*Campylobacterales*	0.04	0.22	0.38	6.75	1.62
	*Polaribacter*	3.03	0.67	0.70	3.57	1.76
	*Oceanospirillales*	7.08	5.30	6.90	3.45	1.53
	*Alteromonadales*	7.27	77.51	77.16	3.07	1.90
	NS3a marine group	0.59	0.31	0.33	2.90	1.63
	*Spongiispira*	0.05	2.63	3.93	2.74	1.75
	*Rhodospirillales*	0.14	0.12	0.15	2.2	1.66
	OM60(NOR5)	7.31	0.59	0.54	2.08	1.52
	*Burkholderiales*	0.78	0.12	0.10	2.02	1.93
	Other *Flavobacteriales*	1.04	1.03	1.09	2.02	2.16
	*Pirellulales*	1.45	0.02	0.01	2.05	1.54
	*Cellvibrionales*	7.79	0.60	0.57	1.82	1.56
	SAR86	0.55	0.18	0.22	1.79	1.54
	*Caulobacterales*	2.08	0.02	0.02	NA	1.78
	*Flavobacteriales*, all	32.61	2.18	2.36	1.51	2.63
	KI89A clade	0.34	0.04	0.03	1.49	1.80
Group III: weak response to predator removal, strong response to P-amendment	SAR116^*a*^	1.09	0.24	0.25	1.35	1.49
	*Cytophagales*	0.24	0.03	0.03	1.13	2.48
	SAR92	0.26	0.00	0.02	1.12	1.75
	*Flavobacteriales*, NS groups	21.97	1.13	1.24	1.10	2.93
	*Rickettsiales*	0.11	0.02	0.01	1.04	2.79
	*Bacteriovoracales*	0.026	0.003	0.001	0.92	2.10
	SAR11 clade	0.97	0.46	0.57	0.90	2.98
	*Thiotrichales*	0.35	0.04	0.05	0.91	2.86
	*Balneolales*	1.53	0.10	0.13	0.86	2.51
Group II: strong response to predator removal and weak response to P-amendment	*Nereida* ^*a*^	3.04	8.69	7.98	5.90	1.39
	*Rhodobacterales*	12.60	12.27	10.71	5.33	1.28
	*Rhizobiales*	2.60	0.07	0.05	5.12	1.07
	*Sphingomonadales*	0.61	0.02	0.01	3.64	0.67

a Borderline groups.

**FIG 6:**
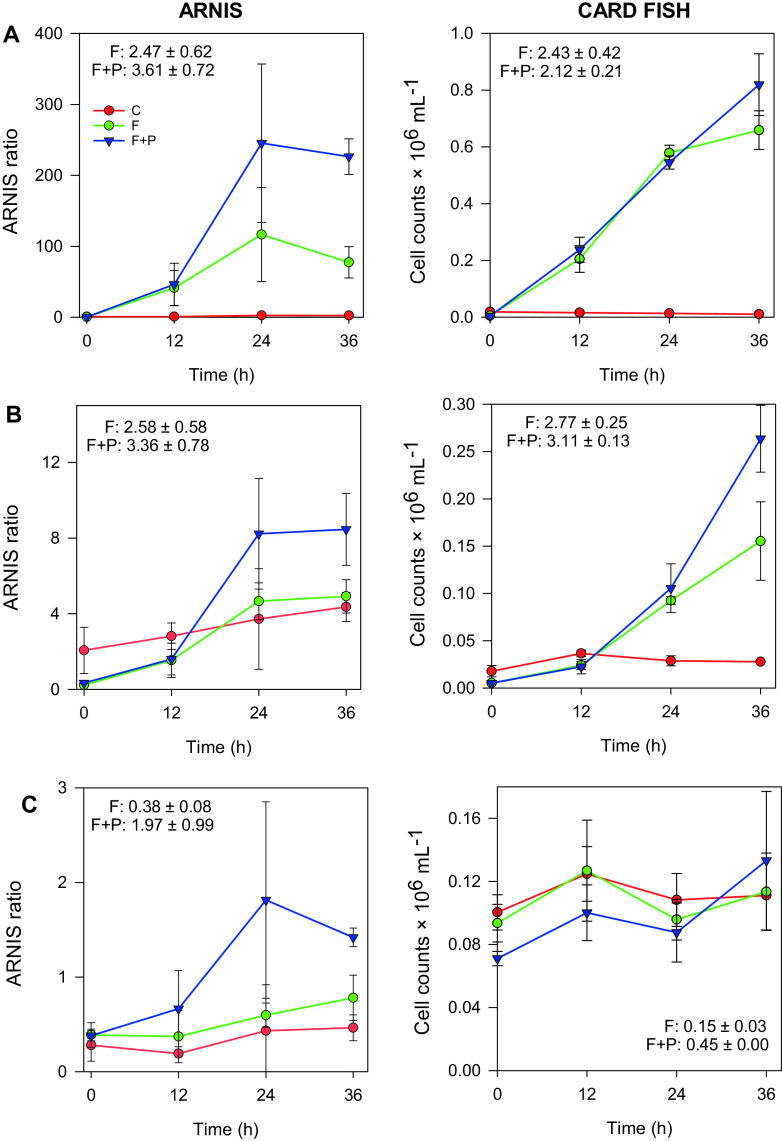
Comparison of growth curves of *Alteromonadaceae* (A), *Roseobacter* (B), and SAR11 clade (C) by ARNIS and CARD-FISH counts. Numbers in the graphs specified as F and F+P represent growth rates (day^−1^) in the respective treatments as average ± SD of 3 replicates. F, filtered treatment; F+P, filtered and P-amended treatment.

For three selected bacterial groups (*Alteromonadaceae*, *Roseobacter*, and SAR11 clade), CARD-FISH counts were determined for the comparison of growth rates. While growth rates based on ARNIS and CARD-FISH counts agreed well in the F treatment for all groups, in the F+P treatment, growth rates for SAR11 and *Alteromonadaceae* were substantially higher by ARNIS than by microscopy (Fig. 6).

### Effects of manipulations in the community of aerobic anoxygenic phototrophs (AAPs).

AAP bacteria initially represented 11% of the bacterial community. They were dominated by the *Gammaproteobacteria* group K and contained around 10% of *Rhodobacterales*. Approximately one-quarter of the community could not be identified (Fig. 2B). Prefiltration reduced AAP numbers from an initial 5.5 × 10^4^ ± 1.1 × 10^4^ cells ml^−1^ to 1.4  × 10^4^ ± 0.17 × 10^4^ cells ml^−1^. However, they rapidly increased from 5% to 13% of total bacteria in the F treatment and up to almost 15% in the F+P treatment. Bulk growth rates by epifluorescence microscopic counts in the first 24 h in the F treatment were higher (2.24 ± 0.20 day^−1^) than those in the F+P treatment (1.74 ± 0.09 day^−1^), compared with rates of the control (0.26 ± 0.06). (Fig. 1B). However, after 36 h, a higher increase in cell numbers in the F+P treatment than that of the F treatment was observed (Fig. 1A). Based on microscopic counts, growth rates of AAP bacteria were higher in all the treatments than those of heterotrophic bacteria (Fig. 1B).

**FIG 7:**
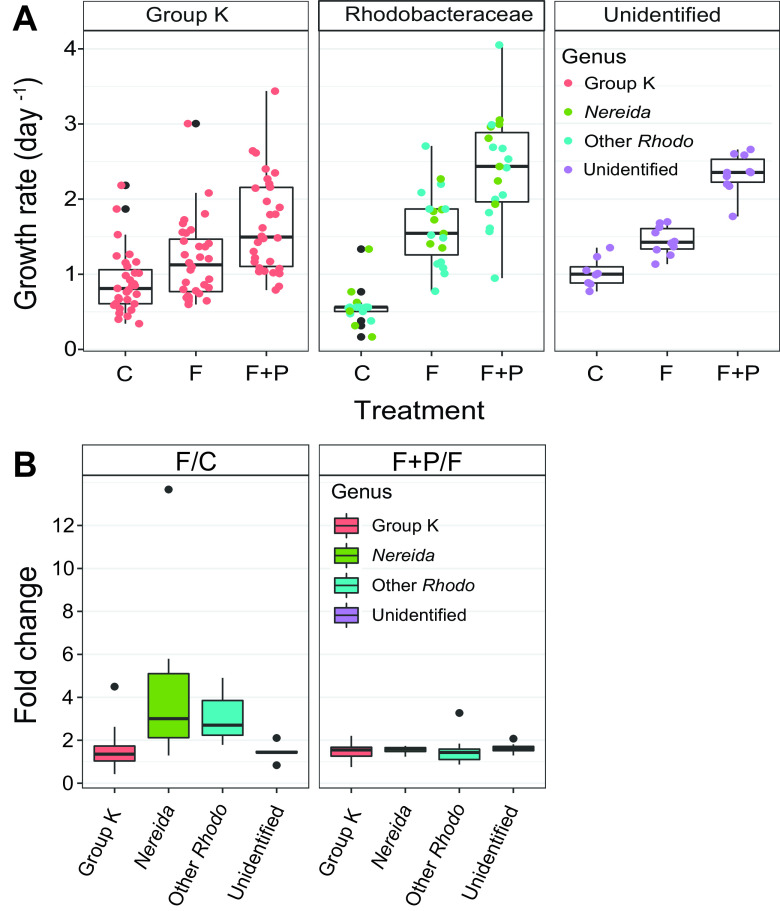
Growth response of aerobic anoxygenic phototrophic (AAP) bacteria calculated by ARNIS. (A) Growth rates of the three main AAP bacterial groups in the control and treatments. Dots represent individual ASVs within the given taxonomic group. (B) Response of taxonomical groups to manipulations, based on growth rates calculated by ARNIS, shown as the fold change of growth rates in the filtered (F) treatment versus control (F/C) and as the fold change of growth rates in the filtered and P-amended (F+P) versus F treatments (F+P/F). Black dots represent outliers. Rhodo, *Rhodobacteraceae*.

In total, 130 individual growth curves were reconstructed using ARNIS (see Fig. S5 in the supplemental material). Growth rates were calculated for ca. 60 amplicon sequence variants (ASVs) (Table S1). Average growth rates in the AAP community based on ARNIS were 1.34 ± 0.53 day^−1^ in the F and 1.98 ± 0.75 day^−1^ in the F+P treatment during the first 24 h, compared with 0.91 ± 0.45 day^−1^ in the control (Fig. 1B). The highest growth rates were observed for *Nereida*-related ASVs (*Rhodobacteraceae*) with an average growth rate 1.71 ± 0.32 day^−1^ and 2.63 ± 0.43 day^−1^ in the F and F+P treatment, respectively (Fig. 7A). *Rhodobacteraceae*, similarly to those in the 16S rRNA gene library, presented with a stronger response to grazer removal but a relatively weak response to P-amendment (Fig. 7B). On the contrary, members of the most abundant group K were growing slower and showed a weaker, but equal response to both manipulations by an approximately 1.4-fold increase (Fig. 7B).

10.1128/mSystems.00934-21.10 FIG S5 Growth curves of 130 phylotypes (ASVs) of aerobic anoxygenic phototrophic (AAP) bacteria (based on *puf*M gene library) in the control, filtered, and filtered + P-amended (phosphate) treatments. Growth curves were reconstructed in the R environment with package ggplot2 v3.3.5 using calculated values of ARNIS ratios (details in Materials and Methods). Growth curves here are shown as averaged values of ARNIS ratios of three replicates in each time point, with symbols representing the individual values of replicates. Note the different values on the *y* axis. Download FIG S5, PDF file, 0.4 MB. Copyright © 2021 Fecskeová et al. 2021 Fecskeová et al. https://creativecommons.org/licenses/by/4.0/ This content is distributed under the terms of the Creative Commons Attribution 4.0 International license.

Community composition upon manipulation changed according to the phylogroup-specific growth rates, as follows: fast-growing *Rhodobacteraceae*, especially *Nereida*-related ASVs, grew beyond 30% of the AAP community in the first 24 h, repressing the slowly growing members of group K to one-quarter of the community. Other *Rhodobacteraceae* genera, namely, *Planktomarina* and *Thalassobacter*, increased their populations toward the end of the experiment (48 to 60 h). This response was a little stronger in the P-amended treatment, but the differences between the treatments were not statistically significant (Fig. 2B).

## DISCUSSION

Many studies focusing on the activity of marine bacterioplankton report only the bulk growth rates of the entire bacterial community (1). Estimations of growth rates of a few broader taxonomic groups were previously reported that were usually based on microscopic FISH counts. Typically, target groups included *Gammaproteobacteria* and its subgroups *Alteromonadaceae*, NOR5/OM60, SAR86, the *Roseobacter* clade, and SAR11 of the *Alphaproteobacteria* and *Bacteroidota* (11, 13–19). Even though these studies provided valuable insights into community growth dynamics, the phylogenetic groups they targeted were limited in number and lacked finer taxonomic resolution.

Therefore, we applied the ARNIS approach to estimate growth rates of marine bacterioplankton at a fine-scale taxonomic resolution and analyzed their growth responses to experimental manipulations conducted in Adriatic coastal waters. We reconstructed growth curves of almost 300 OTUs in 2 experimental treatments (Fig. S4), and we assessed growth rates for over 120 OTUs in both treatments (Table S1). Additionally, we applied the ARNIS approach to the community of aerobic anoxygenic phototrophs, and we analyzed growth rates using the *puf*M functional gene. We reconstructed 130 growth curves (Fig. S5) and calculated growths rates of ca. 60 ASV-level phylotypes of AAP bacteria (Table S1). The results of the averaged phylogroup-specific growth rates are similar to growth rates from previous studies based mainly on CARD-FISH counts (11, 15, 17) or the most recent metagenome-based estimates (20).

Our analysis, namely, how the individual phylotypes responded to the top-down and bottom-up manipulation, identified three main groups (Fig. 5). The basic characteristic of the phylogenetic groups belonging to group I is the r-strategy (21, 22). This strategy is used by opportunistic species, which show a relatively weak or variable ability to compete with other species (23). The experimental manipulations meant a shift toward more favorable conditions for group I taxa, both due to the relaxation of the predation pressure and due to the increase in the amount of available phosphorus. OTUs affiliated with *Alteromonadales*, *Oceanospirillales*, and *Campilobacterota* dominated in Group I. *Alteromonadales* are also known to be grazed intensively (24, 25).

Phylotypes belonging to group III are opposite to those in group I in many traits, and the basic characteristics of the K-strategy can be recognized (21, 22). This strategy is used by equilibrium species that generally have slower growth, stable populations, and great competition ability. In our study, these groups were top-down controlled very poorly, meaning that unlike group I phylotypes, they were not selected by predators. Under the experimental conditions, group III included both oligotrophic, defense specialists represented by SAR11 (26, 27), SAR116 (28), or *Rickettsiales*, and slow-growing metabolic specialists, such as *Bacteroidota*, mainly NS5-NS4-related *Flavobacteriaceae* (29) (Fig. 5). As *Bacteroidota* prefer to use high-molecular-weight sugars and proteins derived from phytoplankton (30–32), elimination of these components by filtration might have negatively affected their growth in the F treatment. The results also suggest that the phylotypes within the group III are limited by phosphorus because the addition of phosphate significantly increased their growth rate (Fig. 3).

Phylotypes belonging to group II were very strongly controlled by predators in the control, which suggests a certain preference of predators toward these groups of bacteria. The removal of predators significantly increased the growth rates of these phylotypes. On the other hand, the addition of phosphate had only a minor effect on their growth rate, which indicates that these organisms were not significantly limited by phosphorus. Alternatively, colimitations by other nutrient(s) (for example, nitrogen) restricted their growth even under P-replete conditions (33). *Rhodobacterales*, including those in the AAP community, *Rhizobiales*, and *Sphingomonadales* were the only representatives of this group (Fig. 3, 4, and 5). *Rhodobacterales* are copiotrophs that are metabolically versatile and capable of rapid growth (34). They contain members belonging to the functional group of aerobic anoxygenic phototrophs (35–37), which are also identified in this experiment in the AAP library. Both the AAP and the *Roseobacter* clade are known to be highly susceptible to grazing (11, 38).

Through assessed growth rates of individual phylotypes, we could also study community responses to top-down and simultaneous top-down + bottom-up manipulations, which are two key factors controlling microbial community. Strong coupling between bacterial and heterotrophic nanoflagellate (HNF) abundance in the control indicated that bacteria were controlled by grazing (see Fig. S6 in the supplemental material). Top-down control is characteristic for oligotrophic environments (39–41) and was previously shown for the Kaštela Bay microbial community (42). Even though the original seawater at the sampling site, and more generally in the Adriatic Sea, is characterized by an all-year-round P-limitation (41, 43; total phosphorus here was 0.05 μM, giving a high N:P ratio of 150, Table 1), the addition of phosphate did not cause an immediate increase of microscopic counts. Interestingly, the response was first observed by ARNIS, as most of the OTUs had the highest growth rates in the P-amended treatment in the first 24 h (Fig. 3 and 7A). This positive effect of phosphate was reflected in the microscopic counts only after 36 h (Fig. 1A and 6). No substantial differences in community composition existed between the F and F+P treatments, which suggests that phosphate availability had only a secondary role in this community, as it supported growth but did not shape community composition (Fig. 2, see Fig. S7 in the supplemental material).

10.1128/mSystems.00934-21.9 FIG S4 Growth curves of 290 phylotypes (OTUs) in the total bacterial community (based on 16S rRNA gene) in the control, filtered, and filtered + P-amended (phosphate) treatments. Growth curves were reconstructed in the R environment with the package ggplot2 v3.3.5 using calculated values of ARNIS ratios (details in Materials and Methods). Growth curves are shown as averaged values of ARNIS ratios of three replicates in each time point, with symbols representing the individual values of replicates. Note the different values on the *y* axis. Download FIG S4, PDF file, 0.9 MB. Copyright © 2021 Fecskeová et al. 2021 Fecskeová et al. https://creativecommons.org/licenses/by/4.0/ This content is distributed under the terms of the Creative Commons Attribution 4.0 International license.

10.1128/mSystems.00934-21.7 FIG S6 Relationship between the log abundance of heterotrophic nanoflagellates (HNFs) and log abundance of bacteria in the treatments (F, filtered; F+P, filtered + P-amended) and control (C). The graph includes an empirically determined maximum attainable abundance (MAA) line depicting the HNF abundance that could be attained at a given bacterial abundance and a mean realized abundance (MRA) line. According to Gasol (39), points close to the MAA line (control C) indicate strong coupling between bacteria and HNF which can be interpreted as a strong top-down control on bacteria. Points which lie well below the MRA line indicate weak coupling and conditions when bacterial abundance was not controlled by HNF grazing (41). Download FIG S6, PDF file, 0.01 MB. Copyright © 2021 Fecskeová et al. 2021 Fecskeová et al. https://creativecommons.org/licenses/by/4.0/ This content is distributed under the terms of the Creative Commons Attribution 4.0 International license.

10.1128/mSystems.00934-21.8 FIG S7 Community composition changes within orders *Flavobacteriales* (A), *Oceanospirillales* (B), and *Rhodobacterales* (C) at the genus and ASV level in the treatments (F, filtered; F + P, filtered and P-amended; C, control). NA, not assigned. Download FIG S7, PDF file, 0.6 MB. Copyright © 2021 Fecskeová et al. 2021 Fecskeová et al. https://creativecommons.org/licenses/by/4.0/ This content is distributed under the terms of the Creative Commons Attribution 4.0 International license.

The importance of the grazing pressure was reflected also in rapid changes in the bacterial community composition in the treatments (Fig. 2). These changes were consistent with previous observations that certain phylotypes with a lower abundance are growing at higher rates than abundant phylotypes (7, 11). For example, the initially rare phylotypes of *Polaribacter* and the NS3a marine group were growing at exceptionally high rates for *Bacteroidota* (approximately 2 day^−1^) compared with bulk growth rates of *Bacteroidota* (on average 1 day^−1^) and eventually overgrew the most abundant NS5 and NS4 marine groups (growing at 0.8 day^−1^ on average) (Fig. S7A). Similar patterns were seen in *Rhodobacterales* and *Oceanospirillales* (Fig. S7) but also within the AAP community (Fig. 2B). The observed heterogeneity of responses of individual phylotypes within larger phylogroups shows that phylogenetically close bacterial taxa employ different life strategies. Moreover, the indicated changes within larger phylogroups also highlight the importance of rare phylotypes (especially noticeable for a couple of OTUs affiliated with *Oceanospirillales*, like *Marinobacterium* [OTU12] or *Spongiispira* [OTU7], or *Rhodobacterales*, such as OTU5 [ASV7]) (Table 2, see Fig. S4 and S7 in the supplemental material), which can readily respond to more favorable conditions and outcompete initially more abundant phylotypes (38, 44).

During our experiment, CARD-FISH counts were performed for a few selected groups, and their growth rates were calculated and compared with those by ARNIS. In the F treatment, we observed relatively good agreement between growth rates determined with both methods. However, in the F+P treatment, rates by ARNIS were higher than rates determined by microscopy for the *Alteromonadaceae* and SAR11 group (Fig. 6). The origin of this discrepancy could lie in the fact that ARNIS reflects the amount of 16S rRNA genes, whereas microscopy determines absolute cell numbers. The P-amendment probably caused a transient increase in DNA replication, ahead of cell division. This result can be seen in the CARD-FISH data, where the abundance in F+P treatment increased only at 36 h (Fig. 6). The same phenomenon of unbalanced growth has been observed before in marine bacterioplankton when an increase in assimilation of thymidine preceded the actual cell division (45).

The issue of unbalanced growth is more serious in slowly growing species with generation times comparable to the 24-h period used for growth rate analyses. Indeed, the largest discrepancy between growth rates determined by microscopy and ARNIS was found for the SAR11 group (Fig. 6C). In contrast, for the rapidly growing *Roseobacter* clade, which divided several times during the 24-h period (and were only moderately limited by phosphate), there was a good agreement between DNA synthesis and cell growth (Fig. 6B).

Growth rates of AAP bacteria have been so far determined only at the level of the complete community either by epifluorescence microscopy (11, 12, 46) or bacteriochlorophyll turnover rates (47, 48). In agreement with previous observations (11, 12), this study also detected higher bulk growth rates of AAP bacteria than those of total bacteria in all the treatments assessed by microscopy counts (Fig. 1B). The ARNIS approach revealed that the removal of grazers positively affected all the identified phylogroups in the AAP community, but not to the same extent. Phototrophic *Rhodobacteraceae* displayed the strongest response to grazer removal but a weaker response to P-amendment, consistent with the response of *Rhodobacteraceae* in the total community. In contrast, more abundant but slower growing *Gammaproteobacteria* responded to grazer removal as well as the additional P-amendment. This finding shows that significant differences exist in growth rates and phylotype responses even within functional groups, as with the AAP bacteria. This result is not surprising since AAP bacteria are a phylogenetically diverse group sharing only the ability to harvest light energy using bacteriochlorophyll-containing reaction centers.

In conclusion, in the presented study, we determined growth rates for a large number of marine bacterial phylotypes at a fine-scale taxonomic resolution. Distribution of growth rates in the total bacterial community was close to a normal distribution, suggesting that a common separation of marine bacteria to slowly growing specialists and rapidly growing opportunists may be incorrect. Most of the analyzed phylogroups were between these two contrasting characteristics. We identified different response patterns to experimental manipulations; however, most of the phylotypes were characterized by a clear stimulation by grazer removal and phosphate amendment.

The ARNIS approach was applied successfully on marine bacterioplankton, demonstrating its usefulness in studies that seek better insight into the activities of individual phylogenetic groups within bacterial assemblages (for example, studying cycling of elements and energy flow through marine food webs, the strength of trophic interactions between predators and individual prey type, diversity-stability relationships within microbial communities, and the importance of nutrient-limited versus energy-limited taxa in the functioning of the microbial food web).

Furthermore, we demonstrated that the ARNIS approach may also be applied with specific marker genes to analyze the growth response of selected functional groups of marine bacteria. A great potential of this method is in experiments investigating growth response of various functional groups which can be selectively targeted using specific marker genes. The use of functional genes together with 16S rRNA may significantly expand the scientific potential of the ARNIS method.

## MATERIALS AND METHODS

### Sampling site and experimental design.

The sampling site was in Kaštela Bay (43°31′N, 16°22′E), which is a semienclosed, shallow basin in the eastern part of the Middle Adriatic Sea. The river Jadro is the most important freshwater source, with an average annual inflow of 10 m^3^ s^−1^ (49).

Sixty liters of seawater collected at 0.5 m on 6 May 2019, at 3 p.m., was first prefiltered through 20-μm mesh and pooled in one container. The pooled water was dispensed into three transparent 8-liter polycarbonate bottles (Nalgene, USA) serving as the control (C). The remaining water was filtered using gentle vacuuming through 1.2-μm membrane filters and collected in a large container. The filtered water sample was distributed into other three Nalgene bottles (F treatment). Finally, the last three bottles were filled with 1.2-μm filtered water and amended with phosphate to the final concentration of 0.25 μM (F+P treatment).

All the bottles were incubated at the *in situ* temperature (16°C) in a large orbital shaker incubator (MRC, Holon, Israel) with gentle agitation (40 rpm) to enable sufficient gas exchange and prevent phytoplankton sedimentation. To avoid any interference of photosynthetic primary production in control samples, the experiment was conducted in the dark. Subsamples for DNA extraction, flow cytometry, and microscopy measurements were taken every ca. 12 h from all nine experimental bottles. The experiment was terminated after 60 h.

### Internal standard.

A freshwater AAP bacterium, Limnohabitans planktonicus II-D5, was used as the internal standard for both 16S rRNA and *puf*M genes. Cells were grown in organic medium as described previously (50). The internal standard was prepared from 1 ml of a *L. planktonicus* suspension fixed directly with 4 ml of absolute ethanol. The purity of the culture was tested with FISH-infrared (IR) (50). The resulting suspension (approximately 2 × 10^8^ cells ml^−1^) was aliquoted to 5 cryogenic tubes and stored at 4°C until used.

### Microbial community analysis.

For DNA extraction, 600- to 1,000-ml subsamples of the treatments or 200- to 300-ml subsamples of the controls were taken from the experimental bottles. The subsamples were spiked with the internal standard in the fixed ratio of 5 μl of internal standard per 100 ml of seawater sample. Samples were filtered onto polycarbonate filters (0.2-μm pore size, 47-mm diameter; Whatman Inc.). The filters were stored in cryogenic tubes containing zirconium beads, and tubes were deposited in liquid nitrogen. DNA from the filters was extracted using a phenol-chloroform protocol (51).

For the analysis of the total bacterial community, a 16S rRNA gene amplicon library was prepared from each sample using the Phusion high-fidelity DNA polymerase (Thermo Scientific, USA) and the 515F-Y and 926R primers (52) (see Table S2 in the supplemental material). The library was sequenced on the Illumina MiSeq platform in 2 × 300-bp reads at Macrogen, South Korea. The quality of the obtained sequences was checked using FastQC (53), and primers were trimmed using cutadapt v1.16 (54). The bioinformatic analysis of sequences was conducted in the R environment v4.0.2, using the packages DADA2 v1.16 (55) and phyloseq v1.32 (56). Forward and reverse reads were quality trimmed to 280 and 190 bp, respectively, at a maximum expected error rate of 2, allowing no Ns, using the function filterAndTrim [truncLen=c(280,190), maxN = 0, maxEE=c(2,2), truncQ = 2, rm.phix=TRUE]. Forward and reverse reads were merged using default parameters, and chimeras were removed with the method “consensus” using the function removeBimeraDenovo (method="consensus”). A total of 4,202 ASVs were obtained for the 16S rRNA gene library with an average number of reads per sample of 127,018 ± 15,026. ASVs were classified taxonomically using version 138 of the SILVA reference database (57) accessed in July 2020. For the analysis of lineage-specific growth curves, 2,596 OTUs were generated from the original ASVs using the packages speedyseq (github.com/mikemc/speedyseq) and DECIPHER (58) with complete linkage clustering at threshold 97% [aln <- DECIPHER::AlignSeqs(), d <- DECIPHER::DistanceMatrix(aln), clusters <- DECIPHER::IdClusters(d, method = “complete,” cutoff = 0.03)]. OTUs containing fewer than 10 observations in fewer than 15% of the samples and OTUs identified as chloroplasts were removed. This procedure reduced the numbers of OTUs by 89% but the number of sequences by only 2%.

10.1128/mSystems.00934-21.1 TABLE S2 Sequences of PCR primers and coverage of the 16S rRNA primers of selected groups. Download Table S2, PDF file, 0.1 MB. Copyright © 2021 Fecskeová et al. 2021 Fecskeová et al. https://creativecommons.org/licenses/by/4.0/ This content is distributed under the terms of the Creative Commons Attribution 4.0 International license.

For the analysis of the AAP community, a *puf*M gene amplicon library was prepared with primers pufM_uniF (59) and pufM_WAWR (60) (Table S2). A bioinformatic analysis of sequences was performed similarly to the 16S rRNA gene library, with differences stated below. Sequencing was conducted in 2 × 150-bp reads. Due to the high quality of the obtained reads, sequences were not further trimmed after primer removal. In total, 1,660 ASVs were obtained with 90,314 ± 9,568 sequences per sample. Further filtering of fewer than 3 observations in fewer than 15% of the samples reduced the number of ASVs to 130; however, only 0.4% of sequences were lost. ASVs were classified taxonomically using a custom-made *puf*M database containing >1,500 full-length or almost full-length *puf*M reference and environmental sequences (61).

Bacterial community analyses were performed in the R environment with the phyloseq package v1.32 (56) employing the full library (ASV level), excluding singletons and the internal control. Plots were visualized with the ggplot2 package v3.3.5 (62).

### ARNIS ratio, growth curves, and growth rates.

ARNIS ratios were calculated for each OTU in the 16S rRNA library (ASV in the *puf*M library) in each sample as the number of reads of a given OTU (ASV) divided by the number of reads of the internal control in the given sample, as follows: ARNIS ratio of an OTU = read number of OTU/read number of the internal control. By this method, we obtained values of ARNIS ratios for every OTU at every time point (0 to 60 h). Using these values, we reconstructed growth curves of each OTU (or ASV). Since the majority of the OTUs were growing exponentially, the growth rates of individual OTUs (or ASVs) were determined as the slope of the exponential fit over ARNIS ratios during the period of exponential growth (0 to 24 h). For a reliable growth rate calculation, regressions were considered only where the value of R^2^ of the fit was >0.6. For each replicate, individual regressions were calculated and the final value of a growth rate of an OTU represents their average.

### Catalyzed reporter deposition fluorescence *in situ* hybridization (CARD-FISH).

The abundance of three bacterial lineages, namely, *Alteromonadaceae* and *Colwelliaceae* (probe ALT1412 [13]), SAR11 clade (probe SAR11_441R [63]),, and *Rhodobacteraceae* (probe Ros537 [64]) were enumerated using CARD-FISH and epifluorescence microscopy. The hybridization followed the standard protocol of Wendeberg et al. (65). Samples (8 to 15 ml) were fixed with paraformaldehyde to a final concentration of 1% and filtered onto white polycarbonate filters (47-mm diameter, pore size of 0.22 μm; Isopore, Merck Millipore) that were stored at −20°C until analysis. Bacterial cells were digested with lysozyme (10 mg ml^−1^, 1 h at 37°C; Fluka, Steinheim, Germany) and hybridized with the horseradish peroxidase-labeled oligonucleotide probes (final concentration 50 ng μl^−1^; Biomers, Germany). For probe ALT1413, fluorescence signals were amplified with tyramides (Sigma) labeled with Alexa488 (MolecularProbes, Invitrogen) and counterstained with a Vectashield+Citifluor mixture containing 1 μg ml^−1^ of 4′,6-diamidino-2-phenylindole (DAPI). The preparations were evaluated using a Zeiss Axio Imager D2 epifluorescence microscope equipped with a Collibri LED system. Ten microphotographs were taken for every sample under a UV/blue emission/excitation channel for DAPI fluorescence and blue/green emission/excitation channel for Alexa488. They were counted by a semiautomatic procedure in the ACMEtool2 (technobiology GmbH, Switzerland) image analysis software. For probes SAR11-441R and ROS537, fluorescein isothiocyanate (FITC)-labeled tyramides (Perkin Elmer) were used to amplify CARD-FISH signals, and the samples were evaluated manually under 1,000× magnification using a Zeiss Axio imager M1 epifluorescence microscope equipped with a Collibri LED system. At least 700 DAPI-stained cells per sample were inspected. The cell abundance of all groups was calculated by multiplying the proportion of hybridized cells to DAPI-stained cells by bacterial abundance. For probe sequence and their coverage, see Table S3 in the supplemental material.

10.1128/mSystems.00934-21.2 TABLE S3 Sequences of CARD-FISH probes used in the study and their targets. Download Table S3, PDF file, 0.1 MB. Copyright © 2021 Fecskeová et al. 2021 Fecskeová et al. https://creativecommons.org/licenses/by/4.0/ This content is distributed under the terms of the Creative Commons Attribution 4.0 International license.

### Flow cytometry.

Flow cytometry was used to determine the abundance of autotrophic cells (66) and also the abundance of Sybr green-I-stained bacteria and heterotrophic nanoflagellates (67–69). For the flow cytometry count of autotrophic cells, 2 ml of preserved samples in 0.5% glutaraldehyde was frozen at −80°C and stored until analysis (5 to 10 days). Cyanobacterial cells were distinguished according to light scattering, red emission of cellular chlorophyll content, and orange emission of phycoerythrin-rich cells. The autotrophic community was processed on a Cytoflex cytometer. Samples used for the analysis of bacterial abundances and heterotrophic nanoflagellates were preserved in 2% formaldehyde and stored at 4°C until analysis (5 to 10 days). The heterotrophic samples were processed on a Beckman Coulter EPICS XL-MCL instrument with a high flow rate from 1 to 1.2 μl s^−1^, and the analyzed volume was calculated according to the acquisition time.

### Infrared epifluorescence microscopy.

Total and AAP bacteria were enumerated using the epifluorescence microscope Olympus BX51 and a XM10-IR camera. Subsamples were fixed with 2% formaldehyde (0.2 μm prefiltered), incubated in the dark for 30 to 60 min at room temperature, and stored at −80°C. Cells were collected by filtration through 0.2-μm pore size polycarbonate (PC) filters (Whatman), dried, and stained with DAPI using a 3:1 mixture of Citifluor AF1 and Vectashield. AAP bacteria were identified based on autofluorescence in the near-infrared region (70). Three images were acquired in the same field of view and overlapped afterward. DAPI emission was recorded first, and IR and chlorophyll *a* (Chl *a*) were acquired subsequently. Chlorophyll signal was subtracted from the IR image, due to a weak emission tail of Chl *a* in the IR area, to obtain an exact count of bacteriochlorophyll *a* (BChl *a*) cells.

### Data availability.

Raw reads of the 16S rRNA and the *puf*M amplicon libraries have been deposited in the NCBI Sequence Read Archive (SRA) under accession number PRJNA742048.
