# Supplementary figures and images for: Lineage-Specific Growth Curves Document Large Differences in Response of Individual Groups of Marine Bacteria to the Top-Down and Bottom-Up Controls
Source: mSystems. 2021 Sep 28;6(5):e00934-21. doi: 10.1128/mSystems.00934-21 (PMC8547455; doi:10.1128/mSystems.00934-21)

**A**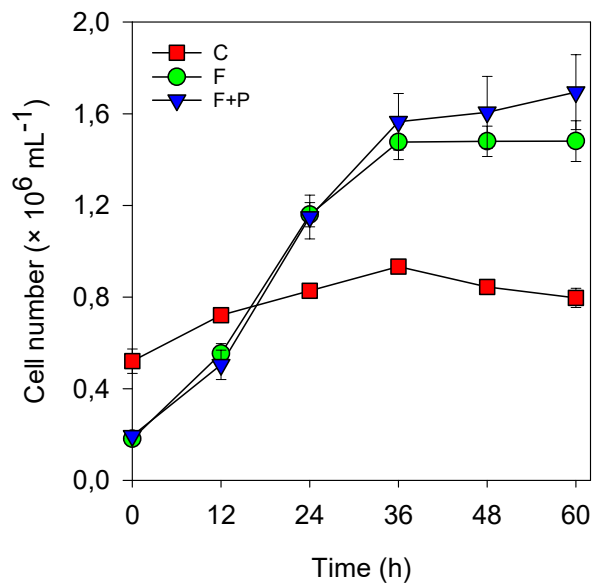**B**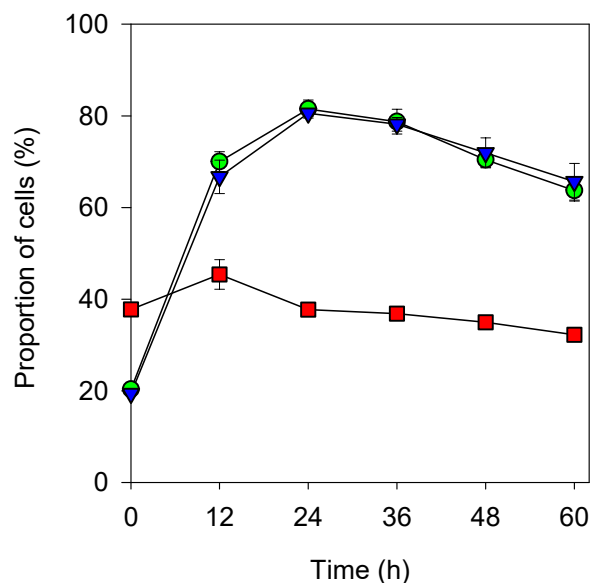**C**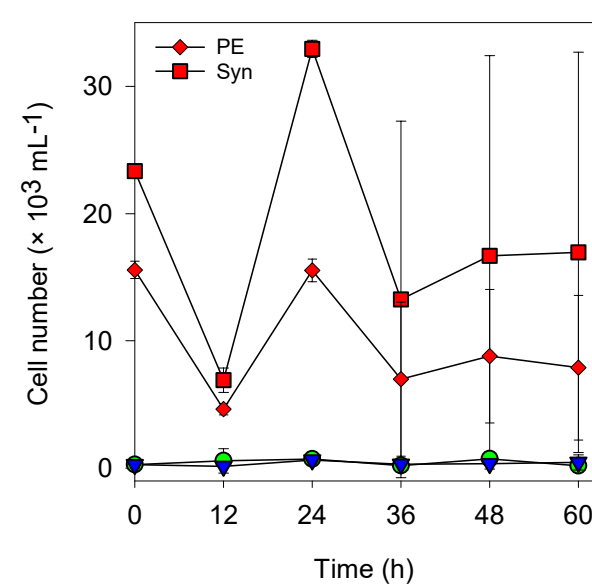**D**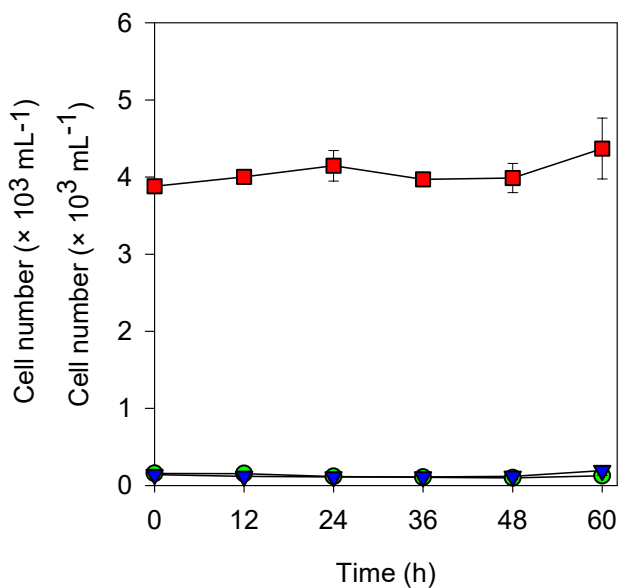

Supplement: FIG S1 [file msystems.00934-21-sf001.pdf]

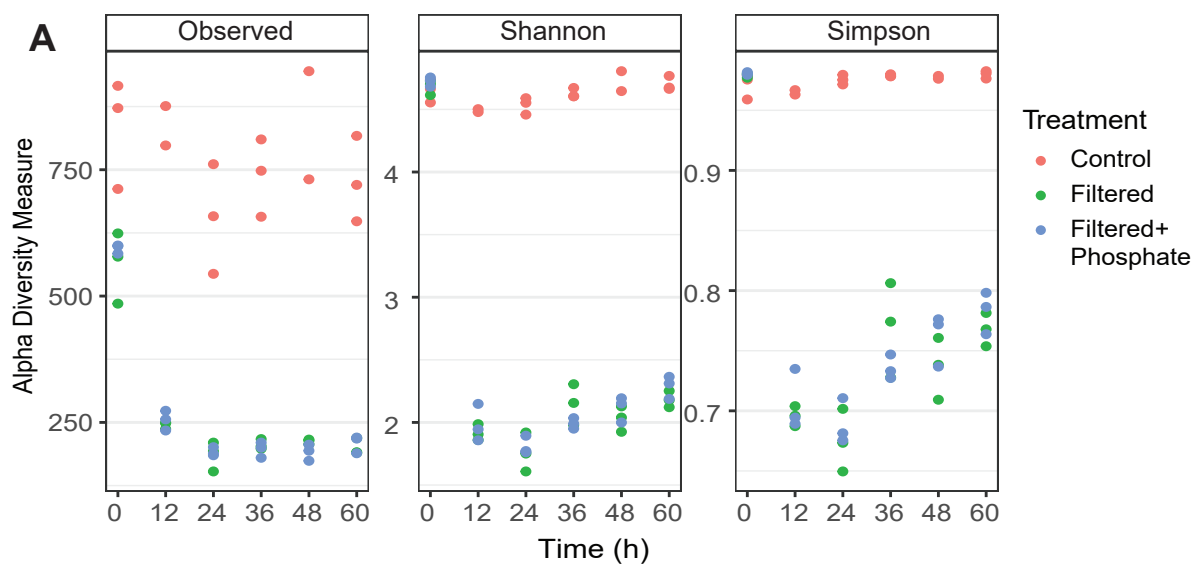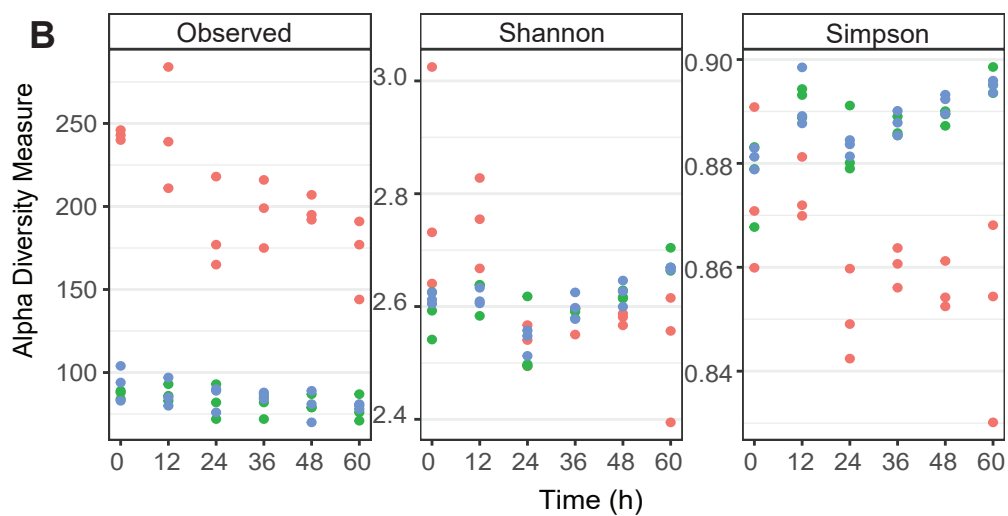

Supplement: FIG S2 [file msystems.00934-21-sf002.pdf]

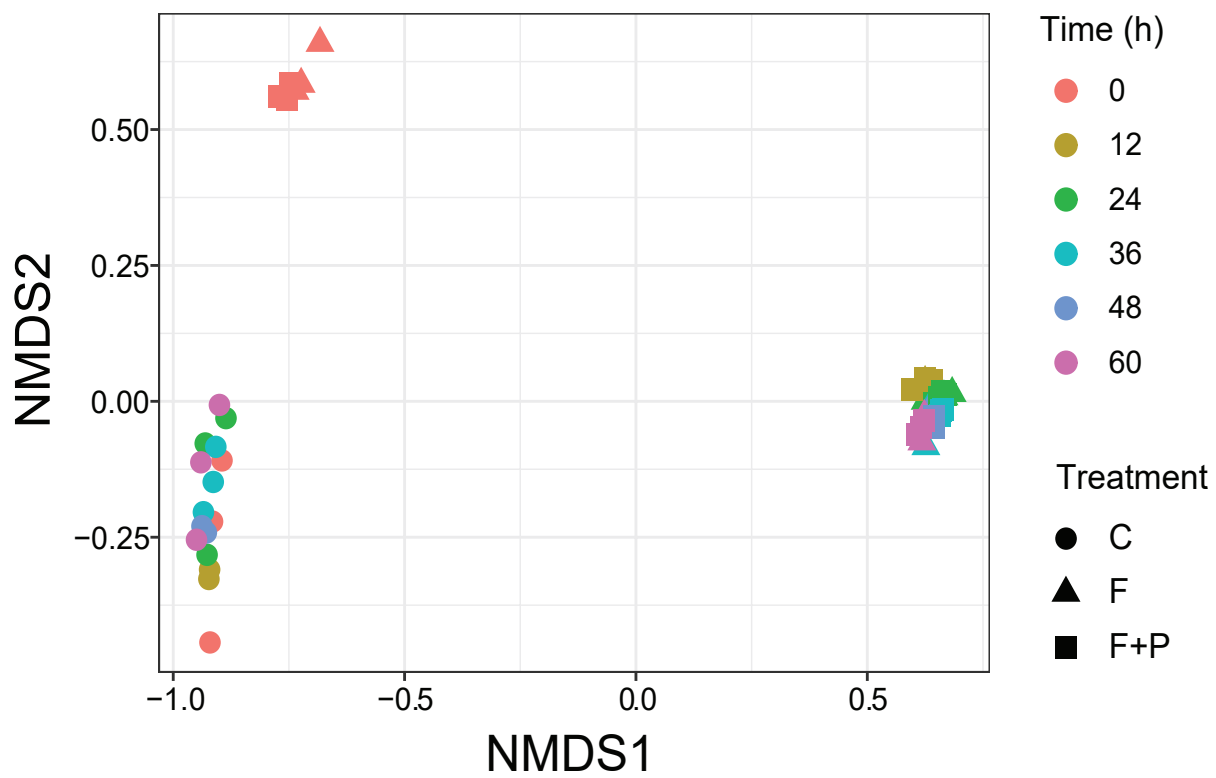

Supplement: FIG S3 [file msystems.00934-21-sf003.pdf]

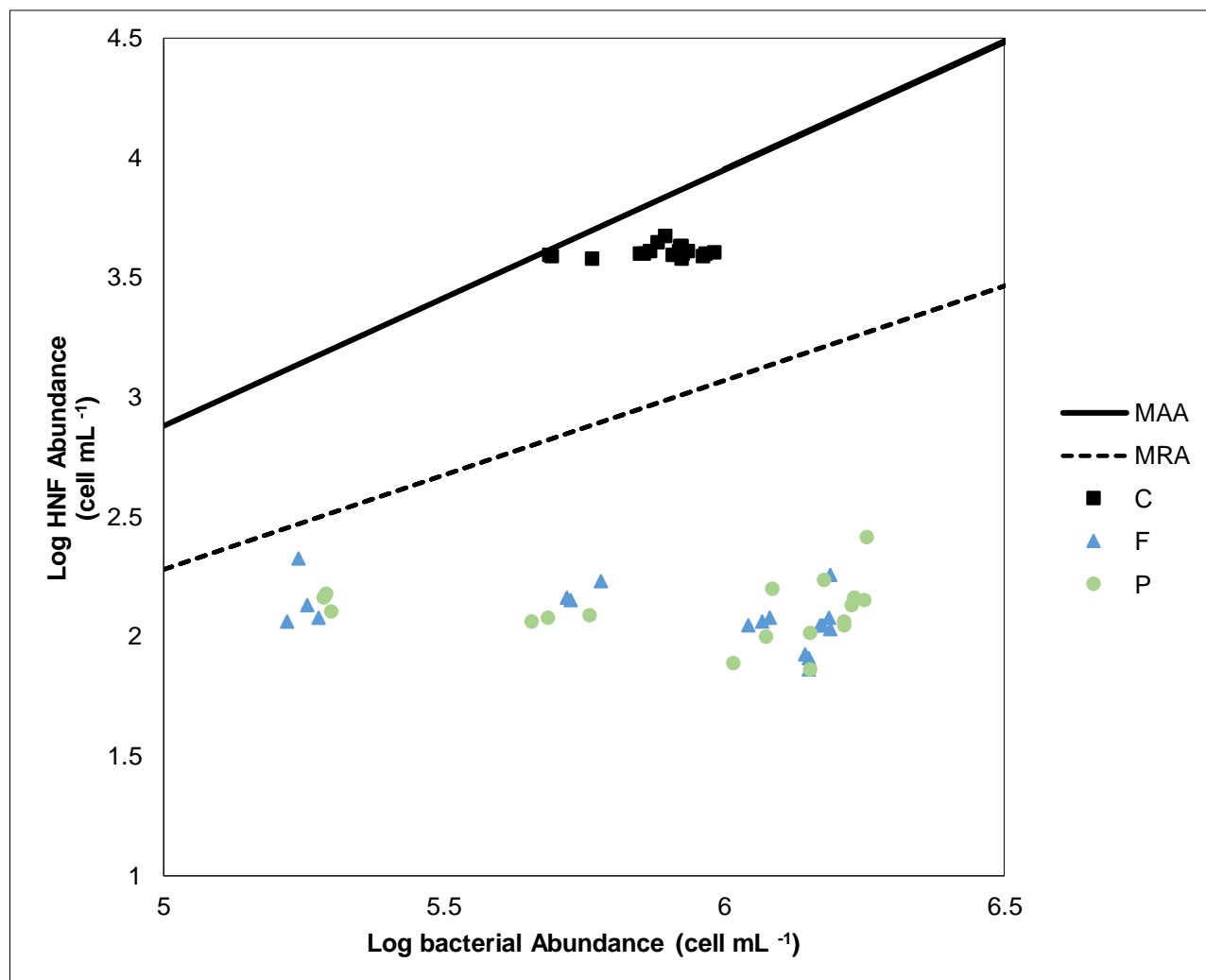

Supplement: FIG S6 [file msystems.00934-21-sf006.pdf]

Relative abundance of

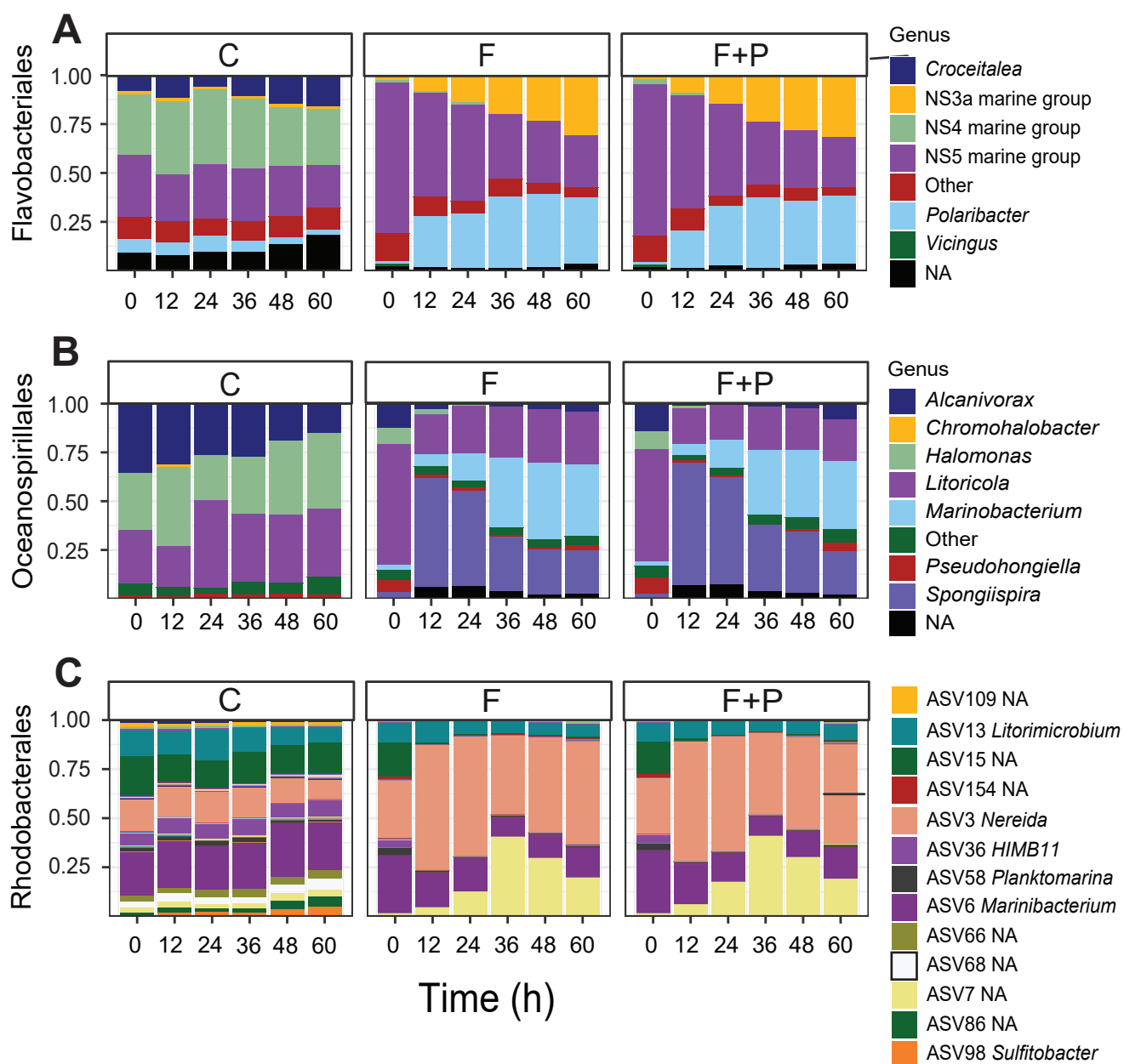

Supplement: FIG S7 [file msystems.00934-21-sf007.pdf]
