# Supplementary material for: Lineage-Specific Growth Curves Document Large Differences in Response of Individual Groups of Marine Bacteria to the Top-Down and Bottom-Up Controls
Source: mSystems. 2021 Sep 28;6(5):e00934-21. doi: 10.1128/mSystems.00934-21 (PMC8547455; doi:10.1128/mSystems.00934-21)
Supplement: FIG S5 [file msystems.00934-21-sf005.pdf]

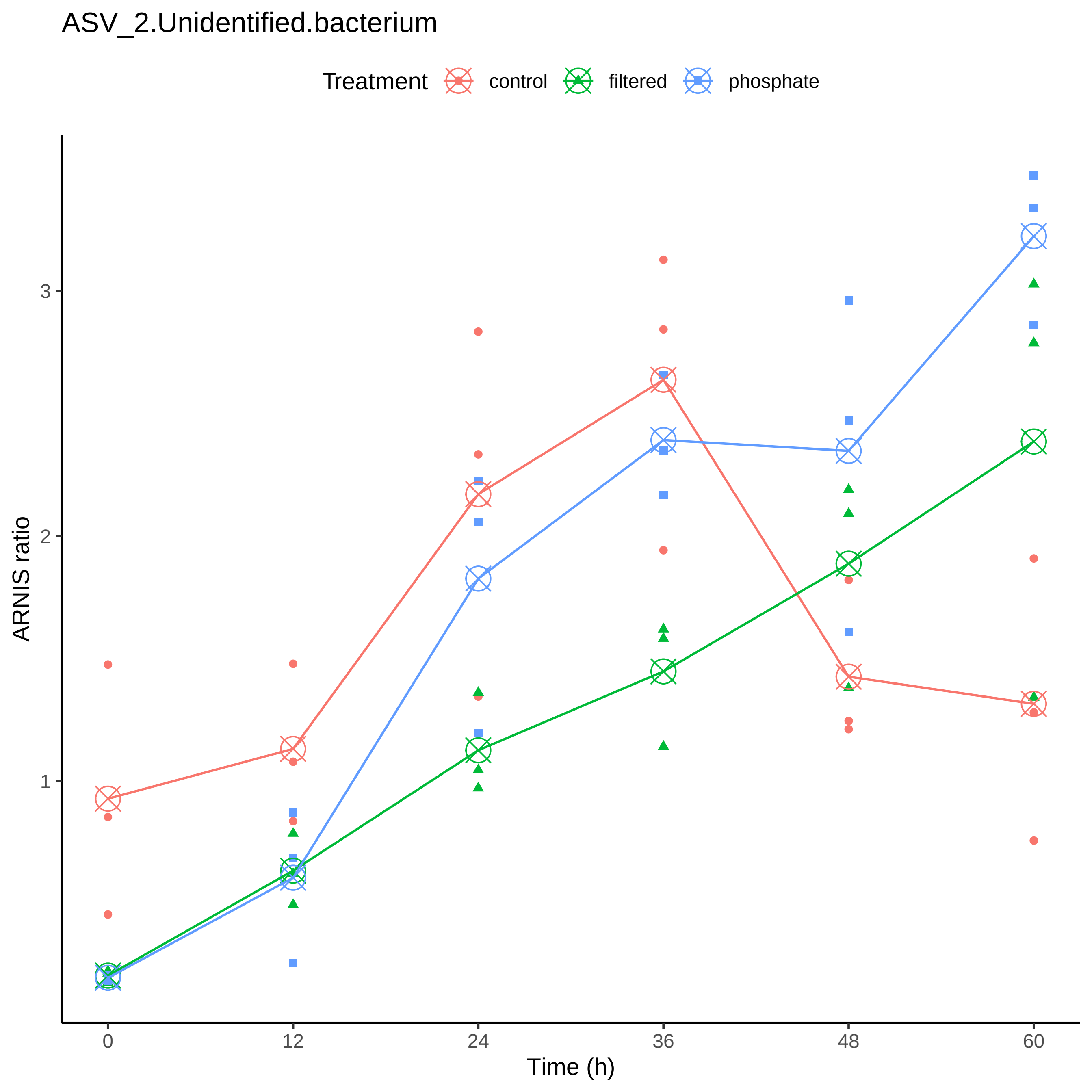

ASV_2.Unidentified.bacterium
Treatment
control
filtered
phosphate
ARNIS ratio
1
2
3
Time (h)
0
12
24
36
48
60

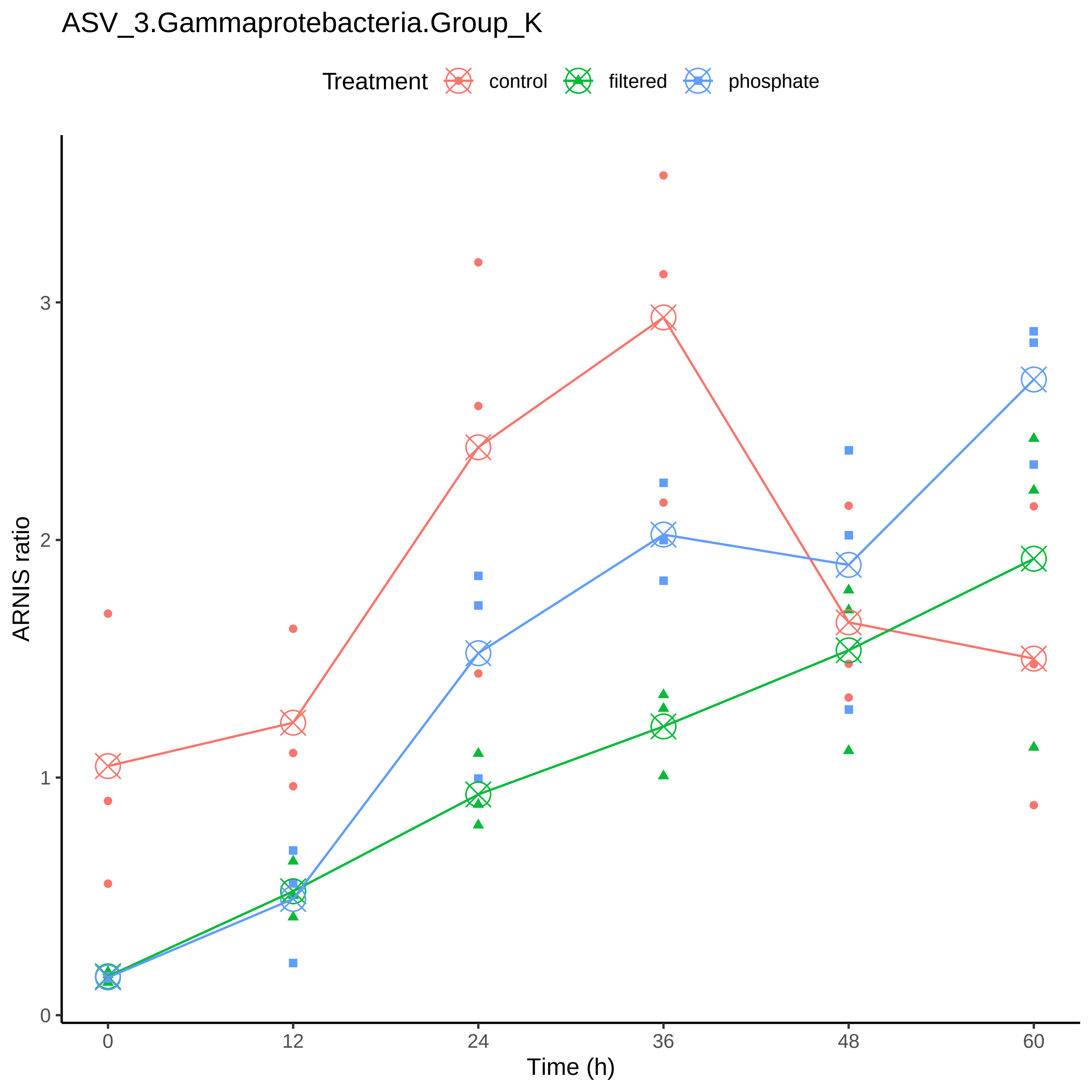
ASV_3.Gammaprotebacteria.Group_K
Treatment
control
filtered
phosphate
ARNIS ratio
0
1
2
3
Time (h)
0
12
24
36
48
60

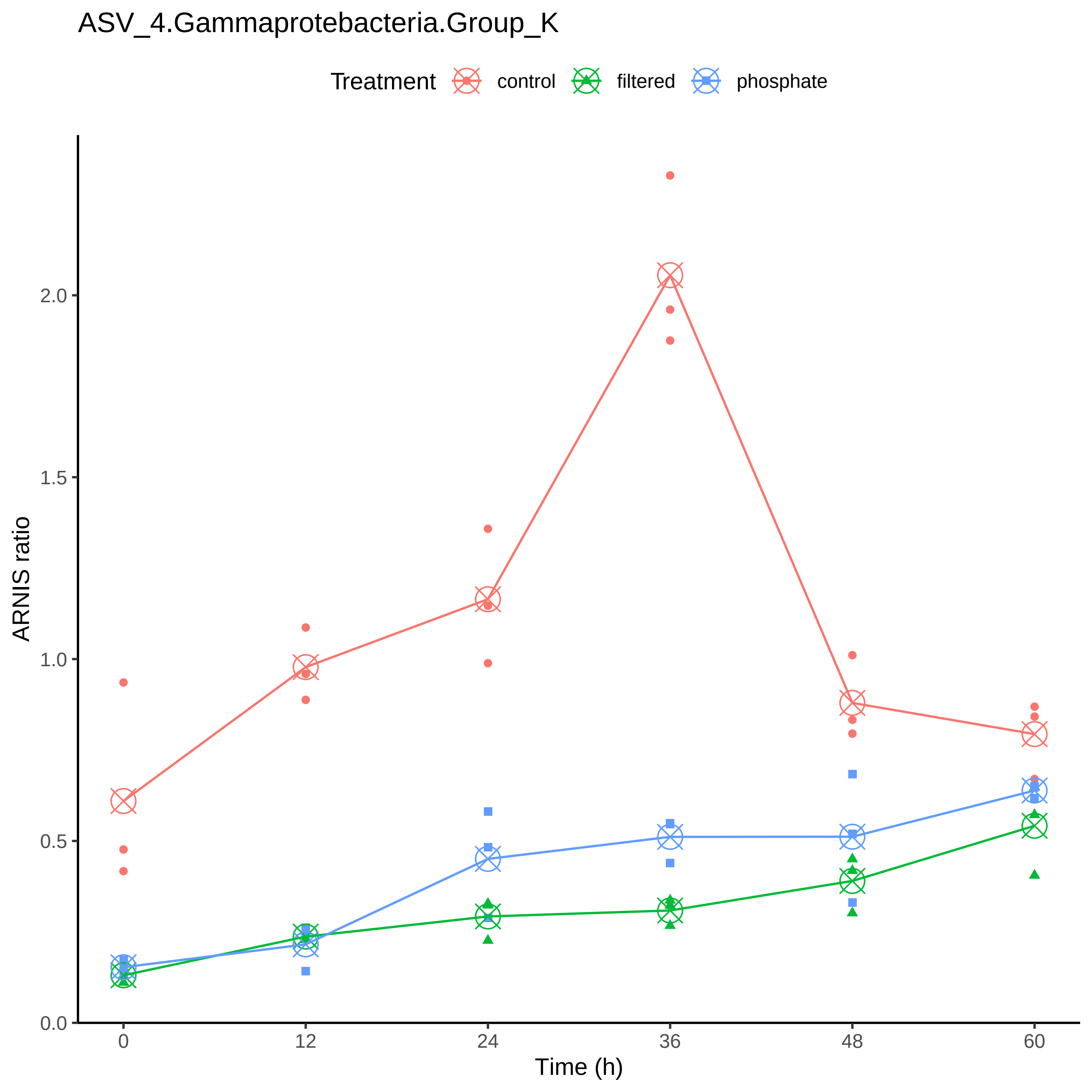

ASV_4.Gammaprotebacteria.Group_K
Treatment
control
filtered
phosphate
ARNIS ratio
0.0
0.5
1.0
1.5
2.0
Time (h)
0
12
24
36
48
60

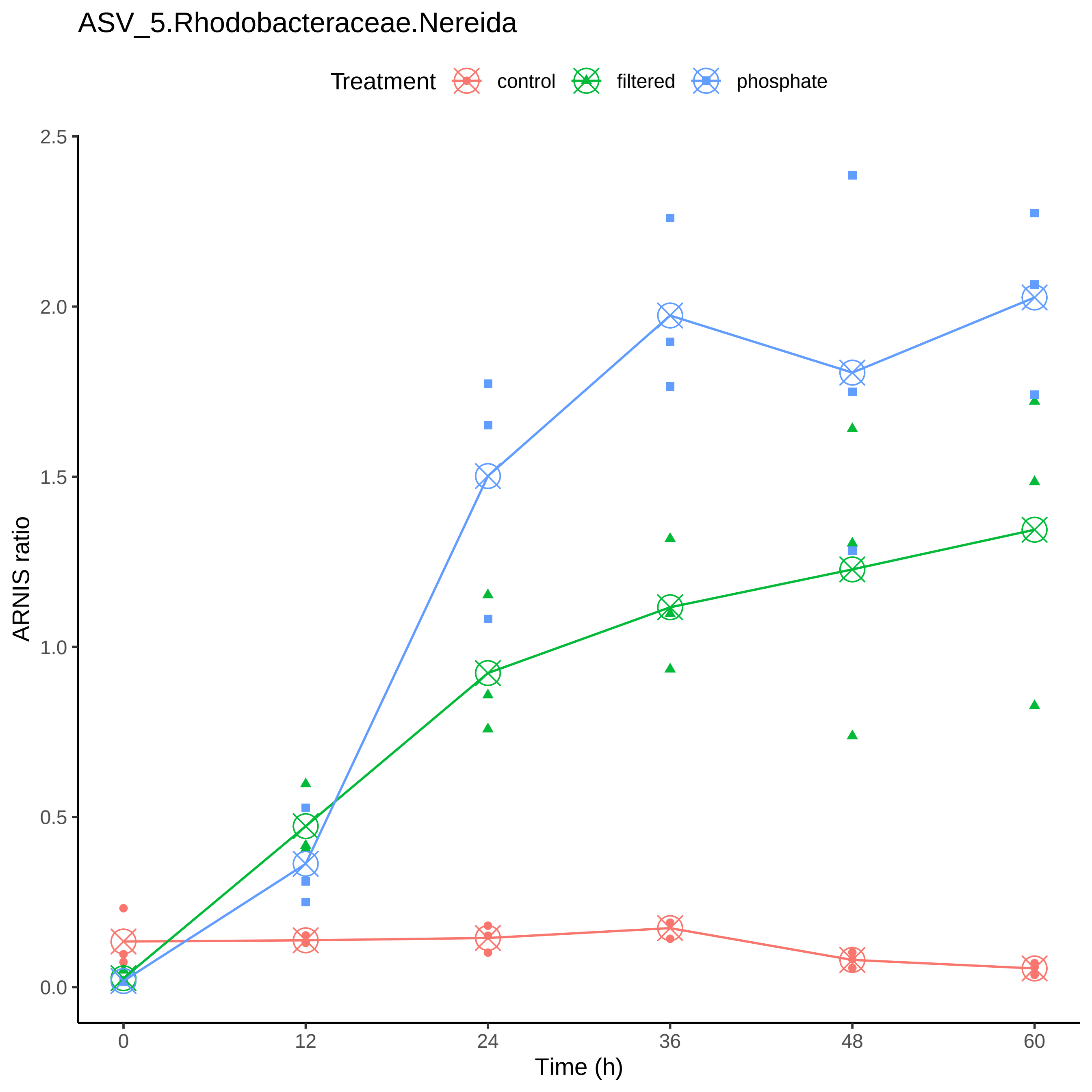
ASV_5.Rhodobacteraceae.Nereida
Treatment
control
filtered
phosphate
ARNIS ratio
2.5
2.0
1.5
1.0
0.5
0.0
0
12
24
36
48
60
Time (h)

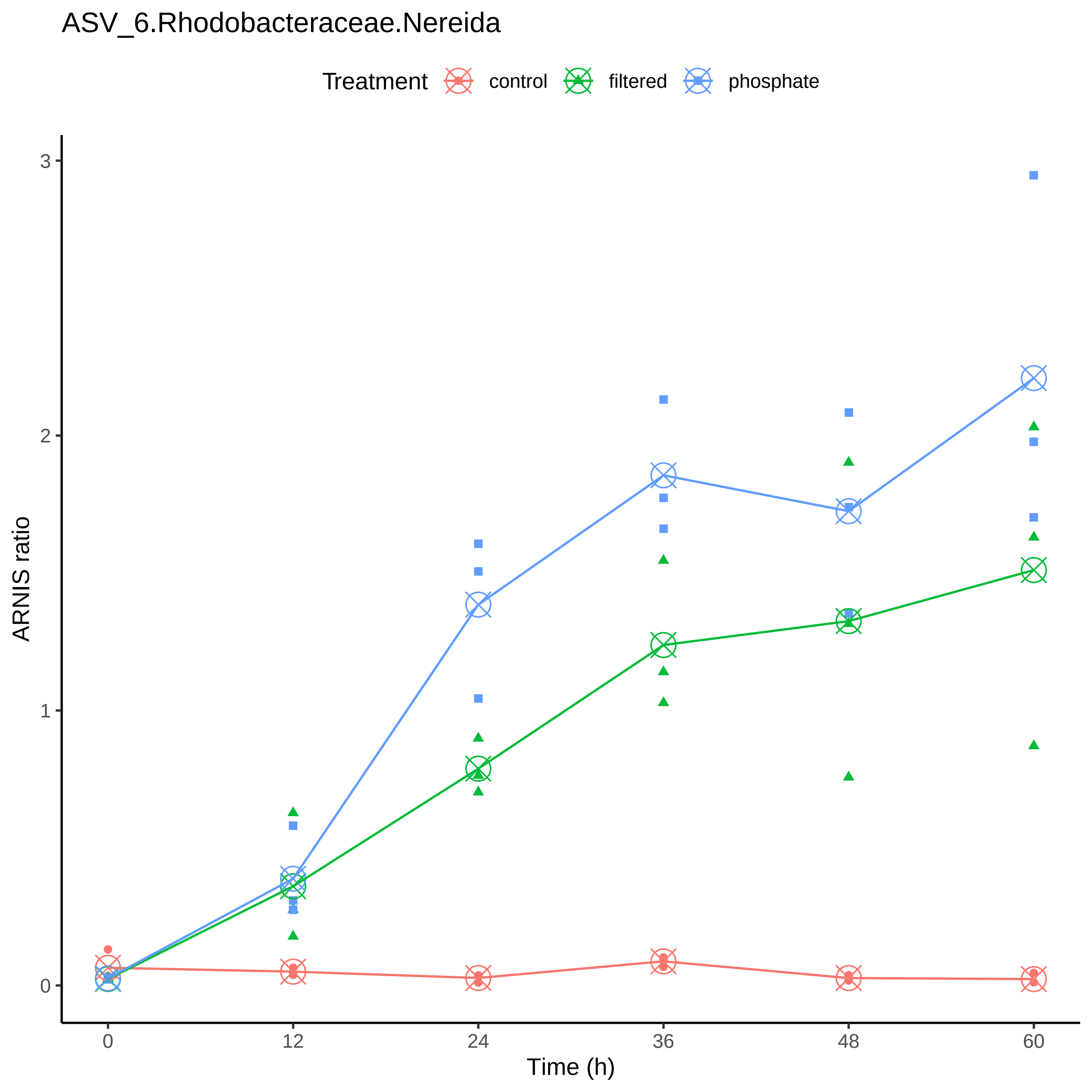

ASV_6.Rhodobacteraceae.Nereida
Treatment
control
filtered
phosphate
ARNIS ratio
0
1
2
3
Time (h)
0
12
24
36
48
60

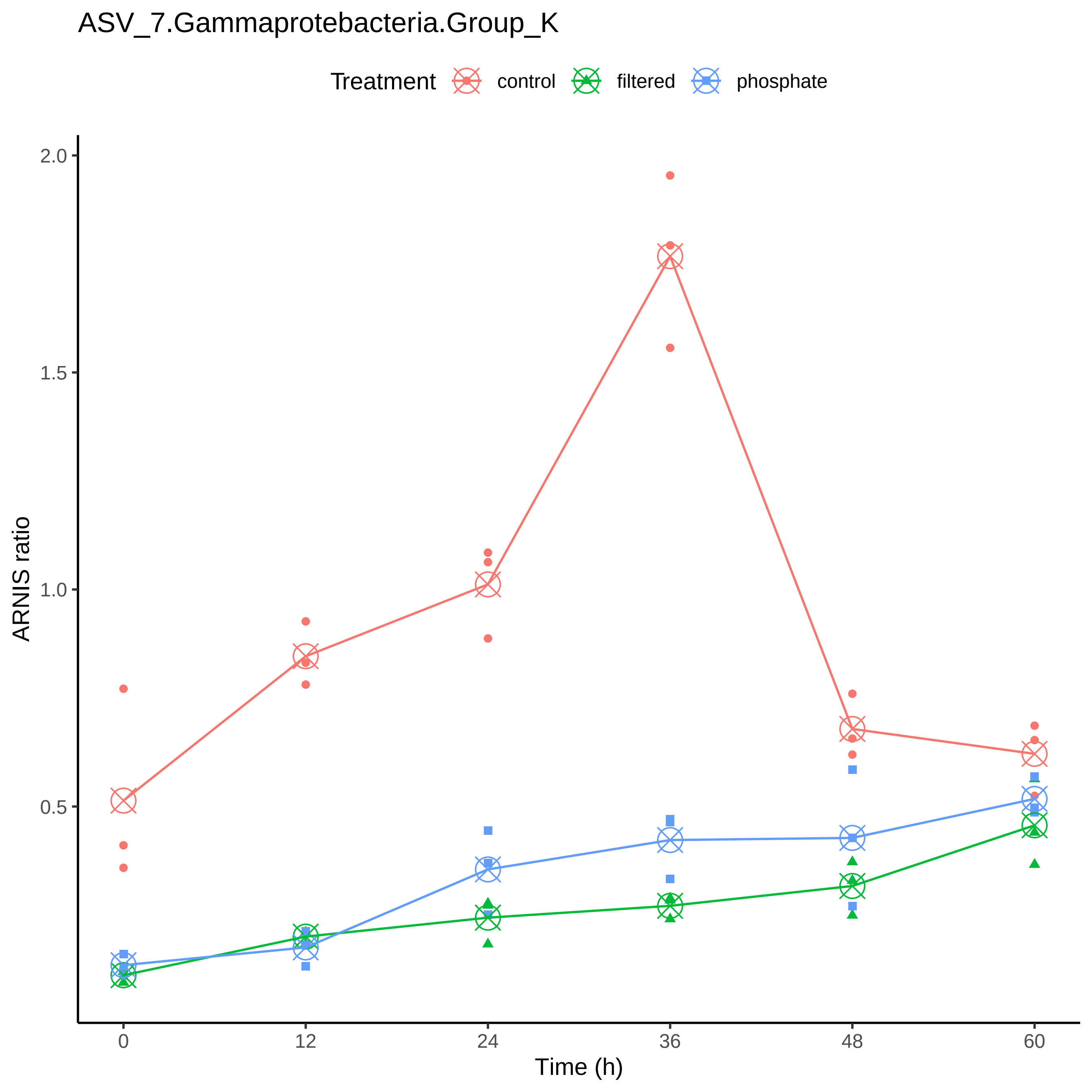

ASV_7.Gammaprotebacteria.Group_K
Treatment
control
filtered
phosphate
ARNIS ratio
2.0
1.5
1.0
0.5
0
12
24
36
48
60
Time (h)

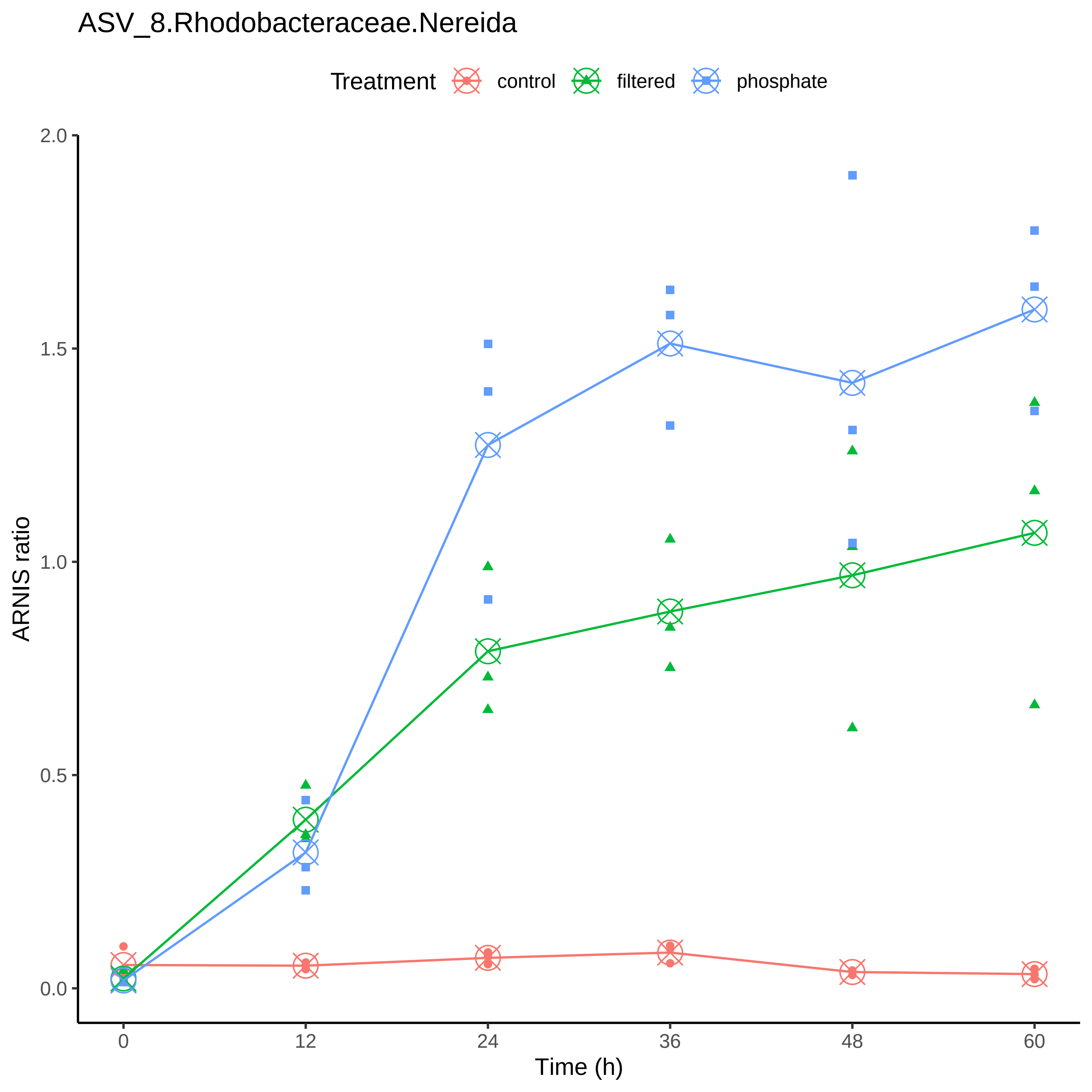

ASV_8.Rhodobacteraceae.Nereida
Treatment
control
filtered
phosphate
ARNIS ratio
2.0
1.5
1.0
0.5
0.0
0
12
24
36
48
60
Time (h)

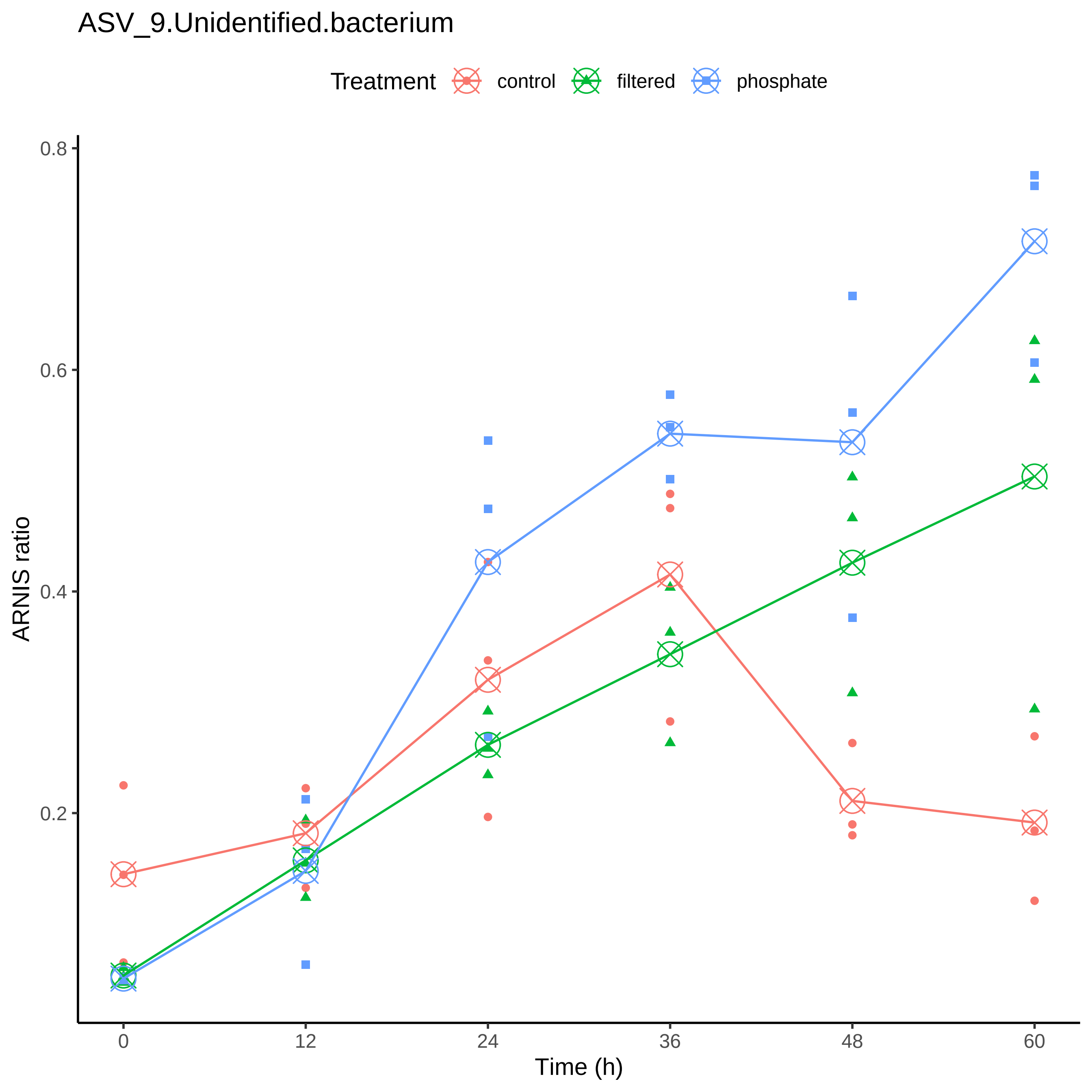

ASV_9.Unidentified.bacterium
Treatment
control
filtered
phosphate
ARNIS ratio
0.2
0.4
0.6
0.8
0
12
24
36
48
60
Time (h)

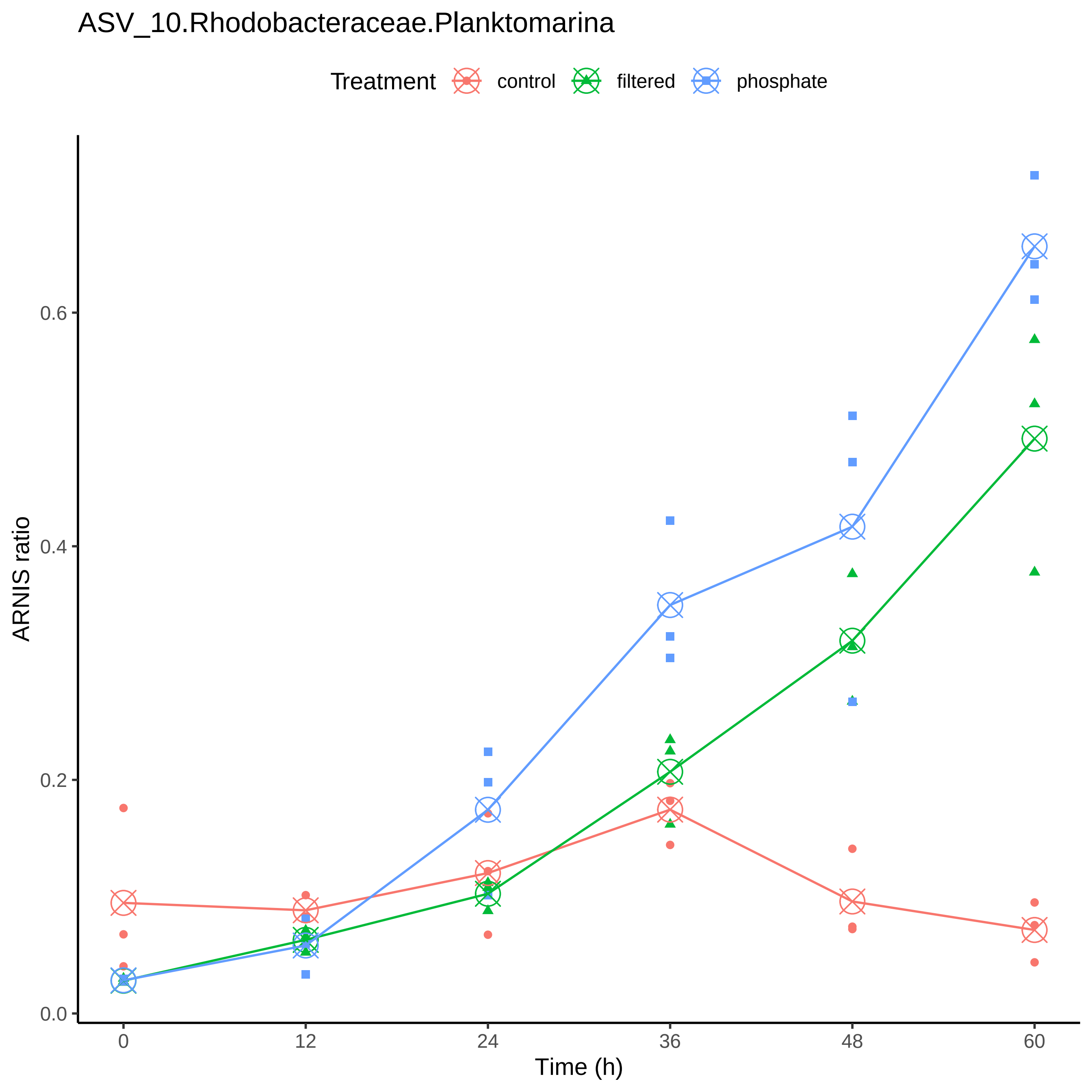

ASV_10.Rhodobacteraceae.Planktomarina
Treatment
control
filtered
phosphate
ARNIS ratio
0.0
0.2
0.4
0.6
Time (h)
0
12
24
36
48
60

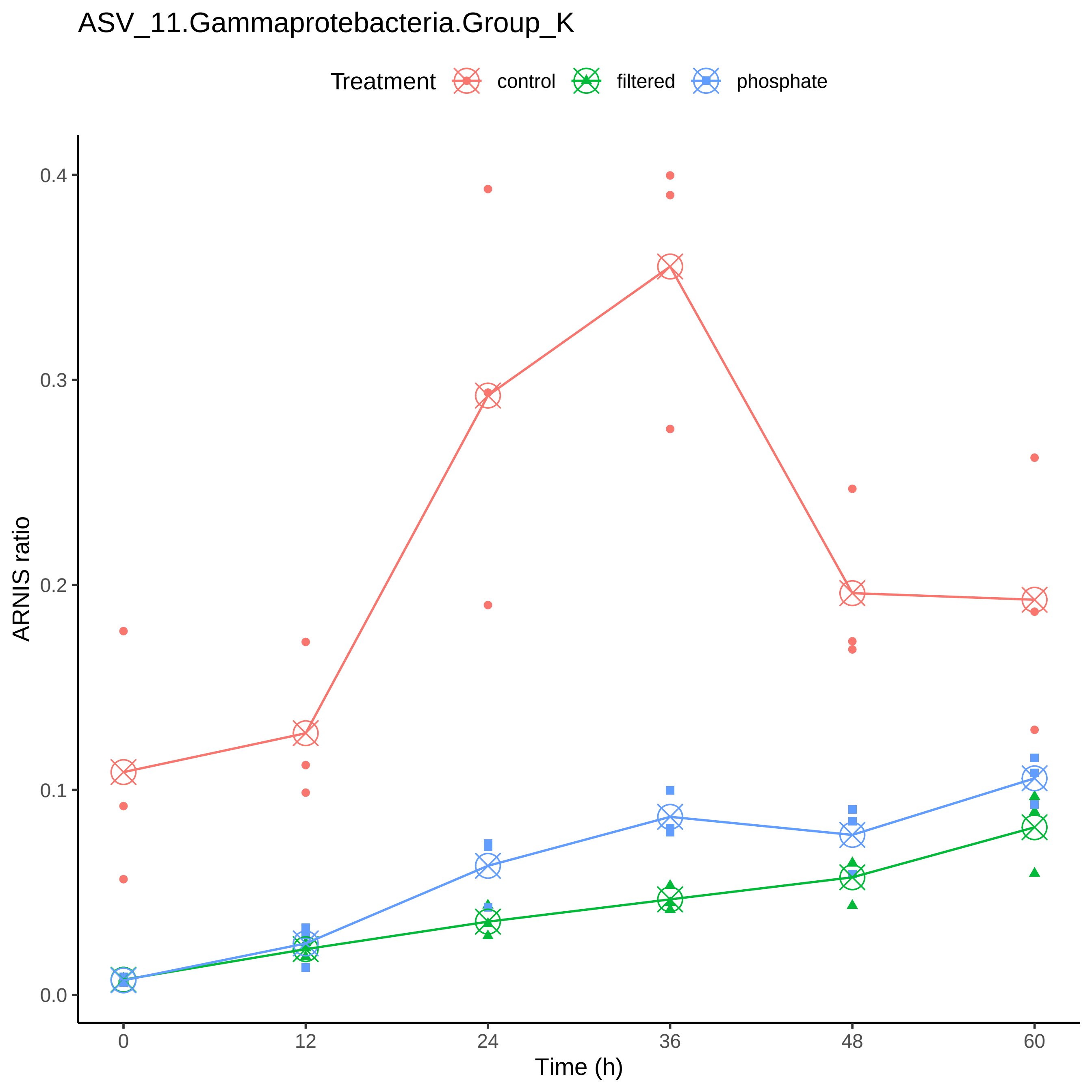

ASV_11.Gammaprotebacteria.Group_K
Treatment
control
filtered
phosphate
ARNIS ratio
0.0
0.1
0.2
0.3
0.4
Time (h)
0
12
24
36
48
60

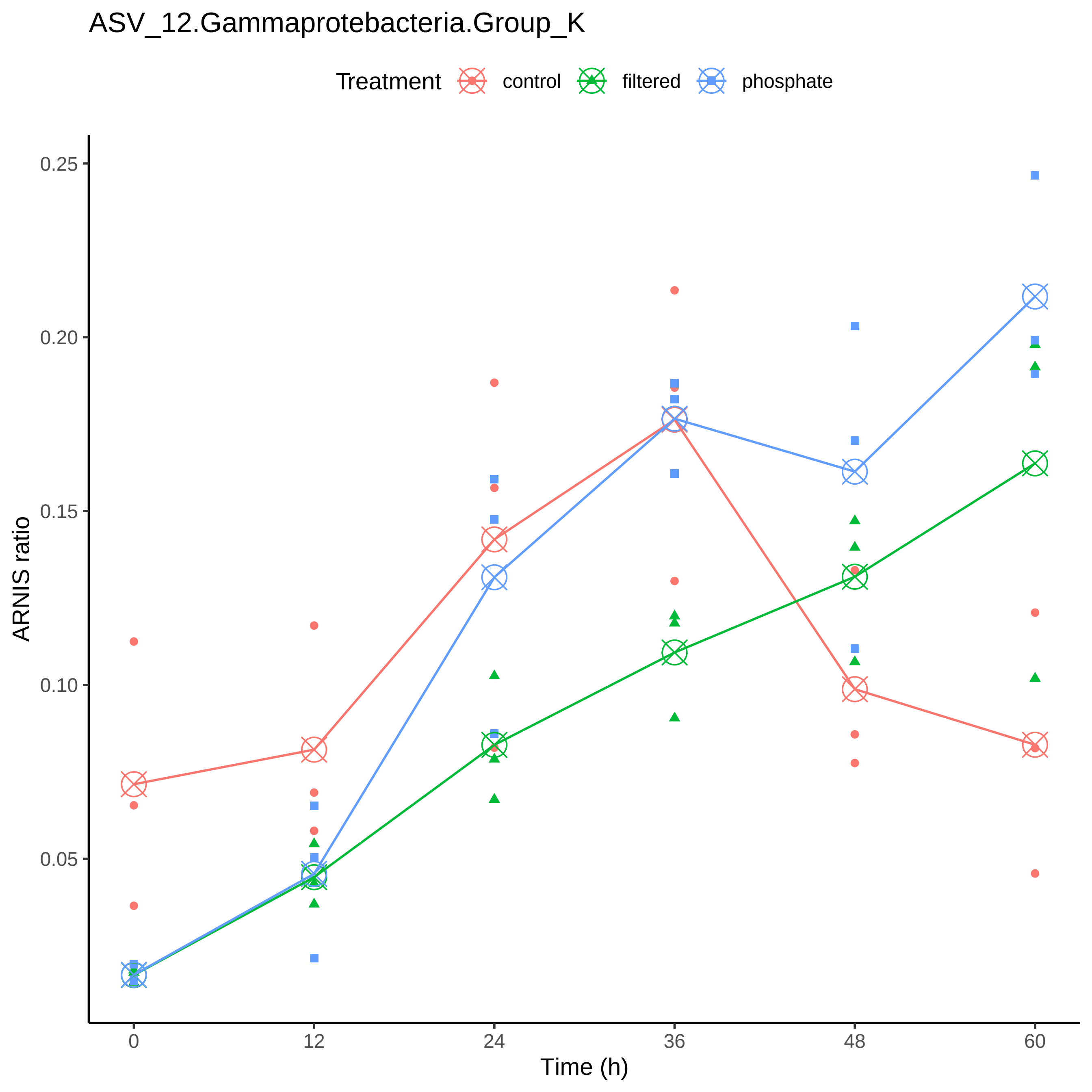
ASV_12.Gammaprotebacteria.Group_K
Treatment
control
filtered
phosphate
ARNIS ratio
0.25
0.20
0.15
0.10
0.05
0
12
24
36
48
60
Time (h)

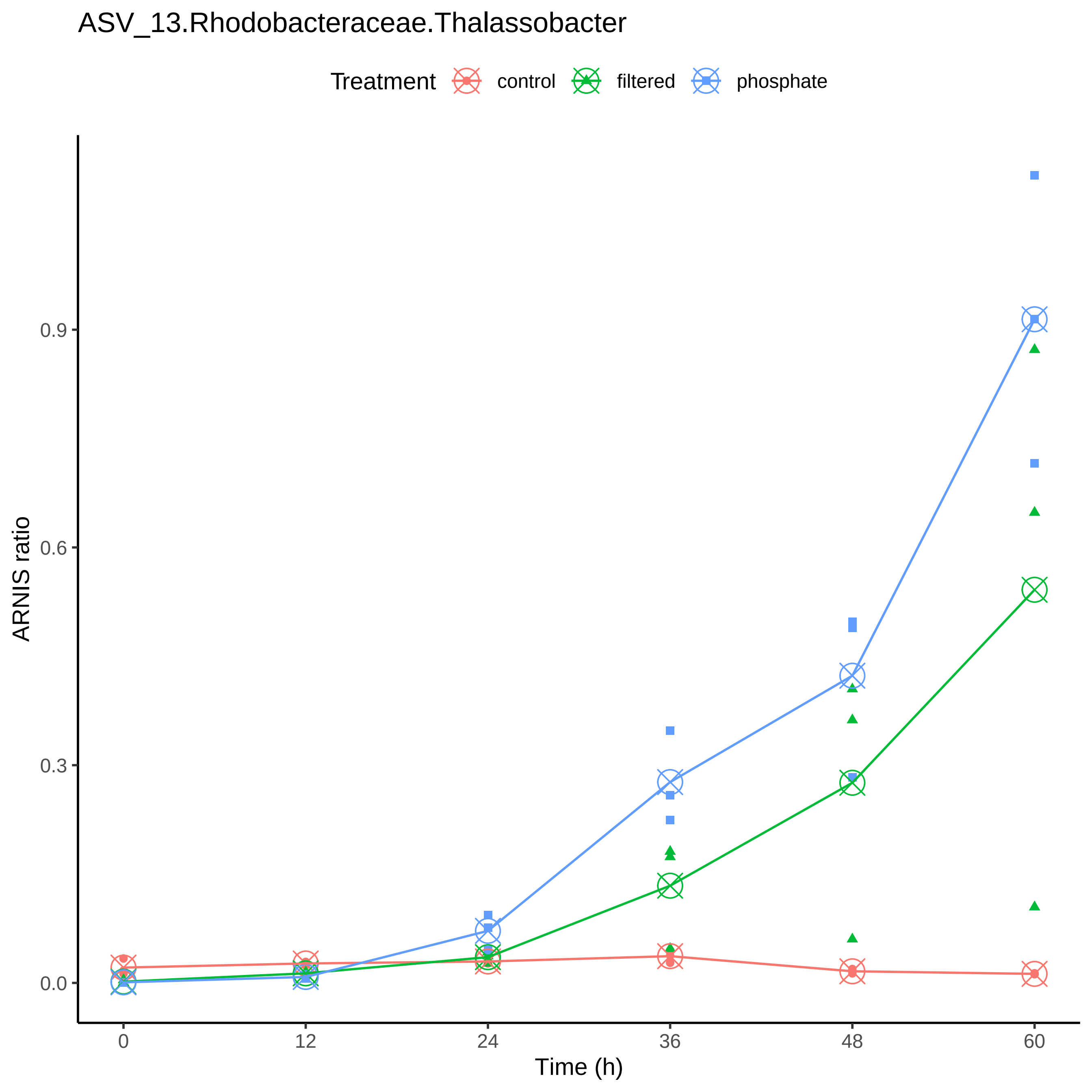

ASV_13.Rhodobacteraceae.Thalassobacter
Treatment
control
filtered
phosphate
ARNIS ratio
0.0
0.3
0.6
0.9
0
12
24
36
48
60
Time (h)

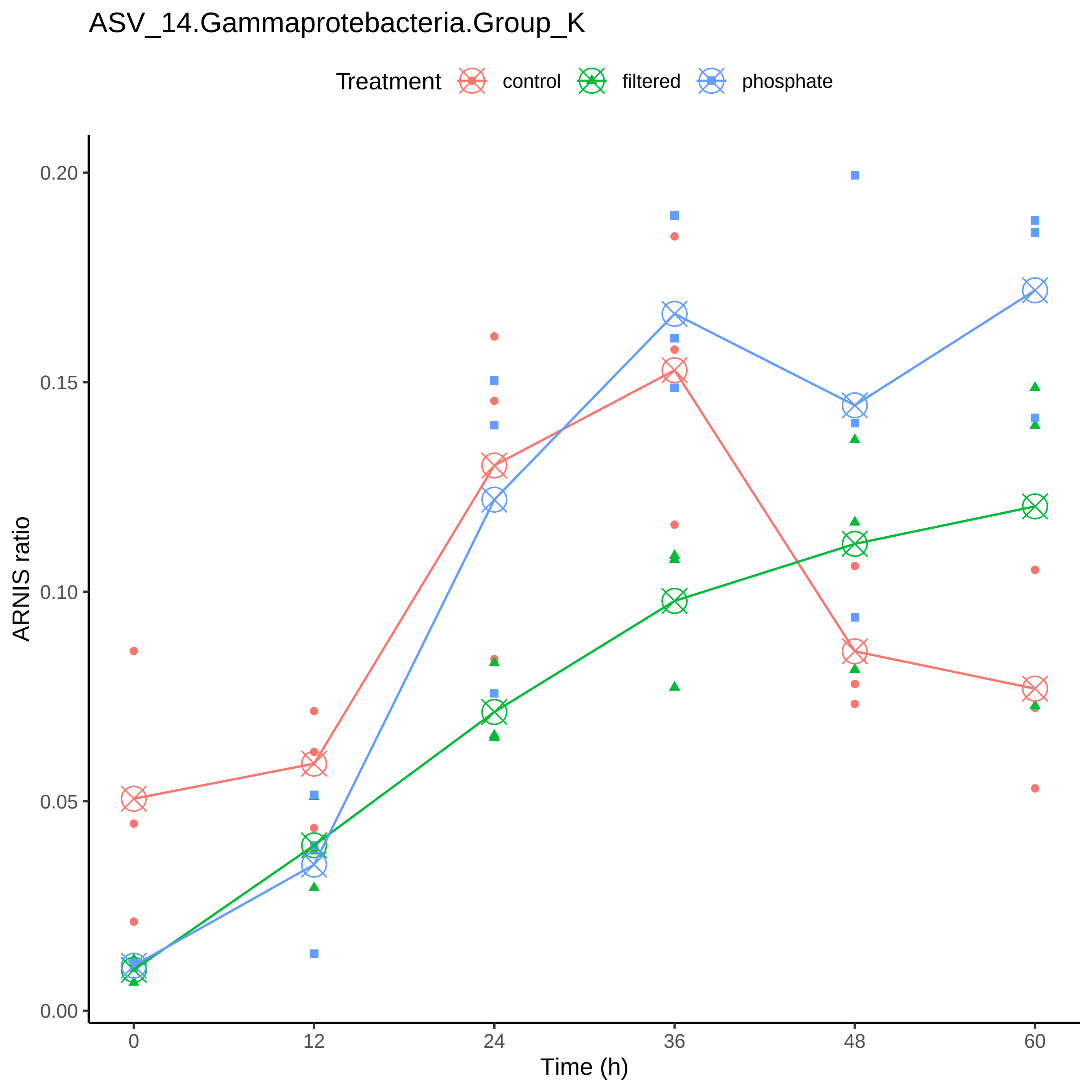

ASV_14.Gammaprotebacteria.Group_K
Treatment
control
filtered
phosphate
ARNIS ratio
0.00
0.05
0.10
0.15
0.20
Time (h)
0
12
24
36
48
60

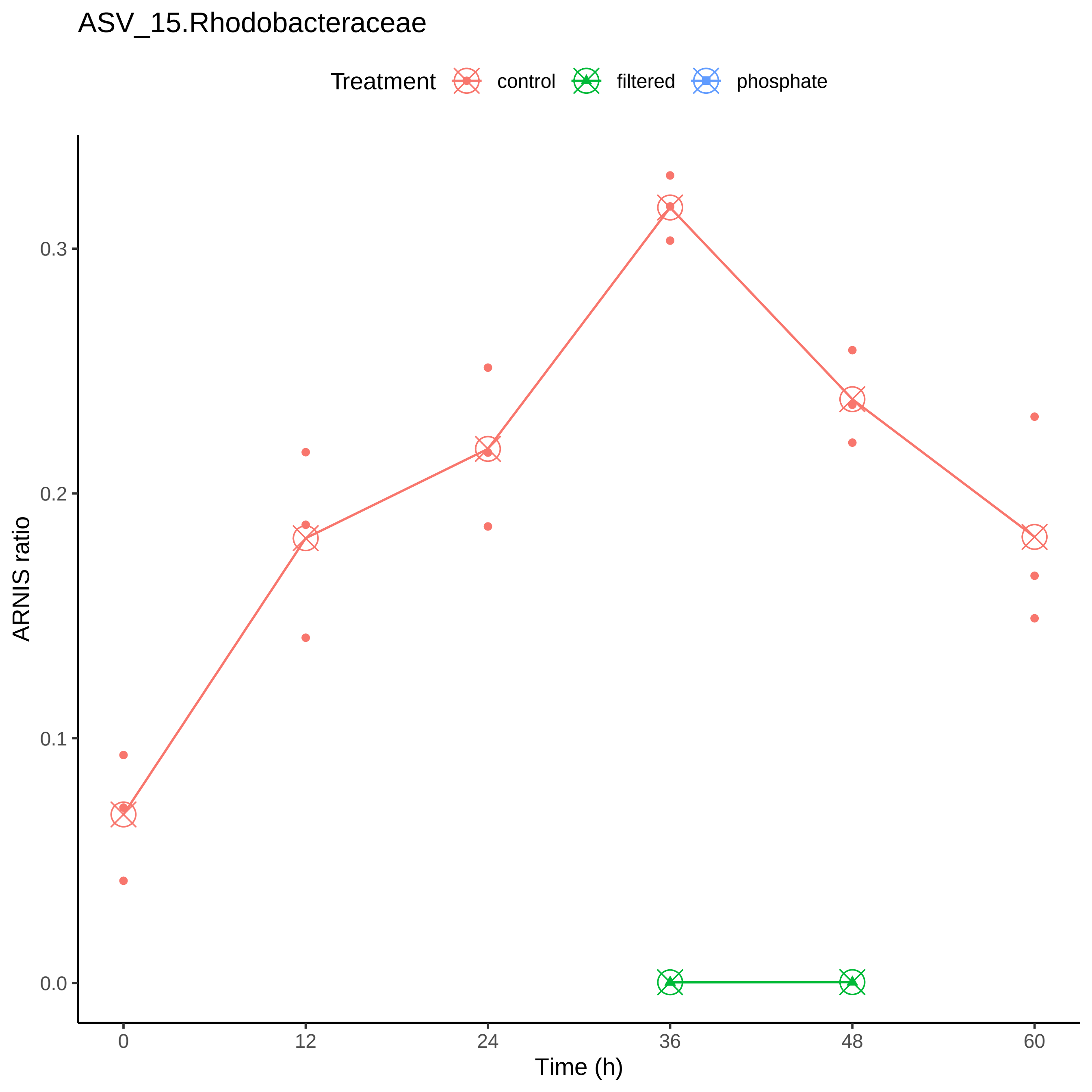
ASV_15.Rhodobacteraceae
Treatment
control
filtered
phosphate
0.3
0.2
0.1
0.0
ARNIS ratio
0
12
24
36
48
60
Time (h)

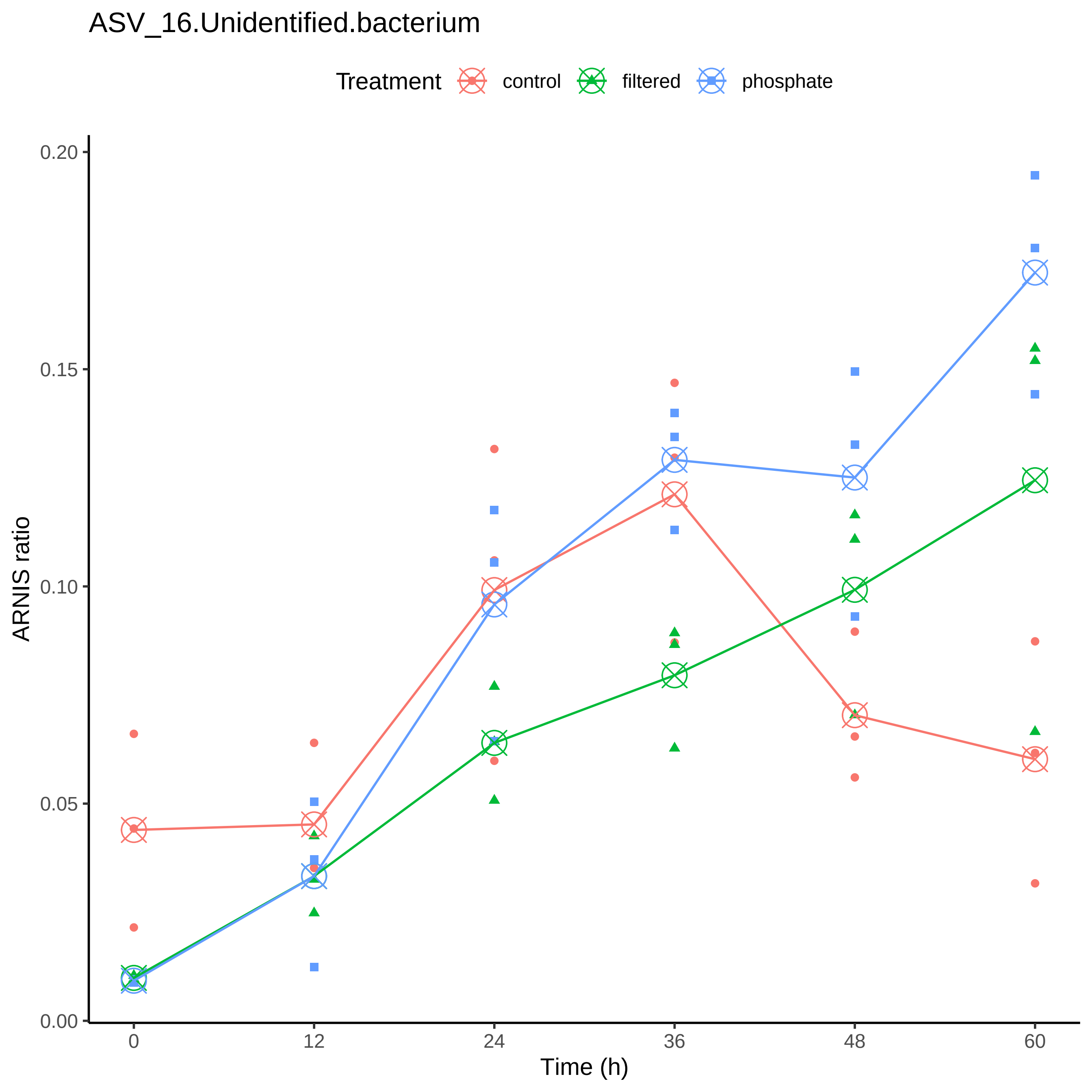

ASV_16.Unidentified.bacterium
Treatment
control
filtered
phosphate
ARNIS ratio
0.00
0.05
0.10
0.15
0.20
Time (h)
0
12
24
36
48
60

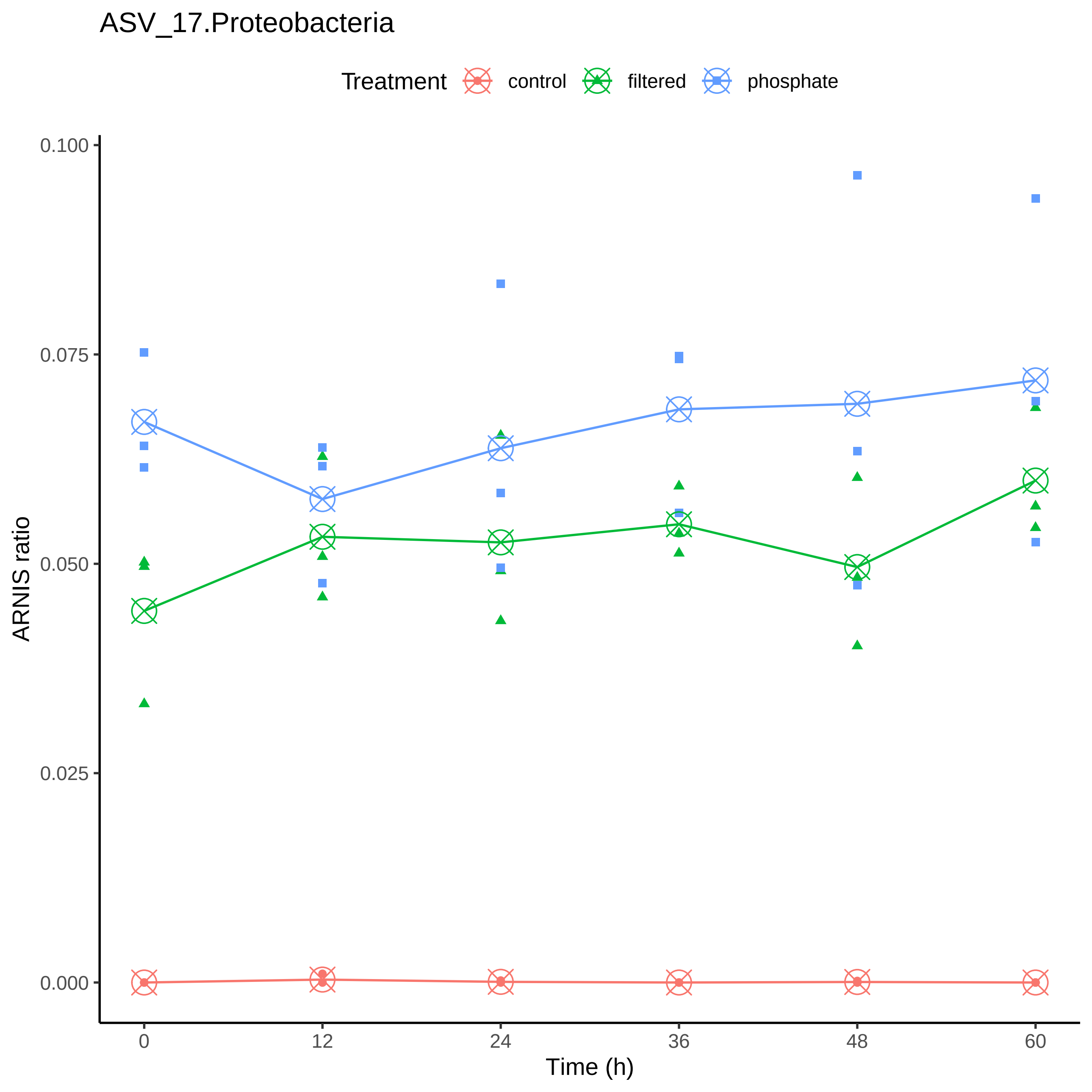

ASV_17.Proteobacteria
Treatment
control
filtered
phosphate
ARNIS ratio
0.100
0.075
0.050
0.025
0.000
Time (h)
0
12
24
36
48
60

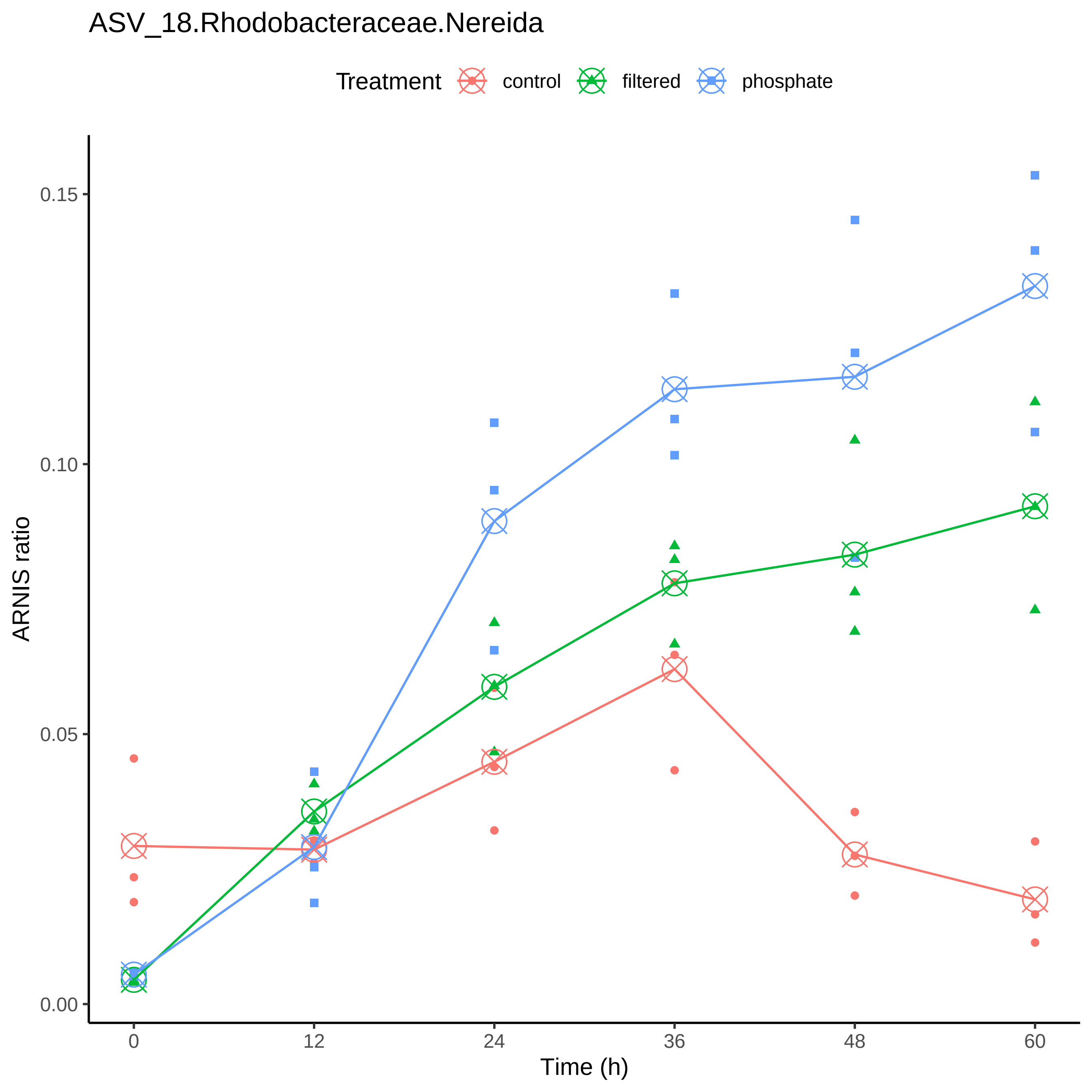

ASV_18.Rhodobacteraceae.Nereida
Treatment
control
filtered
phosphate
ARNIS ratio
0.15
0.10
0.05
0.00
0
12
24
36
48
60
Time (h)

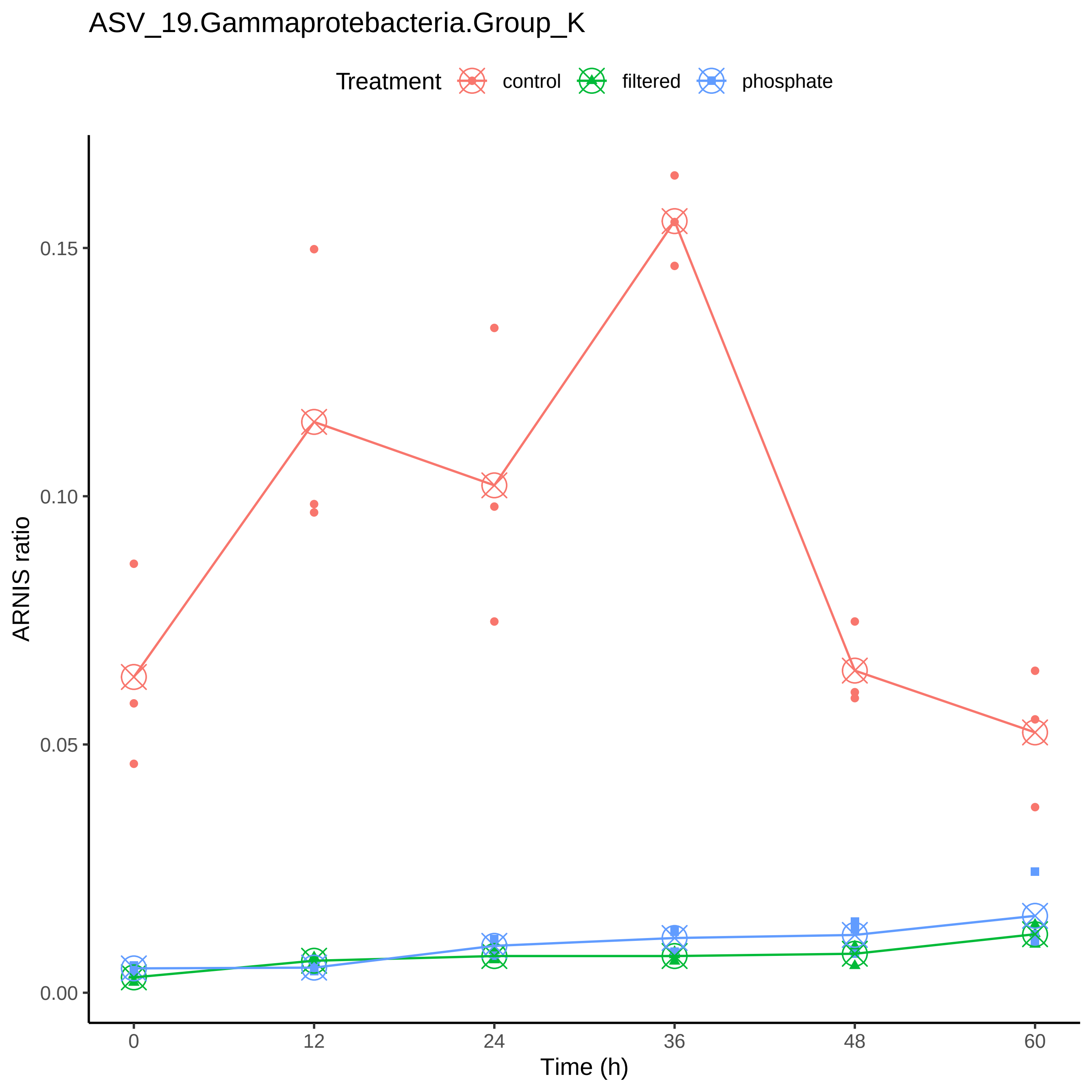

ASV_19.Gammaprotebacteria.Group_K
Treatment
control
filtered
phosphate
ARNIS ratio
0.15
0.10
0.05
0.00
0
12
24
36
48
60
Time (h)

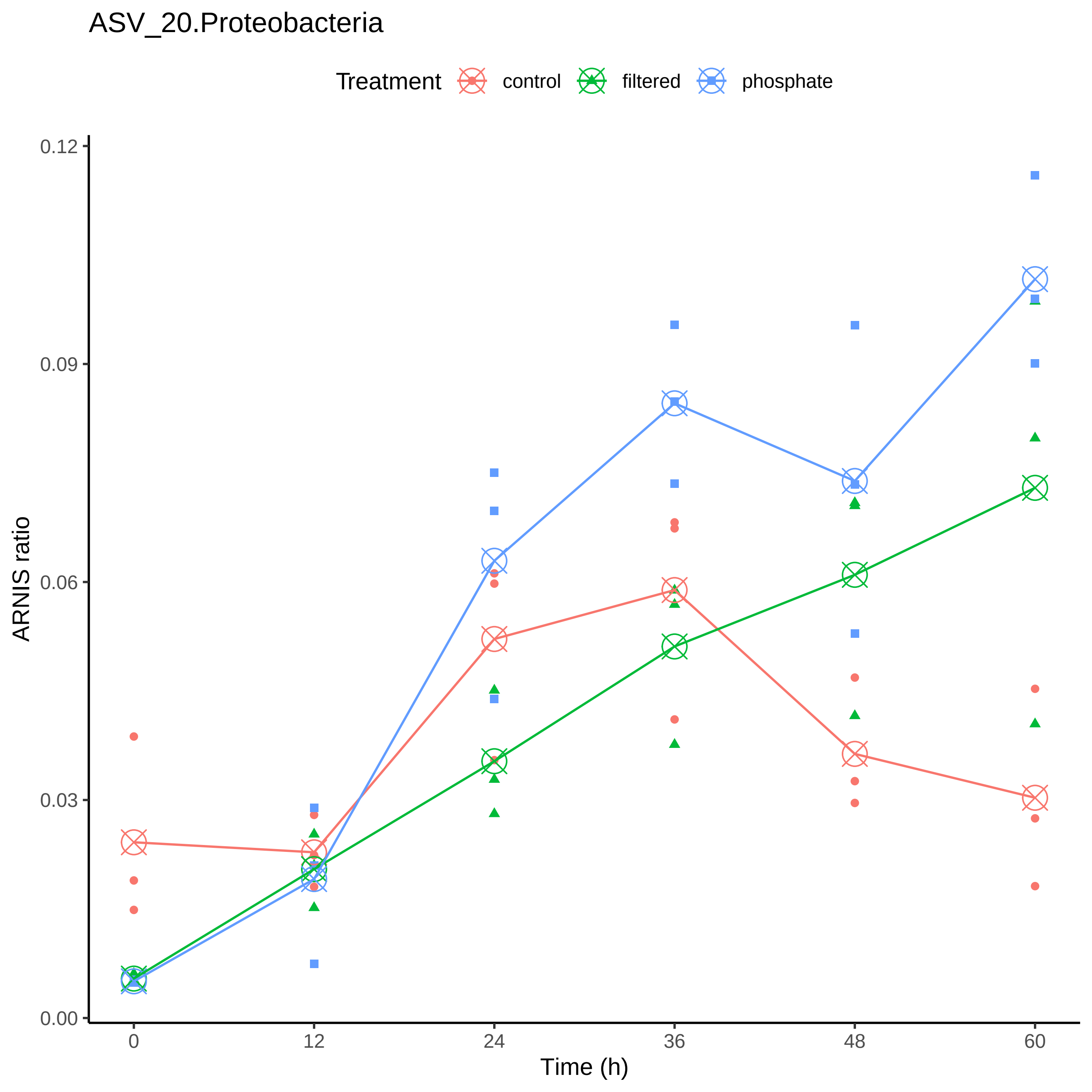

ASV_20.Proteobacteria
Treatment
control
filtered
phosphate
ARNIS ratio
0.00
0.03
0.06
0.09
0.12
0
12
24
36
48
60
Time (h)

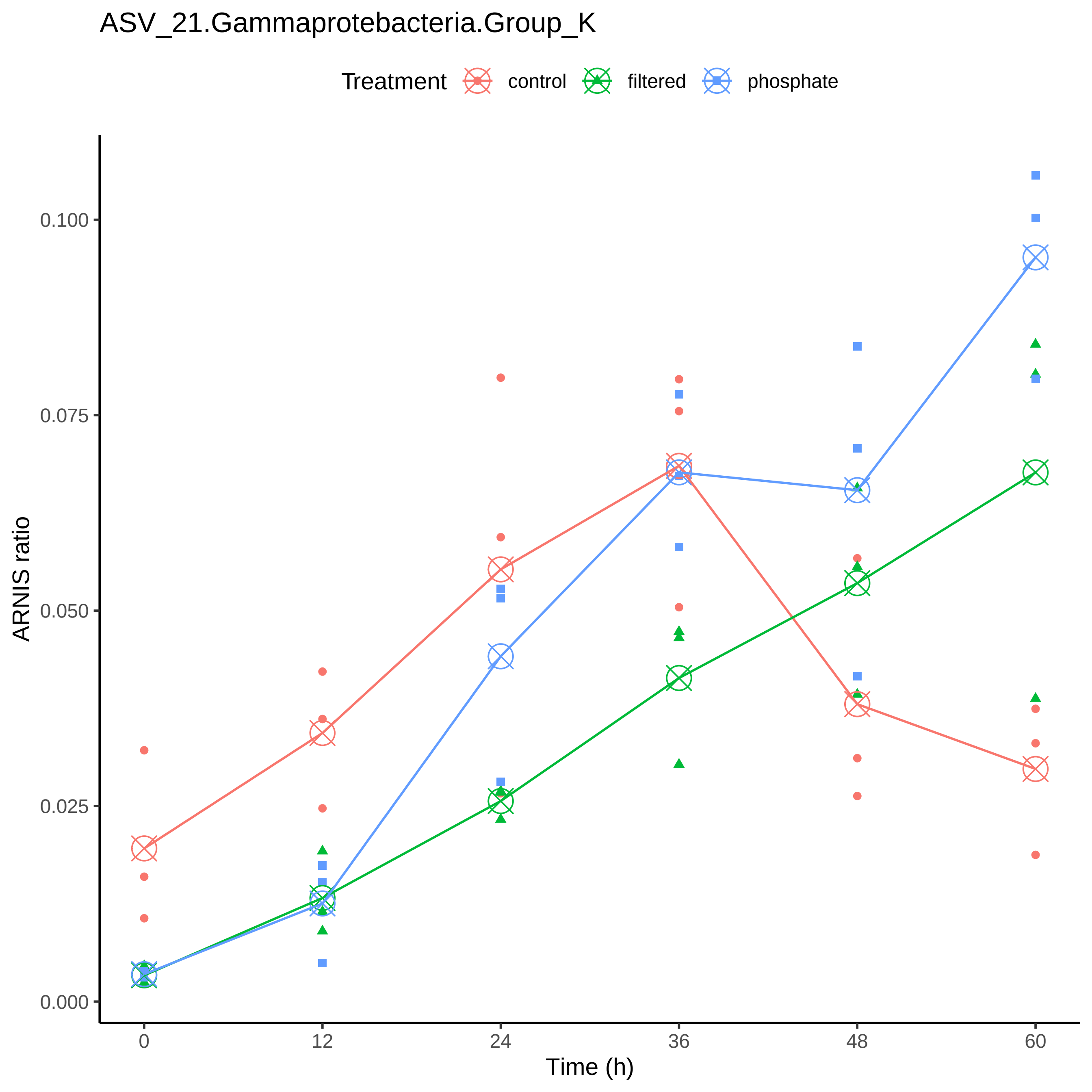

ASV_21.Gammaprotebacteria.Group_K
Treatment
control
filtered
phosphate
ARNIS ratio
0.100
0.075
0.050
0.025
0.000
0
12
24
36
48
60
Time (h)

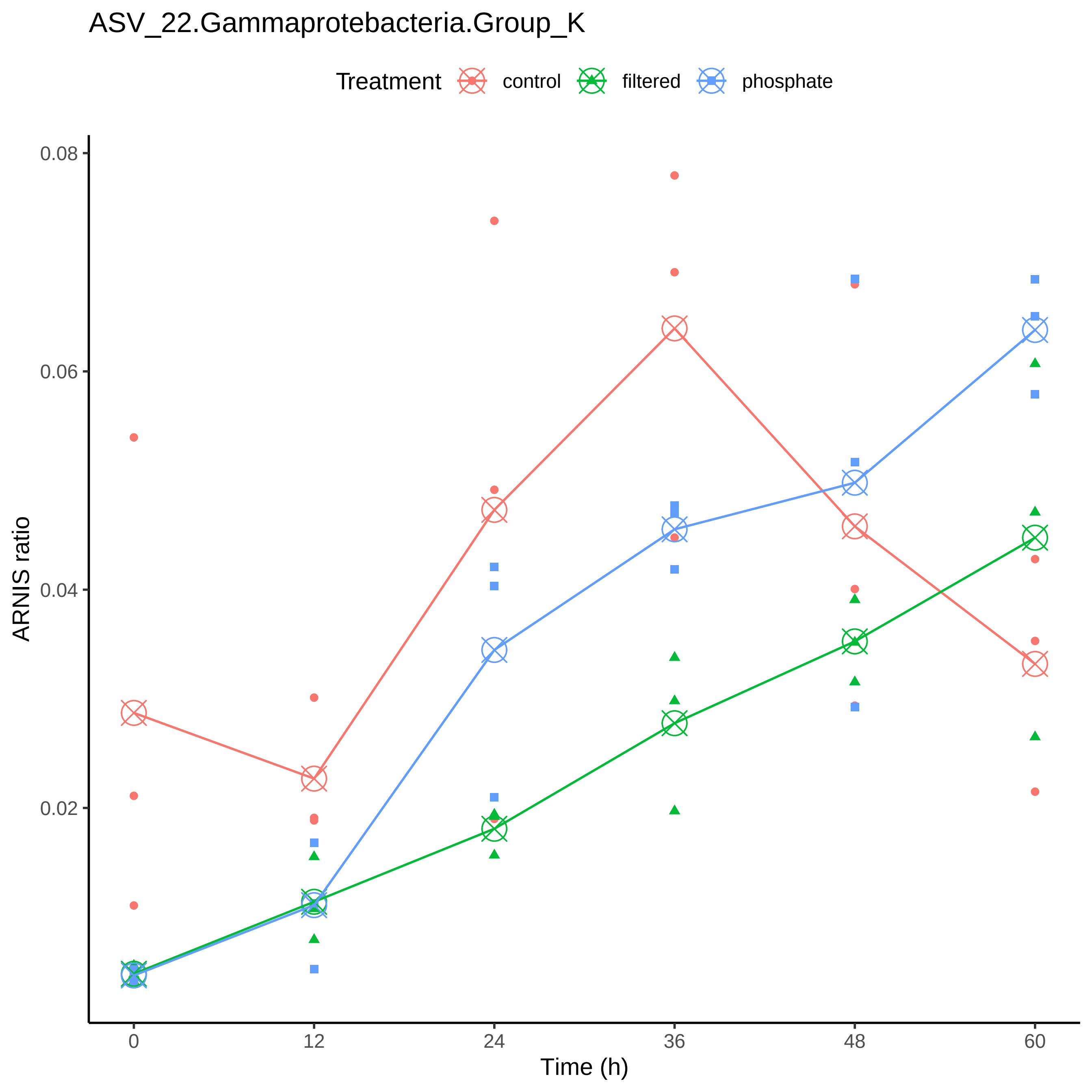

ASV_22.Gammaprotebacteria.Group_K
Treatment
control
filtered
phosphate
ARNIS ratio
0.08
0.06
0.04
0.02
0
12
24
36
48
60
Time (h)

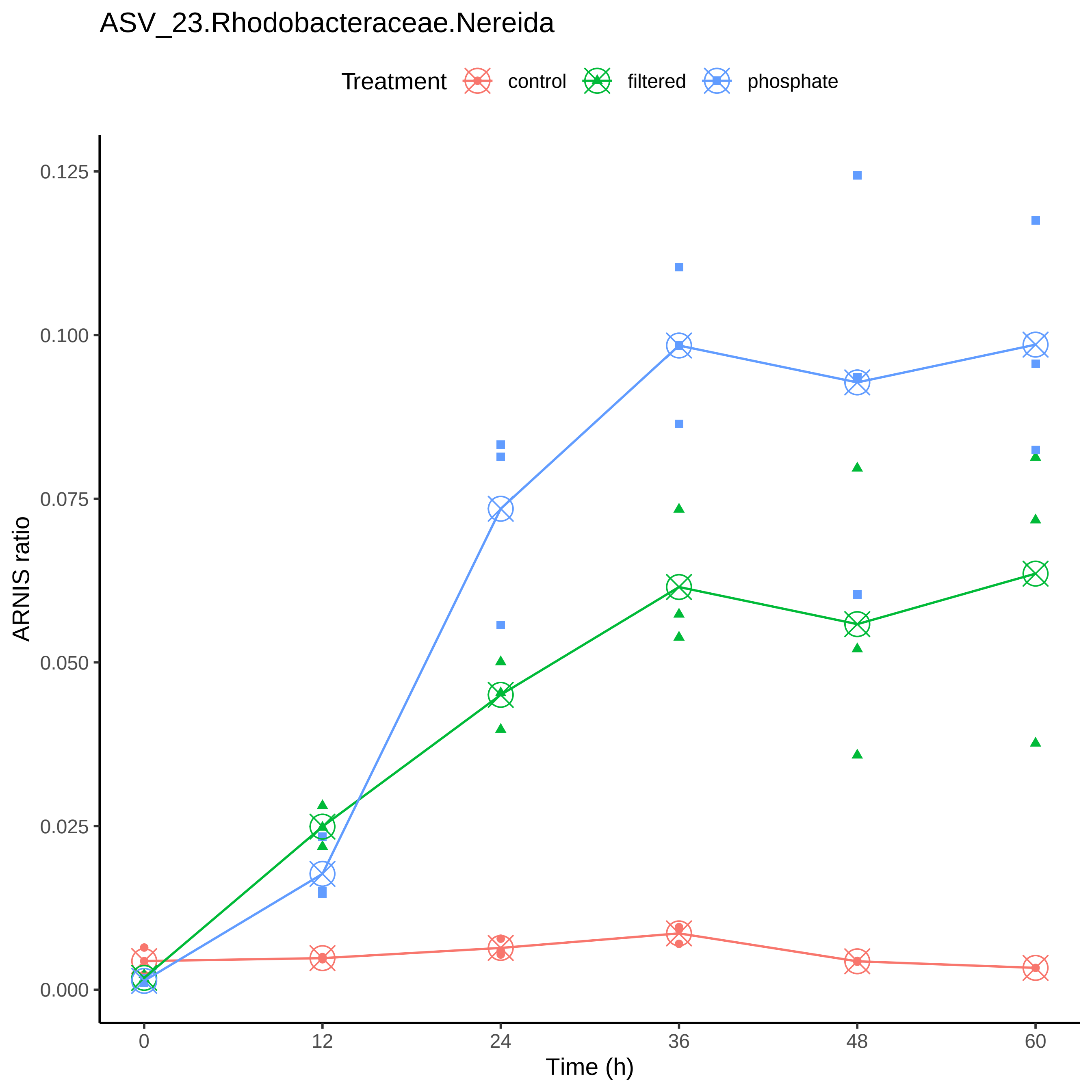
ASV_23.Rhodobacteraceae.Nereida
Treatment
control
filtered
phosphate
ARNIS ratio
0.125
0.100
0.075
0.050
0.025
0.000
0
12
24
36
48
60
Time (h)

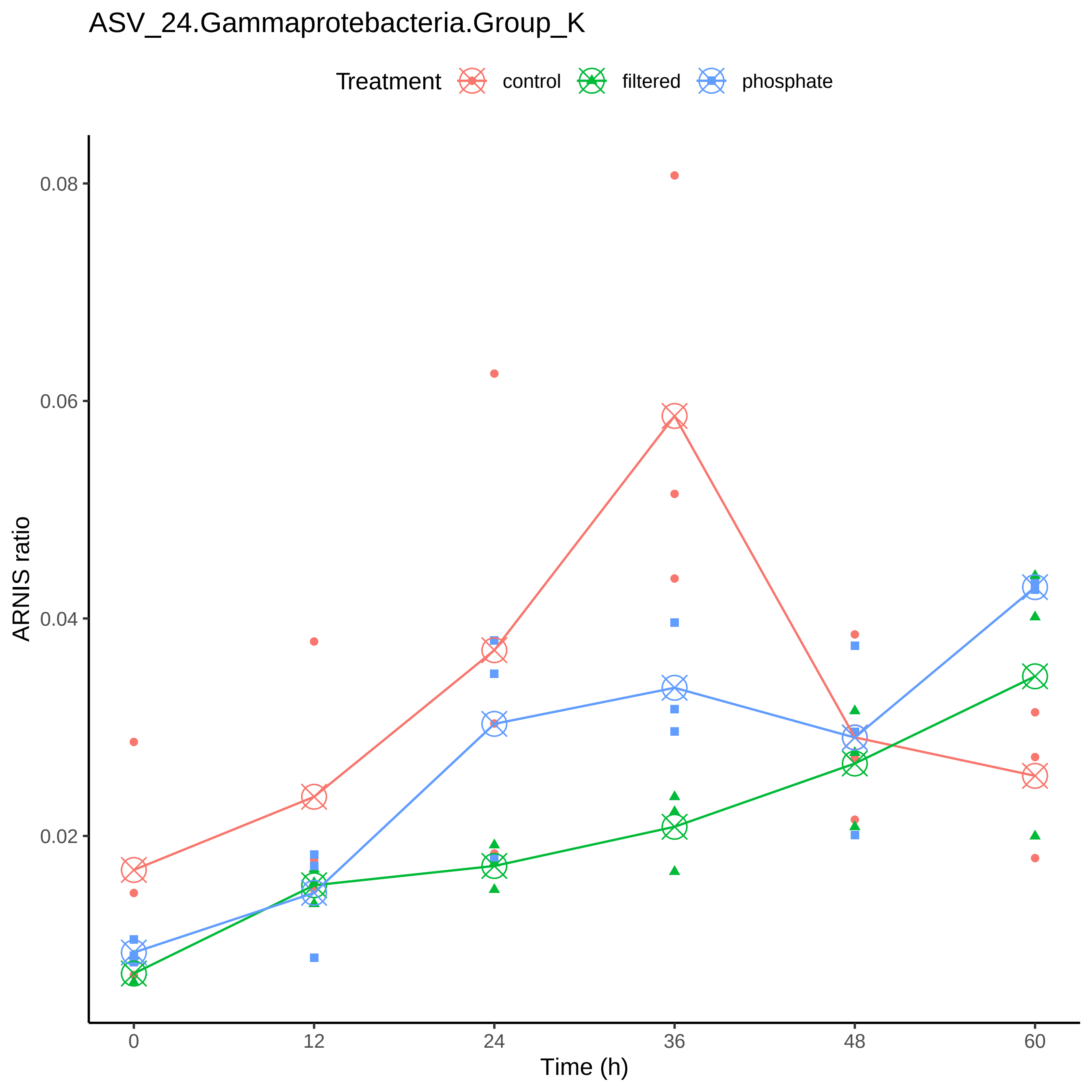
ASV_24.Gammaprotebacteria.Group_K
Treatment
control
filtered
phosphate
ARNIS ratio
0.08
0.06
0.04
0.02
0
12
24
36
48
60
Time (h)

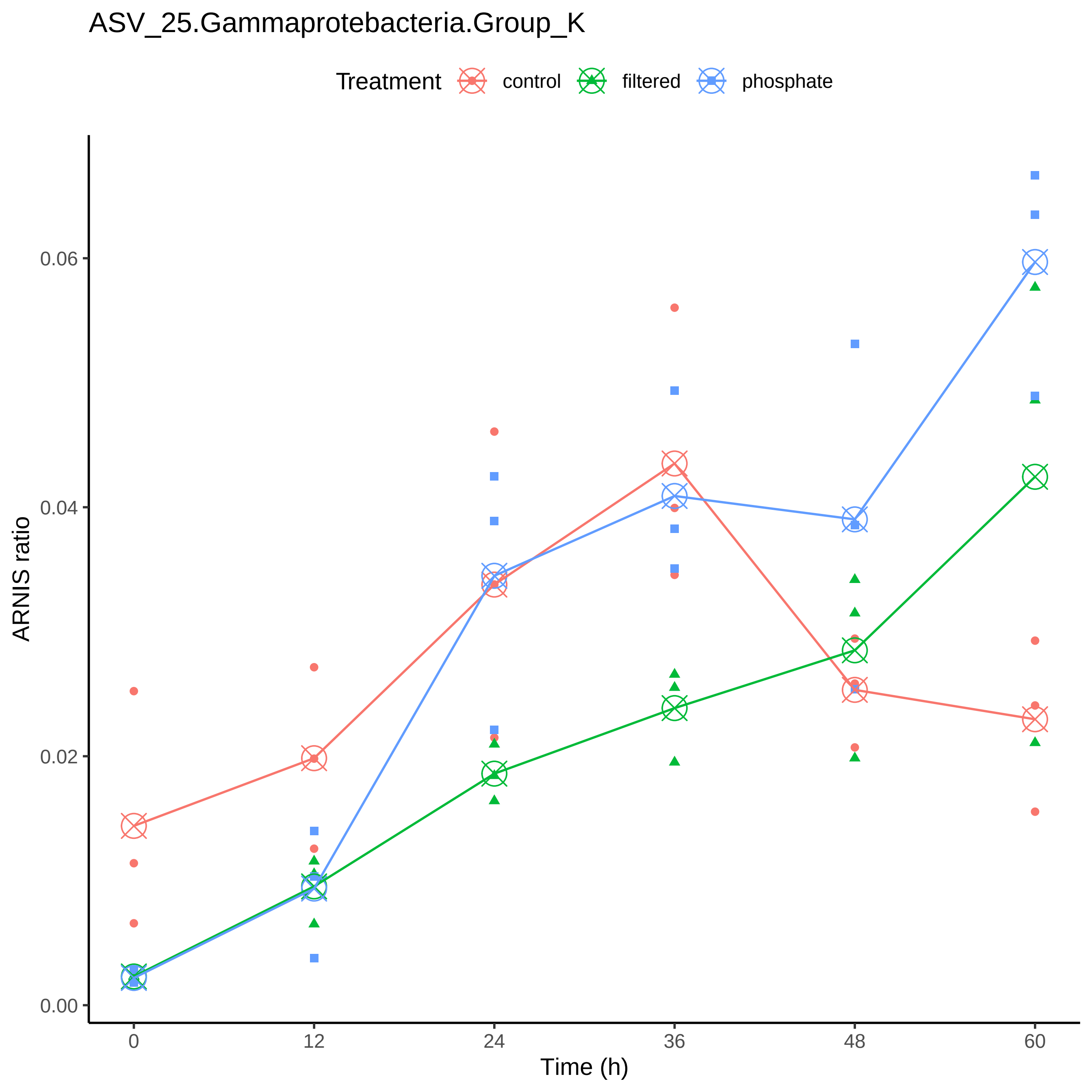
ASV_25.Gammaprotebacteria.Group_K
Treatment
control
filtered
phosphate
ARNIS ratio
0.00
0.02
0.04
0.06
Time (h)
0
12
24
36
48
60

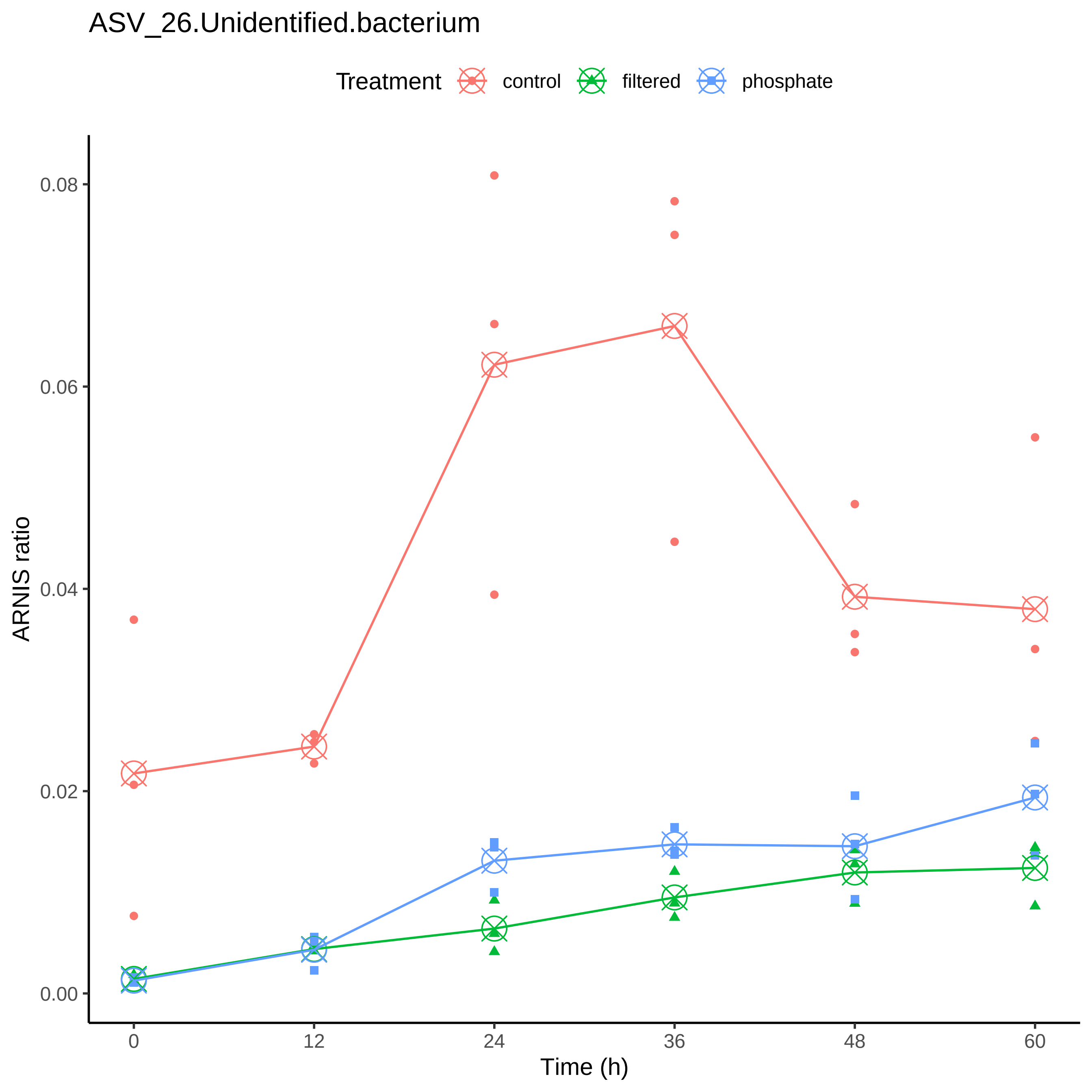

ASV_26.Unidentified.bacterium
Treatment
control
filtered
phosphate
ARNIS ratio
0.08
0.06
0.04
0.02
0.00
0
12
24
36
48
60
Time (h)

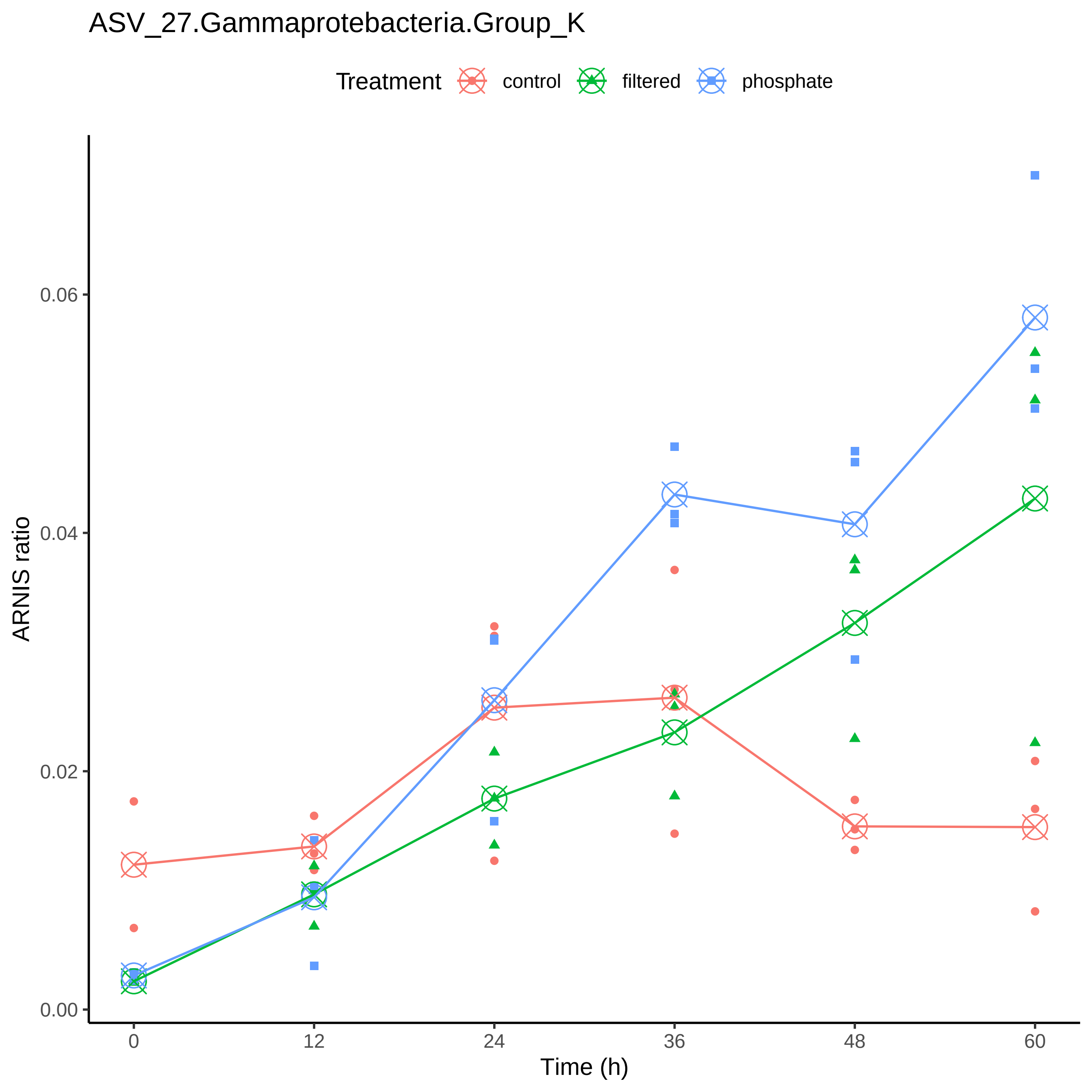
ASV_27.Gammaprotebacteria.Group_K
Treatment
control
filtered
phosphate
ARNIS ratio
0.06
0.04
0.02
0.00
0
12
24
36
48
60
Time (h)

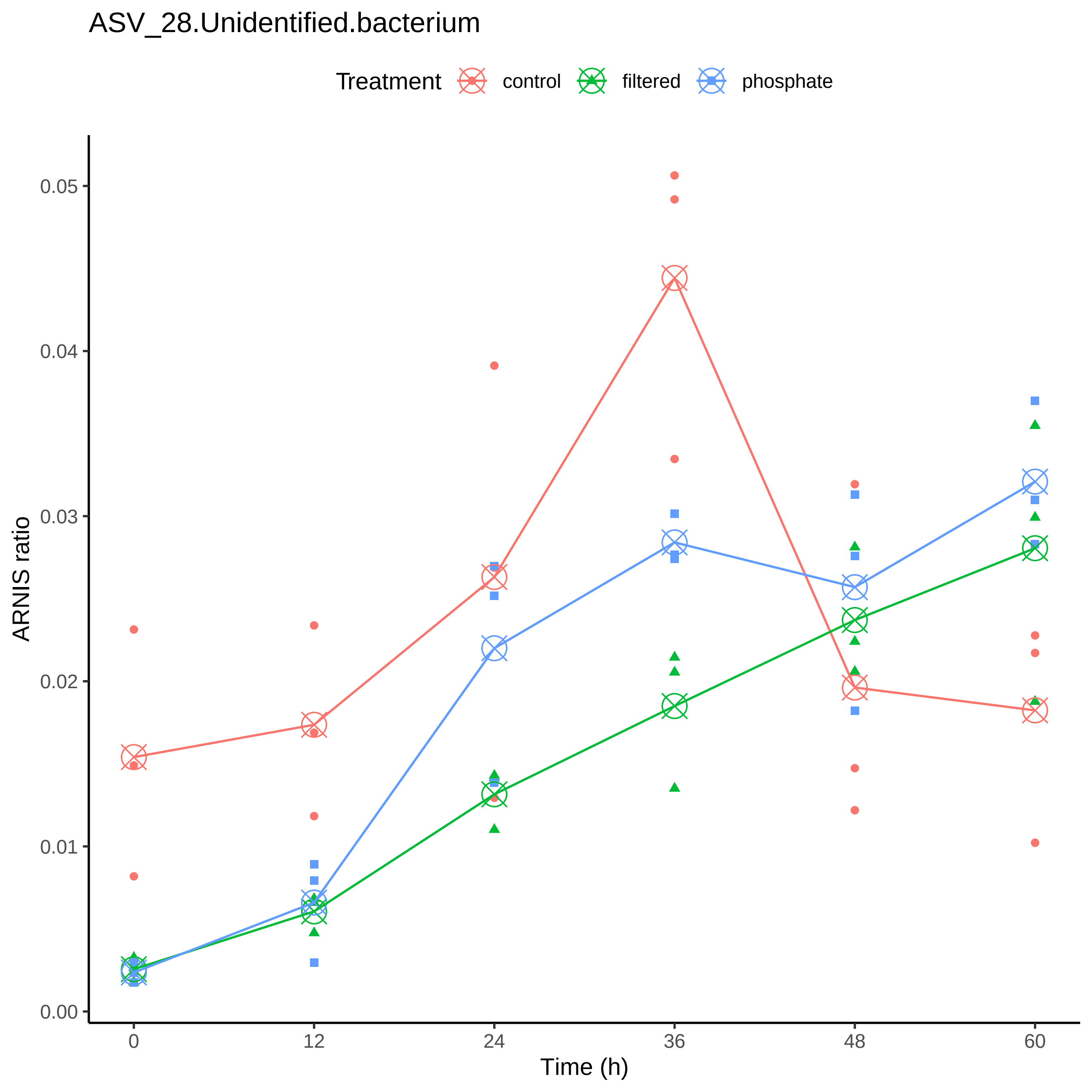

ASV_28.Unidentified.bacterium
Treatment
control
filtered
phosphate
ARNIS ratio
0.05
0.04
0.03
0.02
0.01
0.00
0
12
24
36
48
60
Time (h)

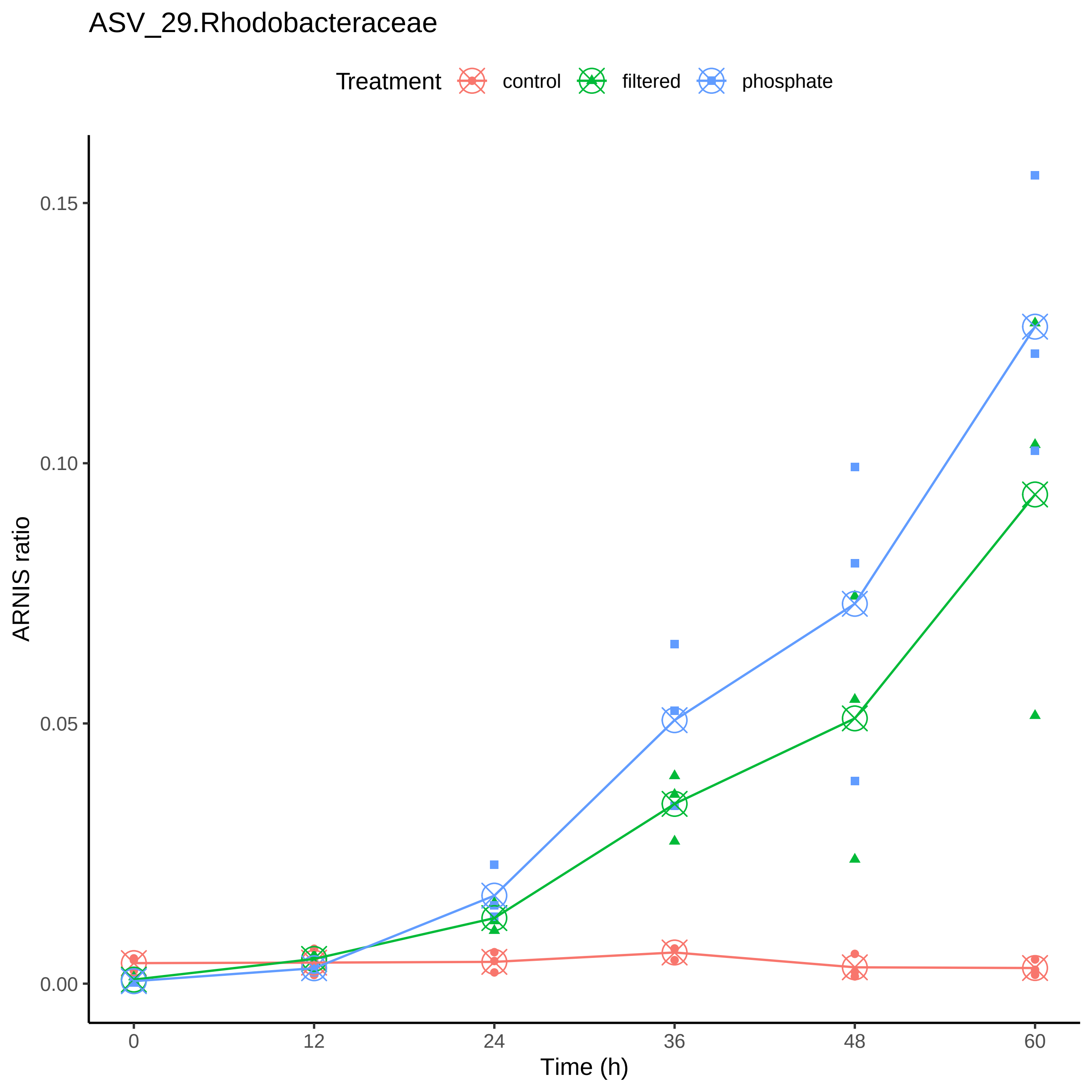
ASV_29.Rhodobacteraceae
Treatment
control
filtered
phosphate
ARNIS ratio
0.15
0.10
0.05
0.00
0
12
24
36
48
60
Time (h)

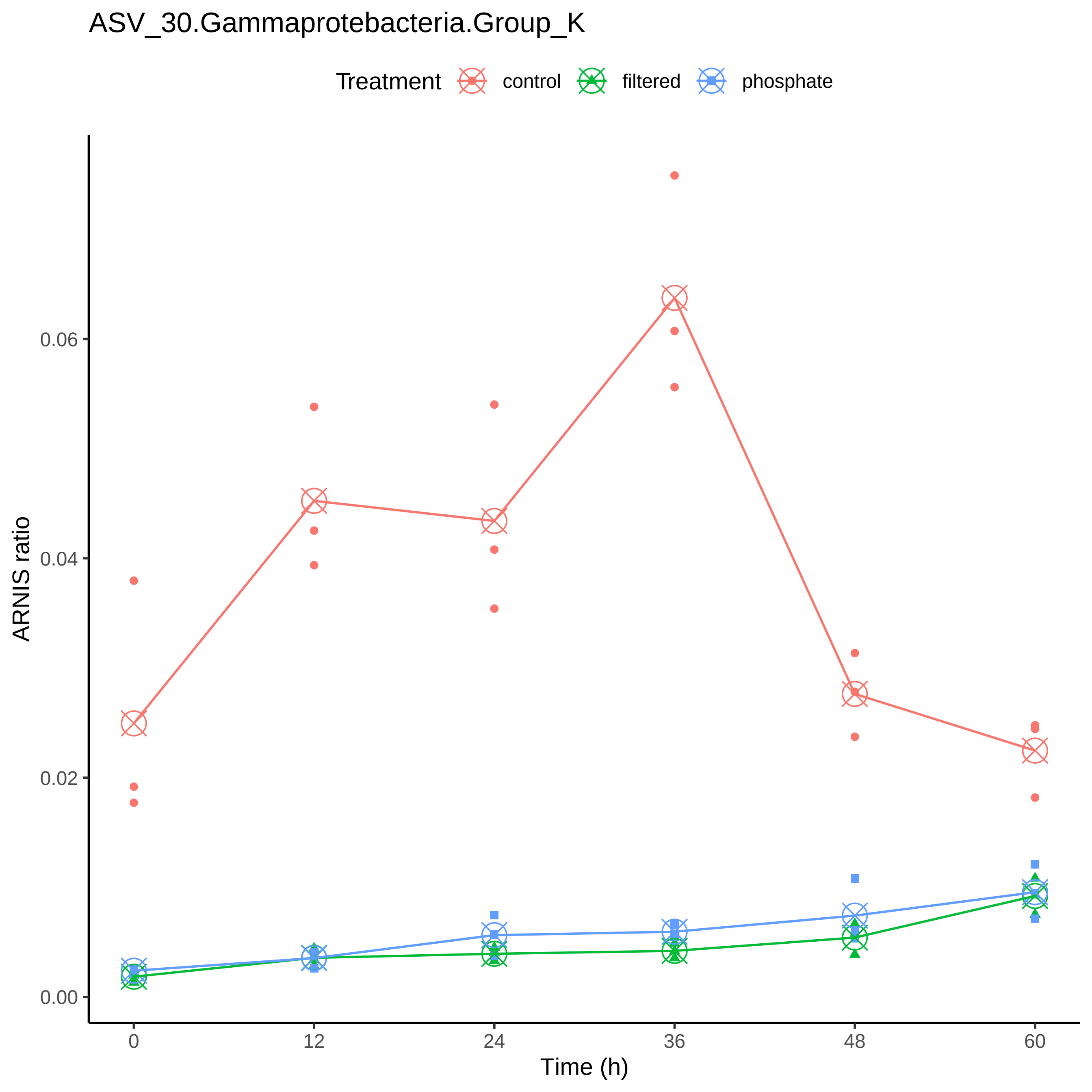

ASV_30.Gammaprotebacteria.Group_K
Treatment
control
filtered
phosphate
ARNIS ratio
0.00
0.02
0.04
0.06
Time (h)
0
12
24
36
48
60

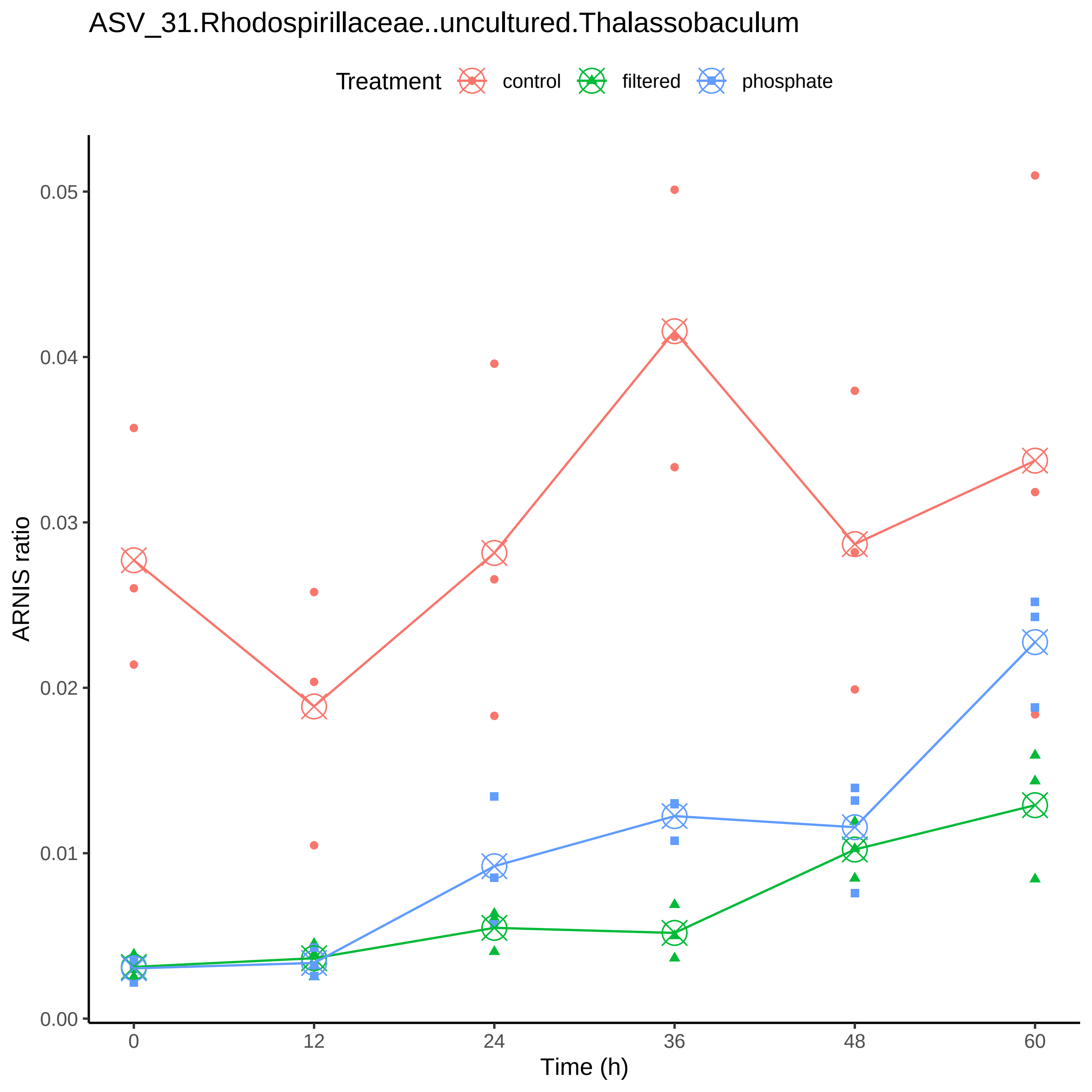

ASV_31.Rhodospirillaceae..uncultured.Thalassobaculum
Treatment
control
filtered
phosphate
ARNIS ratio
0.00
0.01
0.02
0.03
0.04
0.05
0
12
24
36
48
60
Time (h)

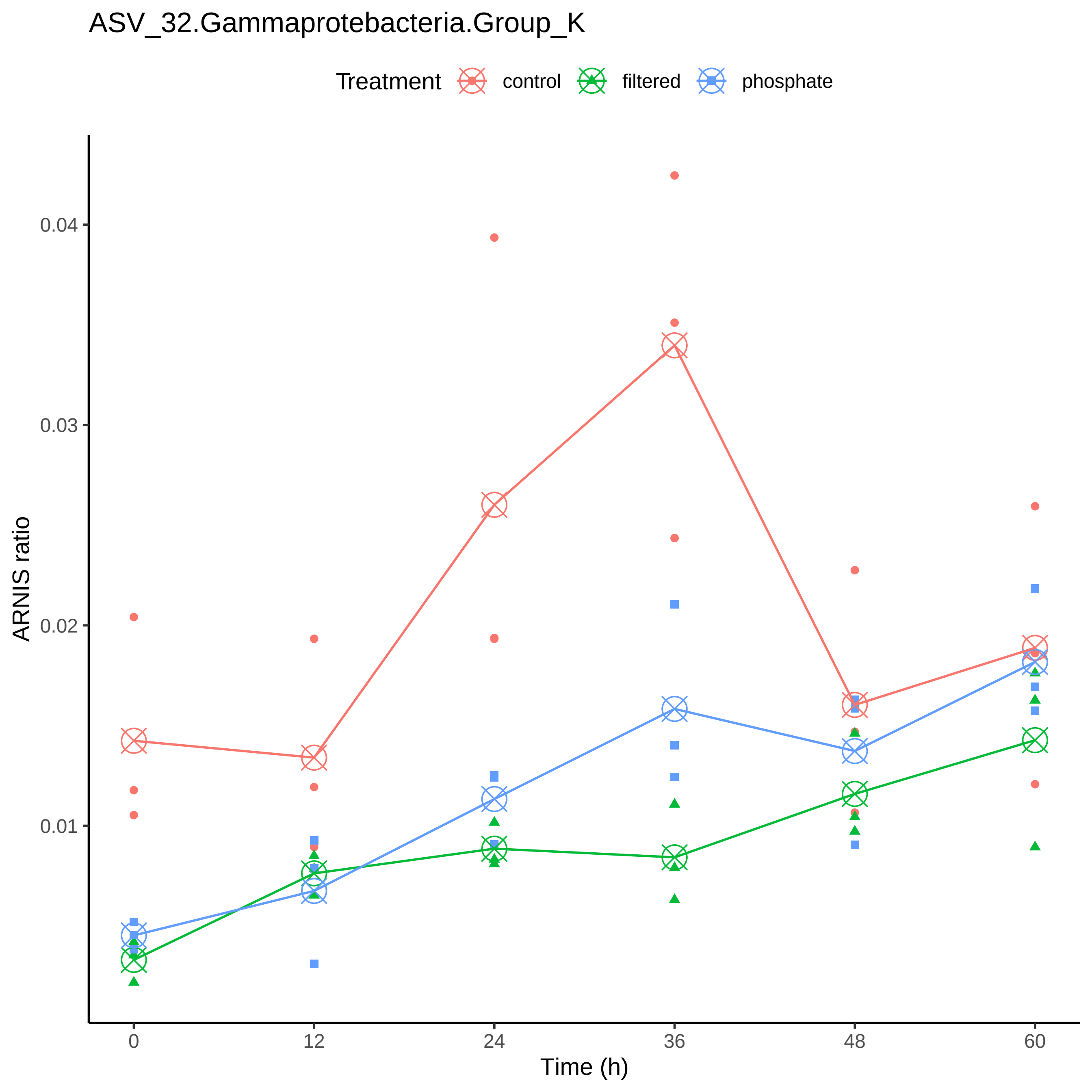

ASV_32.Gammaprotebacteria.Group_K
Treatment
control
filtered
phosphate
ARNIS ratio
0.04
0.03
0.02
0.01
0
12
24
36
48
60
Time (h)

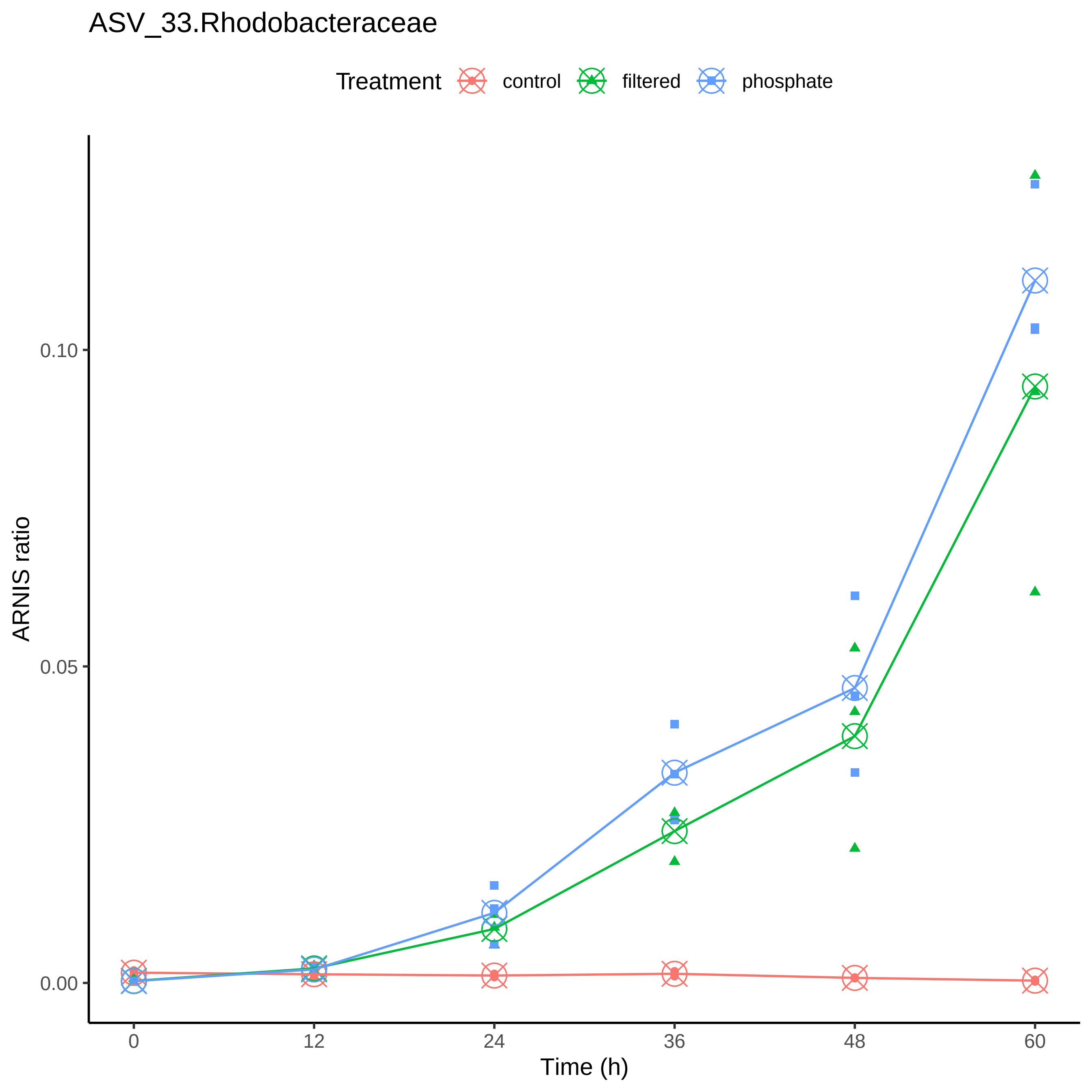
ASV_33.Rhodobacteraceae
Treatment
control
filtered
phosphate
ARNIS ratio
0.10
0.05
0.00
0
12
24
36
48
60
Time (h)

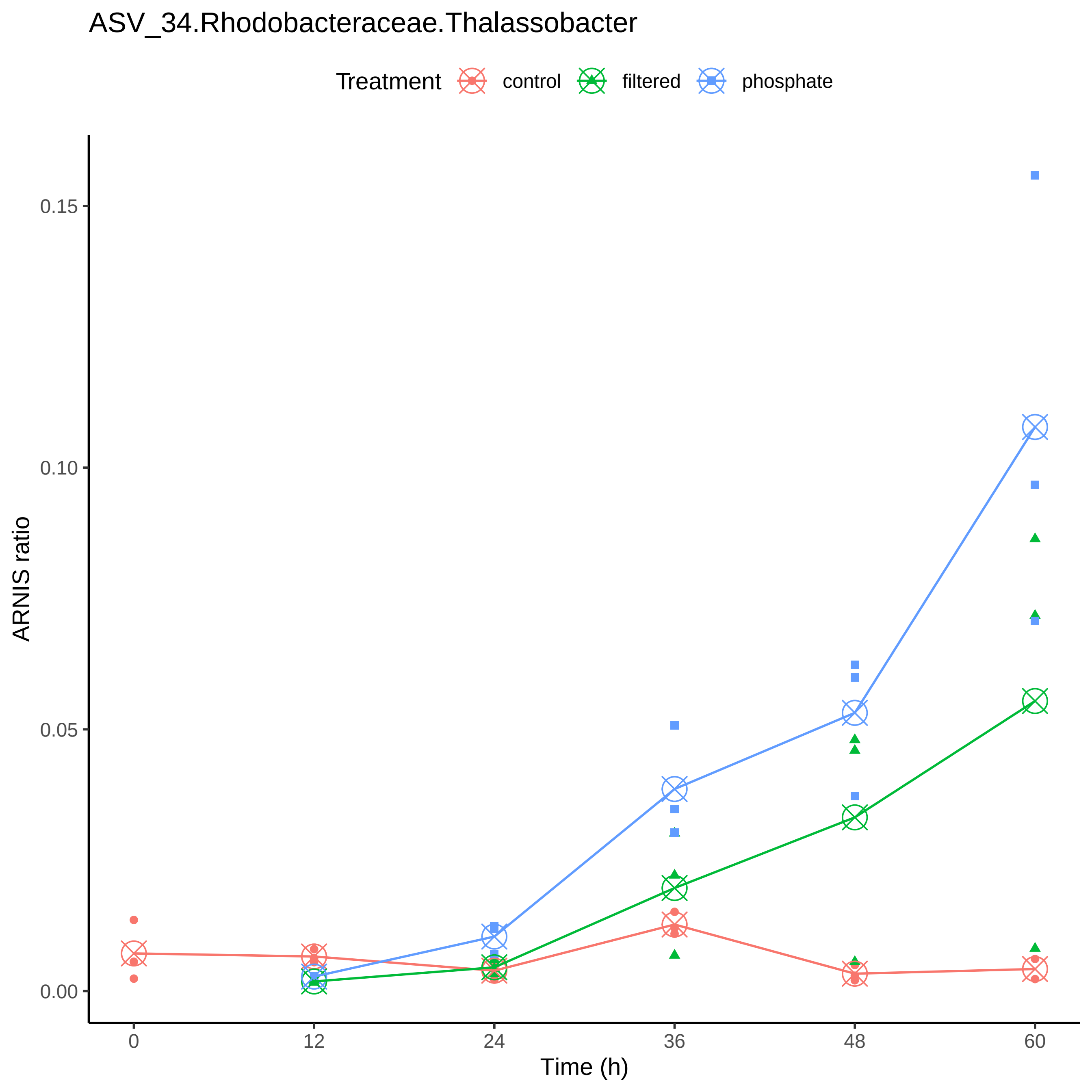
ASV_34.Rhodobacteraceae.Thalassobacter
Treatment
control
filtered
phosphate
ARNIS ratio
0.00
0.05
0.10
0.15
0
12
24
36
48
60
Time (h)

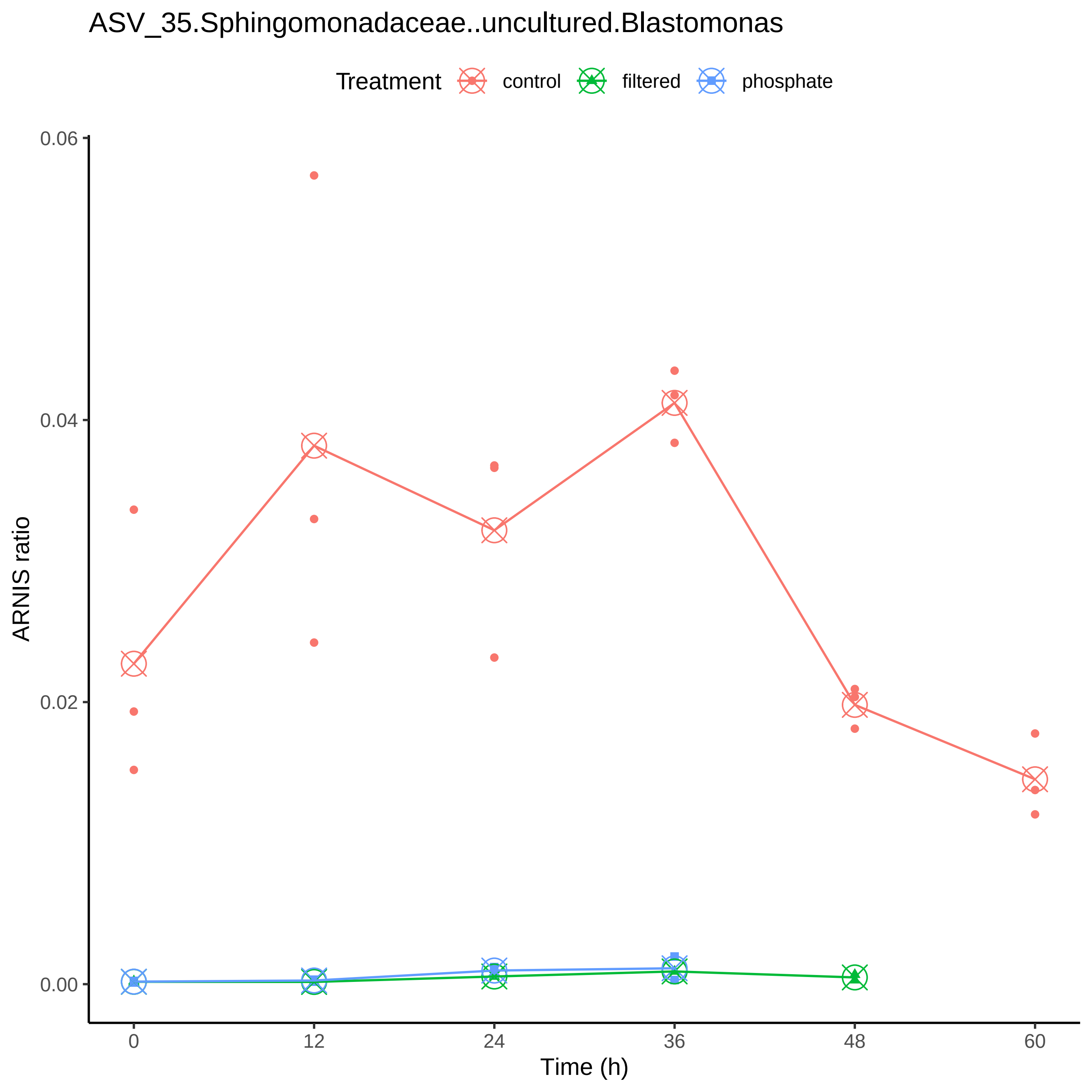

ASV_35.Sphingomonadaceae..uncultured.Blastomonas
Treatment
control
filtered
phosphate
0.06
0.04
0.02
0.00
ARNIS ratio
0
12
24
36
48
60
Time (h)

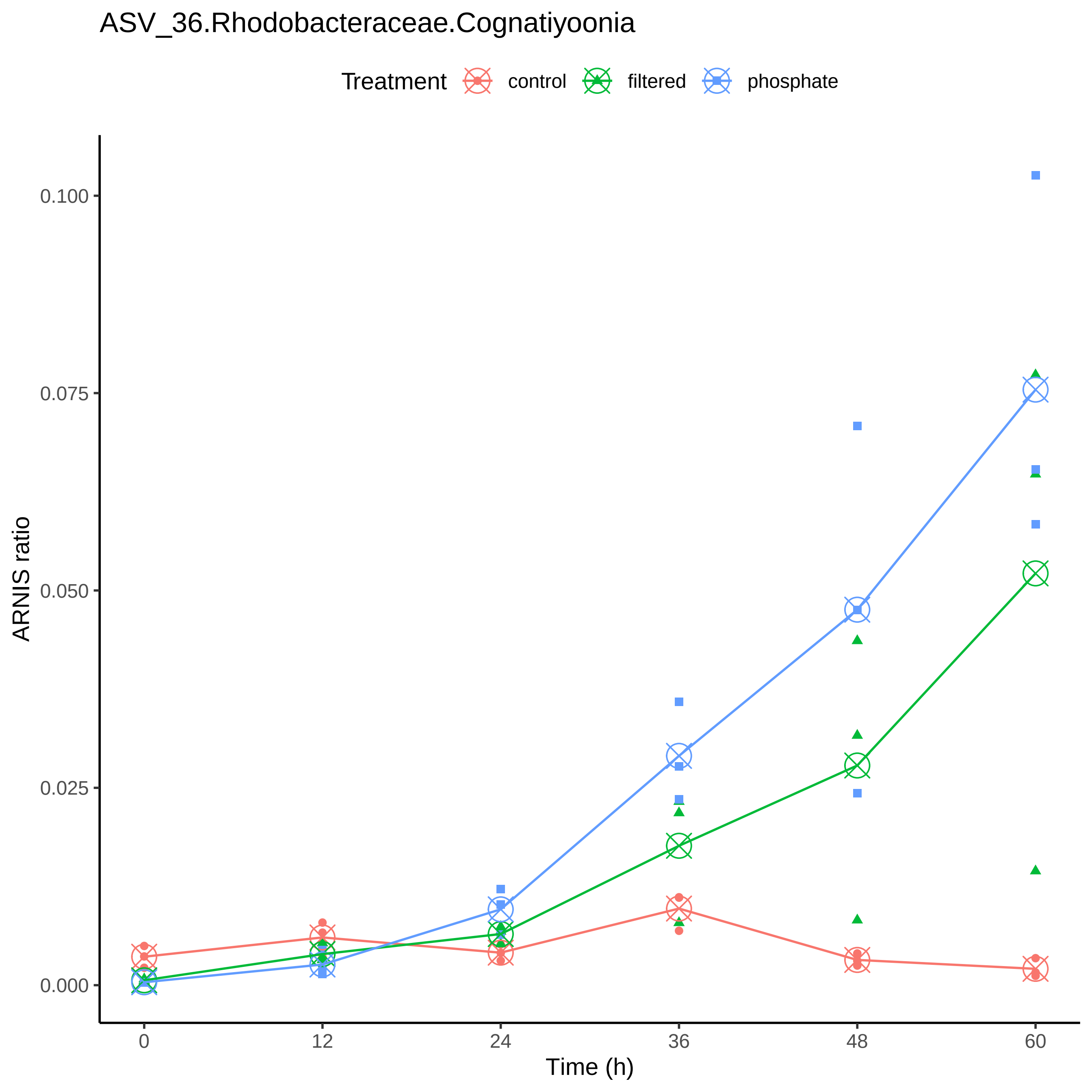
ASV_36.Rhodobacteraceae.Cognatiyoonia
Treatment
control
filtered
phosphate
ARNIS ratio
0.100
0.075
0.050
0.025
0.000
0
12
24
36
48
60
Time (h)

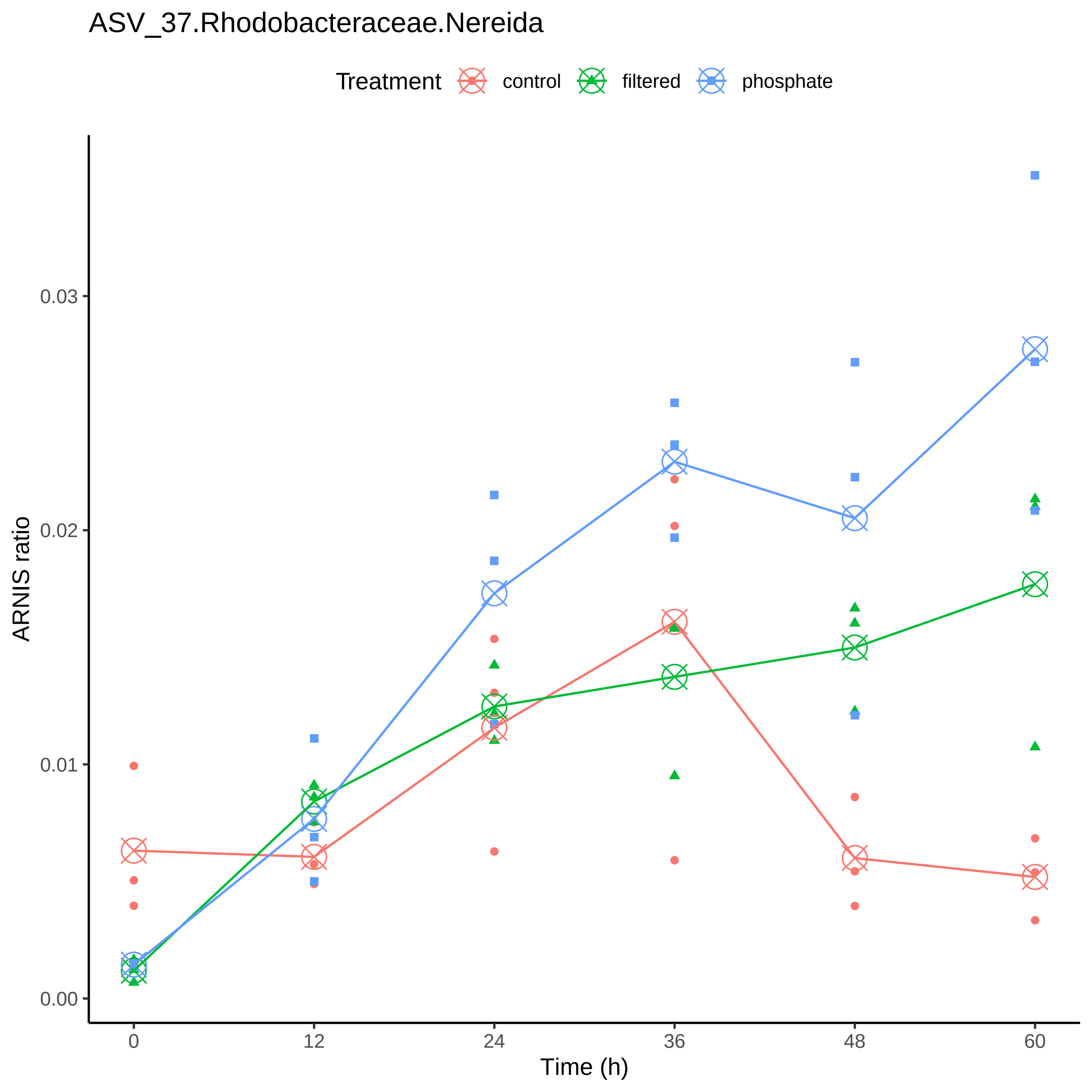

ASV_37.Rhodobacteraceae.Nereida
Treatment
control
filtered
phosphate
ARNIS ratio
0.00
0.01
0.02
0.03
0
12
24
36
48
60
Time (h)

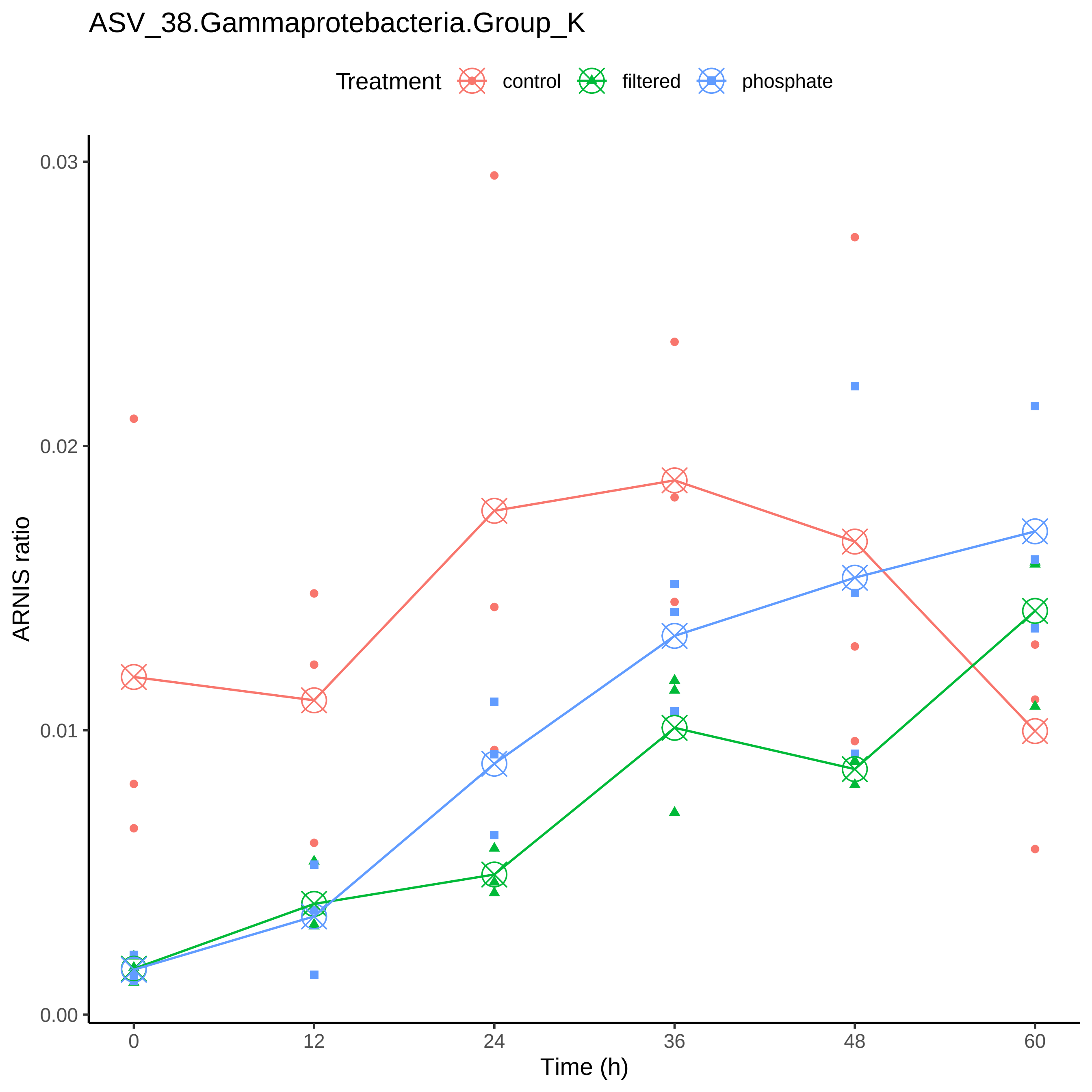
ASV_38.Gammaprotebacteria.Group_K
Treatment
control
filtered
phosphate
ARNIS ratio
0.00
0.01
0.02
0.03
Time (h)
0
12
24
36
48
60

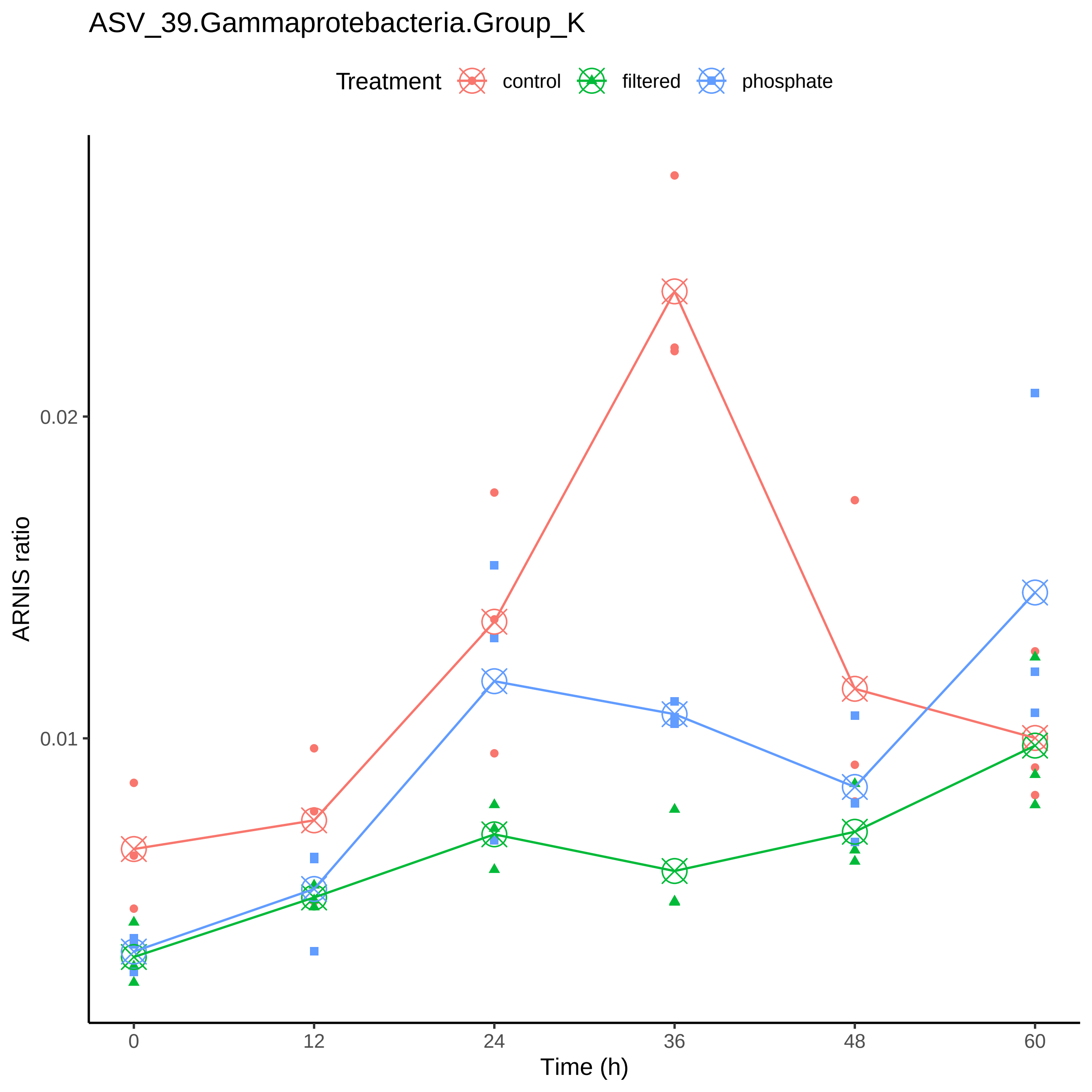

ASV_39.Gammaprotebacteria.Group_K
Treatment
control
filtered
phosphate
ARNIS ratio
0.02
0.01
0
12
24
36
48
60
Time (h)

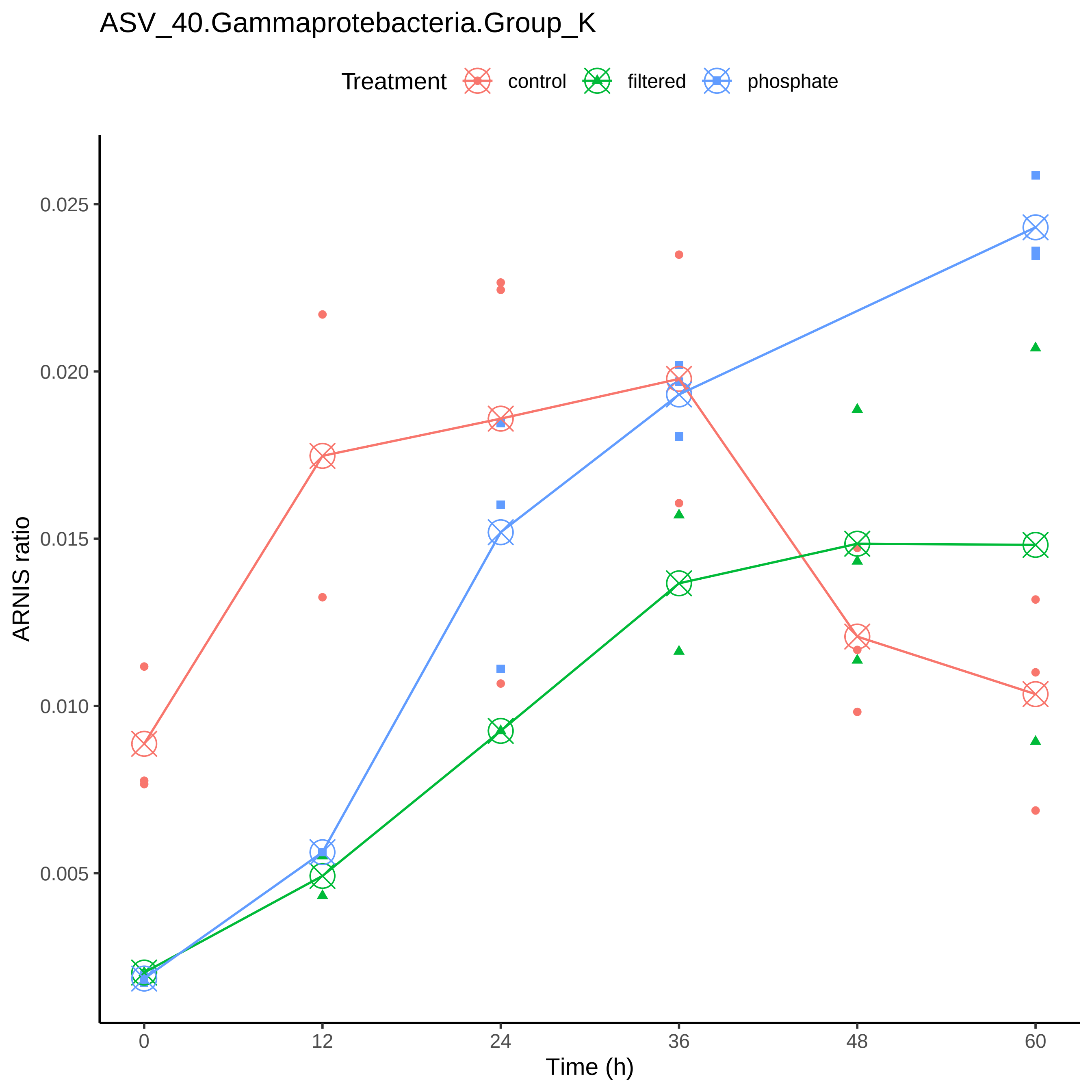
ASV_40.Gammaprotebacteria.Group_K
Treatment
control
filtered
phosphate
0.025
0.020
0.015
0.010
0.005
ARNIS ratio
0
12
24
36
48
60
Time (h)

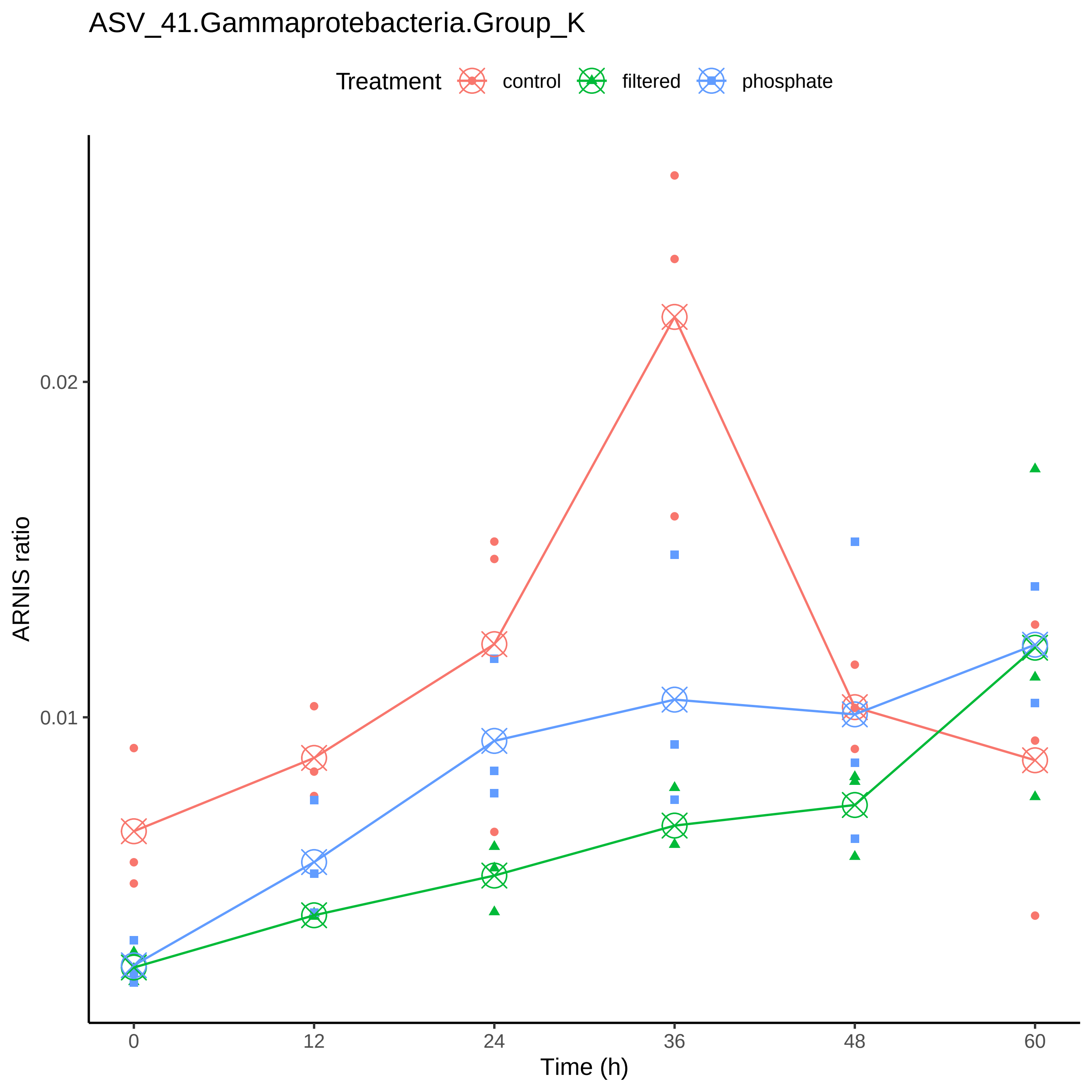

ASV_41.Gammaprotebacteria.Group_K
Treatment
control
filtered
phosphate
0.02
0.01
ARNIS ratio
0
12
24
36
48
60
Time (h)

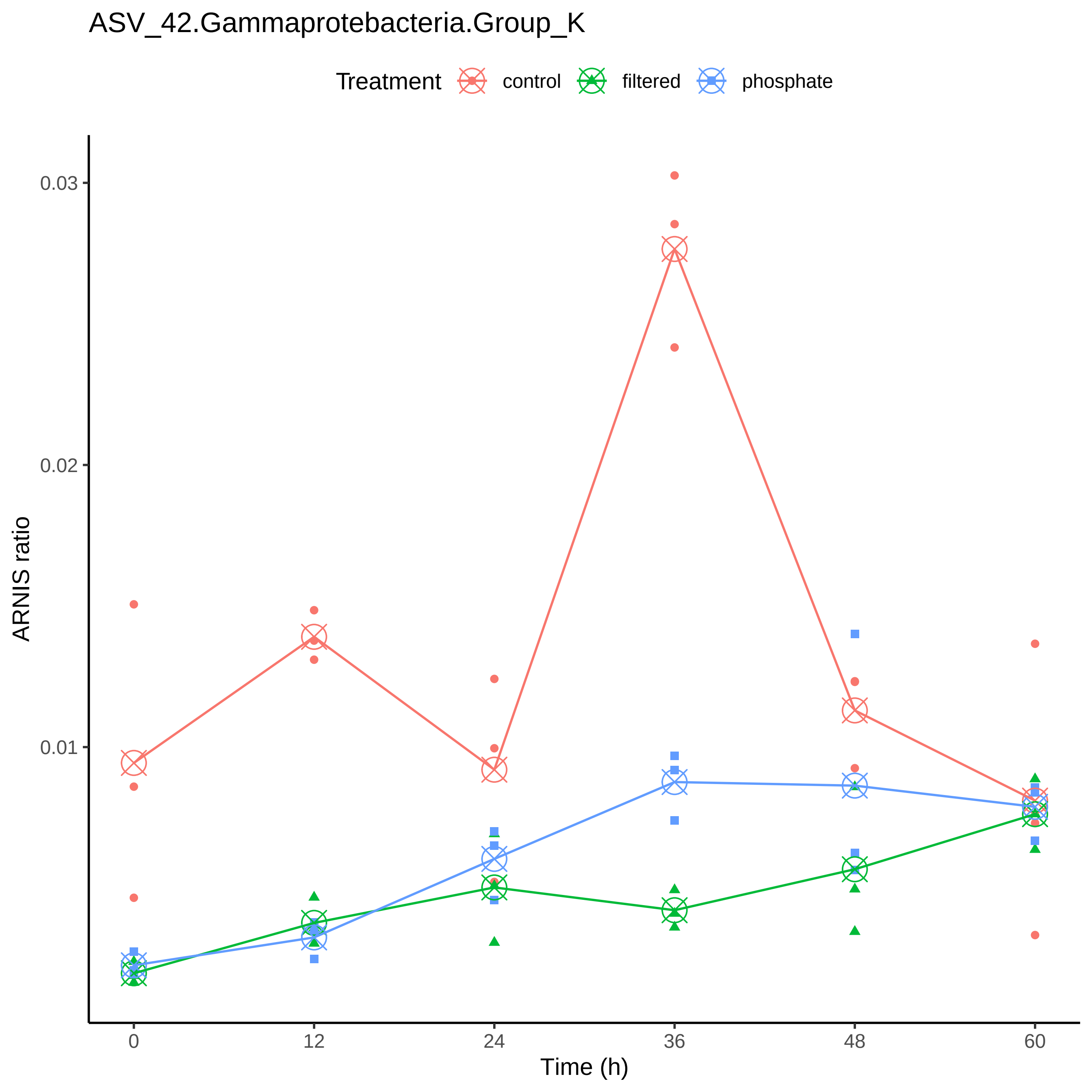
ASV_42.Gammaprotebacteria.Group_K
Treatment
control
filtered
phosphate
ARNIS ratio
0.03
0.02
0.01
0
12
24
36
48
60
Time (h)

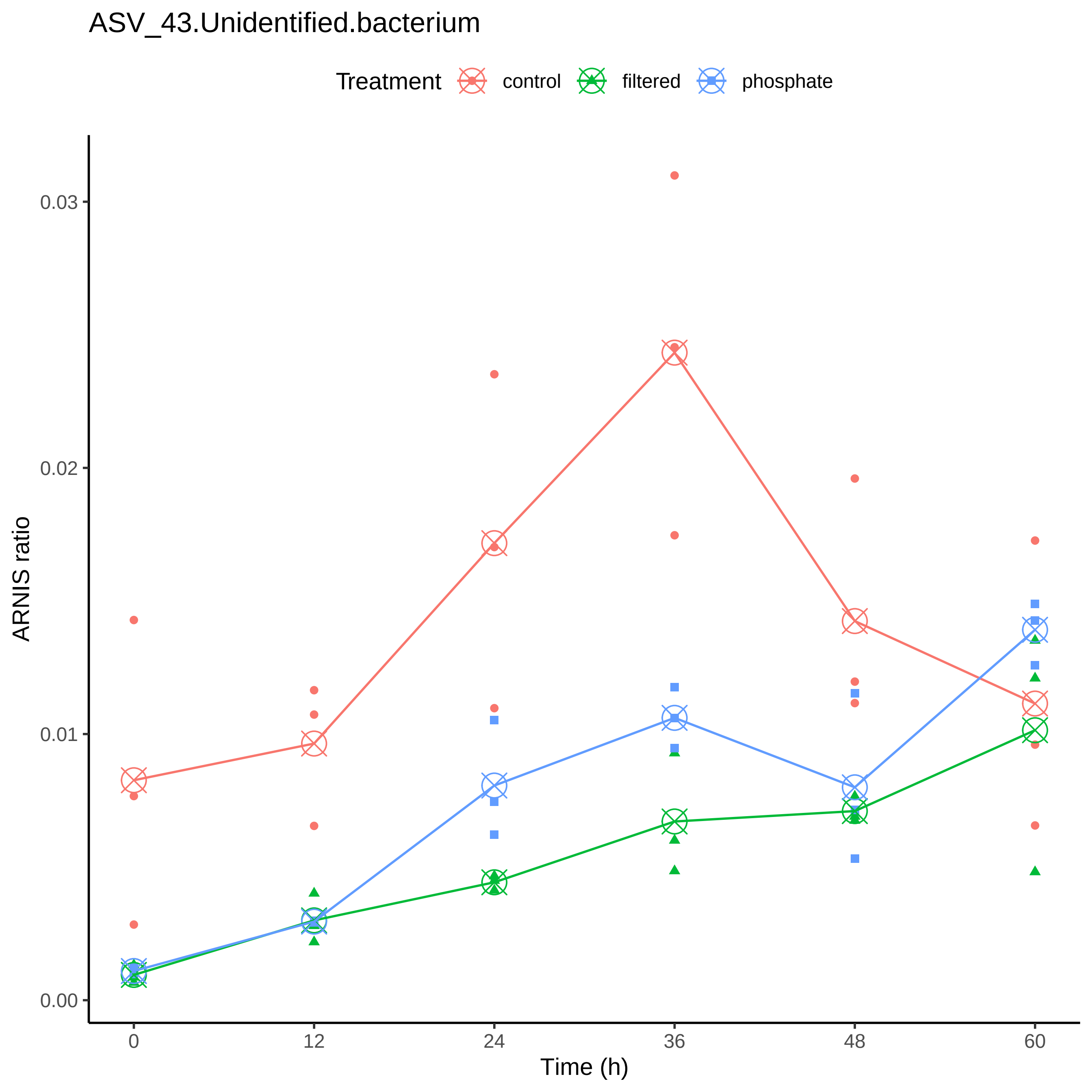

ASV_43.Unidentified.bacterium
Treatment
control
filtered
phosphate
ARNIS ratio
0.00
0.01
0.02
0.03
Time (h)
0
12
24
36
48
60

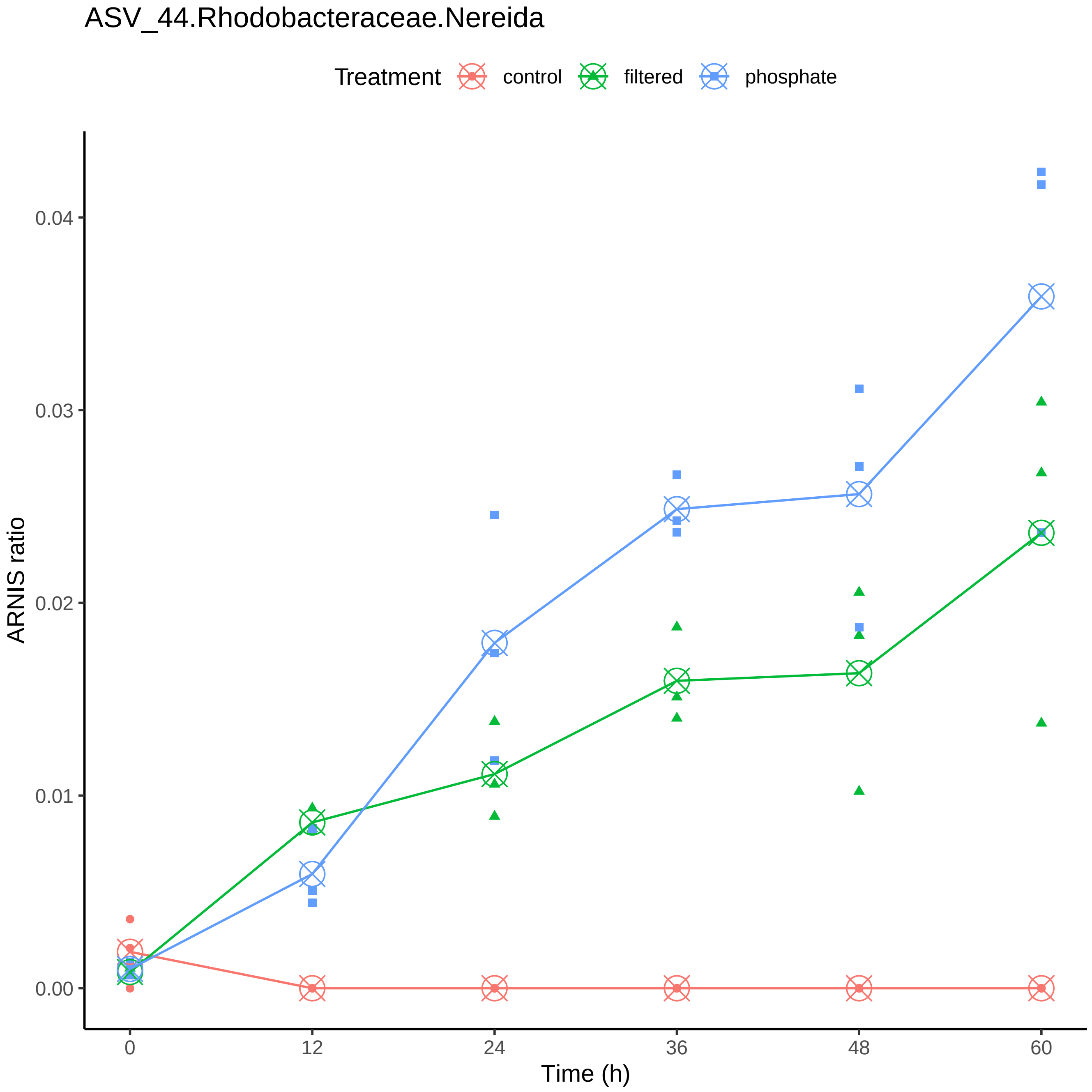
ASV_44.Rhodobacteraceae.Nereida
Treatment
control
filtered
phosphate
ARNIS ratio
0.00
0.01
0.02
0.03
0.04
Time (h)
0
12
24
36
48
60

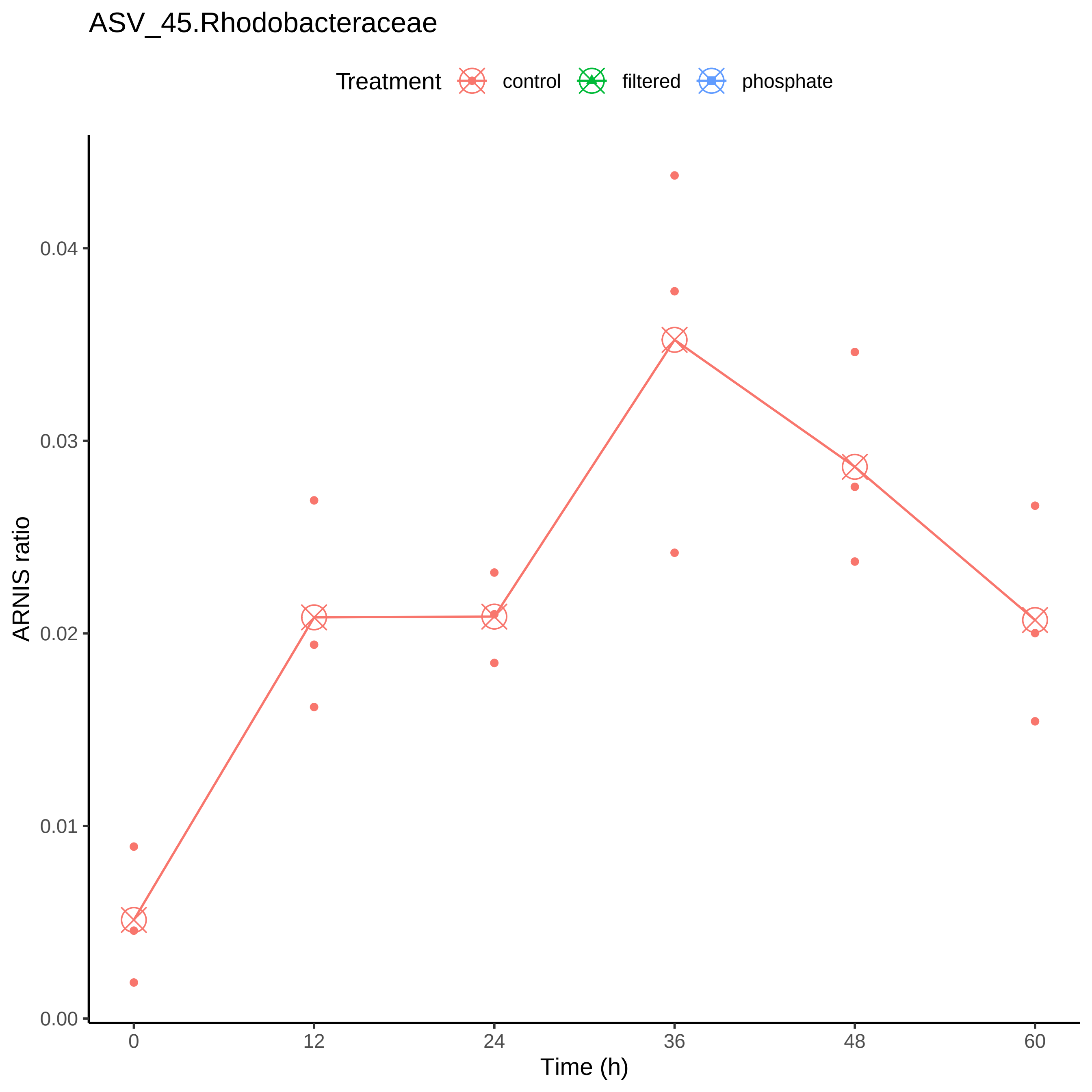

ASV_45.Rhodobacteraceae
Treatment
control
filtered
phosphate
0.04
0.03
0.02
0.01
0.00
ARNIS ratio
0
12
24
36
48
60
Time (h)

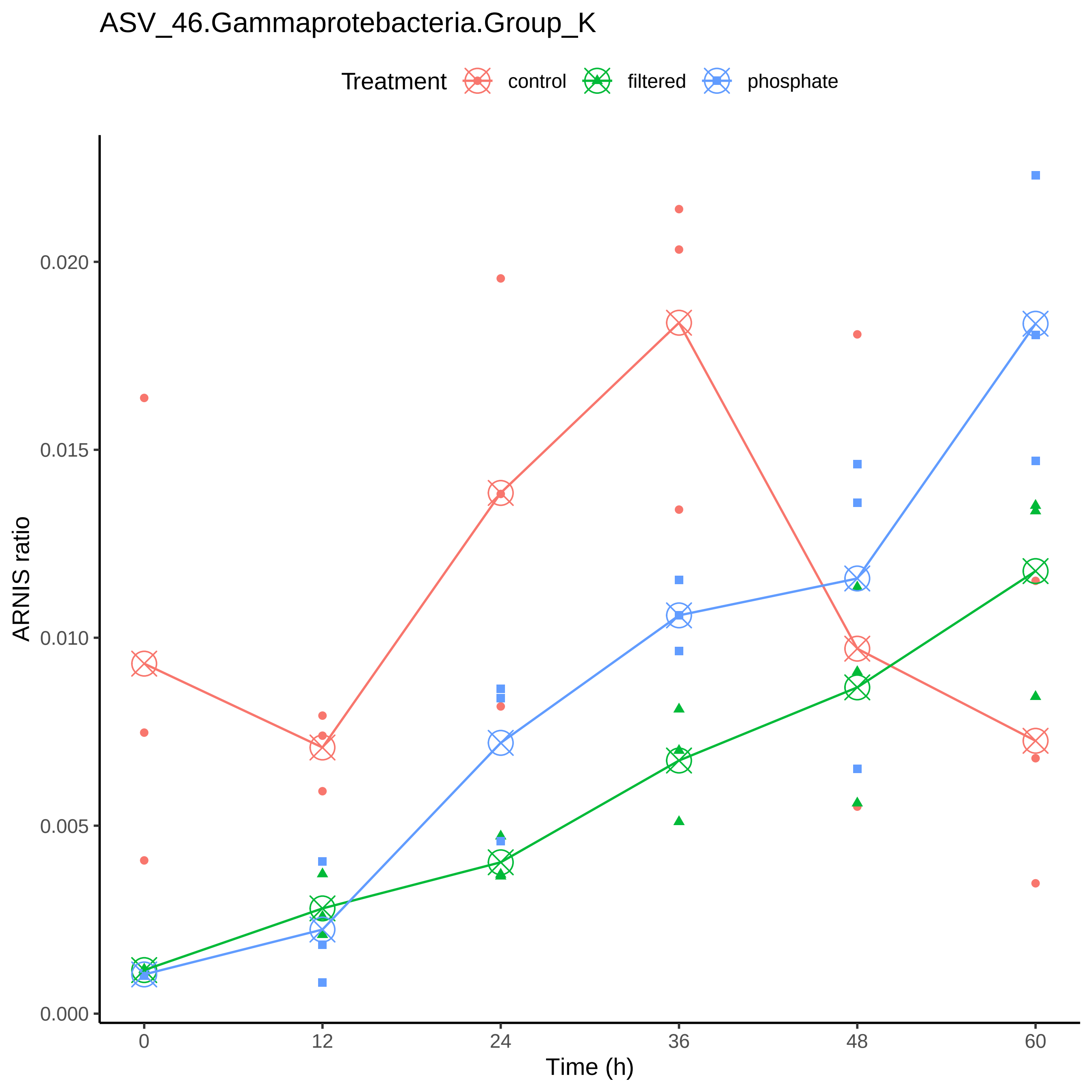

ASV_46.Gammaprotebacteria.Group_K
Treatment
control
filtered
phosphate
ARNIS ratio
0.000
0.005
0.010
0.015
0.020
0
12
24
36
48
60
Time (h)

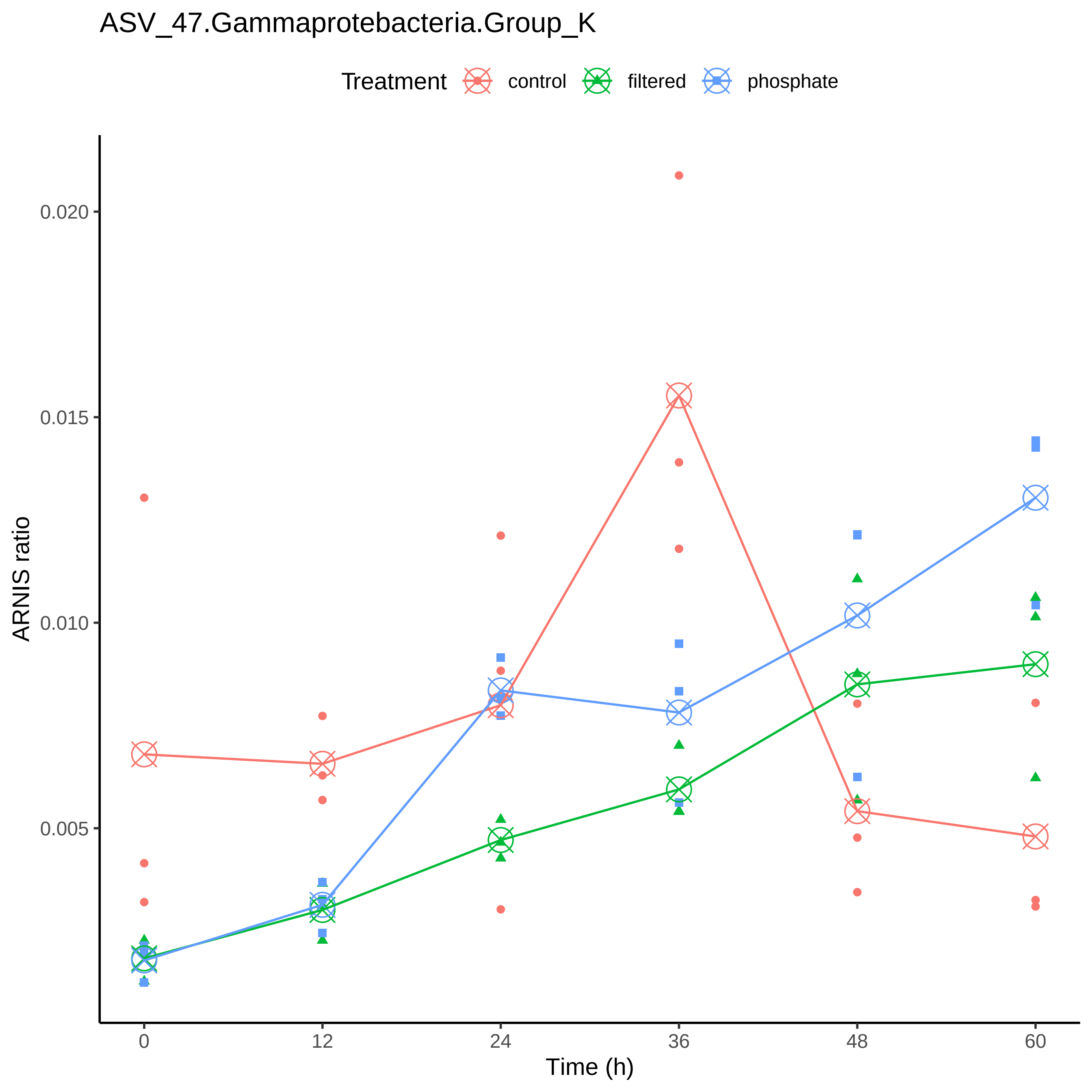
ASV_47.Gammaprotebacteria.Group_K
Treatment
control
filtered
phosphate
ARNIS ratio
0.005
0.010
0.015
0.020
Time (h)
0
12
24
36
48
60

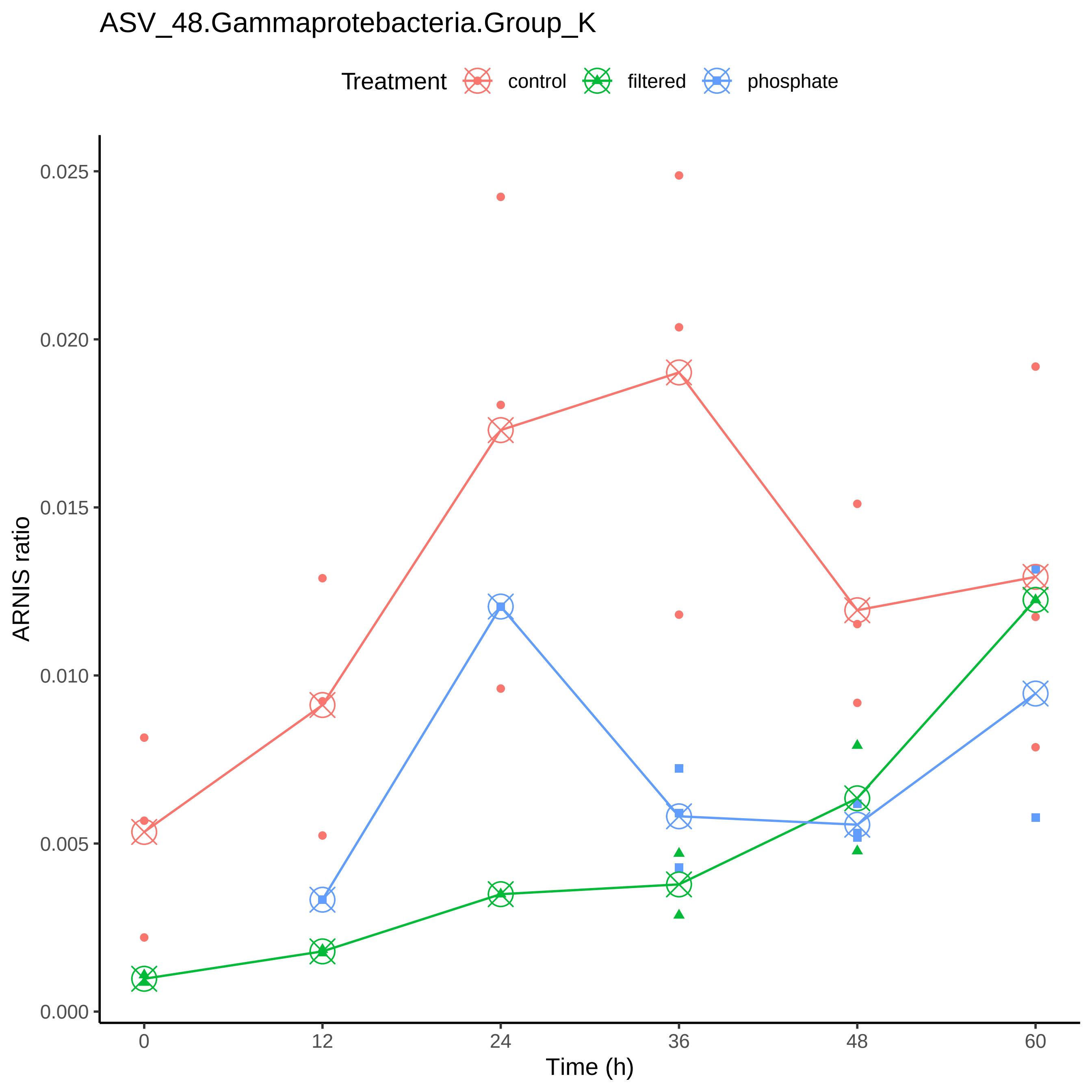

ASV_48.Gammaprotebacteria.Group_K
Treatment
control
filtered
phosphate
ARNIS ratio
0.025
0.020
0.015
0.010
0.005
0.000
0
12
24
36
48
60
Time (h)

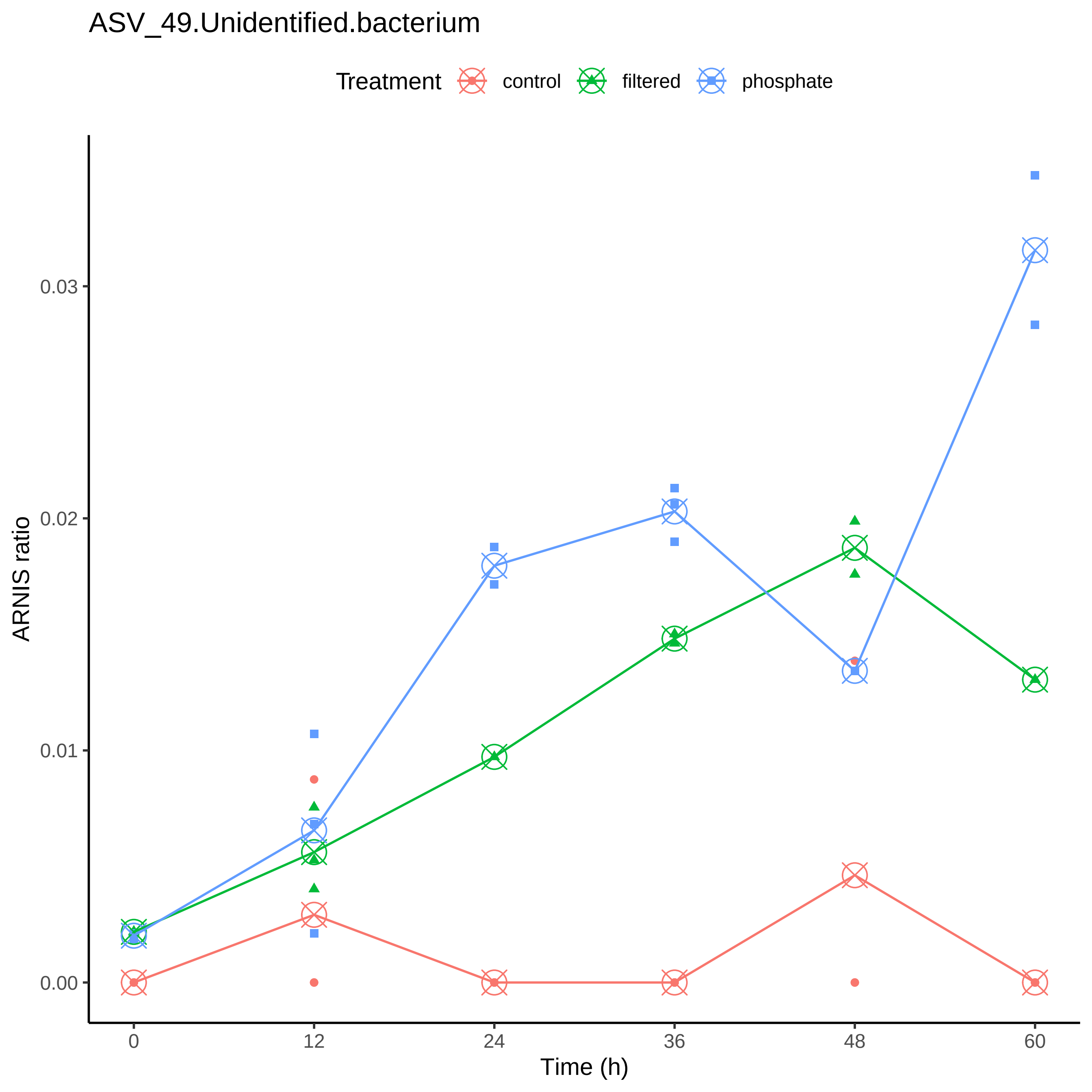

ASV_49.Unidentified.bacterium
Treatment
control
filtered
phosphate
ARNIS ratio
0.03
0.02
0.01
0.00
0
12
24
36
48
60
Time (h)

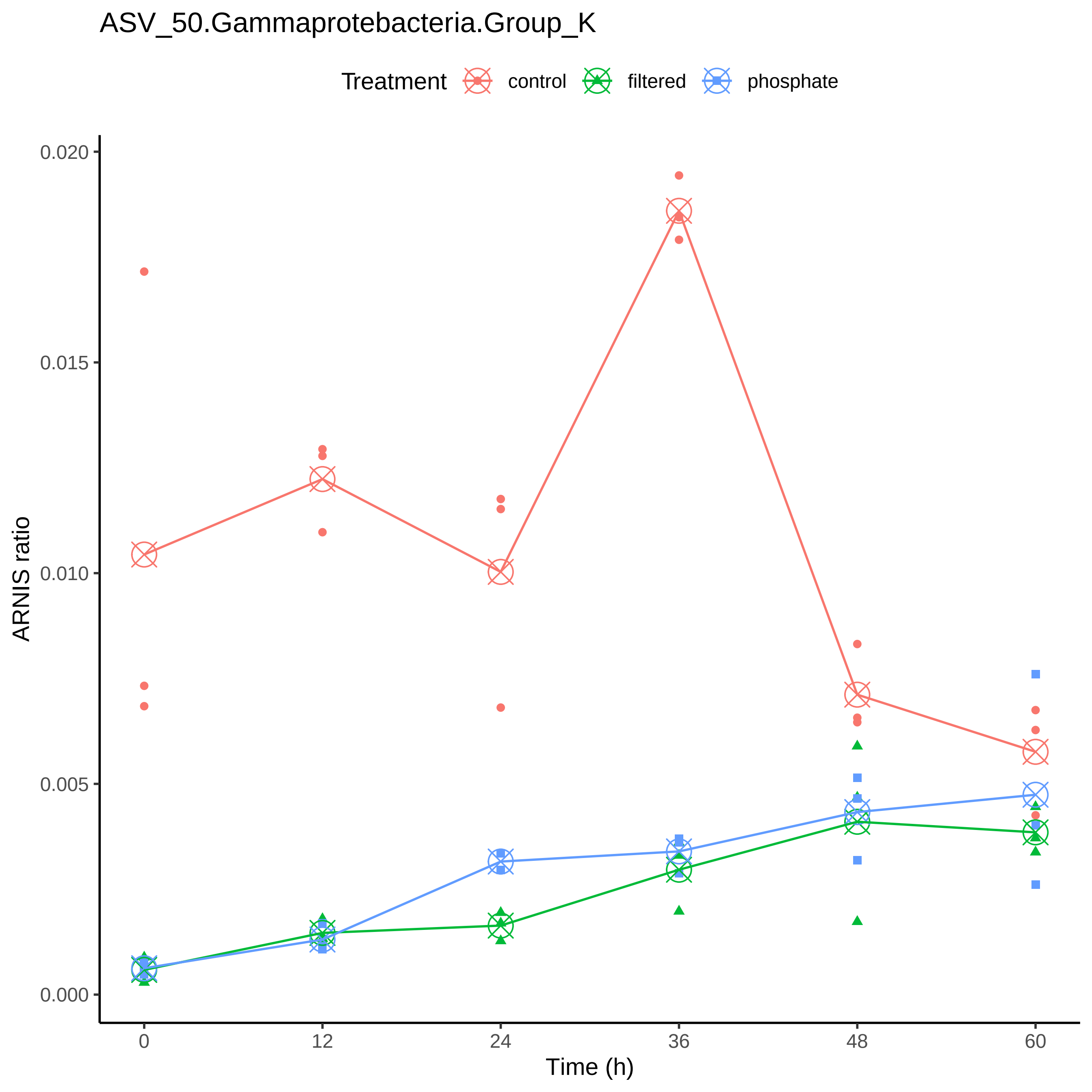
ASV_50.Gammaprotebacteria.Group_K
Treatment
control
filtered
phosphate
ARNIS ratio
0.020
0.015
0.010
0.005
0.000
0
12
24
36
48
60
Time (h)

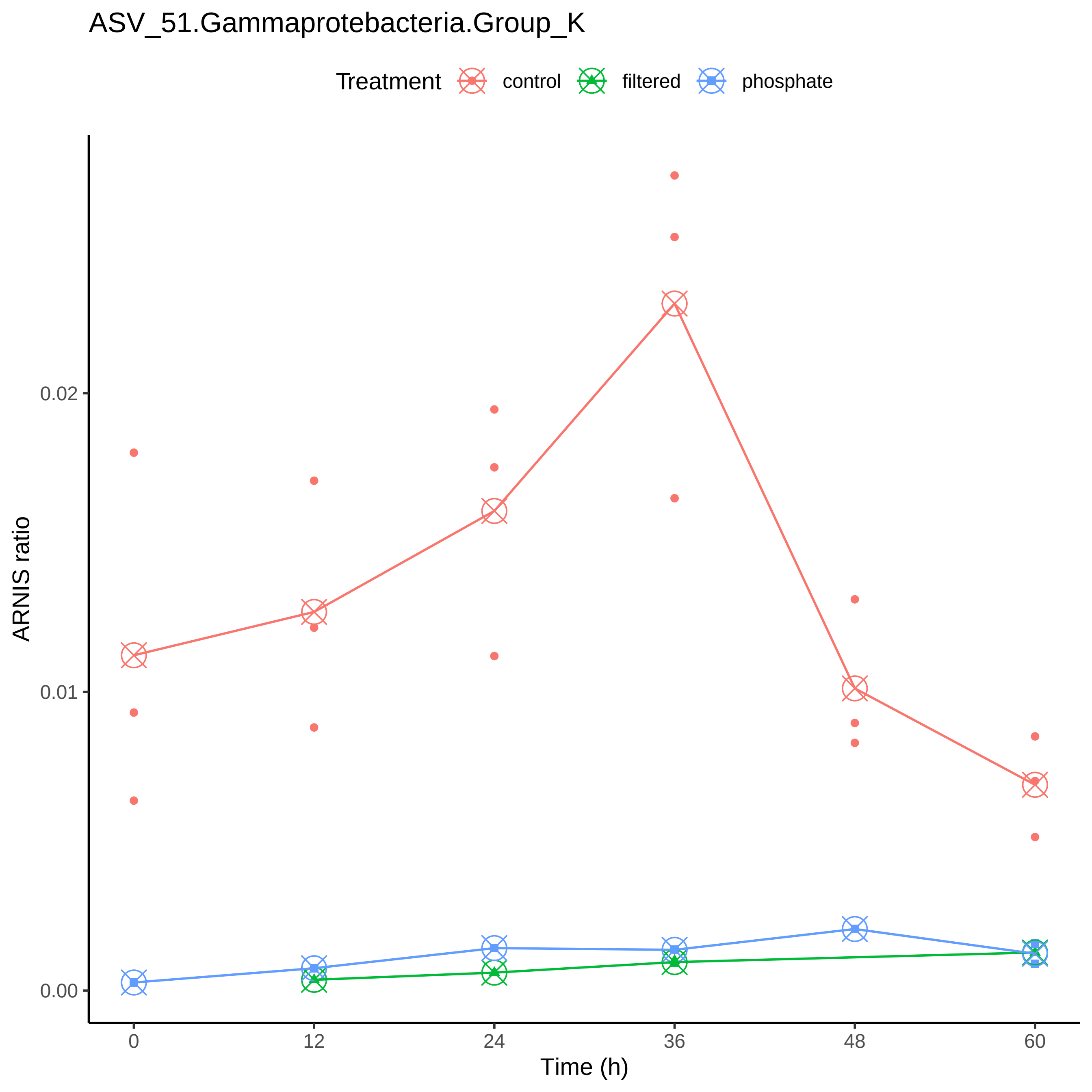

ASV_51.Gammaprotebacteria.Group_K
Treatment
control
filtered
phosphate
ARNIS ratio
0.02
0.01
0.00
0
12
24
36
48
60
Time (h)

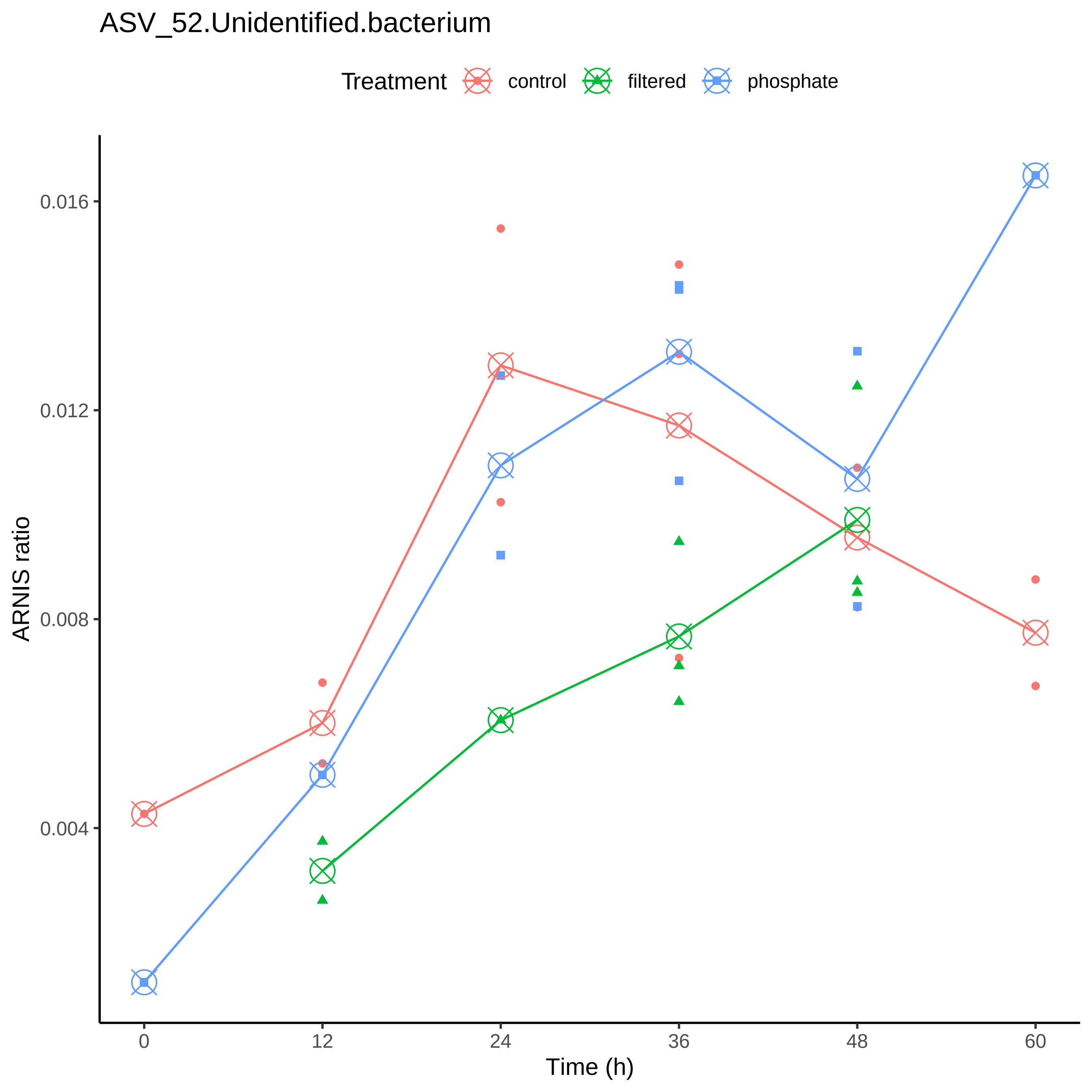
ASV_52.Unidentified.bacterium
Treatment
control
filtered
phosphate
ARNIS ratio
0.016
0.012
0.008
0.004
Time (h)
0
12
24
36
48
60

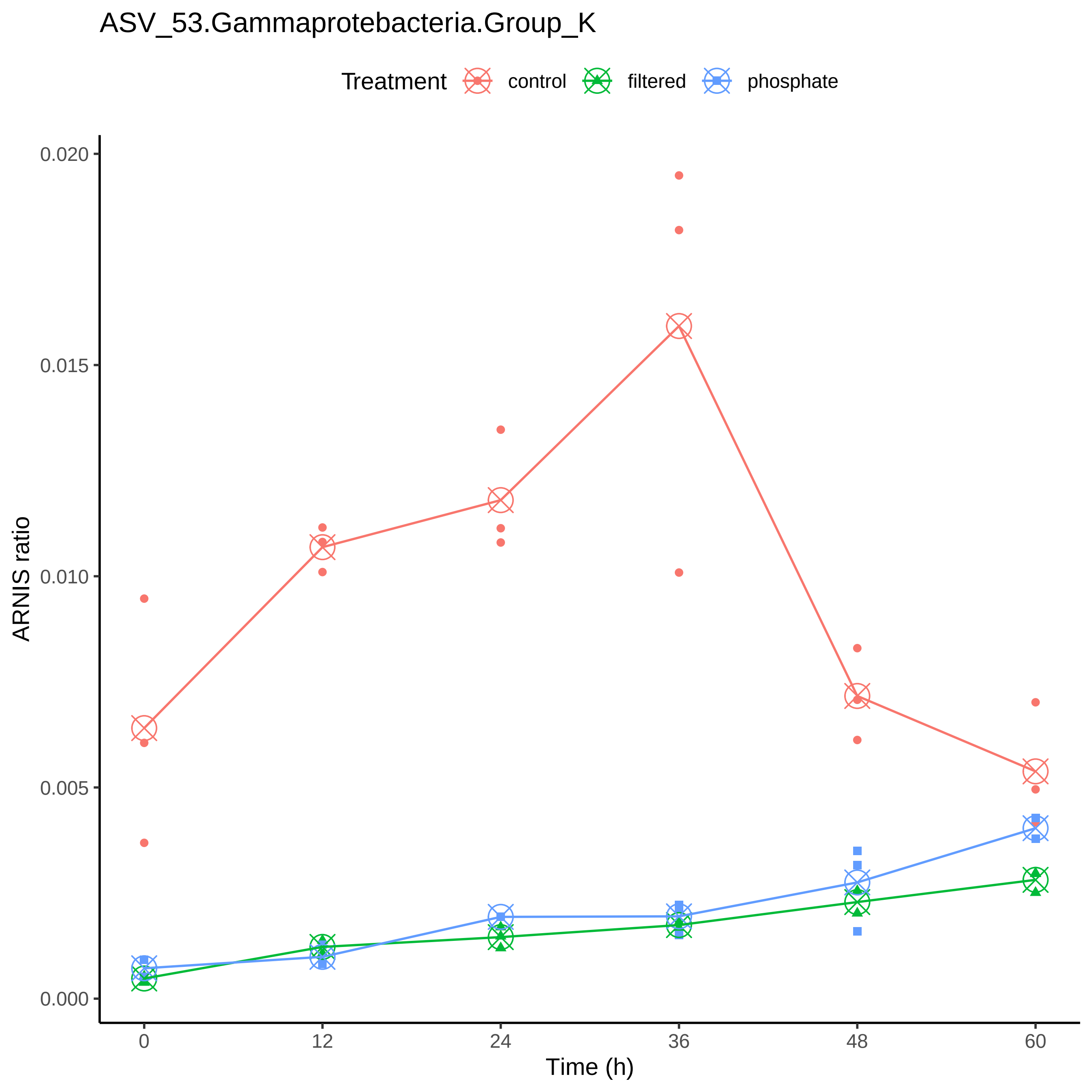

ASV_53.Gammaprotebacteria.Group_K
Treatment
control
filtered
phosphate
ARNIS ratio
0.020
0.015
0.010
0.005
0.000
Time (h)
0
12
24
36
48
60

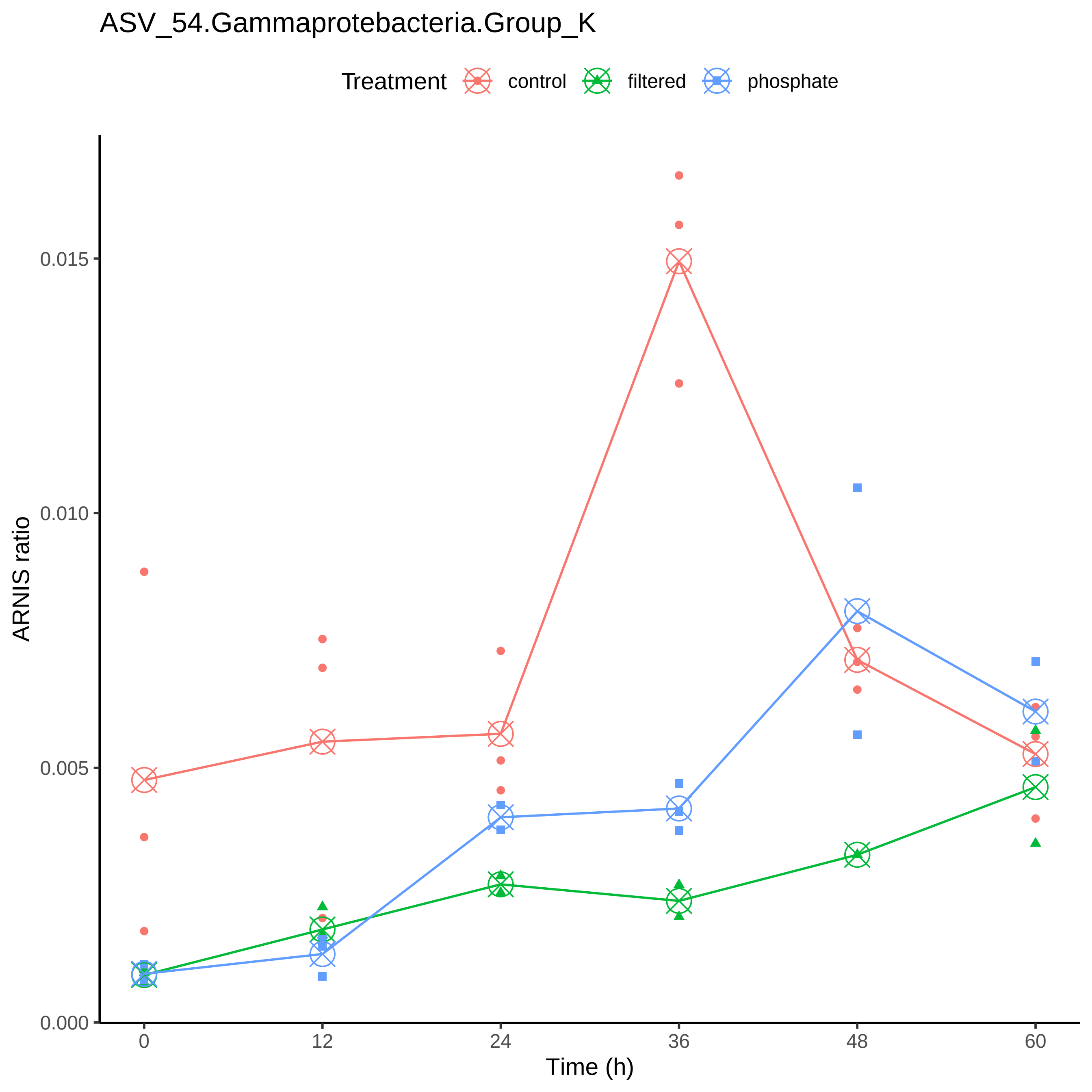
ASV_54.Gammaprotebacteria.Group_K
Treatment
control
filtered
phosphate
ARNIS ratio
0.000
0.005
0.010
0.015
Time (h)
0
12
24
36
48
60

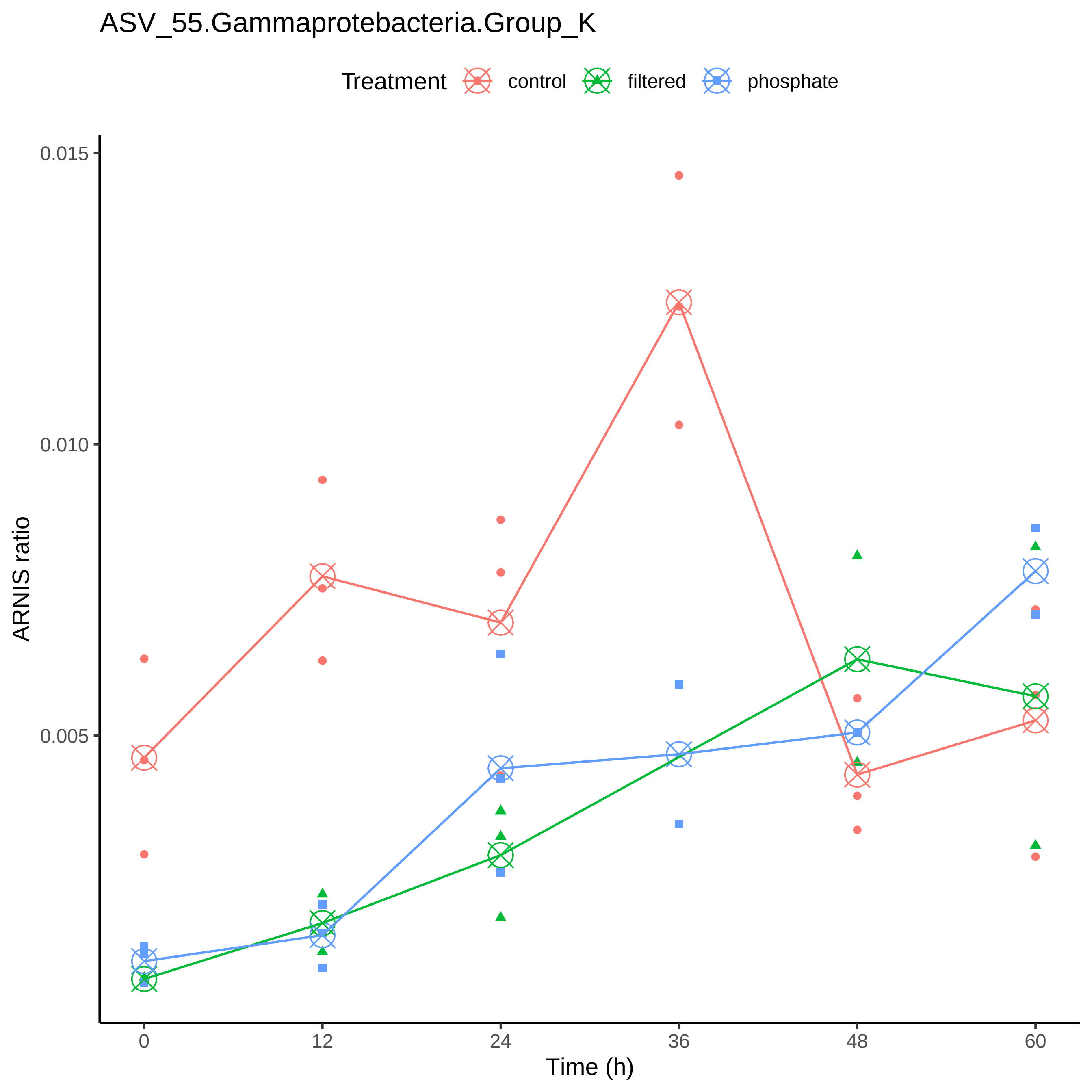

ASV_55.Gammaprotebacteria.Group_K
Treatment
control
filtered
phosphate
0.015
0.010
0.005
ARNIS ratio
0
12
24
36
48
60
Time (h)

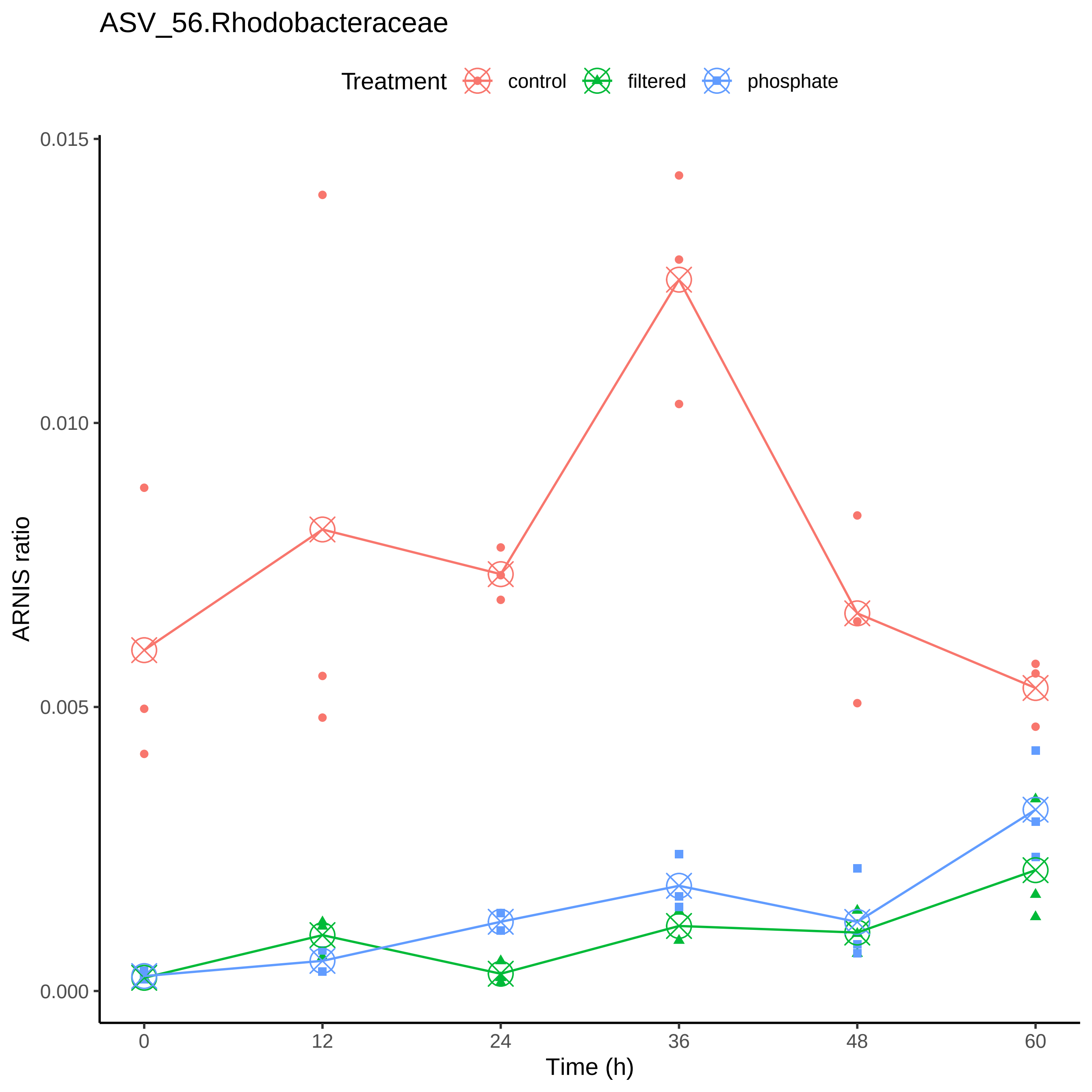

ASV_56.Rhodobacteraceae
Treatment
control
filtered
phosphate
ARNIS ratio
0.015
0.010
0.005
0.000
Time (h)
0
12
24
36
48
60

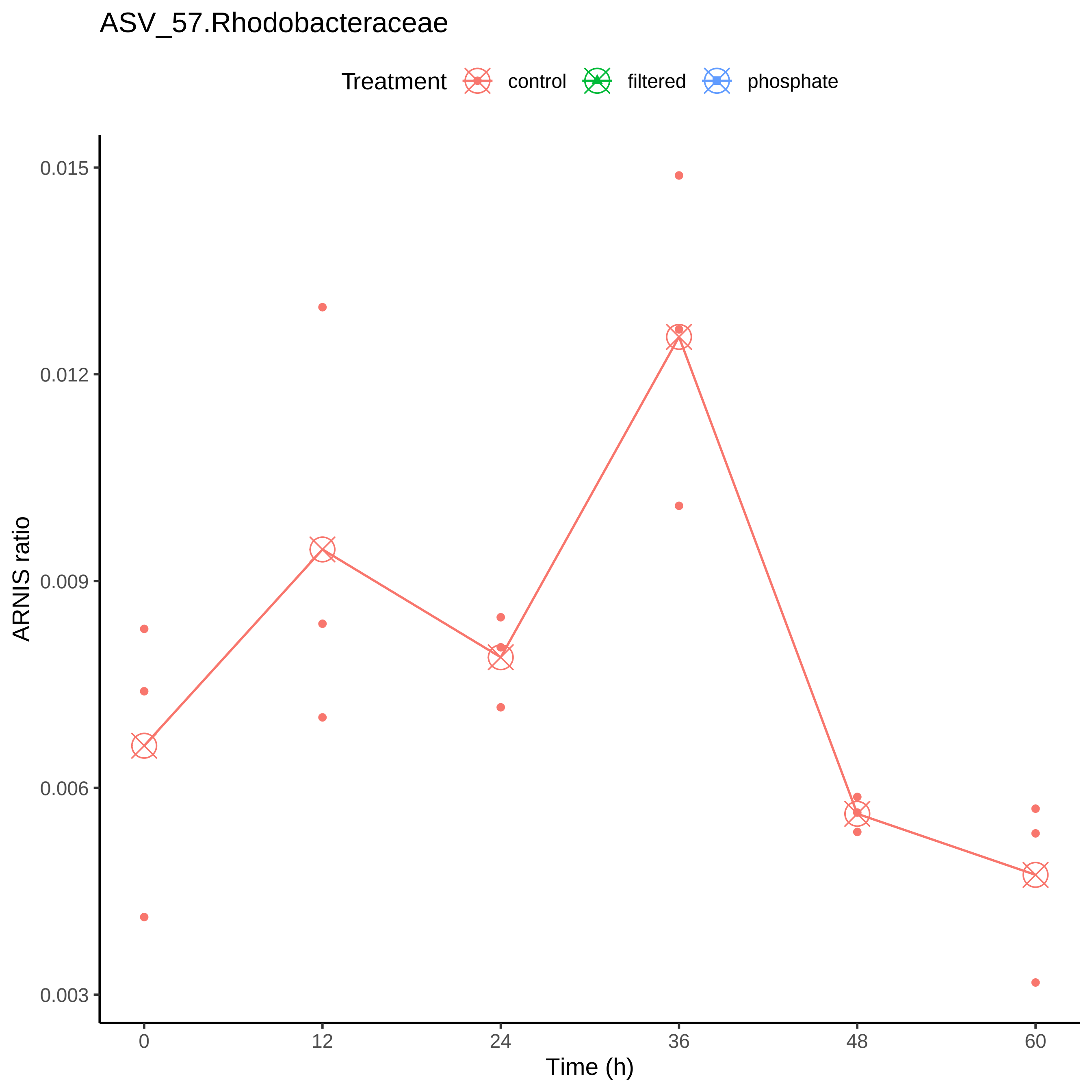
ASV_57.Rhodobacteraceae
Treatment
control
filtered
phosphate
ARNIS ratio
0.015
0.012
0.009
0.006
0.003
Time (h)
0
12
24
36
48
60

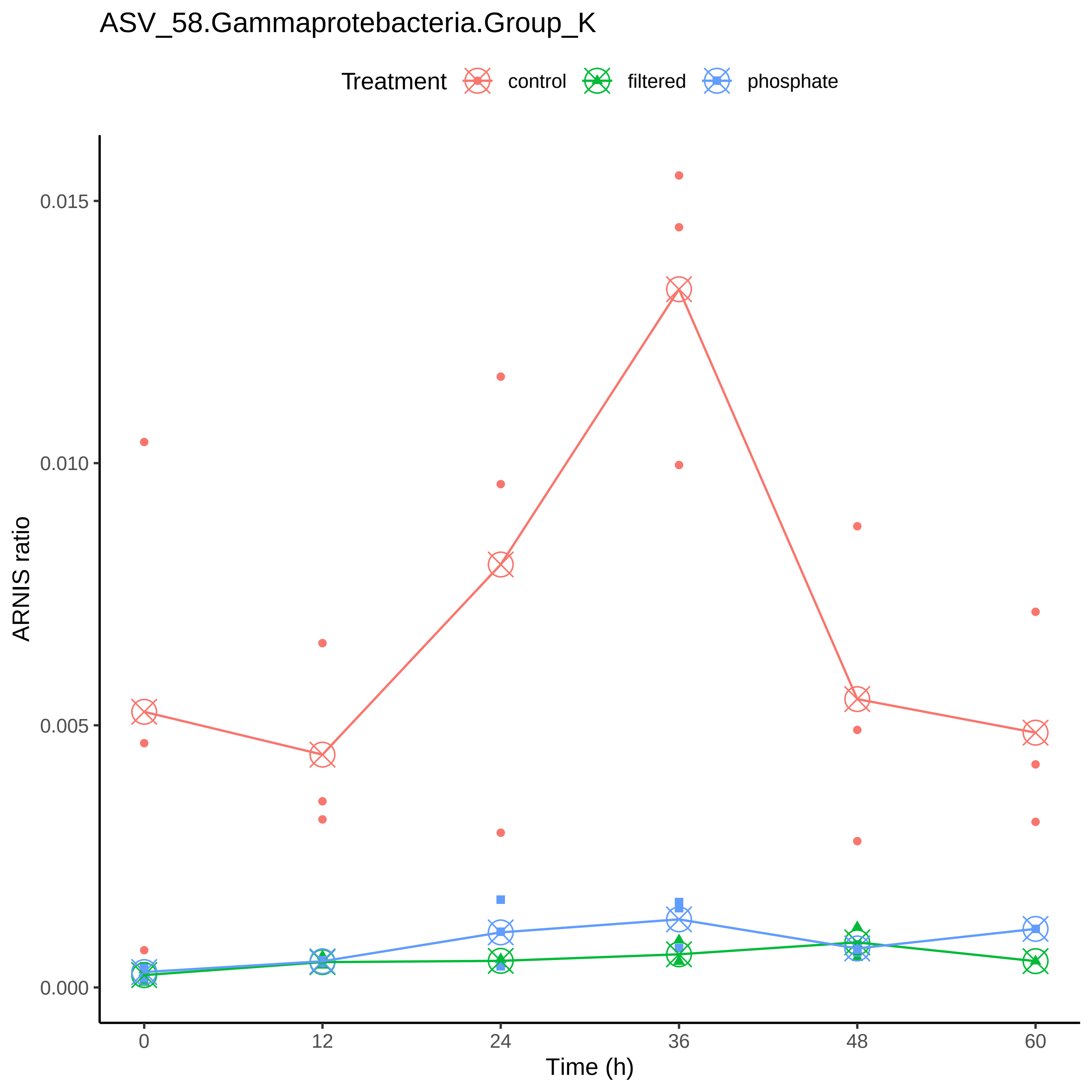

ASV_58.Gammaprotebacteria.Group_K
Treatment
control
filtered
phosphate
ARNIS ratio
0.015
0.010
0.005
0.000
0
12
24
36
48
60
Time (h)

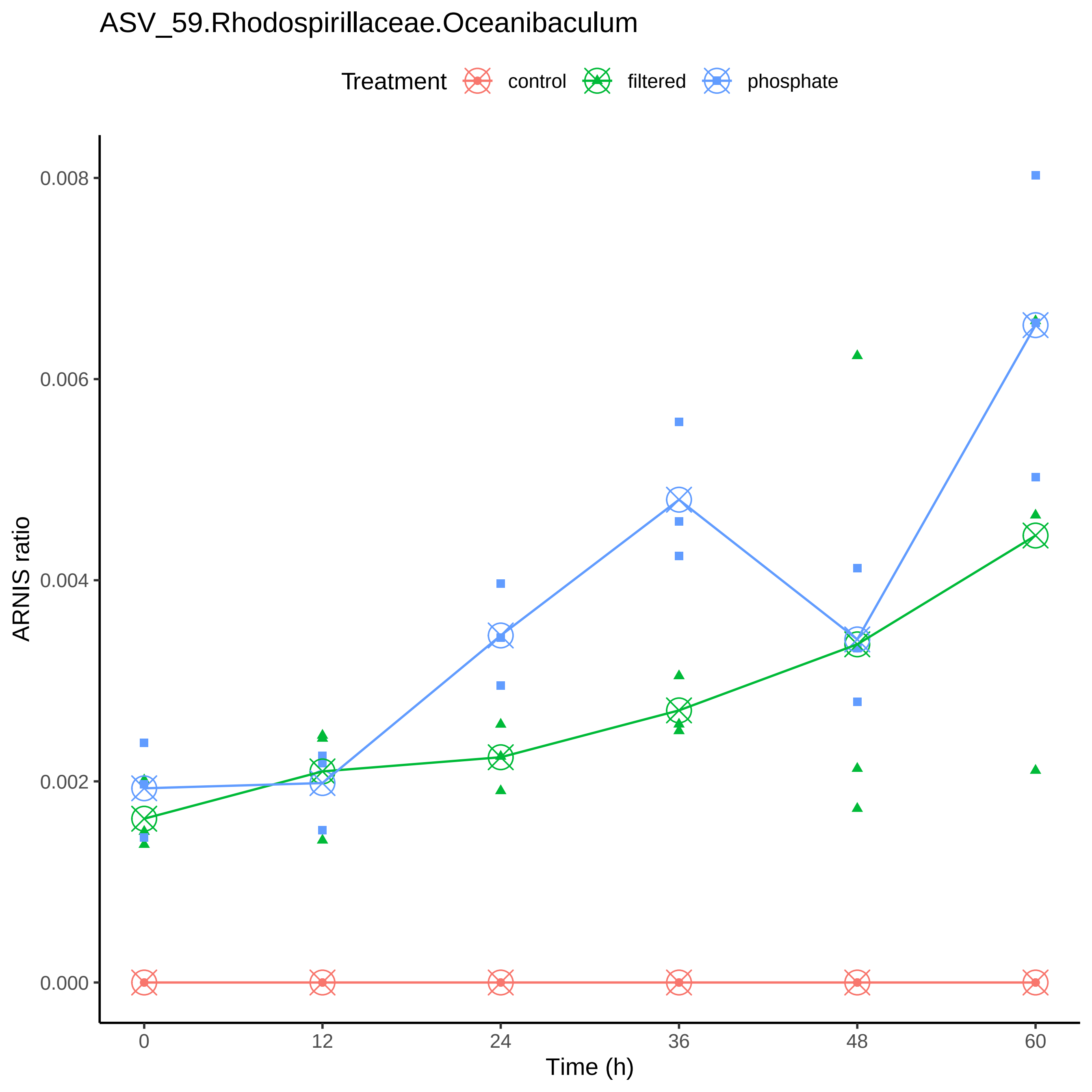

ASV_59.Rhodospirillaceae.Oceanibaculum
Treatment
control
filtered
phosphate
ARNIS ratio
0.000
0.002
0.004
0.006
0.008
Time (h)
0
12
24
36
48
60

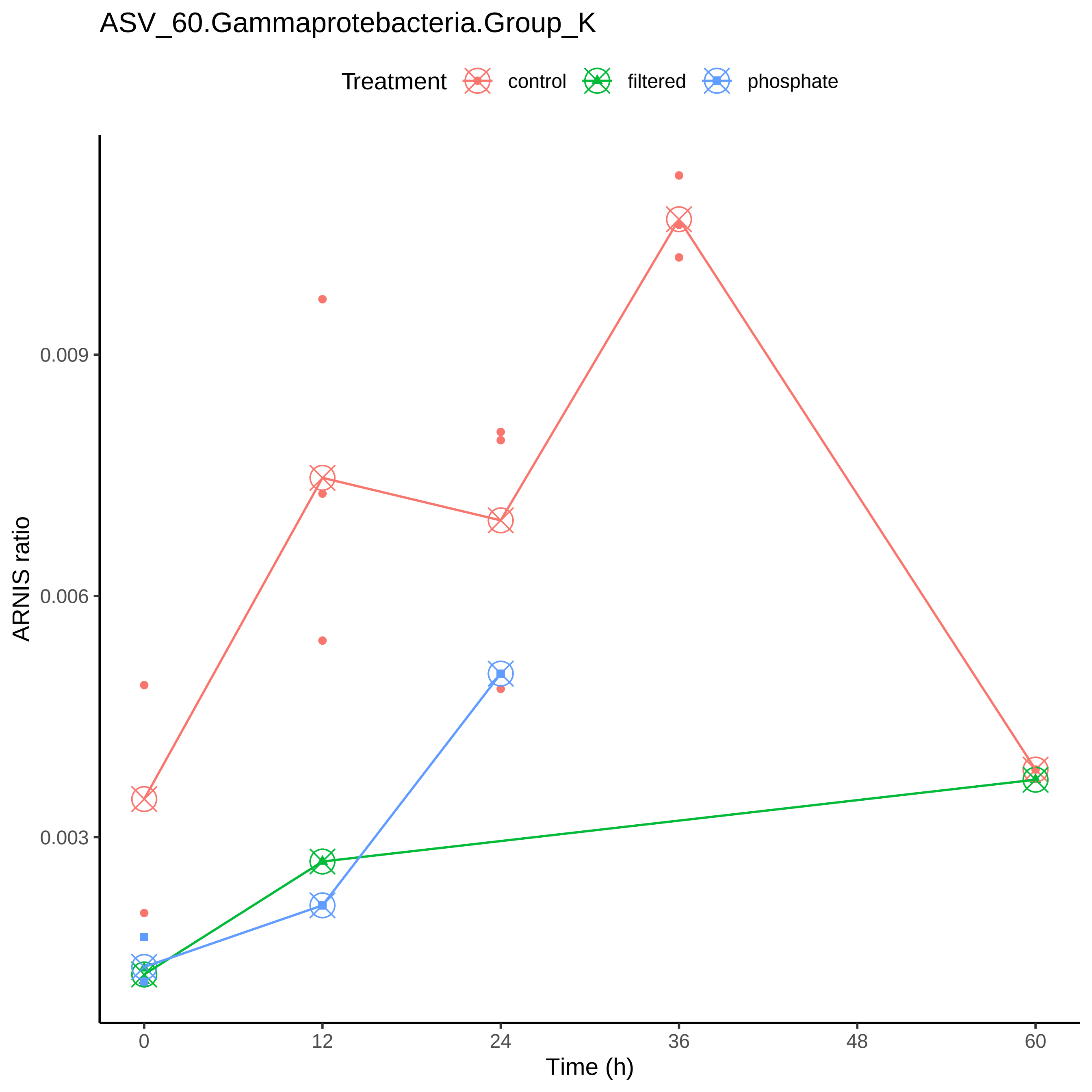
ASV_60.Gammaprotebacteria.Group_K
Treatment
control
filtered
phosphate
0.009
0.006
0.003
ARNIS ratio
0
12
24
36
48
60
Time (h)

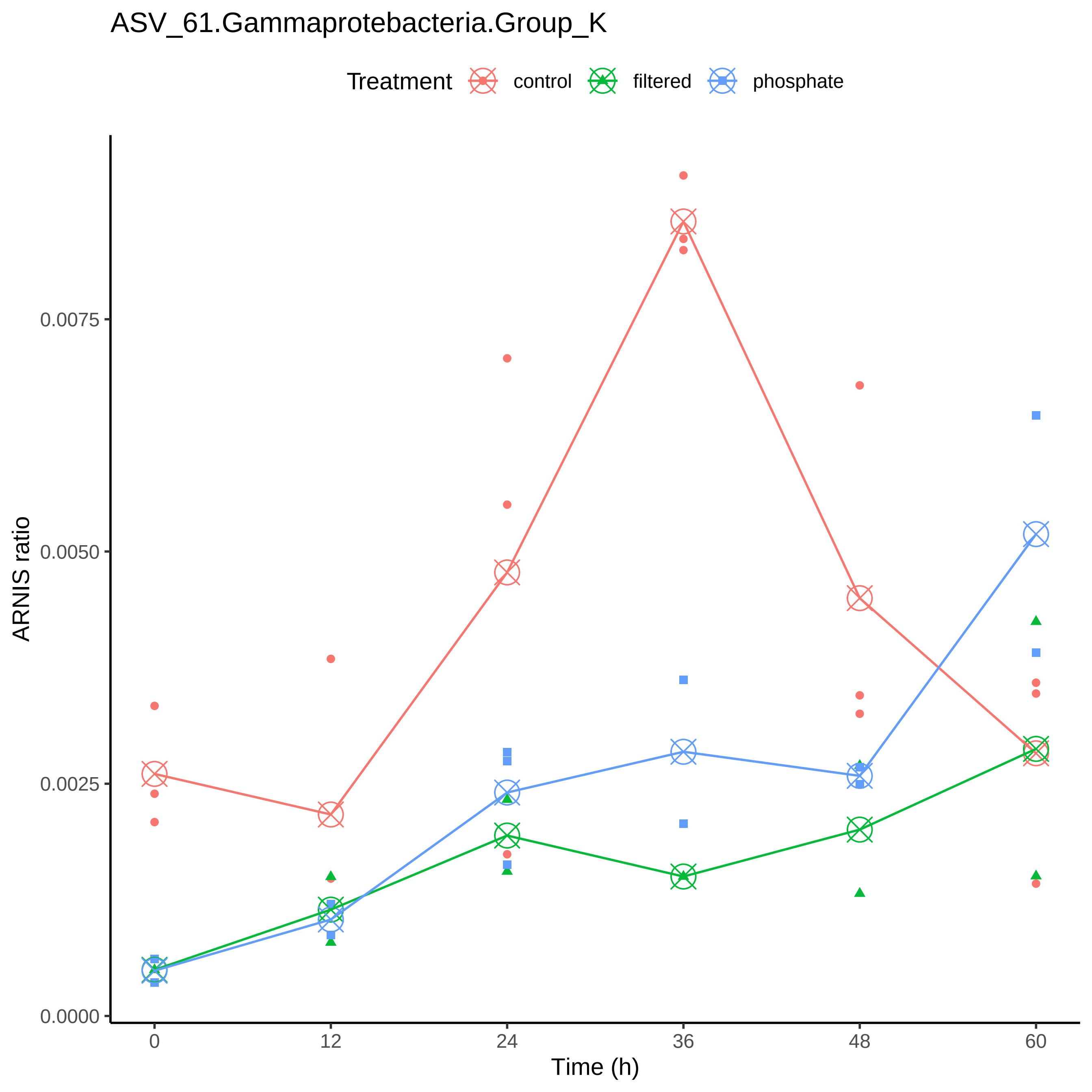

ASV_61.Gammaprotebacteria.Group_K
Treatment
control
filtered
phosphate
ARNIS ratio
0.0000
0.0025
0.0050
0.0075
0
12
24
36
48
60
Time (h)

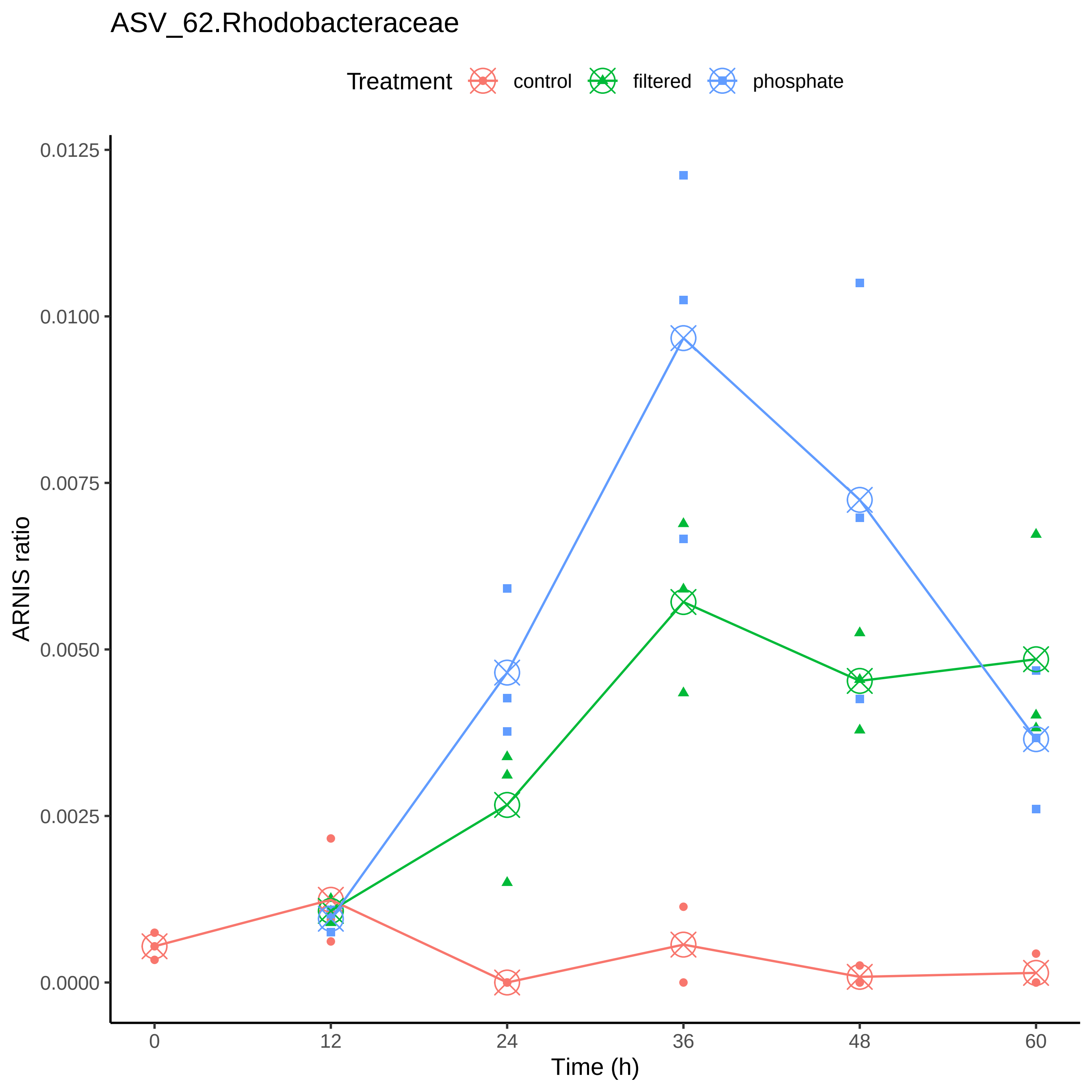

ASV_62.Rhodobacteraceae
Treatment
control
filtered
phosphate
ARNIS ratio
0.0125
0.0100
0.0075
0.0050
0.0025
0.0000
0
12
24
36
48
60
Time (h)

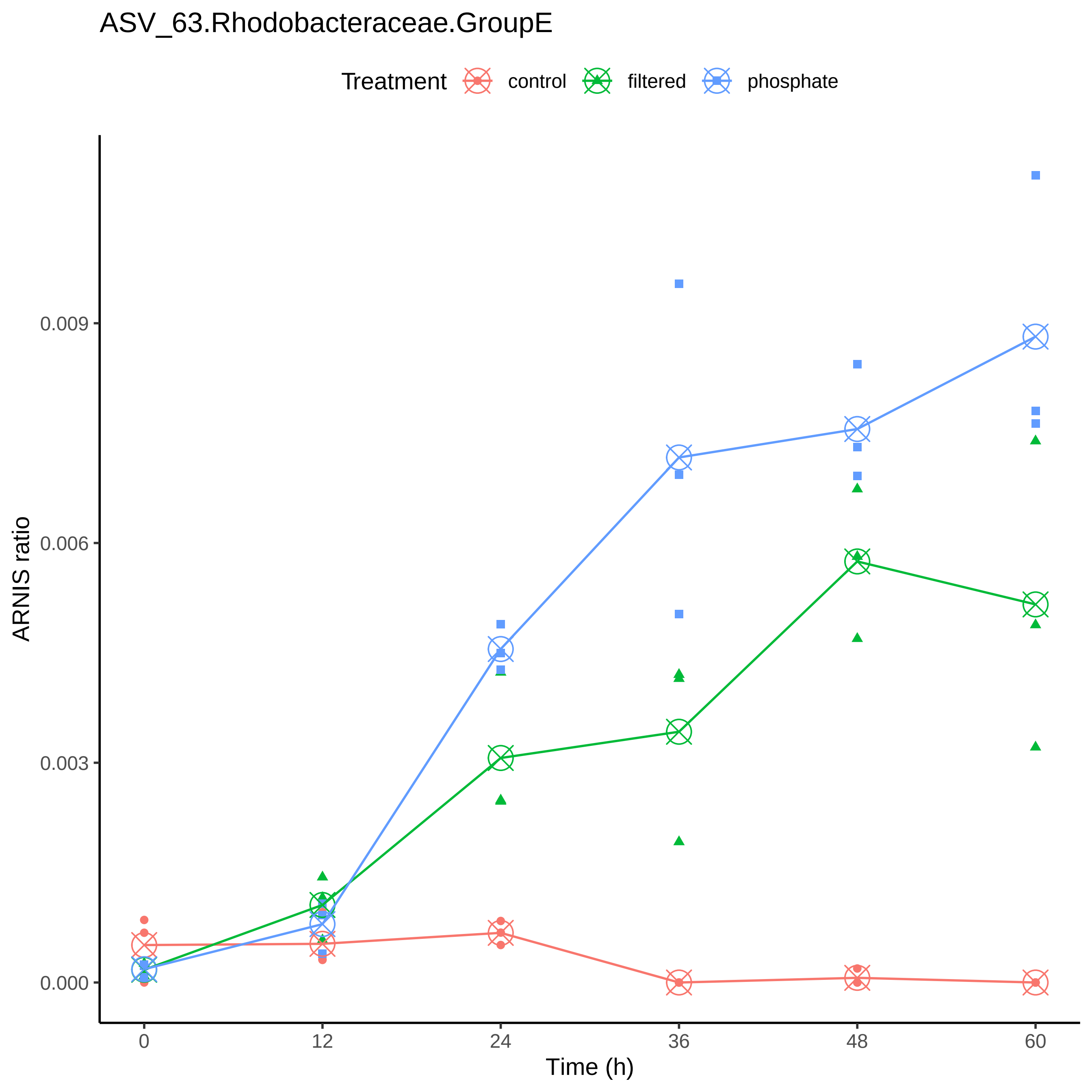

ASV_63.Rhodobacteraceae.GroupE
Treatment
control
filtered
phosphate
ARNIS ratio
0.000
0.003
0.006
0.009
0
12
24
36
48
60
Time (h)

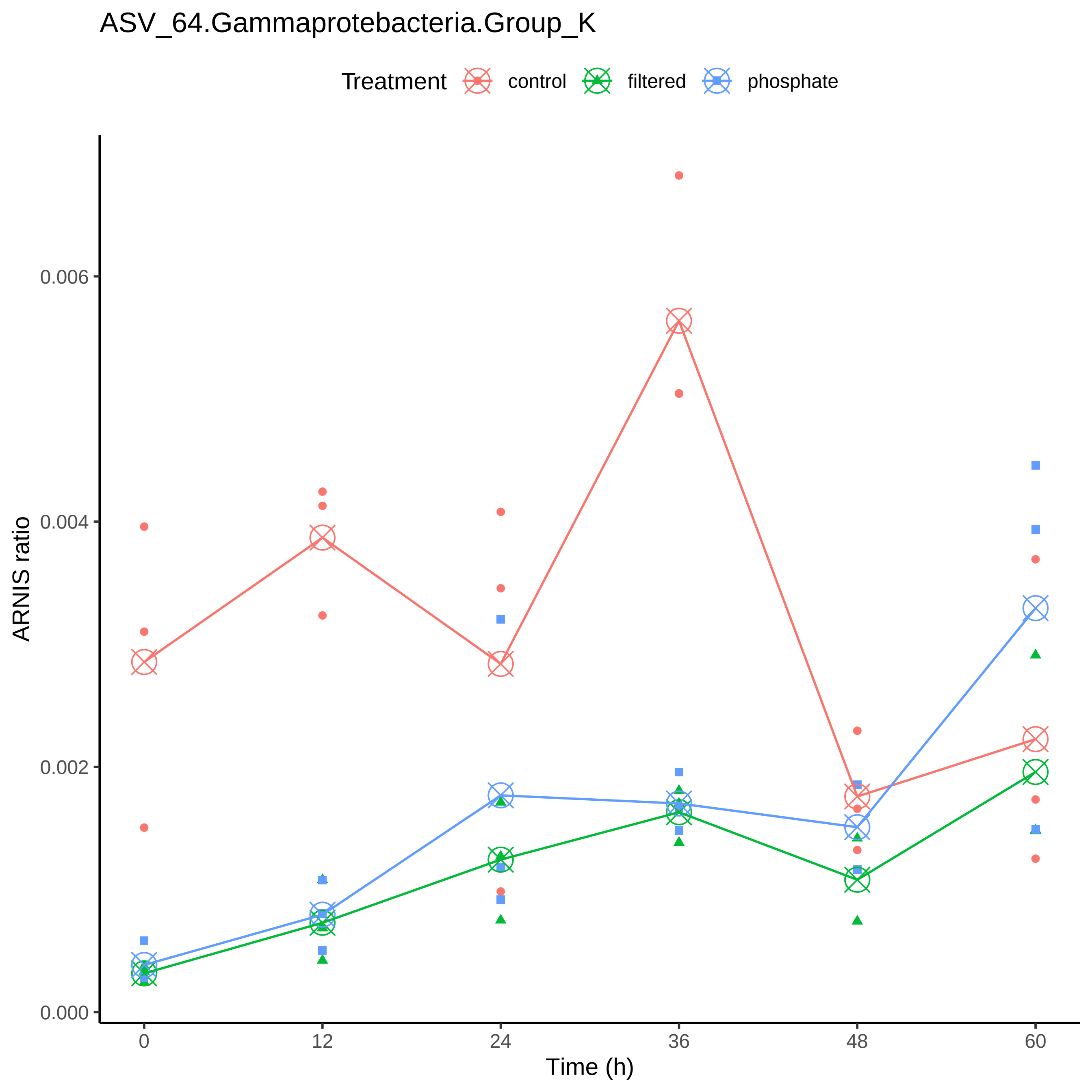
ASV_64.Gammaprotebacteria.Group_K
Treatment
control
filtered
phosphate
ARNIS ratio
0.000
0.002
0.004
0.006
0
12
24
36
48
60
Time (h)

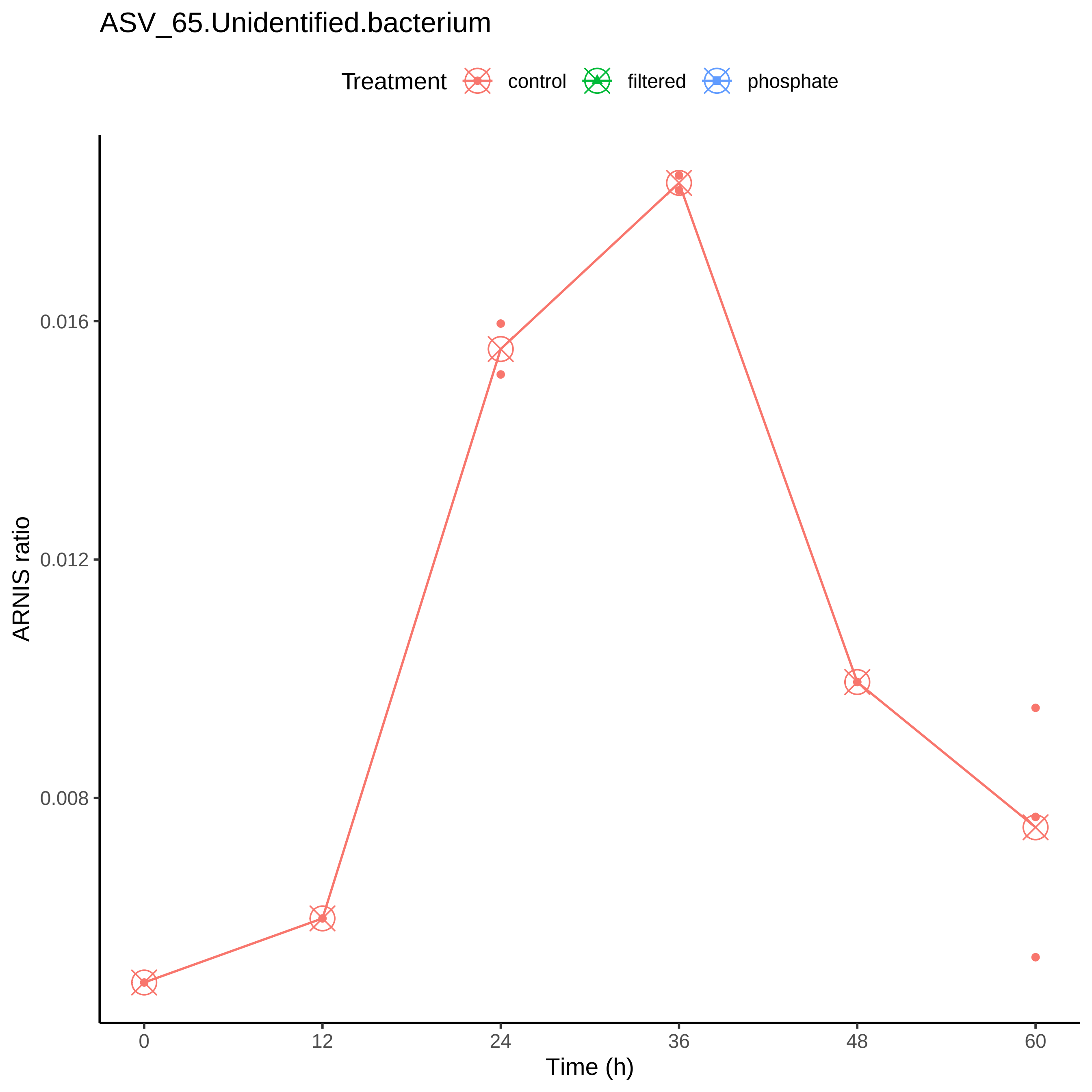

ASV_65.Unidentified.bacterium
Treatment
control
filtered
phosphate
ARNIS ratio
0.016
0.012
0.008
0
12
24
36
48
60
Time (h)

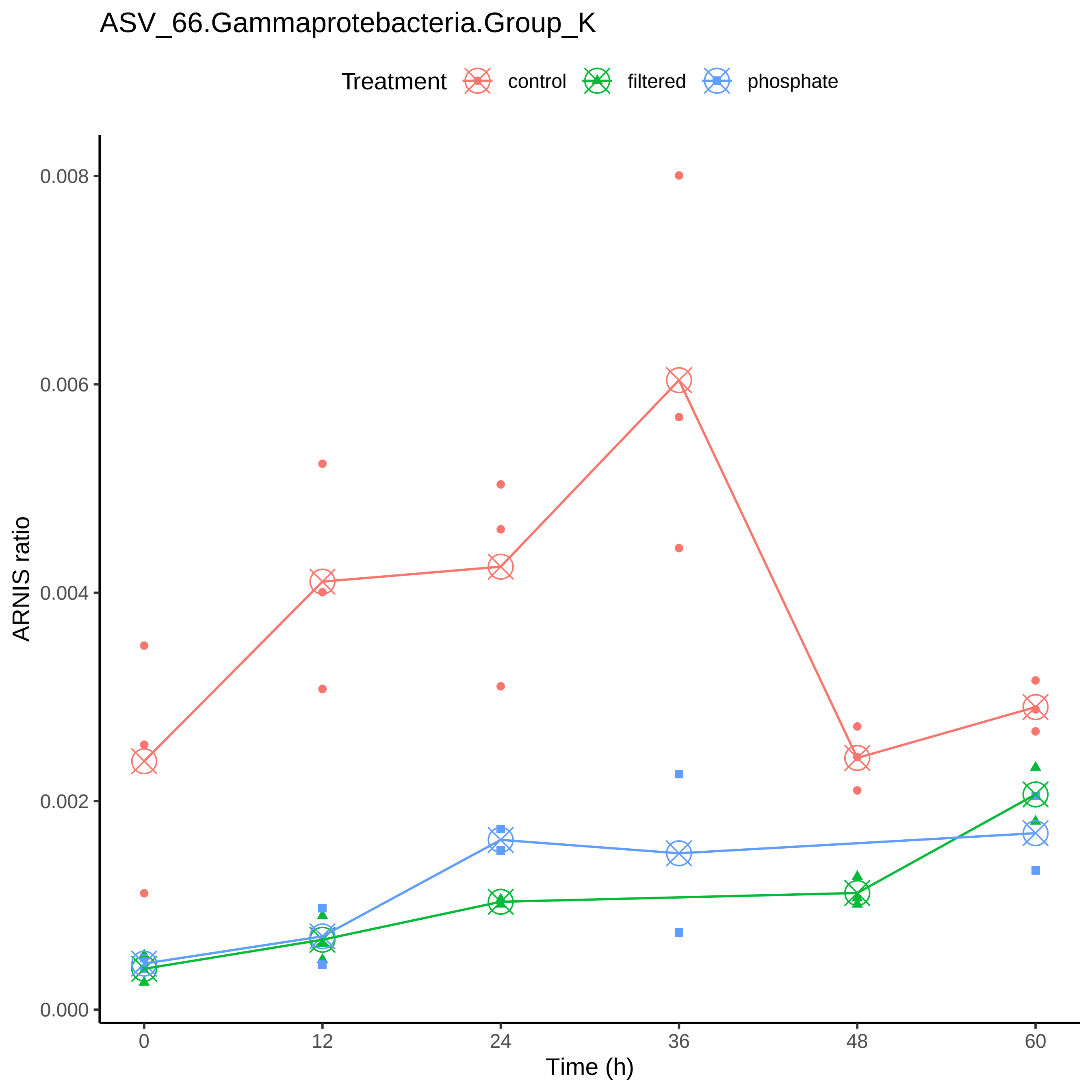
ASV_66.Gammaprotebacteria.Group_K
Treatment
control
filtered
phosphate
ARNIS ratio
0.000
0.002
0.004
0.006
0.008
Time (h)
0
12
24
36
48
60

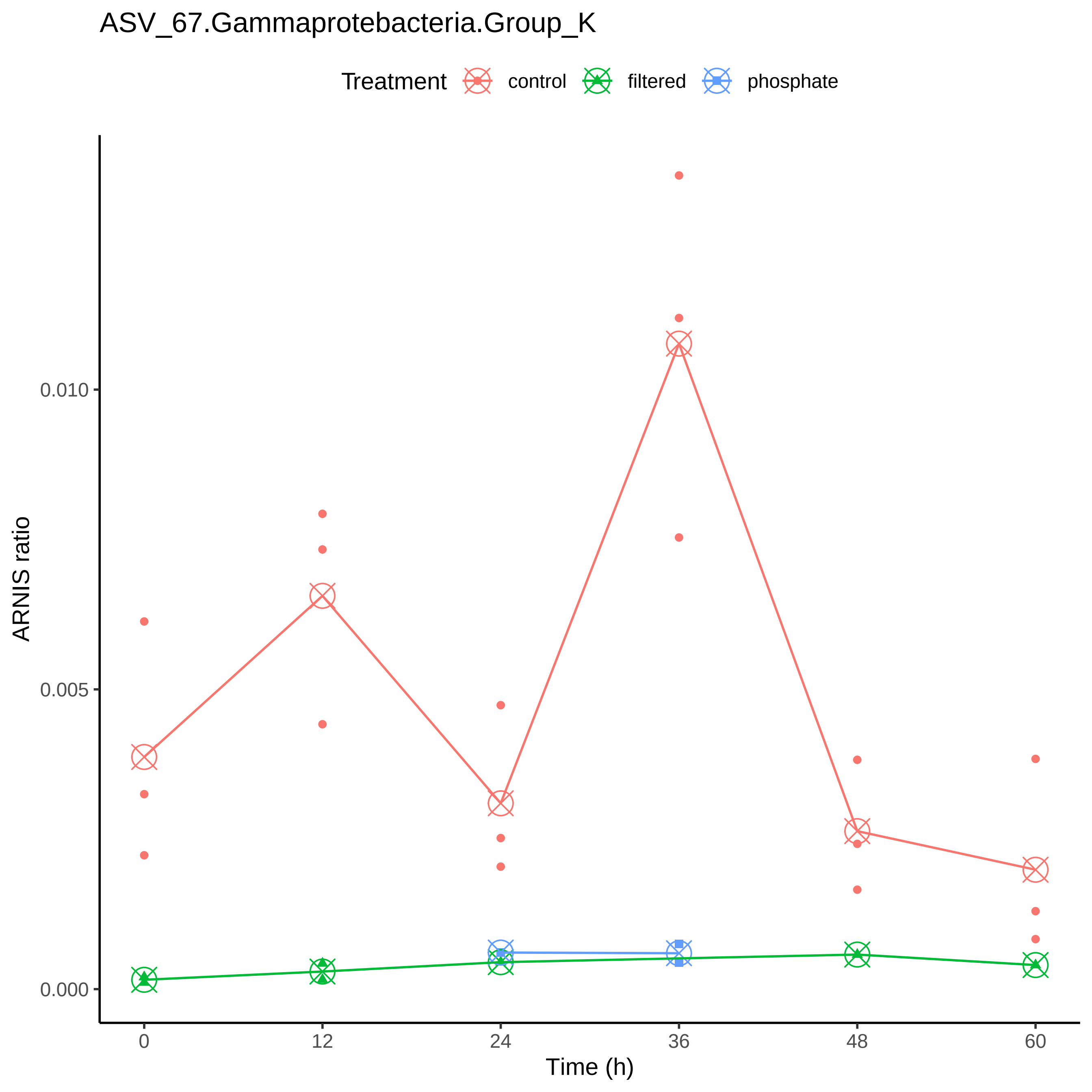

ASV_67.Gammaprotebacteria.Group_K
Treatment
control
filtered
phosphate
ARNIS ratio
0.010
0.005
0.000
0
12
24
36
48
60
Time (h)

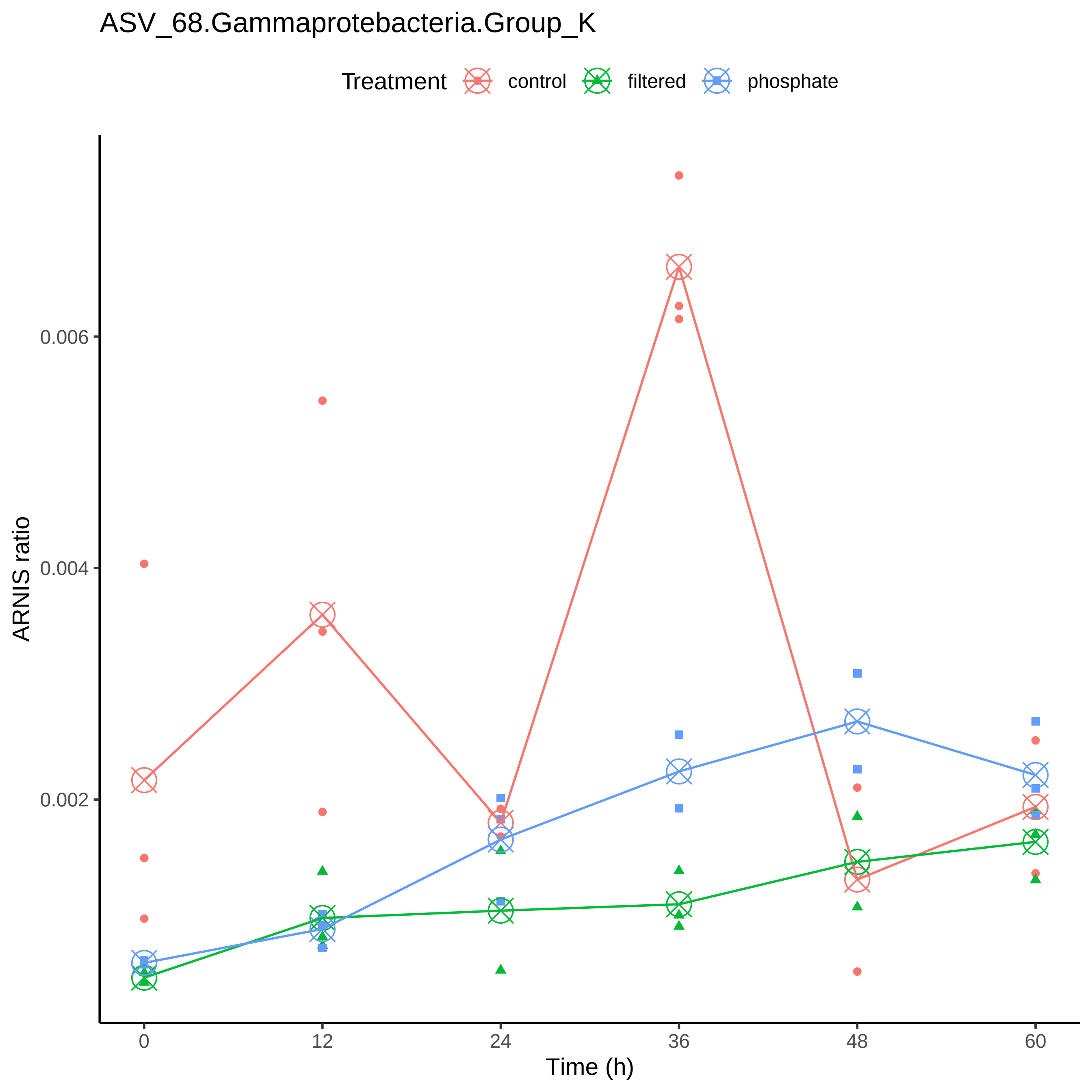

ASV_68.Gammaprotebacteria.Group_K
Treatment
control
filtered
phosphate
ARNIS ratio
0.002
0.004
0.006
Time (h)
0
12
24
36
48
60

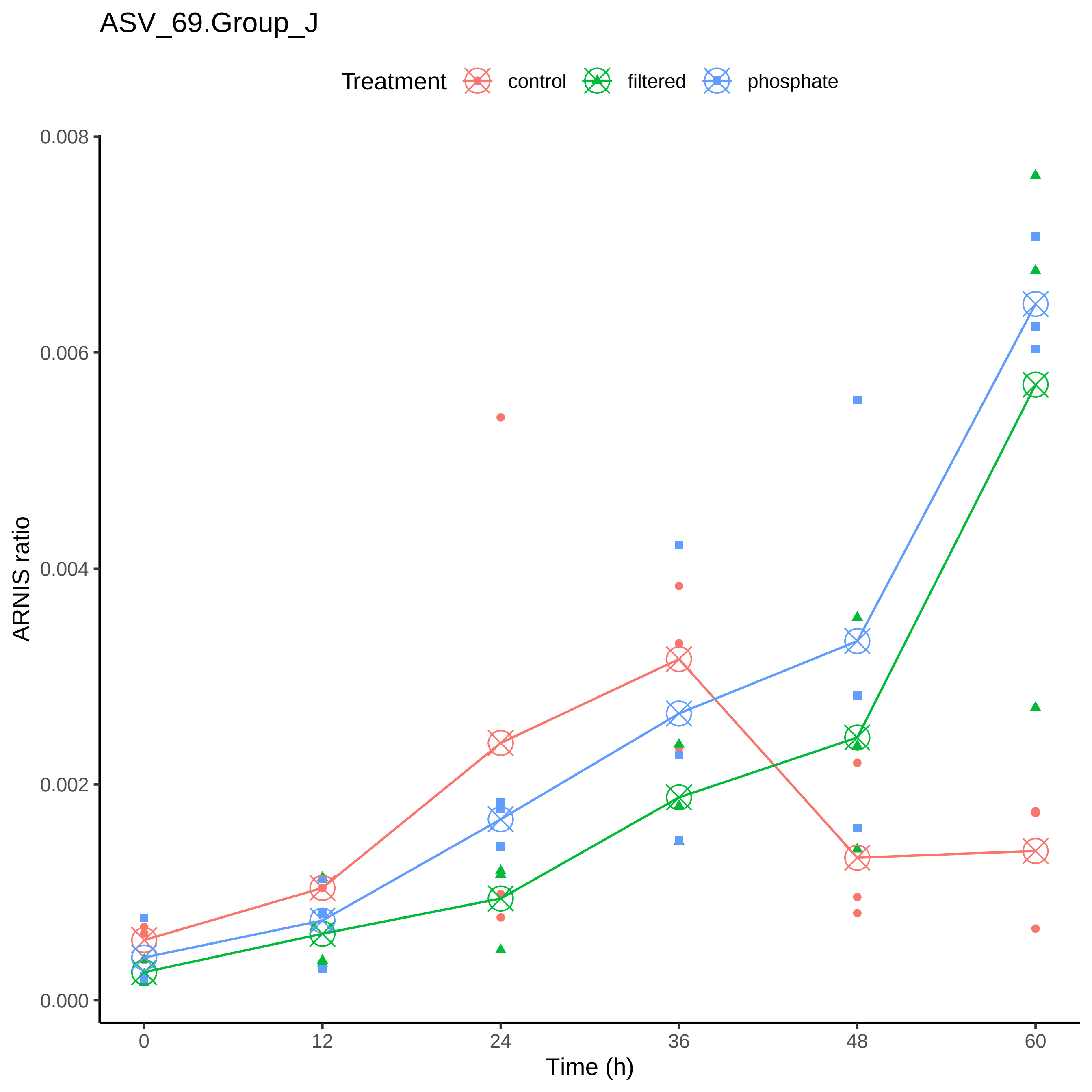

ASV_69.Group_J
Treatment
control
filtered
phosphate
0.008
0.006
0.004
0.002
0.000
ARNIS ratio
0
12
24
36
48
60
Time (h)

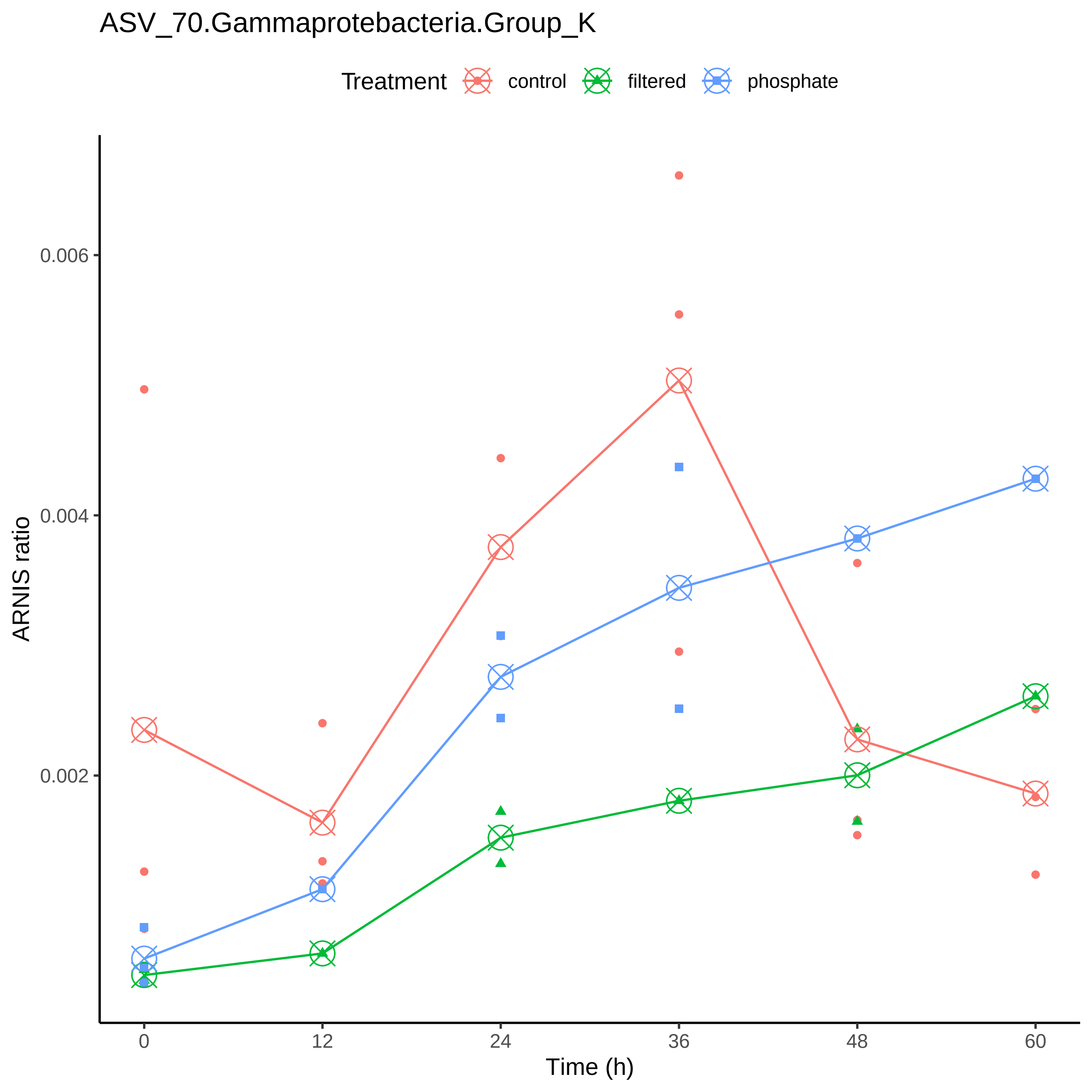
ASV_70.Gammaprotebacteria.Group_K
Treatment
control
filtered
phosphate
ARNIS ratio
0.006
0.004
0.002
0
12
24
36
48
60
Time (h)

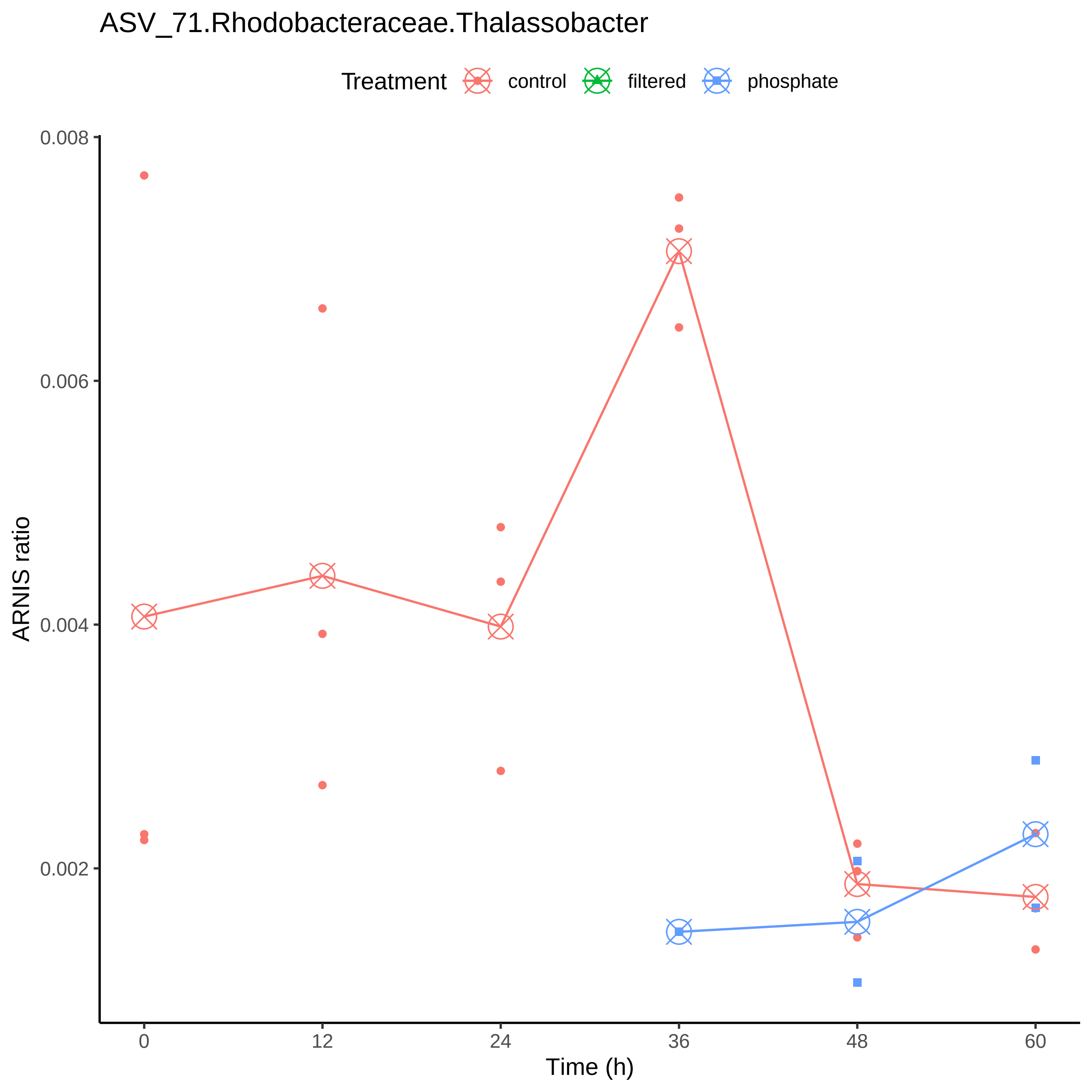

ASV_71.Rhodobacteraceae.Thalassobacter
Treatment
control
filtered
phosphate
0.008
0.006
0.004
0.002
ARNIS ratio
0
12
24
36
48
60
Time (h)

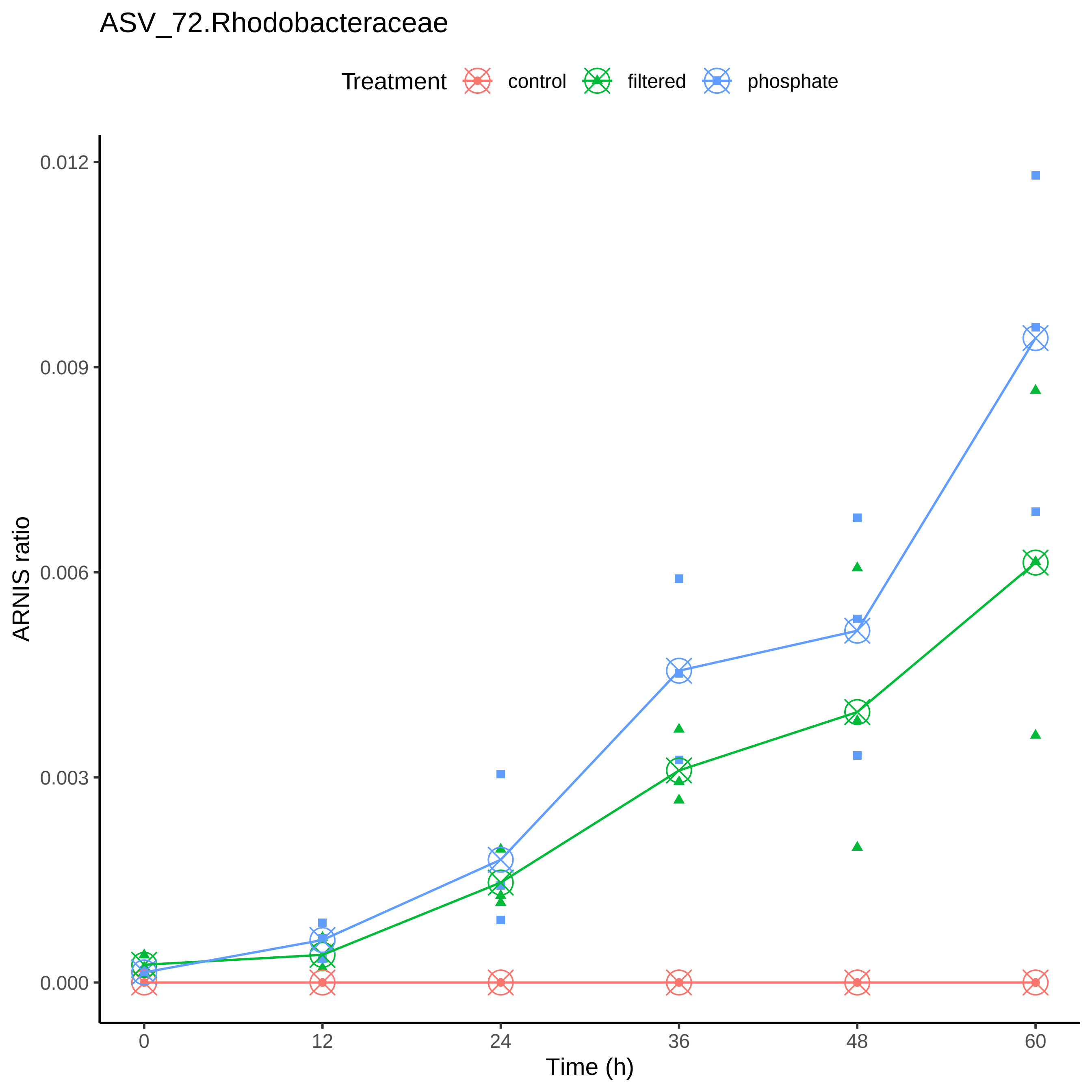
ASV_72.Rhodobacteraceae
Treatment
control
filtered
phosphate
ARNIS ratio
0.012
0.009
0.006
0.003
0.000
Time (h)
0
12
24
36
48
60

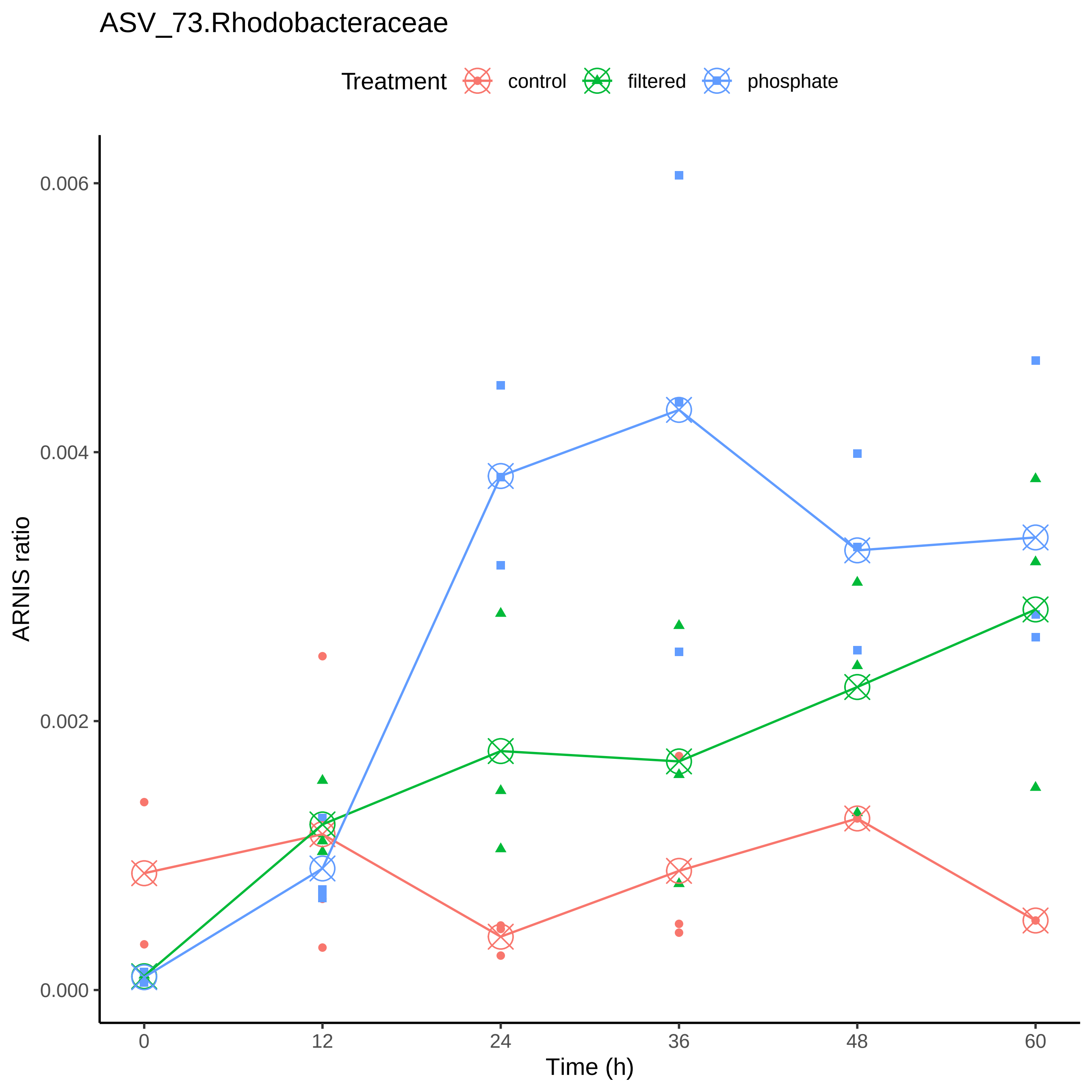
ASV_73.Rhodobacteraceae
Treatment
control
filtered
phosphate
ARNIS ratio
0.006
0.004
0.002
0.000
0
12
24
36
48
60
Time (h)

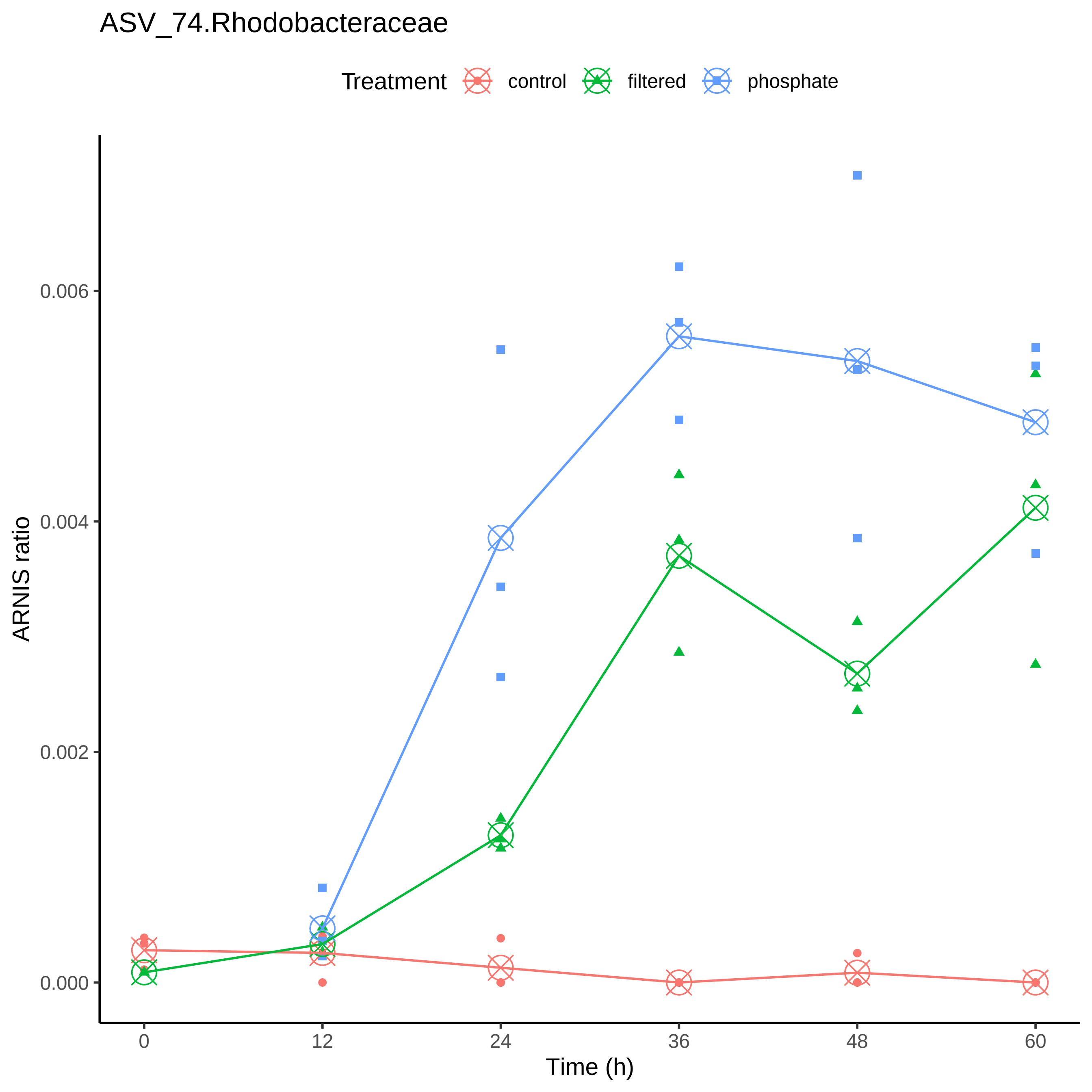

ASV_74.Rhodobacteraceae
Treatment
control
filtered
phosphate
ARNIS ratio
0.000
0.002
0.004
0.006
Time (h)
0
12
24
36
48
60

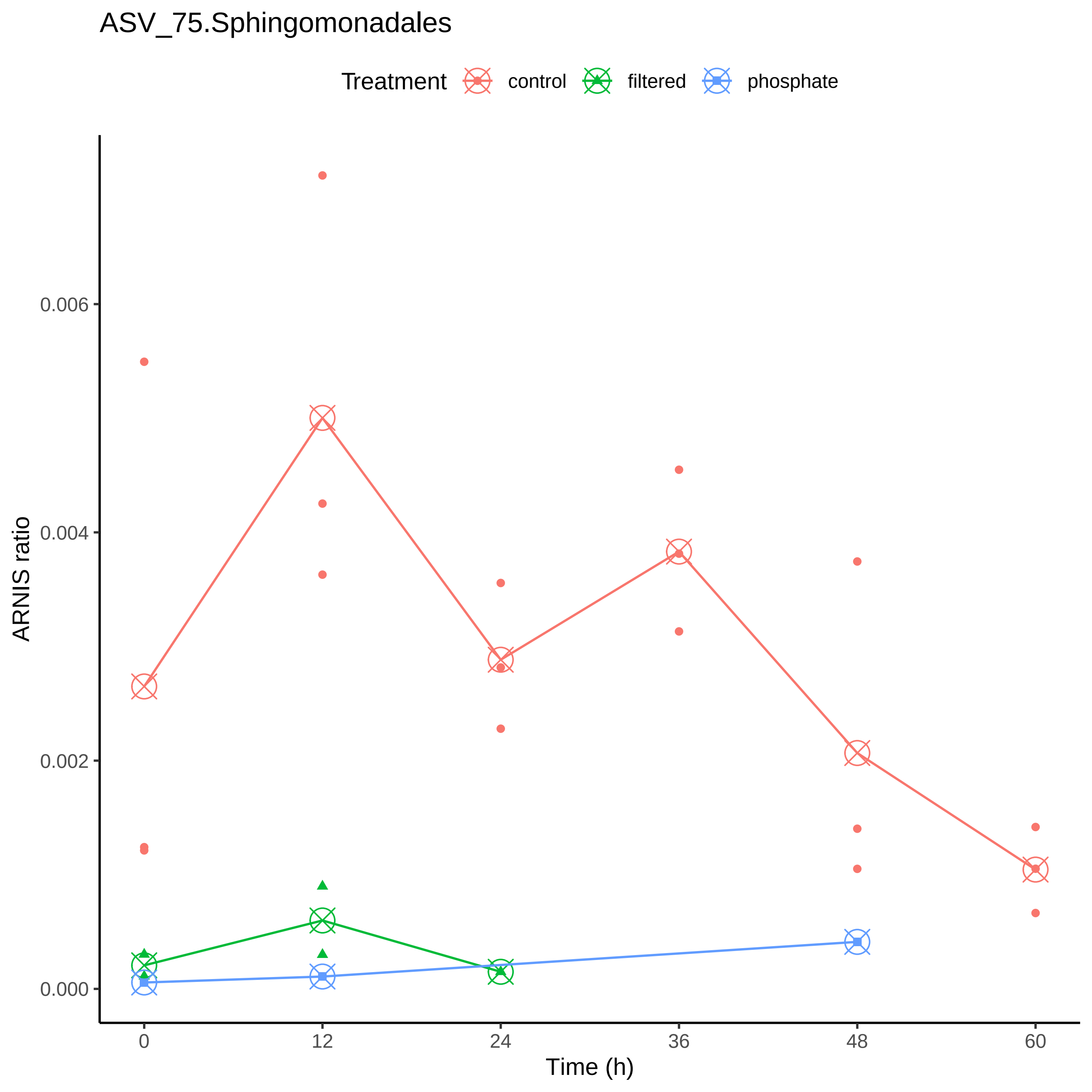

ASV_75.Sphingomonadales
Treatment
control
filtered
phosphate
ARNIS ratio
0.000
0.002
0.004
0.006
0
12
24
36
48
60
Time (h)

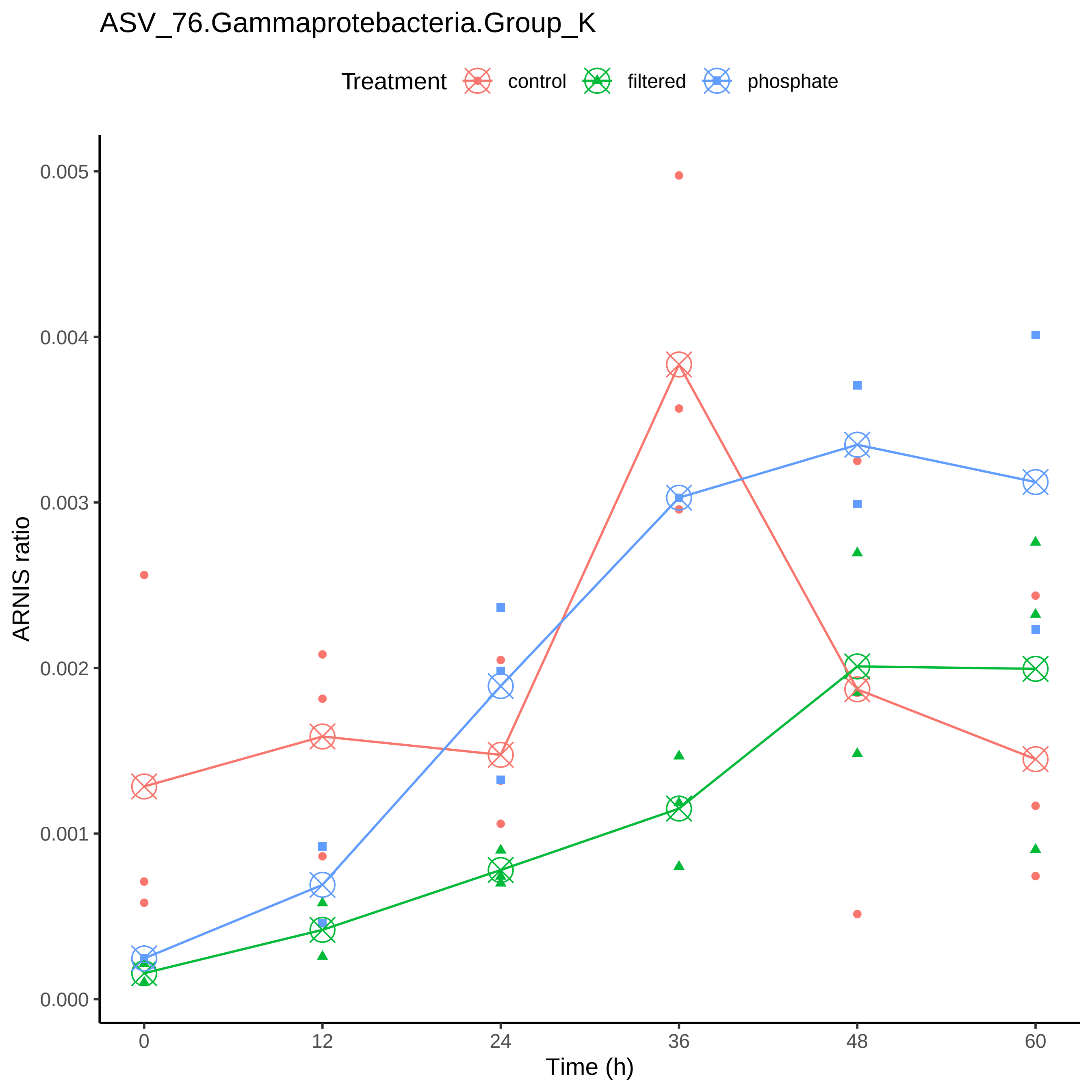
ASV_76.Gammaprotebacteria.Group_K
Treatment
control
filtered
phosphate
ARNIS ratio
0.000
0.001
0.002
0.003
0.004
0.005
0
12
24
36
48
60
Time (h)

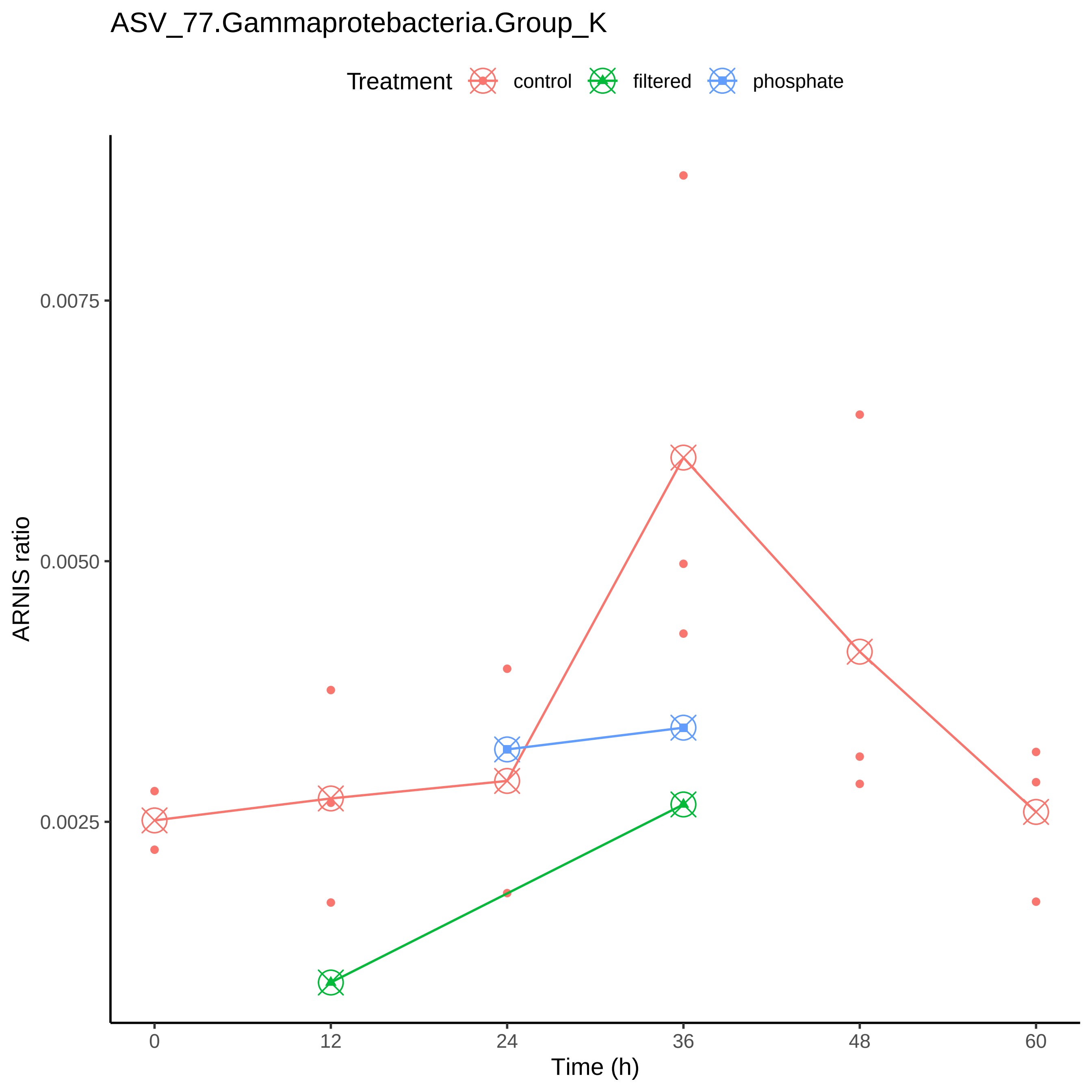

ASV_77.Gammaprotebacteria.Group_K
Treatment
control
filtered
phosphate
ARNIS ratio
0.0075
0.0050
0.0025
0
12
24
36
48
60
Time (h)

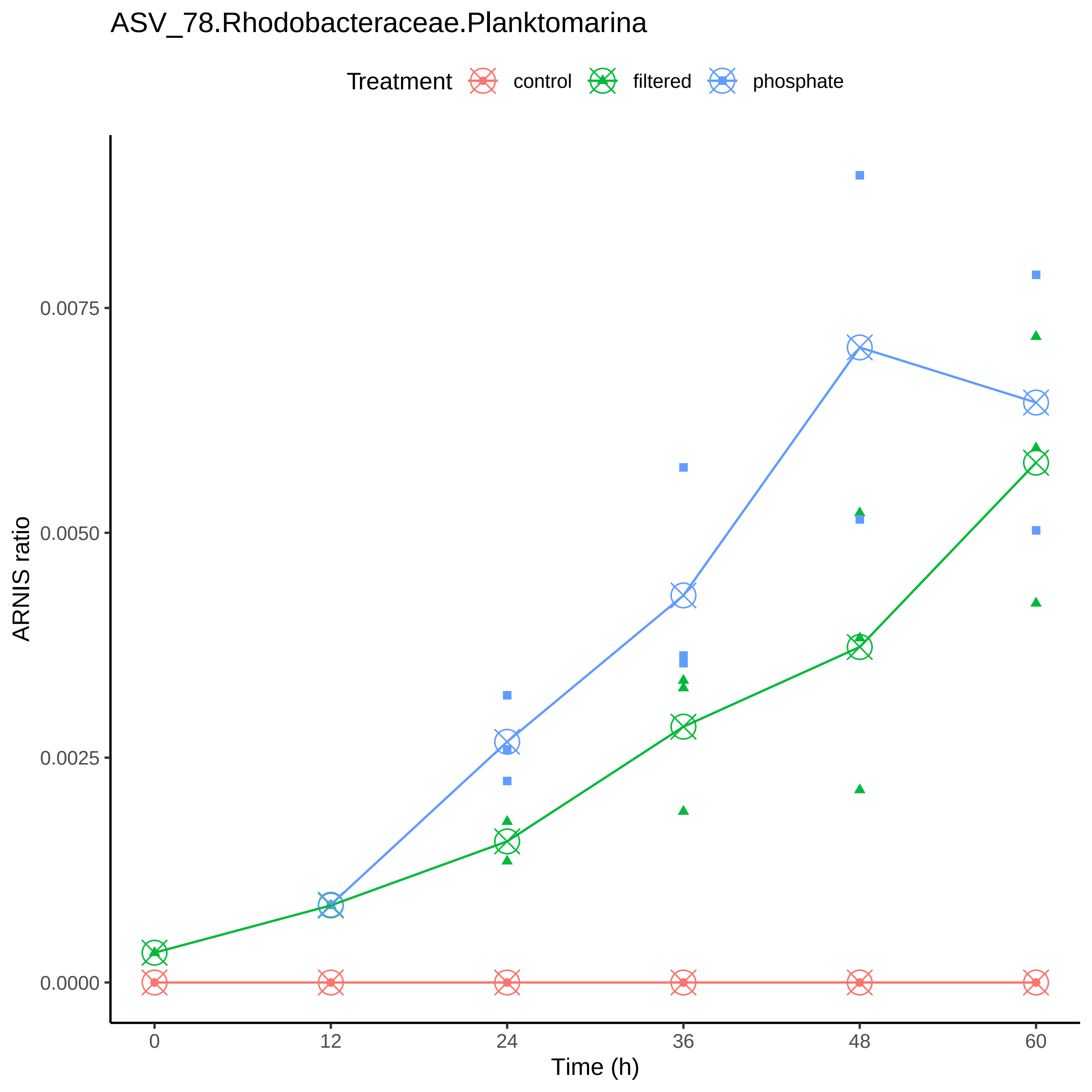

ASV_78.Rhodobacteraceae.Planktomarina
Treatment
control
filtered
phosphate
ARNIS ratio
0.0000
0.0025
0.0050
0.0075
Time (h)
0
12
24
36
48
60

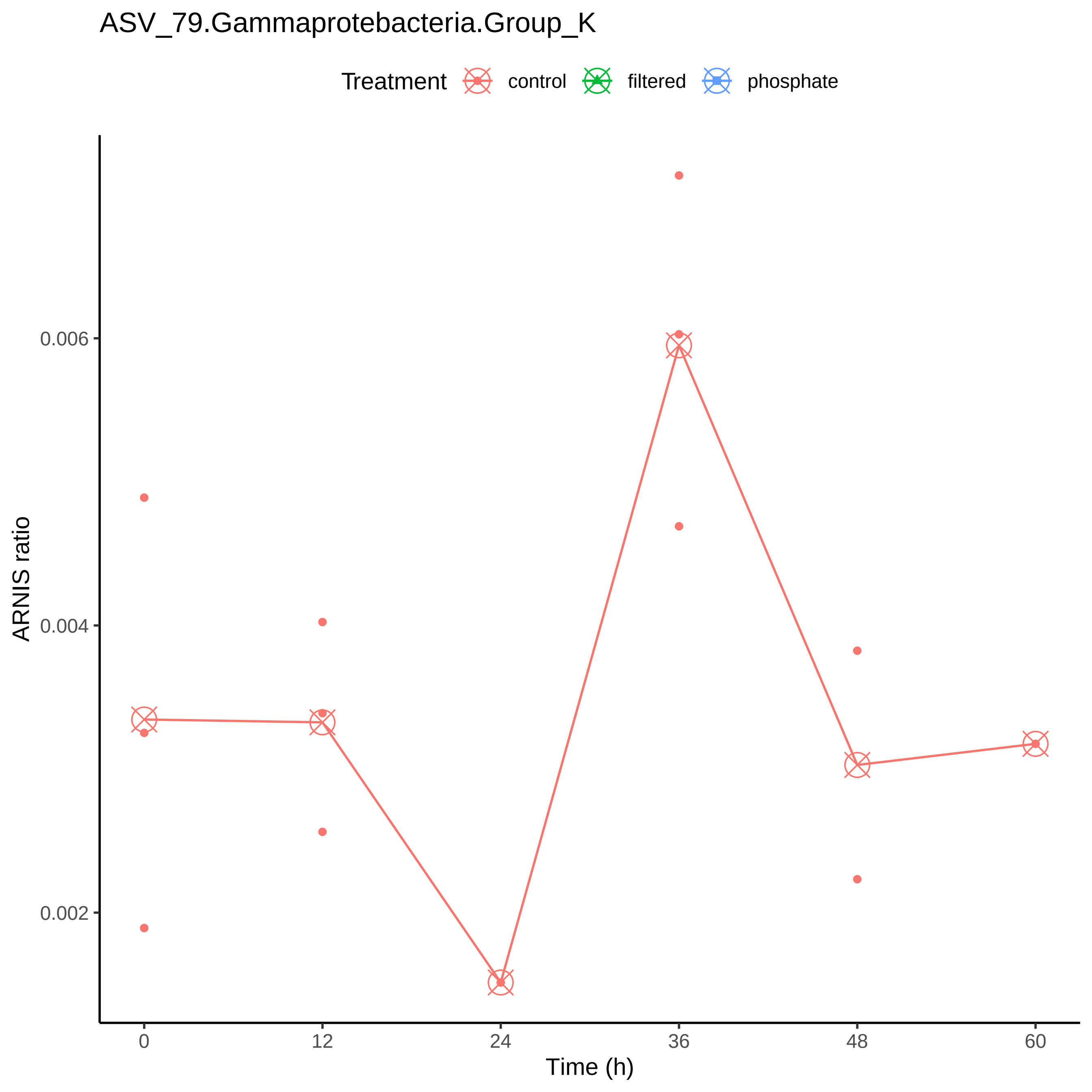

ASV_79.Gammaprotebacteria.Group_K
Treatment
control
filtered
phosphate
ARNIS ratio
0.006
0.004
0.002
0
12
24
36
48
60
Time (h)

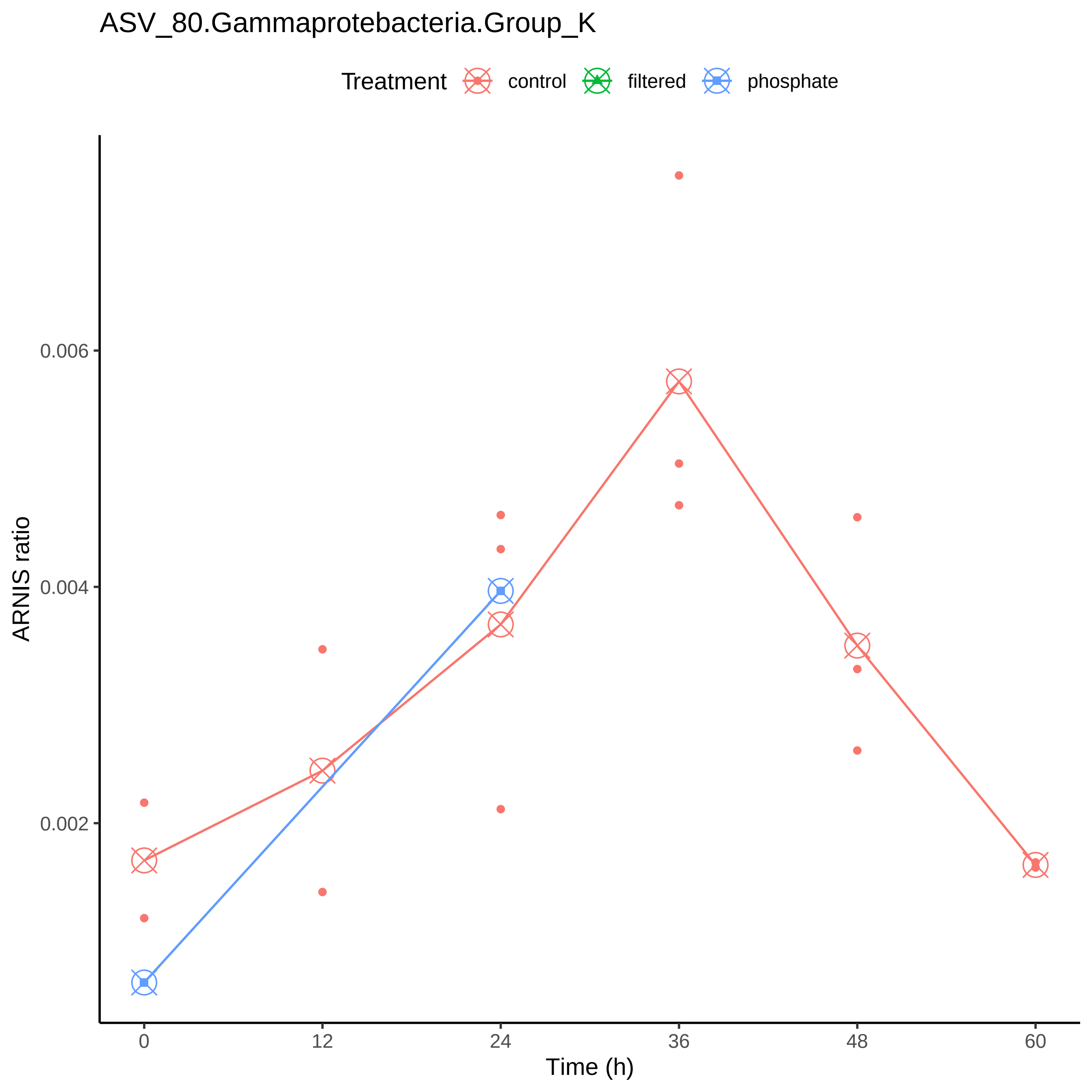
ASV_80.Gammaprotebacteria.Group_K
Treatment
control
filtered
phosphate
ARNIS ratio
0.006
0.004
0.002
0
12
24
36
48
60
Time (h)

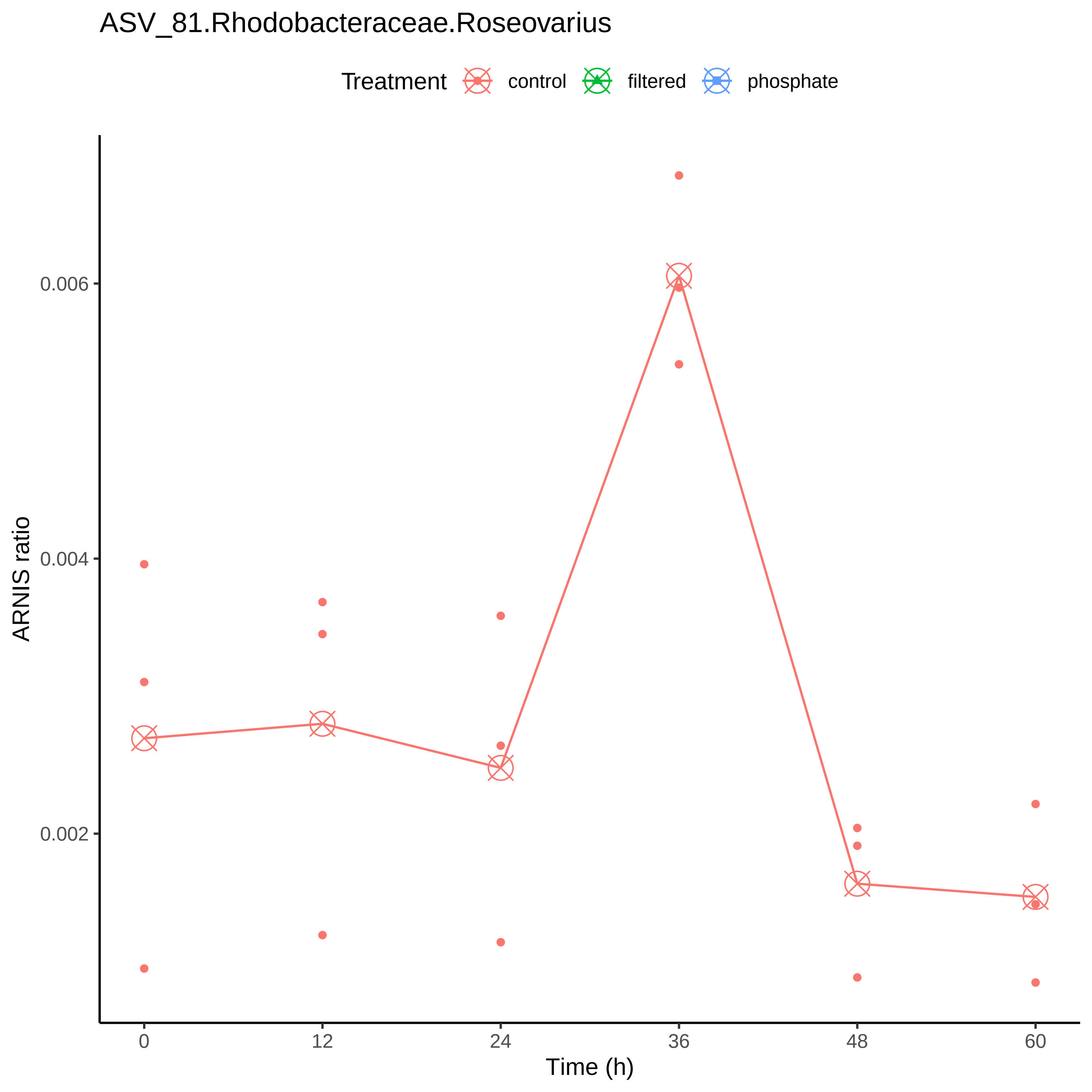
ASV_81.Rhodobacteraceae.Roseovarius
Treatment
control
filtered
phosphate
ARNIS ratio
0.006
0.004
0.002
0
12
24
36
48
60
Time (h)

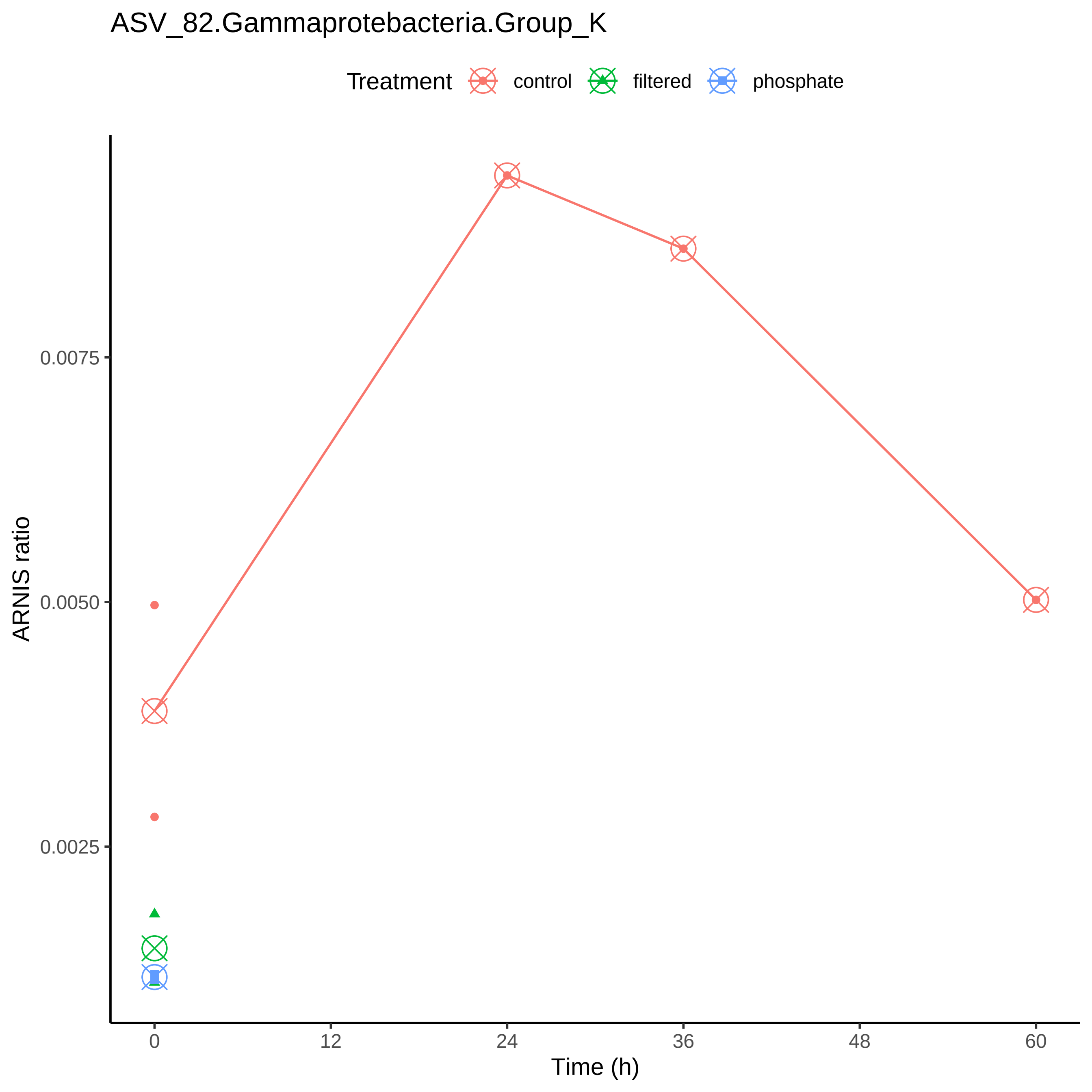

ASV_82.Gammaprotebacteria.Group_K
Treatment
control
filtered
phosphate
ARNIS ratio
0.0075
0.0050
0.0025
0
12
24
36
48
60
Time (h)

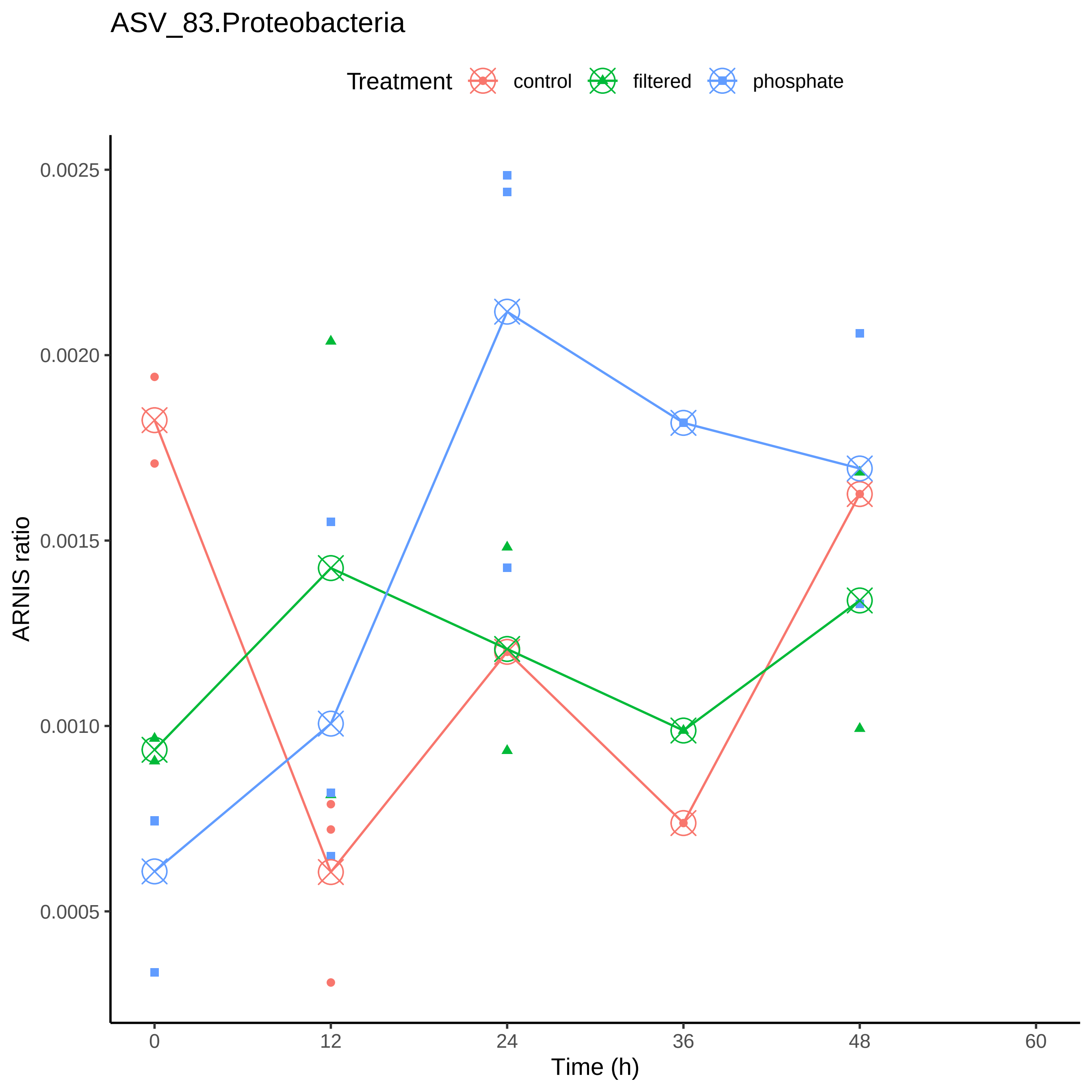
ASV_83.Proteobacteria
Treatment
control
filtered
phosphate
ARNIS ratio
0.0025
0.0020
0.0015
0.0010
0.0005
0
12
24
36
48
60
Time (h)

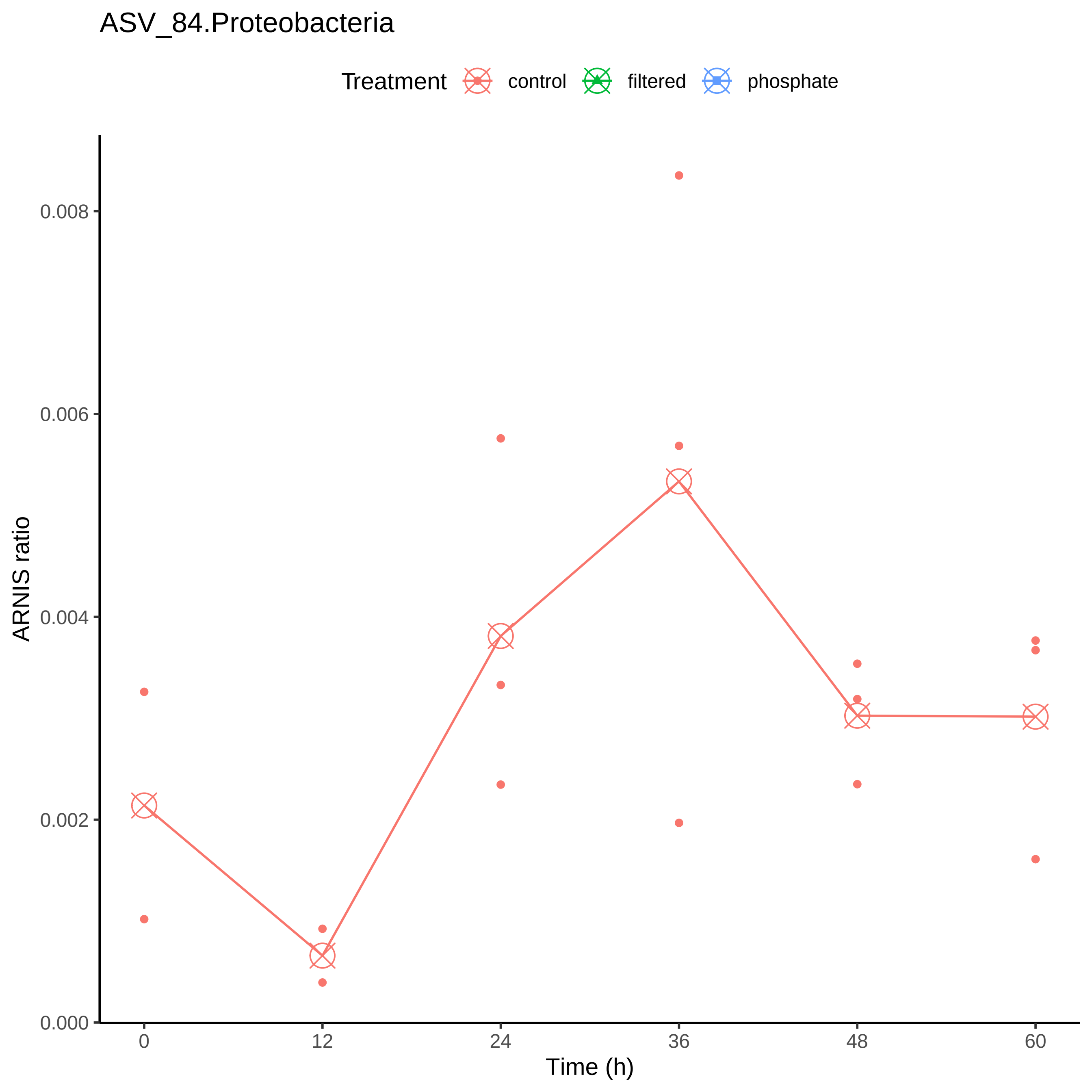
ASV_84.Proteobacteria
Treatment
control
filtered
phosphate
ARNIS ratio
0.008
0.006
0.004
0.002
0.000
0
12
24
36
48
60
Time (h)

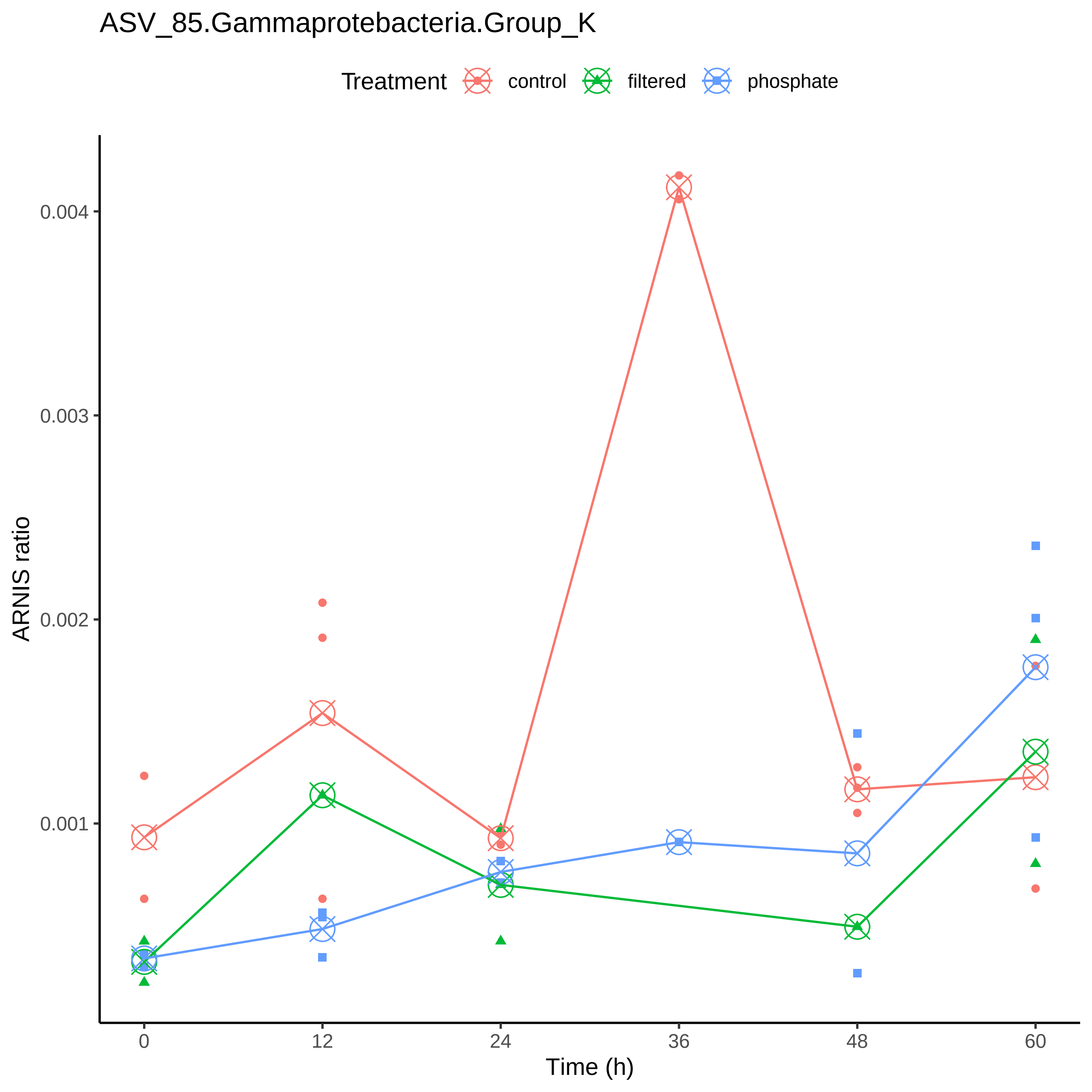
ASV_85.Gammaprotebacteria.Group_K
Treatment
control
filtered
phosphate
ARNIS ratio
0.004
0.003
0.002
0.001
0
12
24
36
48
60
Time (h)

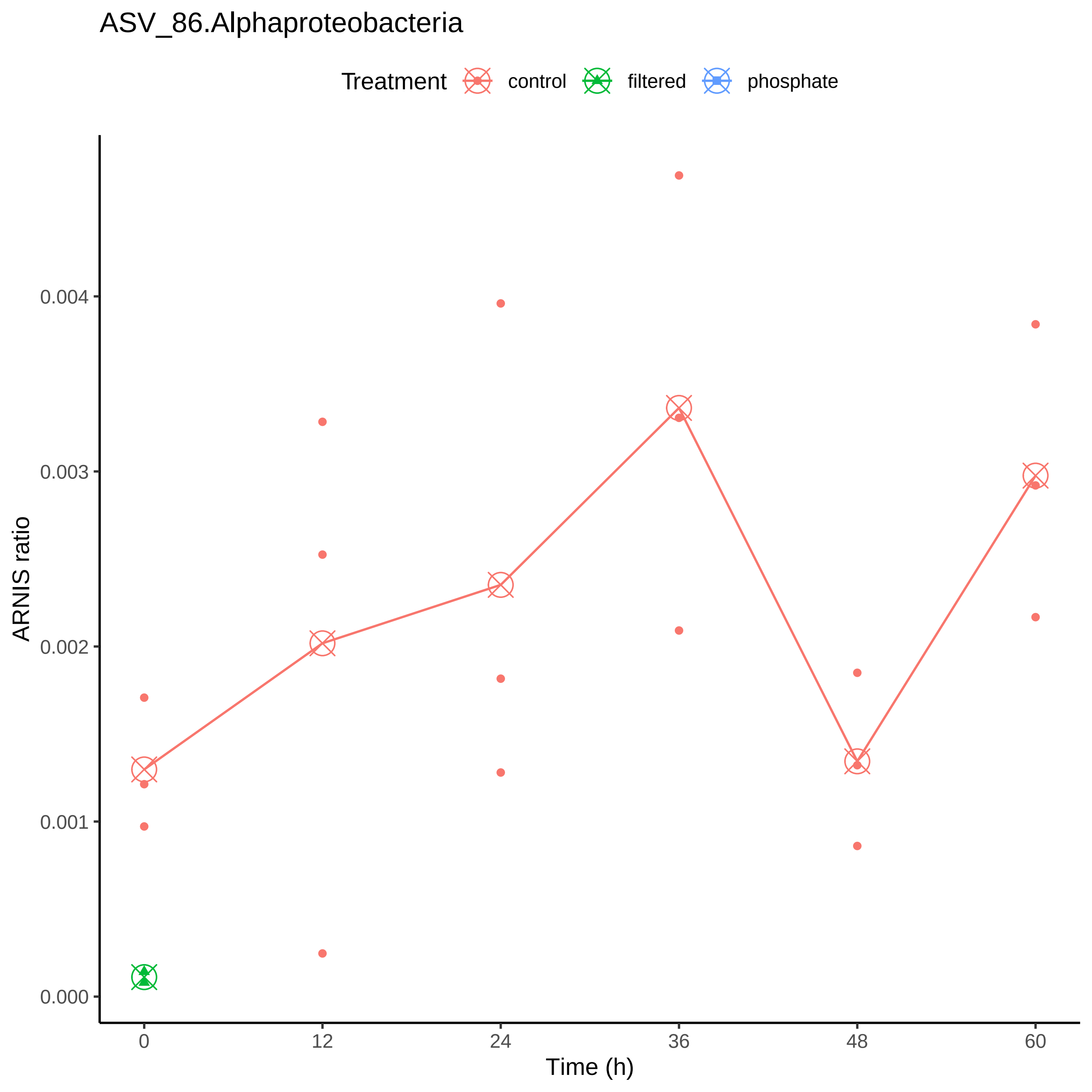
ASV_86.Alphaproteobacteria
Treatment
control
filtered
phosphate
ARNIS ratio
0.000
0.001
0.002
0.003
0.004
0
12
24
36
48
60
Time (h)

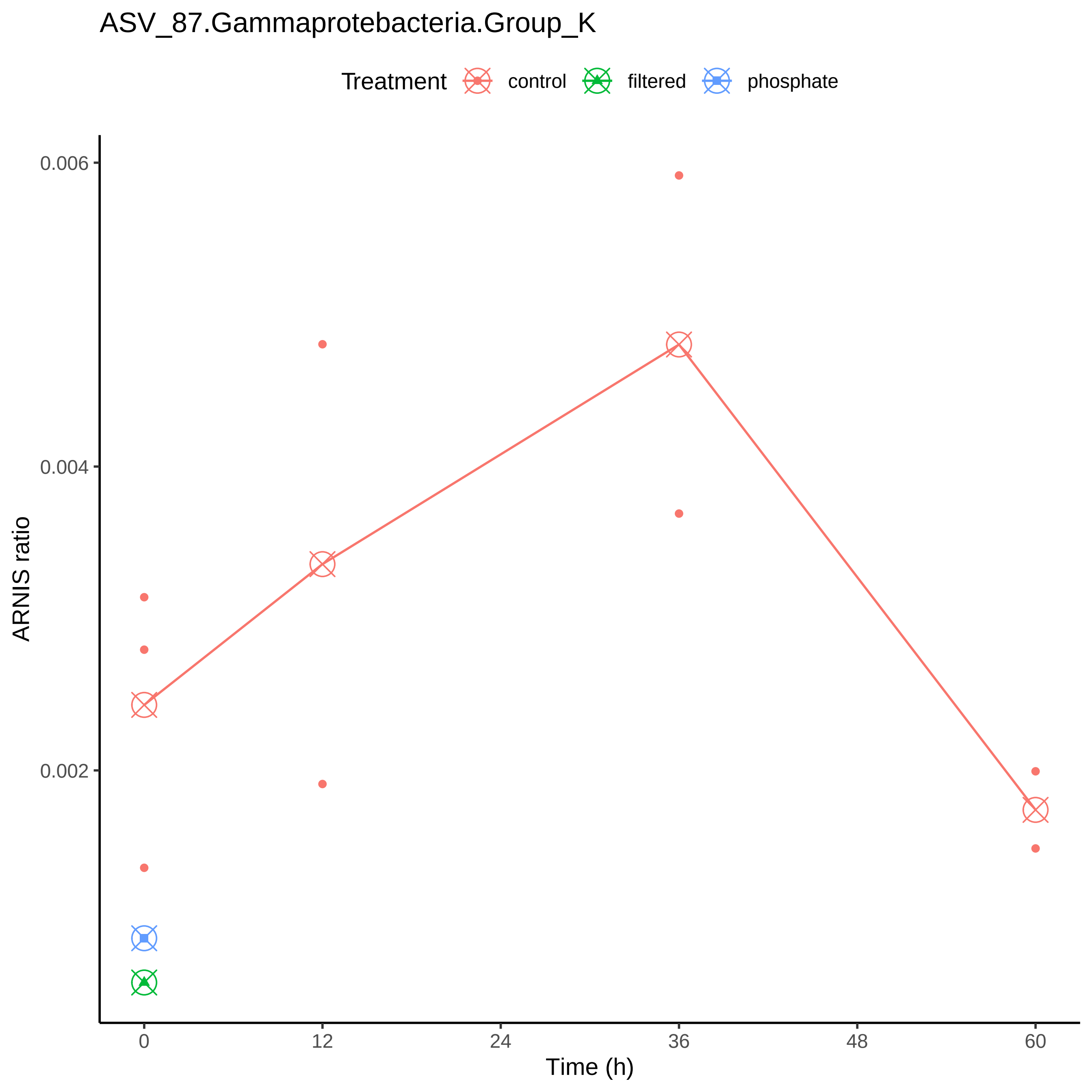
ASV_87.Gammaprotebacteria.Group_K
Treatment
control
filtered
phosphate
ARNIS ratio
0.006
0.004
0.002
Time (h)
0
12
24
36
48
60

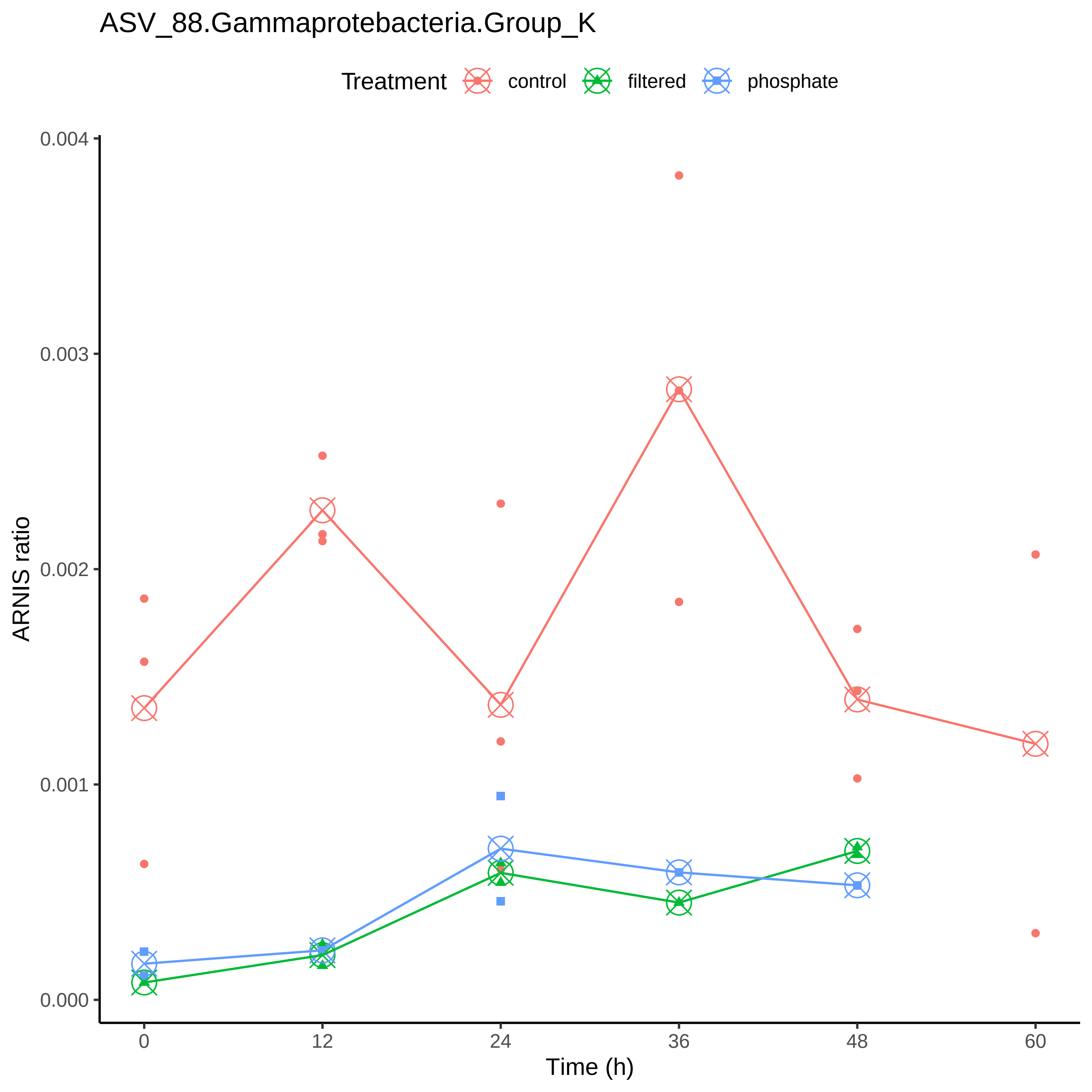

ASV_88.Gammaprotebacteria.Group_K
Treatment
control
filtered
phosphate
ARNIS ratio
0.004
0.003
0.002
0.001
0.000
Time (h)
0
12
24
36
48
60

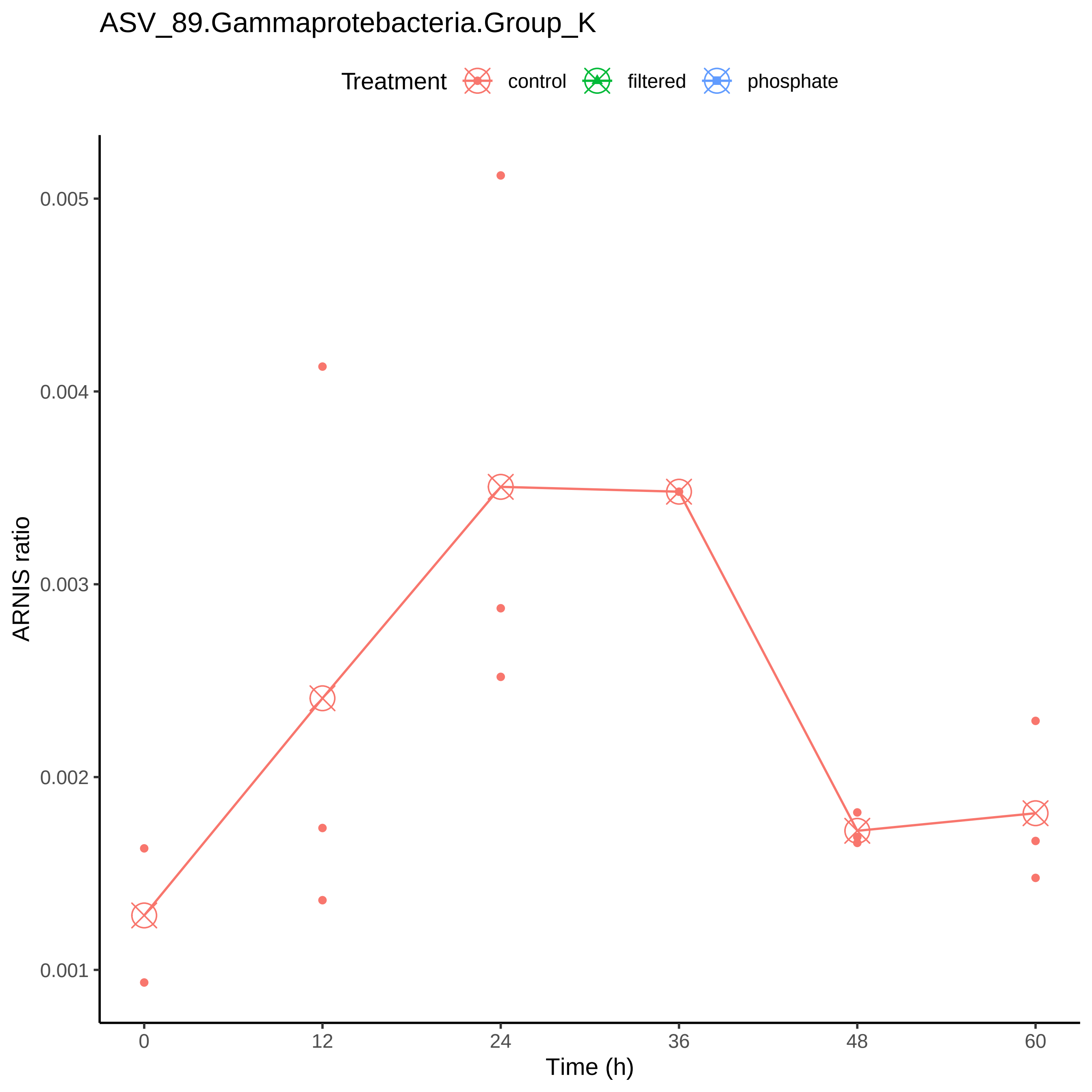

ASV_89.Gammaprotebacteria.Group_K
Treatment
control
filtered
phosphate
ARNIS ratio
0.005
0.004
0.003
0.002
0.001
Time (h)
0
12
24
36
48
60

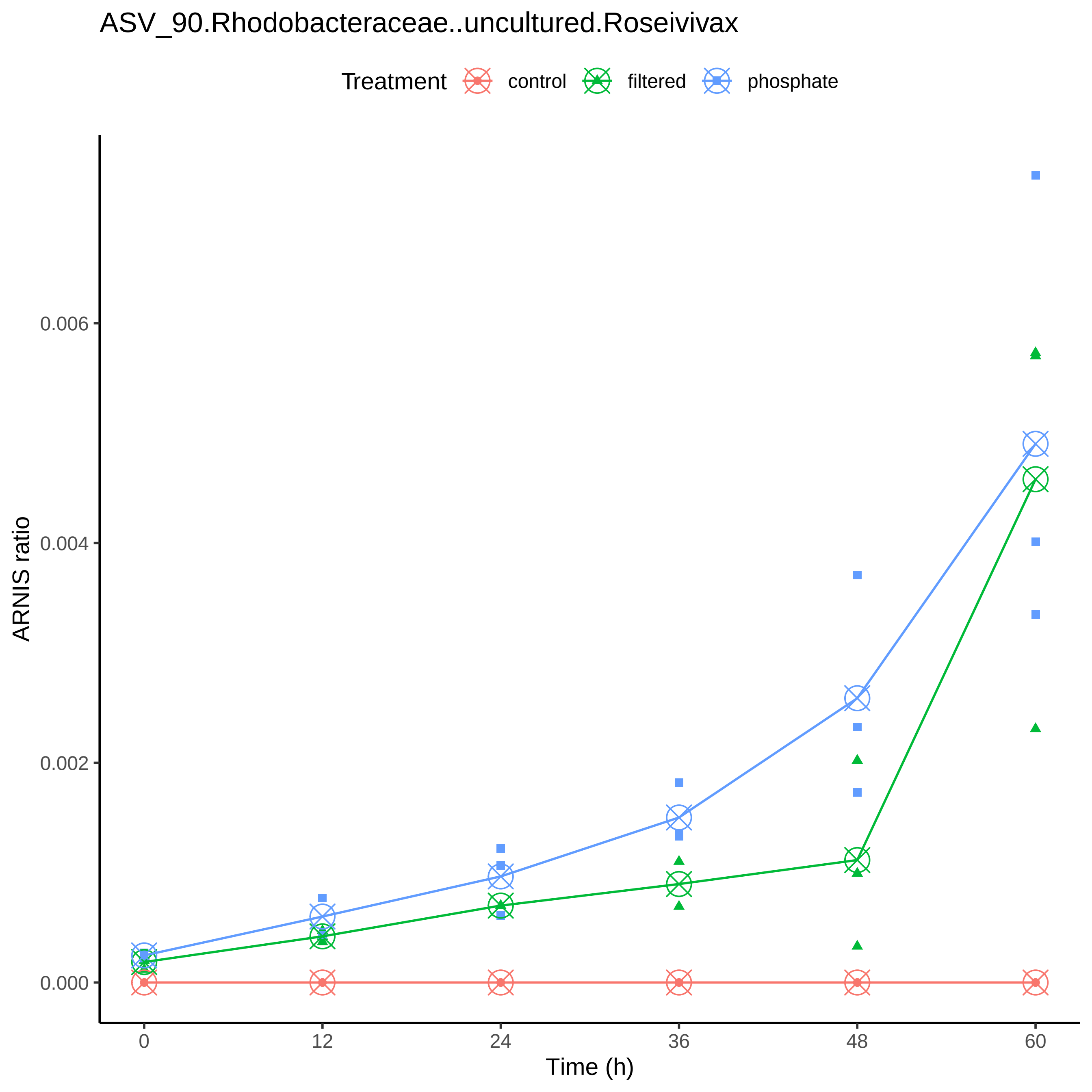

ASV_90.Rhodobacteraceae..uncultured.Roseivivax
Treatment
control
filtered
phosphate
0.006
0.004
0.002
0.000
ARNIS ratio
0
12
24
36
48
60
Time (h)

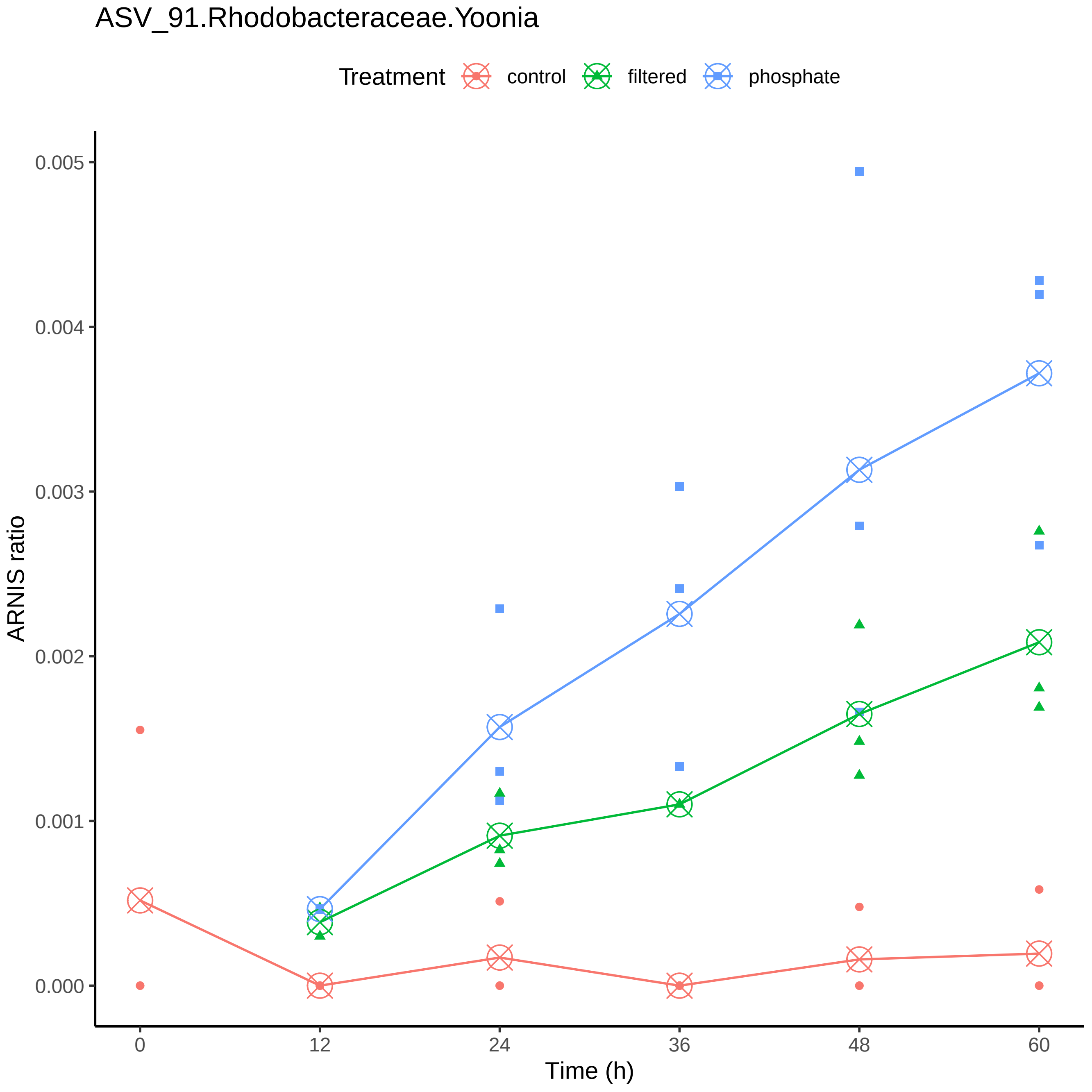

ASV_91.Rhodobacteraceae.Yoonia
Treatment
control
filtered
phosphate
ARNIS ratio
0.005
0.004
0.003
0.002
0.001
0.000
0
12
24
36
48
60
Time (h)

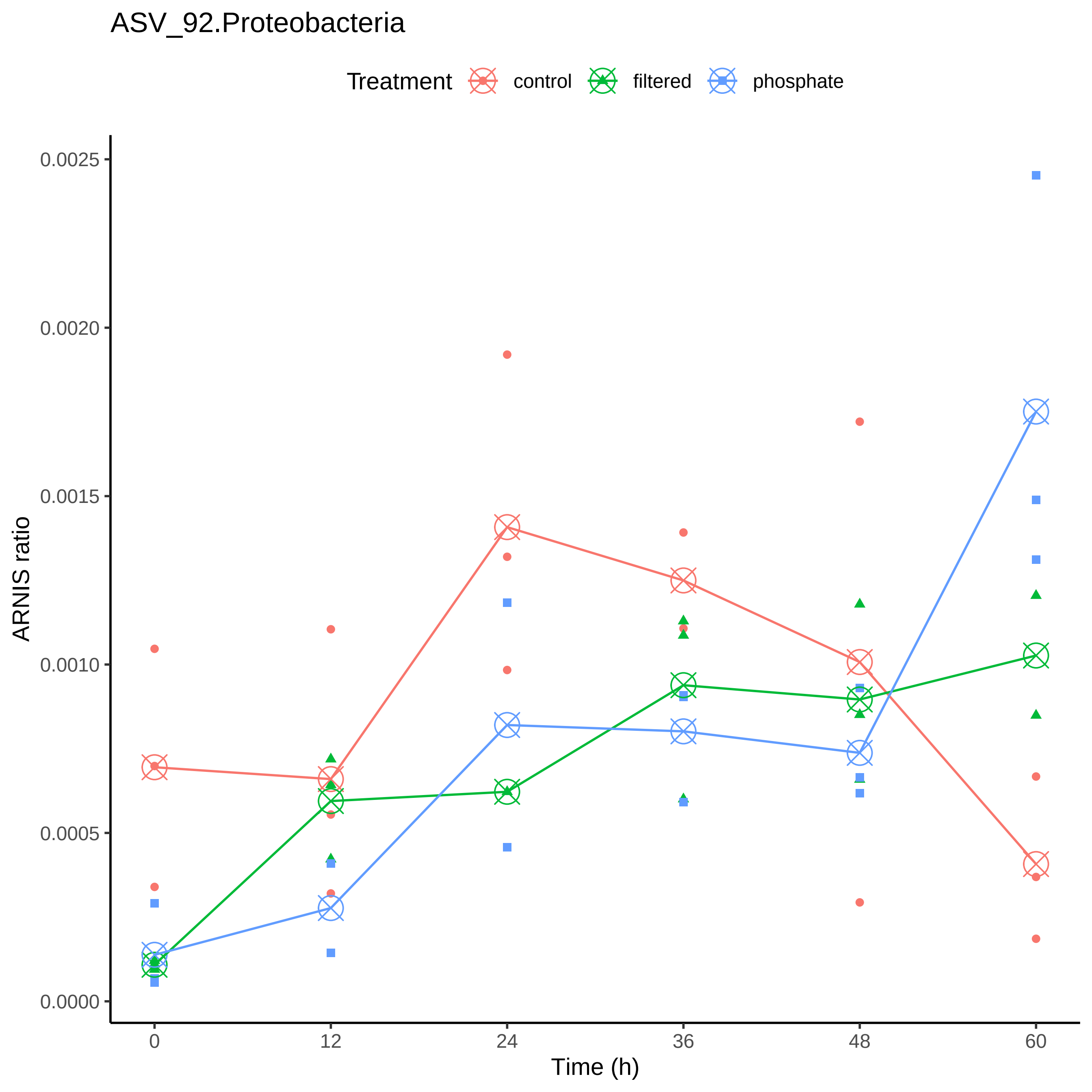
ASV_92.Proteobacteria
Treatment
control
filtered
phosphate
ARNIS ratio
0.0025
0.0020
0.0015
0.0010
0.0005
0.0000
Time (h)
0
12
24
36
48
60

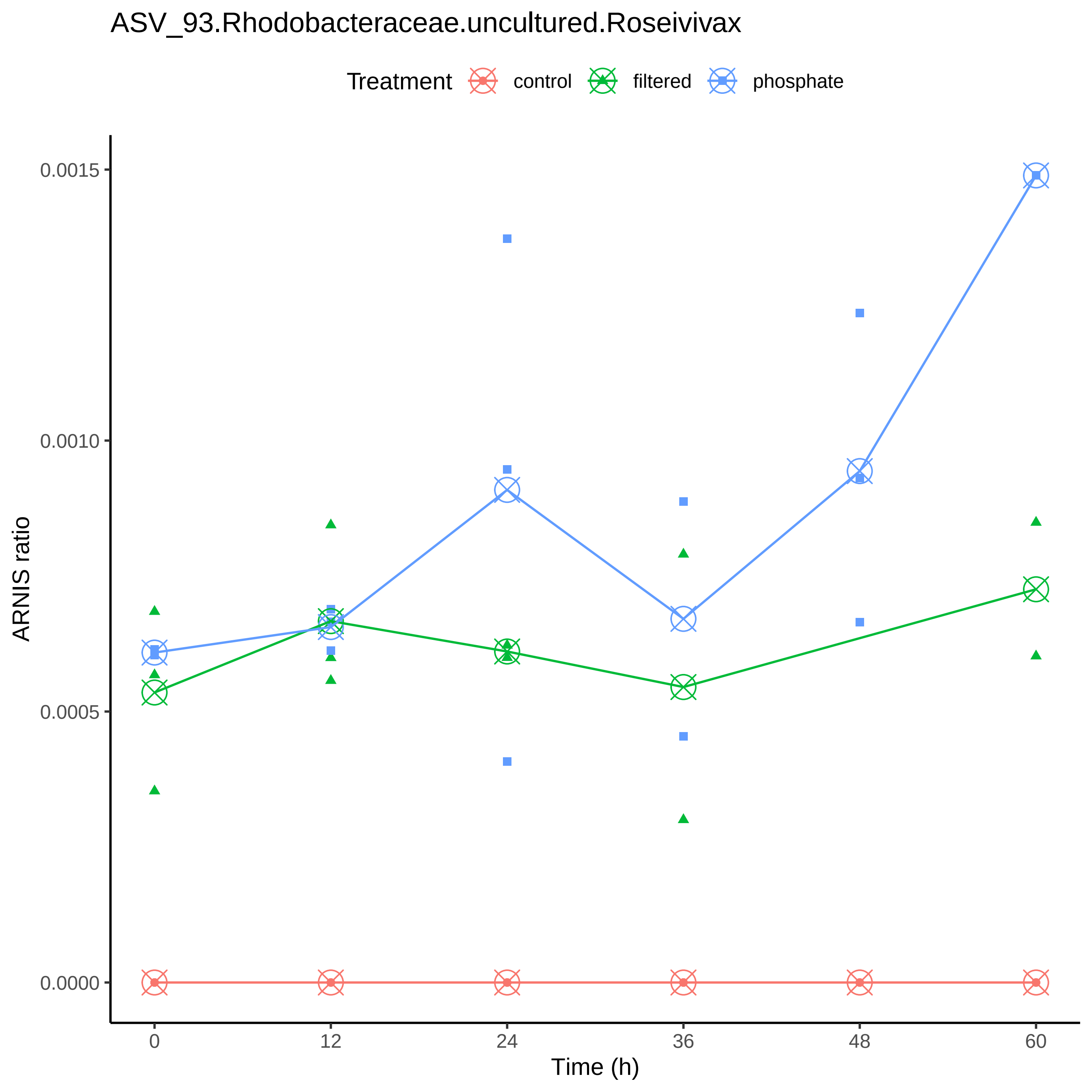

ASV_93.Rhodobacteraceae.uncultured.Roseivivax
Treatment
control
filtered
phosphate
ARNIS ratio
0.0015
0.0010
0.0005
0.0000
0
12
24
36
48
60
Time (h)

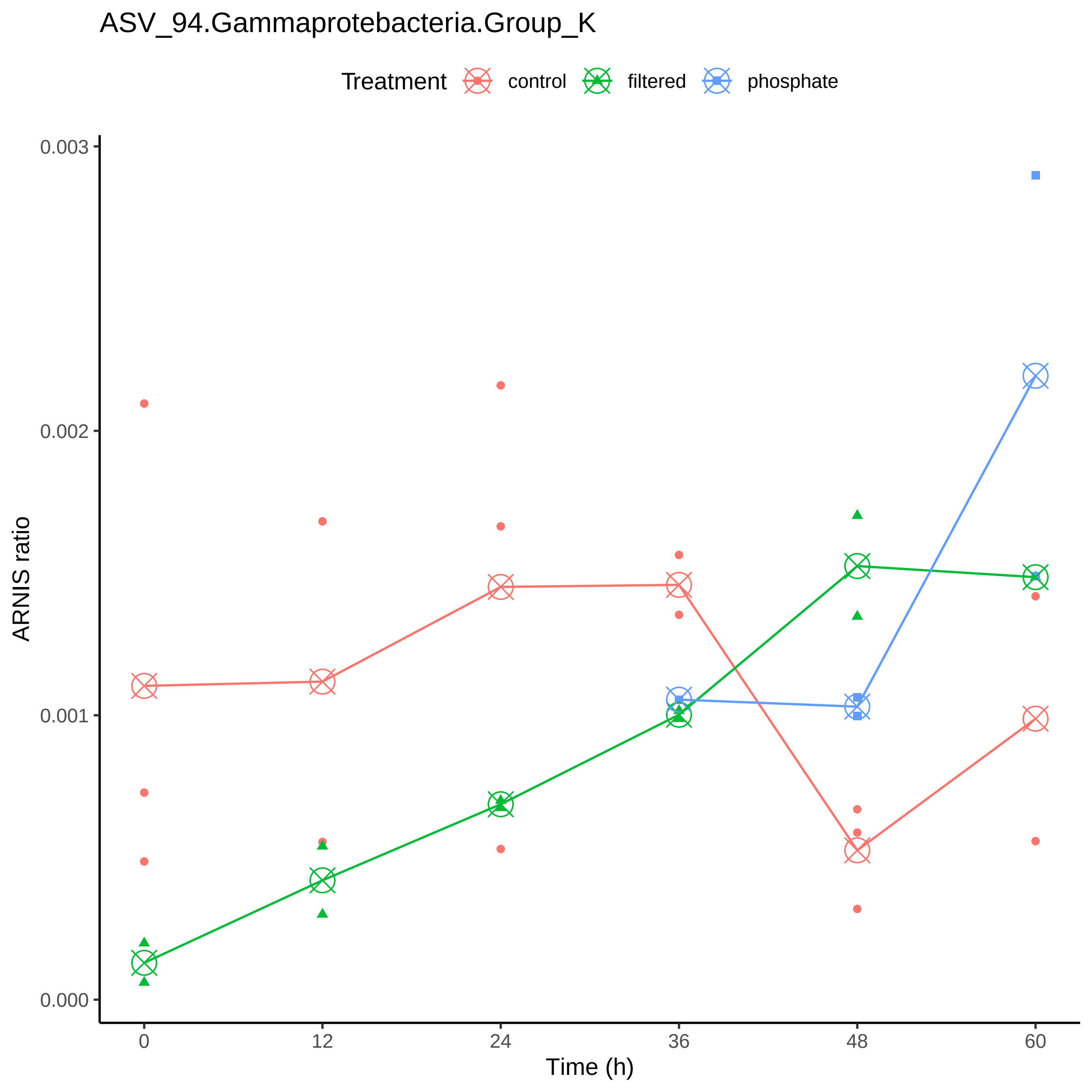
ASV_94.Gammaprotebacteria.Group_K
Treatment
control
filtered
phosphate
ARNIS ratio
0.003
0.002
0.001
0.000
Time (h)
0
12
24
36
48
60

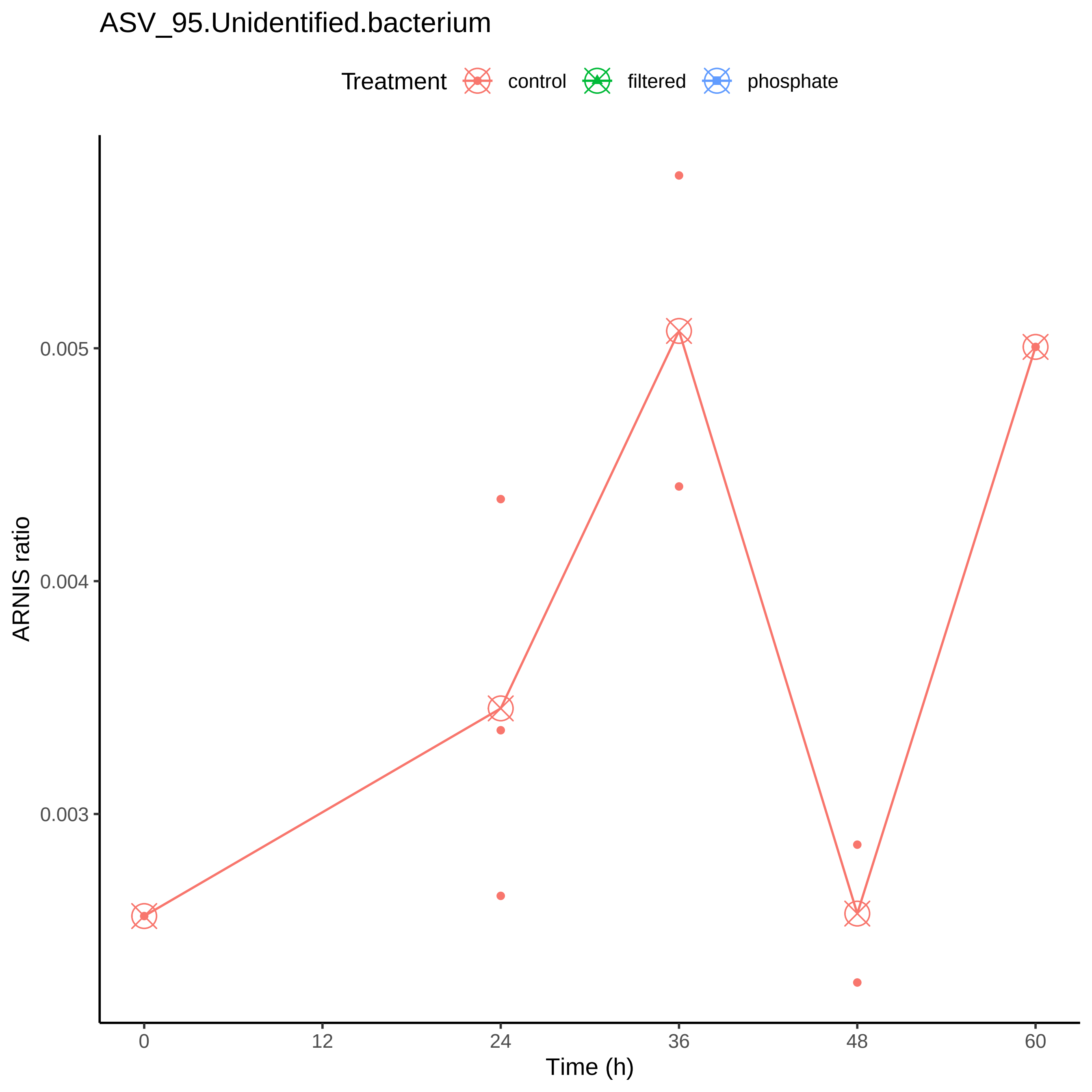

ASV_95.Unidentified.bacterium
Treatment
control
filtered
phosphate
ARNIS ratio
0.005
0.004
0.003
Time (h)
0
12
24
36
48
60

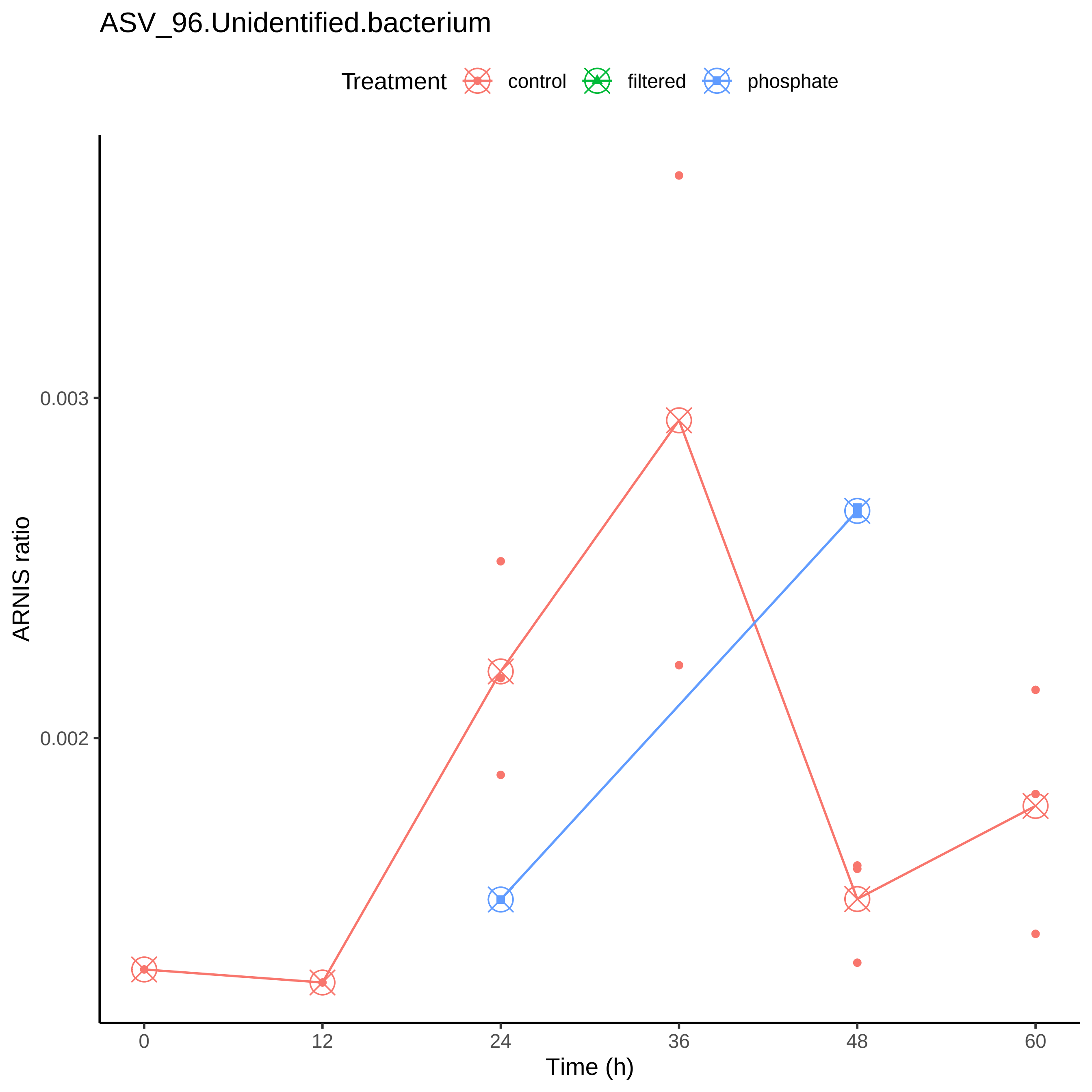
ASV_96.Unidentified.bacterium
Treatment
control
filtered
phosphate
ARNIS ratio
0.003
0.002
Time (h)
0
12
24
36
48
60

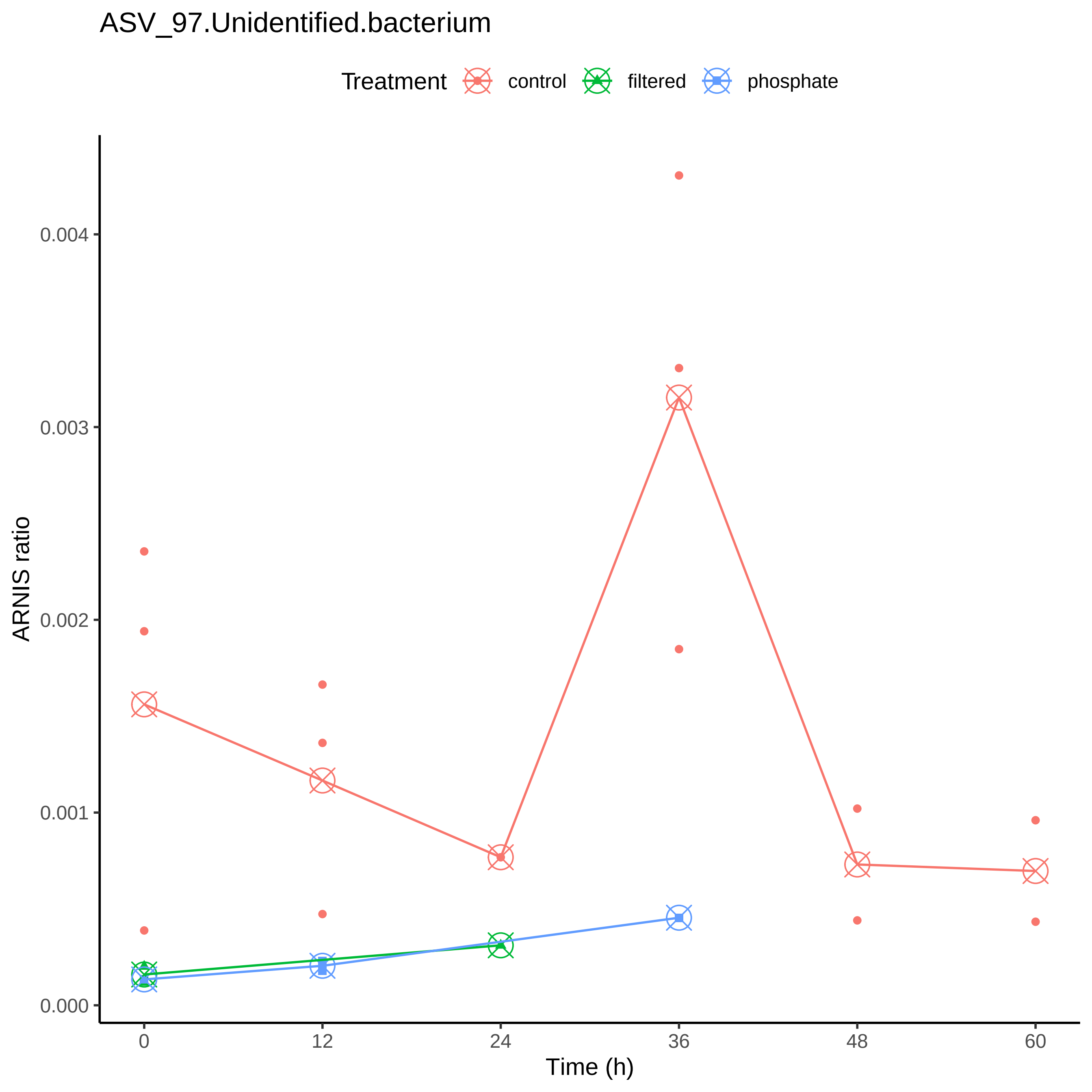

ASV_97.Unidentified.bacterium
Treatment
control
filtered
phosphate
ARNIS ratio
0.004
0.003
0.002
0.001
0.000
0
12
24
36
48
60
Time (h)

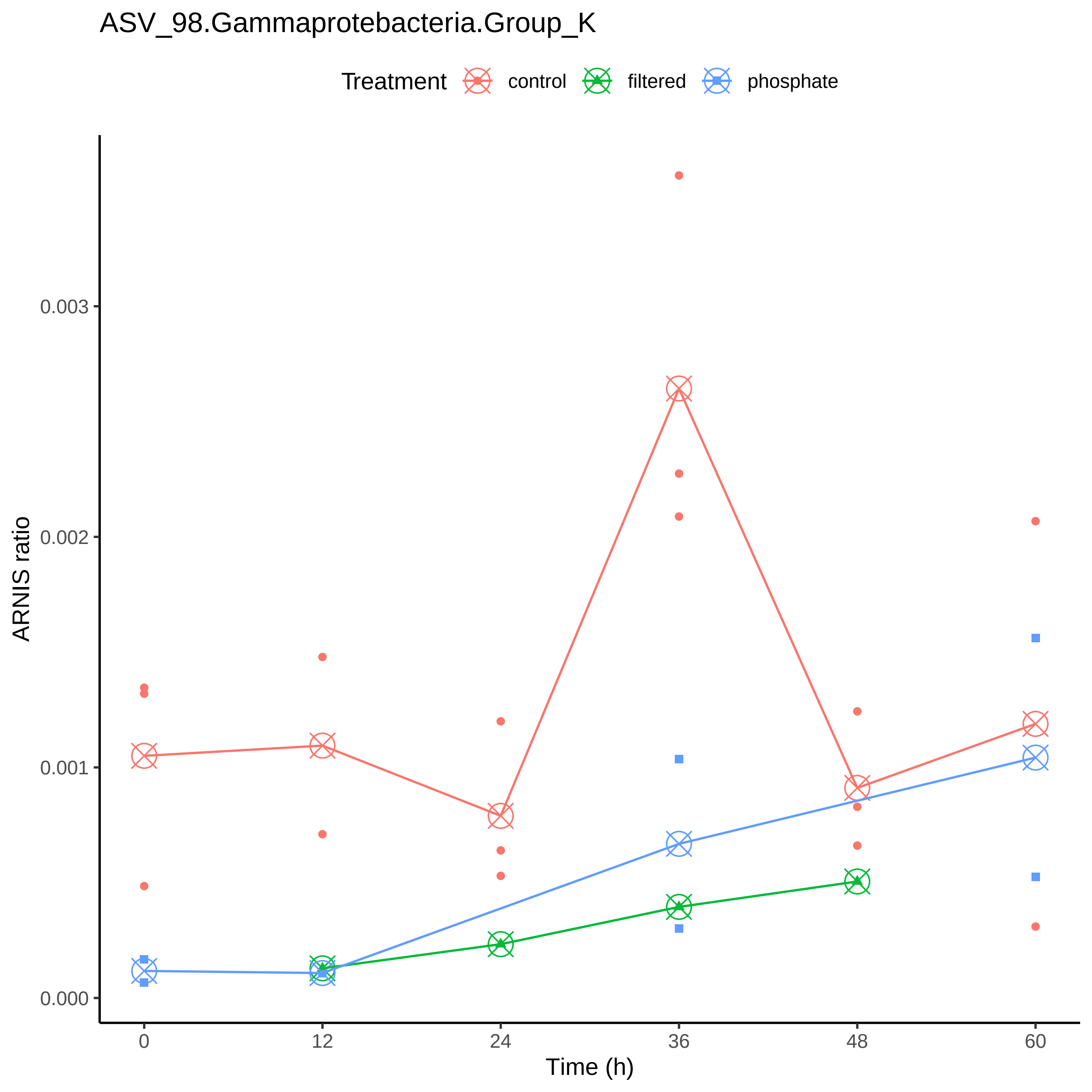

ASV_98.Gammaprotebacteria.Group_K
Treatment
control
filtered
phosphate
ARNIS ratio
0.003
0.002
0.001
0.000
0
12
24
36
48
60
Time (h)

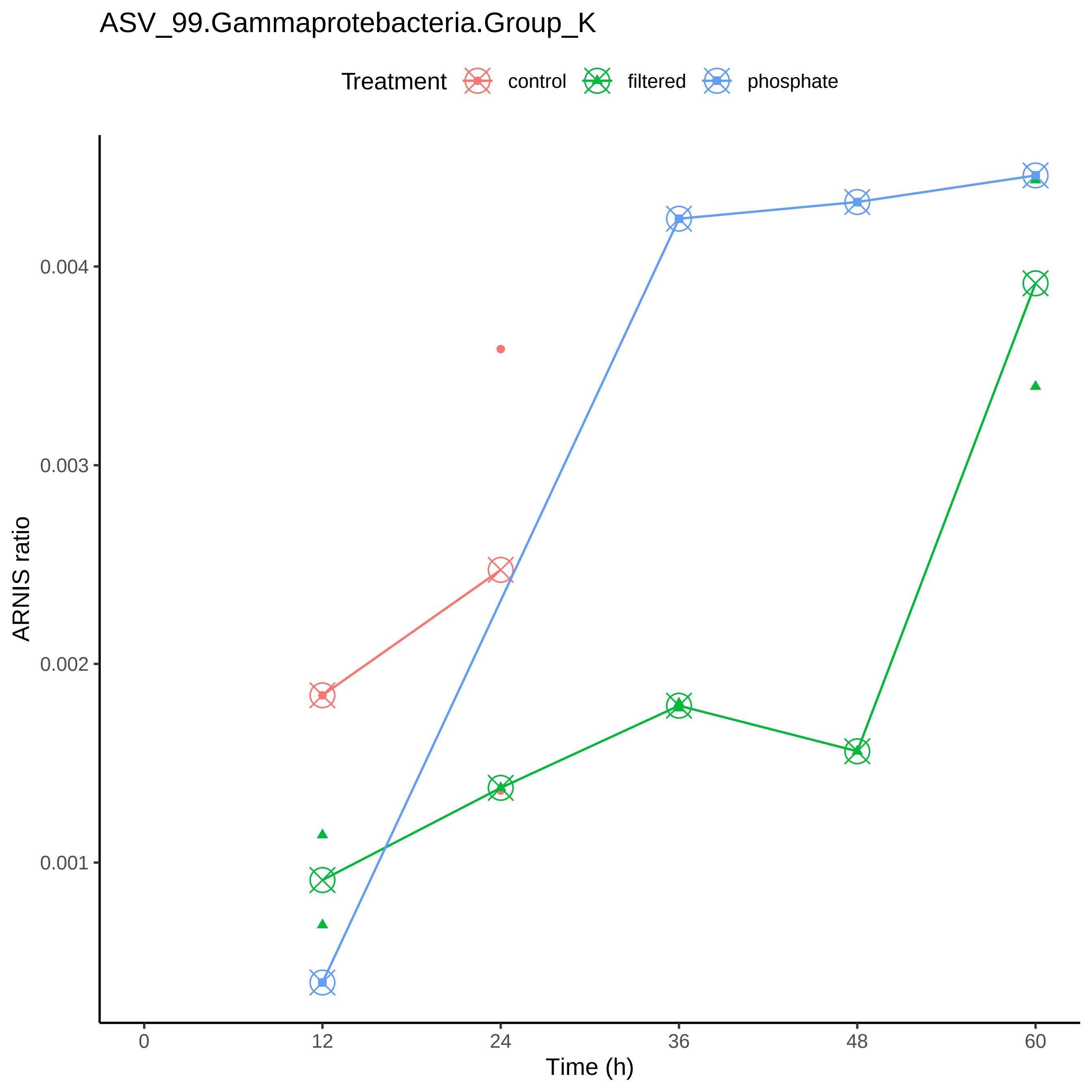

ASV_99.Gammaprotebacteria.Group_K
Treatment
control
filtered
phosphate
ARNIS ratio
0.001
0.002
0.003
0.004
Time (h)
0
12
24
36
48
60

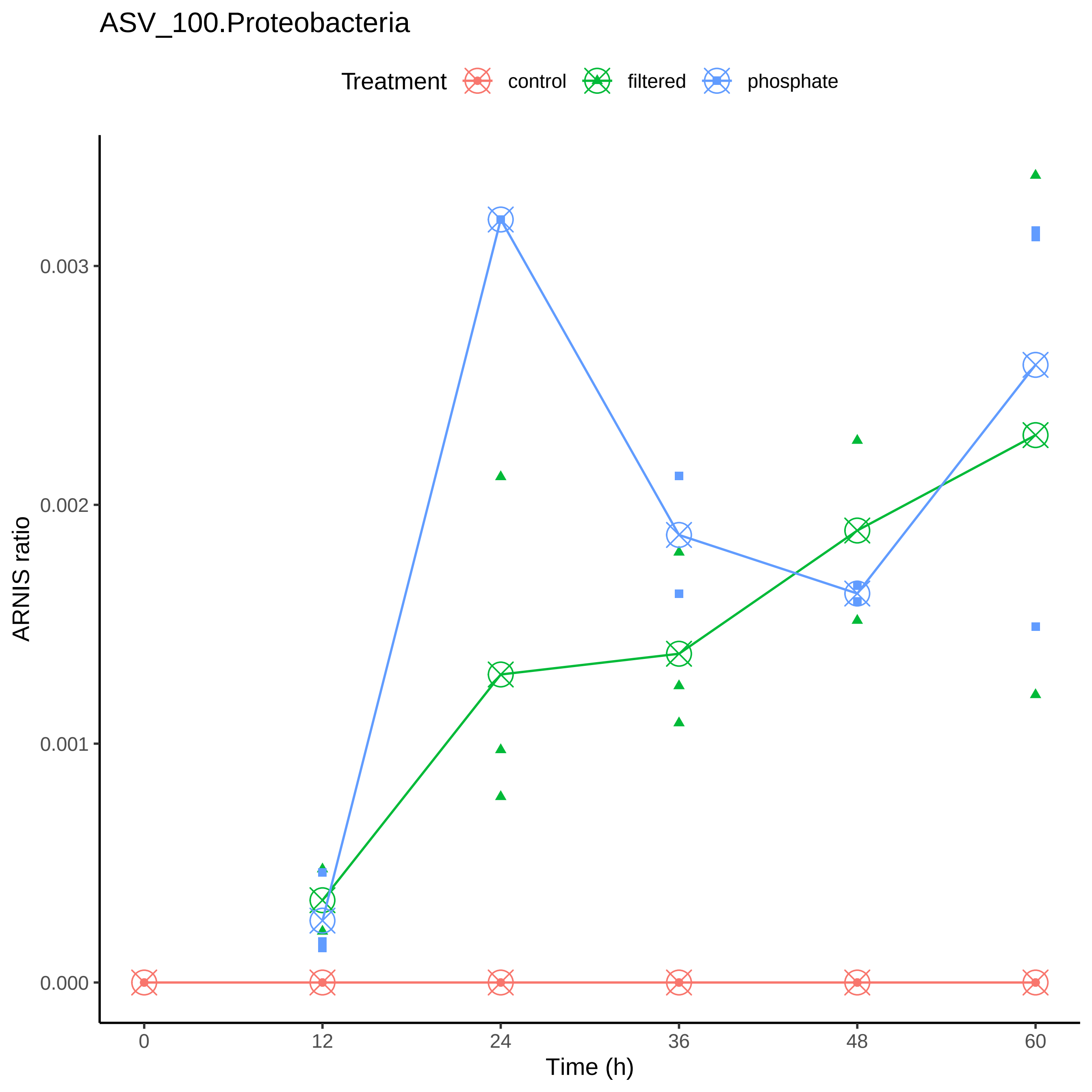
ASV_100.Proteobacteria
Treatment
control
filtered
phosphate
ARNIS ratio
0.003
0.002
0.001
0.000
0
12
24
36
48
60
Time (h)

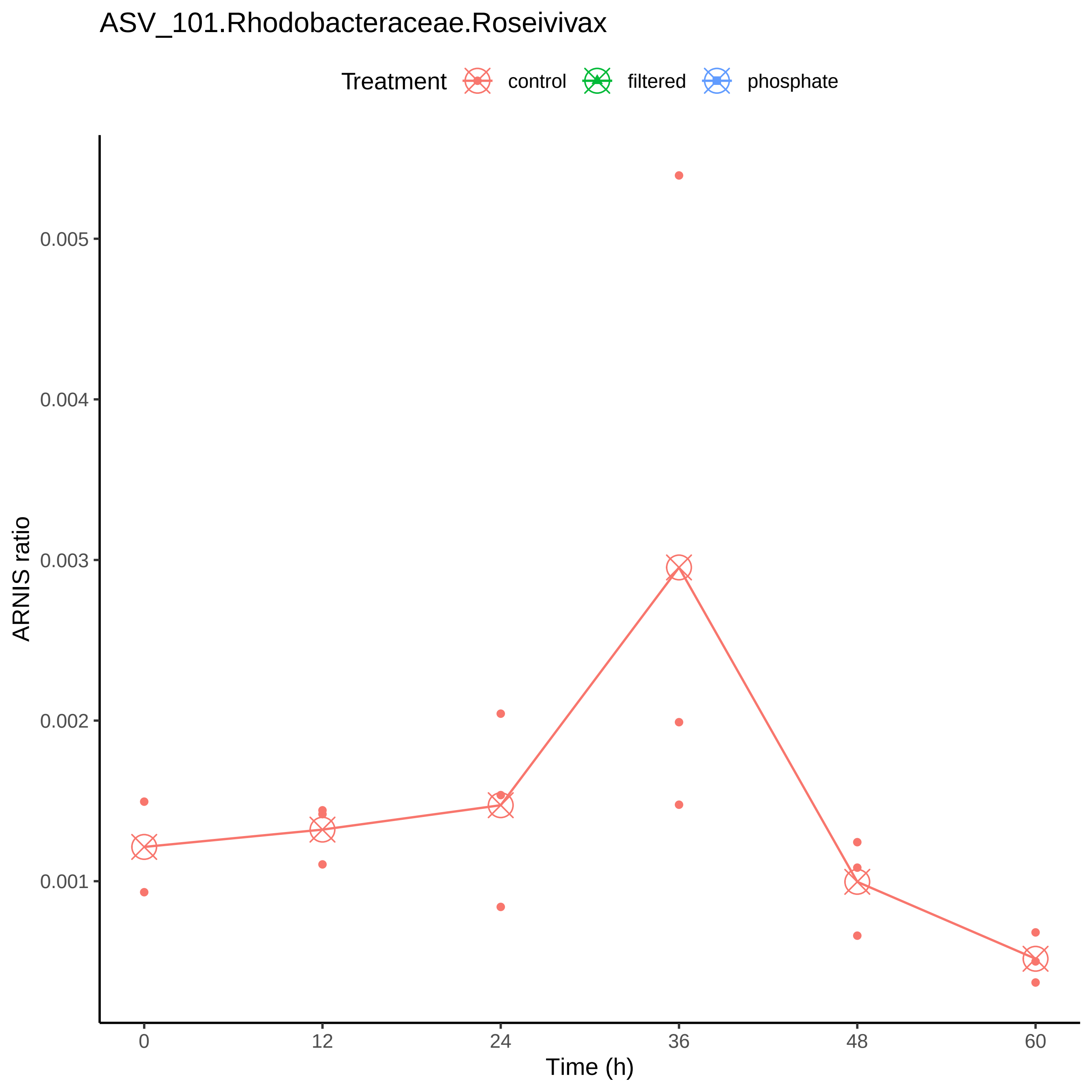

ASV_101.Rhodobacteraceae.Roseivivax
Treatment
control
filtered
phosphate
ARNIS ratio
0.005
0.004
0.003
0.002
0.001
0
12
24
36
48
60
Time (h)

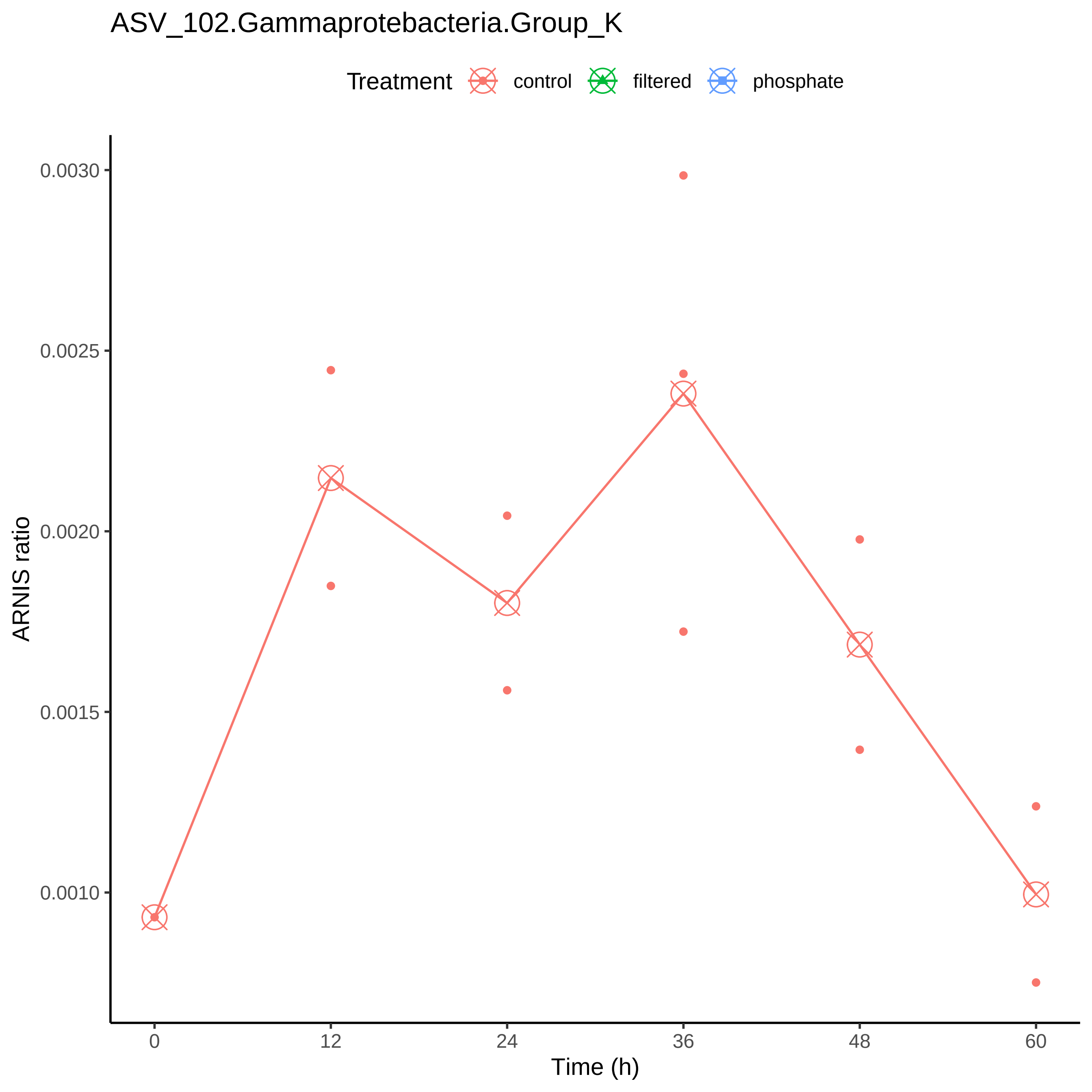
ASV_102.Gammaprotebacteria.Group_K
Treatment
control
filtered
phosphate
0.0030
0.0025
0.0020
0.0015
0.0010
ARNIS ratio
0
12
24
36
48
60
Time (h)

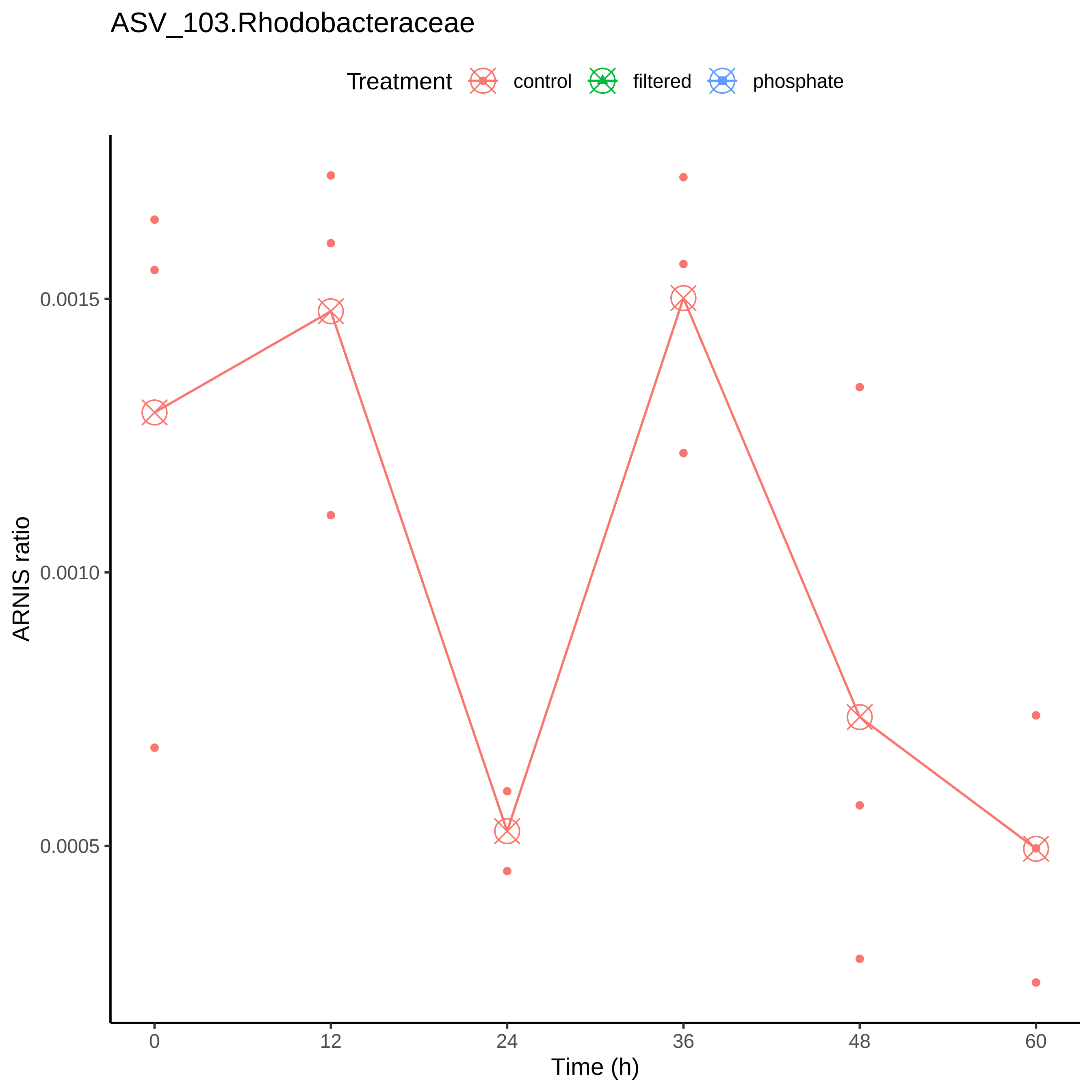

ASV_103.Rhodobacteraceae
Treatment
control
filtered
phosphate
ARNIS ratio
0.0015
0.0010
0.0005
0
12
24
36
48
60
Time (h)

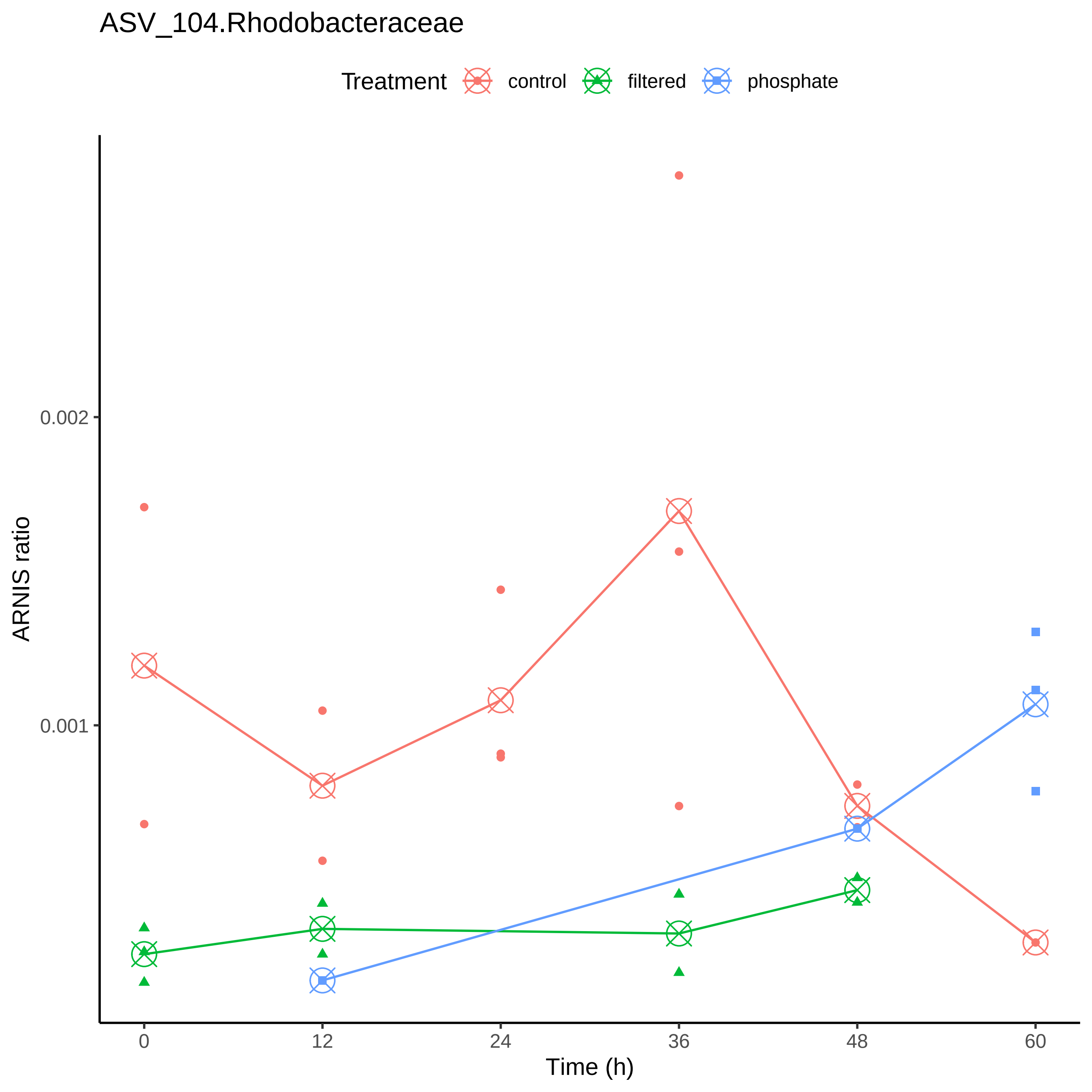

ASV_104.Rhodobacteraceae
Treatment
control
filtered
phosphate
ARNIS ratio
0.002
0.001
0
12
24
36
48
60
Time (h)

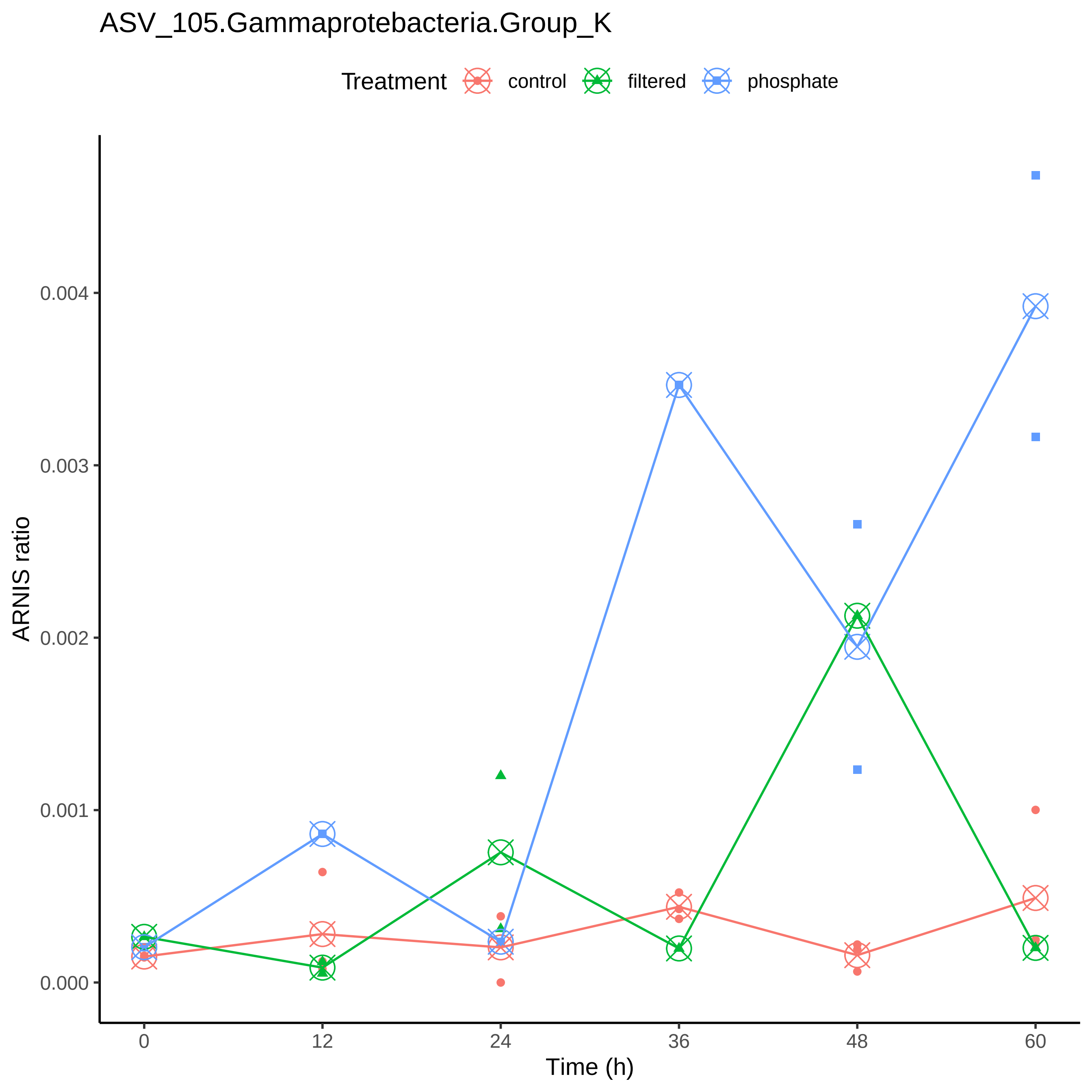

ASV_105.Gammaprotebacteria.Group_K
Treatment
control
filtered
phosphate
ARNIS ratio
0.000
0.001
0.002
0.003
0.004
Time (h)
0
12
24
36
48
60

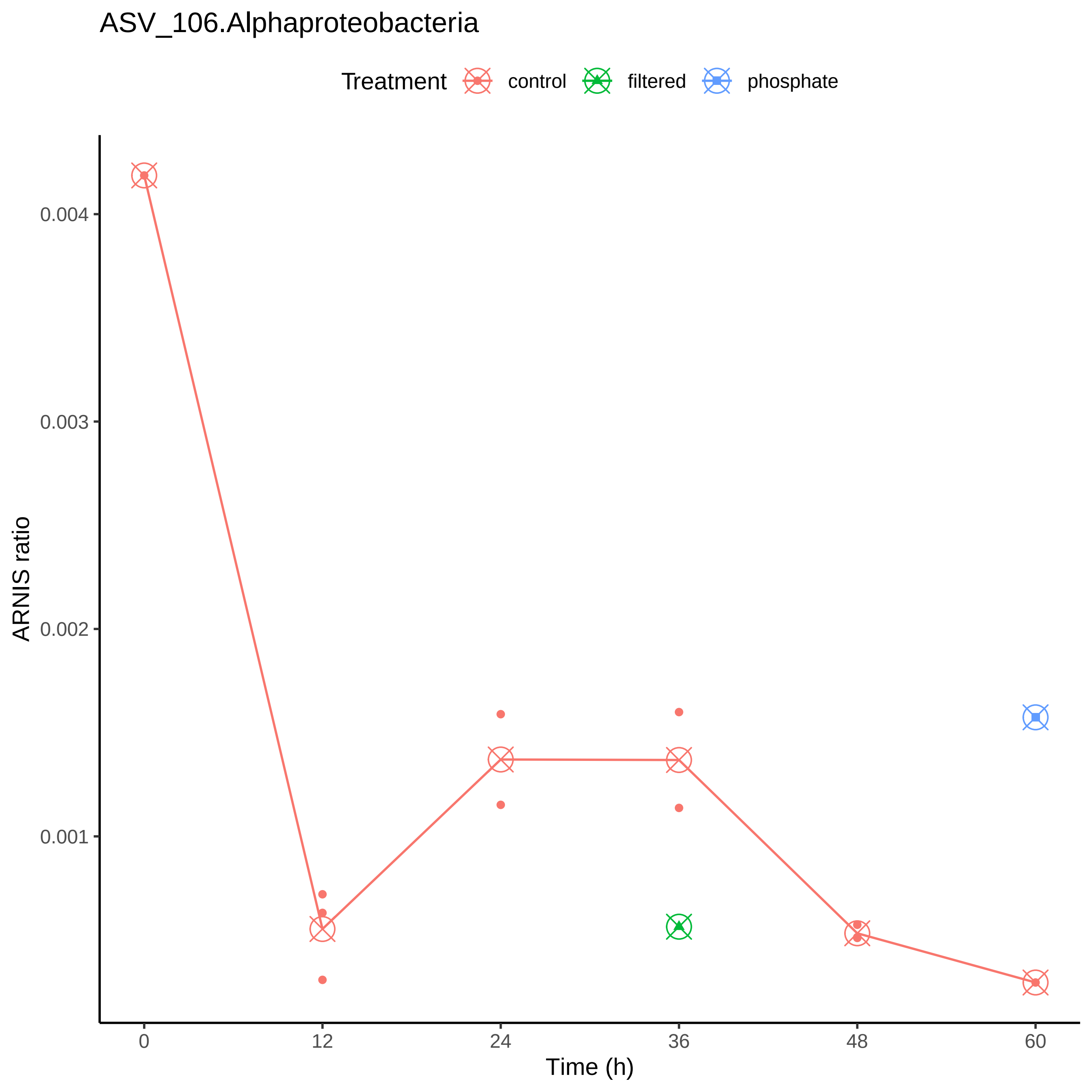
ASV_106.Alphaproteobacteria
Treatment
control
filtered
phosphate
0.004
0.003
0.002
0.001
ARNIS ratio
0
12
24
36
48
60
Time (h)

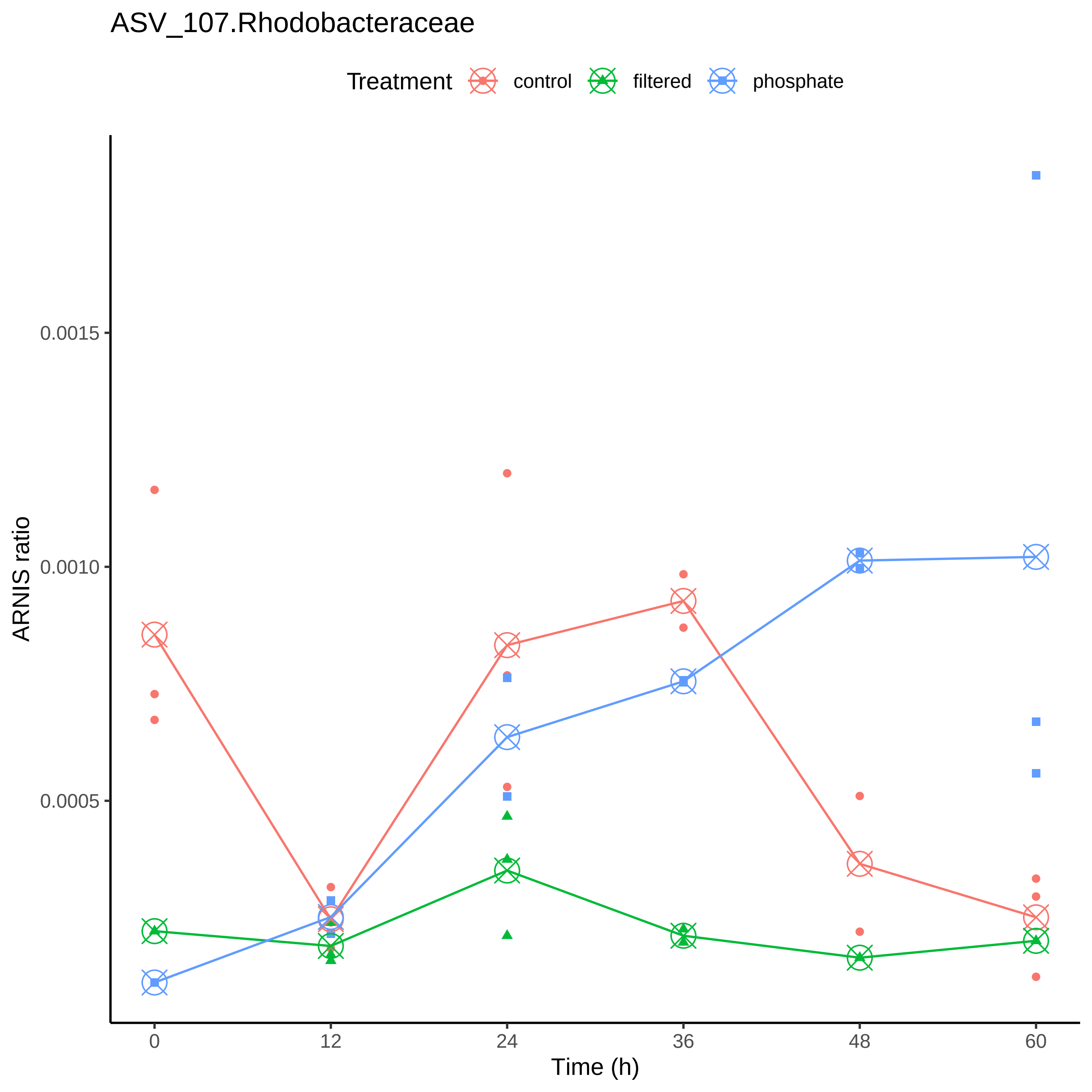

ASV_107.Rhodobacteraceae
Treatment
control
filtered
phosphate
ARNIS ratio
0.0015
0.0010
0.0005
0
12
24
36
48
60
Time (h)

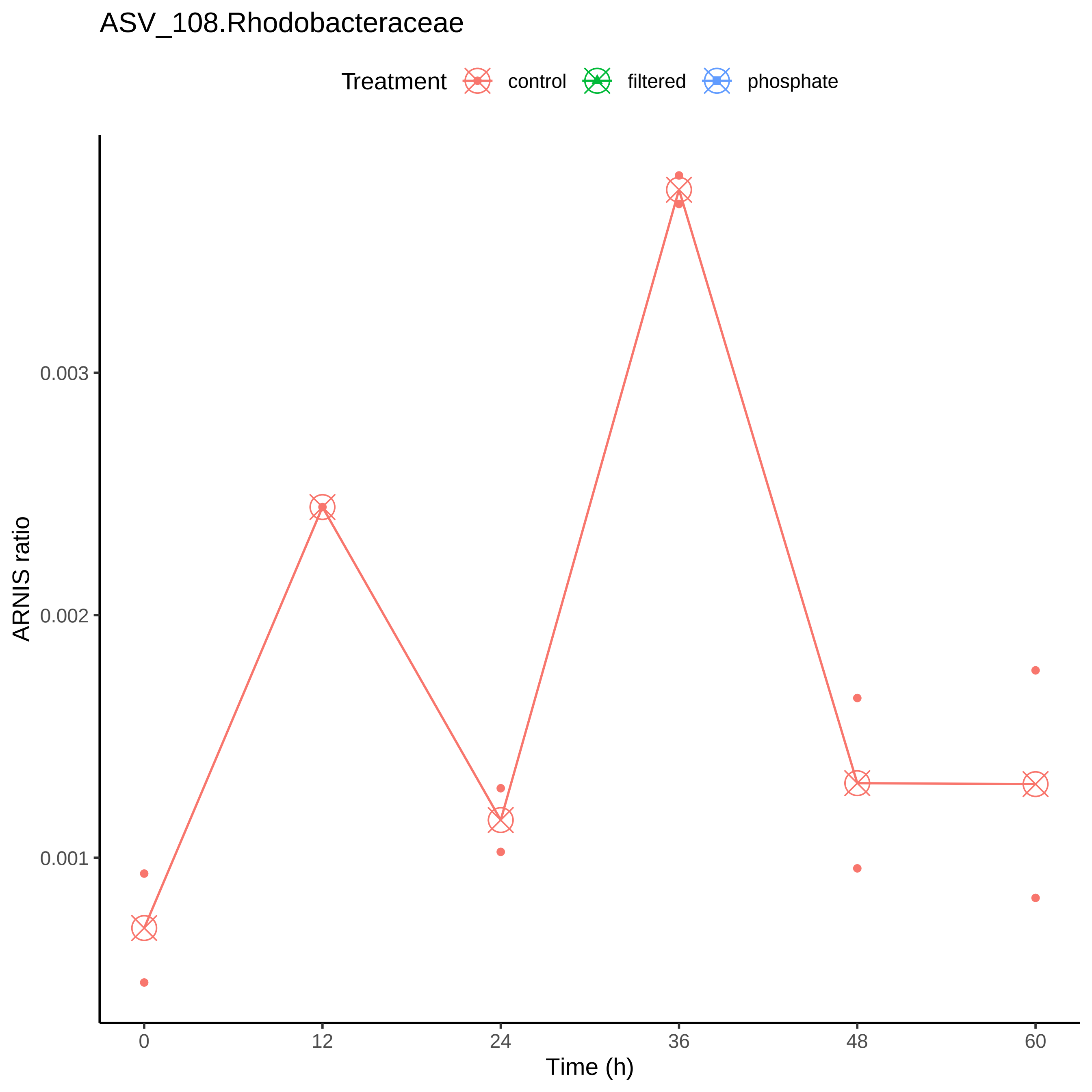

ASV_108.Rhodobacteraceae
Treatment
control
filtered
phosphate
ARNIS ratio
0.003
0.002
0.001
0
12
24
36
48
60
Time (h)

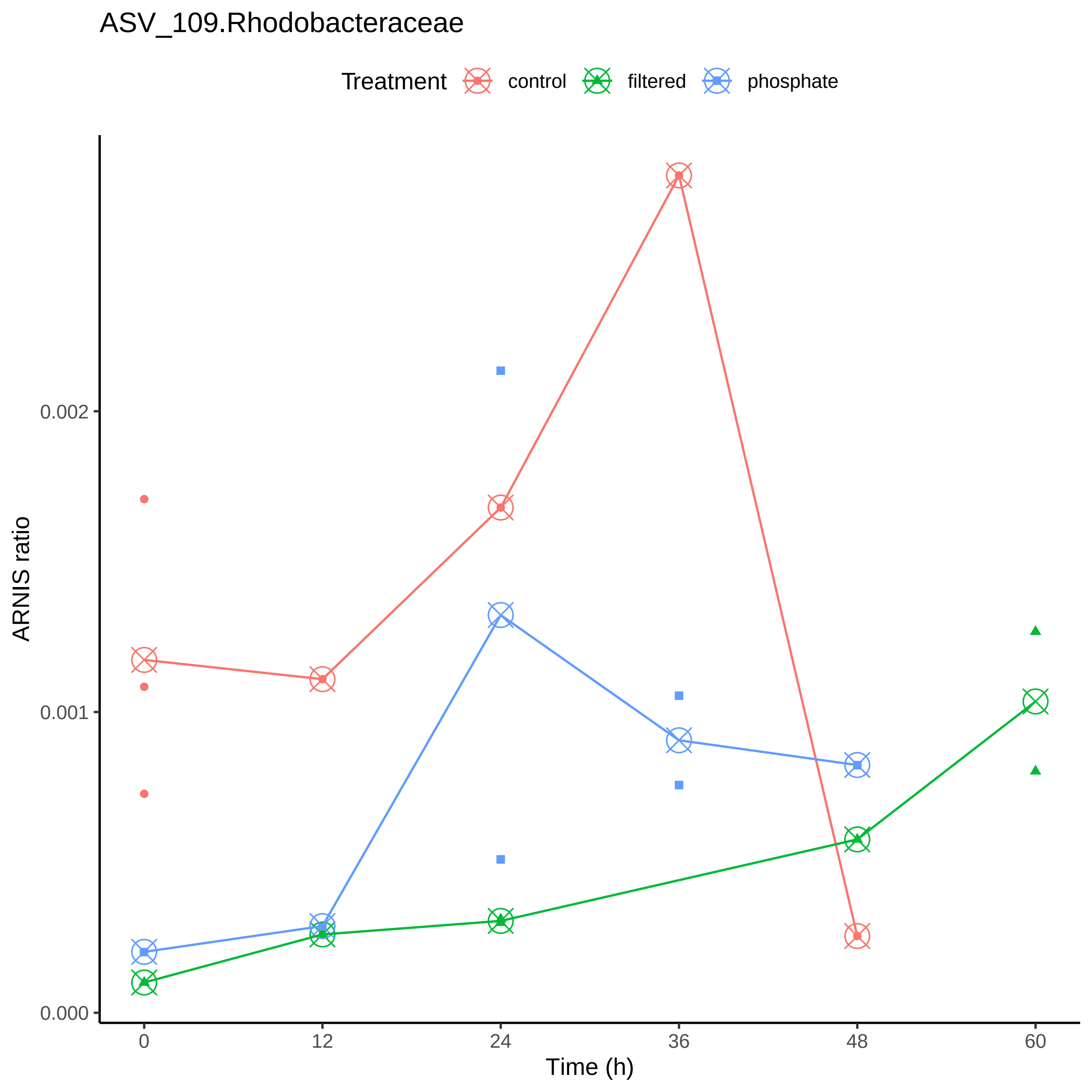
ASV_109.Rhodobacteraceae
Treatment
control
filtered
phosphate
ARNIS ratio
0.000
0.001
0.002
0
12
24
36
48
60
Time (h)

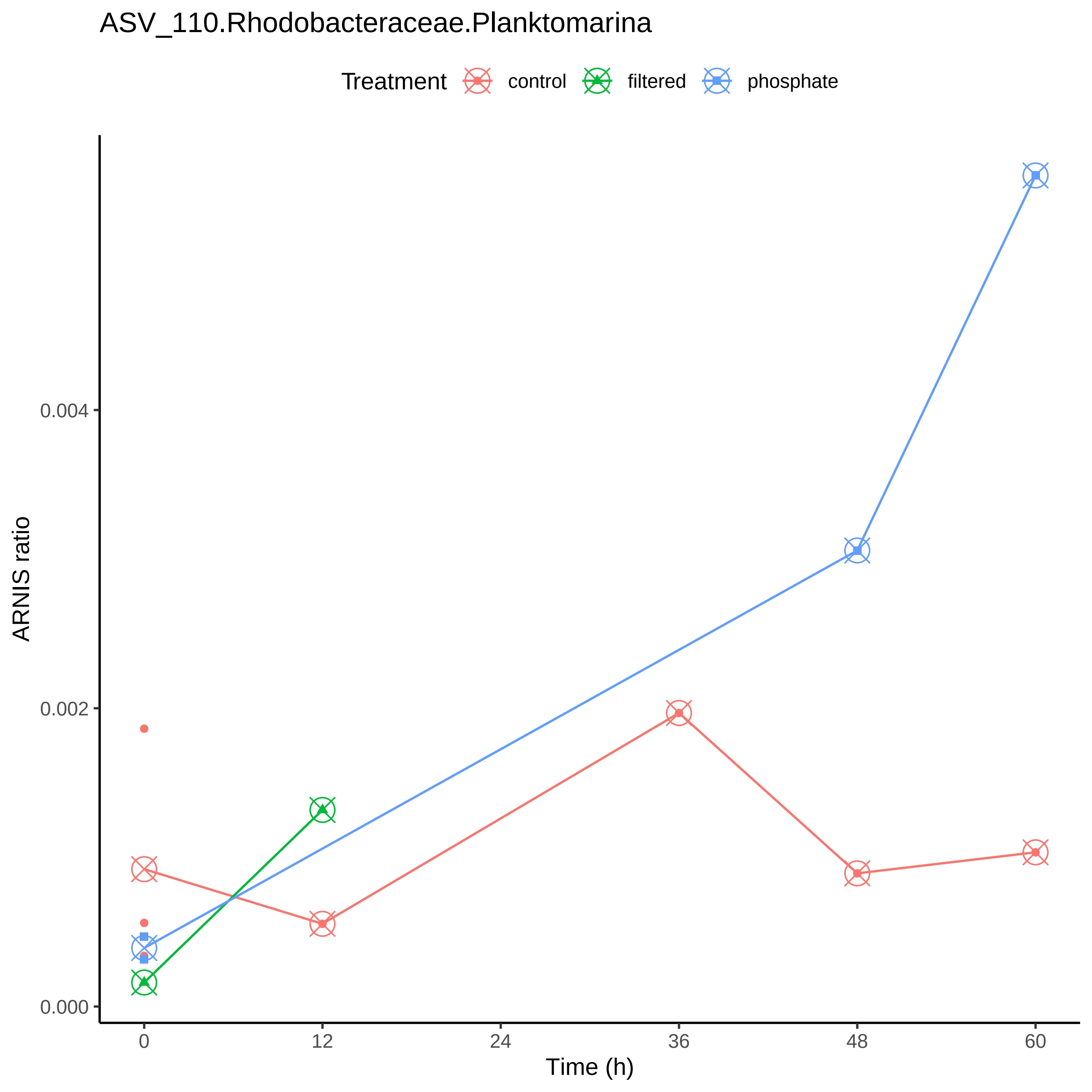
ASV_110.Rhodobacteraceae.Planktomarina
Treatment
control
filtered
phosphate
ARNIS ratio
0.004
0.002
0.000
0
12
24
36
48
60
Time (h)

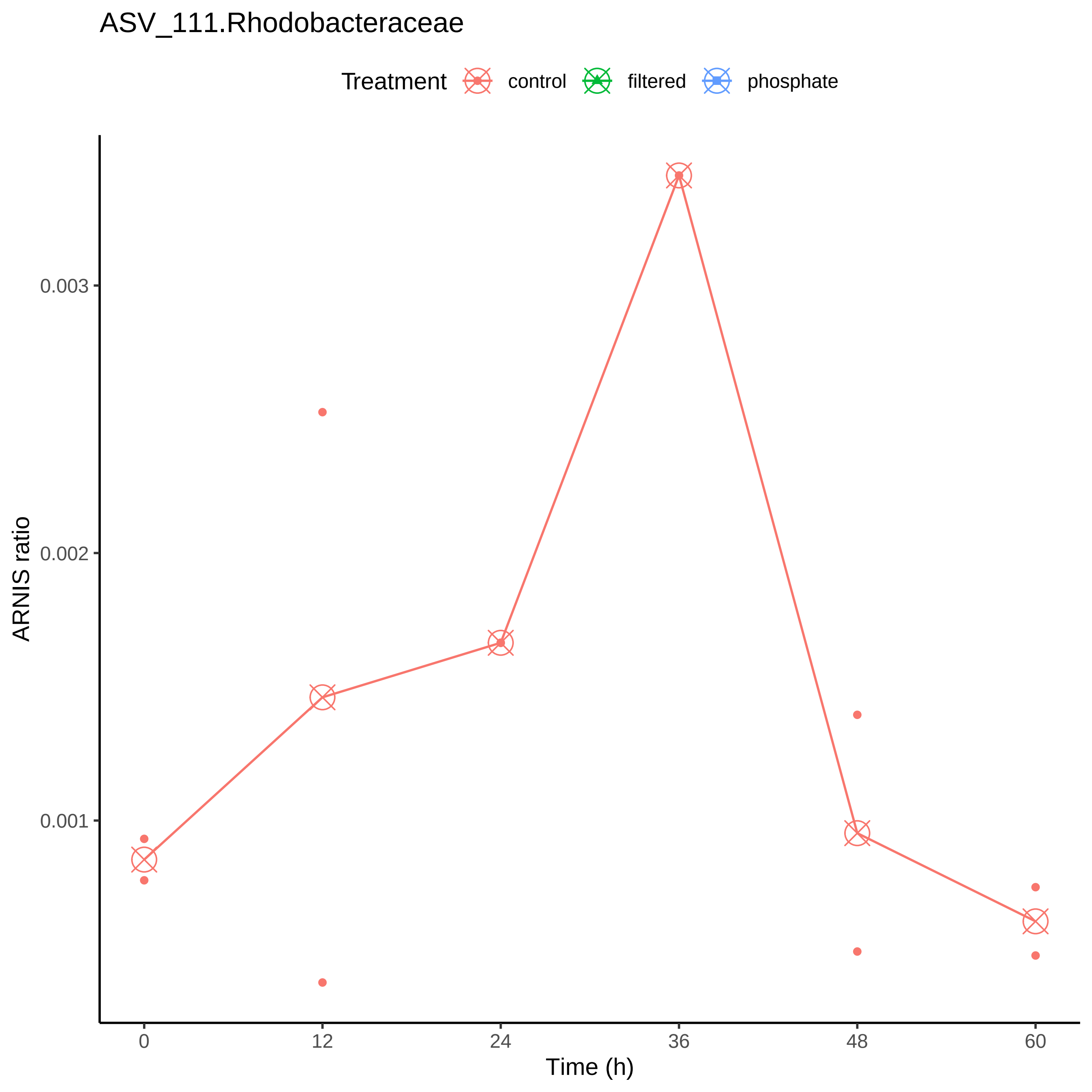
ASV_111.Rhodobacteraceae
Treatment
control
filtered
phosphate
ARNIS ratio
0.003
0.002
0.001
0
12
24
36
48
60
Time (h)

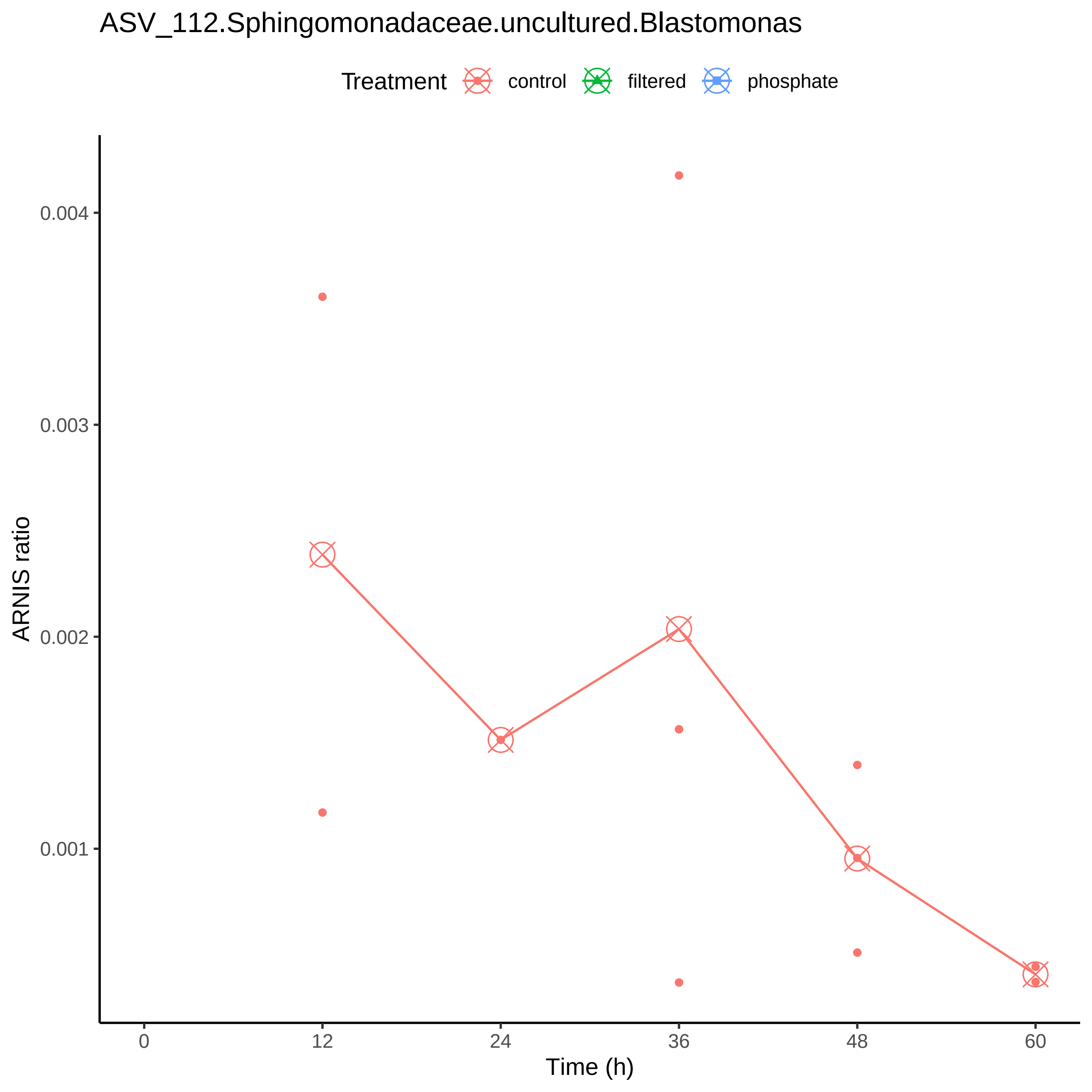
ASV_112.Sphingomonadaceae.uncultured.Blastomonas
Treatment
control
filtered
phosphate
ARNIS ratio
0.004
0.003
0.002
0.001
0
12
24
36
48
60
Time (h)

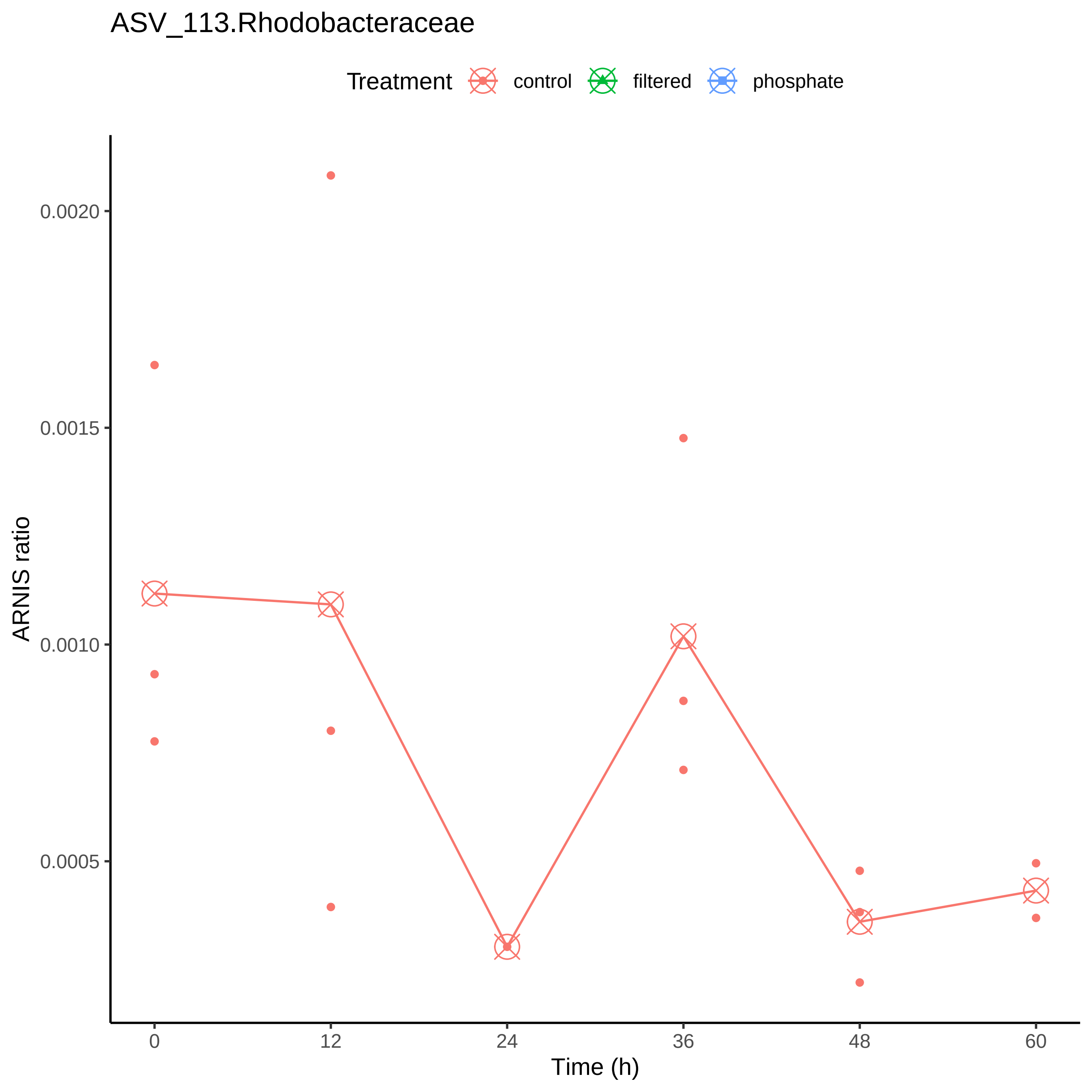
ASV_113.Rhodobacteraceae
Treatment
control
filtered
phosphate
ARNIS ratio
0.0020
0.0015
0.0010
0.0005
0
12
24
36
48
60
Time (h)

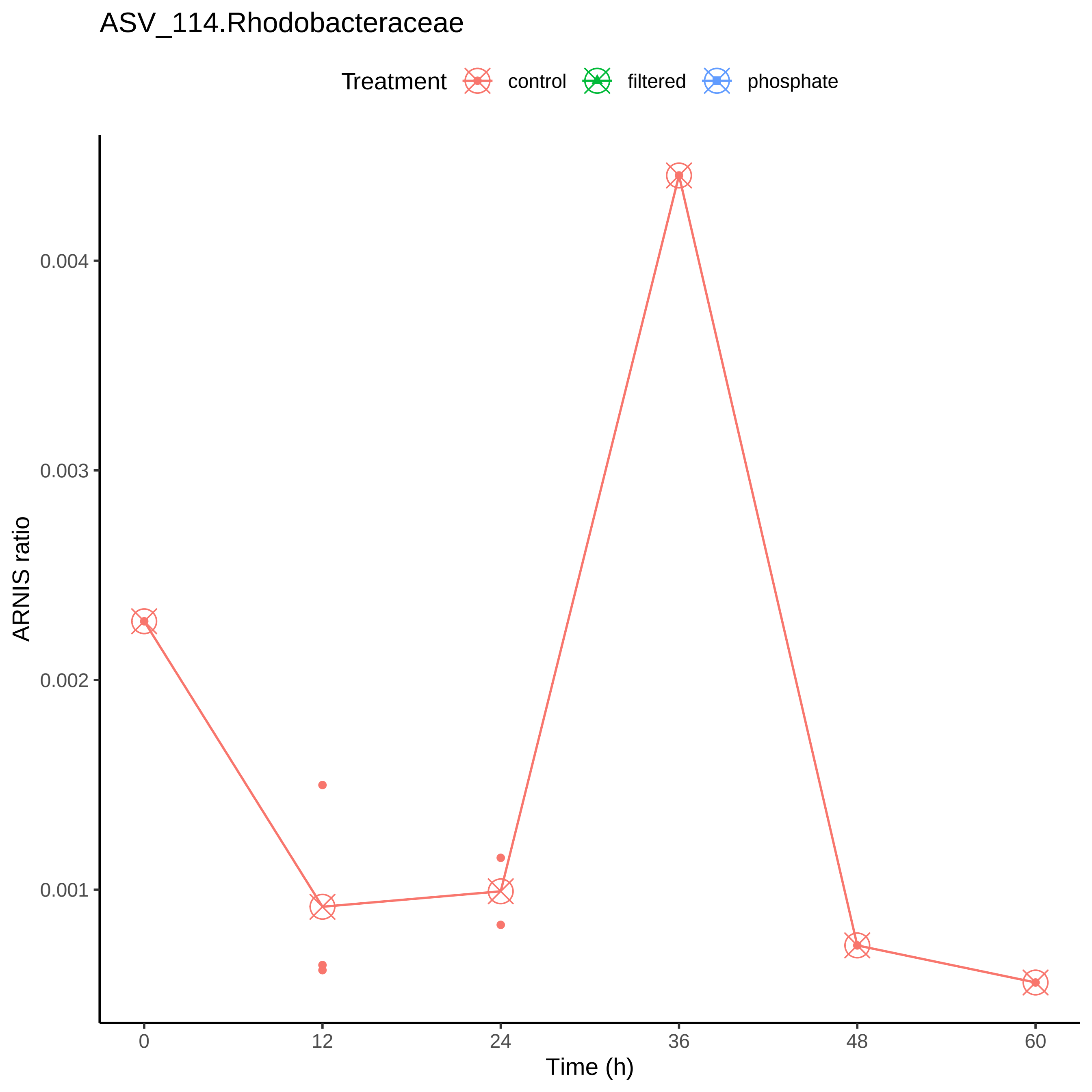
ASV_114.Rhodobacteraceae
Treatment
control
filtered
phosphate
ARNIS ratio
0.004
0.003
0.002
0.001
0
12
24
36
48
60
Time (h)

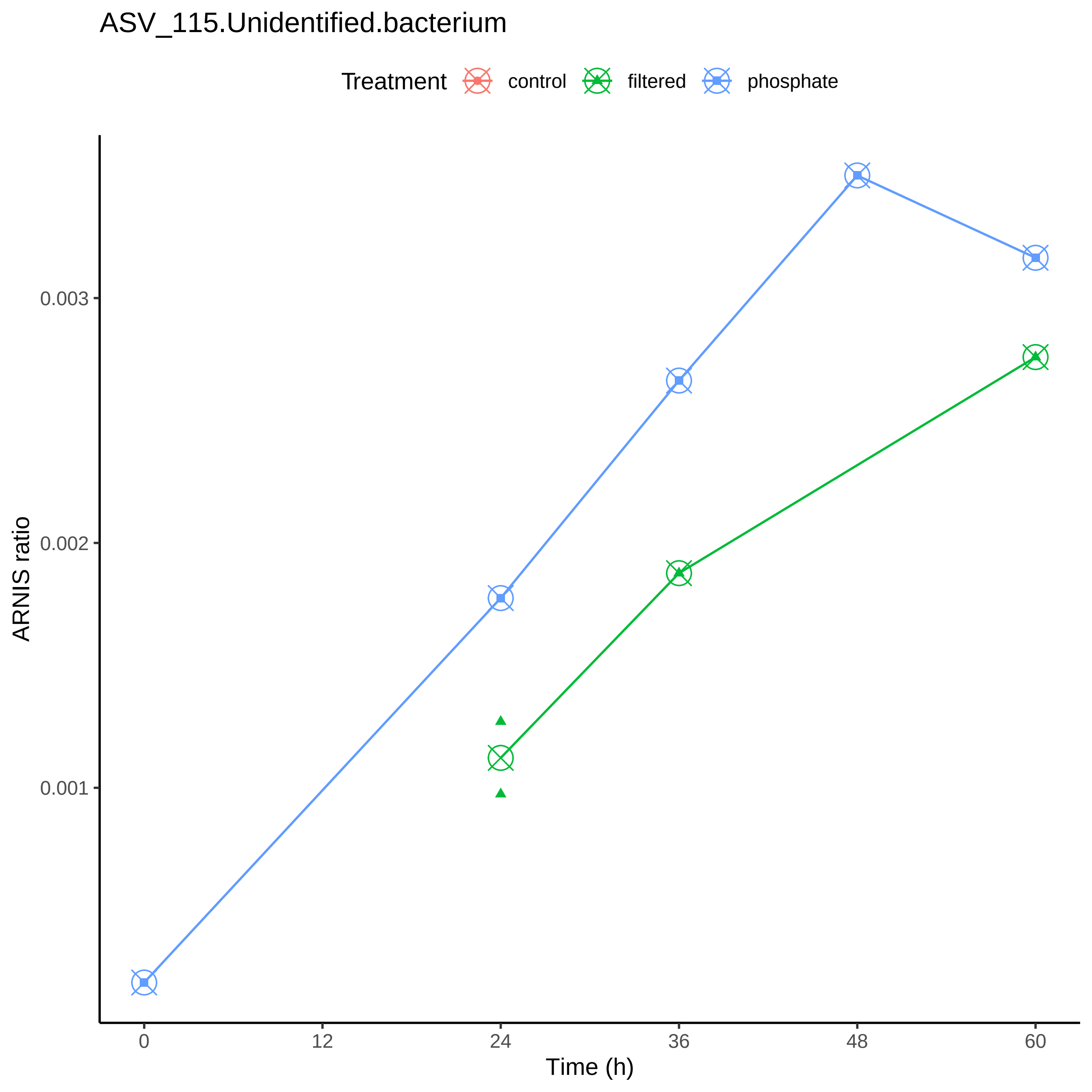

ASV_115.Unidentified.bacterium
Treatment
control
filtered
phosphate
ARNIS ratio
0.003
0.002
0.001
0
12
24
36
48
60
Time (h)

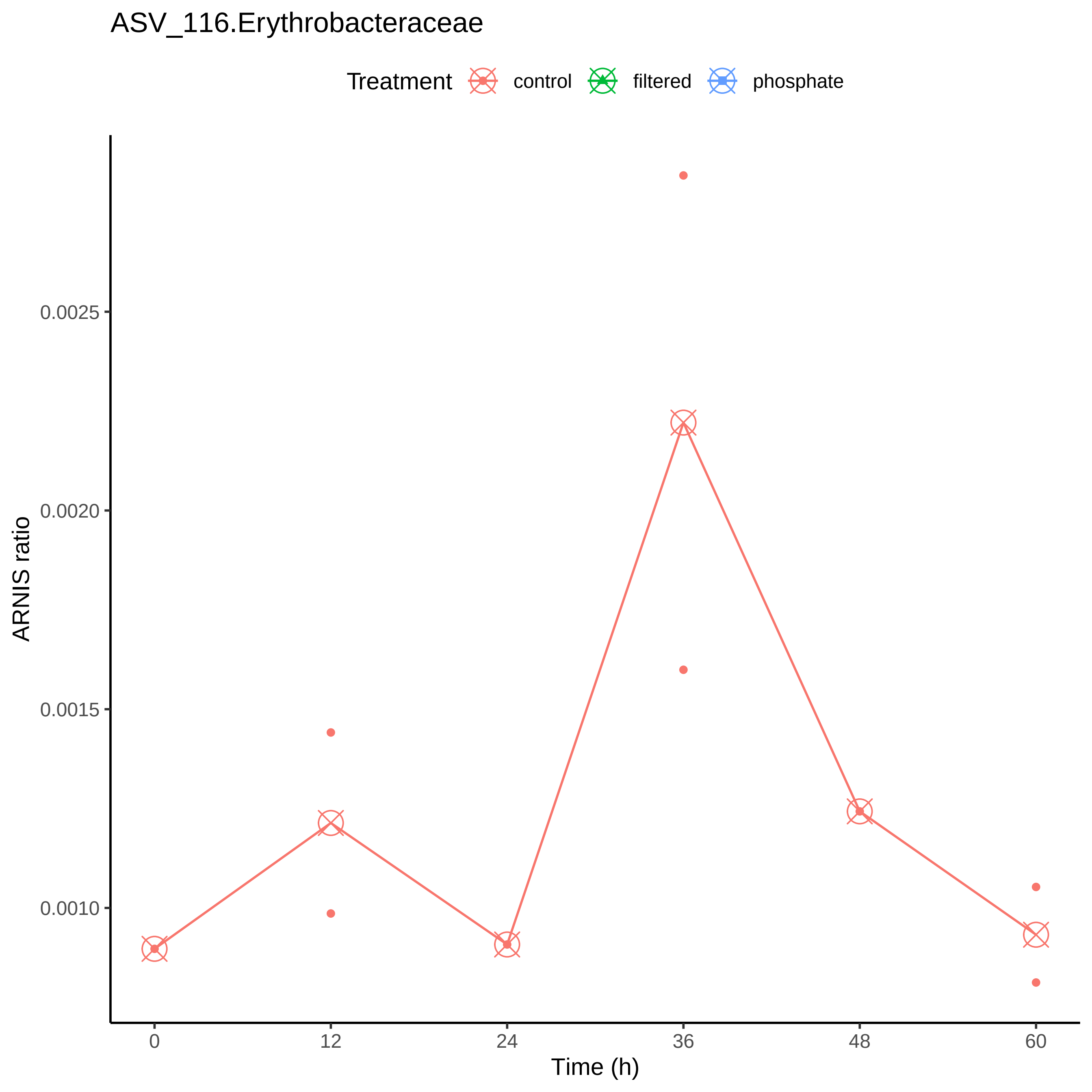

ASV_116.Erythrobacteraceae
Treatment
control
filtered
phosphate
ARNIS ratio
0.0025
0.0020
0.0015
0.0010
0
12
24
36
48
60
Time (h)

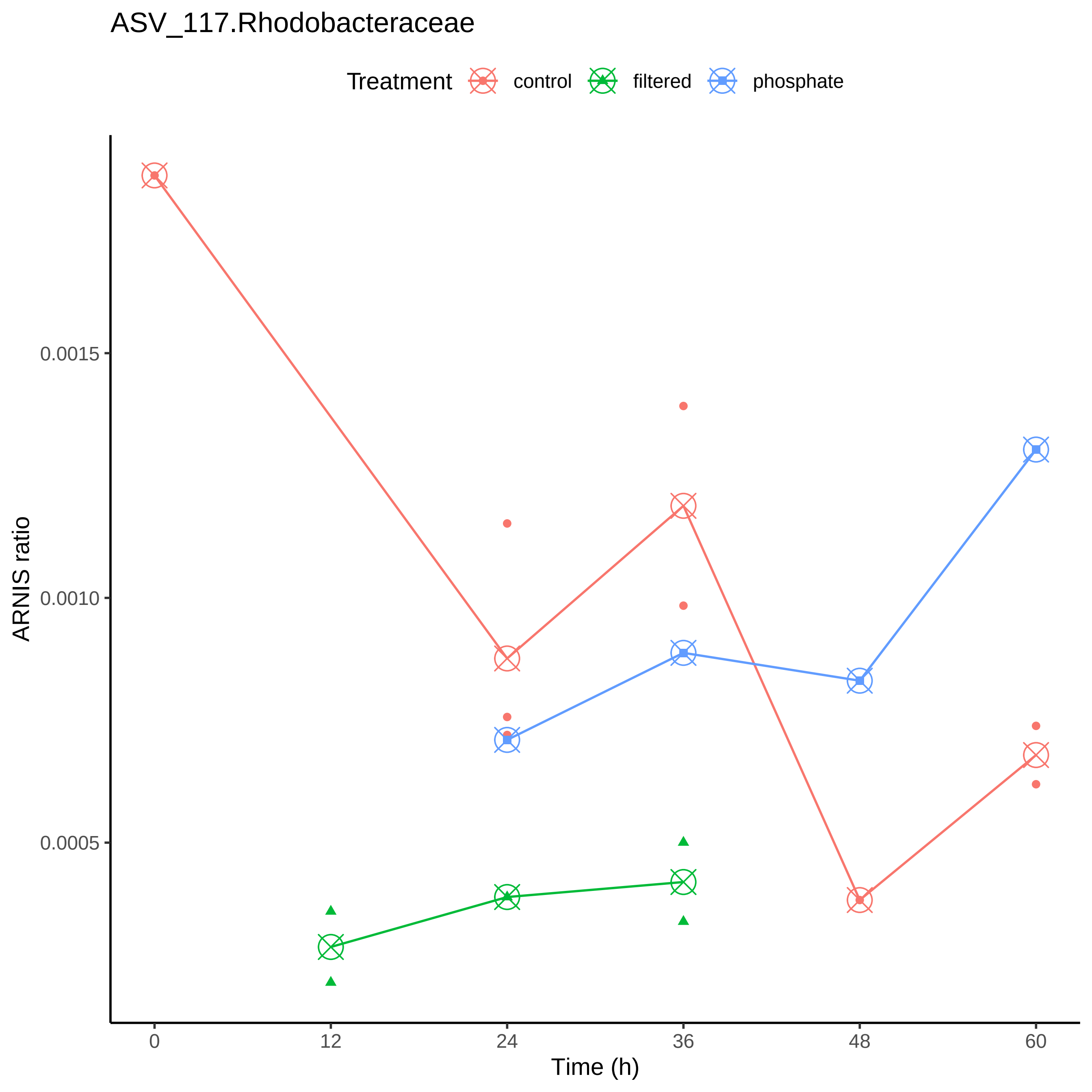

ASV_117.Rhodobacteraceae
Treatment
control
filtered
phosphate
ARNIS ratio
0.0015
0.0010
0.0005
Time (h)
0
12
24
36
48
60

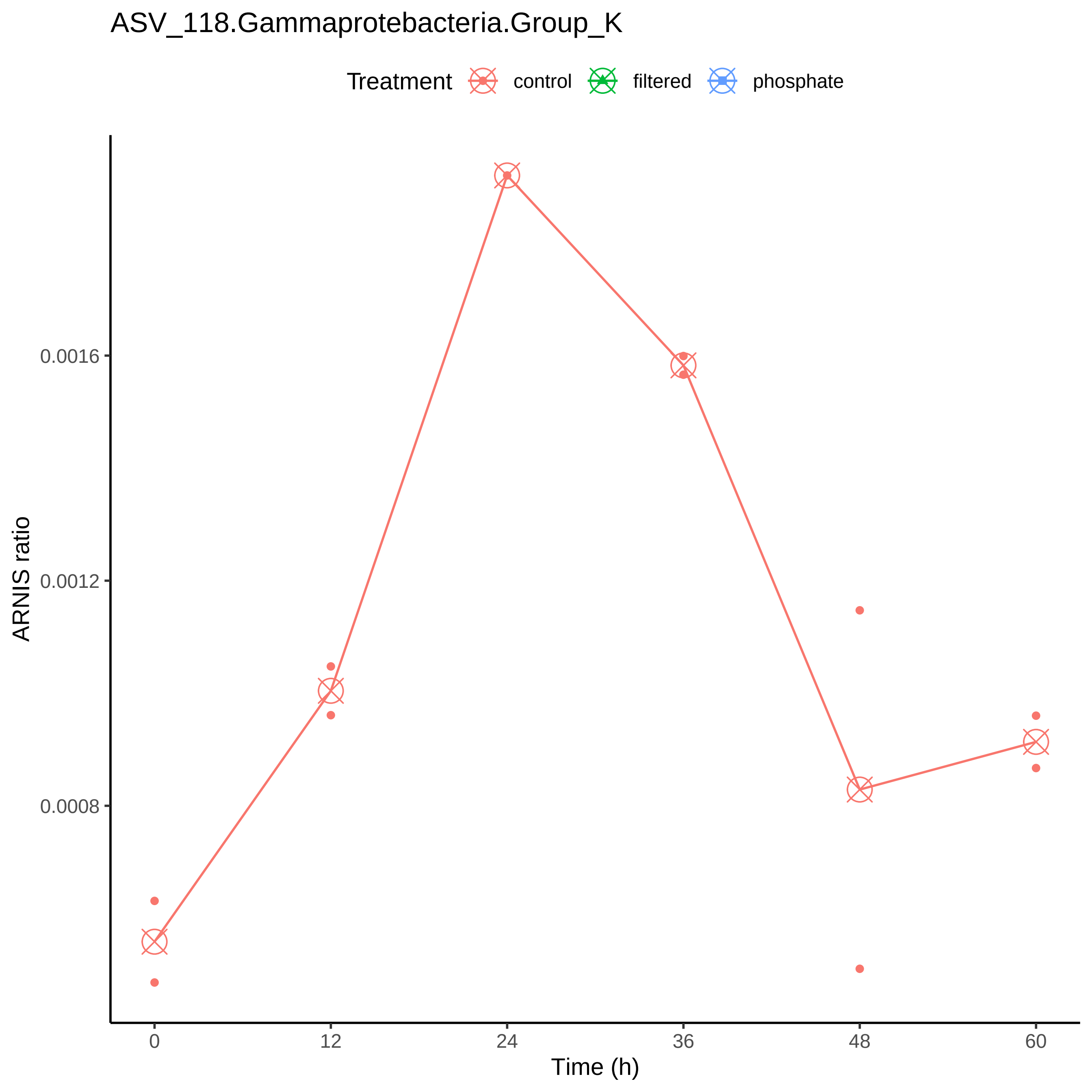
ASV_118.Gammaprotebacteria.Group_K
Treatment
control
filtered
phosphate
ARNIS ratio
0.0016
0.0012
0.0008
0
12
24
36
48
60
Time (h)

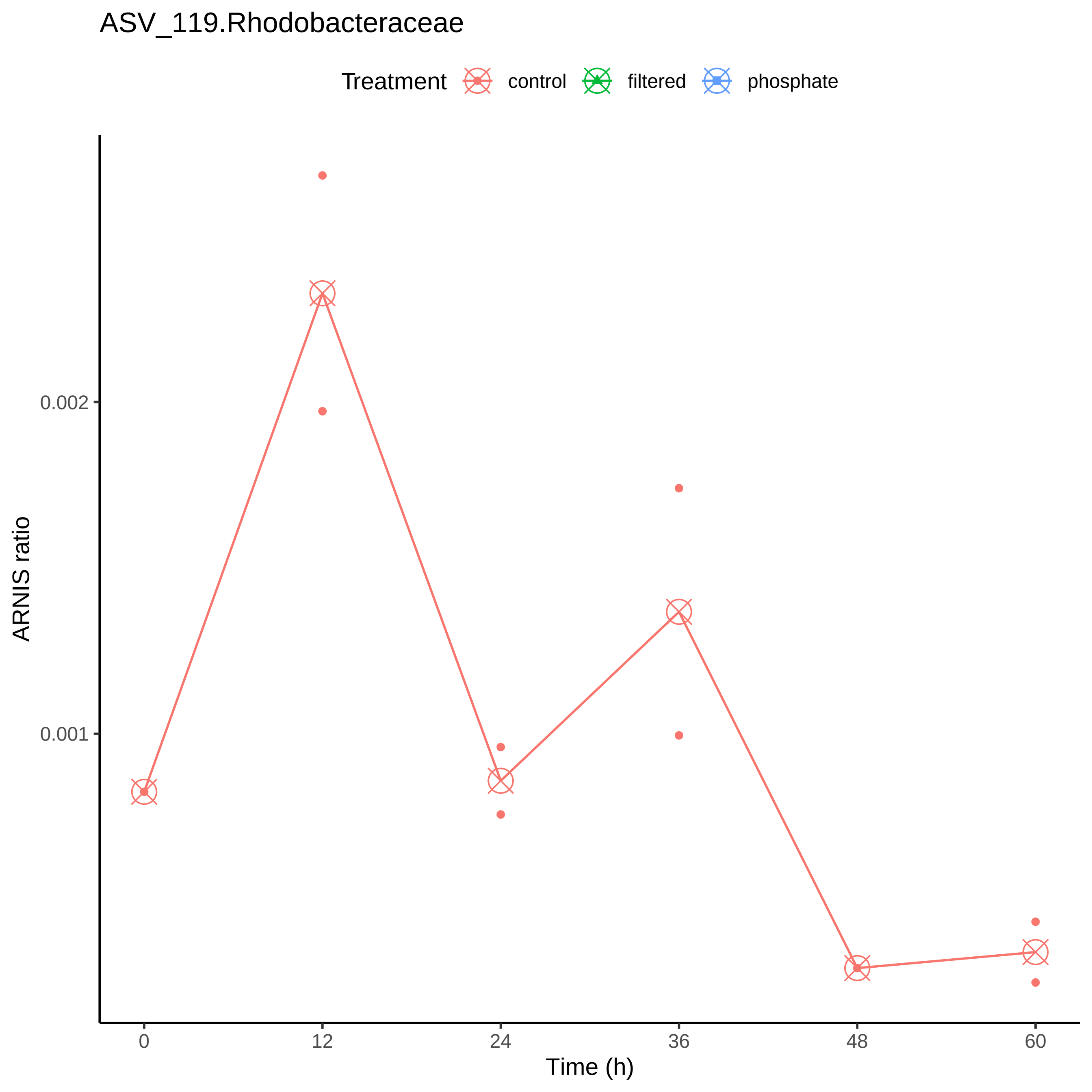
ASV_119.Rhodobacteraceae
Treatment
control
filtered
phosphate
ARNIS ratio
0.002
0.001
0
12
24
36
48
60
Time (h)

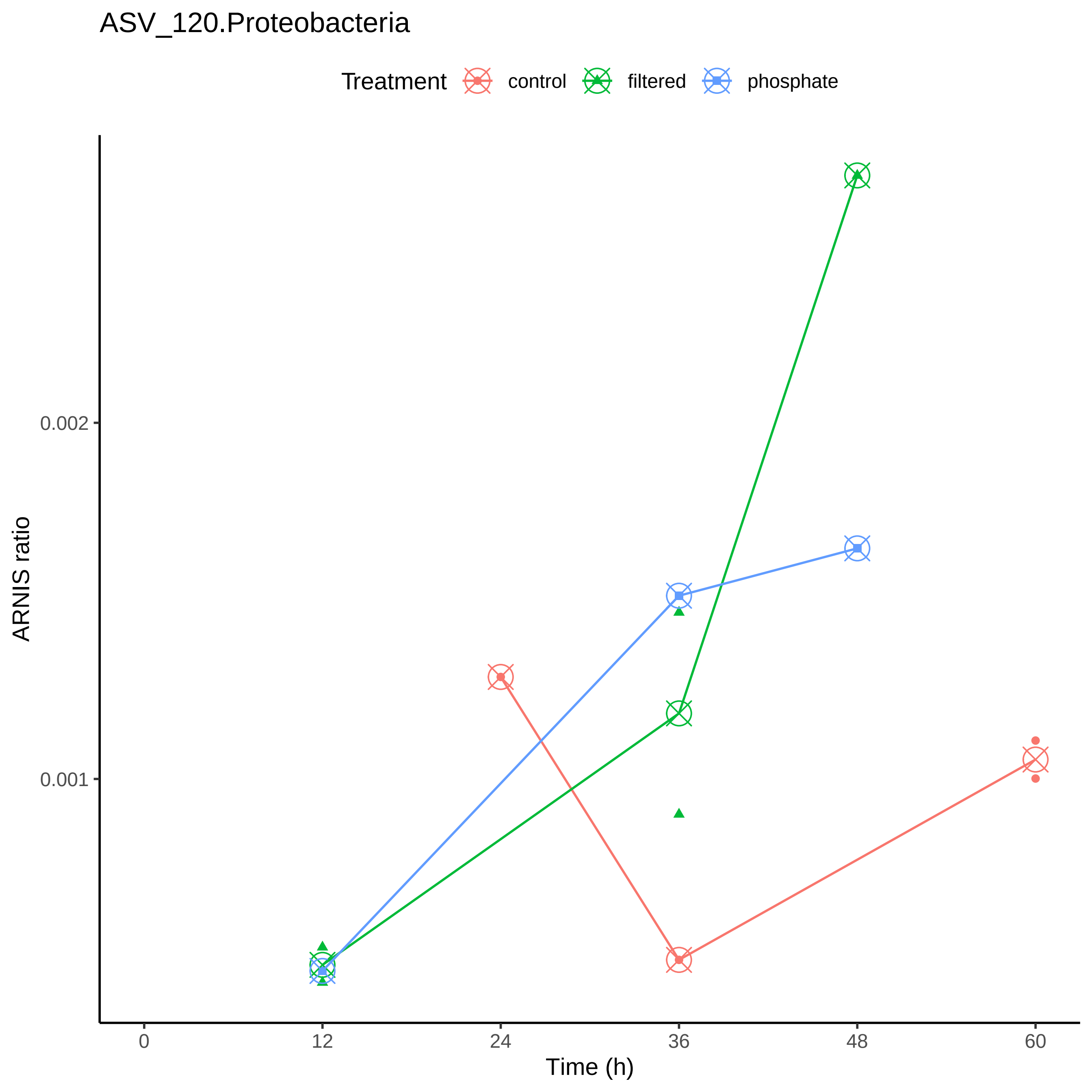

ASV_120.Proteobacteria
Treatment
control
filtered
phosphate
ARNIS ratio
0.002
0.001
0
12
24
36
48
60
Time (h)

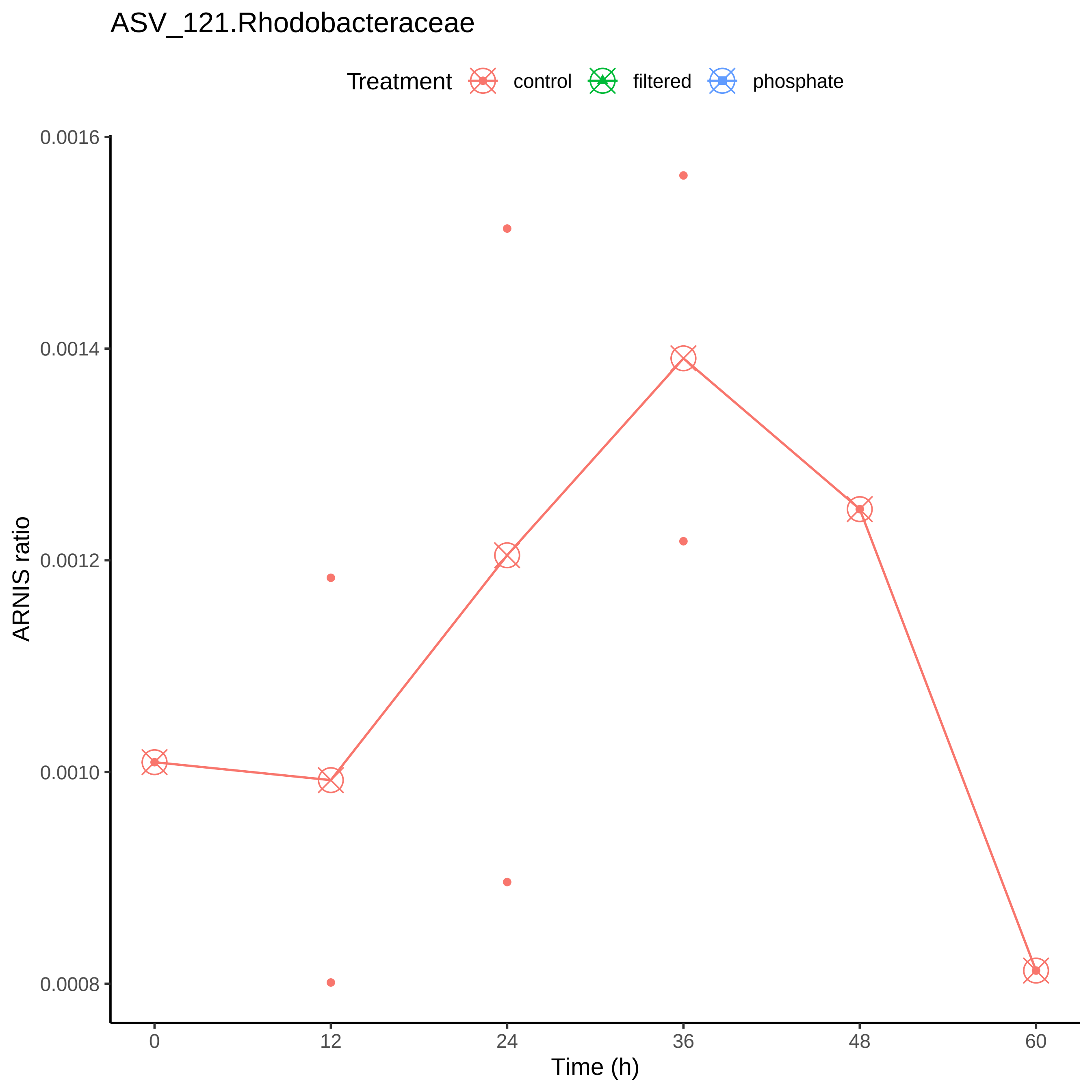
ASV_121.Rhodobacteraceae
Treatment
control
filtered
phosphate
ARNIS ratio
0.0016
0.0014
0.0012
0.0010
0.0008
Time (h)
0
12
24
36
48
60

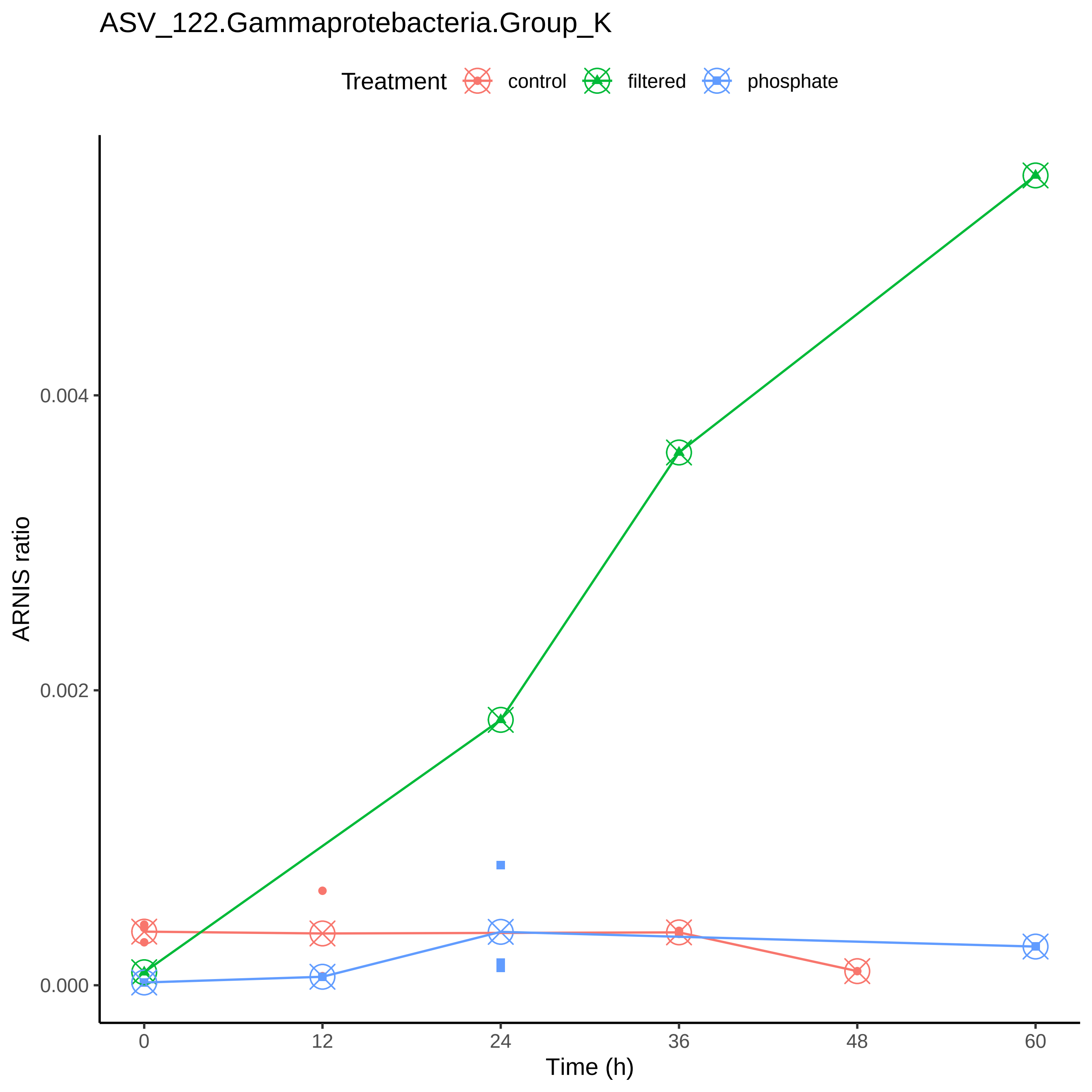

ASV_122.Gammaprotebacteria.Group_K
Treatment
control
filtered
phosphate
ARNIS ratio
0.004
0.002
0.000
0
12
24
36
48
60
Time (h)

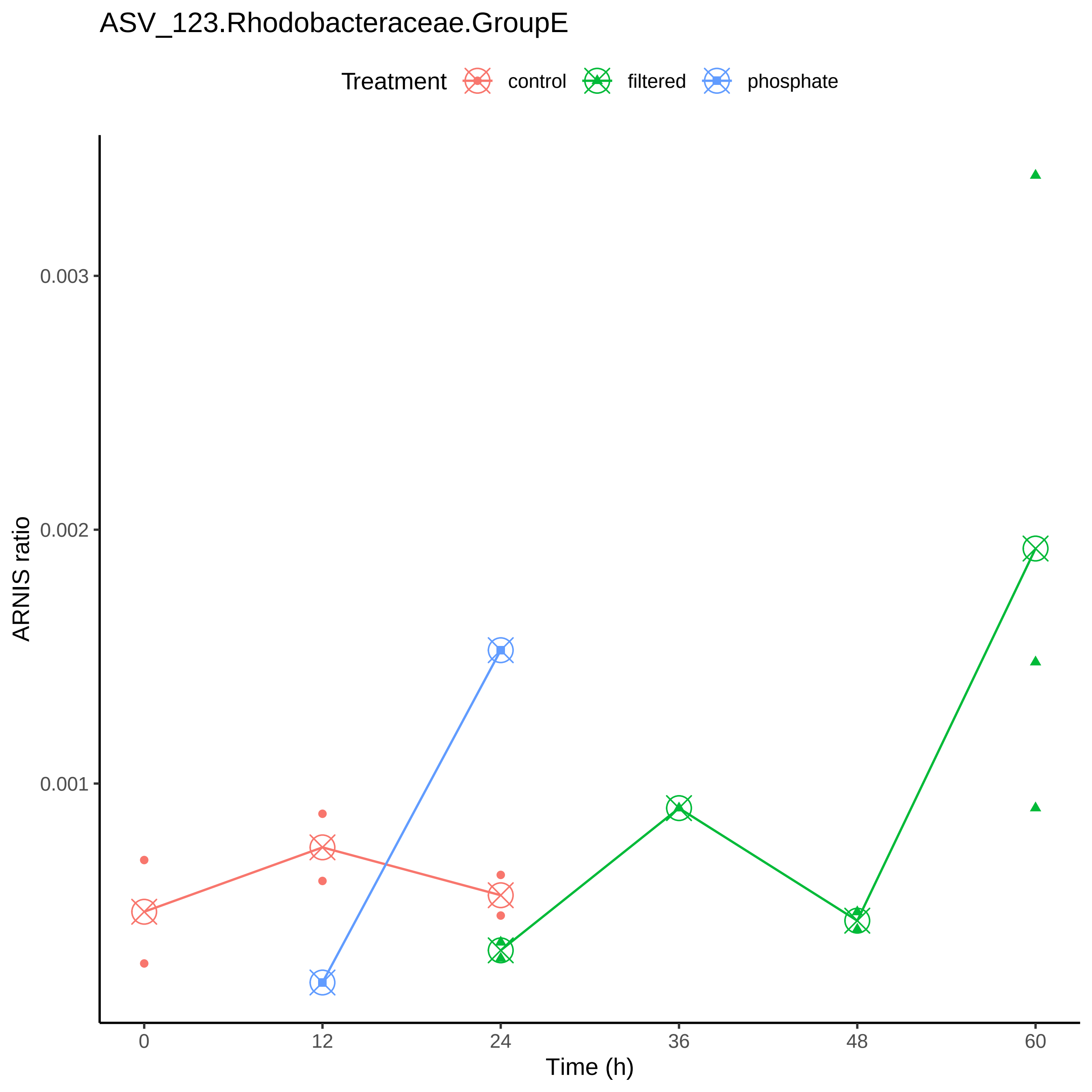
ASV_123.Rhodobacteraceae.GroupE
Treatment
control
filtered
phosphate
ARNIS ratio
0.003
0.002
0.001
Time (h)
0
12
24
36
48
60

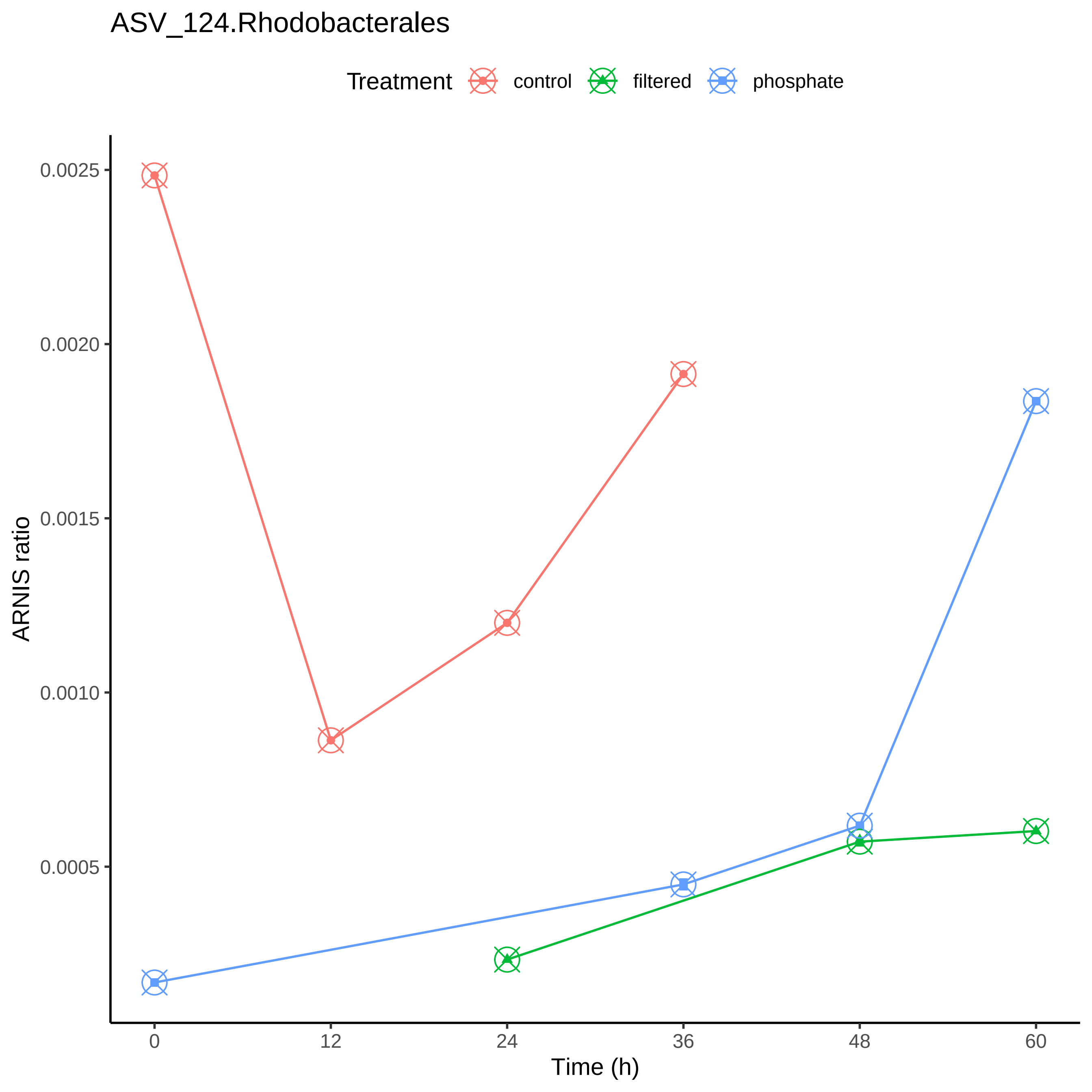
ASV_124.Rhodobacterales
Treatment
control
filtered
phosphate
ARNIS ratio
0.0025
0.0020
0.0015
0.0010
0.0005
0
12
24
36
48
60
Time (h)

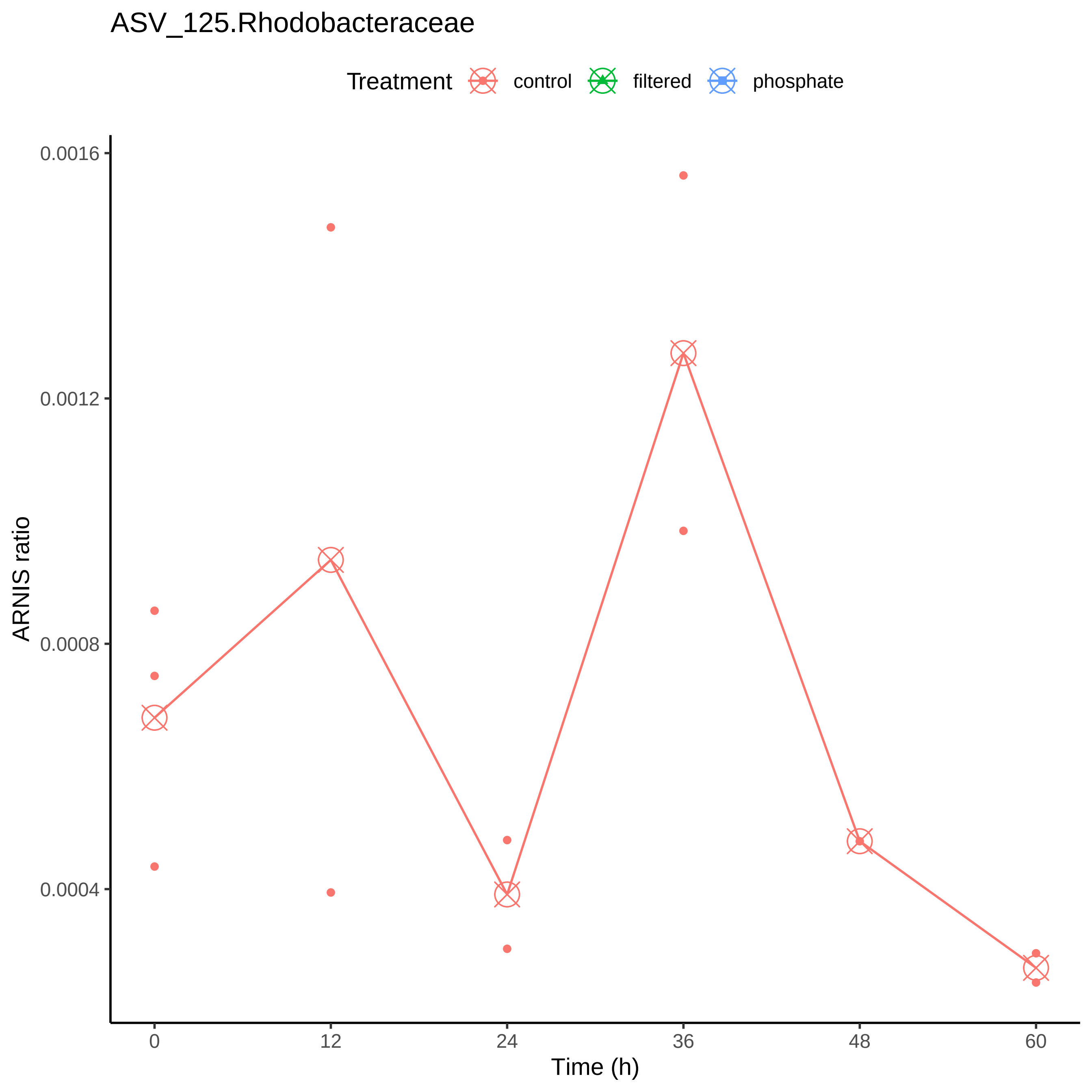

ASV_125.Rhodobacteraceae
Treatment
control
filtered
phosphate
0.0016
0.0012
0.0008
0.0004
ARNIS ratio
0
12
24
36
48
60
Time (h)

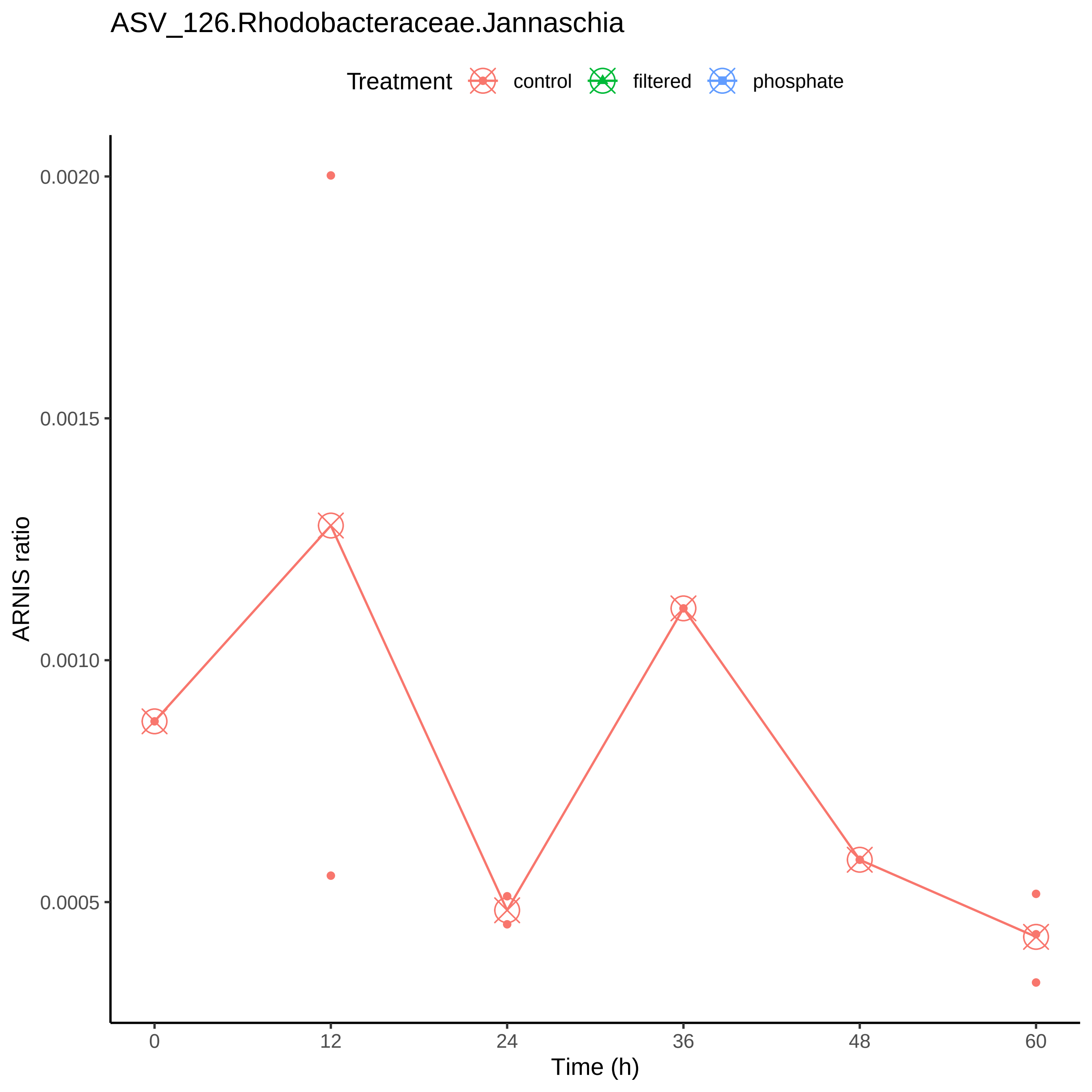

ASV_126.Rhodobacteraceae.Jannaschia
Treatment
control
filtered
phosphate
ARNIS ratio
0.0020
0.0015
0.0010
0.0005
0
12
24
36
48
60
Time (h)

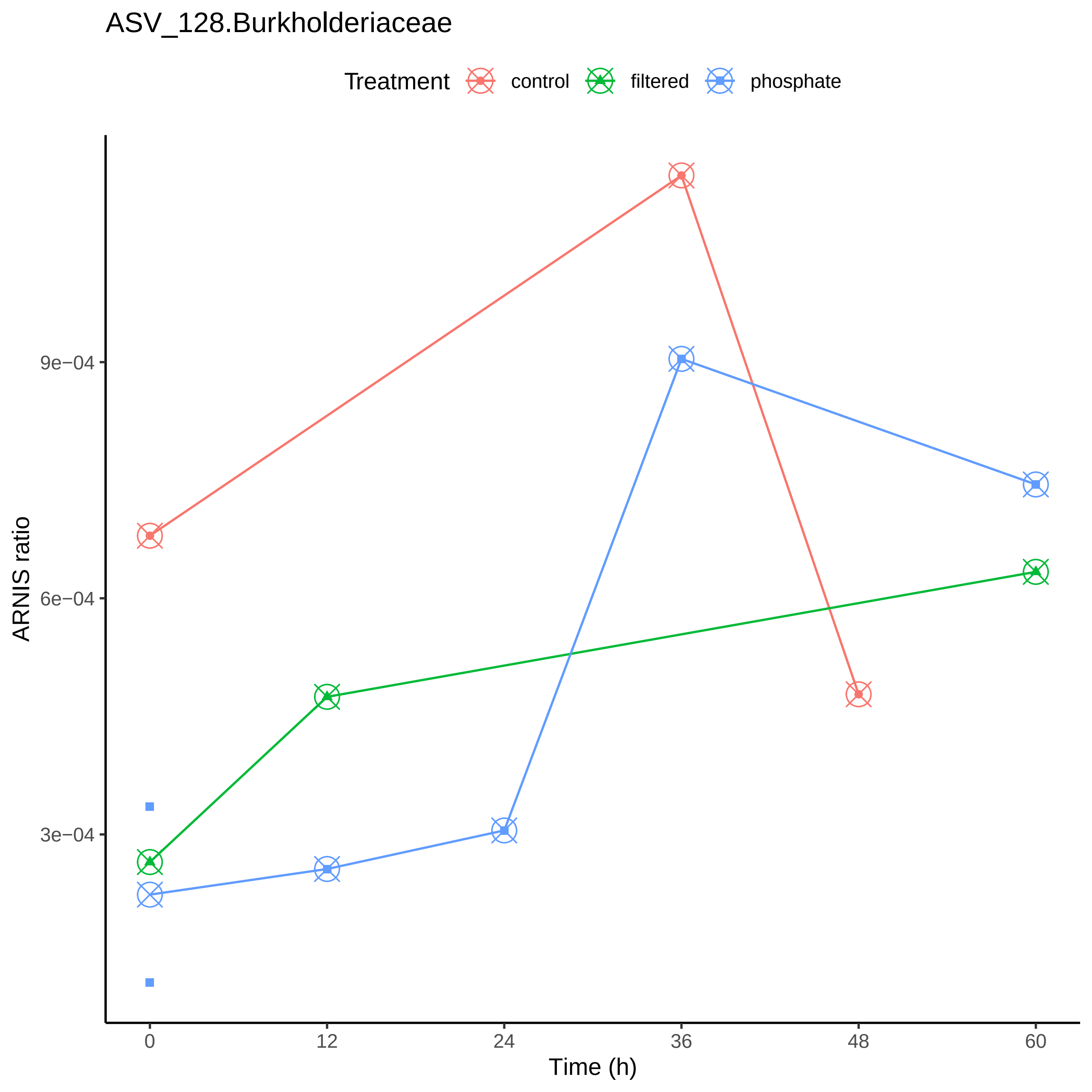

ASV_128.Burkholderiaceae
Treatment
control
filtered
phosphate
ARNIS ratio
9e−04
6e−04
3e−04
0
12
24
36
48
60
Time (h)

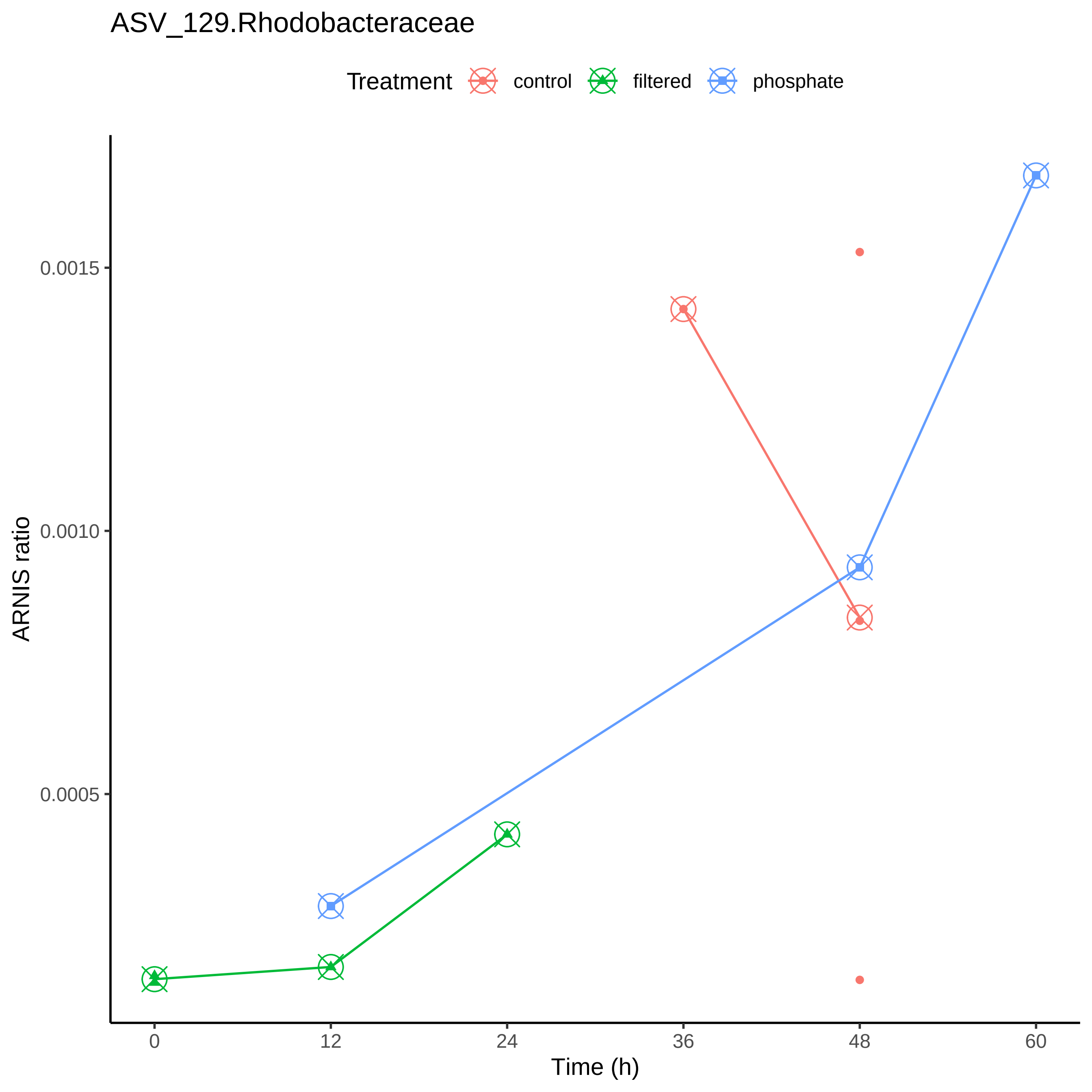

ASV_129.Rhodobacteraceae
Treatment
control
filtered
phosphate
ARNIS ratio
0.0015
0.0010
0.0005
0
12
24
36
48
60
Time (h)

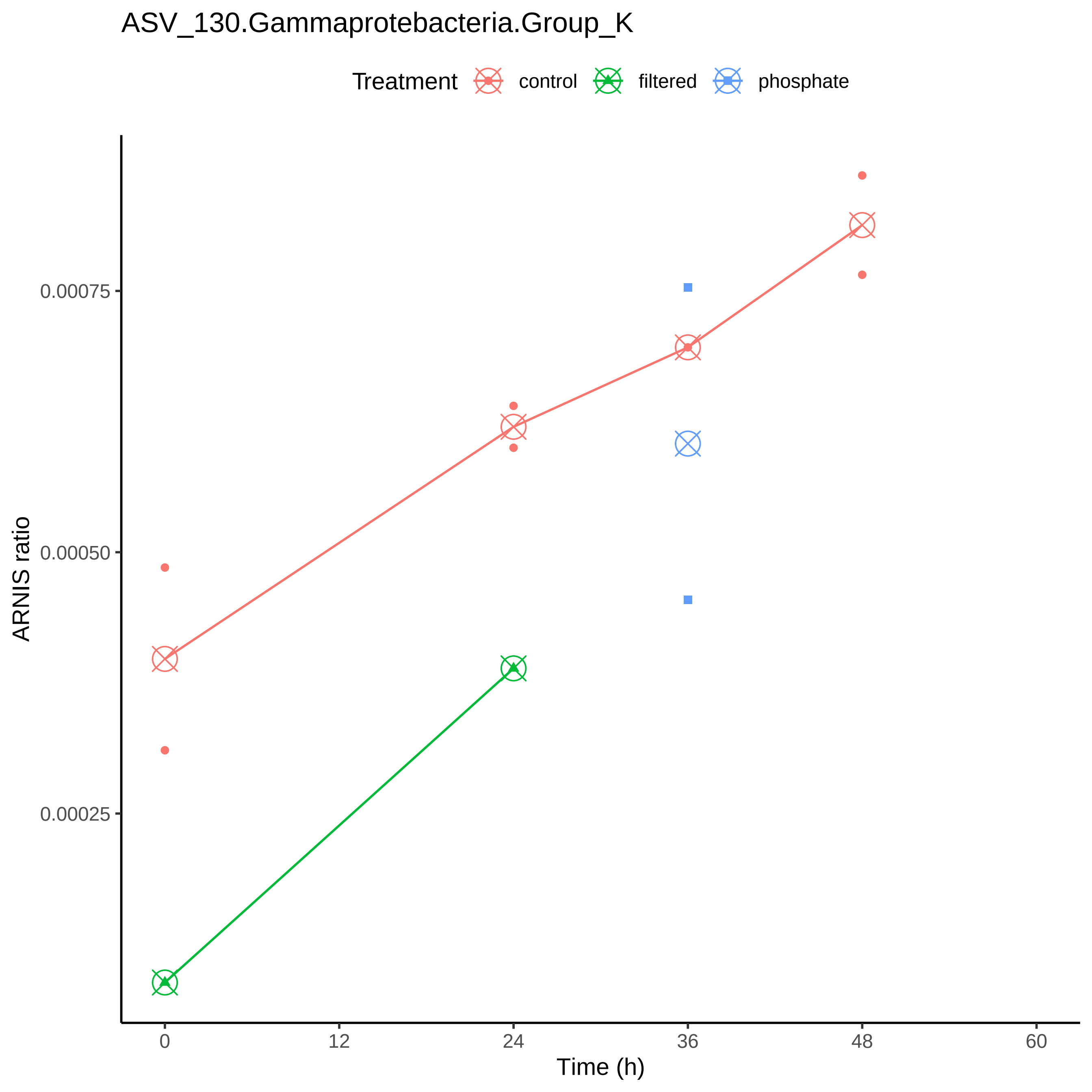

ASV_130.Gammaprotebacteria.Group_K
Treatment
control
filtered
phosphate
ARNIS ratio
0.00075
0.00050
0.00025
0
12
24
36
48
60
Time (h)
