# Supplementary material for: Lineage-Specific Growth Curves Document Large Differences in Response of Individual Groups of Marine Bacteria to the Top-Down and Bottom-Up Controls
Source: mSystems. 2021 Sep 28;6(5):e00934-21. doi: 10.1128/mSystems.00934-21 (PMC8547455; doi:10.1128/mSystems.00934-21)
Supplement: FIG S4 [file msystems.00934-21-sf004.pdf]

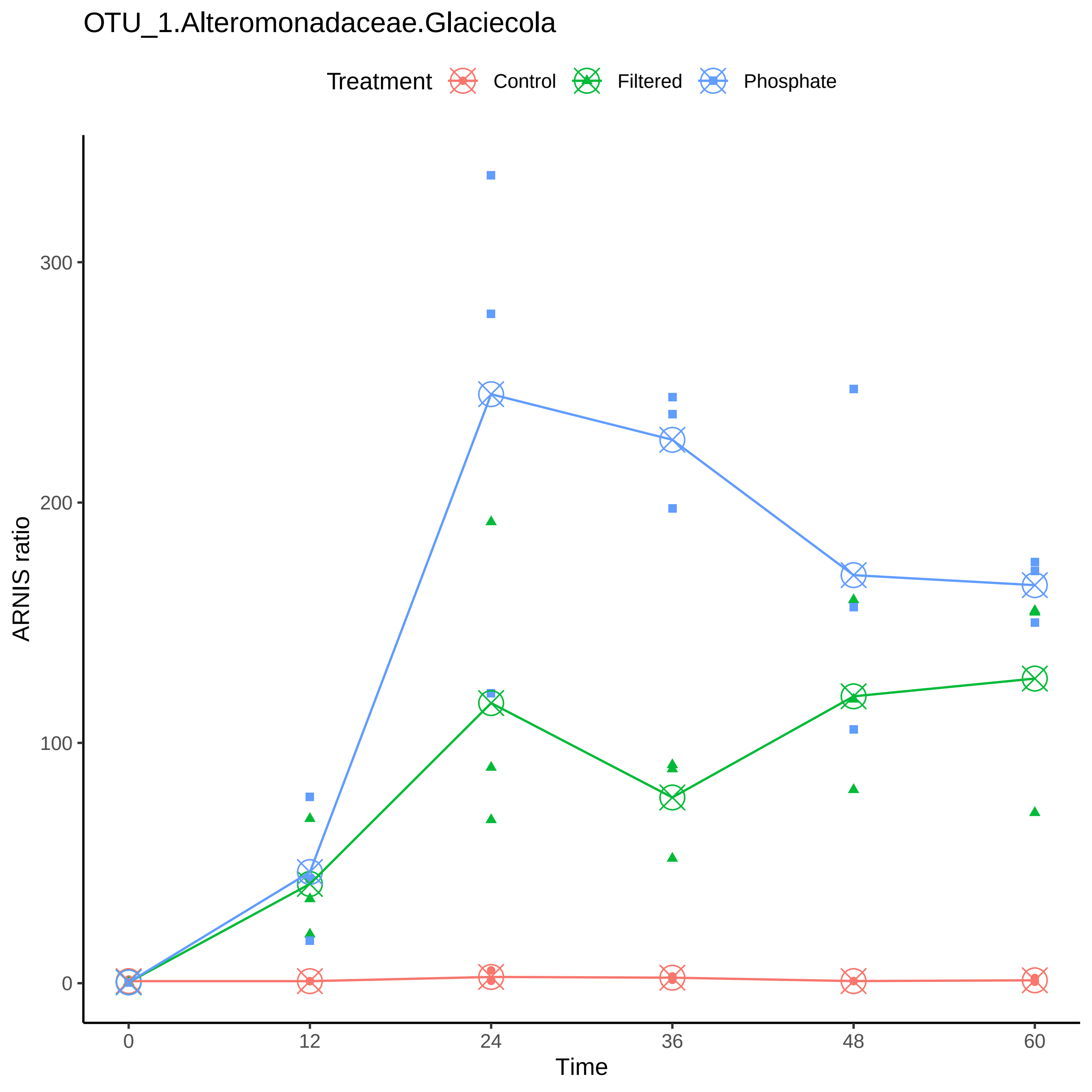

OTU_1.Alteromonadaceae.Glaciecola
Treatment
Control
Filtered
Phosphate
ARNIS ratio
0
100
200
300
Time
0
12
24
36
48
60

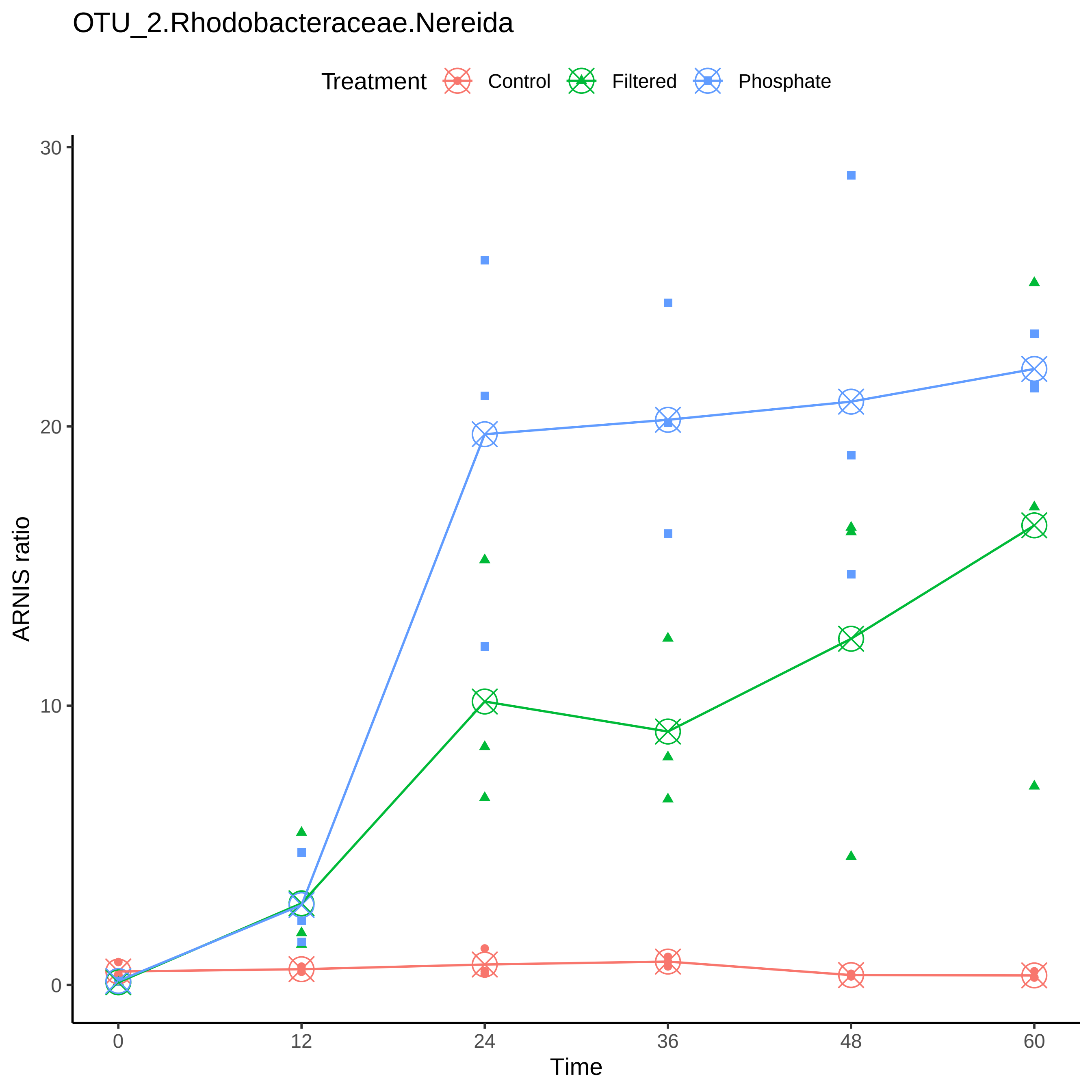
OTU_2.Rhodobacteraceae.Nereida
Treatment
Control
Filtered
Phosphate
ARNIS ratio
30
20
10
0
0
12
24
36
48
60
Time

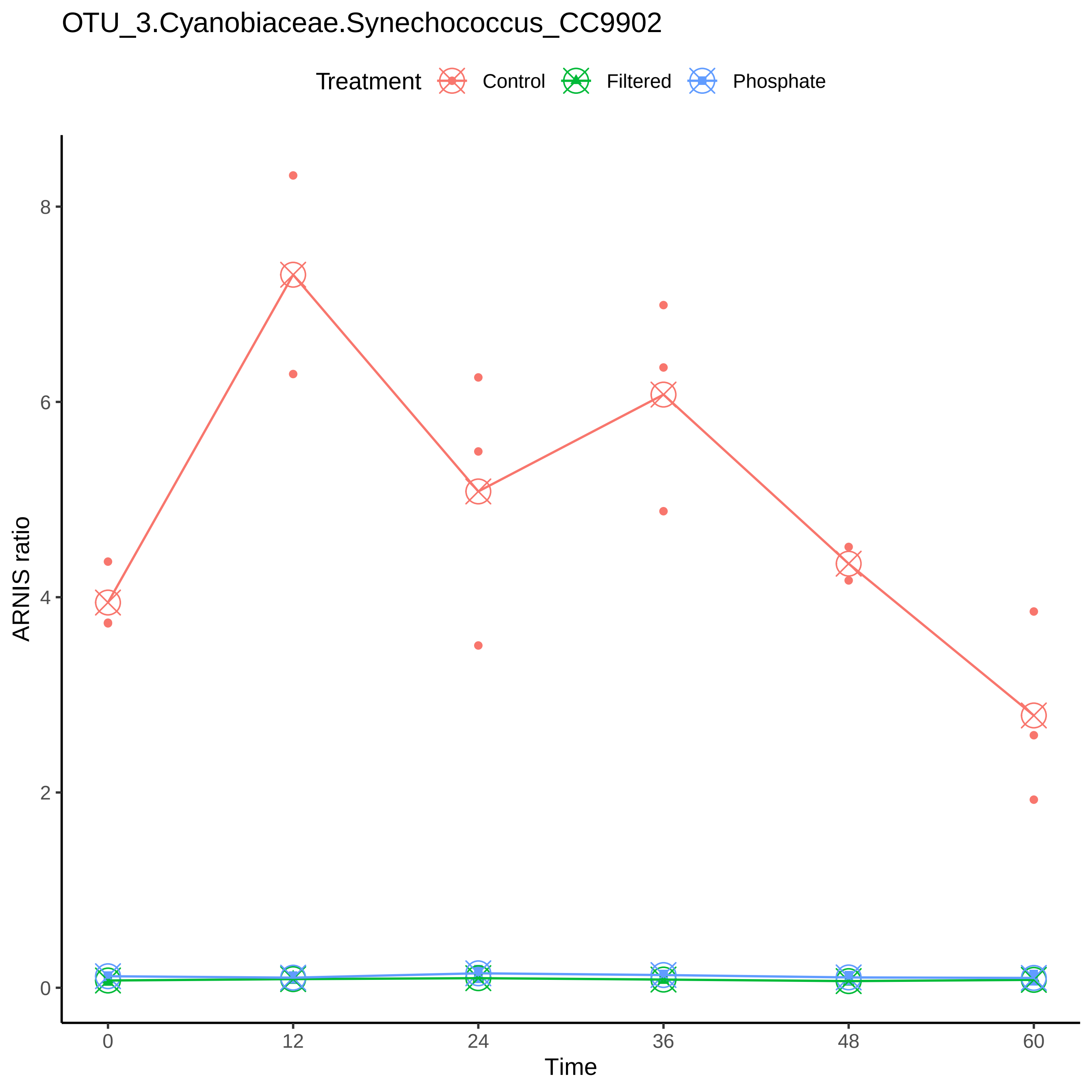

OTU_3.Cyanobiaceae.Synechococcus_CC9902
Treatment
Control
Filtered
Phosphate
ARNIS ratio
0
2
4
6
8
Time
0
12
24
36
48
60

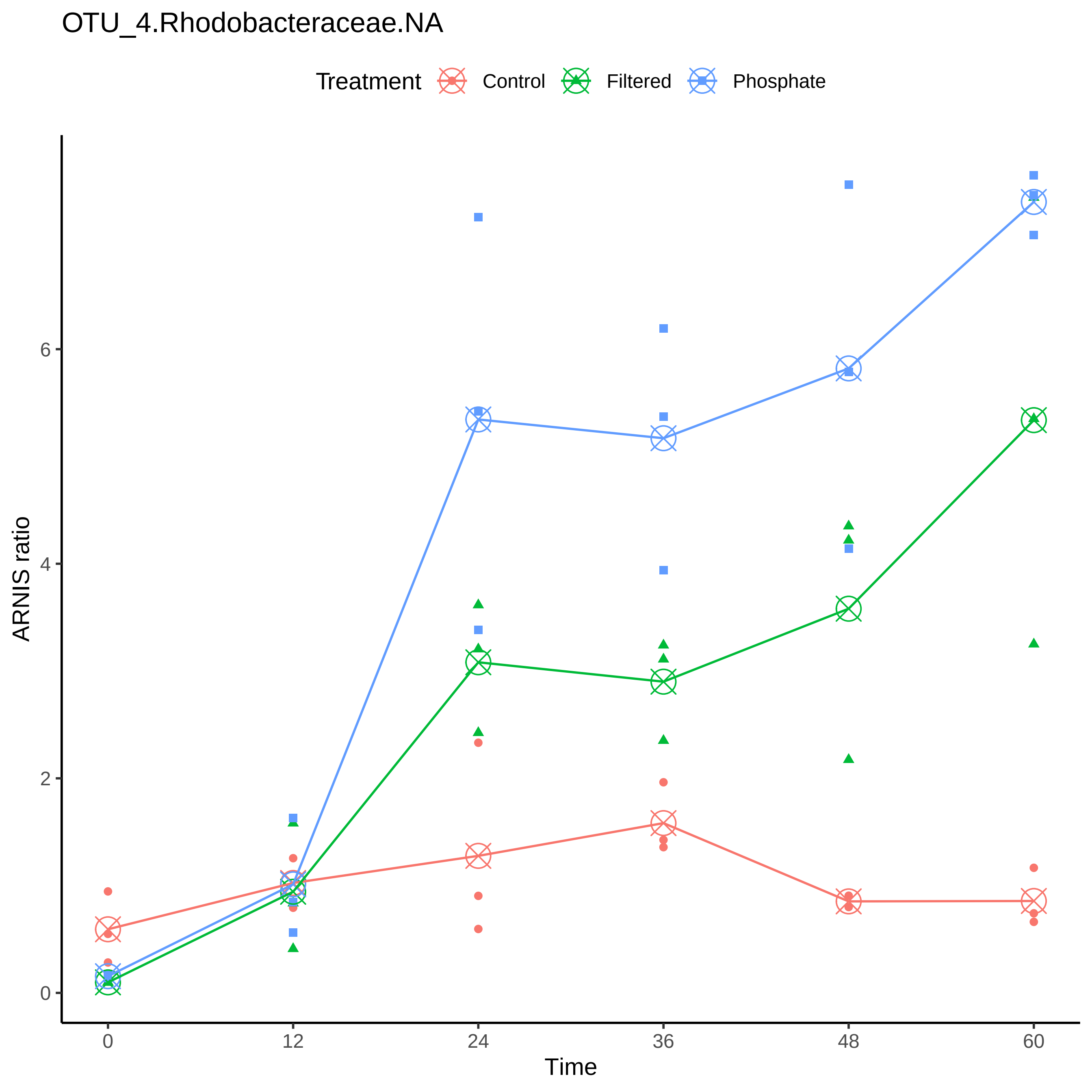

OTU_4.Rhodobacteraceae.NA
Treatment
Control
Filtered
Phosphate
ARNIS ratio
0
2
4
6
Time
0
12
24
36
48
60

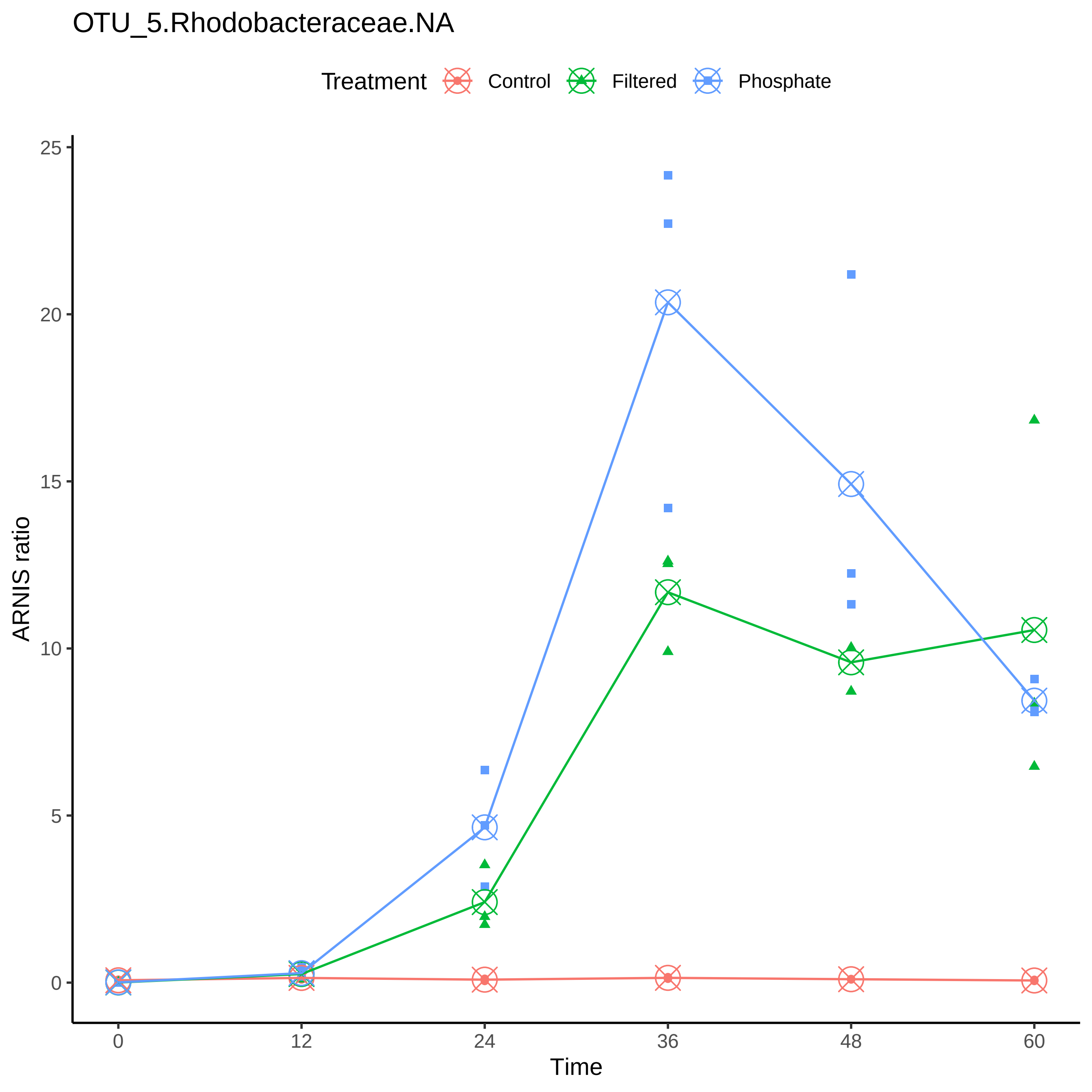
OTU_5.Rhodobacteraceae.NA
Treatment
Control
Filtered
Phosphate
ARNIS ratio
25
20
15
10
5
0
0
12
24
36
48
60
Time

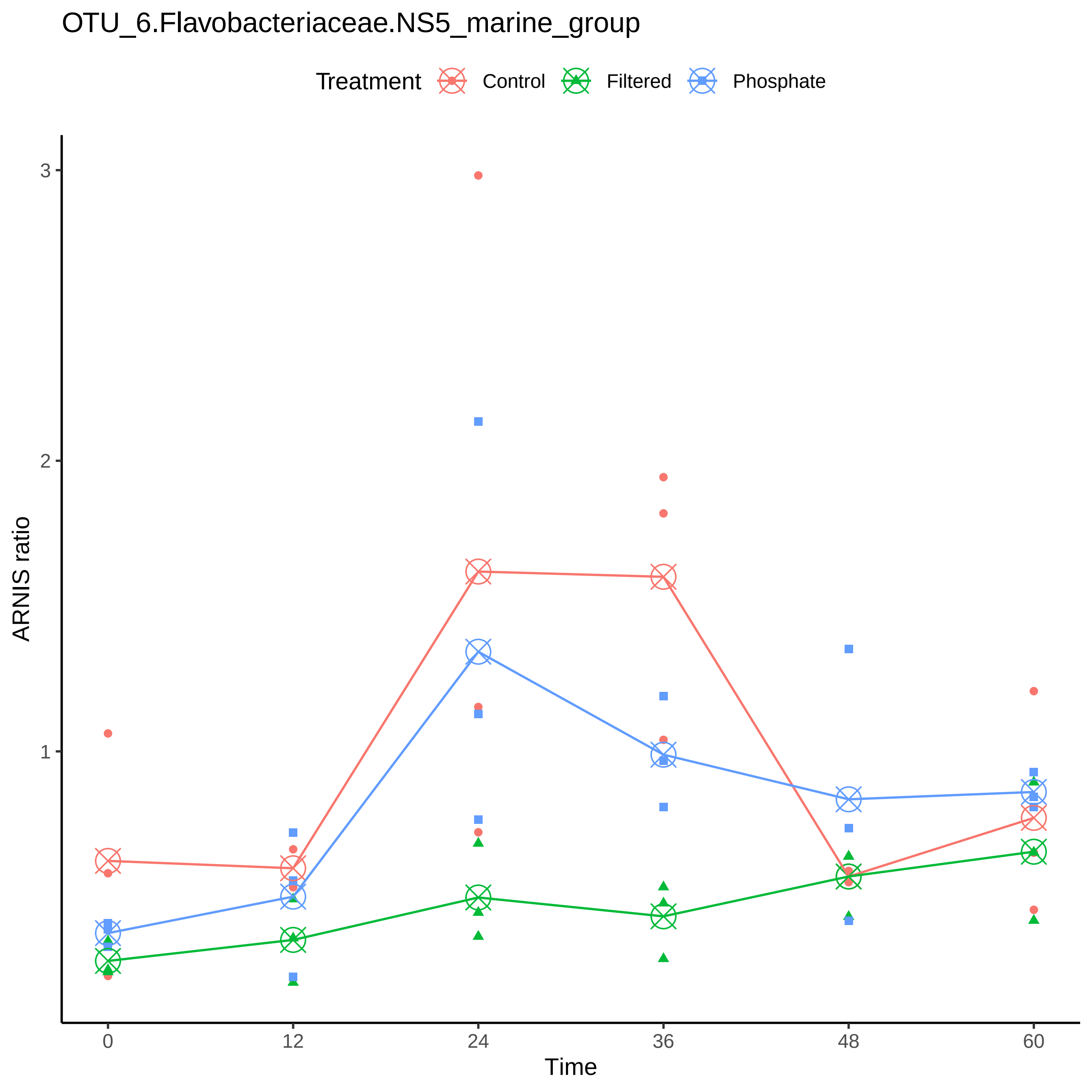

OTU_6.Flavobacteriaceae.NS5_marine_group
Treatment
Control
Filtered
Phosphate
ARNIS ratio
3
2
1
Time
0
12
24
36
48
60

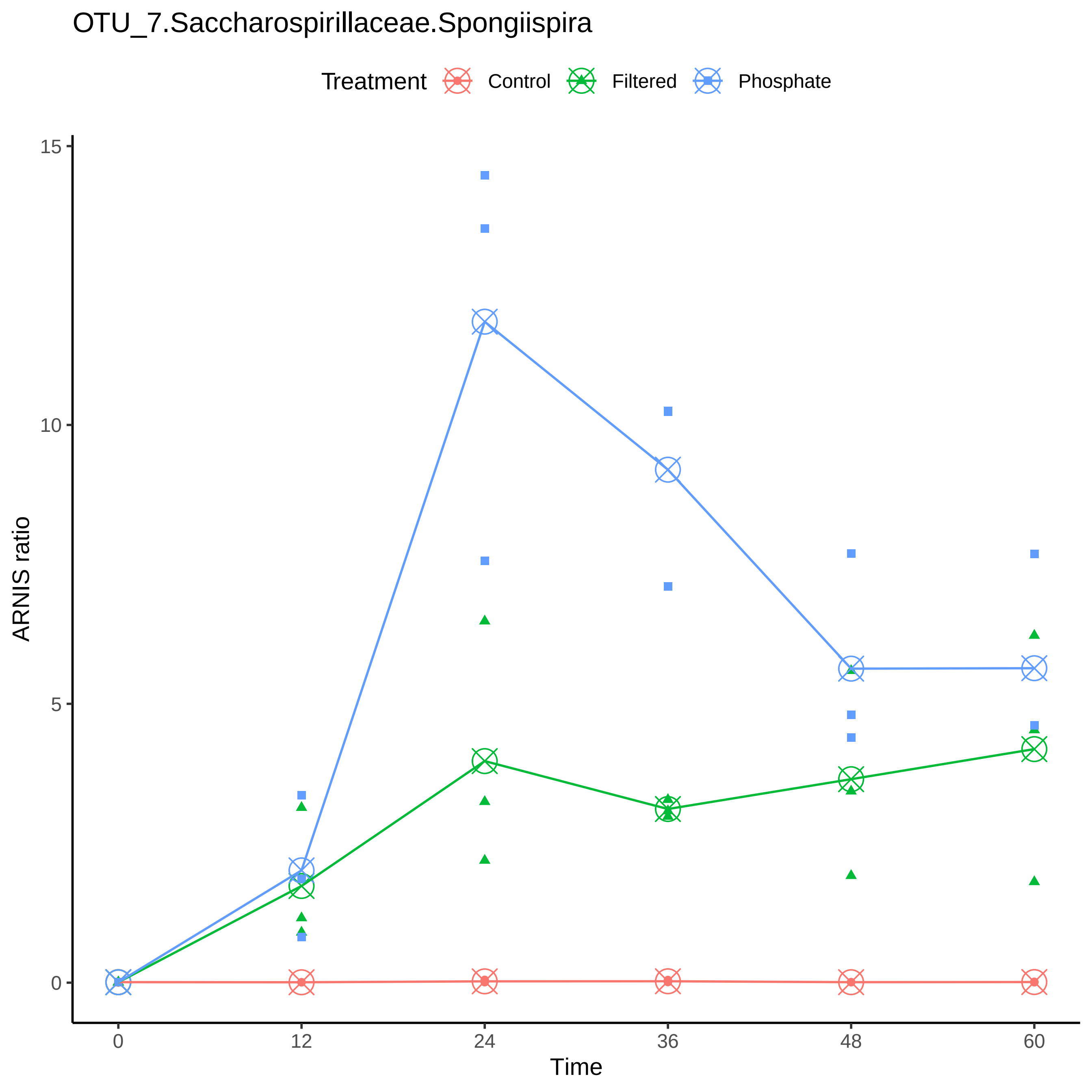

OTU_7.Saccharospirillaceae.Spongiispira
Treatment
Control
Filtered
Phosphate
ARNIS ratio
15
10
5
0
0
12
24
36
48
60
Time

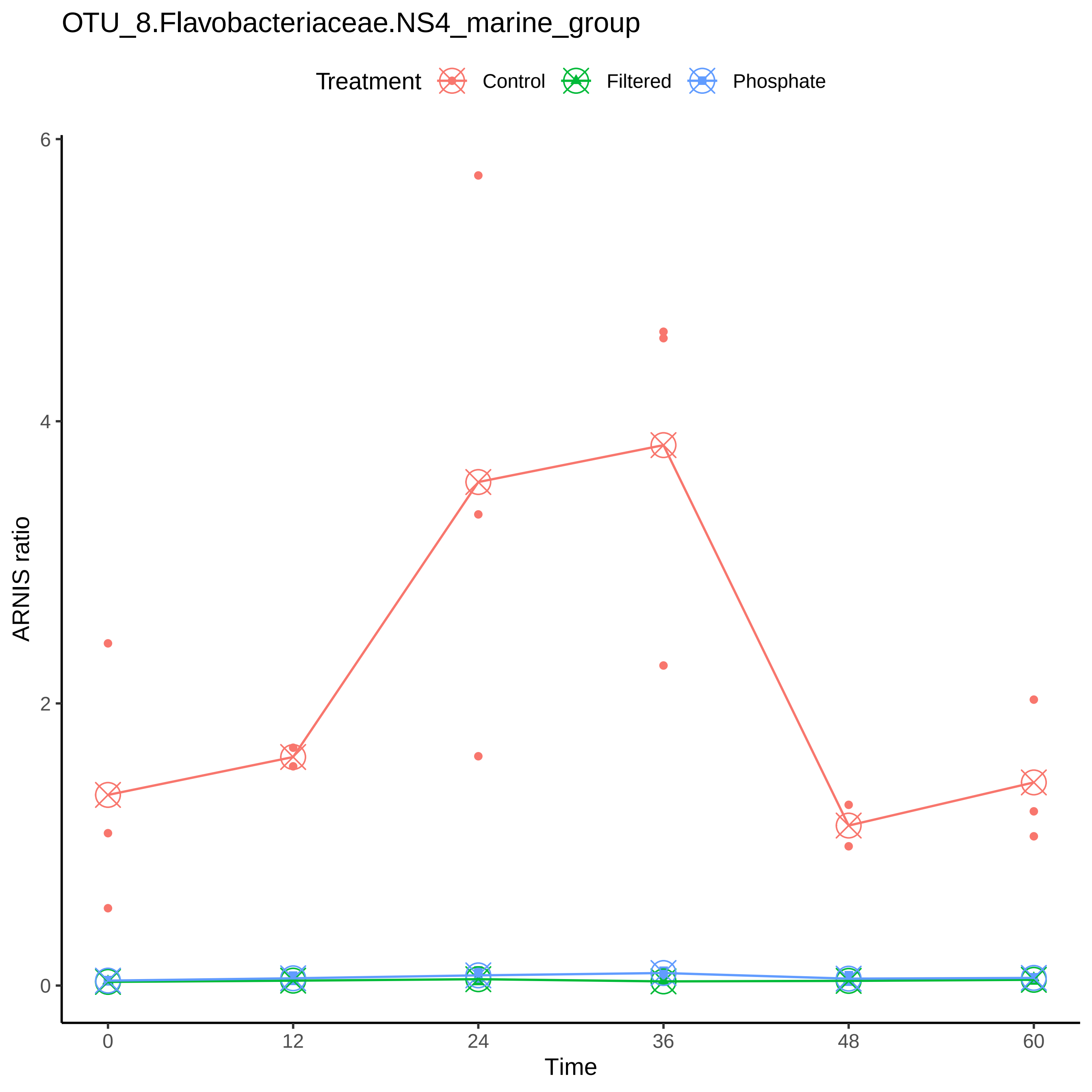
OTU_8.Flavobacteriaceae.NS4_marine_group
Treatment
Control
Filtered
Phosphate
ARNIS ratio
6
4
2
0
0
12
24
36
48
60
Time

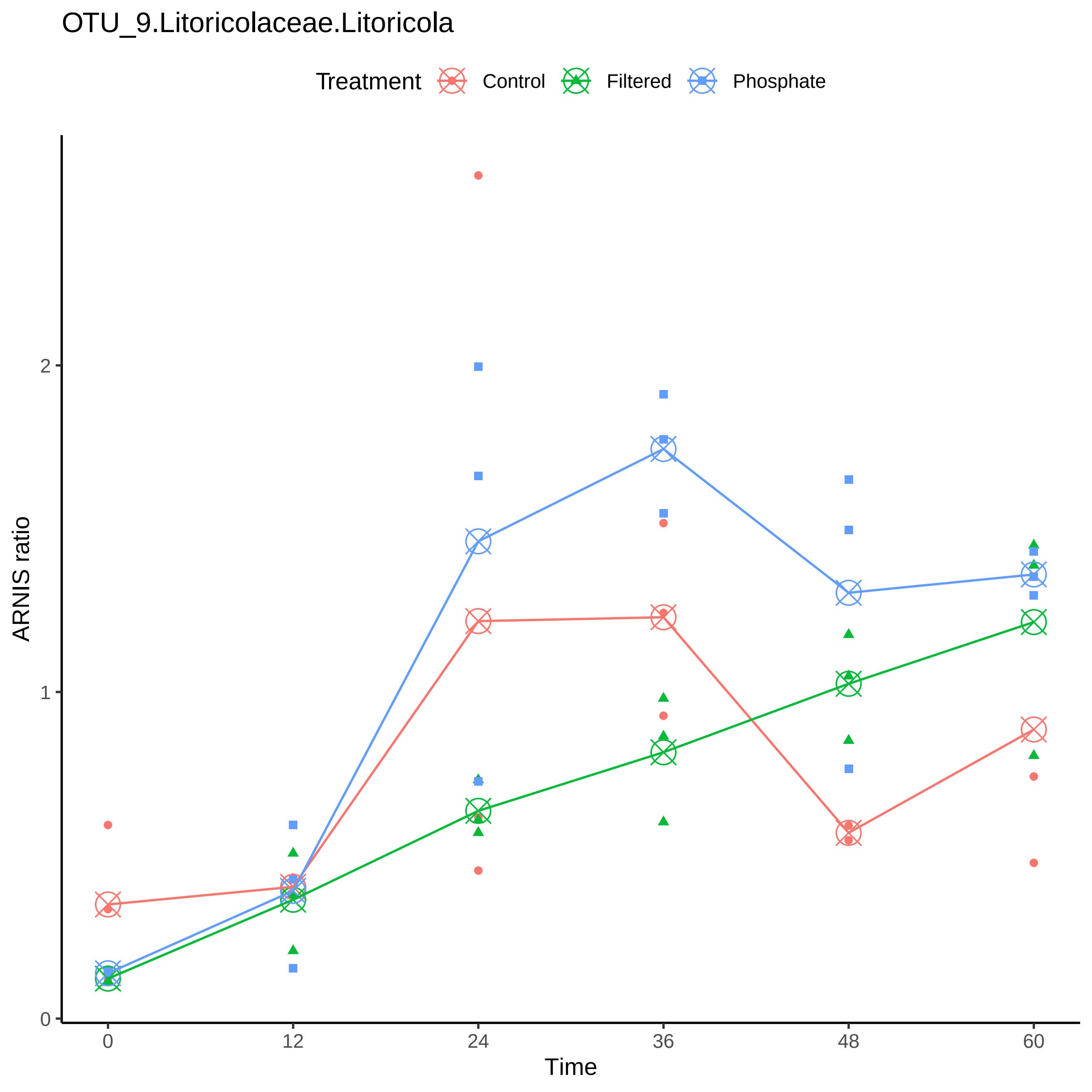

OTU_9.Litoricolaceae.Litoricola
Treatment
Control
Filtered
Phosphate
ARNIS ratio
0
1
2
0
12
24
36
48
60
Time

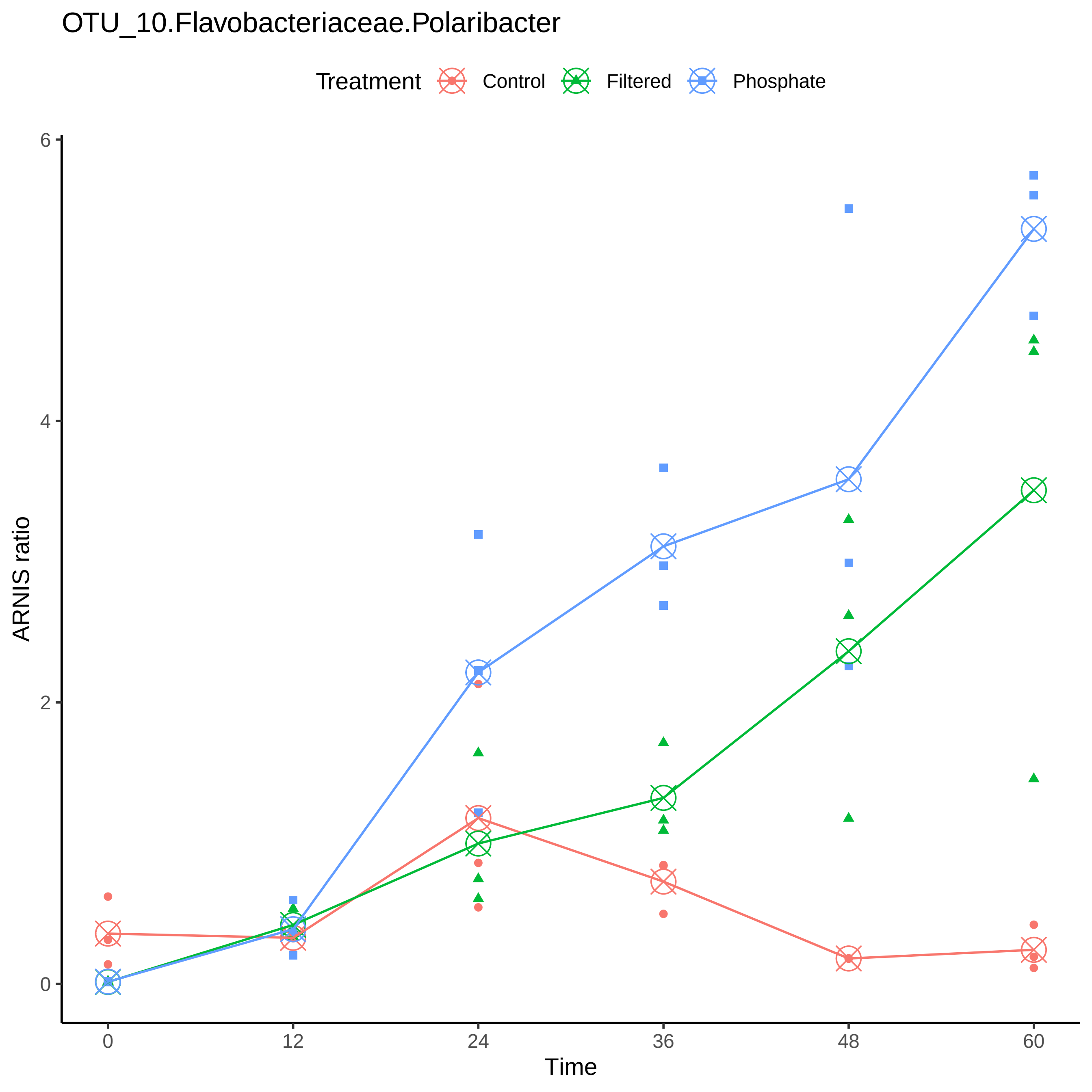
OTU_10.Flavobacteriaceae.Polaribacter
Treatment
Control
Filtered
Phosphate
ARNIS ratio
0
2
4
6
Time
0
12
24
36
48
60

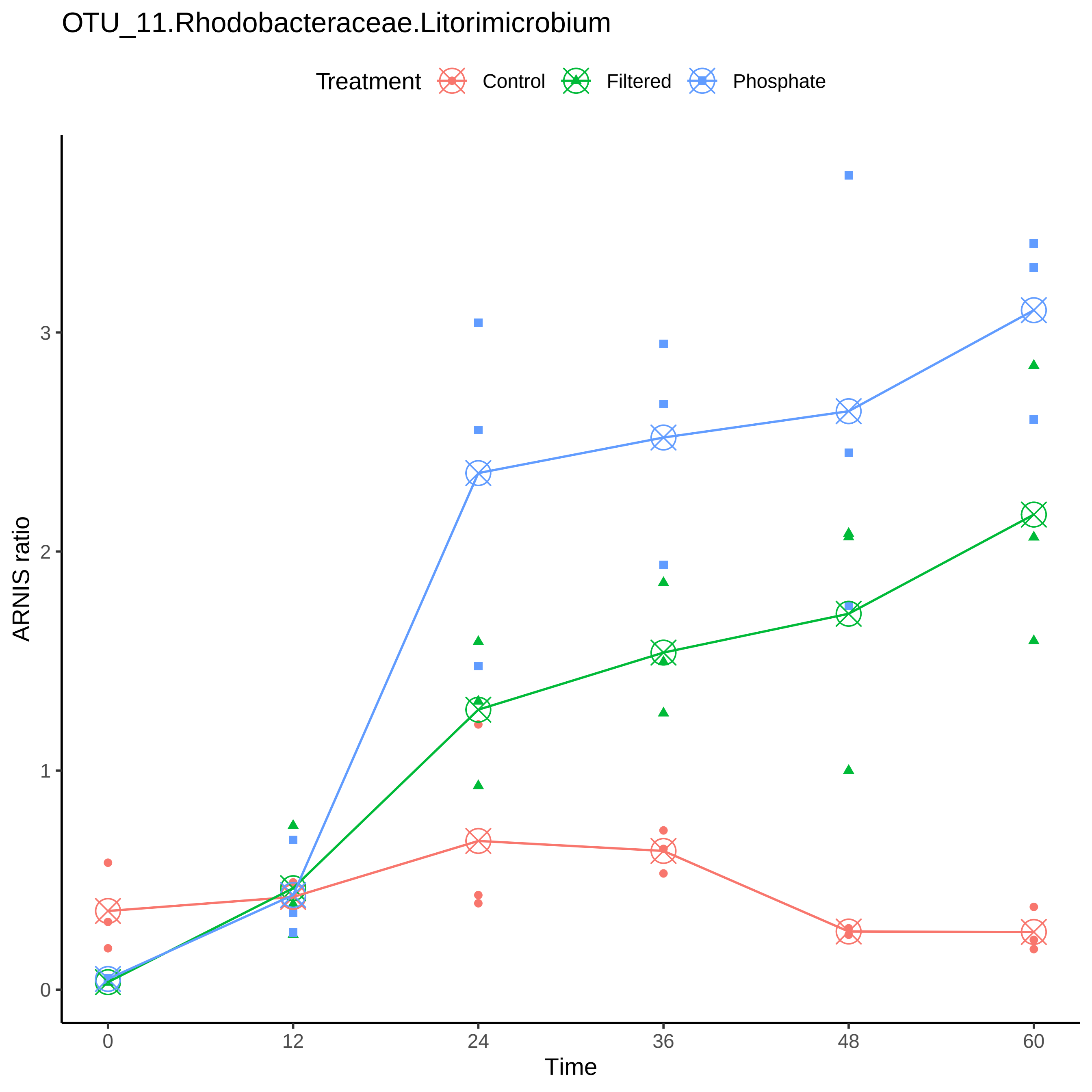
OTU_11.Rhodobacteraceae.Litorimicrobium
Treatment
Control
Filtered
Phosphate
ARNIS ratio
0
1
2
3
Time
0
12
24
36
48
60

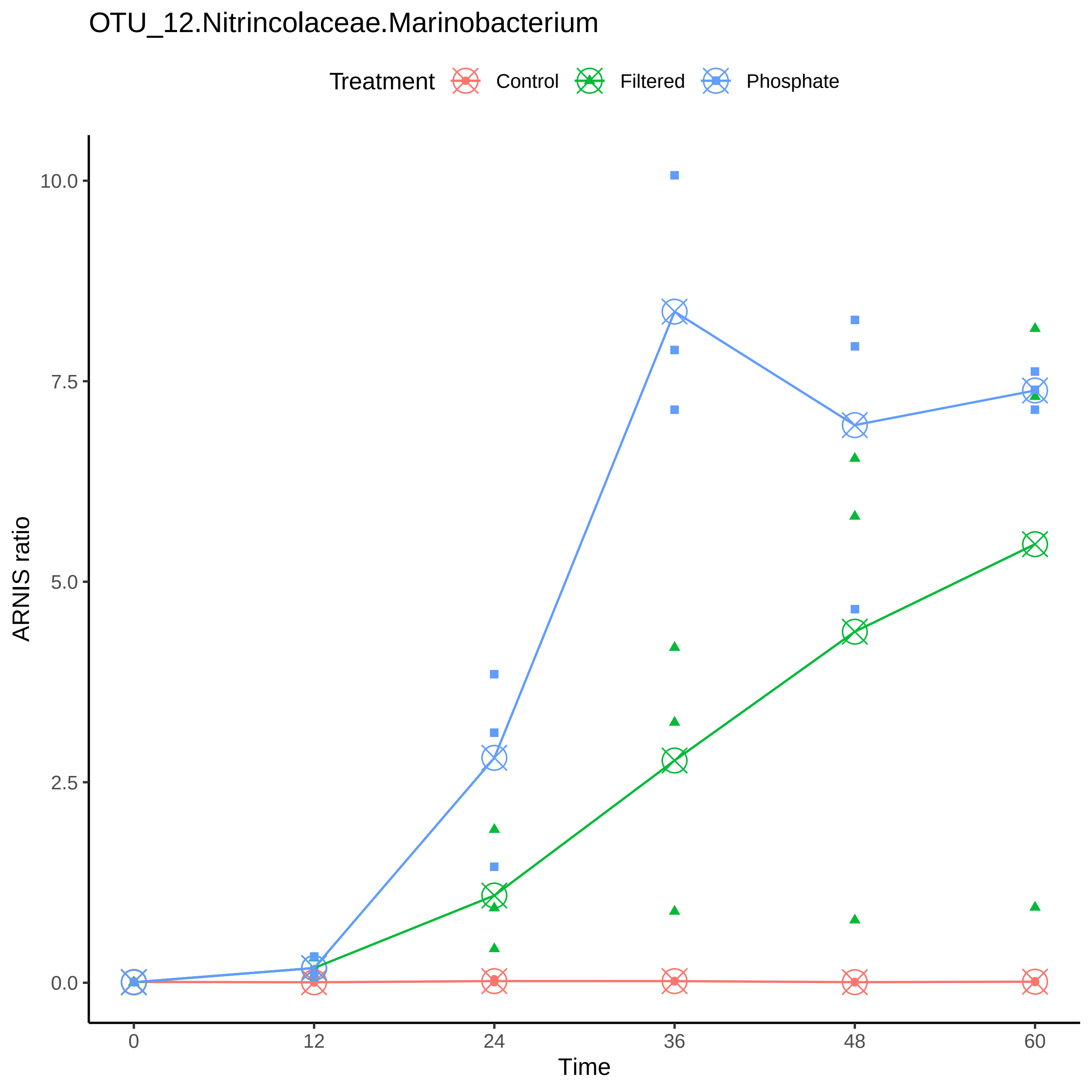
OTU_12.Nitrincolaceae.Marinobacterium
Treatment
Control
Filtered
Phosphate
ARNIS ratio
10.0
7.5
5.0
2.5
0.0
0
12
24
36
48
60
Time

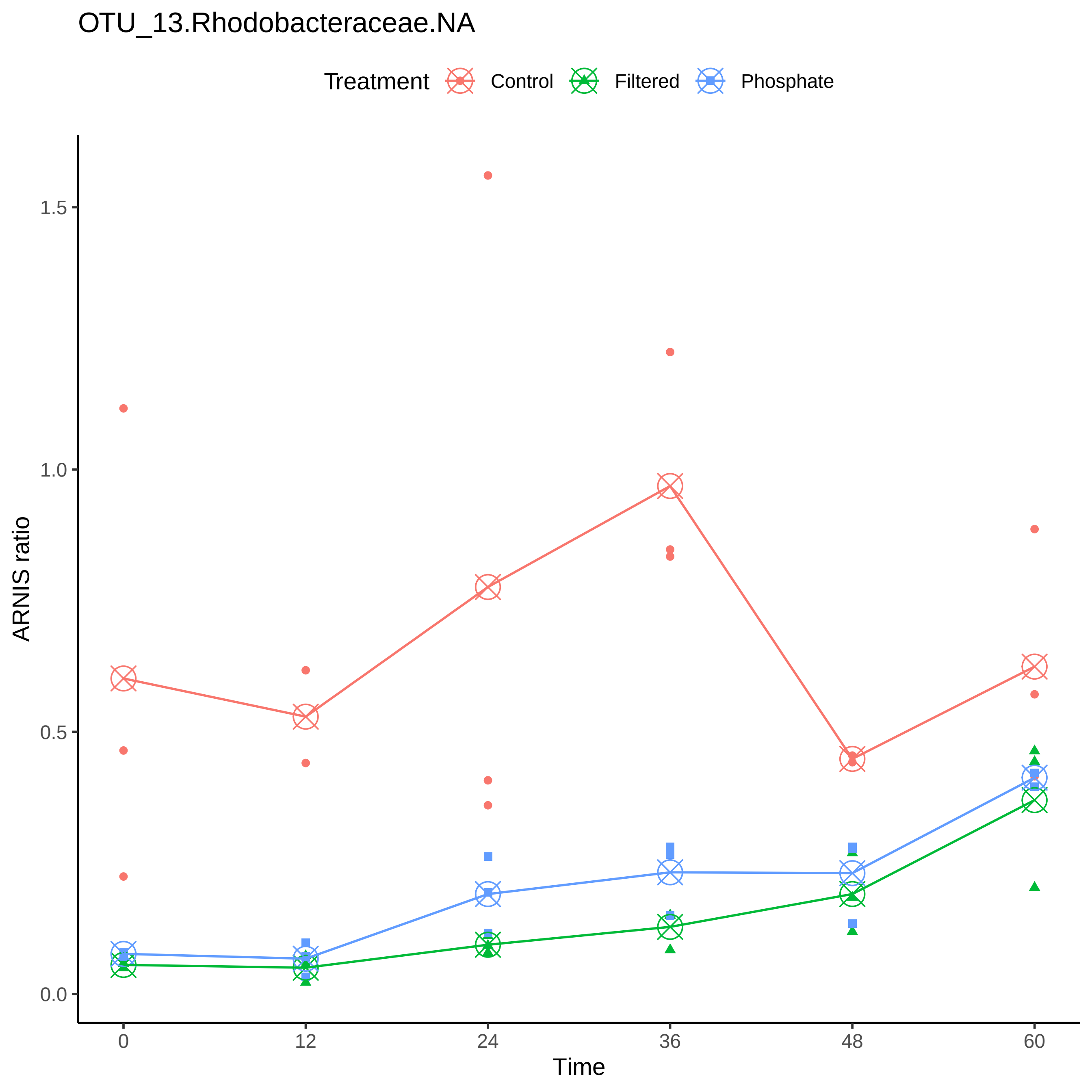

OTU_13.Rhodobacteraceae.NA
Treatment
Control
Filtered
Phosphate
ARNIS ratio
1.5
1.0
0.5
0.0
0
12
24
36
48
60
Time

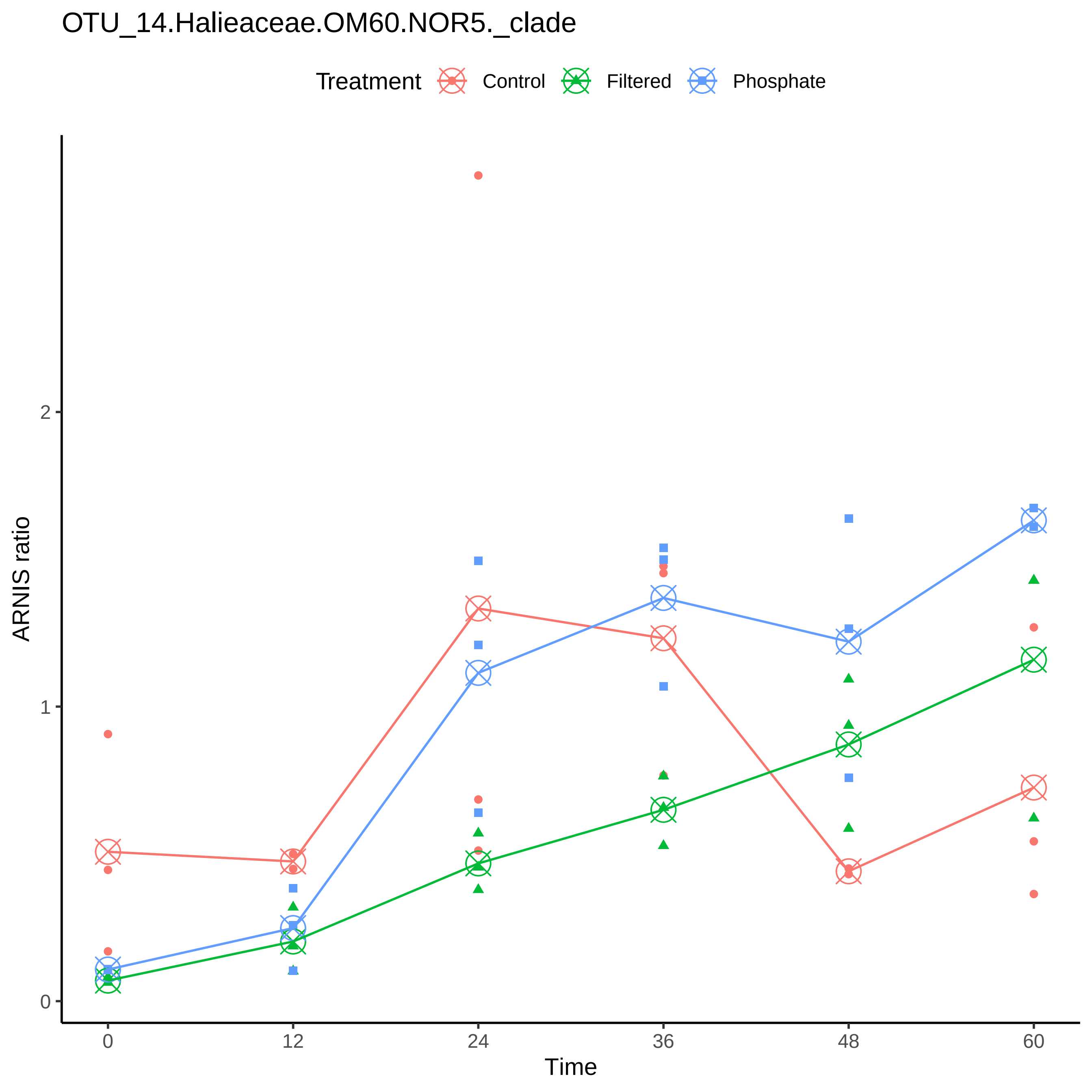
OTU_14.Halieaceae.OM60.NOR5._clade
Treatment
Control
Filtered
Phosphate
ARNIS ratio
0
1
2
0
12
24
36
48
60
Time

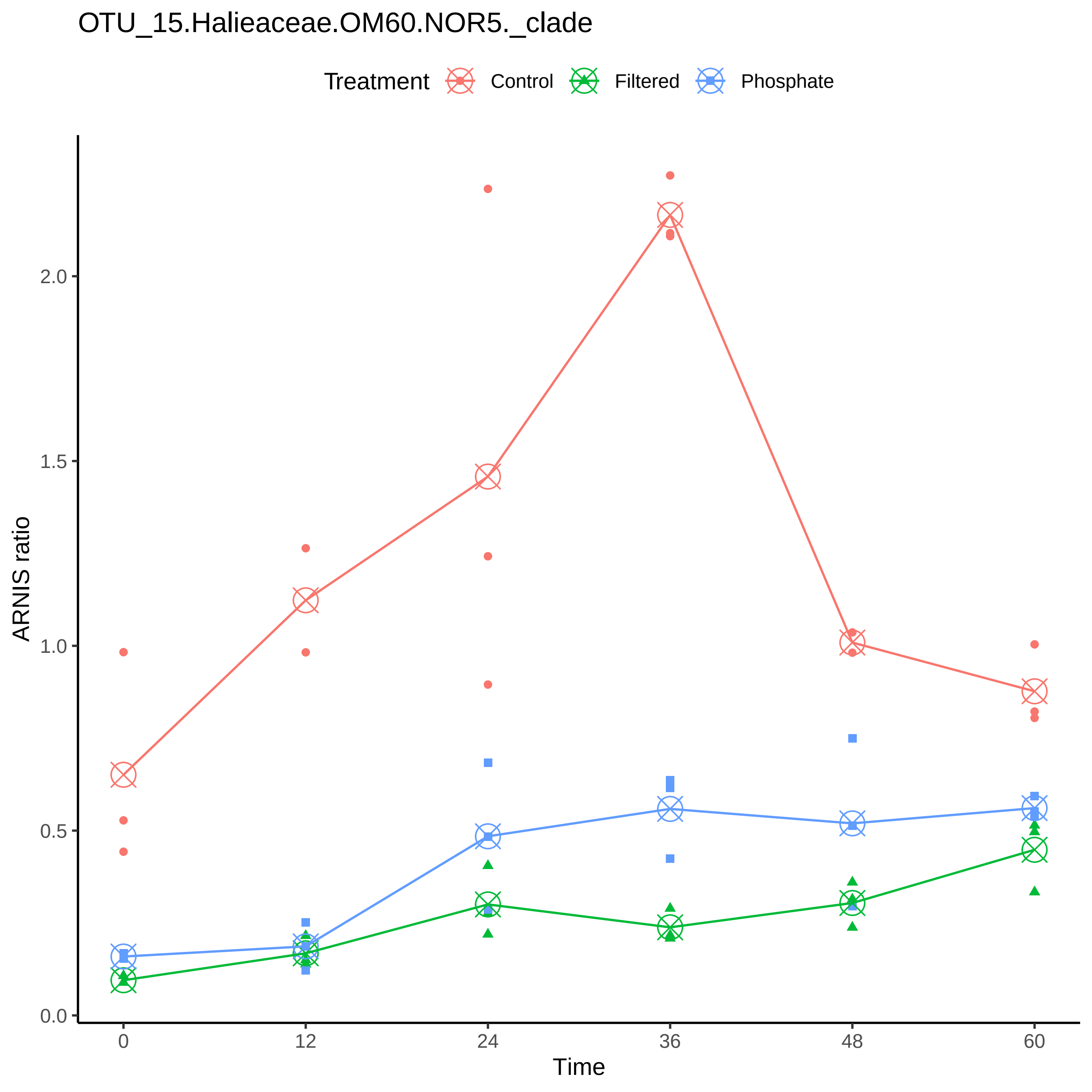

OTU_15.Halieaceae.OM60.NOR5._clade
Treatment
Control
Filtered
Phosphate
ARNIS ratio
0.0
0.5
1.0
1.5
2.0
Time
0
12
24
36
48
60

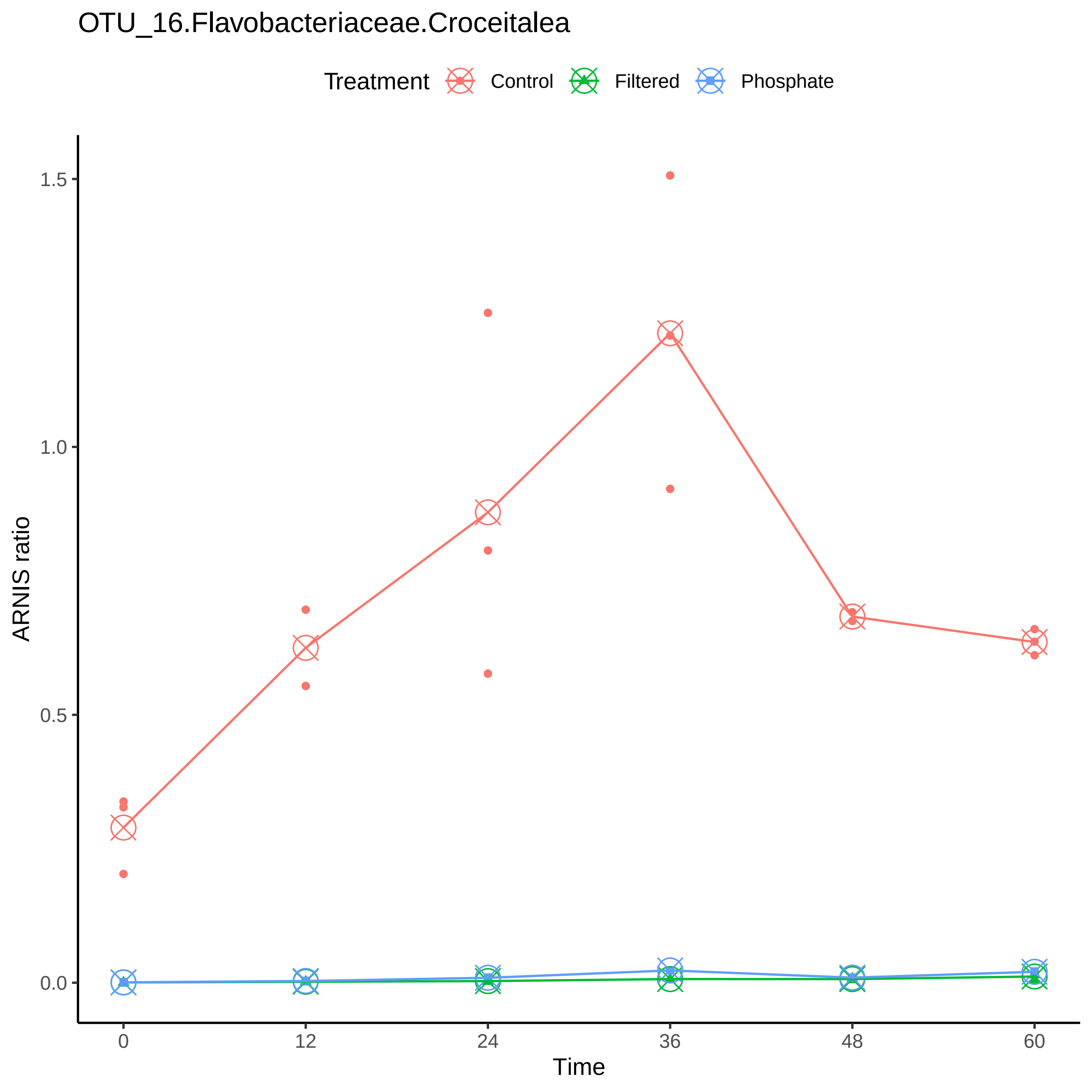
OTU_16.Flavobacteriaceae.Croceitalea
Treatment
Control
Filtered
Phosphate
ARNIS ratio
1.5
1.0
0.5
0.0
0
12
24
36
48
60
Time

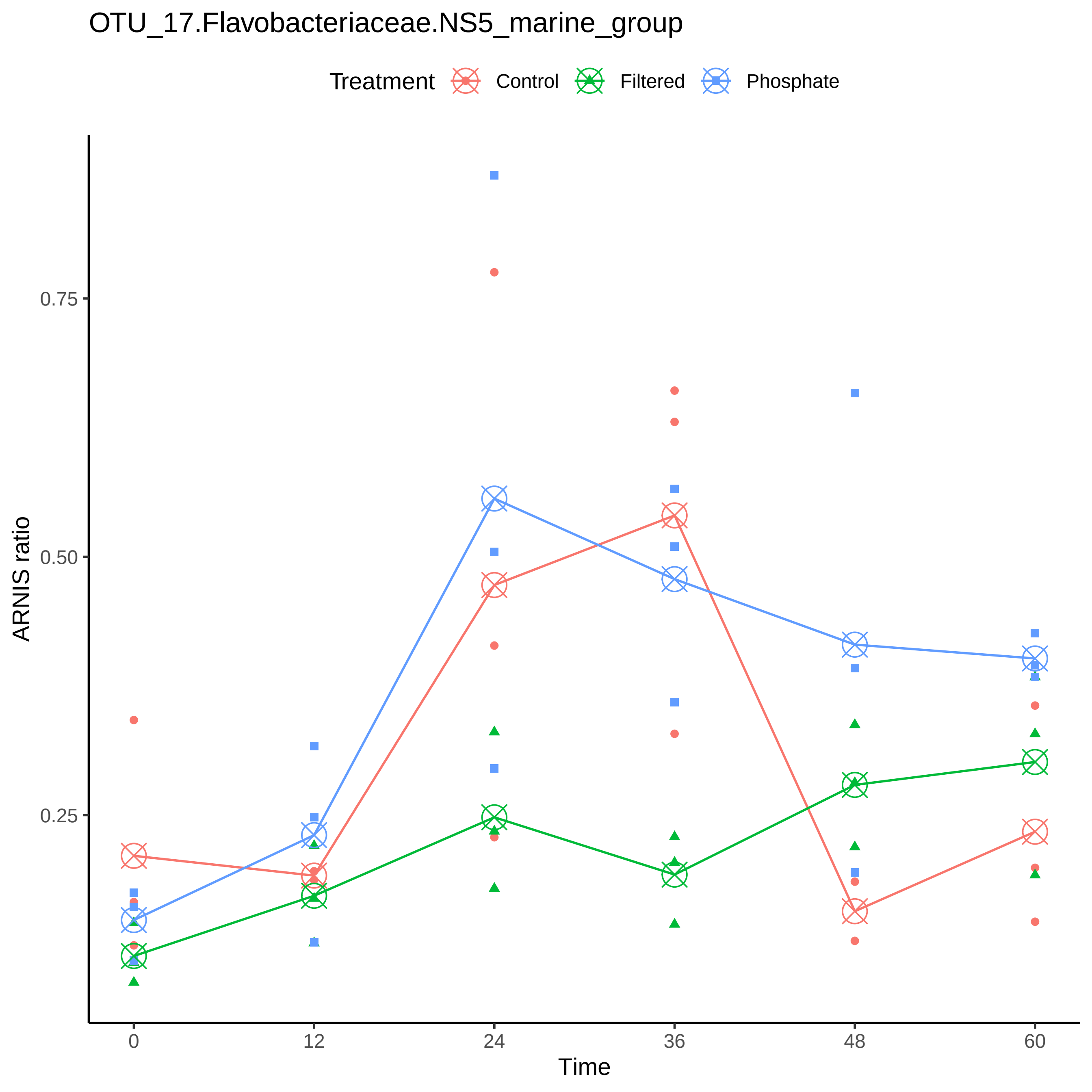

OTU_17.Flavobacteriaceae.NS5_marine_group
Treatment
Control
Filtered
Phosphate
ARNIS ratio
0.75
0.50
0.25
0
12
24
36
48
60
Time

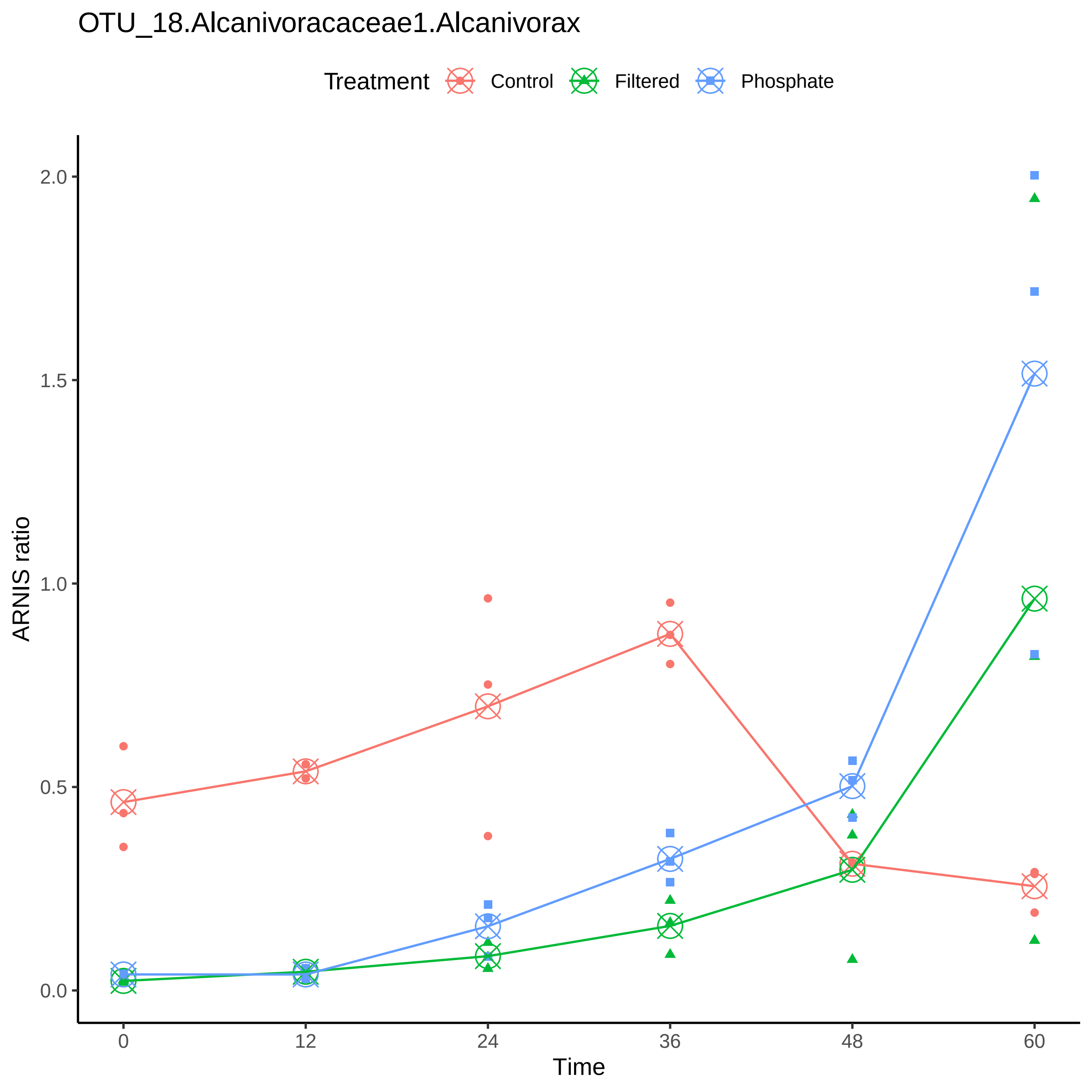

OTU_18.Alcanivoracaceae1.Alcanivorax
Treatment
Control
Filtered
Phosphate
ARNIS ratio
2.0
1.5
1.0
0.5
0.0
0
12
24
36
48
60
Time

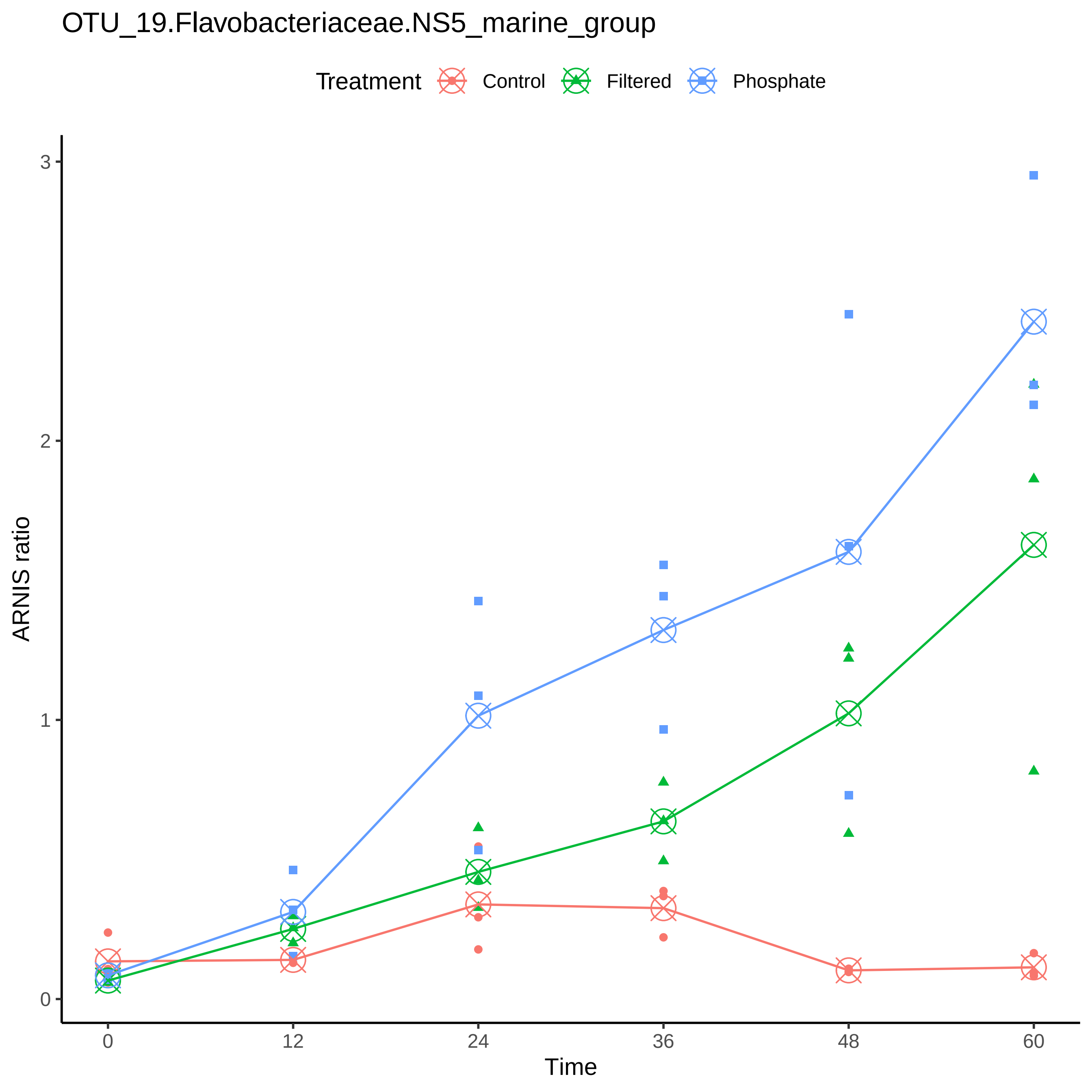

OTU_19.Flavobacteriaceae.NS5_marine_group
Treatment
Control
Filtered
Phosphate
ARNIS ratio
0
1
2
3
Time
0
12
24
36
48
60

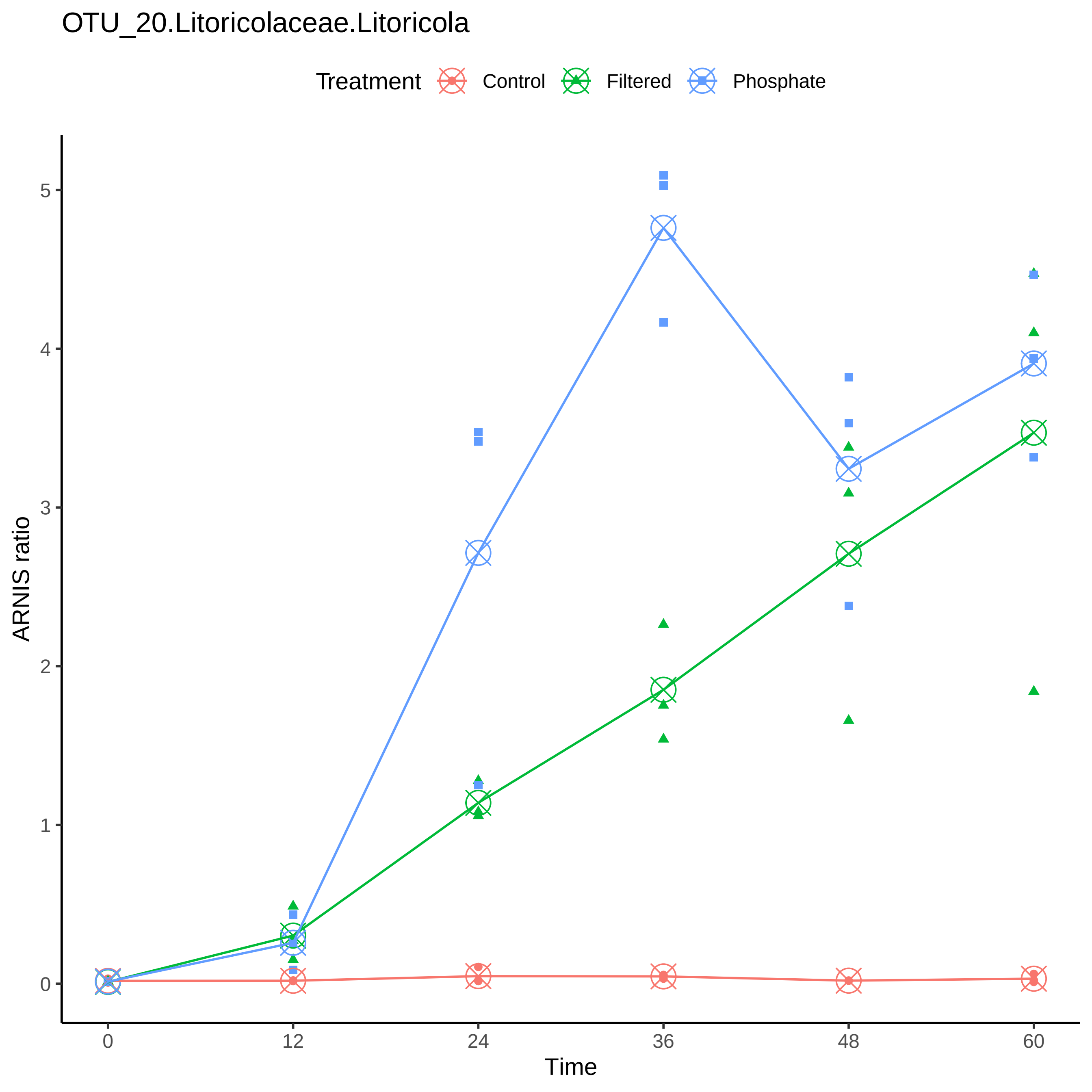
OTU_20.Litoricolaceae.Litoricola
Treatment
Control
Filtered
Phosphate
ARNIS ratio
0
1
2
3
4
5
Time
0
12
24
36
48
60

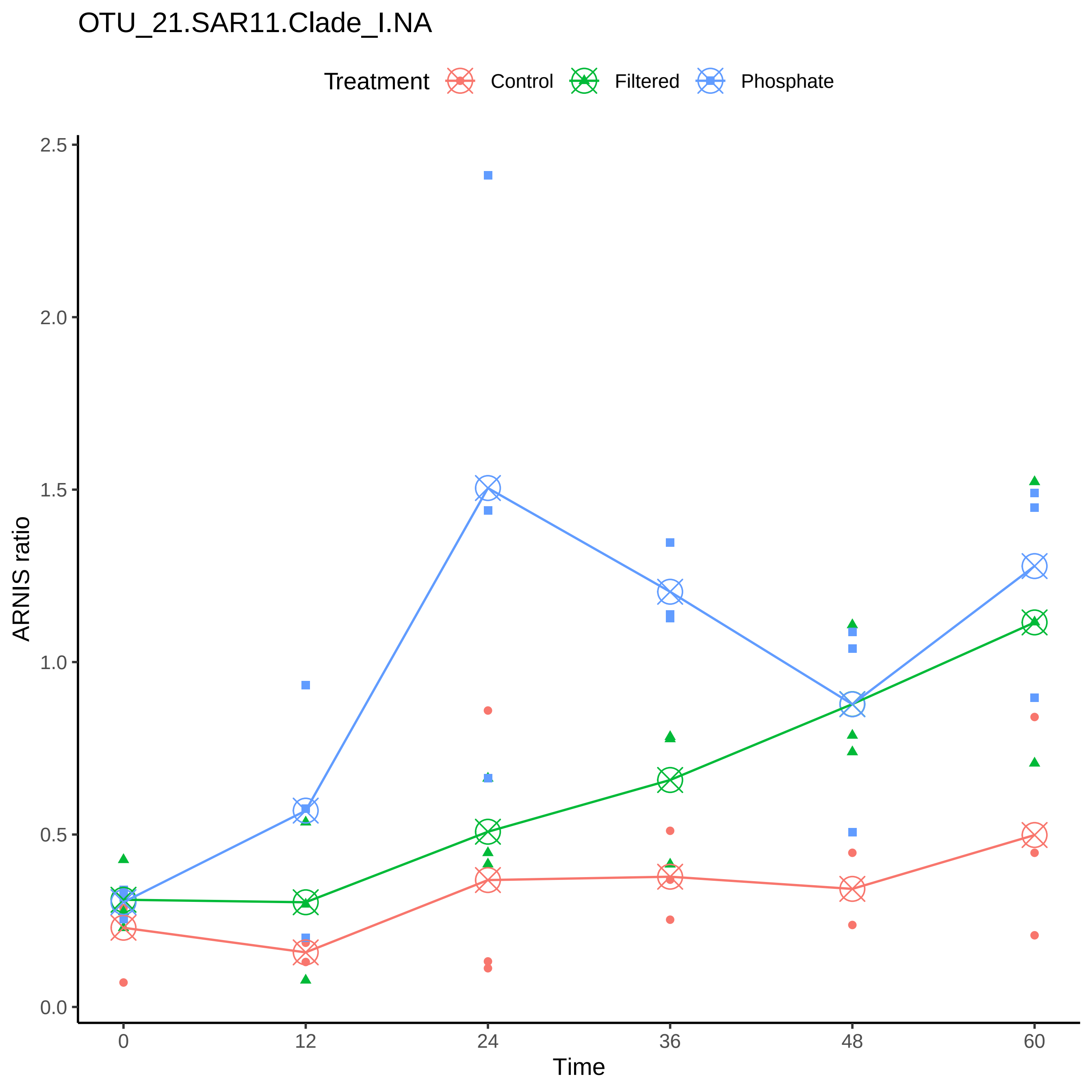

OTU_21.SAR11.Clade_I.NA
Treatment
Control
Filtered
Phosphate
ARNIS ratio
2.5
2.0
1.5
1.0
0.5
0.0
0
12
24
36
48
60
Time

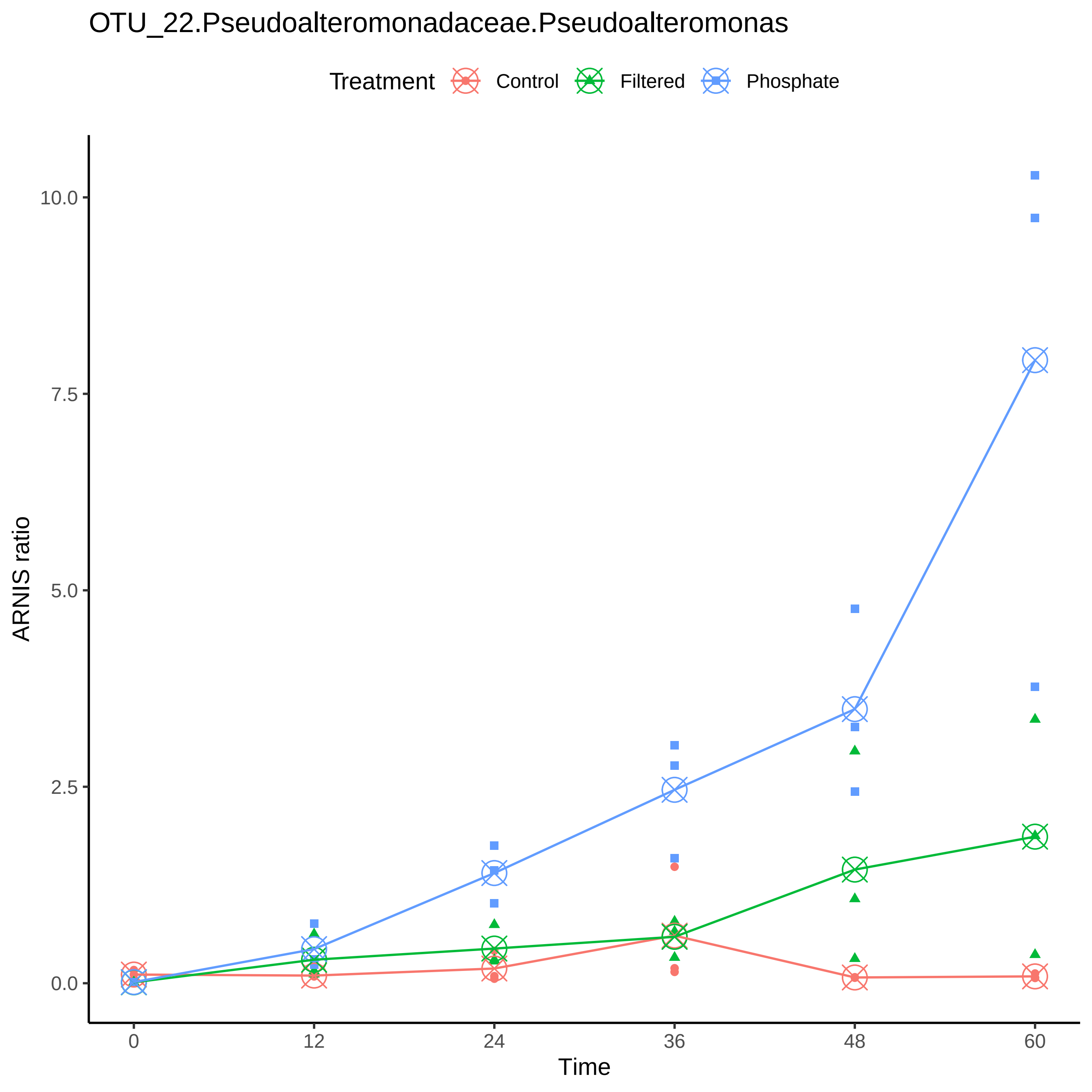

OTU_22.Pseudoalteromonadaceae.Pseudoalteromonas
Treatment
Control
Filtered
Phosphate
ARNIS ratio
10.0
7.5
5.0
2.5
0.0
0
12
24
36
48
60
Time

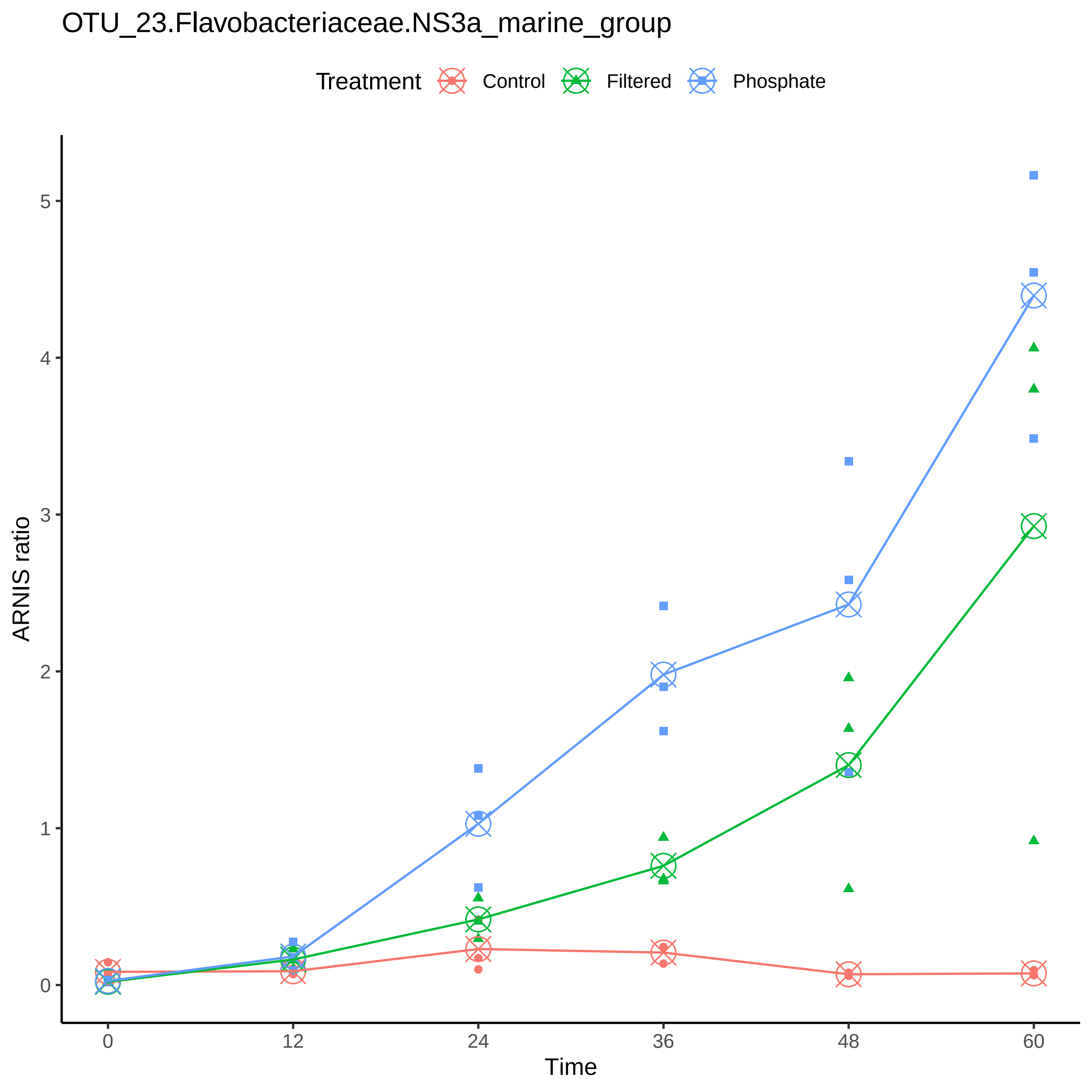
OTU_23.Flavobacteriaceae.NS3a_marine_group
Treatment
Control
Filtered
Phosphate
ARNIS ratio
0
1
2
3
4
5
Time
0
12
24
36
48
60

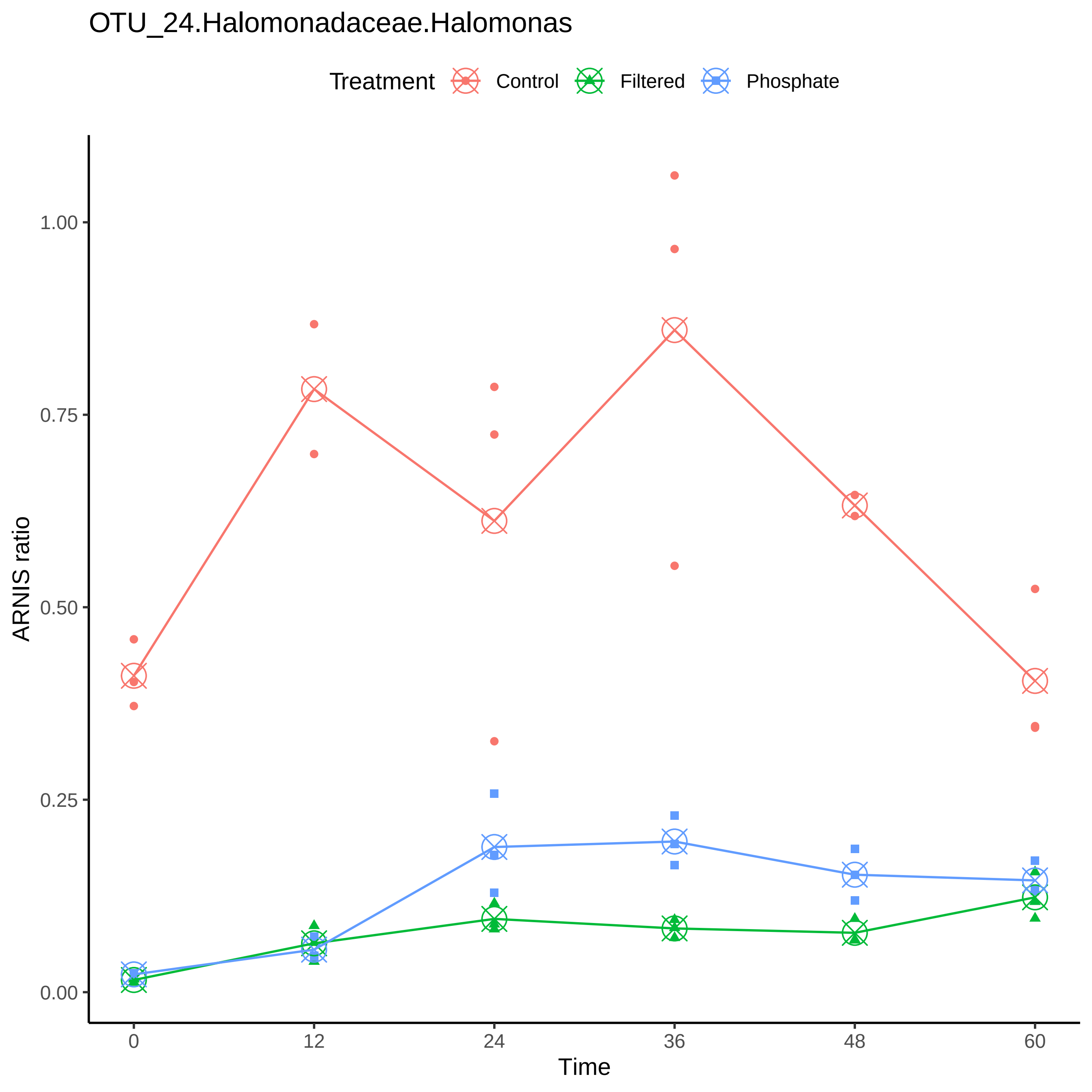
OTU_24.Halomonadaceae.Halomonas
Treatment
Control
Filtered
Phosphate
ARNIS ratio
1.00
0.75
0.50
0.25
0.00
0
12
24
36
48
60
Time

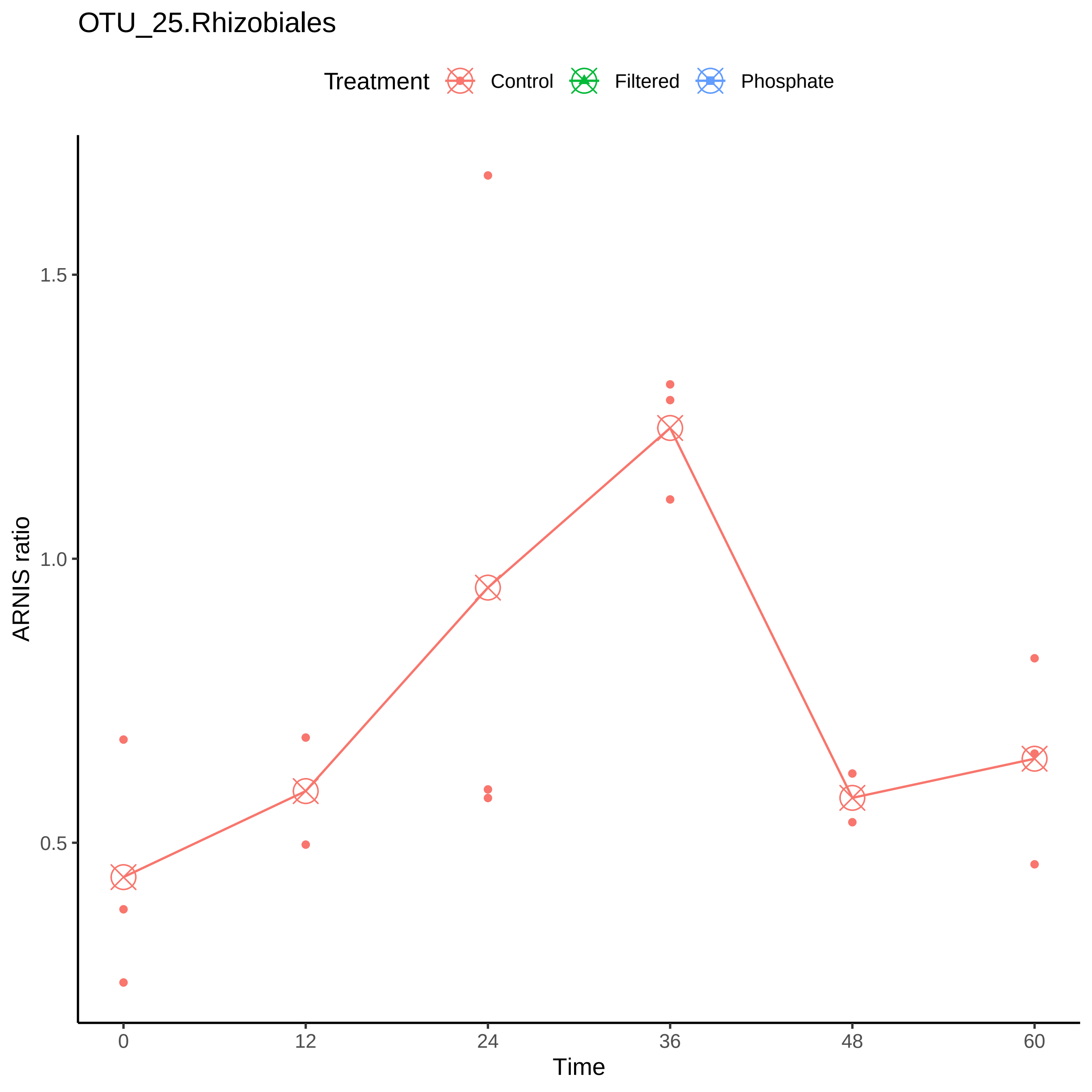
OTU_25.Rhizobiales
Treatment
Control
Filtered
Phosphate
ARNIS ratio
1.5
1.0
0.5
0
12
24
36
48
60
Time

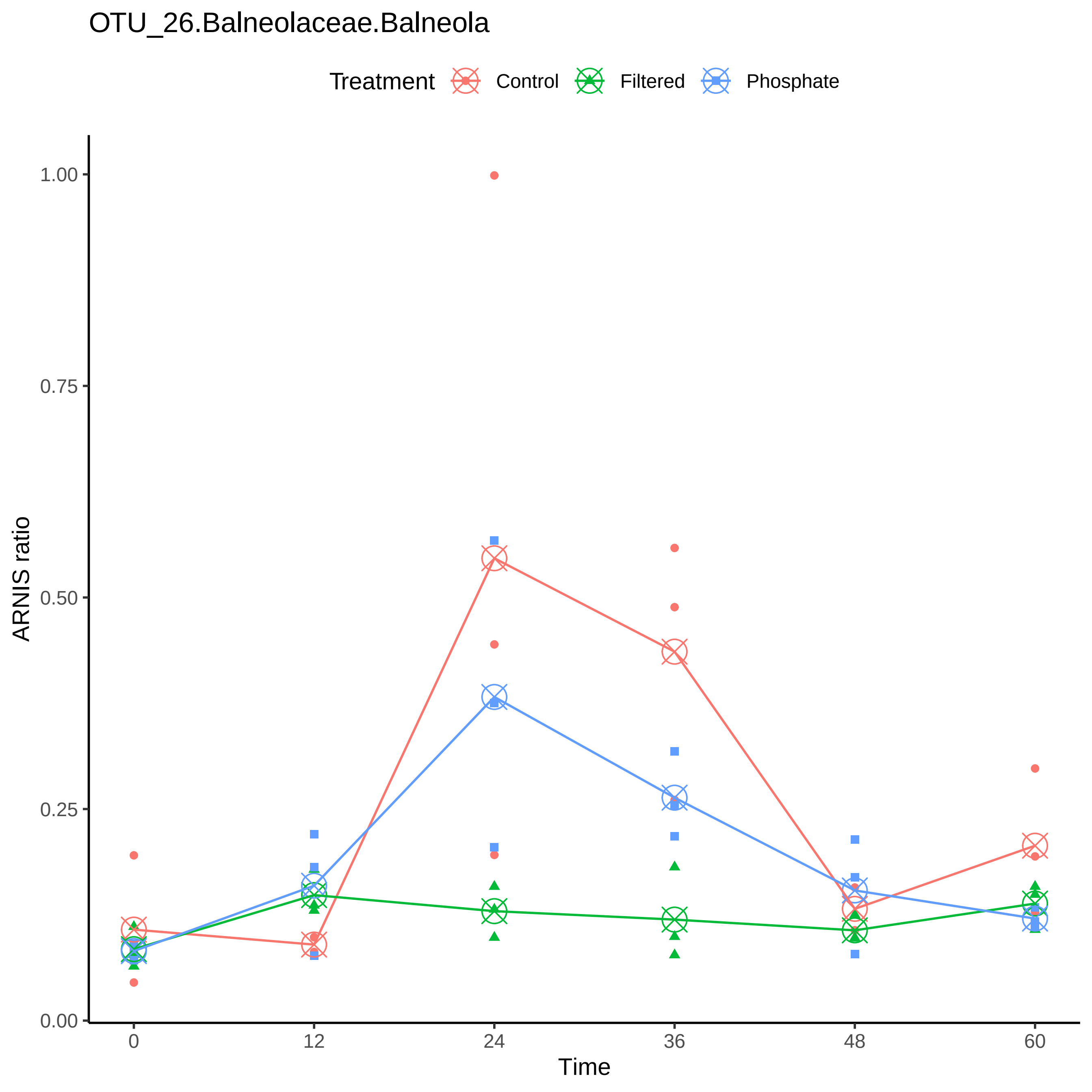
OTU_26.Balneolaceae.Balneola
Treatment
Control
Filtered
Phosphate
ARNIS ratio
1.00
0.75
0.50
0.25
0.00
0
12
24
36
48
60
Time

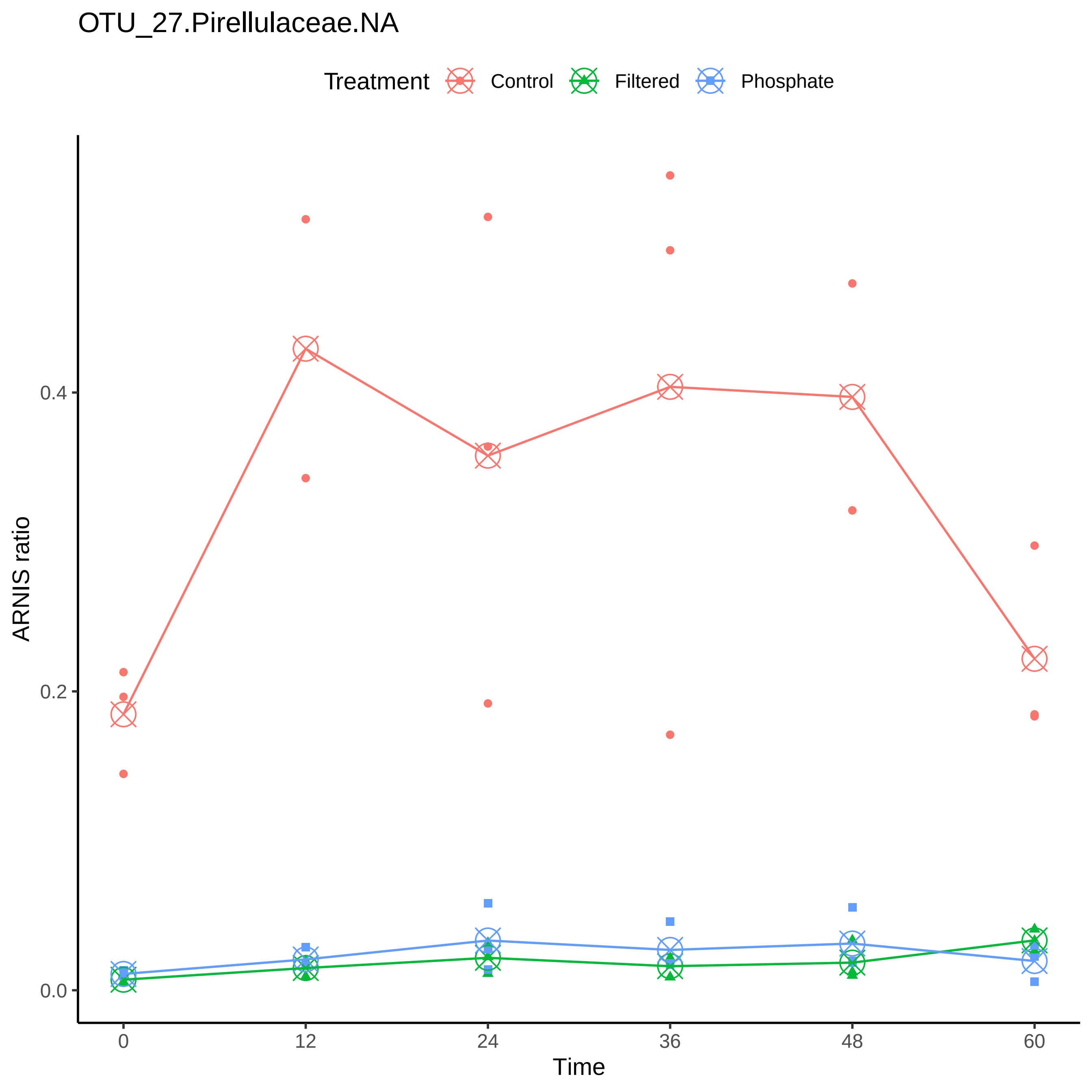

OTU_27.Pirellulaceae.NA
Treatment
Control
Filtered
Phosphate
ARNIS ratio
0.0
0.2
0.4
0
12
24
36
48
60
Time

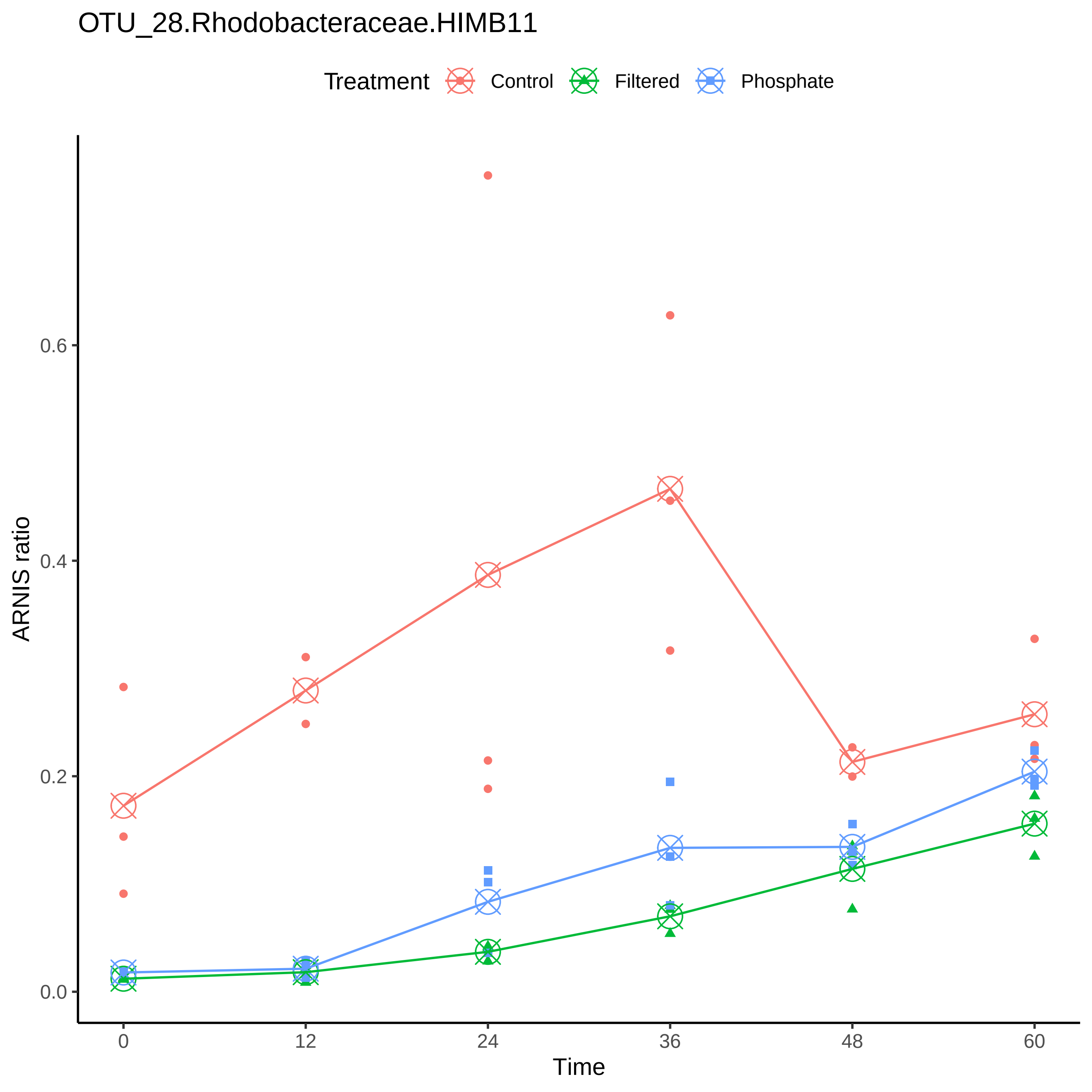
OTU_28.Rhodobacteraceae.HIMB11
Treatment
Control
Filtered
Phosphate
ARNIS ratio
0.0
0.2
0.4
0.6
Time
0
12
24
36
48
60

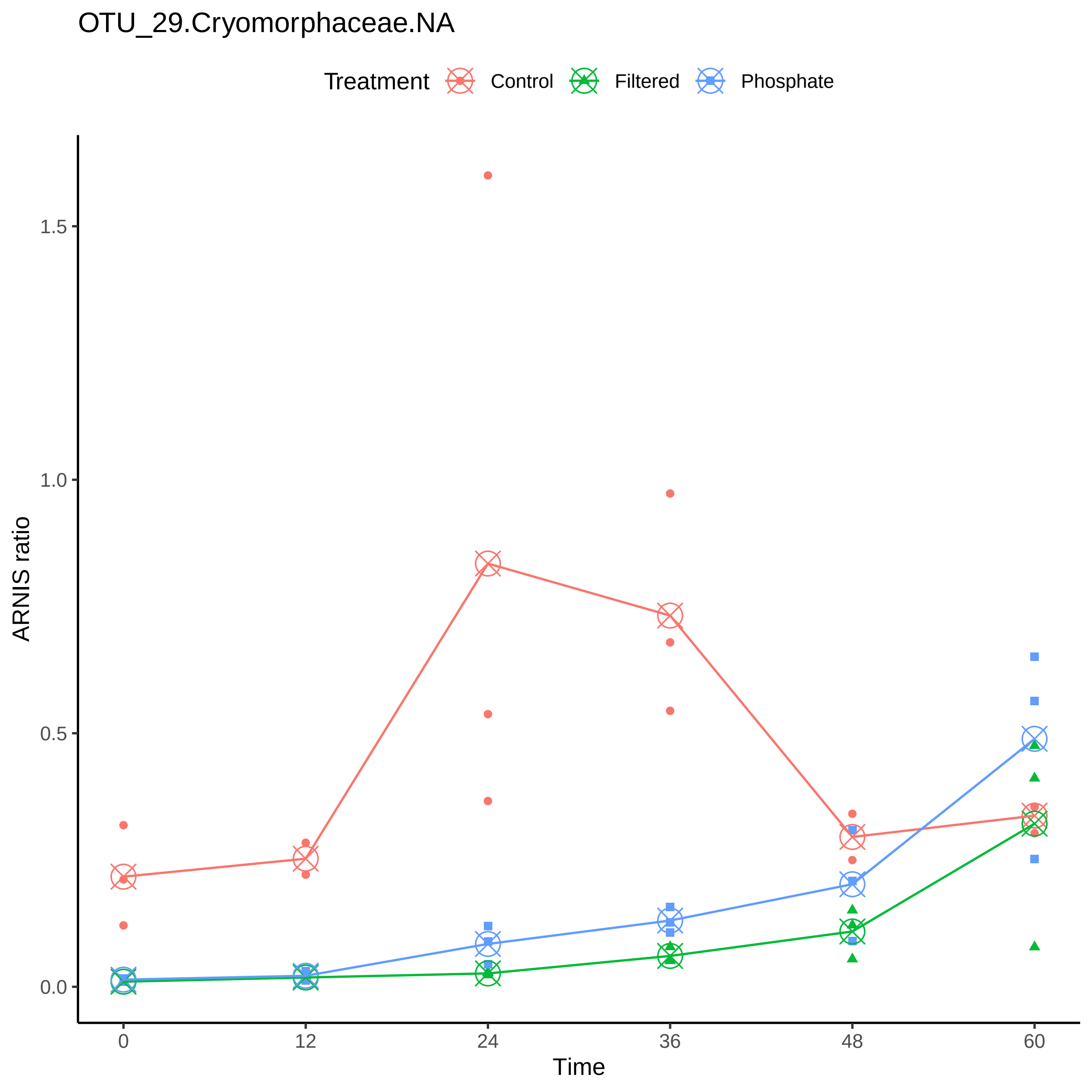

OTU_29.Cryomorphaceae.NA
Treatment
Control
Filtered
Phosphate
ARNIS ratio
1.5
1.0
0.5
0.0
0
12
24
36
48
60
Time

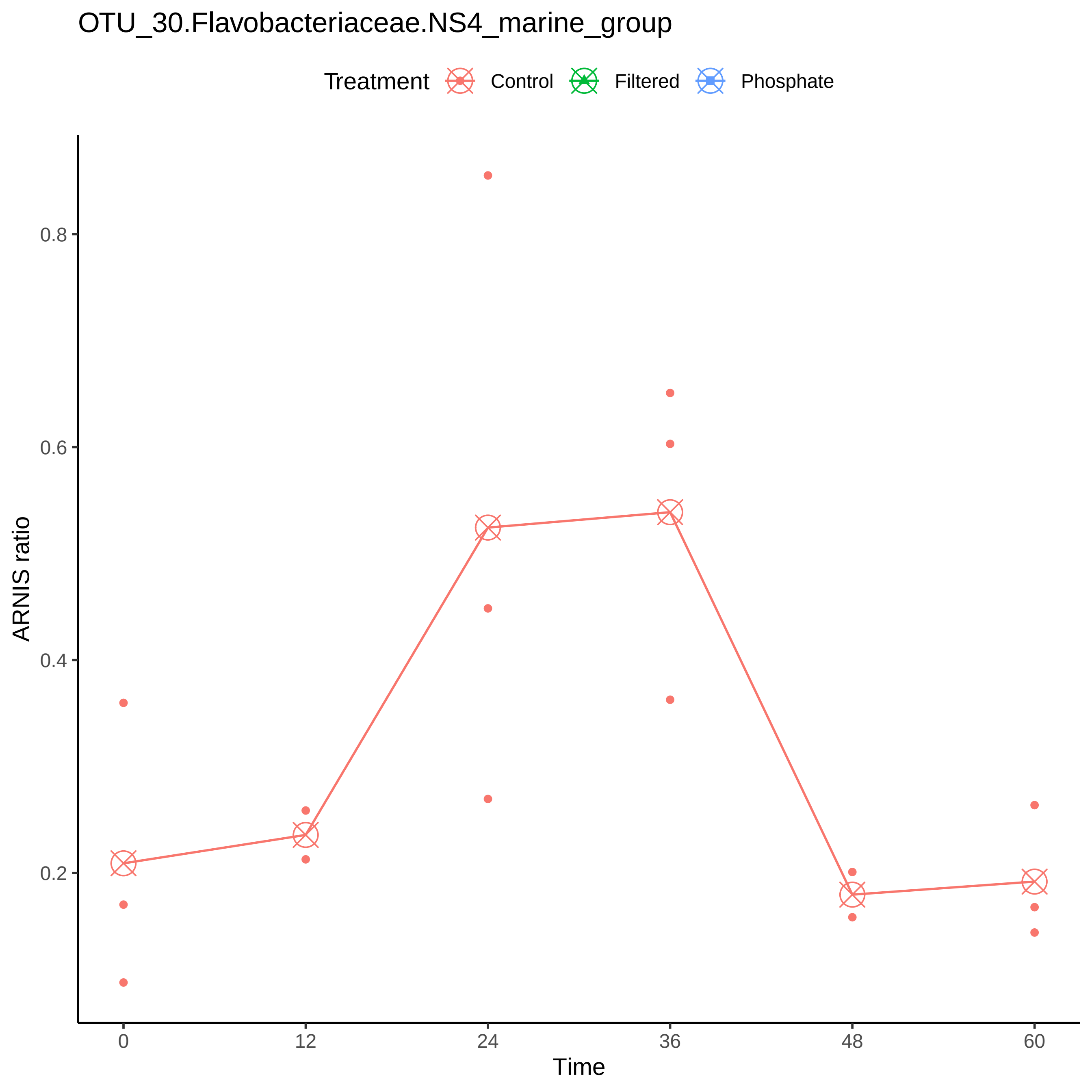

OTU_30.Flavobacteriaceae.NS4_marine_group
Treatment
Control
Filtered
Phosphate
ARNIS ratio
0.2
0.4
0.6
0.8
Time
0
12
24
36
48
60

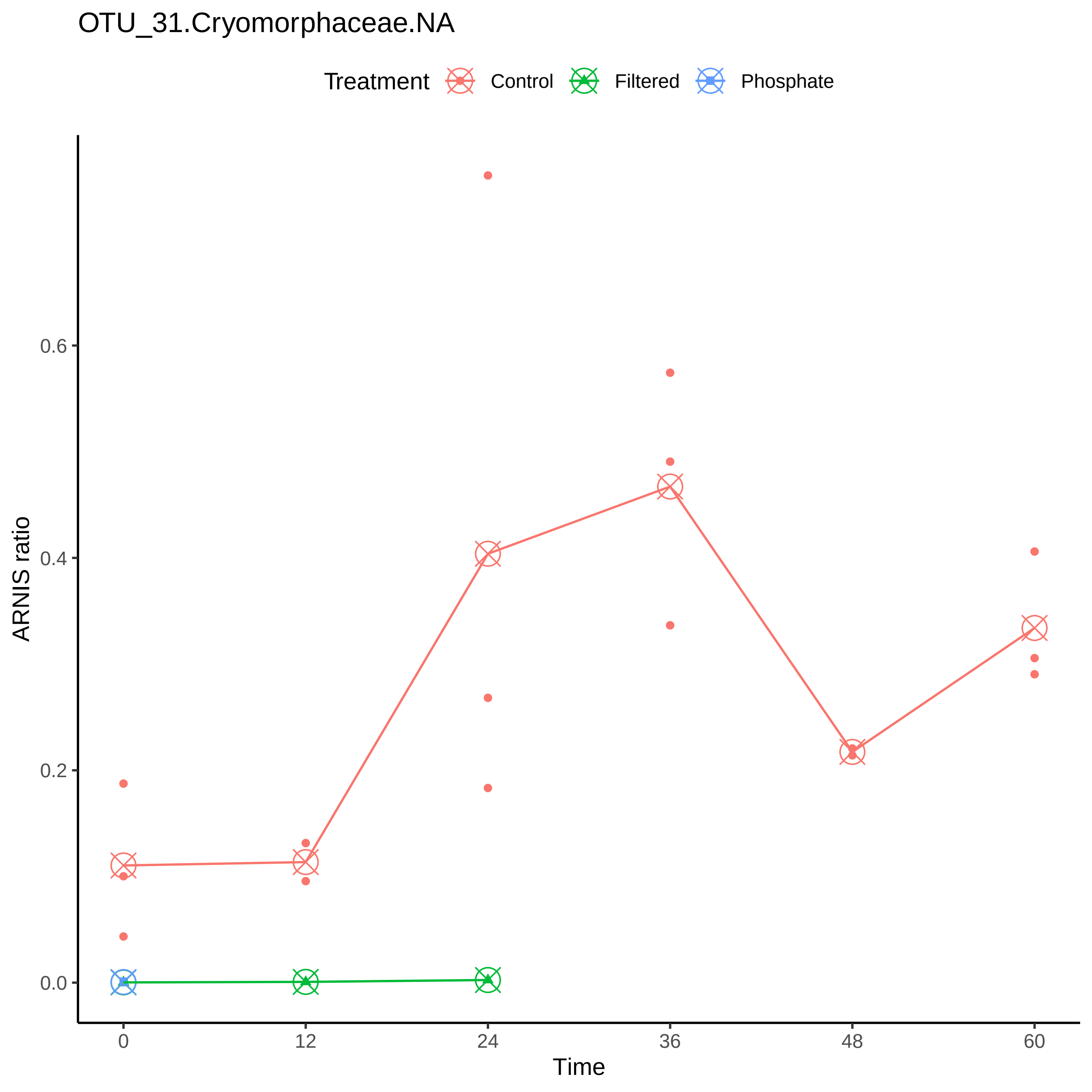

OTU_31.Cryomorphaceae.NA
Treatment
Control
Filtered
Phosphate
ARNIS ratio
0.0
0.2
0.4
0.6
Time
0
12
24
36
48
60

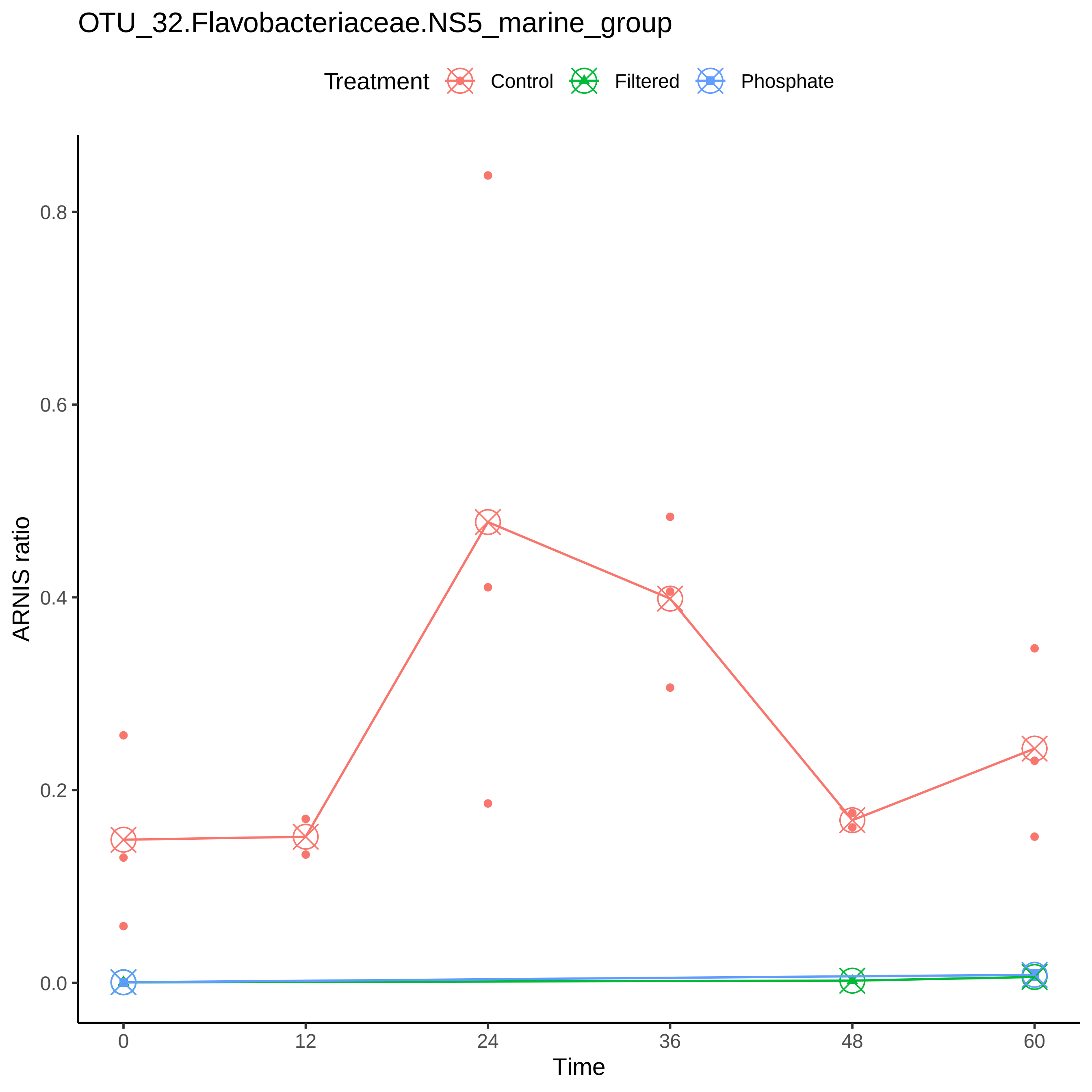
OTU_32.Flavobacteriaceae.NS5_marine_group
Treatment
Control
Filtered
Phosphate
ARNIS ratio
0.0
0.2
0.4
0.6
0.8
0
12
24
36
48
60
Time

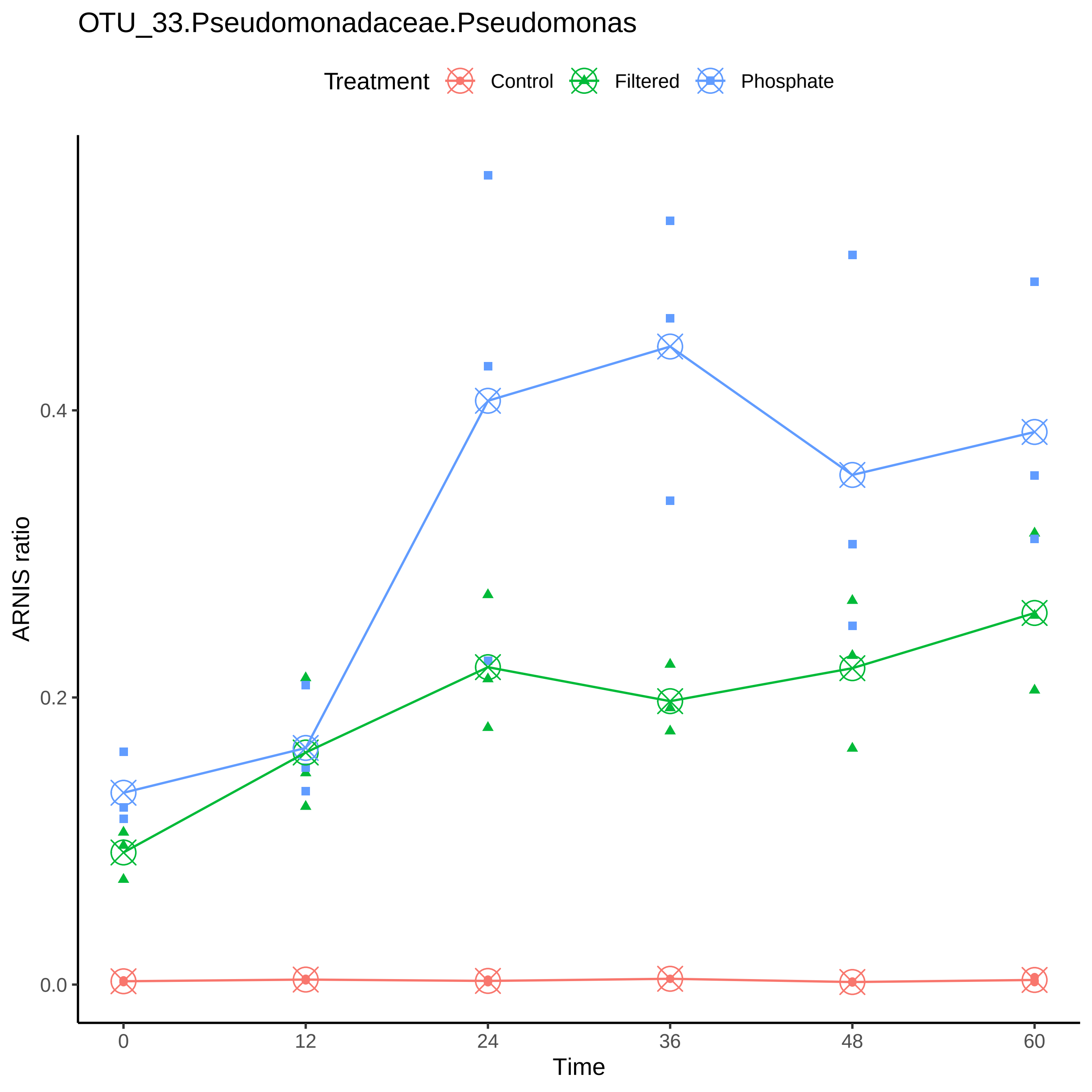
OTU_33.Pseudomonadaceae.Pseudomonas
Treatment
Control
Filtered
Phosphate
ARNIS ratio
0.0
0.2
0.4
Time
0
12
24
36
48
60

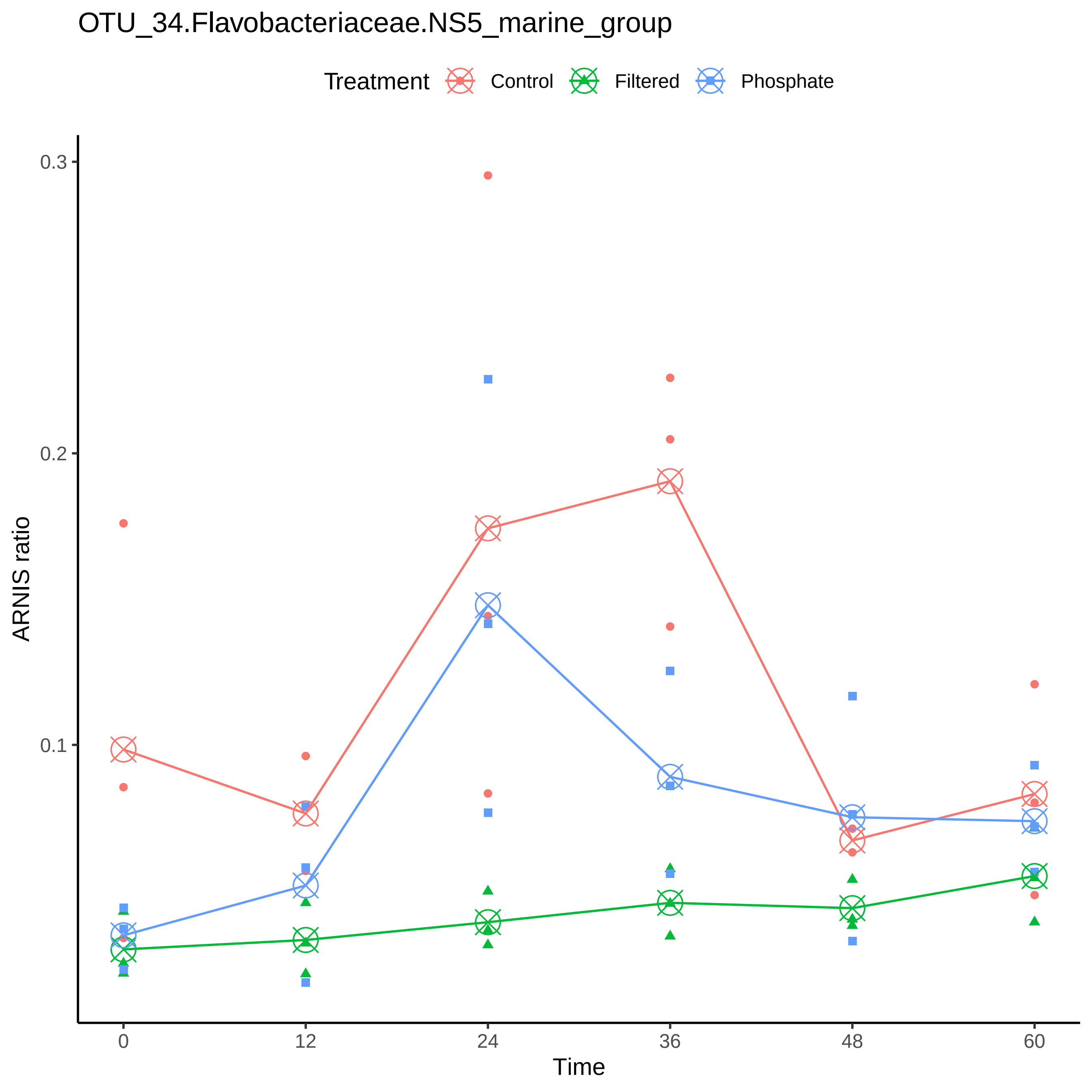
OTU_34.Flavobacteriaceae.NS5_marine_group
Treatment
Control
Filtered
Phosphate
0.3
0.2
0.1
ARNIS ratio
0
12
24
36
48
60
Time

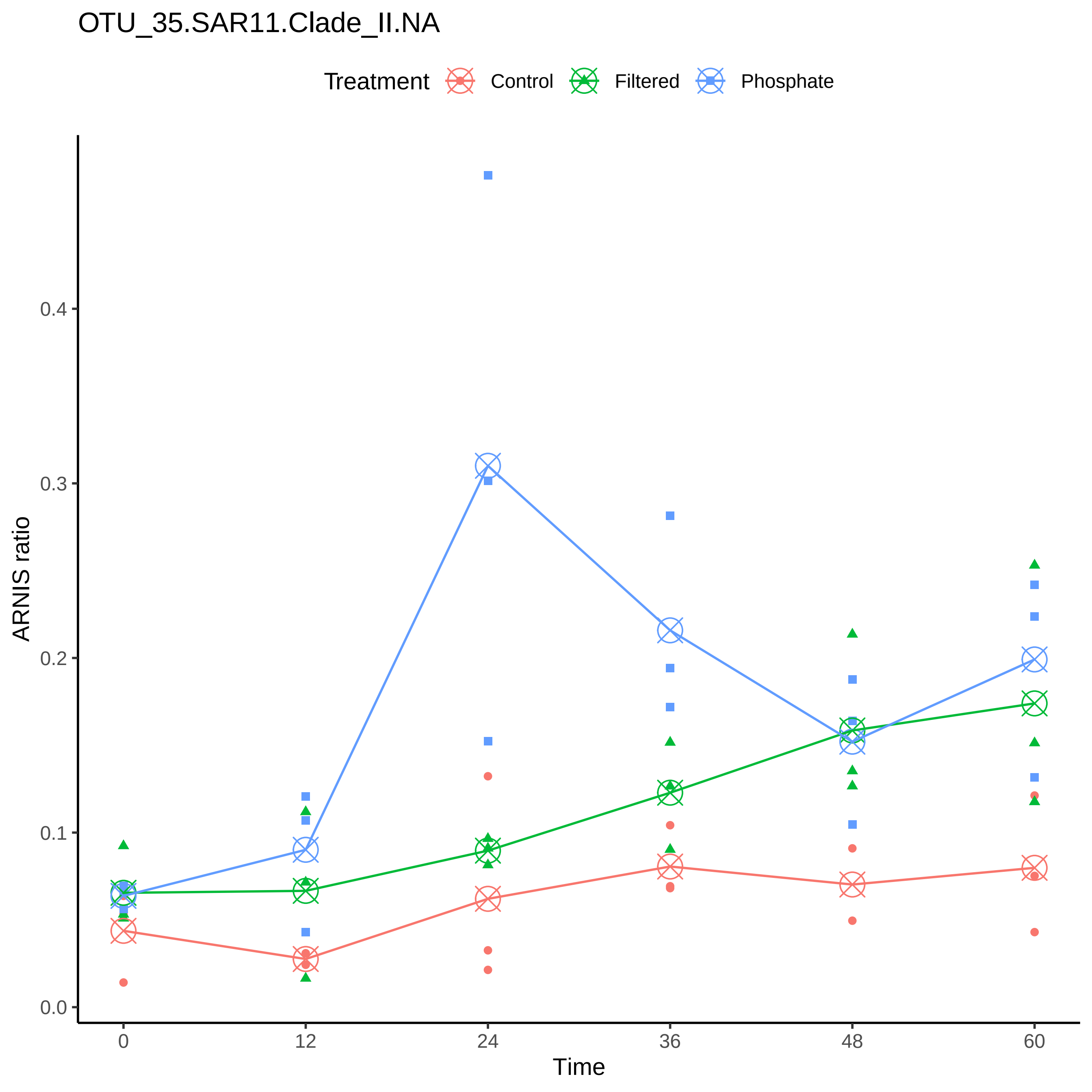

OTU_35.SAR11.Clade_II.NA
Treatment
Control
Filtered
Phosphate
ARNIS ratio
0.0
0.1
0.2
0.3
0.4
Time
0
12
24
36
48
60

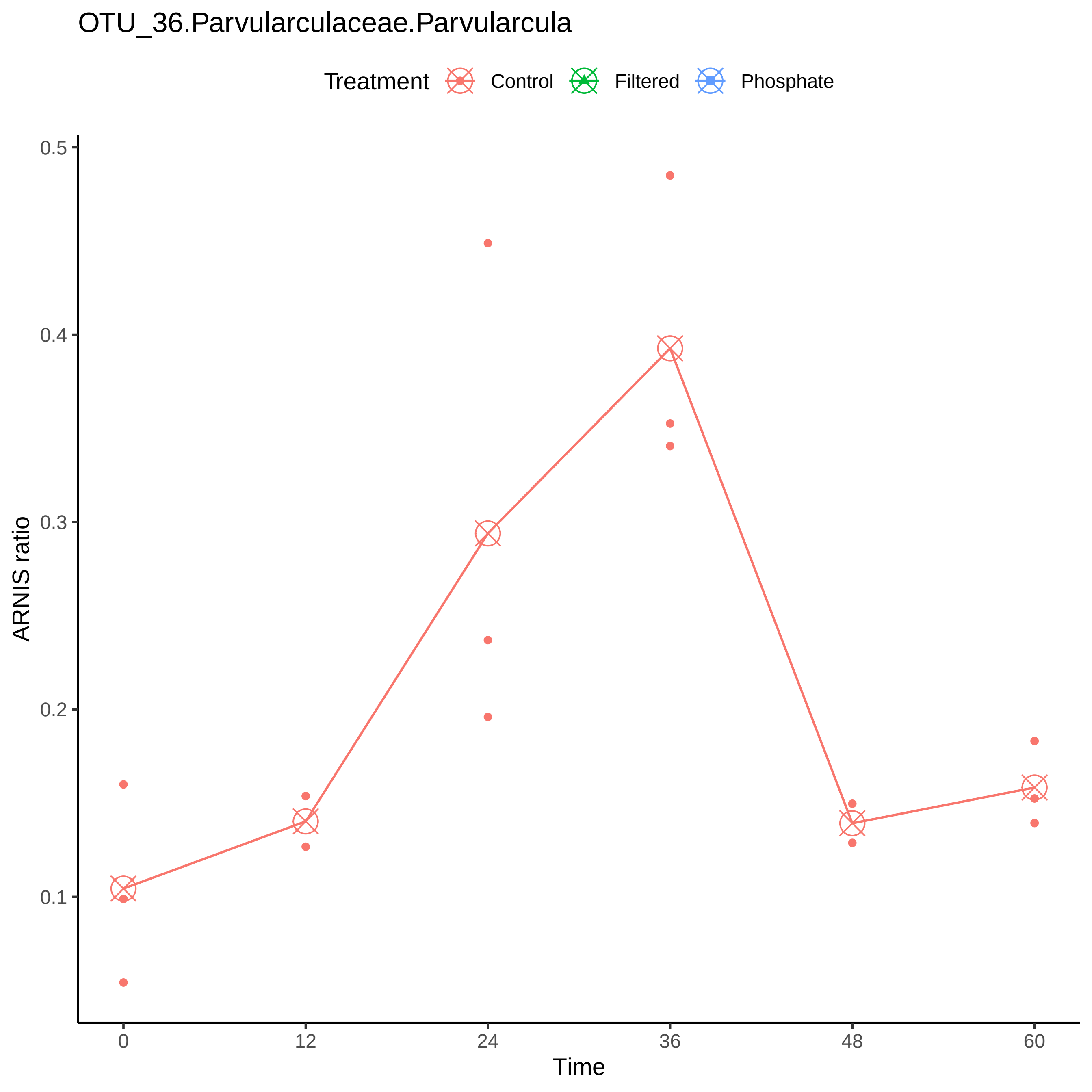
OTU_36.Parvularculaceae.Parvularcula
Treatment
Control
Filtered
Phosphate
ARNIS ratio
0.5
0.4
0.3
0.2
0.1
0
12
24
36
48
60
Time

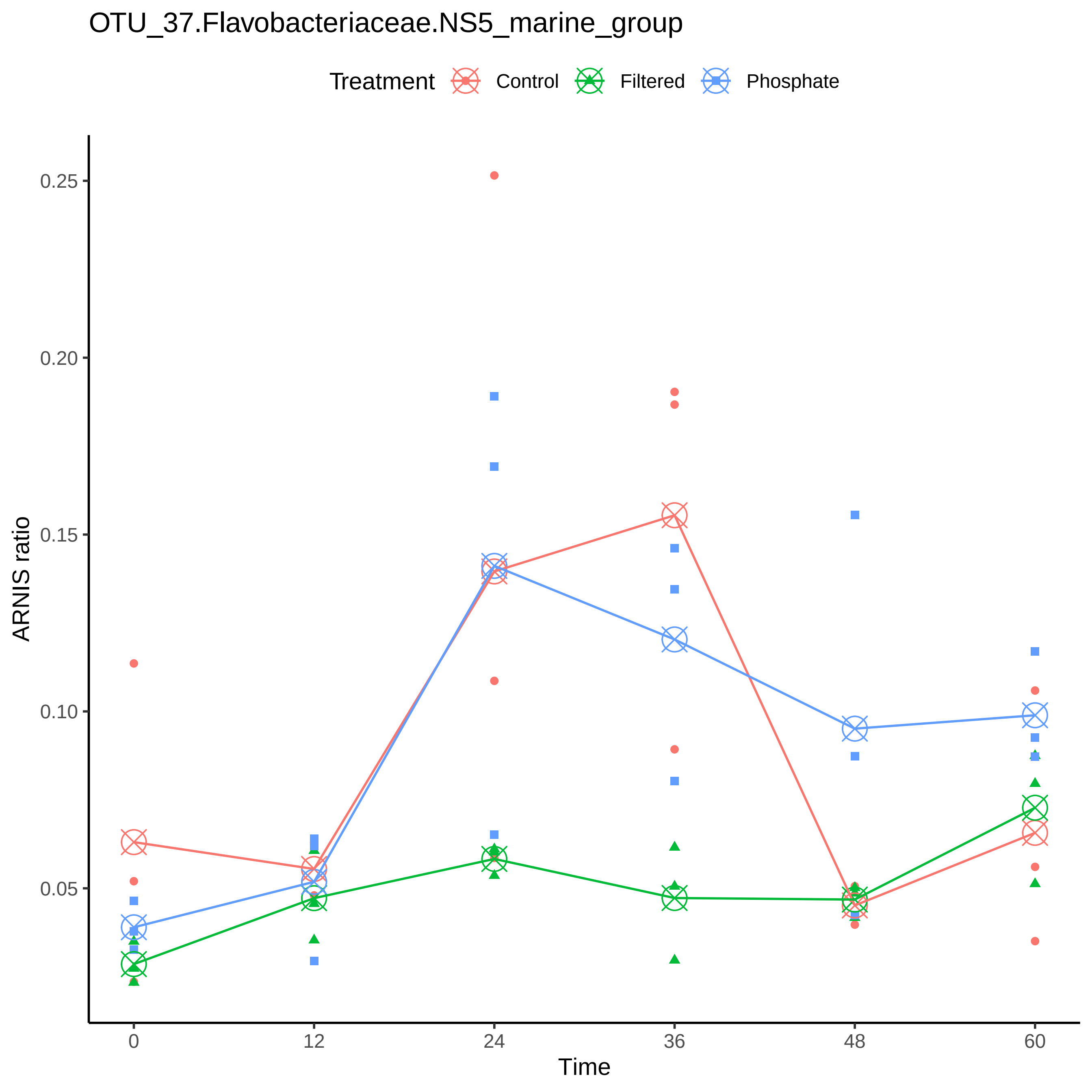

OTU_37.Flavobacteriaceae.NS5_marine_group
Treatment
Control
Filtered
Phosphate
ARNIS ratio
0.25
0.20
0.15
0.10
0.05
Time
0
12
24
36
48
60

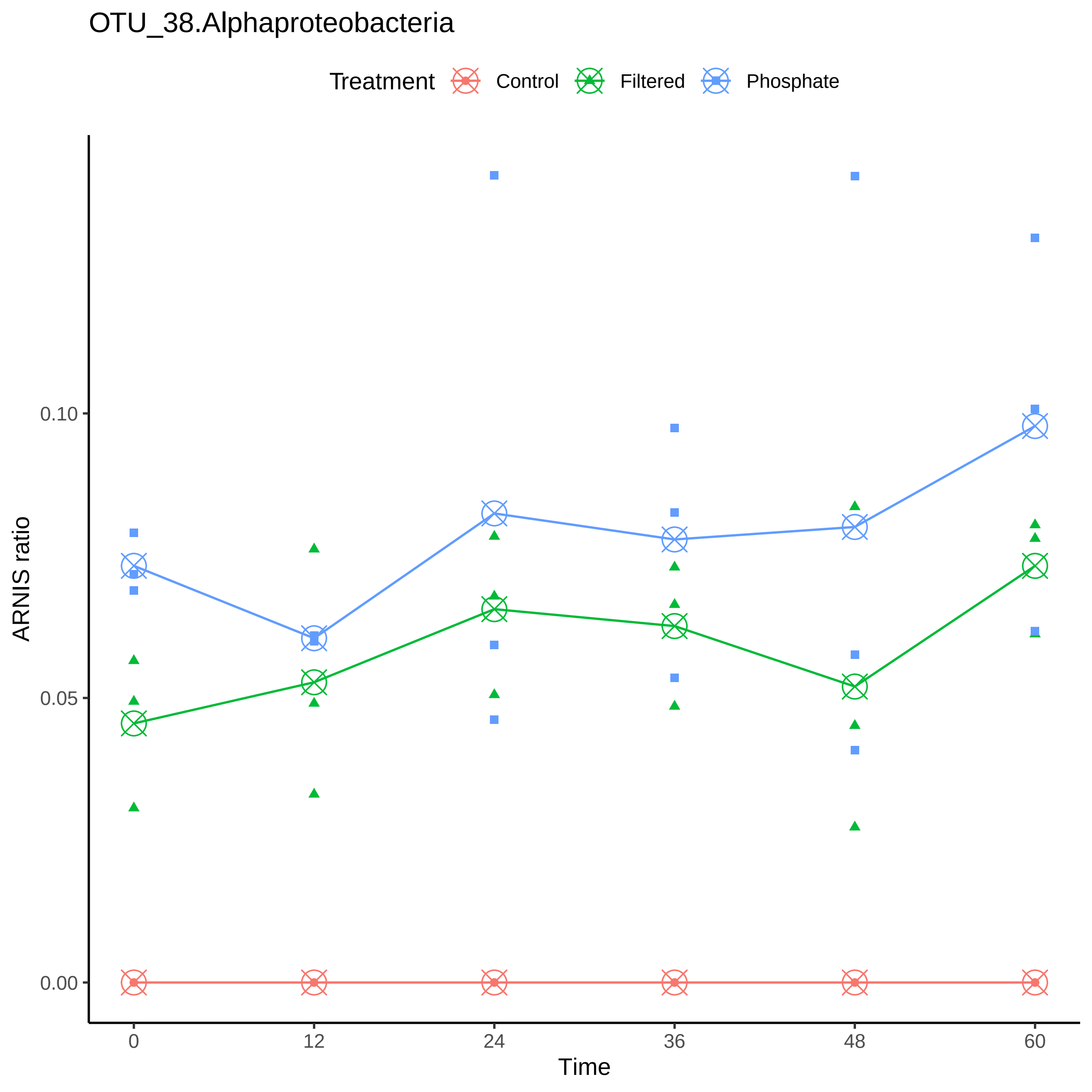

OTU_38.Alphaproteobacteria
Treatment
Control
Filtered
Phosphate
ARNIS ratio
0.10
0.05
0.00
0
12
24
36
48
60
Time

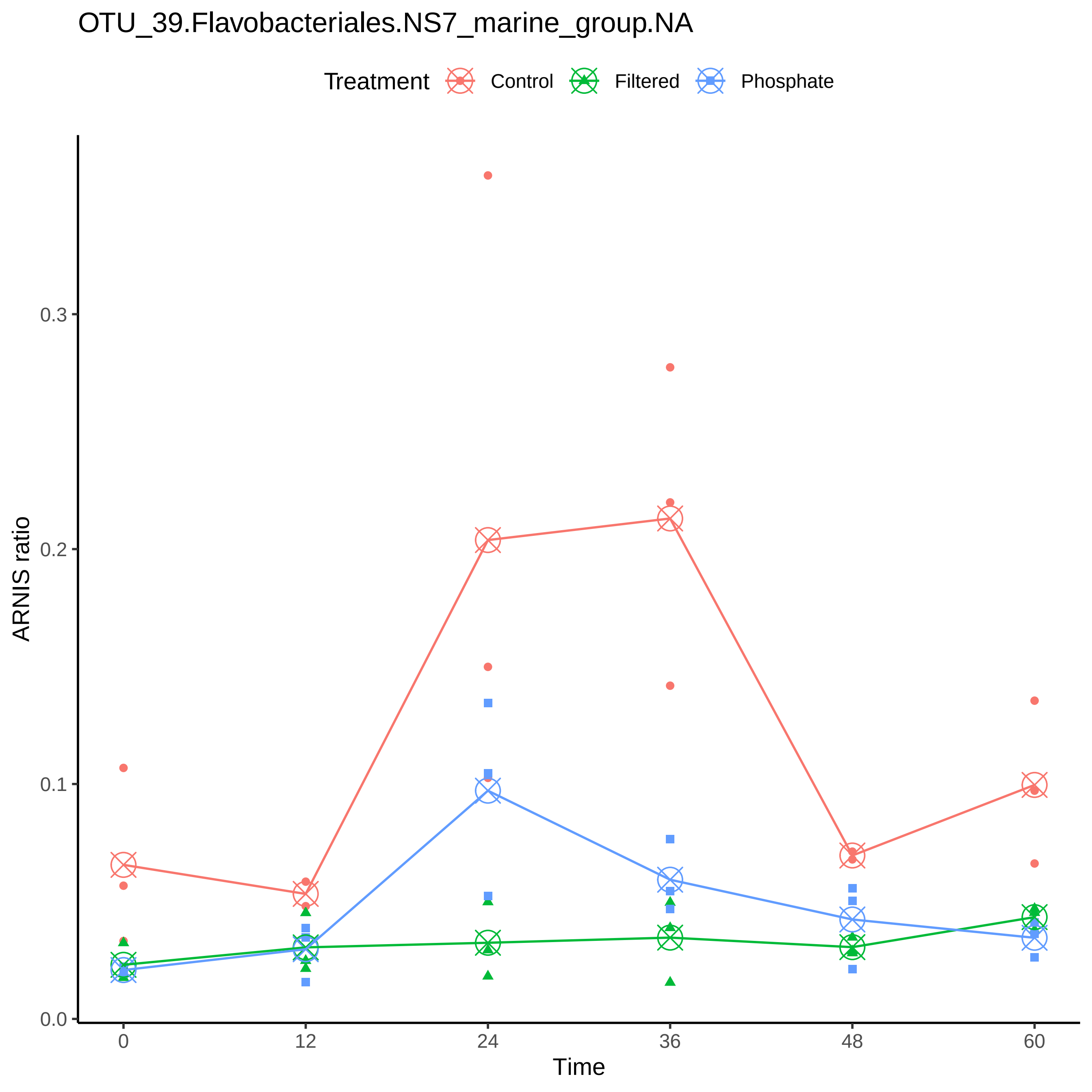
OTU_39.Flavobacteriales.NS7_marine_group.NA
Treatment
Control
Filtered
Phosphate
ARNIS ratio
0.0
0.1
0.2
0.3
Time
0
12
24
36
48
60

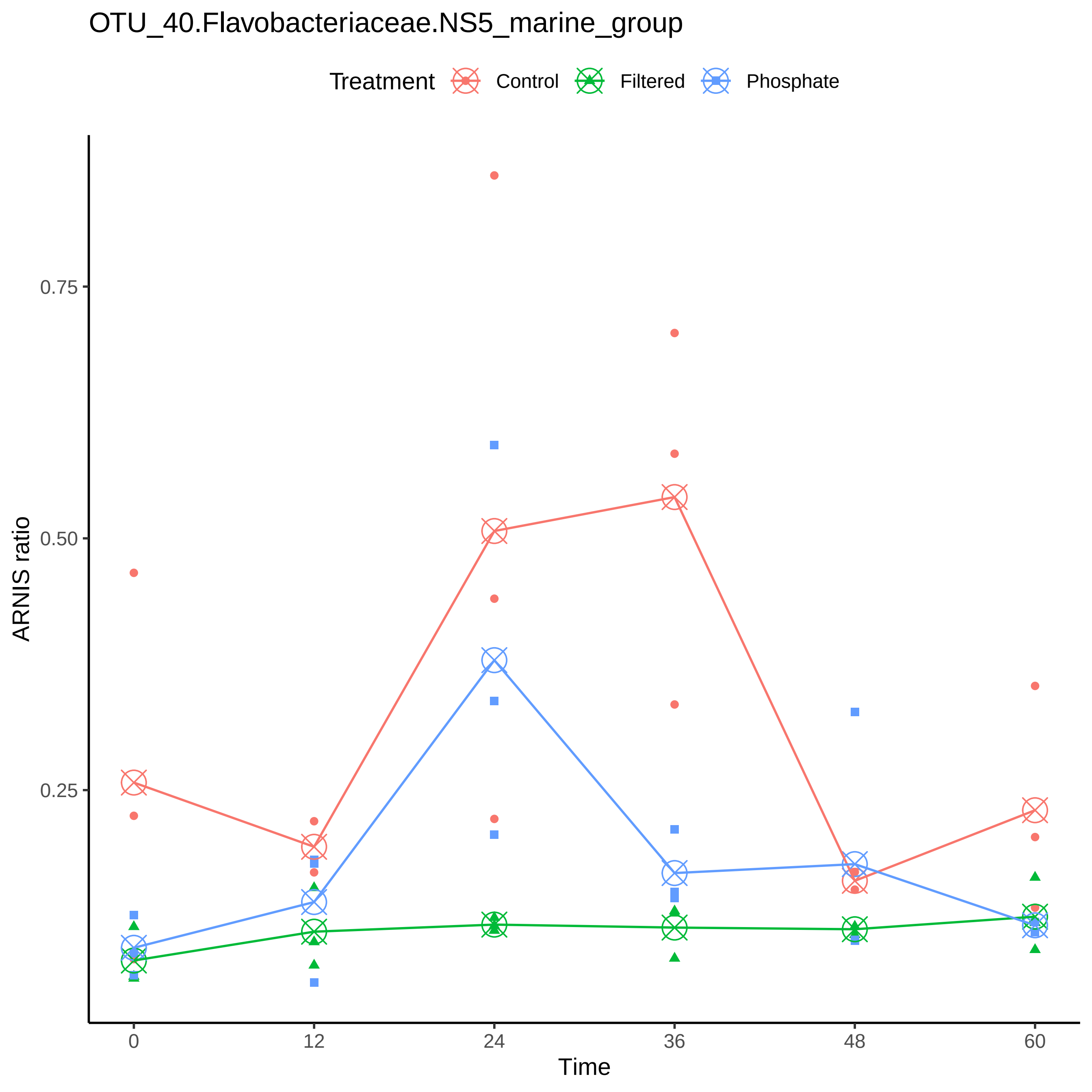
OTU_40.Flavobacteriaceae.NS5_marine_group
Treatment
Control
Filtered
Phosphate
ARNIS ratio
0.75
0.50
0.25
Time
0
12
24
36
48
60

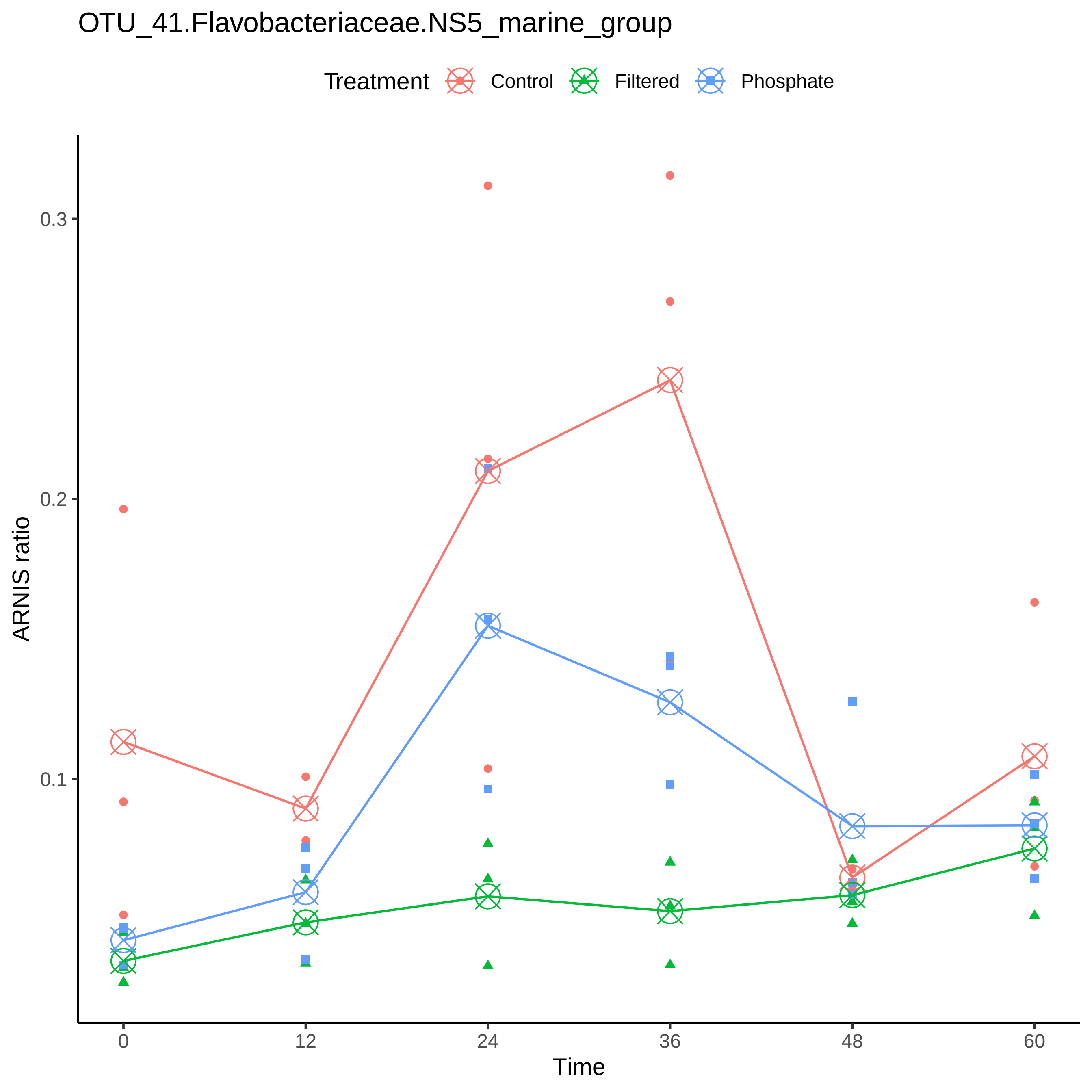

OTU_41.Flavobacteriaceae.NS5_marine_group
Treatment
Control
Filtered
Phosphate
ARNIS ratio
0.1
0.2
0.3
Time
0
12
24
36
48
60

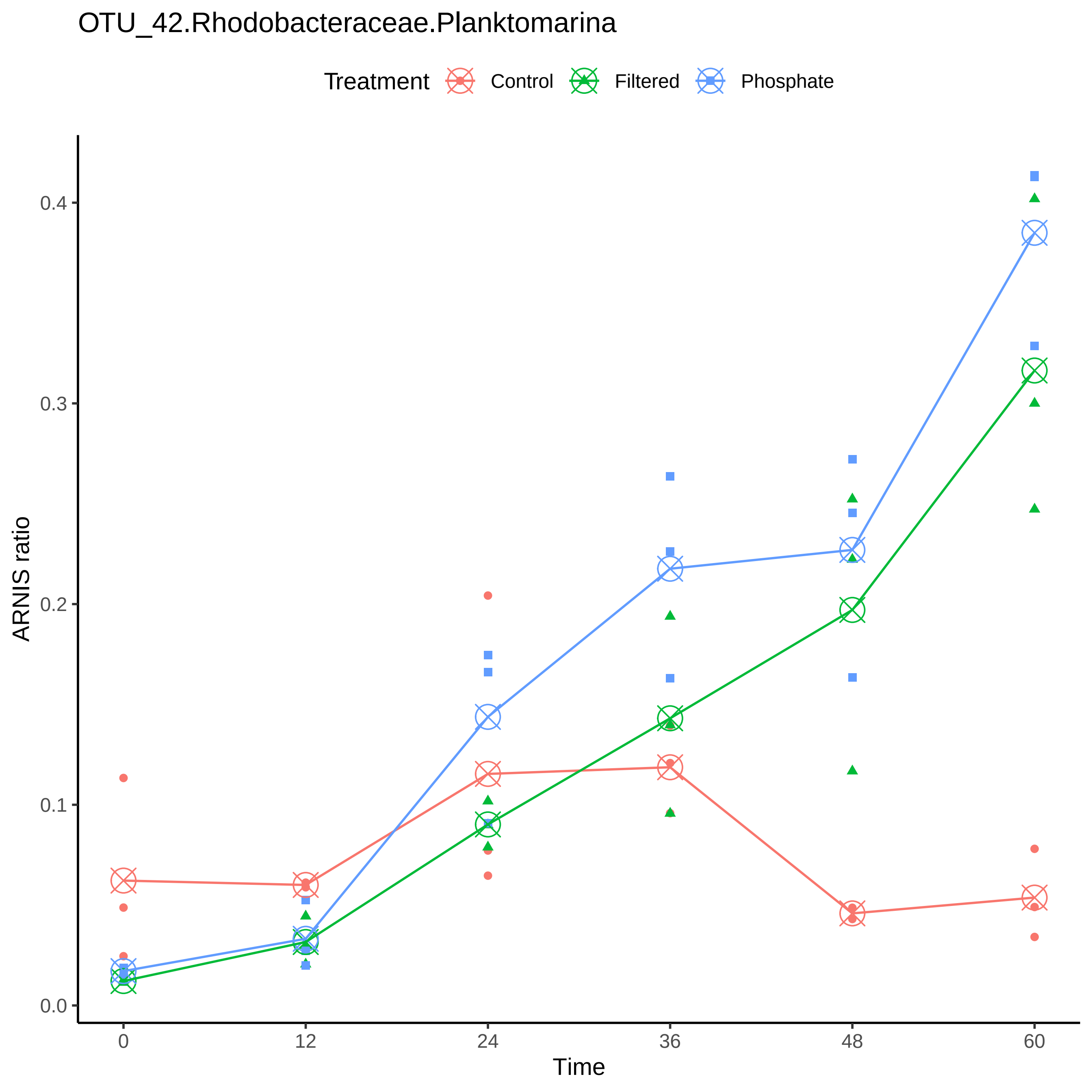
OTU_42.Rhodobacteraceae.Planktomarina
Treatment
Control
Filtered
Phosphate
ARNIS ratio
0.0
0.1
0.2
0.3
0.4
Time
0
12
24
36
48
60

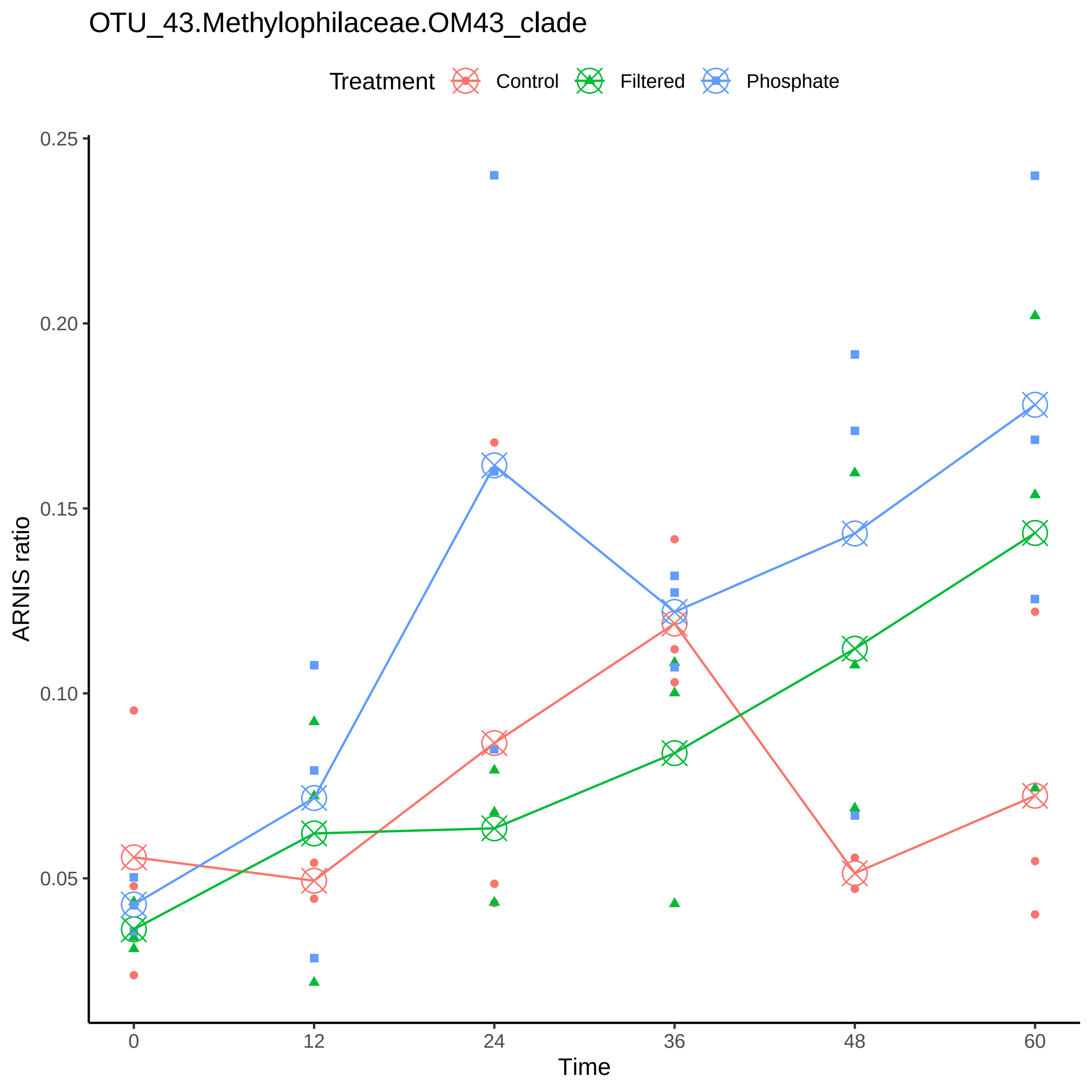
OTU_43.Methylophilaceae.OM43_clade
Treatment
Control
Filtered
Phosphate
ARNIS ratio
0.25
0.20
0.15
0.10
0.05
0
12
24
36
48
60
Time

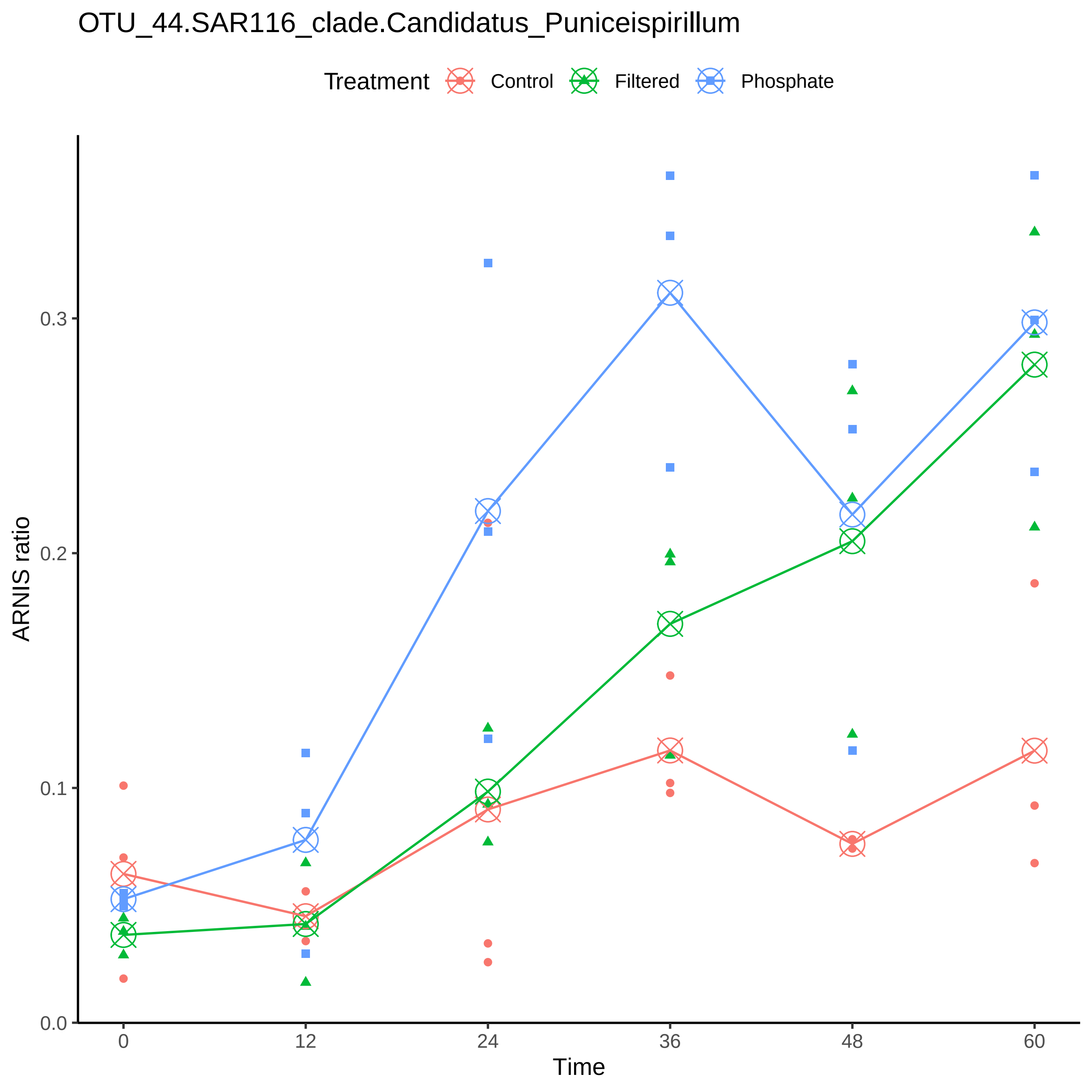

OTU_44.SAR116_clade.Candidatus_Puniceispirillum
Treatment
Control
Filtered
Phosphate
ARNIS ratio
0.0
0.1
0.2
0.3
Time
0
12
24
36
48
60

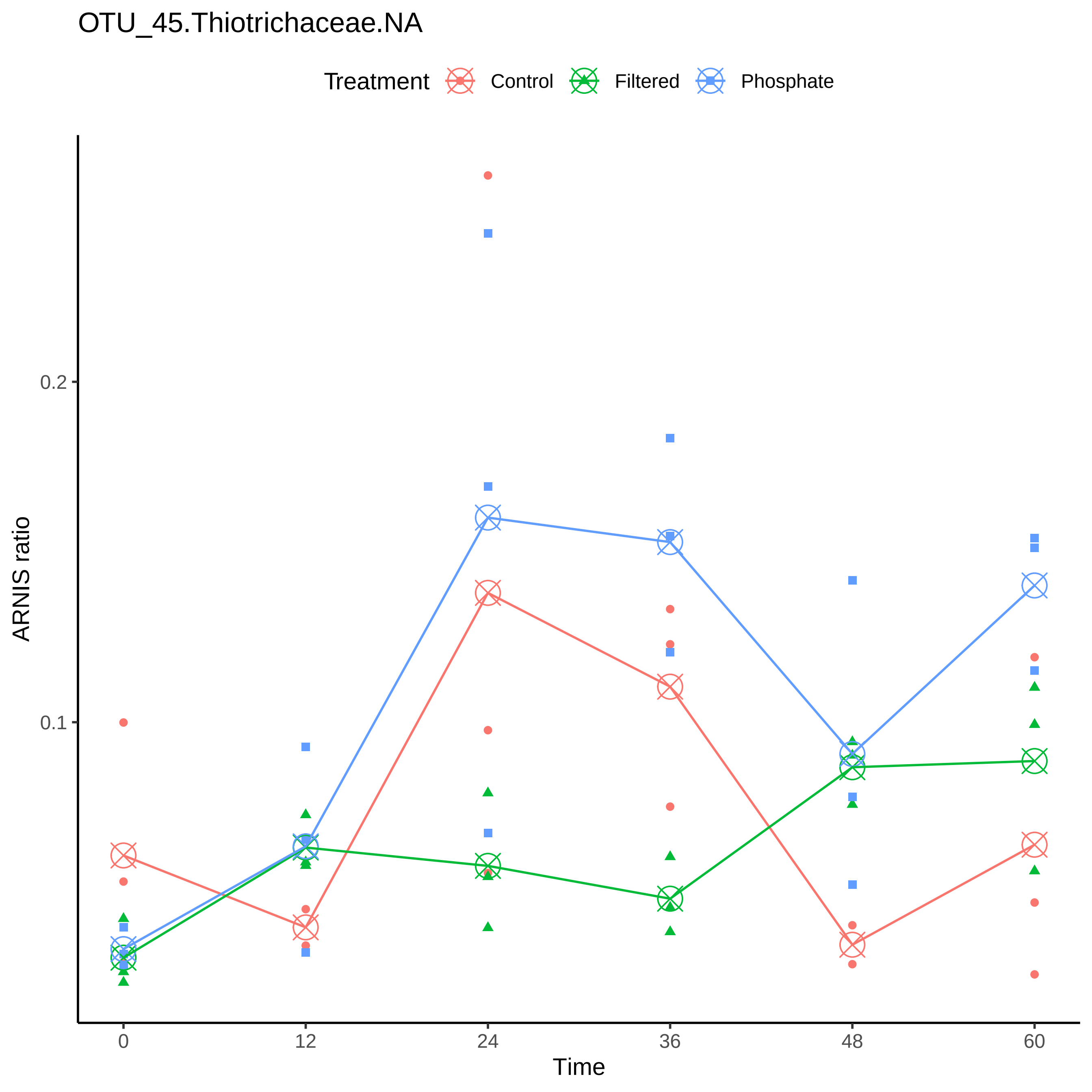

OTU_45.Thiotrichaceae.NA
Treatment
Control
Filtered
Phosphate
ARNIS ratio
0.2
0.1
0
12
24
36
48
60
Time

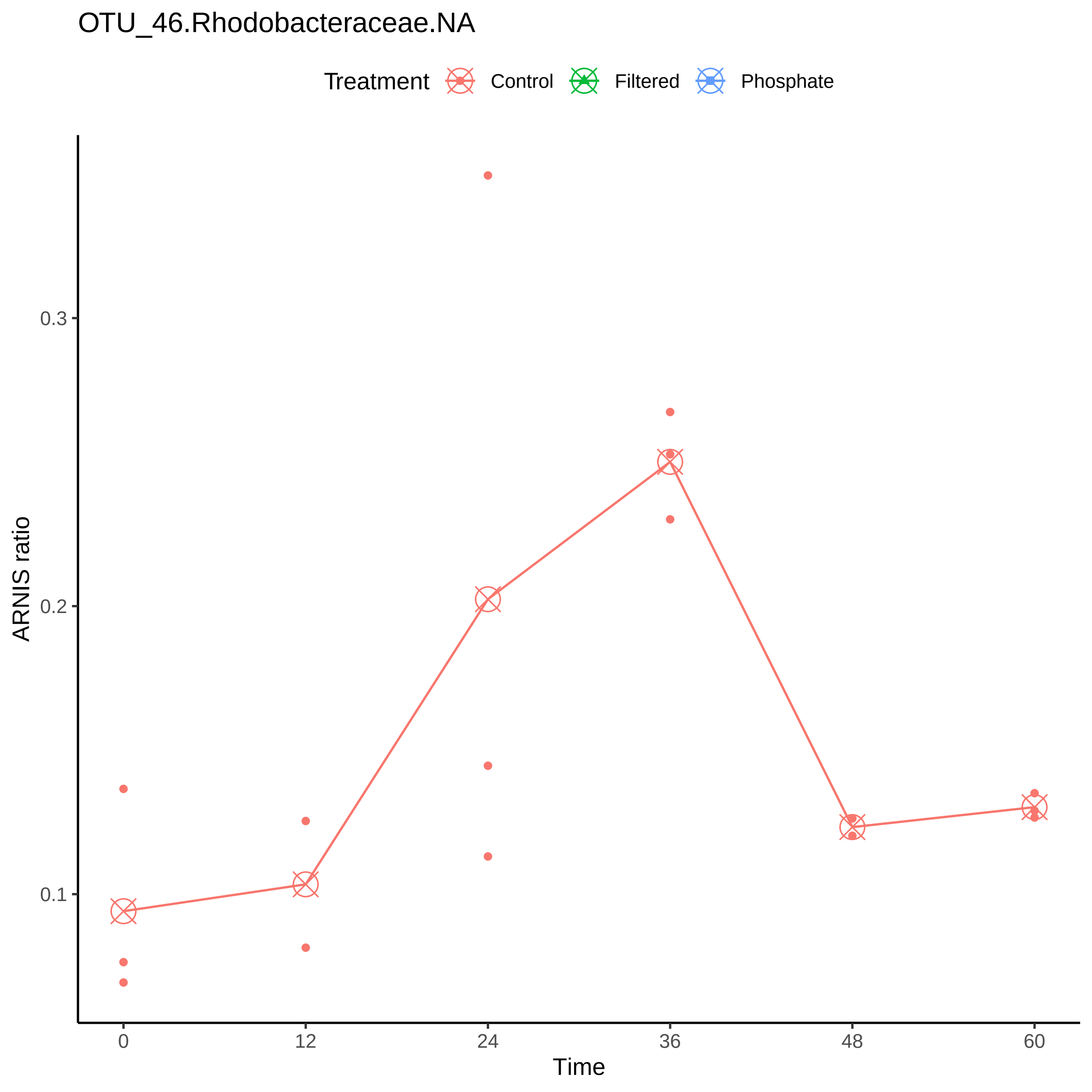
OTU_46.Rhodobacteraceae.NA
Treatment
Control
Filtered
Phosphate
ARNIS ratio
0.1
0.2
0.3
Time
0
12
24
36
48
60

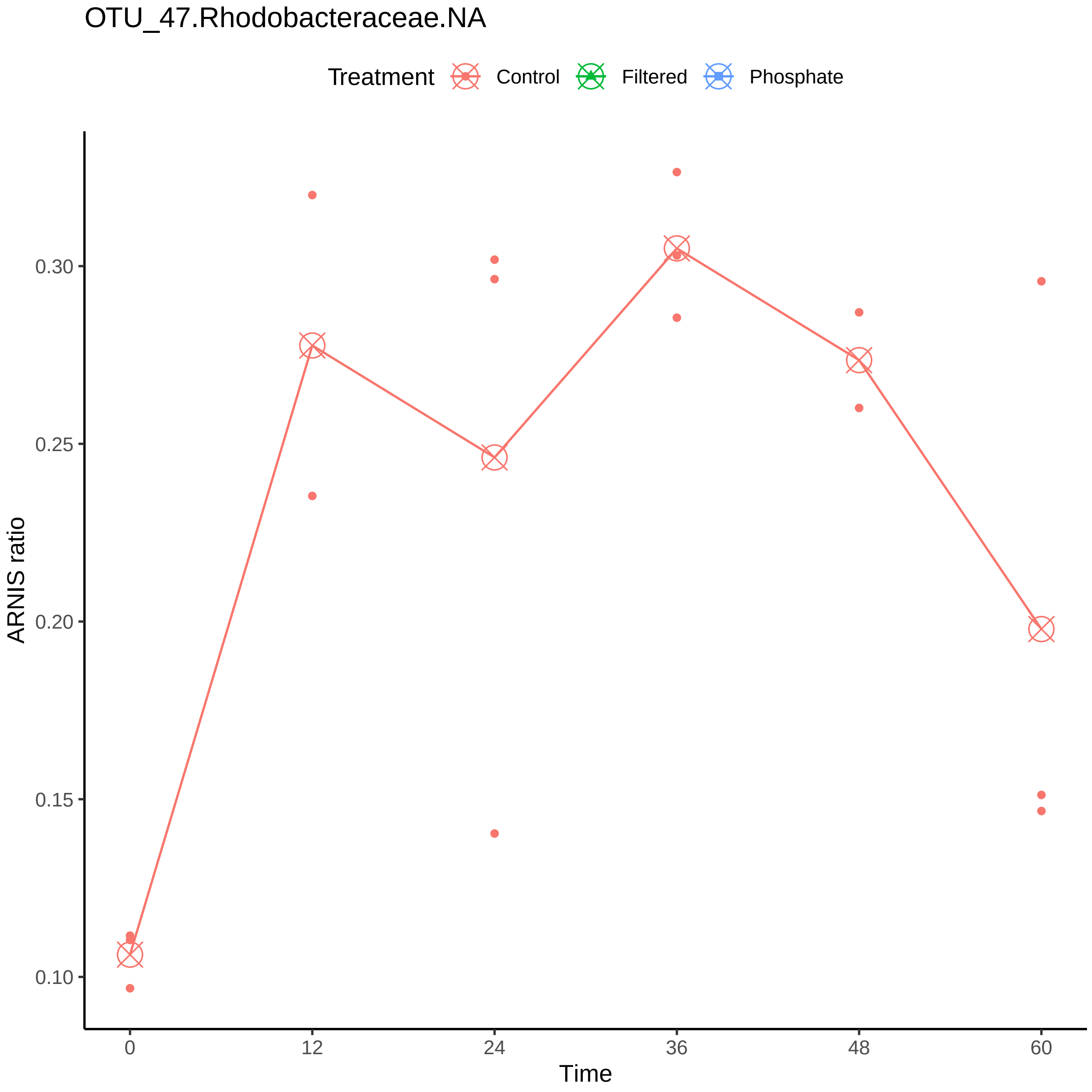
OTU_47.Rhodobacteraceae.NA
Treatment
Control
Filtered
Phosphate
ARNIS ratio
0.10
0.15
0.20
0.25
0.30
0
12
24
36
48
60
Time

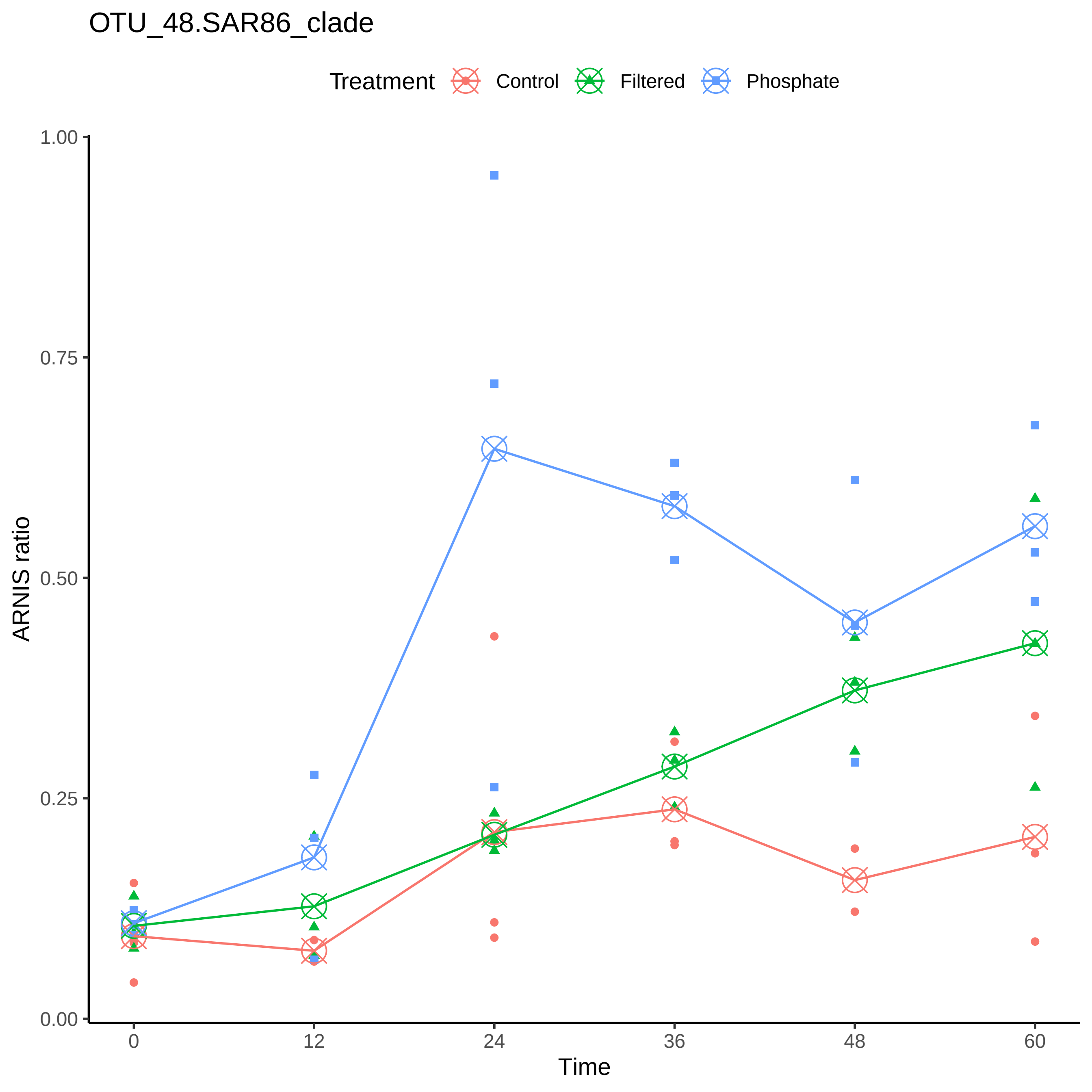

OTU_48.SAR86_clade
Treatment
Control
Filtered
Phosphate
ARNIS ratio
1.00
0.75
0.50
0.25
0.00
0
12
24
36
48
60
Time

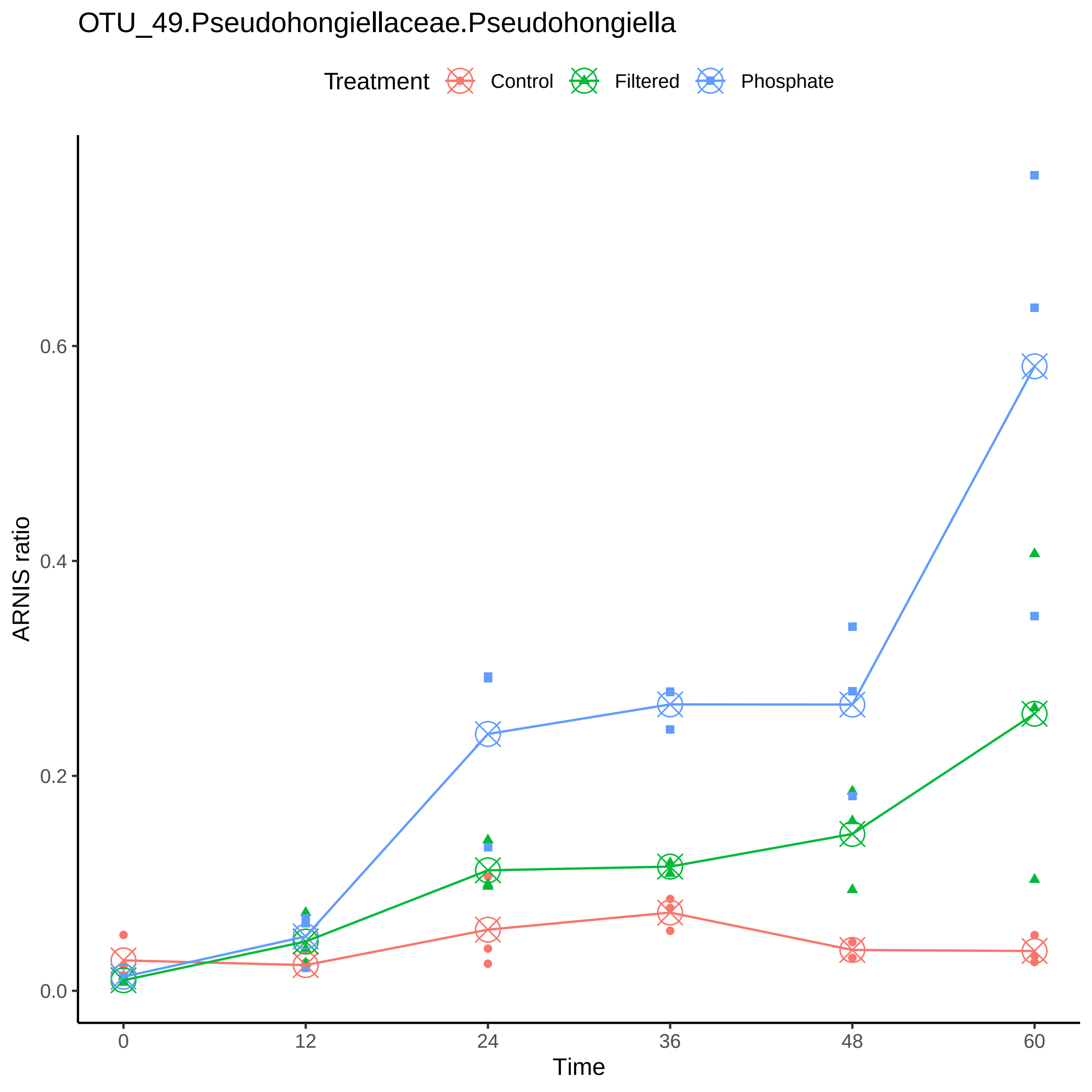

OTU_49.Pseudohongiellaceae.Pseudohongiella
Treatment
Control
Filtered
Phosphate
ARNIS ratio
0.0
0.2
0.4
0.6
Time
0
12
24
36
48
60

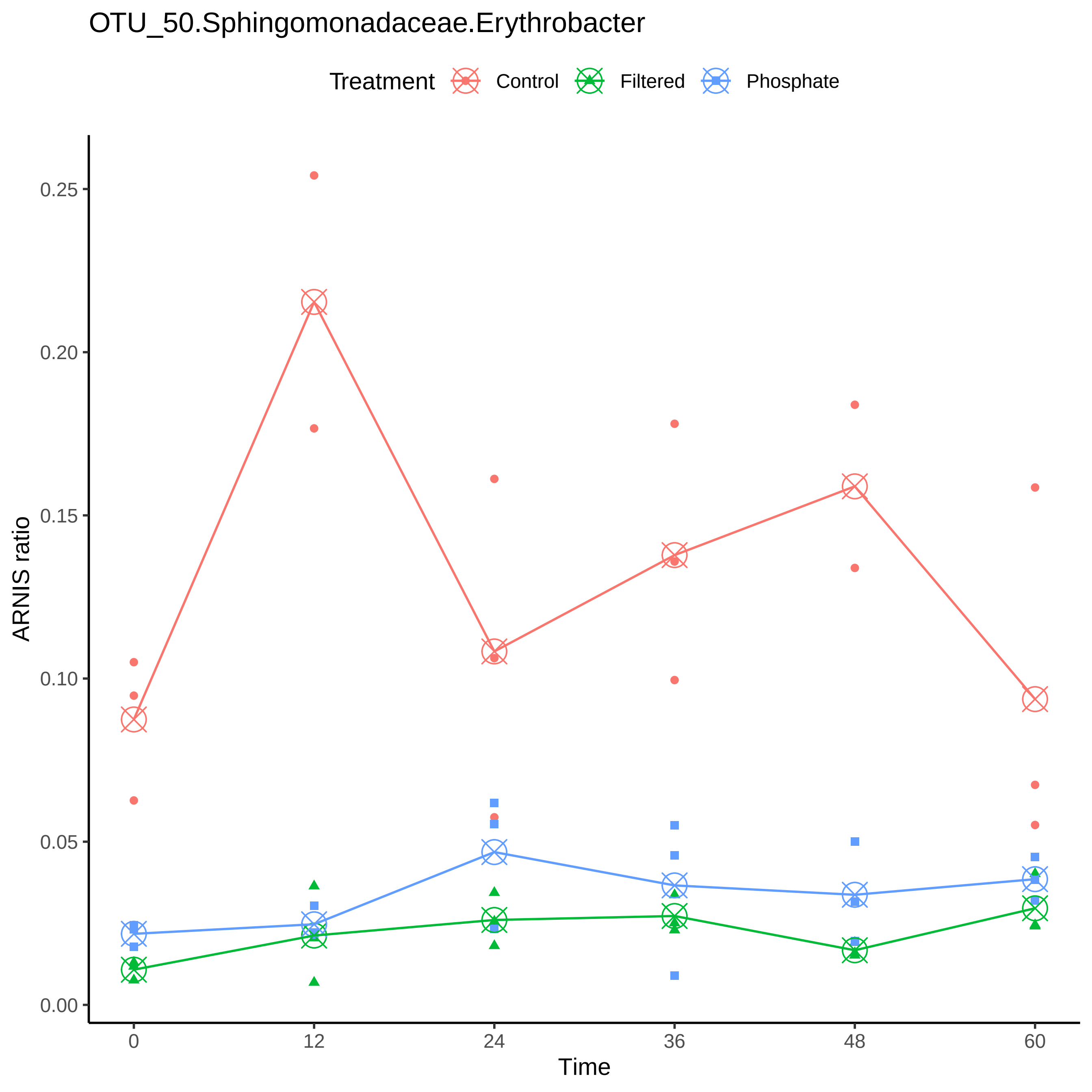
OTU_50.Sphingomonadaceae.Erythrobacter
Treatment
Control
Filtered
Phosphate
ARNIS ratio
0.00
0.05
0.10
0.15
0.20
0.25
Time
0
12
24
36
48
60

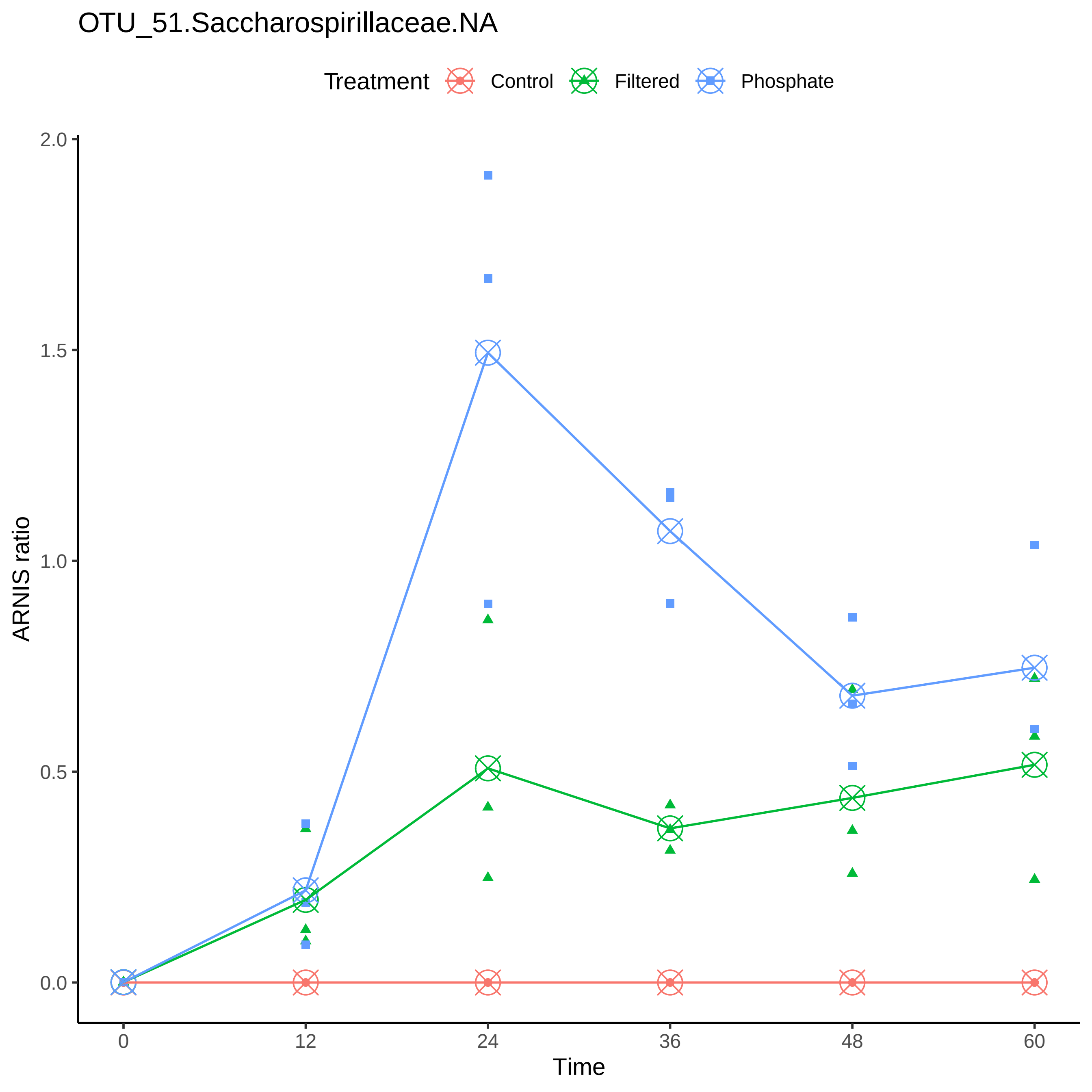
OTU_51.Saccharospirillaceae.NA
Treatment
Control
Filtered
Phosphate
ARNIS ratio
2.0
1.5
1.0
0.5
0.0
0
12
24
36
48
60
Time

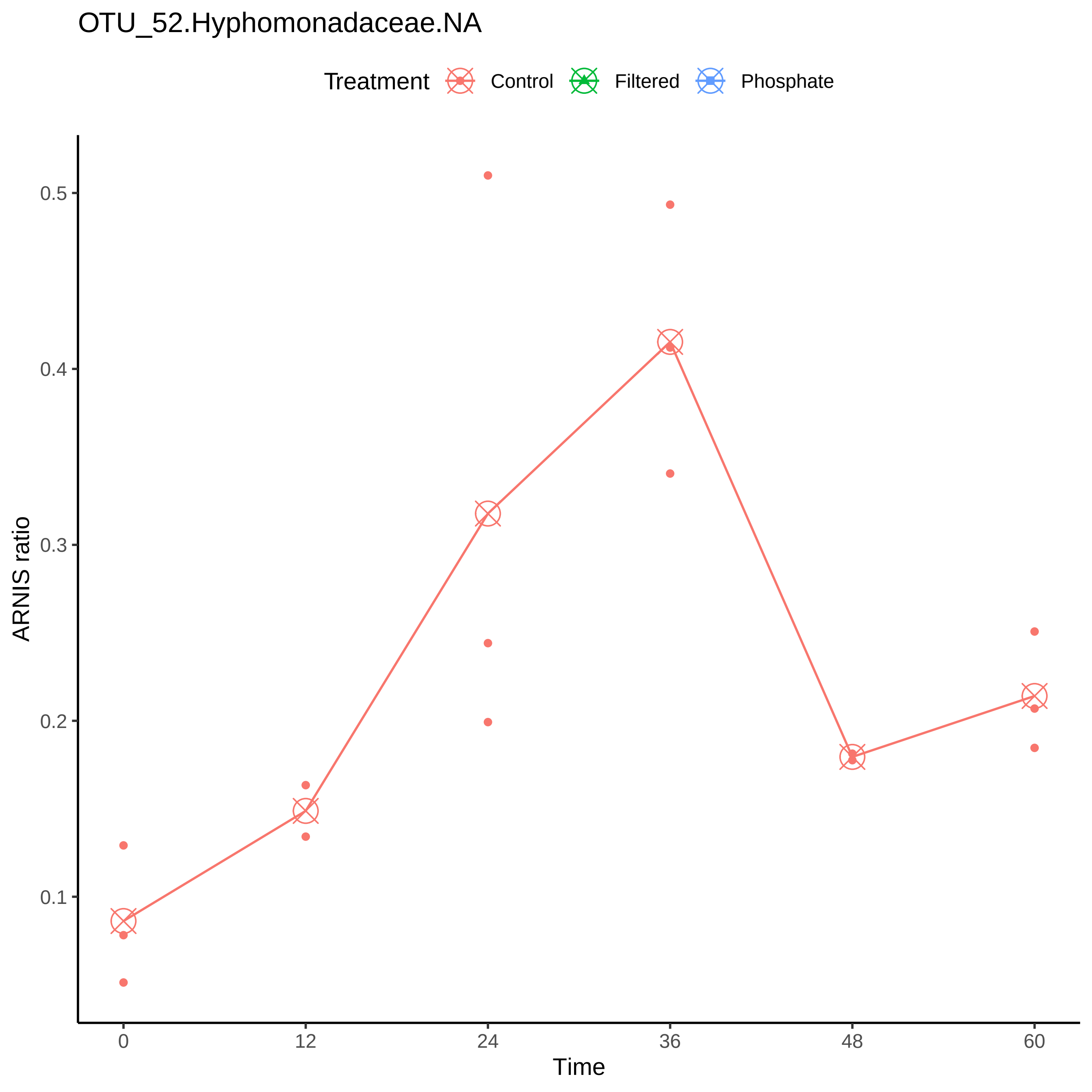

OTU_52.Hyphomonadaceae.NA
Treatment
Control
Filtered
Phosphate
ARNIS ratio
0.5
0.4
0.3
0.2
0.1
0
12
24
36
48
60
Time

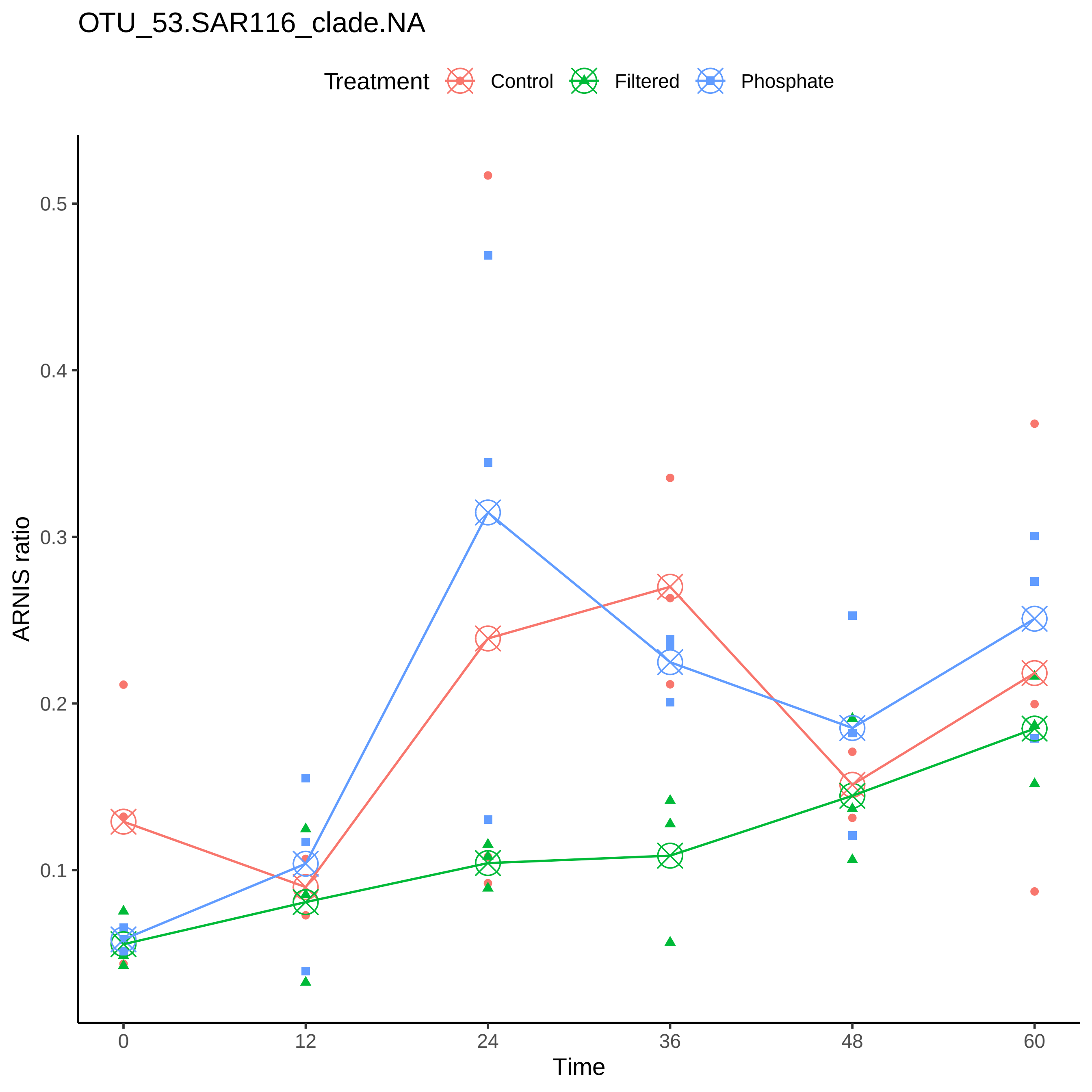
OTU_53.SAR116_clade.NA
Treatment
Control
Filtered
Phosphate
ARNIS ratio
0.5
0.4
0.3
0.2
0.1
0
12
24
36
48
60
Time

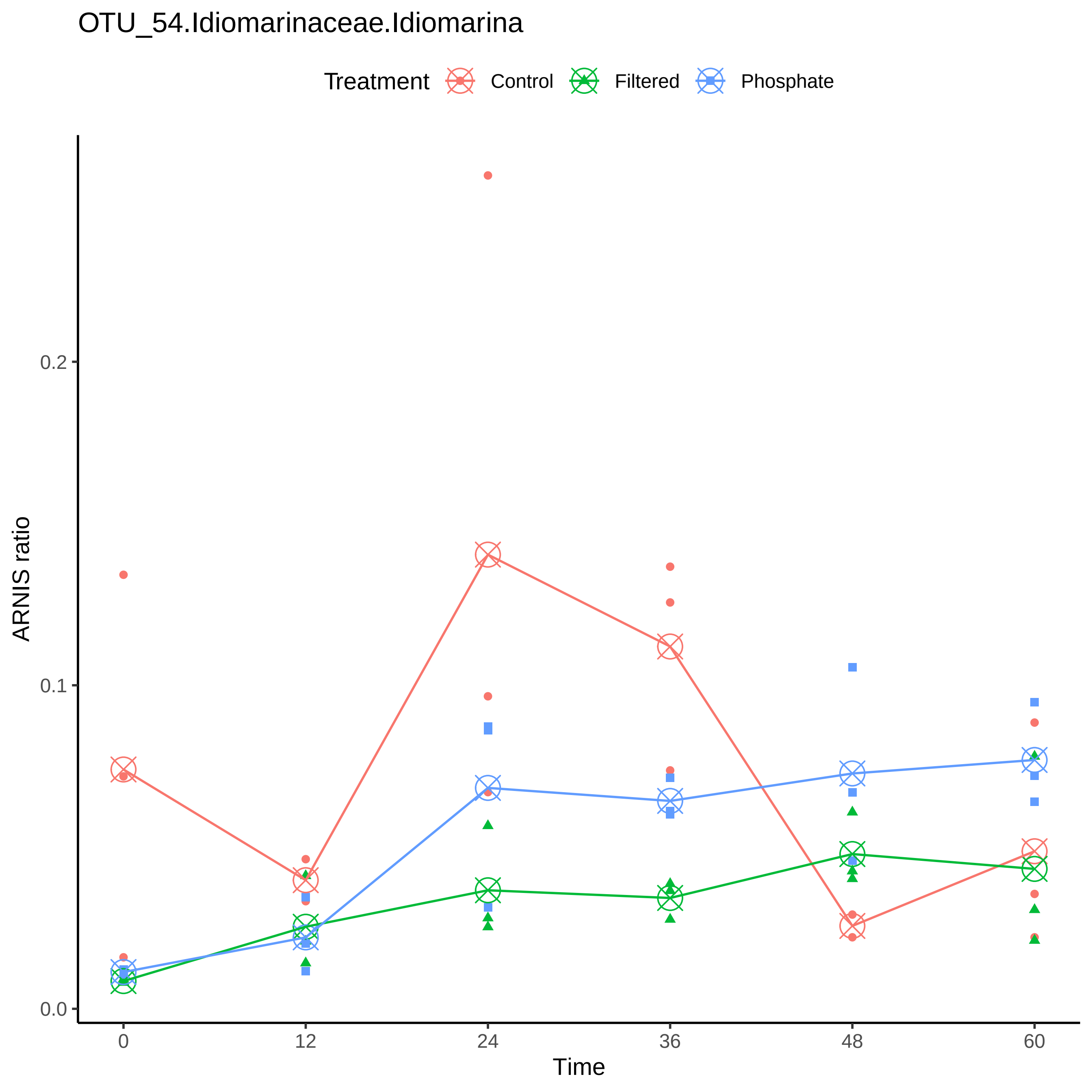

OTU_54.Idiomarinaceae.Idiomarina
Treatment
Control
Filtered
Phosphate
ARNIS ratio
0.2
0.1
0.0
0
12
24
36
48
60
Time

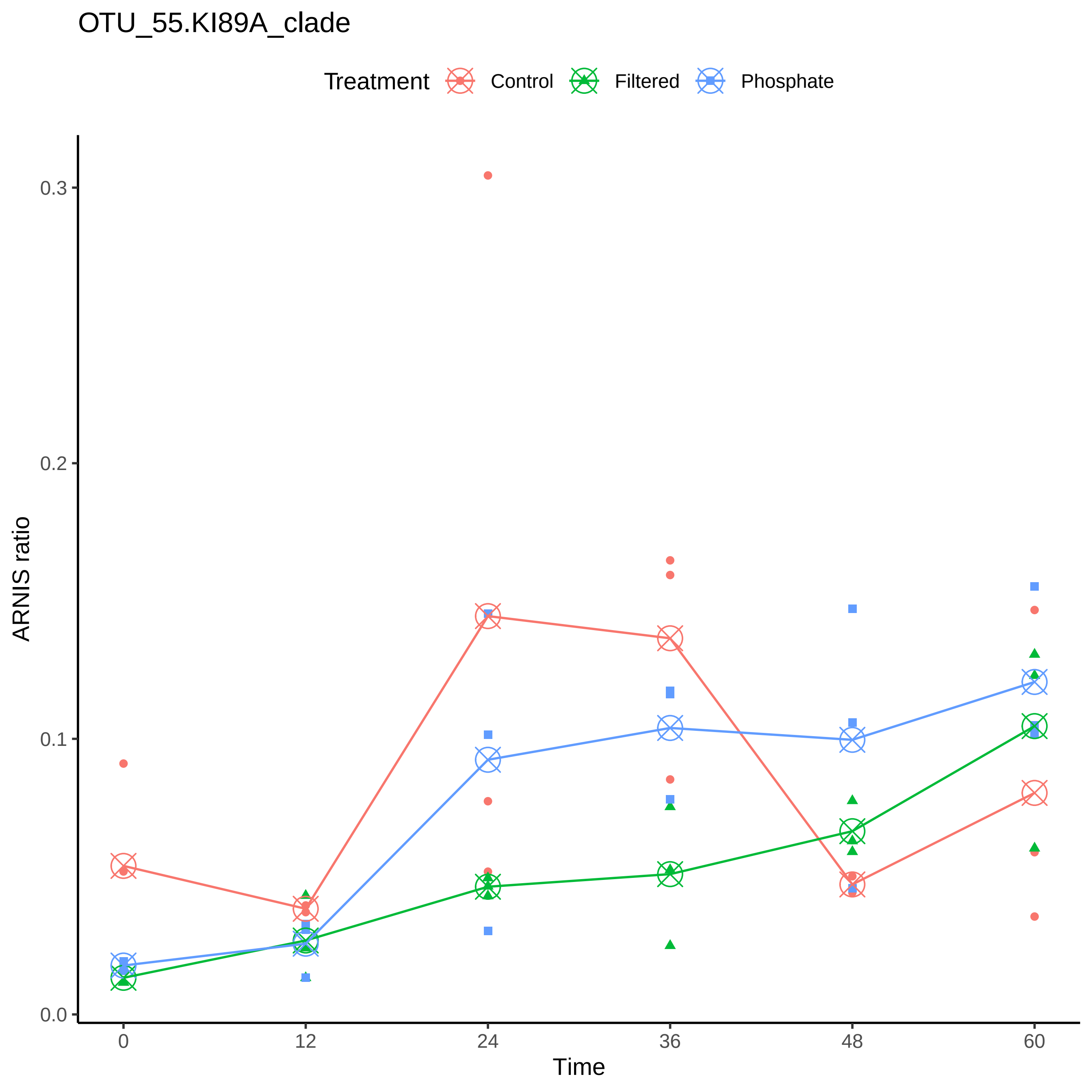
OTU_55.KI89A_clade
Treatment
Control
Filtered
Phosphate
ARNIS ratio
0.0
0.1
0.2
0.3
Time
0
12
24
36
48
60

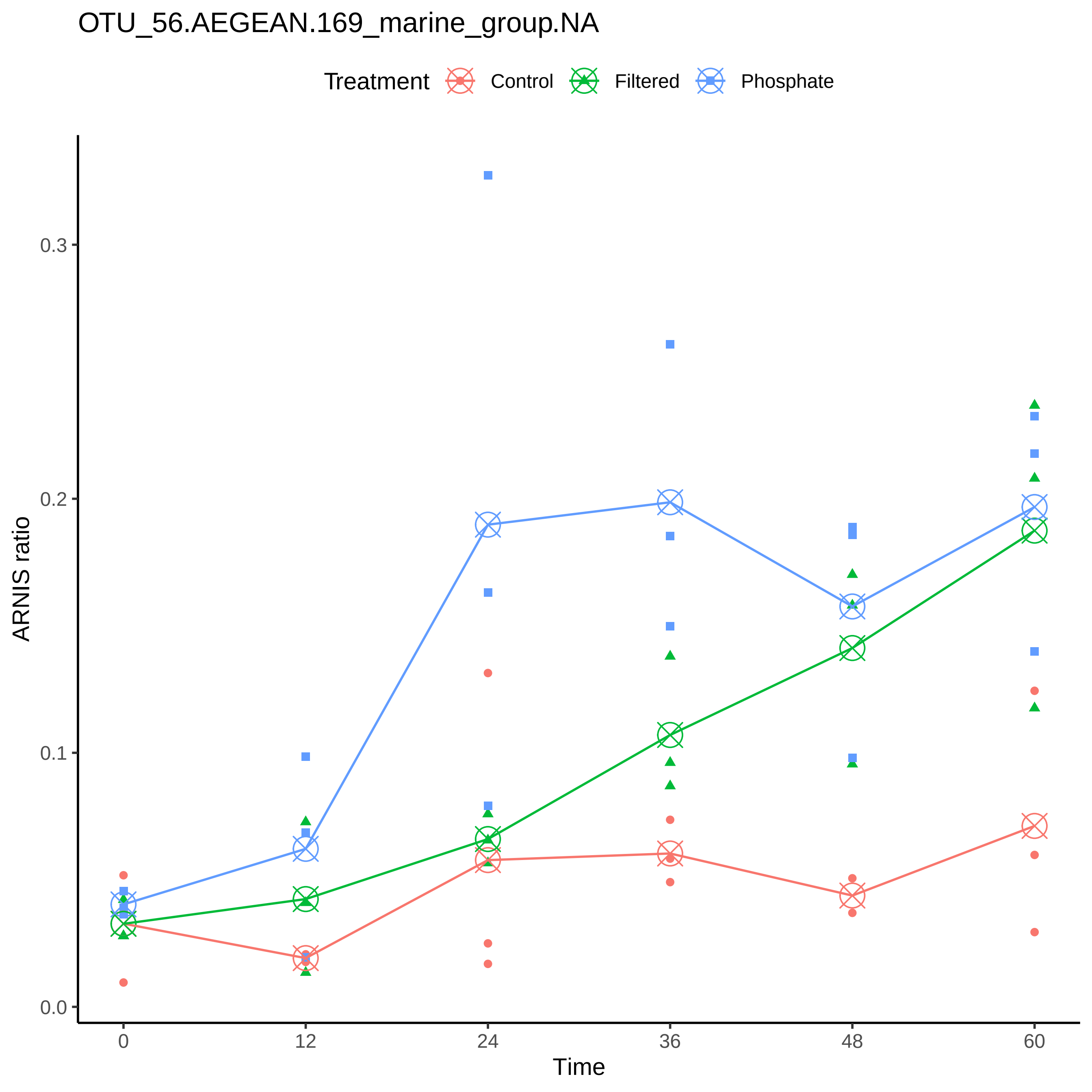
OTU_56.AEGEAN.169_marine_group.NA
Treatment
Control
Filtered
Phosphate
ARNIS ratio
0.0
0.1
0.2
0.3
Time
0
12
24
36
48
60

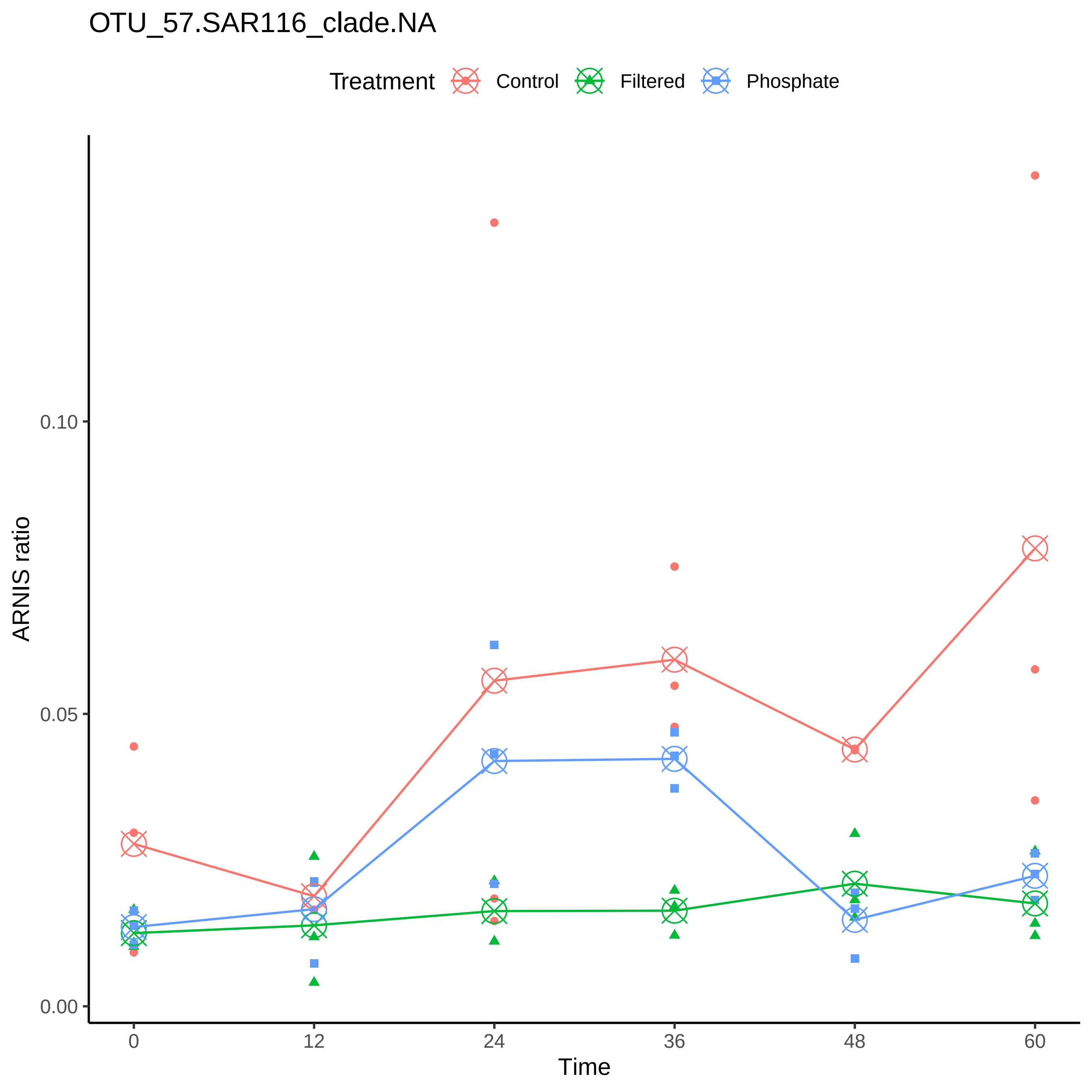
OTU_57.SAR116_clade.NA
Treatment
Control
Filtered
Phosphate
ARNIS ratio
0.10
0.05
0.00
0
12
24
36
48
60
Time

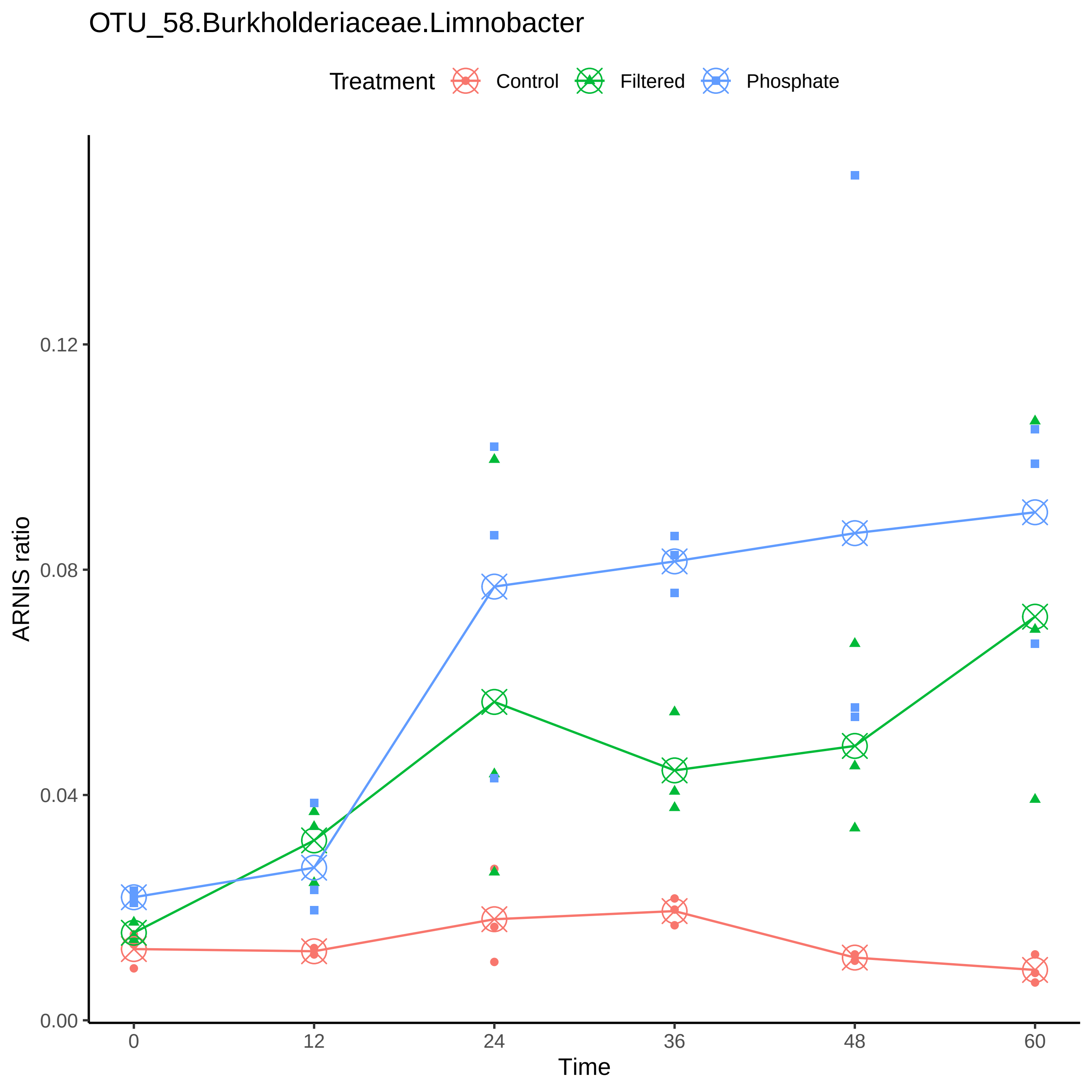

OTU_58.Burkholderiaceae.Limnobacter
Treatment
Control
Filtered
Phosphate
ARNIS ratio
0.00
0.04
0.08
0.12
Time
0
12
24
36
48
60

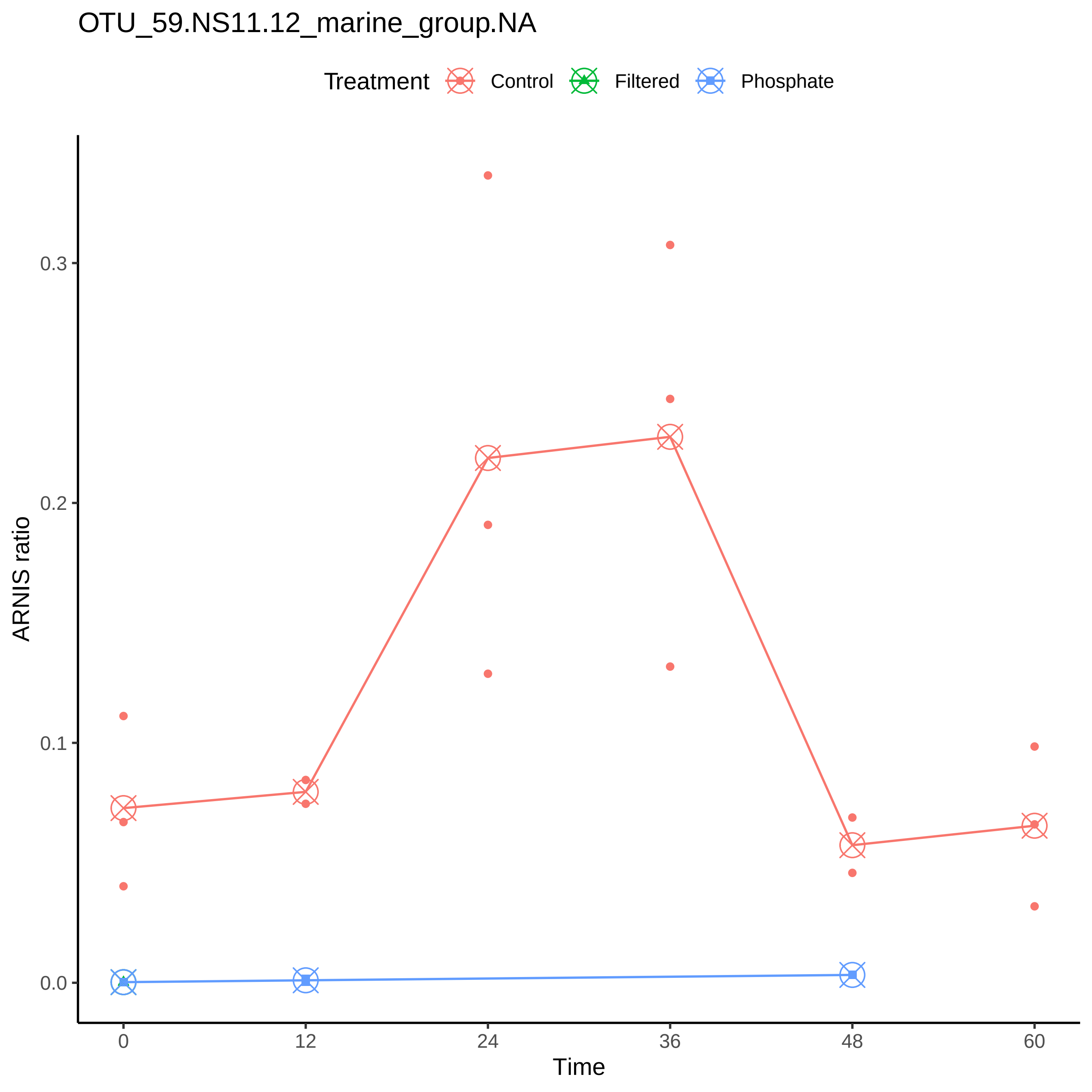

OTU_59.NS11.12_marine_group.NA
Treatment
Control
Filtered
Phosphate
ARNIS ratio
0.0
0.1
0.2
0.3
Time
0
12
24
36
48
60

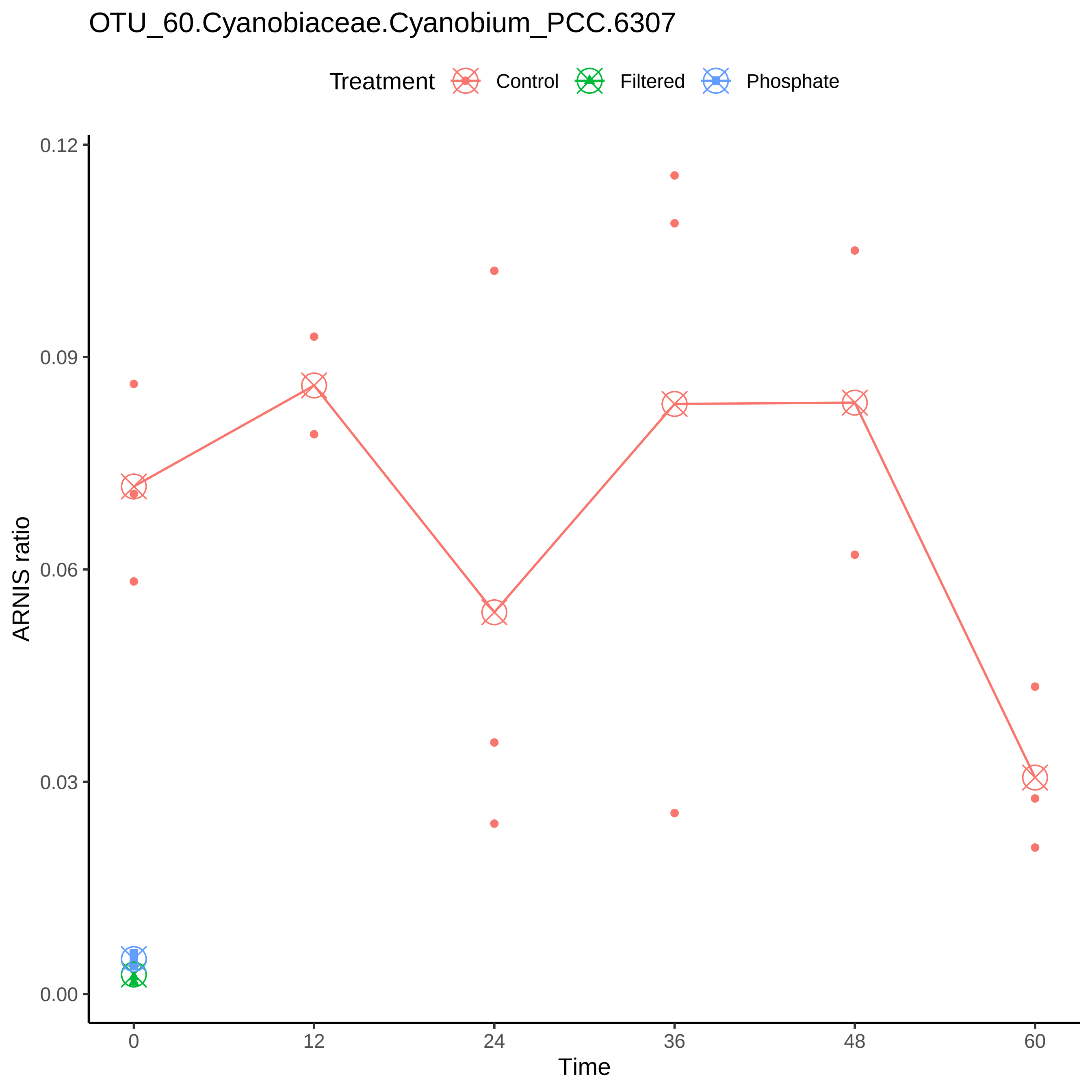
OTU_60.Cyanobiaceae.Cyanobium_PCC.6307
Treatment
Control
Filtered
Phosphate
0.12
0.09
0.06
0.03
0.00
ARNIS ratio
0
12
24
36
48
60
Time

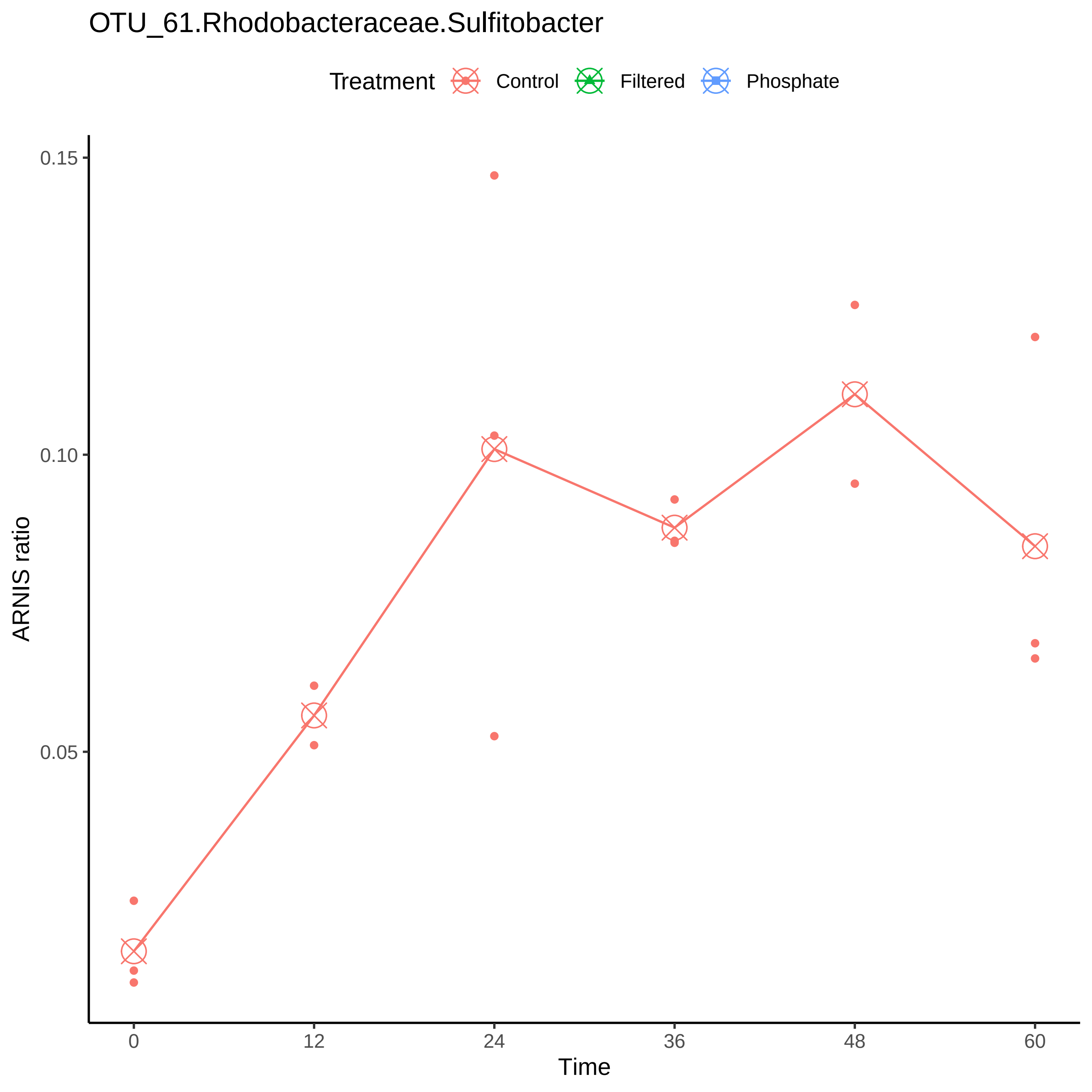

OTU_61.Rhodobacteraceae.Sulfitobacter
Treatment
Control
Filtered
Phosphate
ARNIS ratio
0.15
0.10
0.05
0
12
24
36
48
60
Time

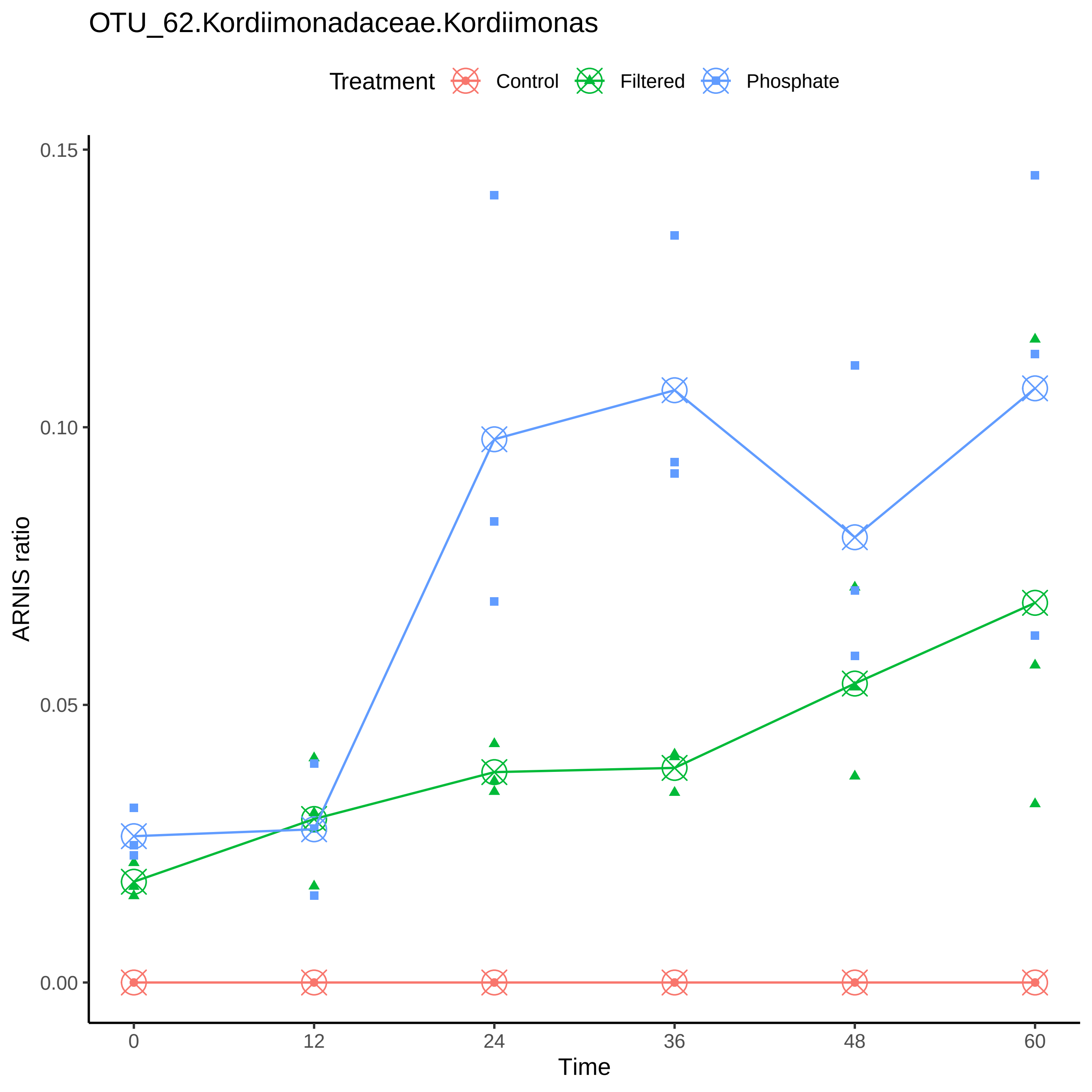
OTU_62.Kordiimonadaceae.Kordiimonas
Treatment
Control
Filtered
Phosphate
ARNIS ratio
0.15
0.10
0.05
0.00
Time
0
12
24
36
48
60

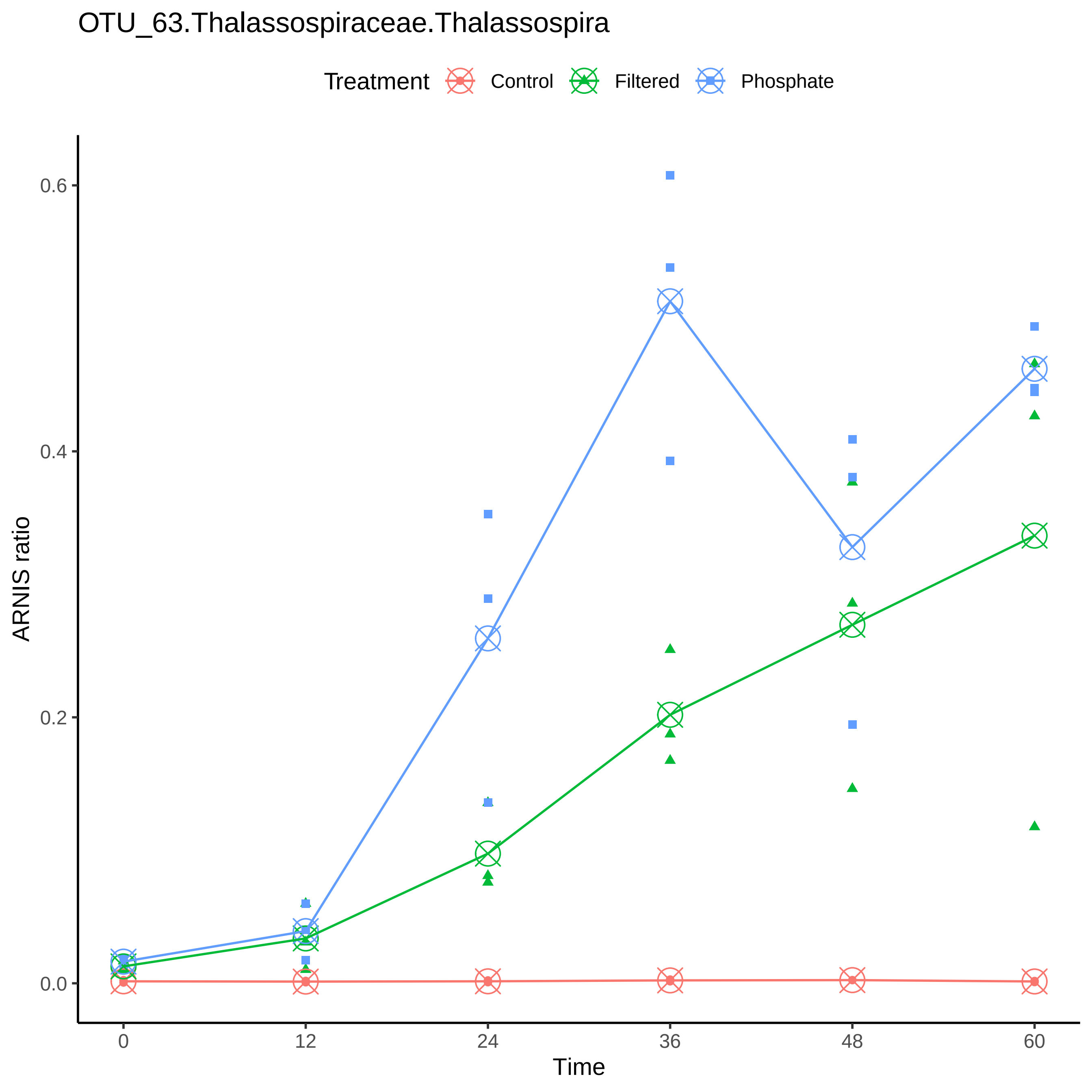

OTU_63.Thalassospiraceae.Thalassospira
Treatment
Control
Filtered
Phosphate
ARNIS ratio
0.0
0.2
0.4
0.6
Time
0
12
24
36
48
60

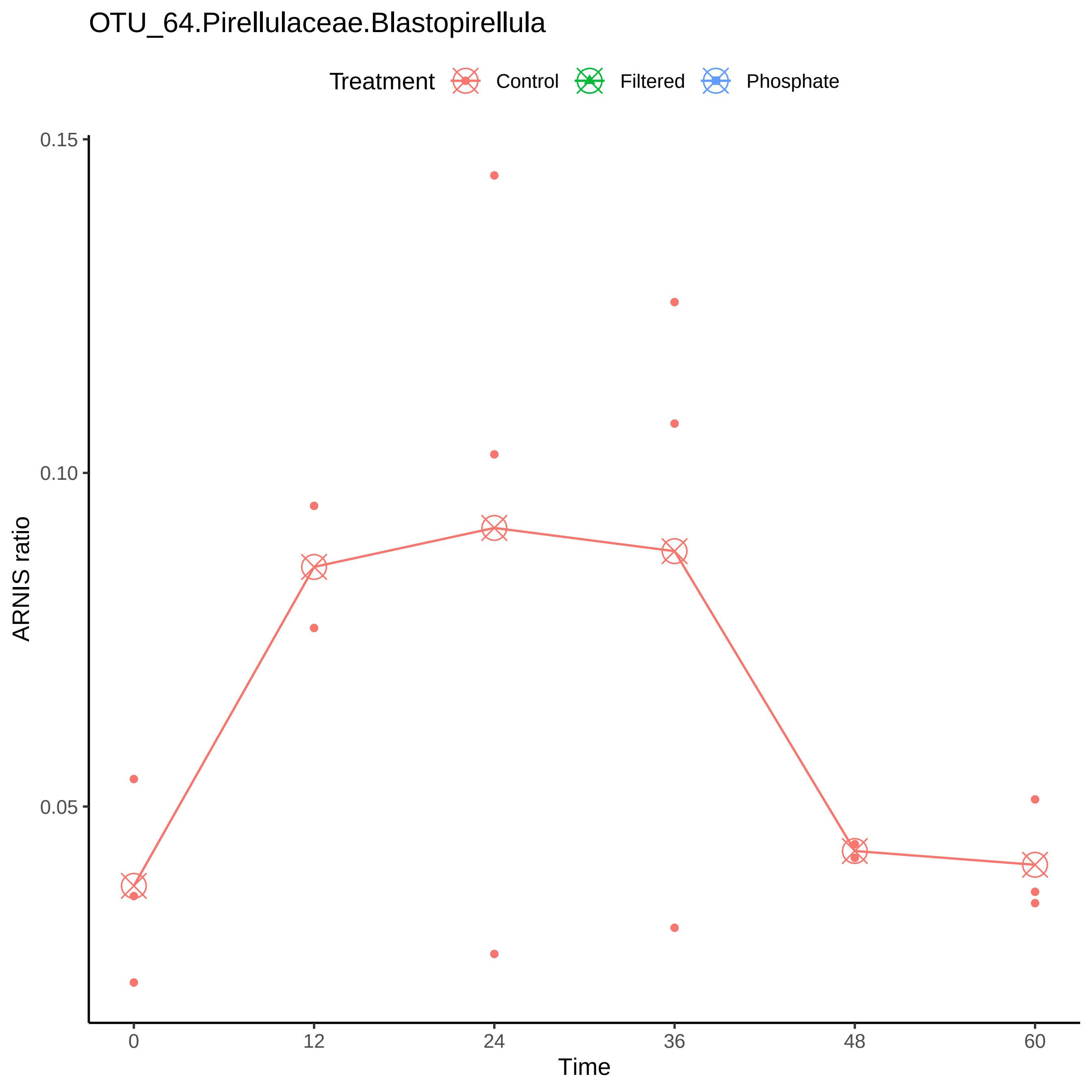
OTU_64.Pirellulaceae.Blastopirellula
Treatment
Control
Filtered
Phosphate
0.15
0.10
0.05
ARNIS ratio
0
12
24
36
48
60
Time

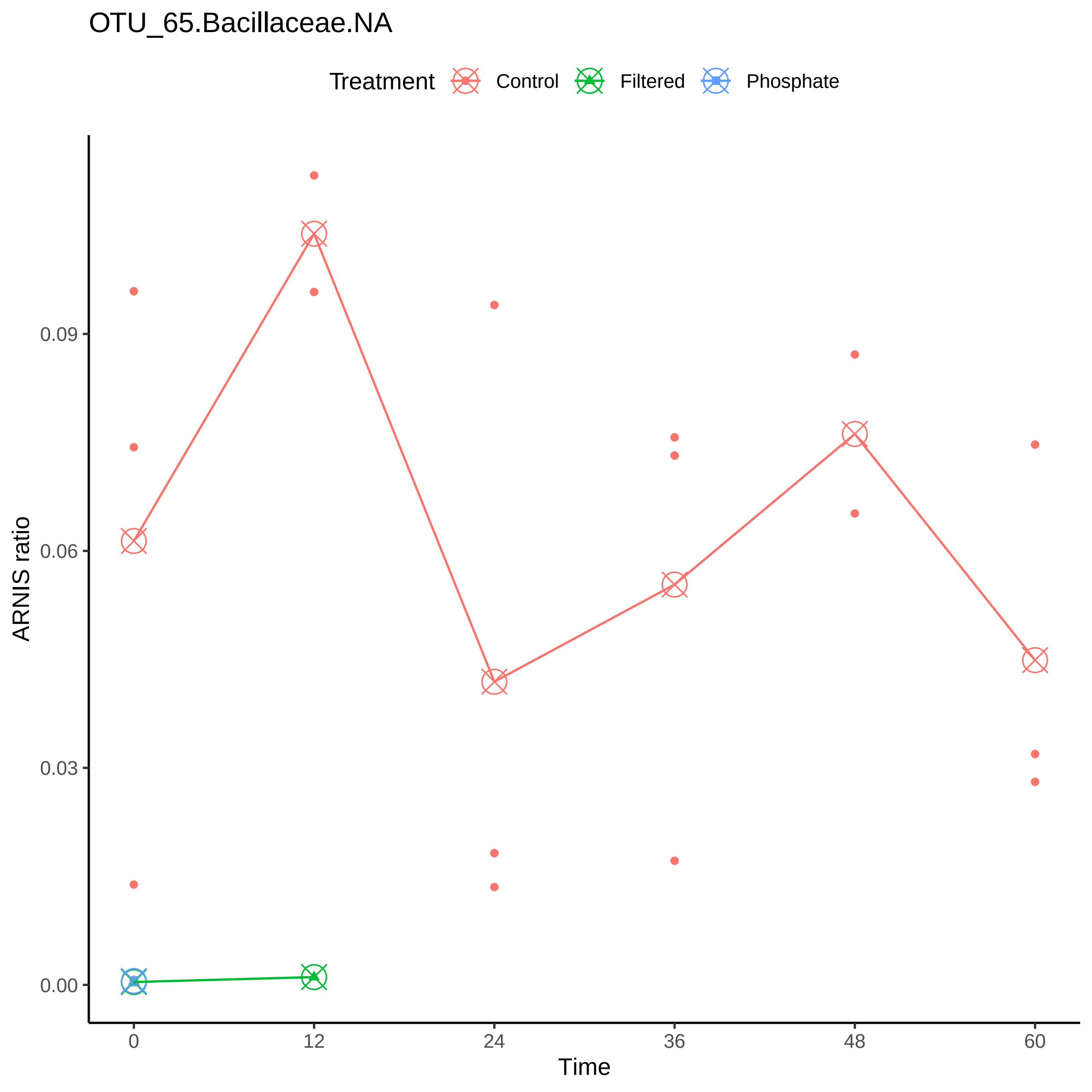
OTU_65.Bacillaceae.NA
Treatment
Control
Filtered
Phosphate
ARNIS ratio
0.09
0.06
0.03
0.00
0
12
24
36
48
60
Time

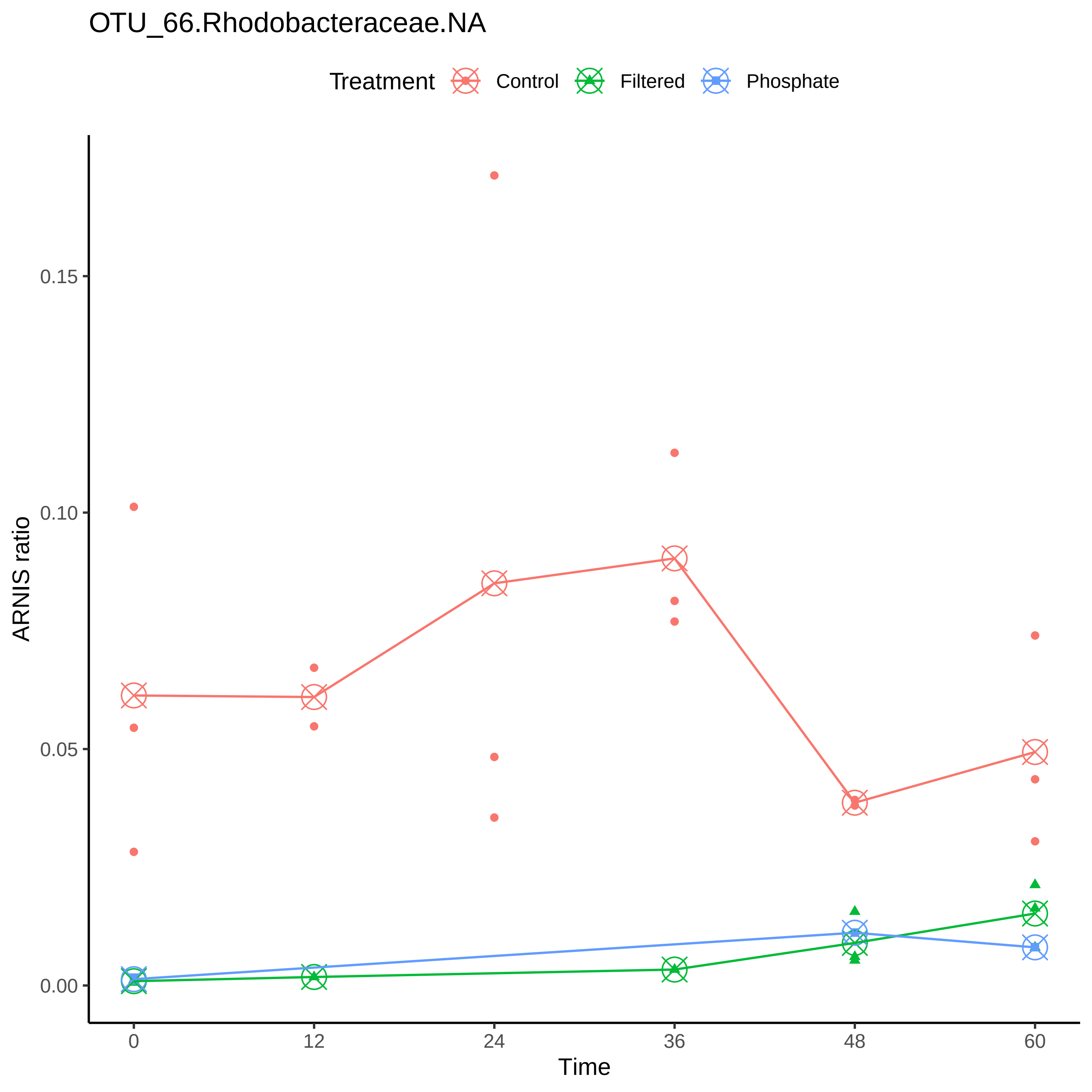
OTU_66.Rhodobacteraceae.NA
Treatment
Control
Filtered
Phosphate
ARNIS ratio
0.15
0.10
0.05
0.00
0
12
24
36
48
60
Time

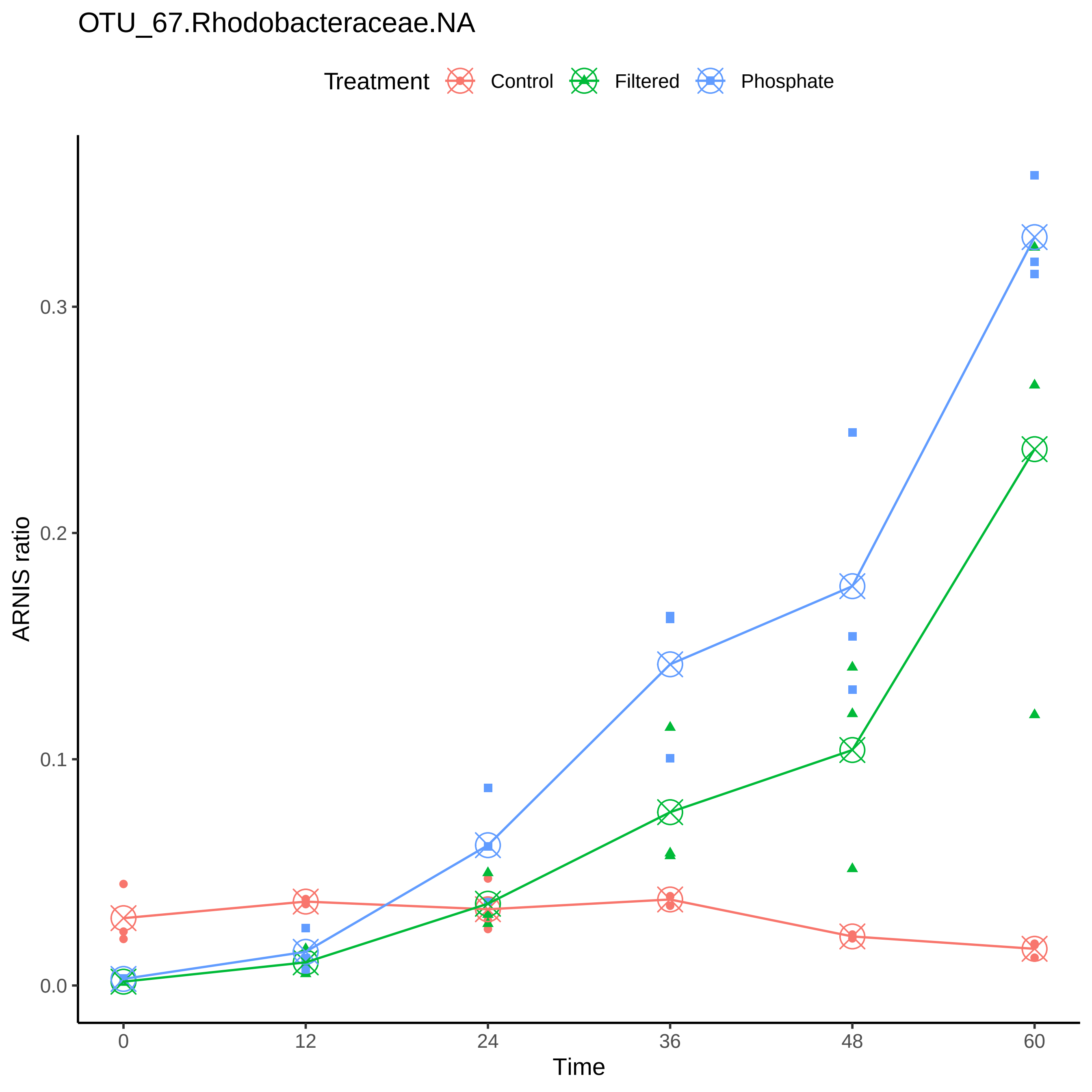
OTU_67.Rhodobacteraceae.NA
Treatment
Control
Filtered
Phosphate
ARNIS ratio
0.0
0.1
0.2
0.3
Time
0
12
24
36
48
60

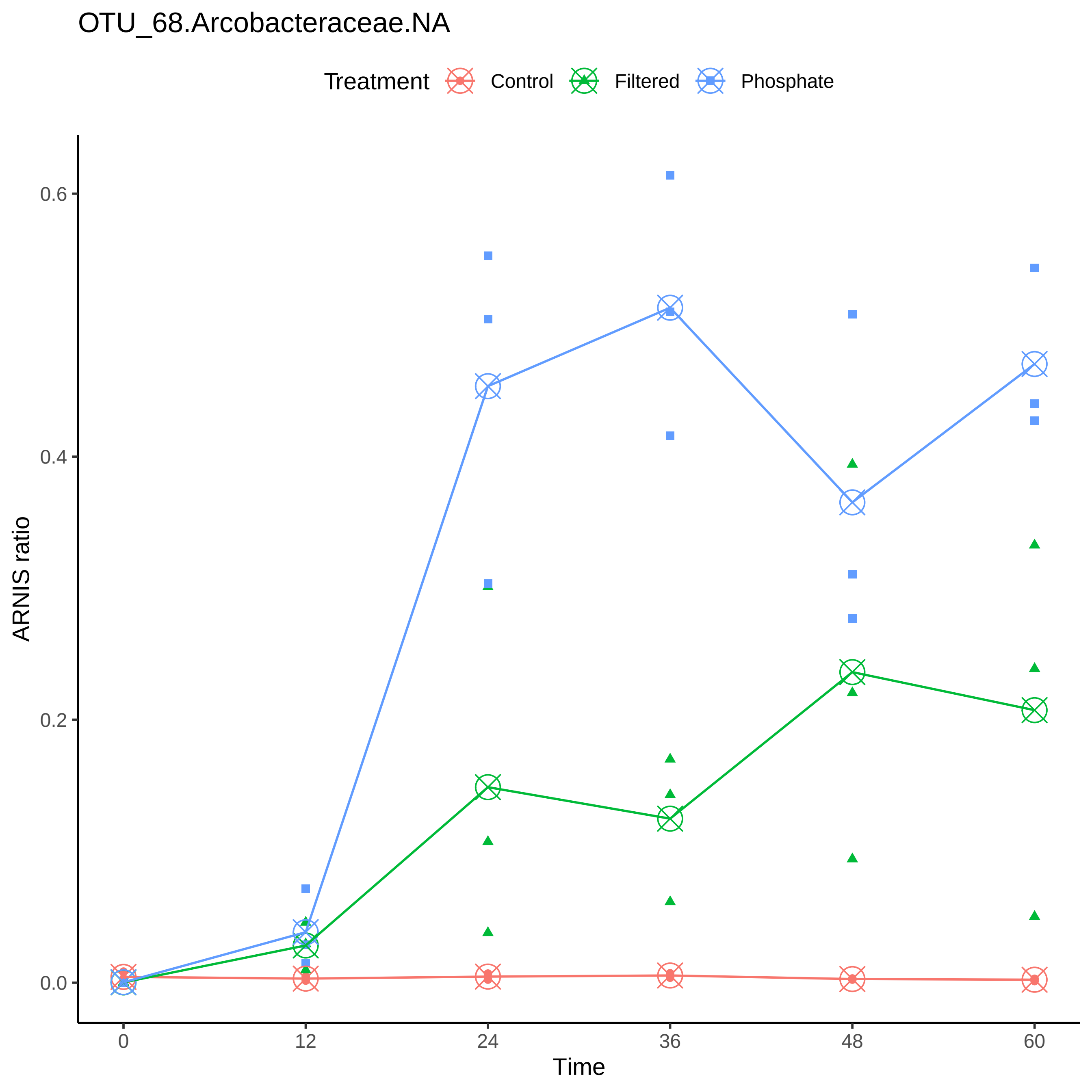
OTU_68.Arcobacteraceae.NA
Treatment
Control
Filtered
Phosphate
ARNIS ratio
0.6
0.4
0.2
0.0
0
12
24
36
48
60
Time

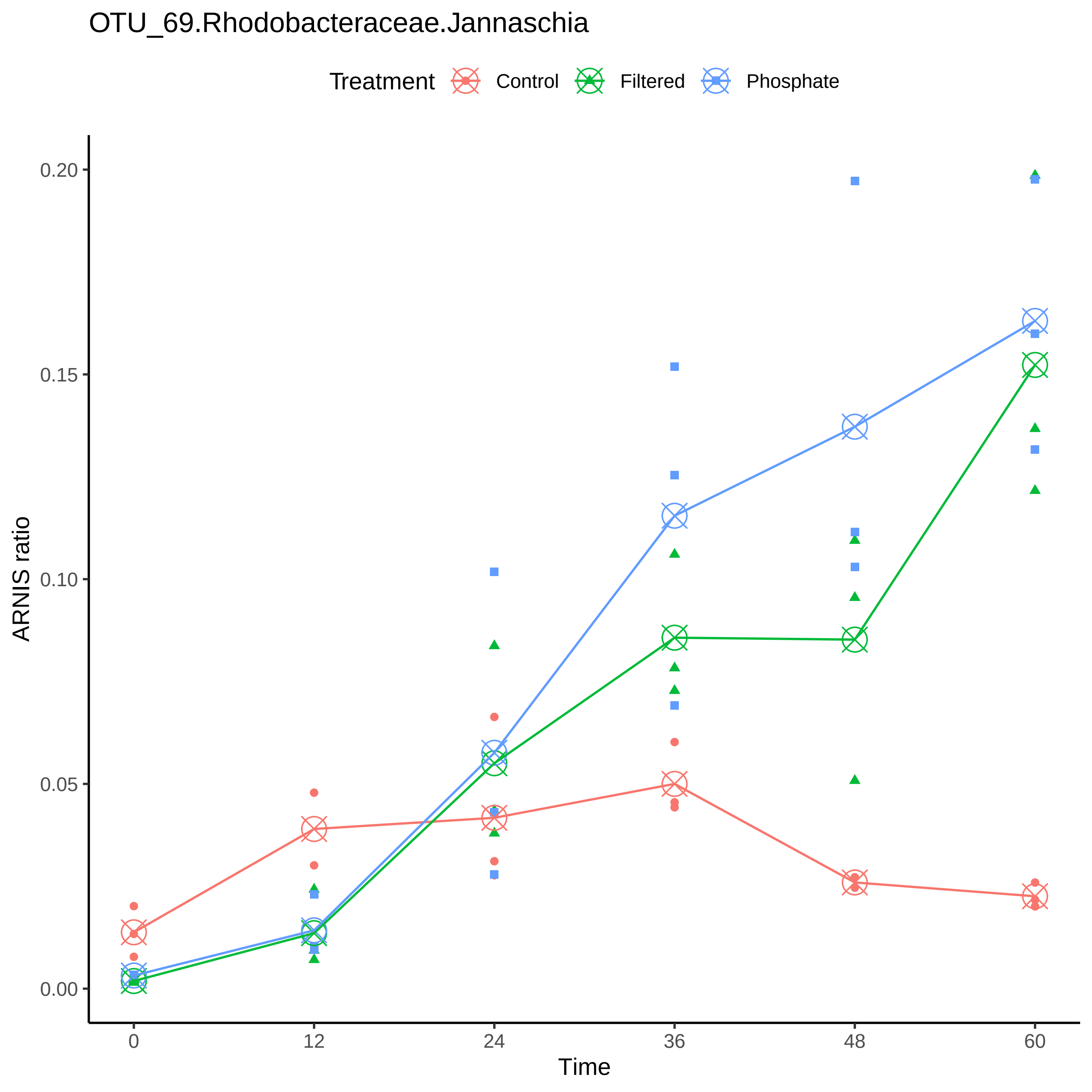

OTU_69.Rhodobacteraceae.Jannaschia
Treatment
Control
Filtered
Phosphate
ARNIS ratio
0.00
0.05
0.10
0.15
0.20
0
12
24
36
48
60
Time

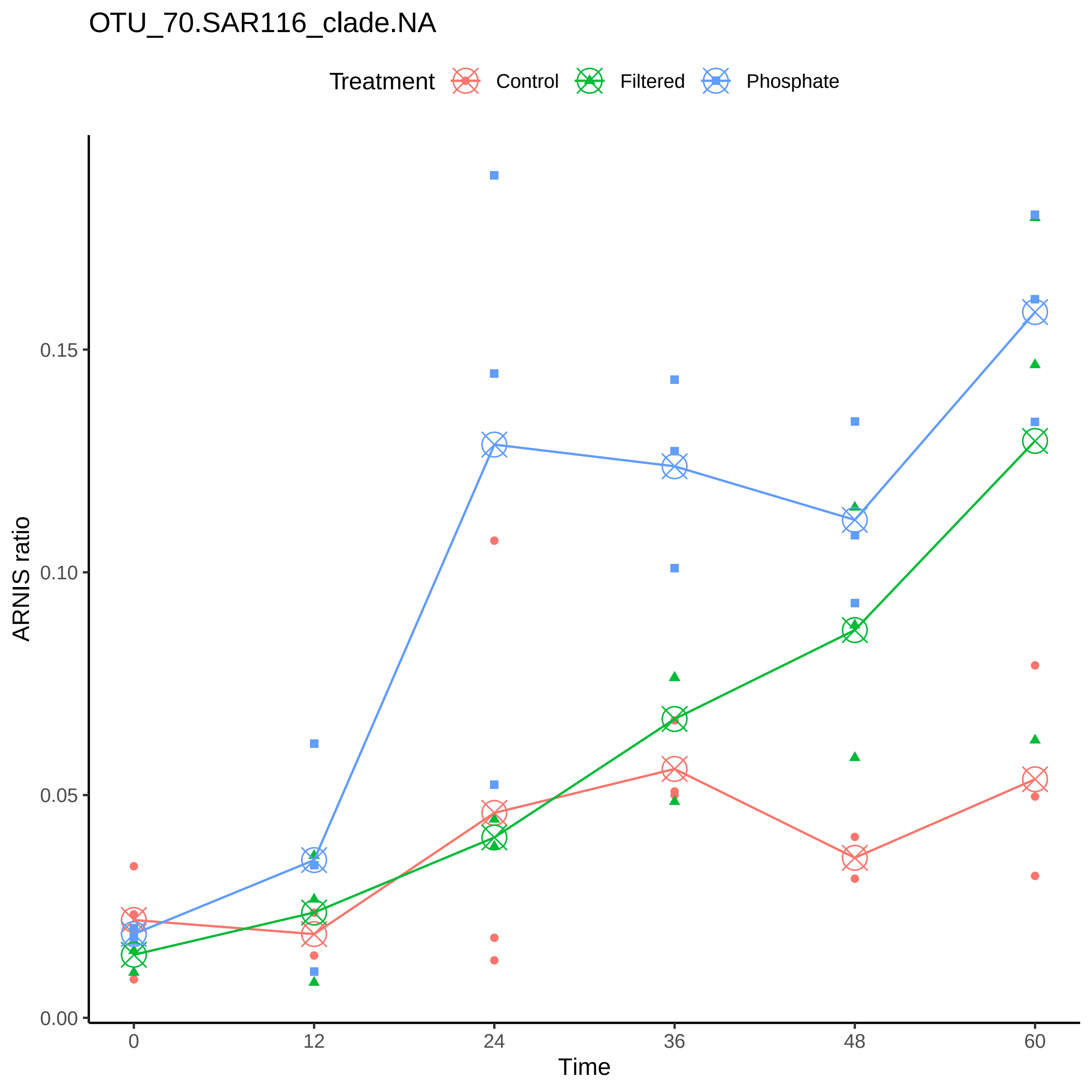

OTU_70.SAR116_clade.NA
Treatment
Control
Filtered
Phosphate
ARNIS ratio
0.00
0.05
0.10
0.15
Time
0
12
24
36
48
60

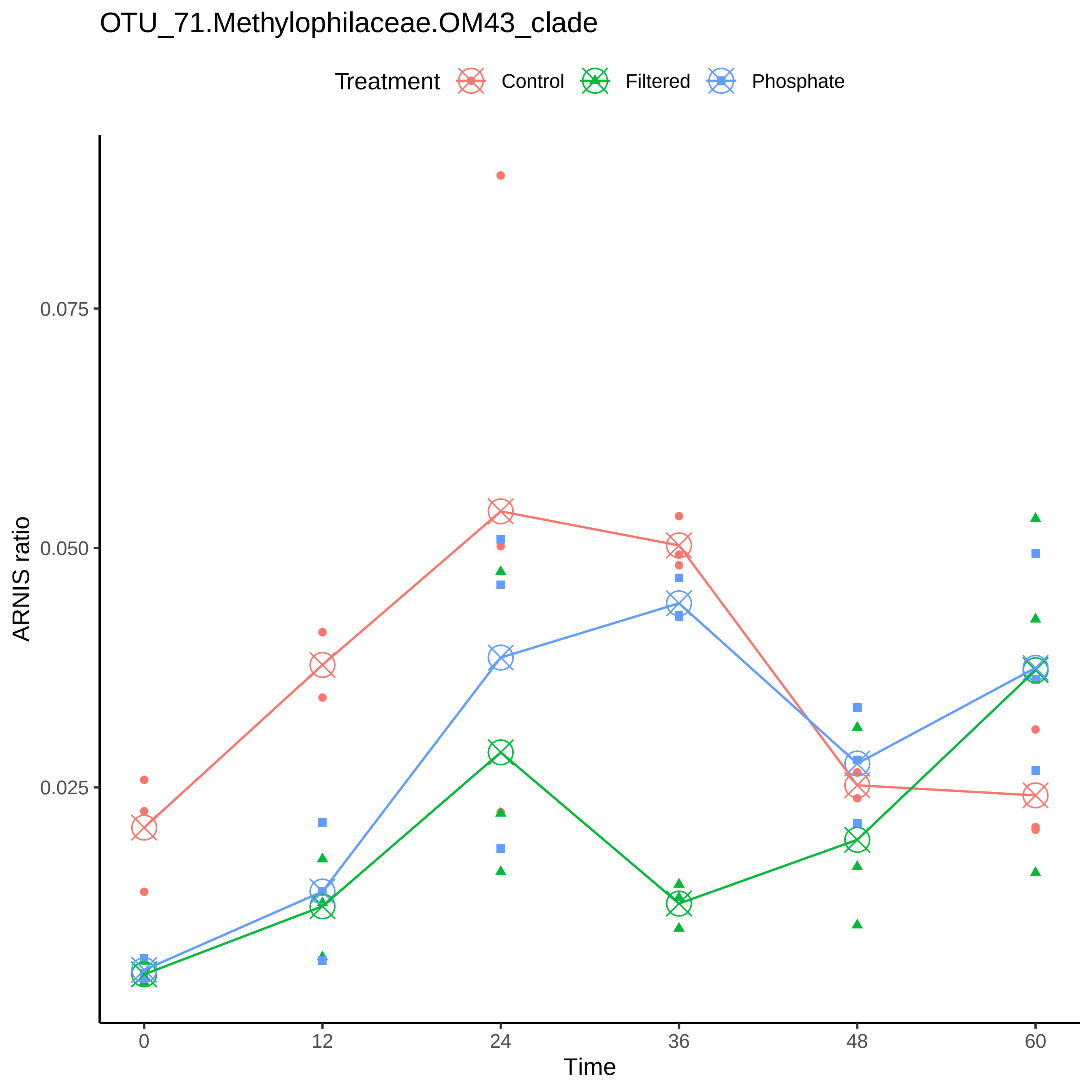
OTU_71.Methylophilaceae.OM43_clade
Treatment
Control
Filtered
Phosphate
ARNIS ratio
0.075
0.050
0.025
Time
0
12
24
36
48
60

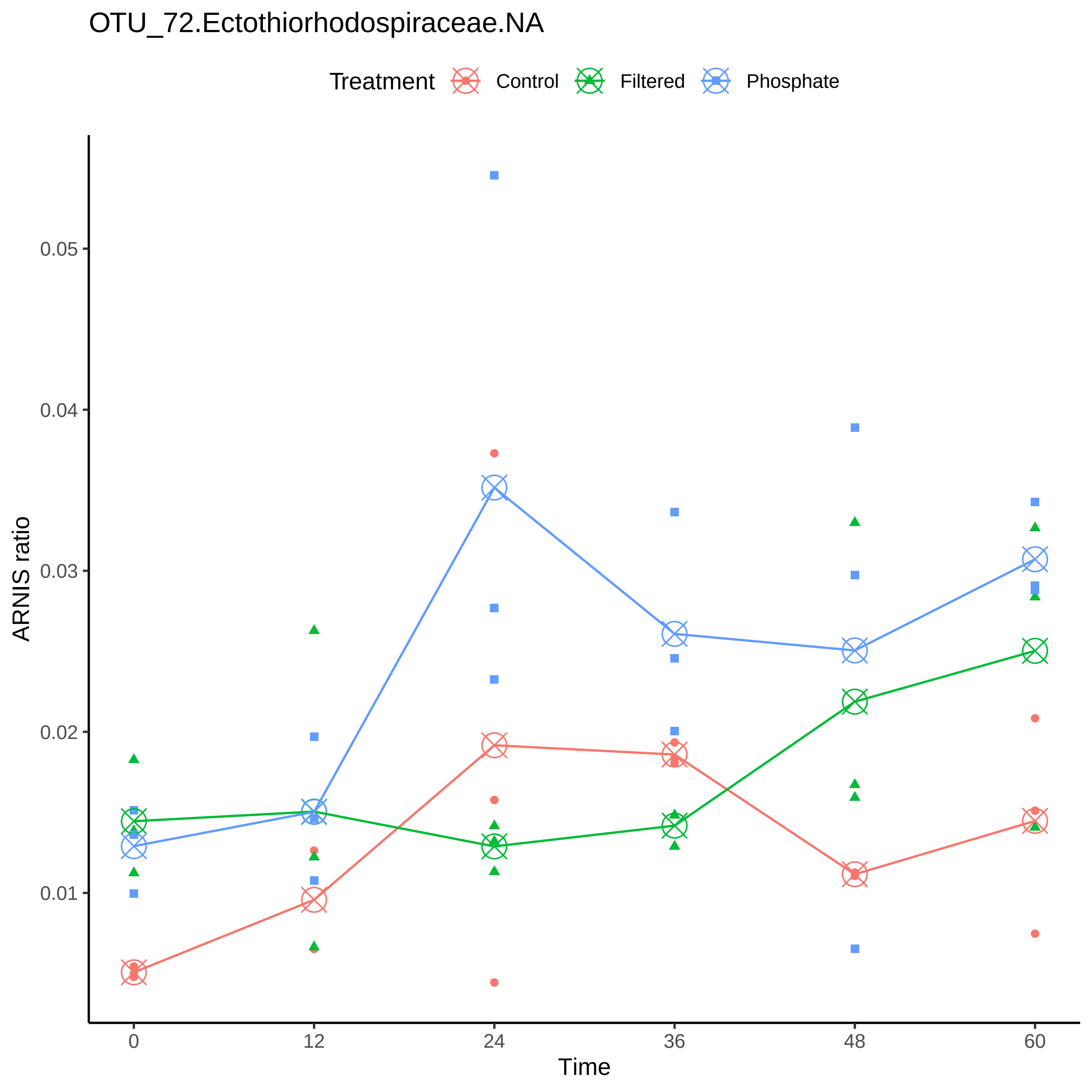
OTU_72.Ectothiorhodospiraceae.NA
Treatment
Control
Filtered
Phosphate
ARNIS ratio
0.05
0.04
0.03
0.02
0.01
0
12
24
36
48
60
Time

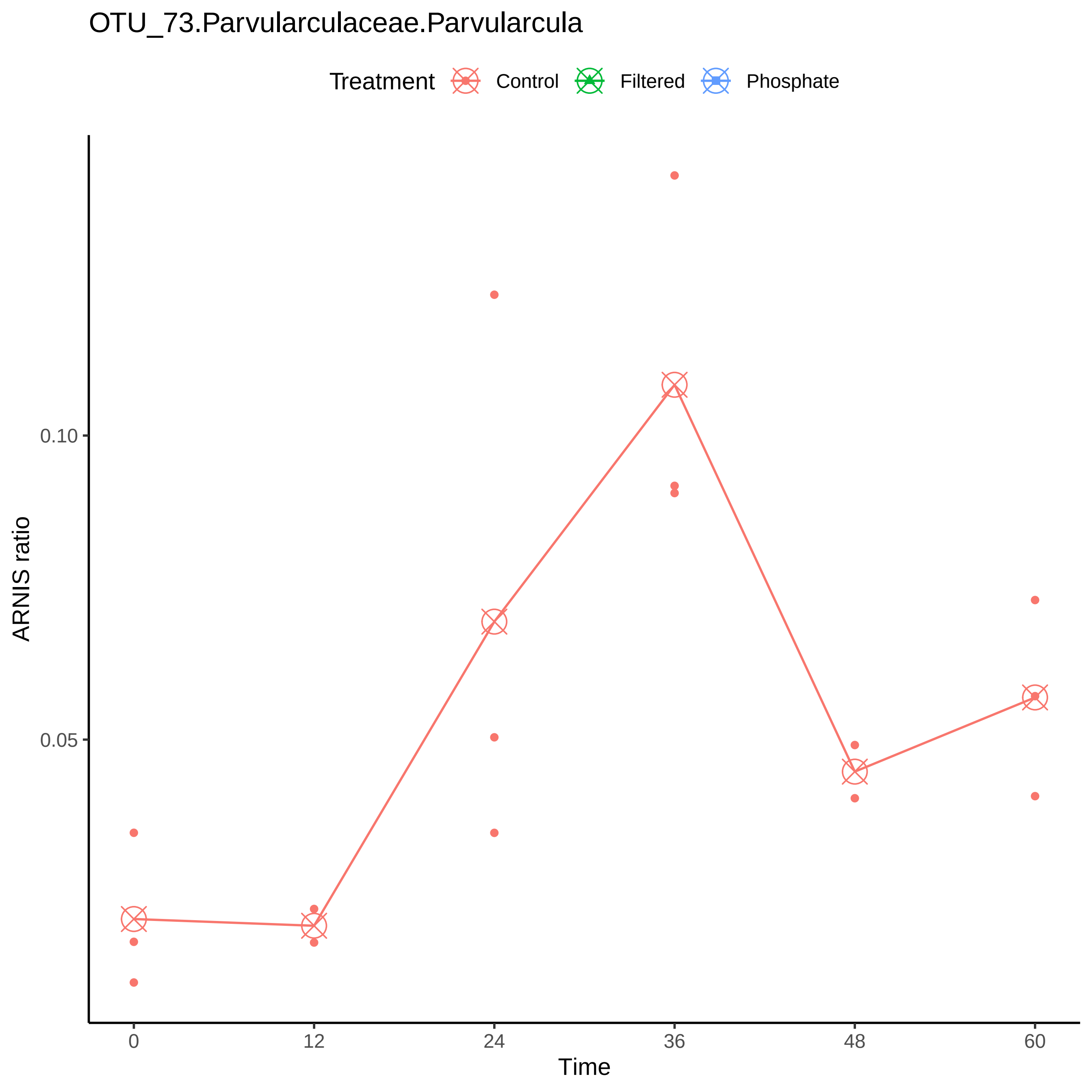

OTU_73.Parvularculaceae.Parvularcula
Treatment
Control
Filtered
Phosphate
ARNIS ratio
0.10
0.05
0
12
24
36
48
60
Time

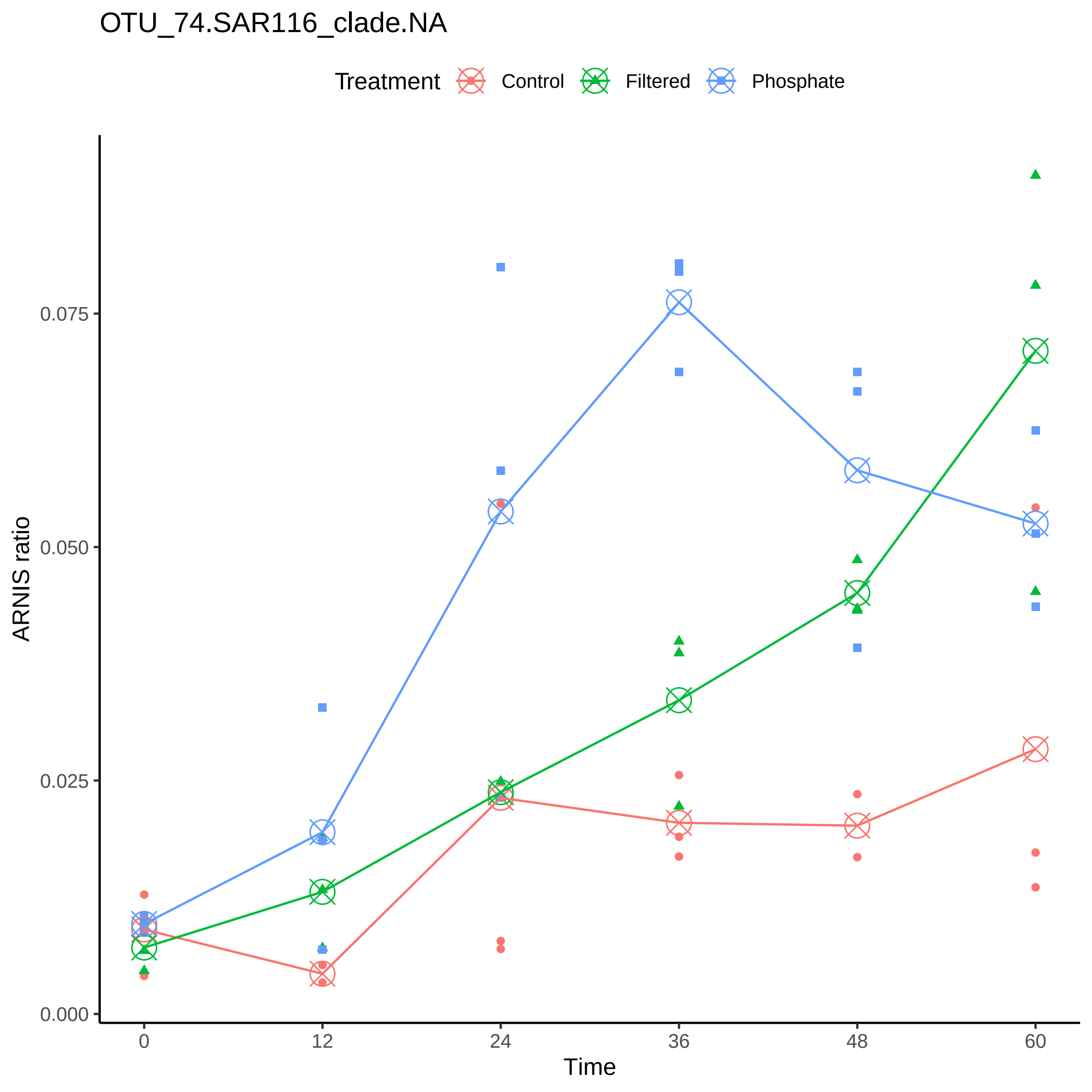
OTU_74.SAR116_clade.NA
Treatment
Control
Filtered
Phosphate
ARNIS ratio
0.000
0.025
0.050
0.075
Time
0
12
24
36
48
60

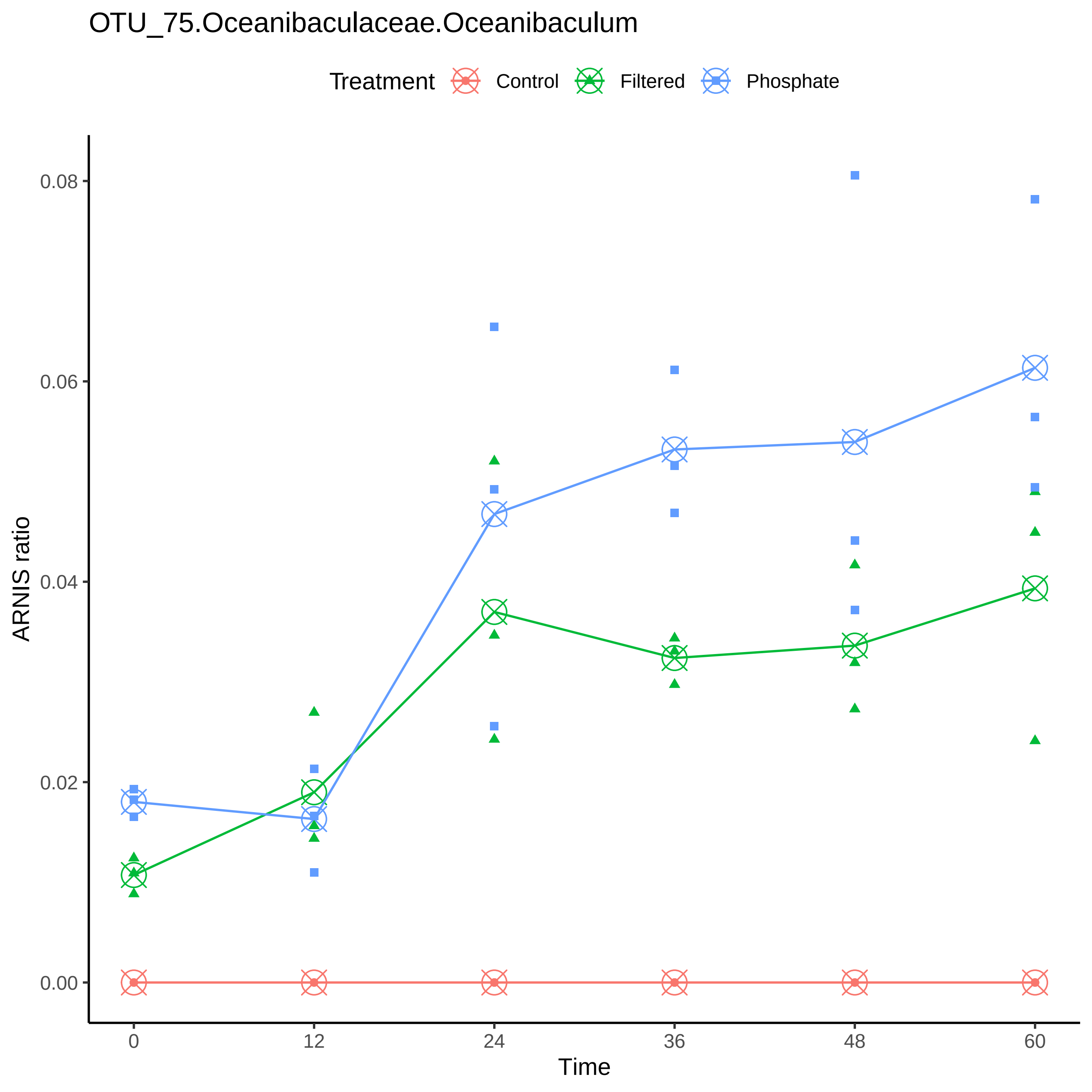
OTU_75.Oceanibaculaceae.Oceanibaculum
Treatment
Control
Filtered
Phosphate
ARNIS ratio
0.08
0.06
0.04
0.02
0.00
0
12
24
36
48
60
Time

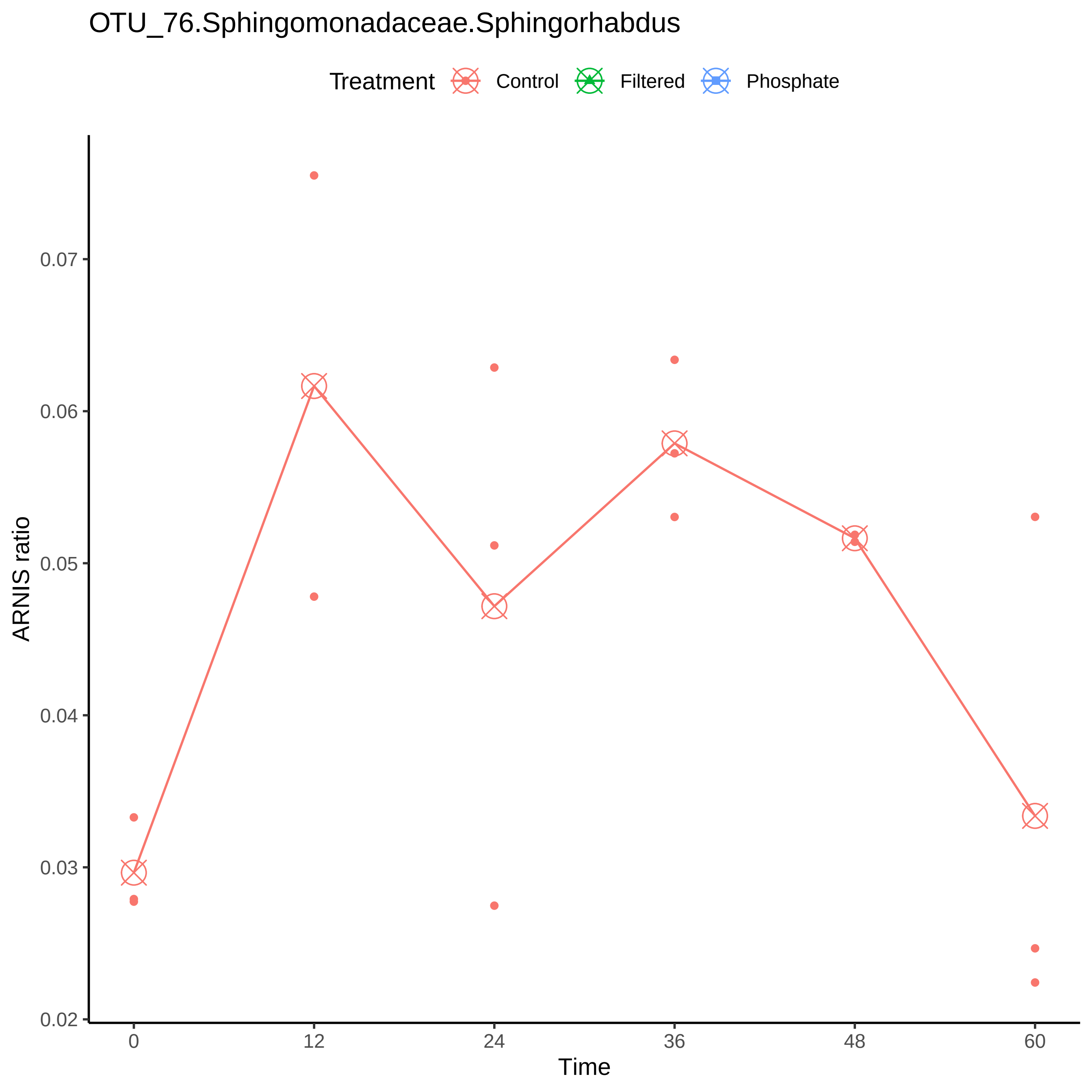
OTU_76.Sphingomonadaceae.Sphingorhabdus
Treatment
Control
Filtered
Phosphate
0.07
0.06
0.05
0.04
0.03
0.02
ARNIS ratio
0
12
24
36
48
60
Time

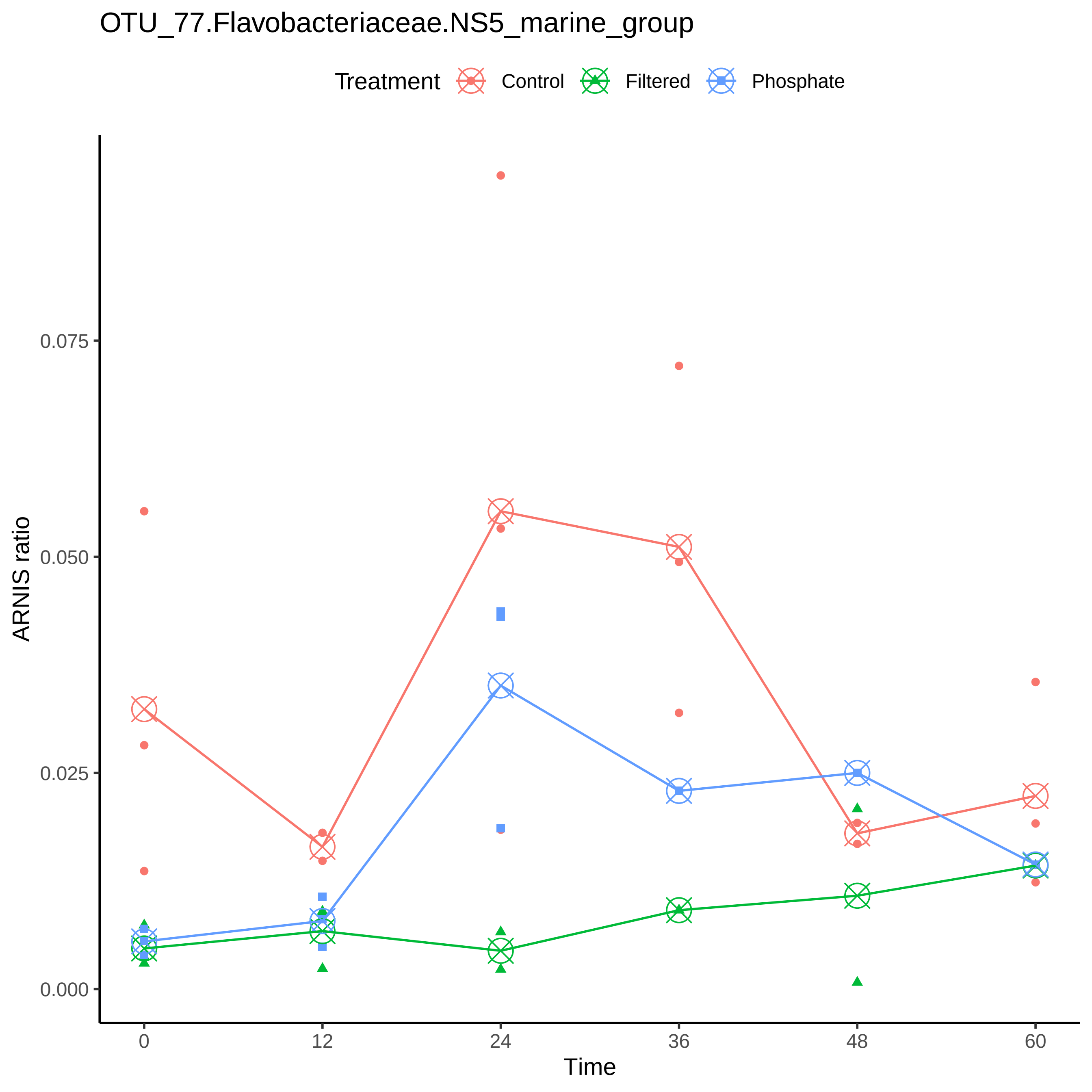
OTU_77.Flavobacteriaceae.NS5_marine_group
Treatment
Control
Filtered
Phosphate
ARNIS ratio
0.000
0.025
0.050
0.075
Time
0
12
24
36
48
60

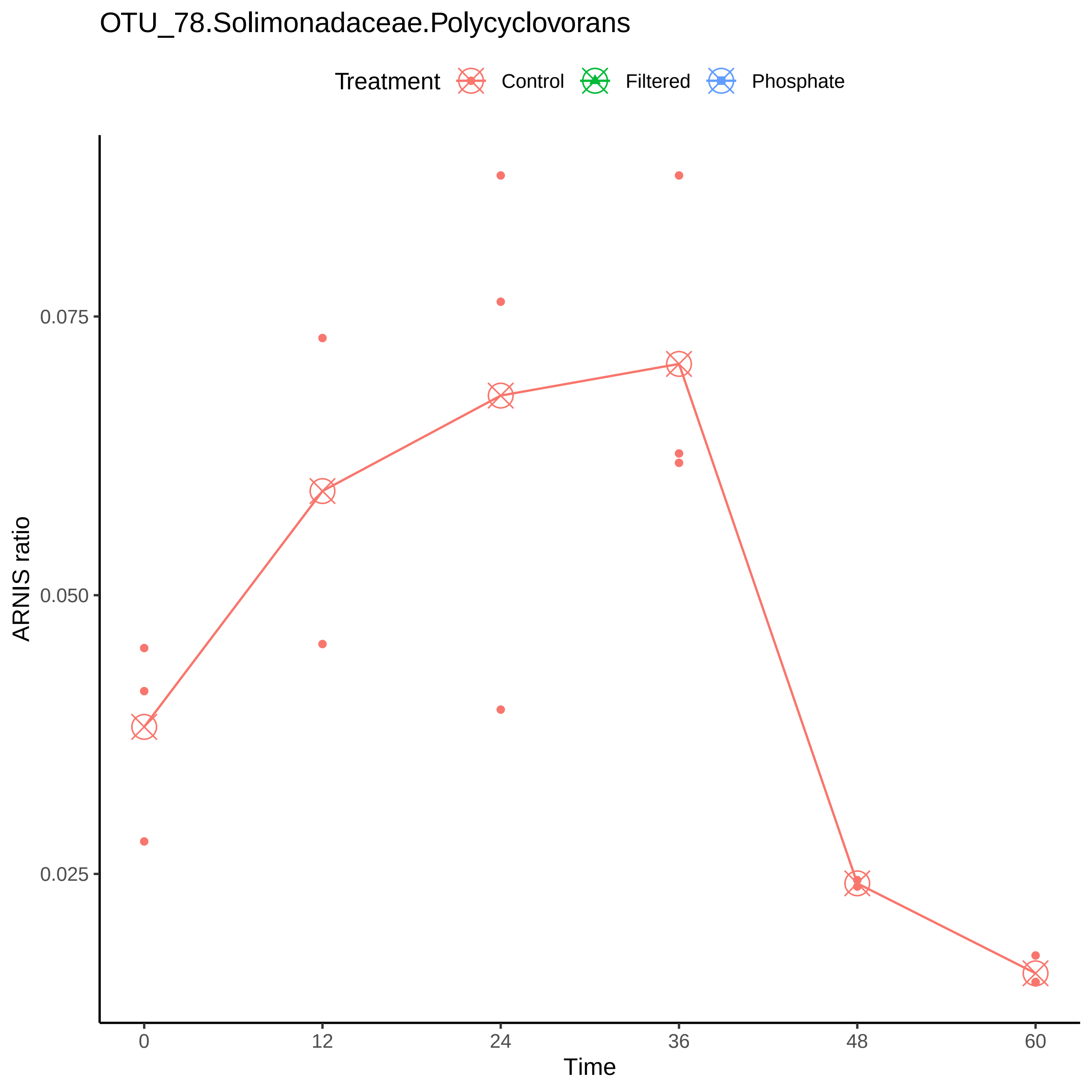

OTU_78.Solimonadaceae.Polycyclovorans
Treatment
Control
Filtered
Phosphate
ARNIS ratio
0.075
0.050
0.025
0
12
24
36
48
60
Time

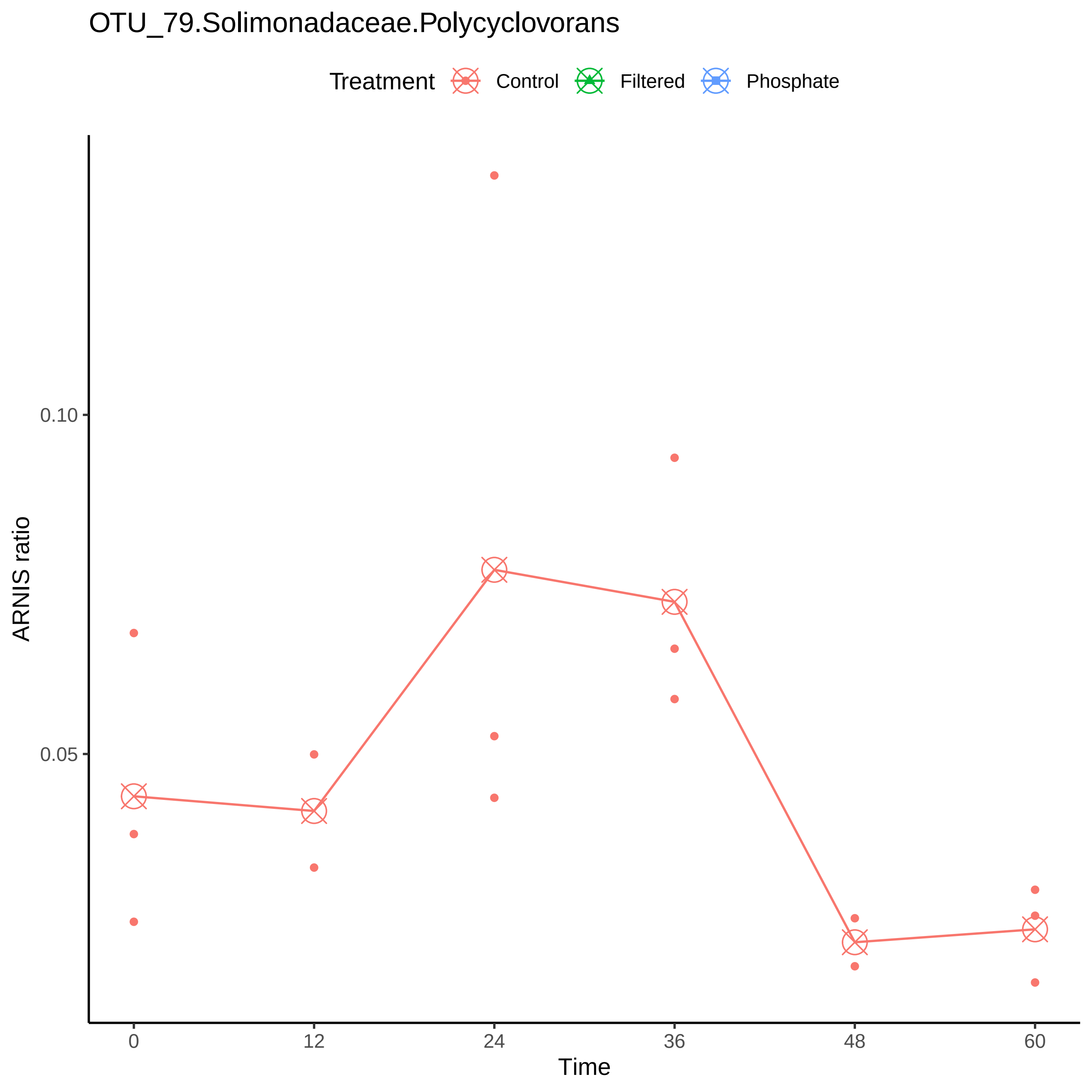

OTU_79.Solimonadaceae.Polycyclovorans
Treatment
Control
Filtered
Phosphate
ARNIS ratio
0.10
0.05
0
12
24
36
48
60
Time

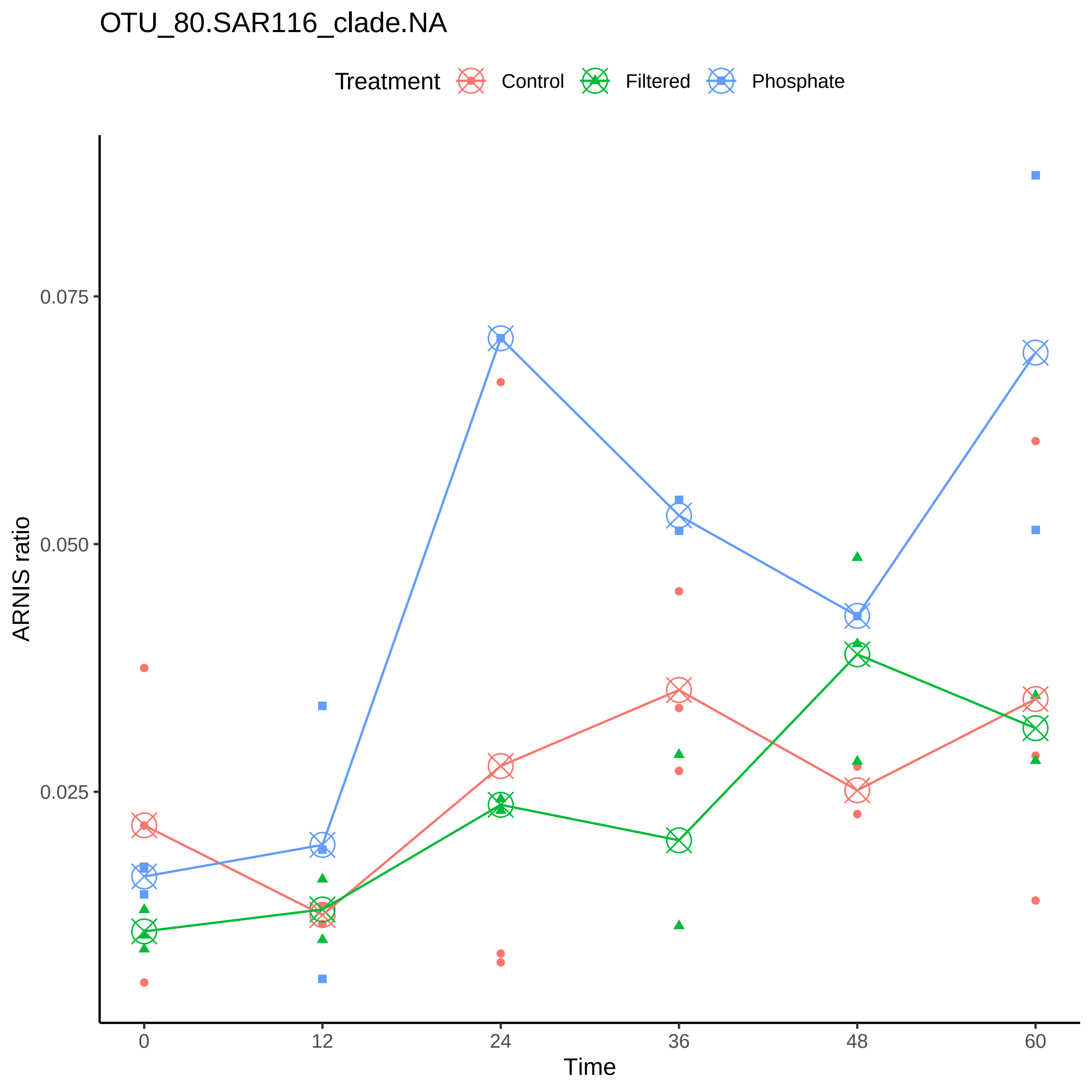

OTU_80.SAR116_clade.NA
Treatment
Control
Filtered
Phosphate
ARNIS ratio
0.075
0.050
0.025
0
12
24
36
48
60
Time

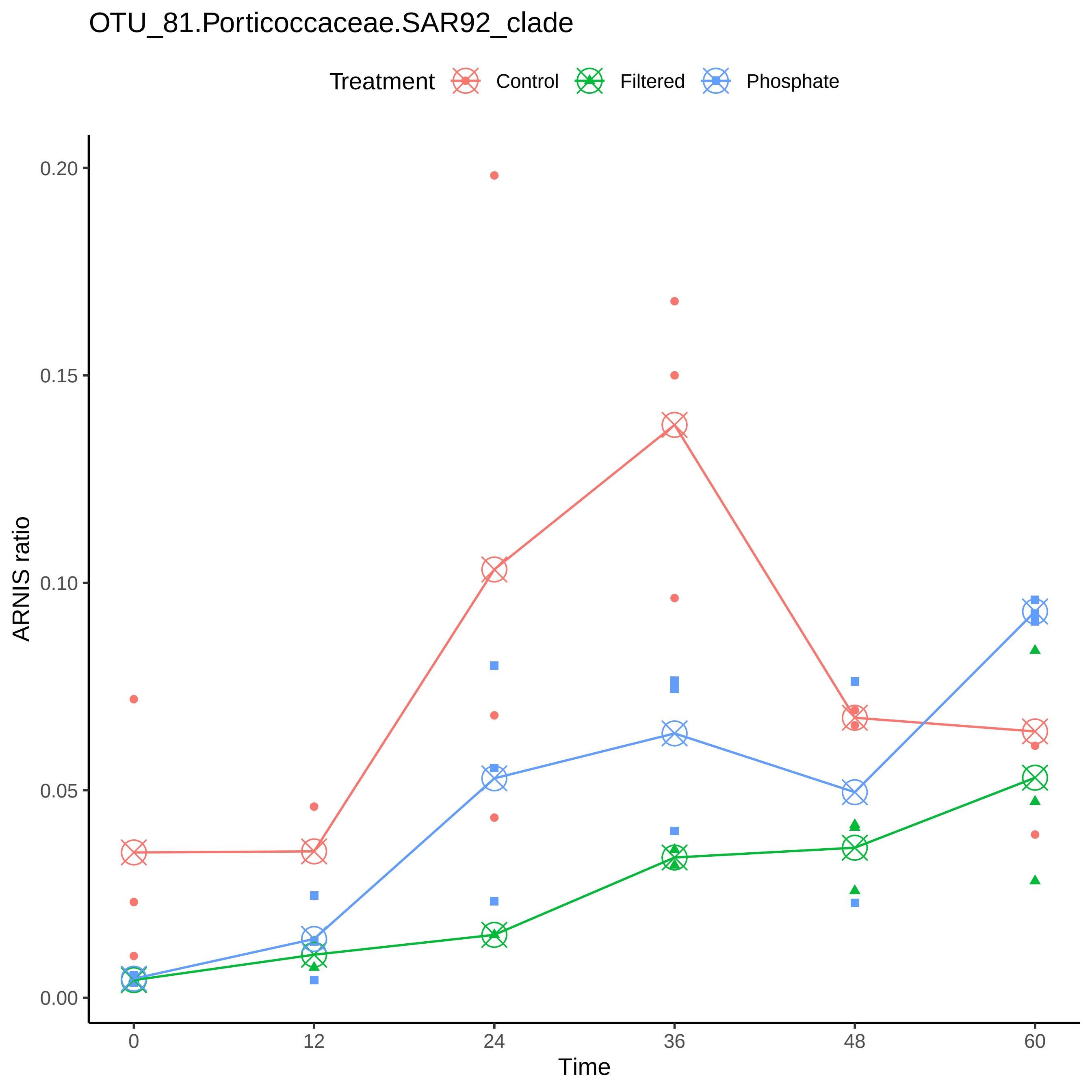

OTU_81.Porticoccaceae.SAR92_clade
Treatment
Control
Filtered
Phosphate
ARNIS ratio
0.00
0.05
0.10
0.15
0.20
Time
0
12
24
36
48
60

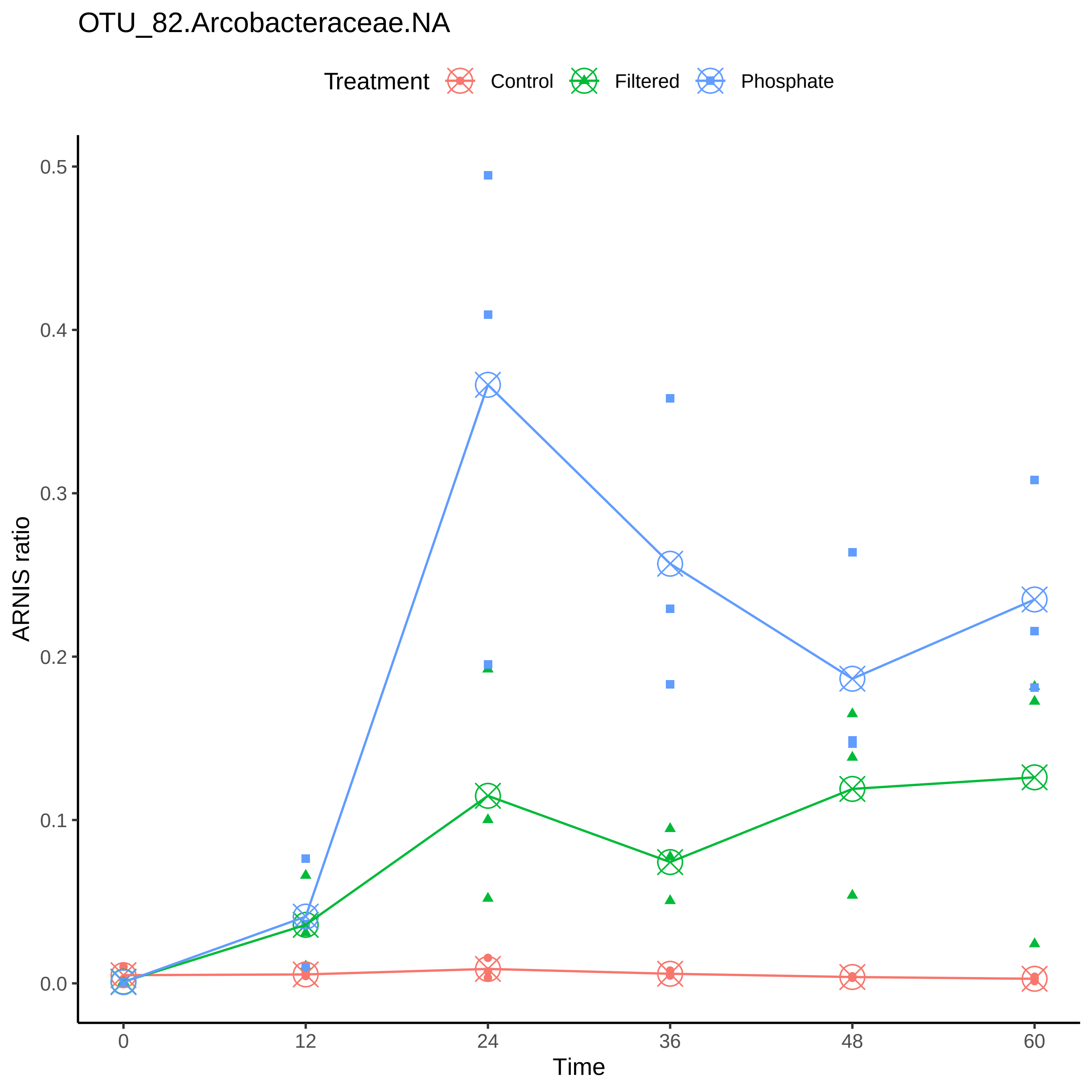

OTU_82.Arcobacteraceae.NA
Treatment
Control
Filtered
Phosphate
ARNIS ratio
0.0
0.1
0.2
0.3
0.4
0.5
0
12
24
36
48
60
Time

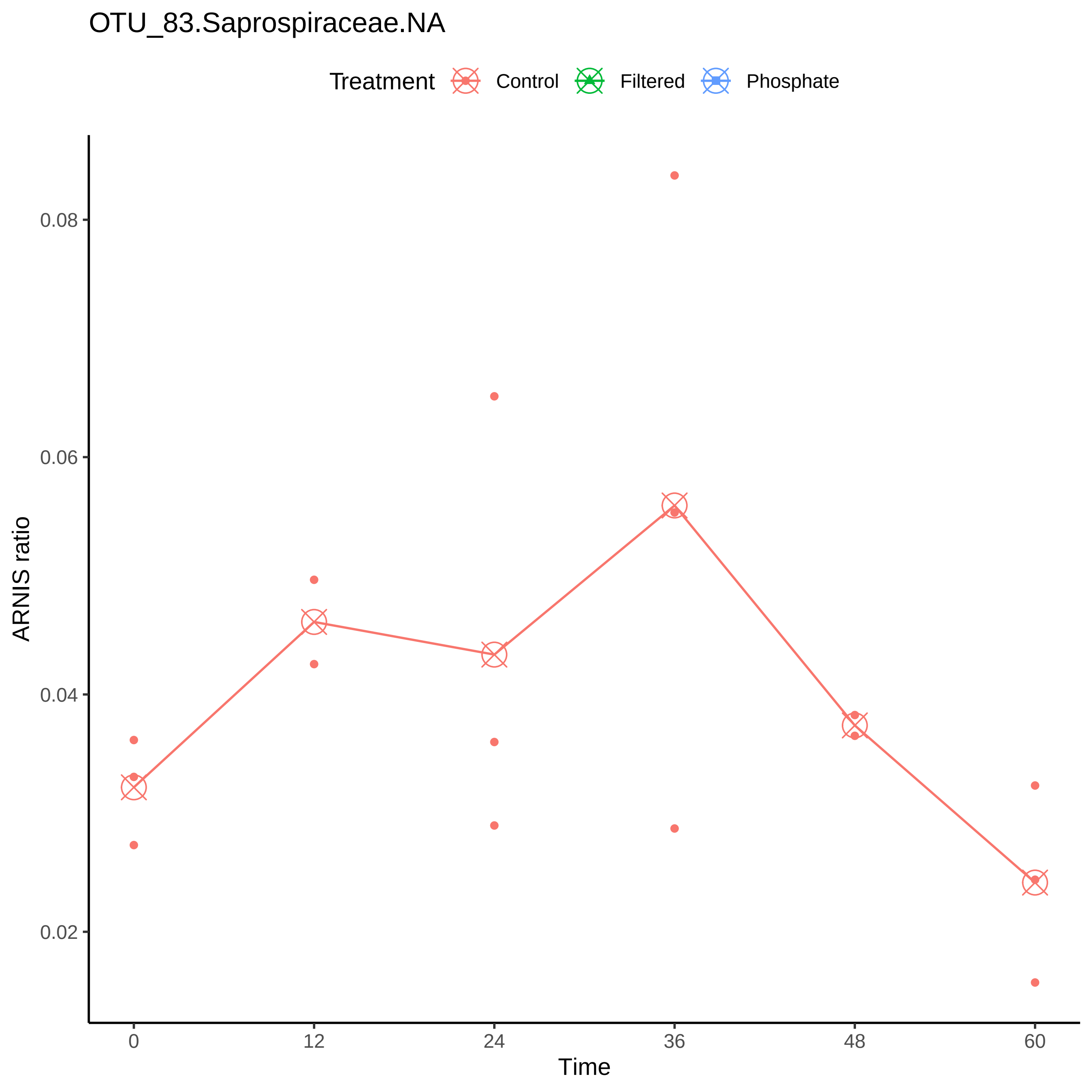
OTU_83.Saprospiraceae.NA
Treatment
Control
Filtered
Phosphate
0.08
0.06
0.04
0.02
ARNIS ratio
0
12
24
36
48
60
Time

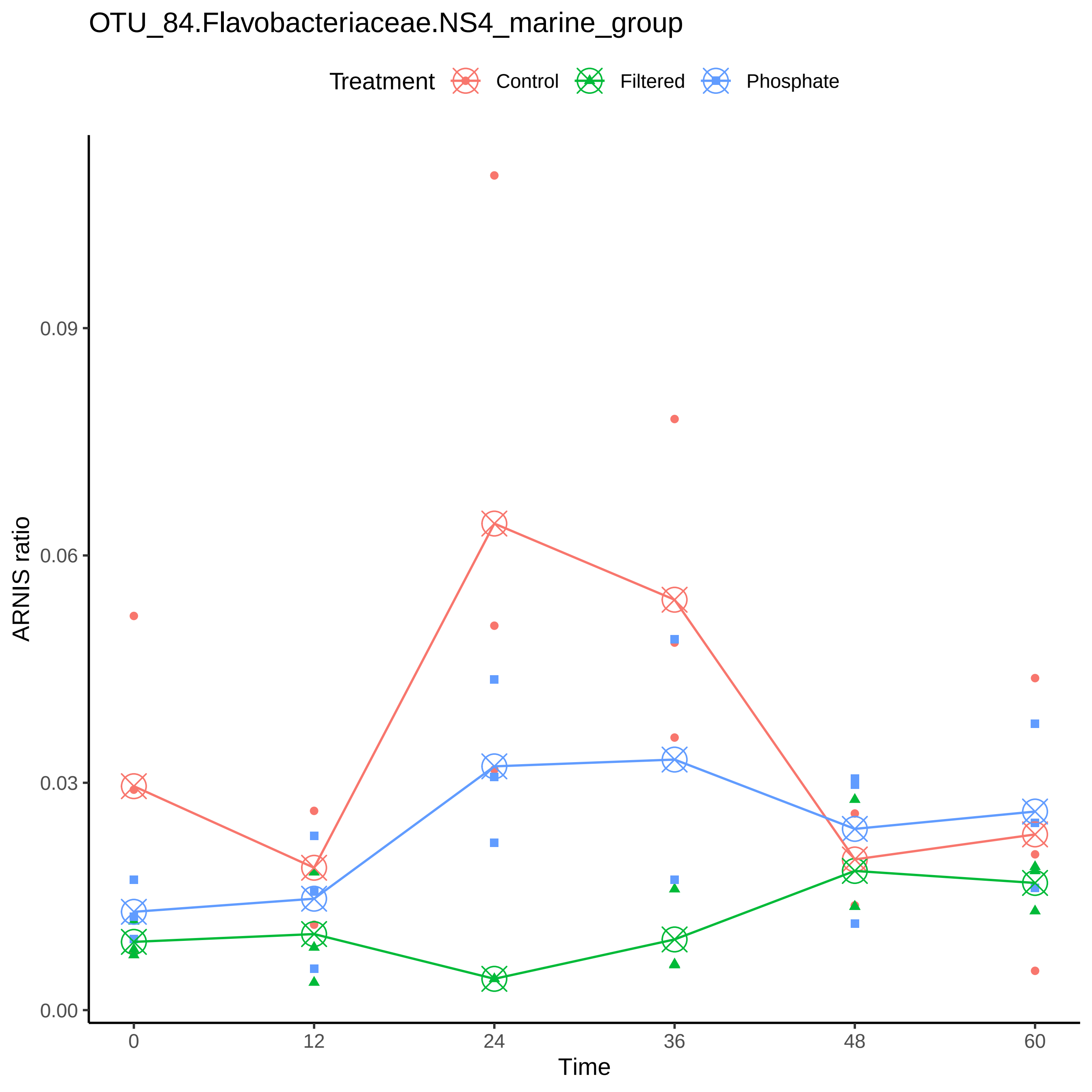
OTU_84.Flavobacteriaceae.NS4_marine_group
Treatment
Control
Filtered
Phosphate
ARNIS ratio
0.00
0.03
0.06
0.09
Time
0
12
24
36
48
60

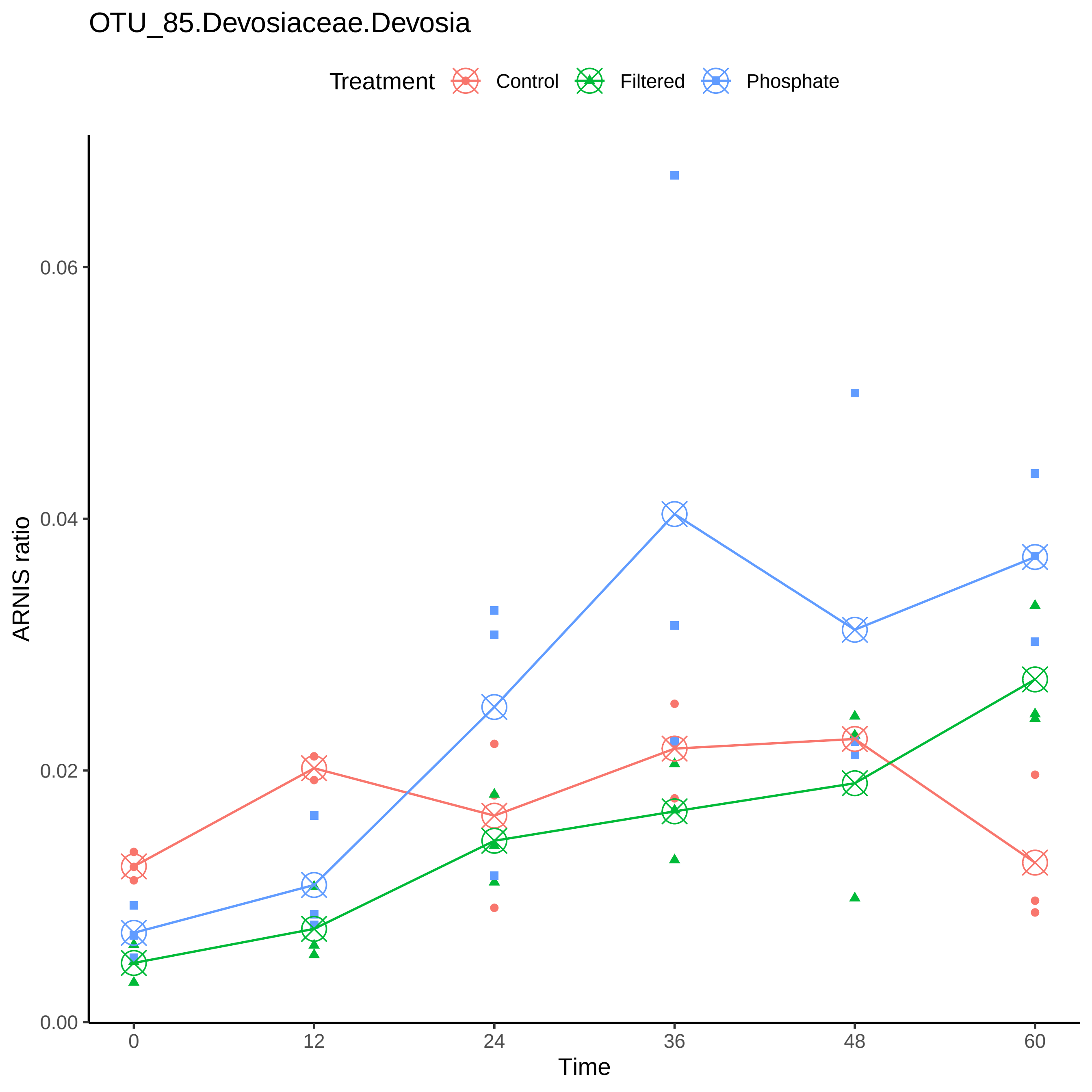

OTU_85.Devosiaceae.Devosia
Treatment
Control
Filtered
Phosphate
ARNIS ratio
0.00
0.02
0.04
0.06
Time
0
12
24
36
48
60

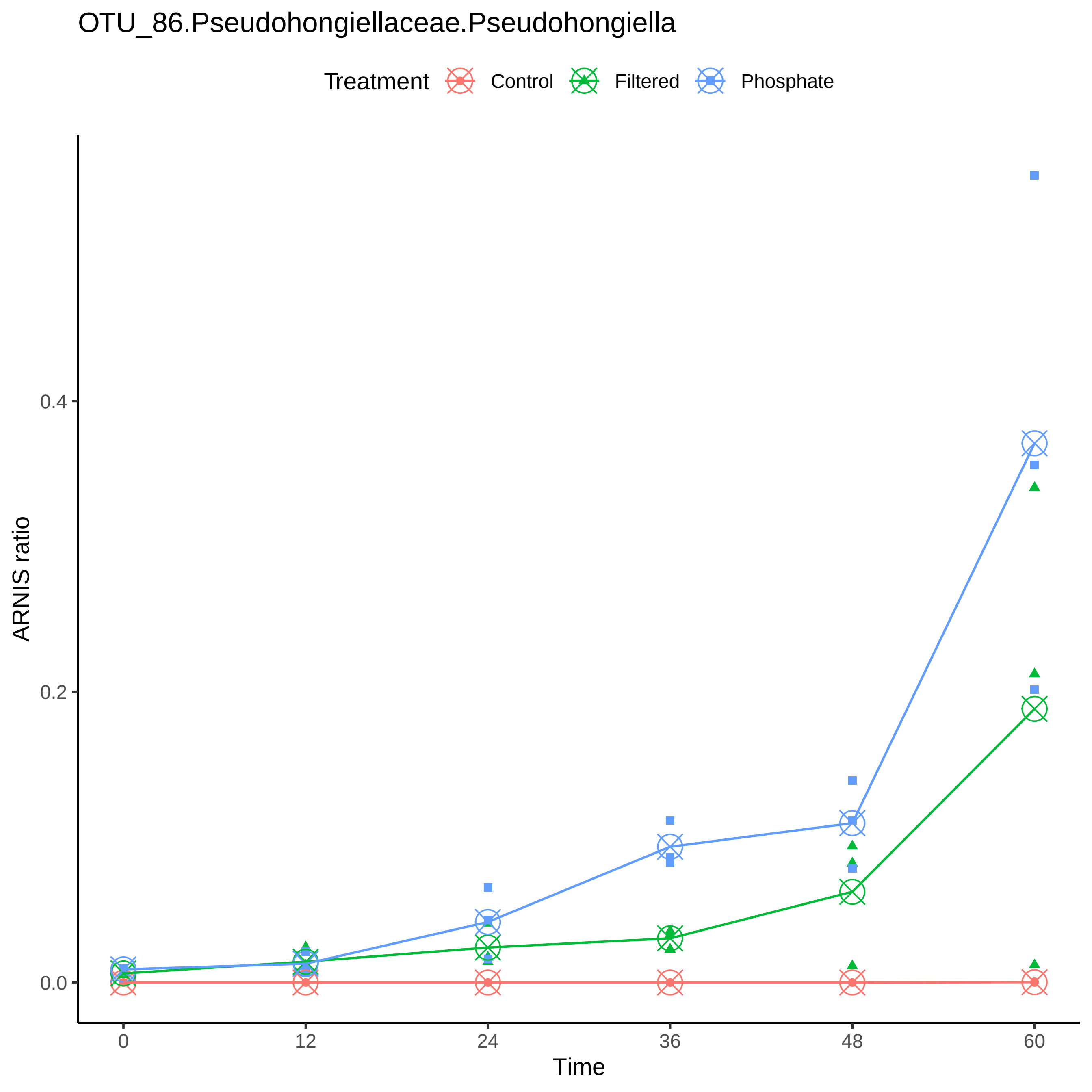
OTU_86.Pseudohongiellaceae.Pseudohongiella
Treatment
Control
Filtered
Phosphate
ARNIS ratio
0.0
0.2
0.4
Time
0
12
24
36
48
60

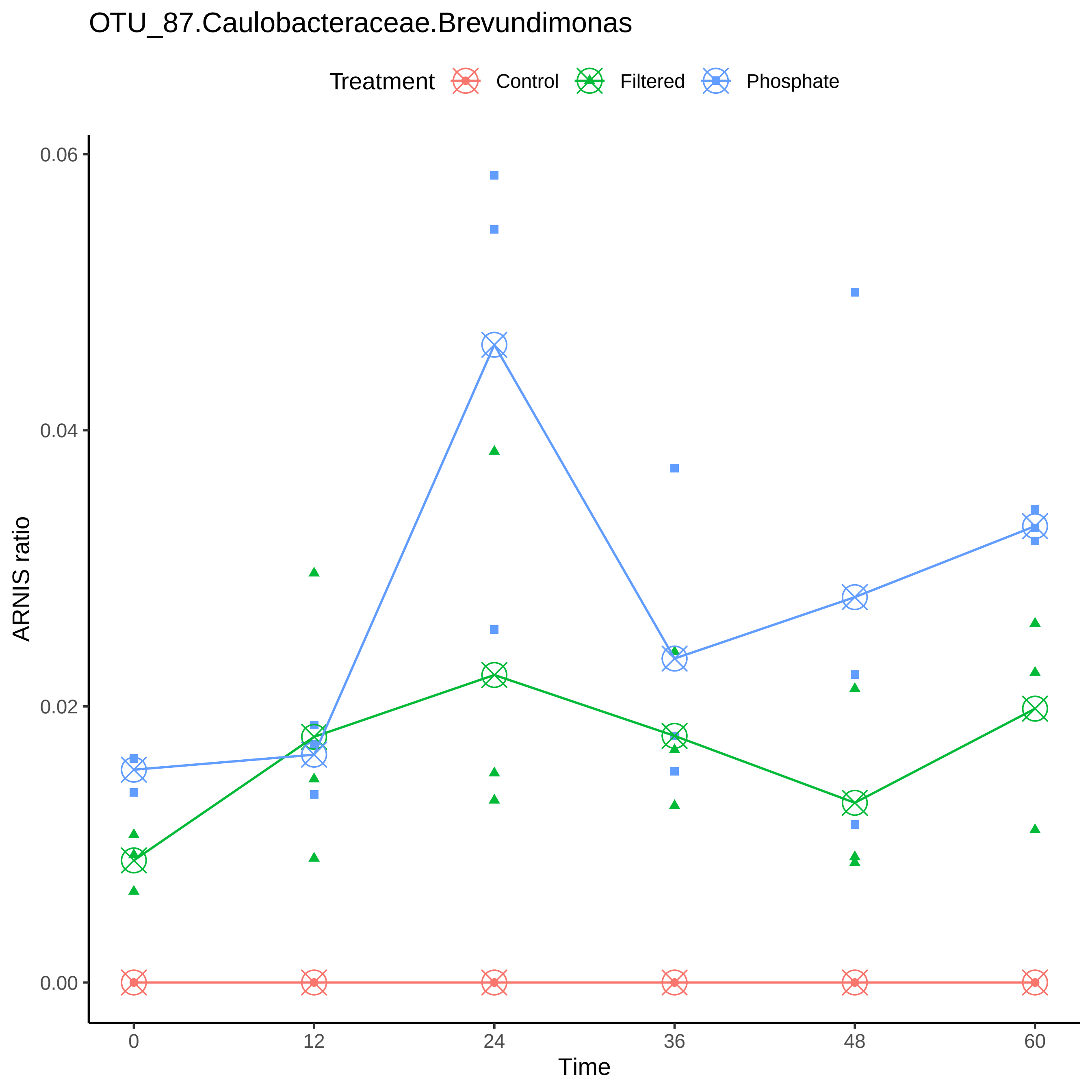

OTU_87.Caulobacteraceae.Brevundimonas
Treatment
Control
Filtered
Phosphate
ARNIS ratio
0.06
0.04
0.02
0.00
Time
0
12
24
36
48
60

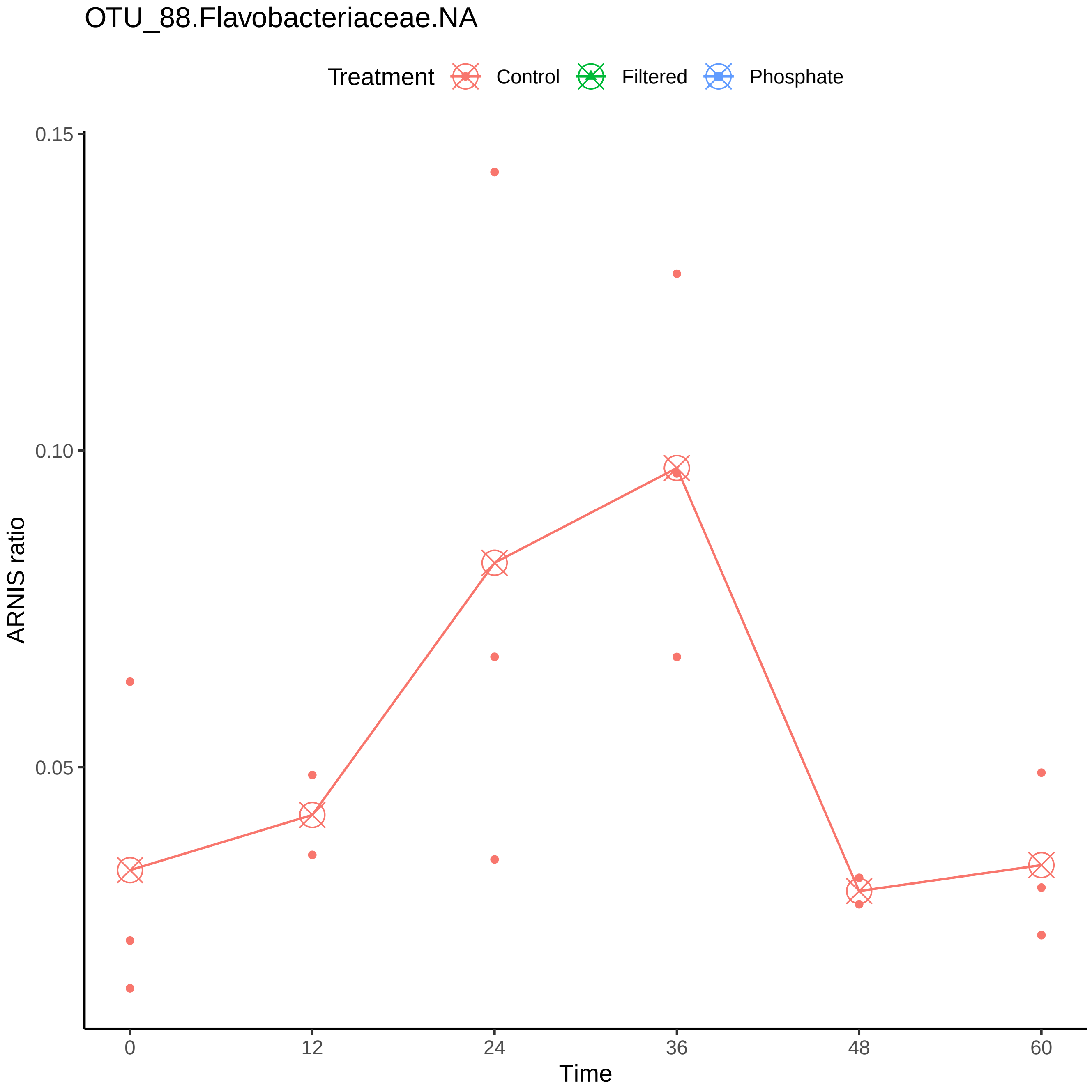
OTU_88.Flavobacteriaceae.NA
Treatment
Control
Filtered
Phosphate
0.15
0.10
0.05
ARNIS ratio
0
12
24
36
48
60
Time

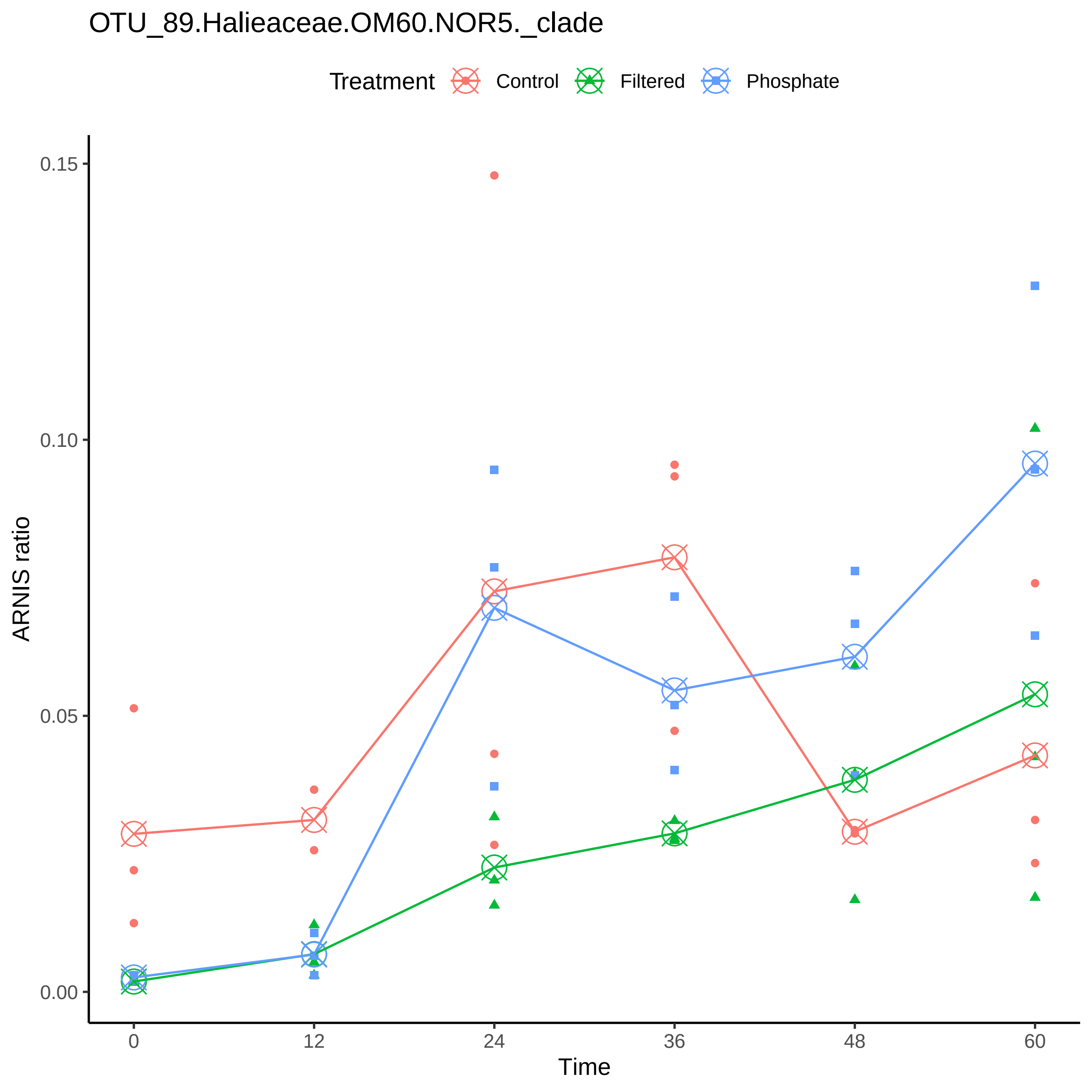

OTU_89.Halieaceae.OM60.NOR5._clade
Treatment
Control
Filtered
Phosphate
ARNIS ratio
0.00
0.05
0.10
0.15
Time
0
12
24
36
48
60

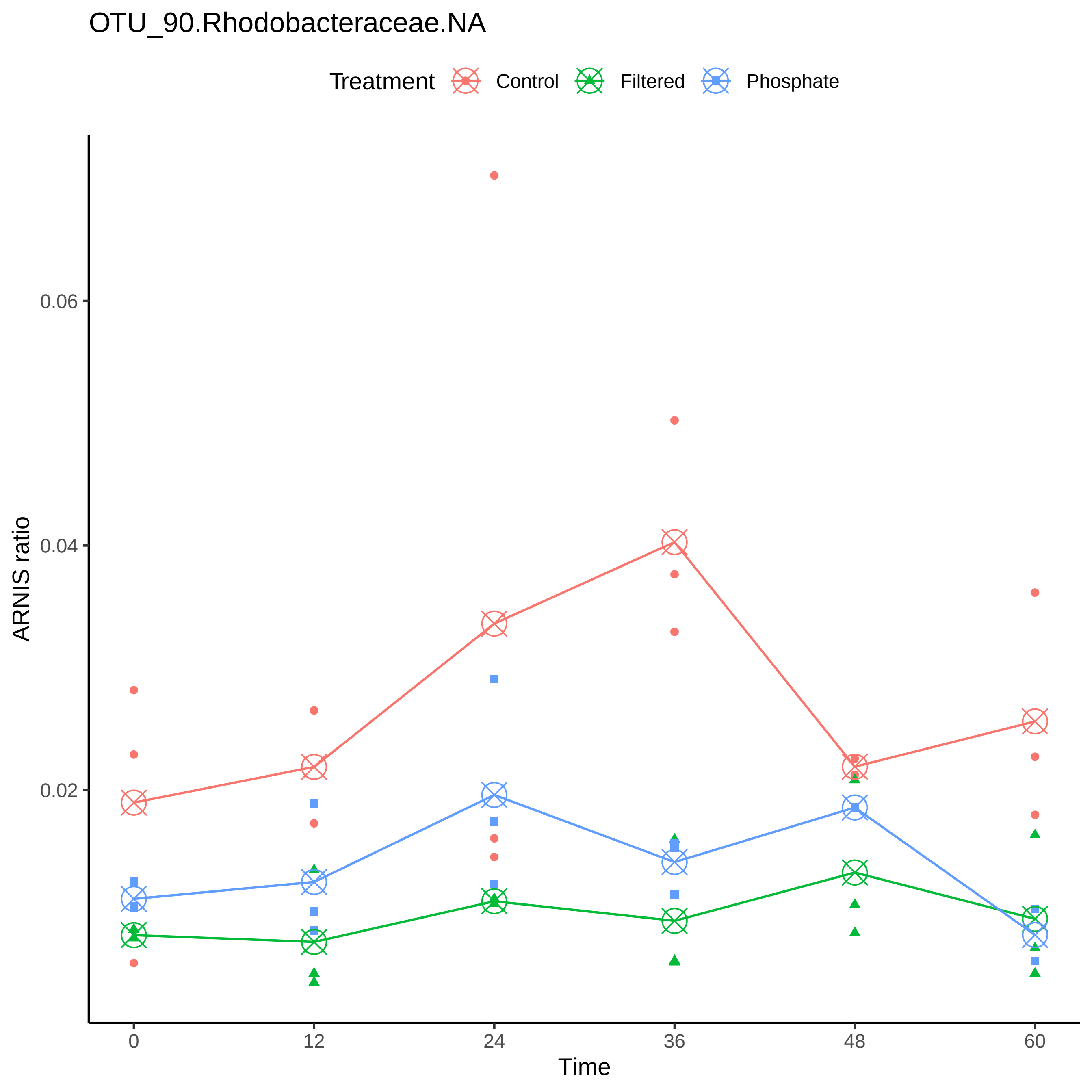

OTU_90.Rhodobacteraceae.NA
Treatment
Control
Filtered
Phosphate
ARNIS ratio
0.06
0.04
0.02
0
12
24
36
48
60
Time

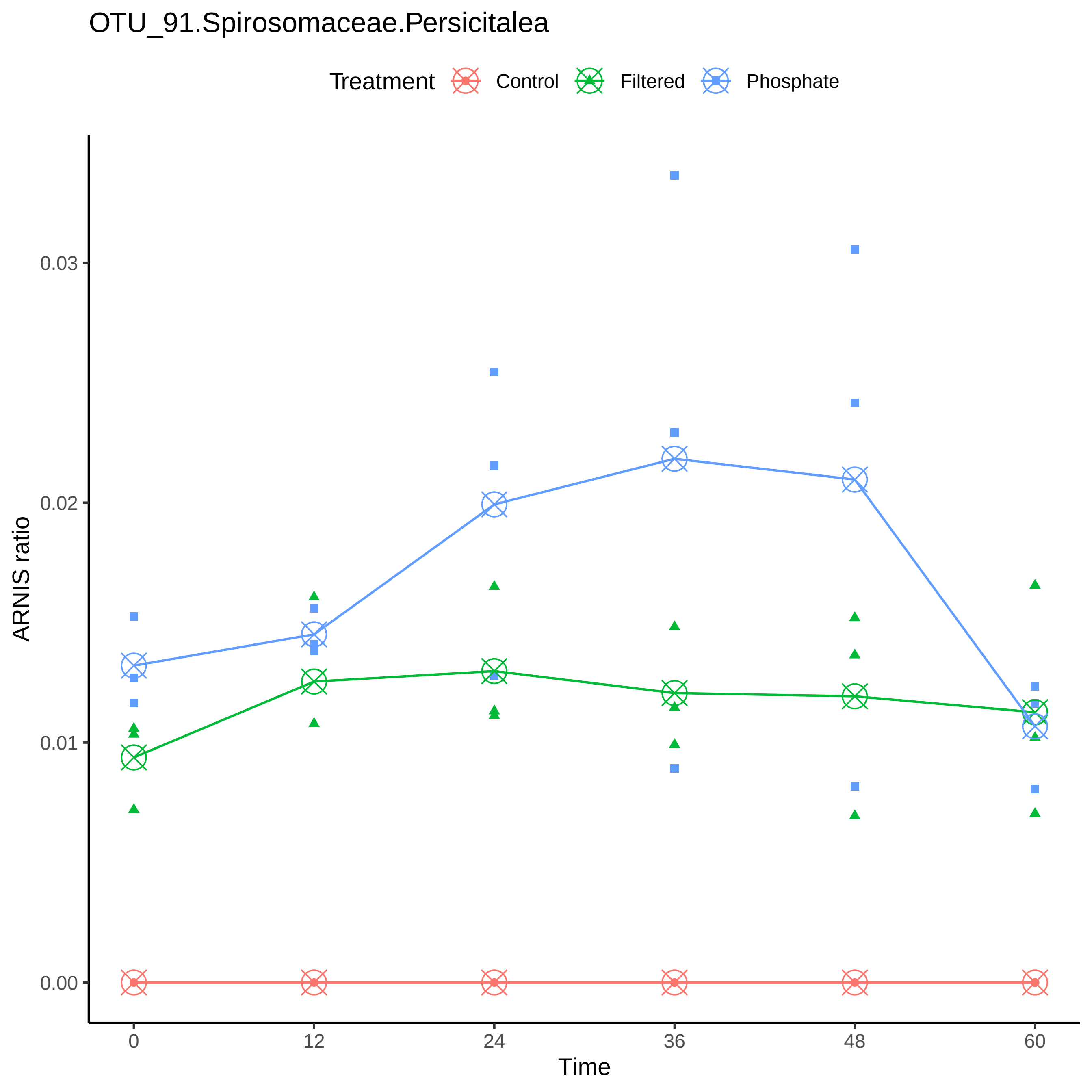

OTU_91.Spirosomaceae.Persicitalea
Treatment
Control
Filtered
Phosphate
ARNIS ratio
0.03
0.02
0.01
0.00
0
12
24
36
48
60
Time

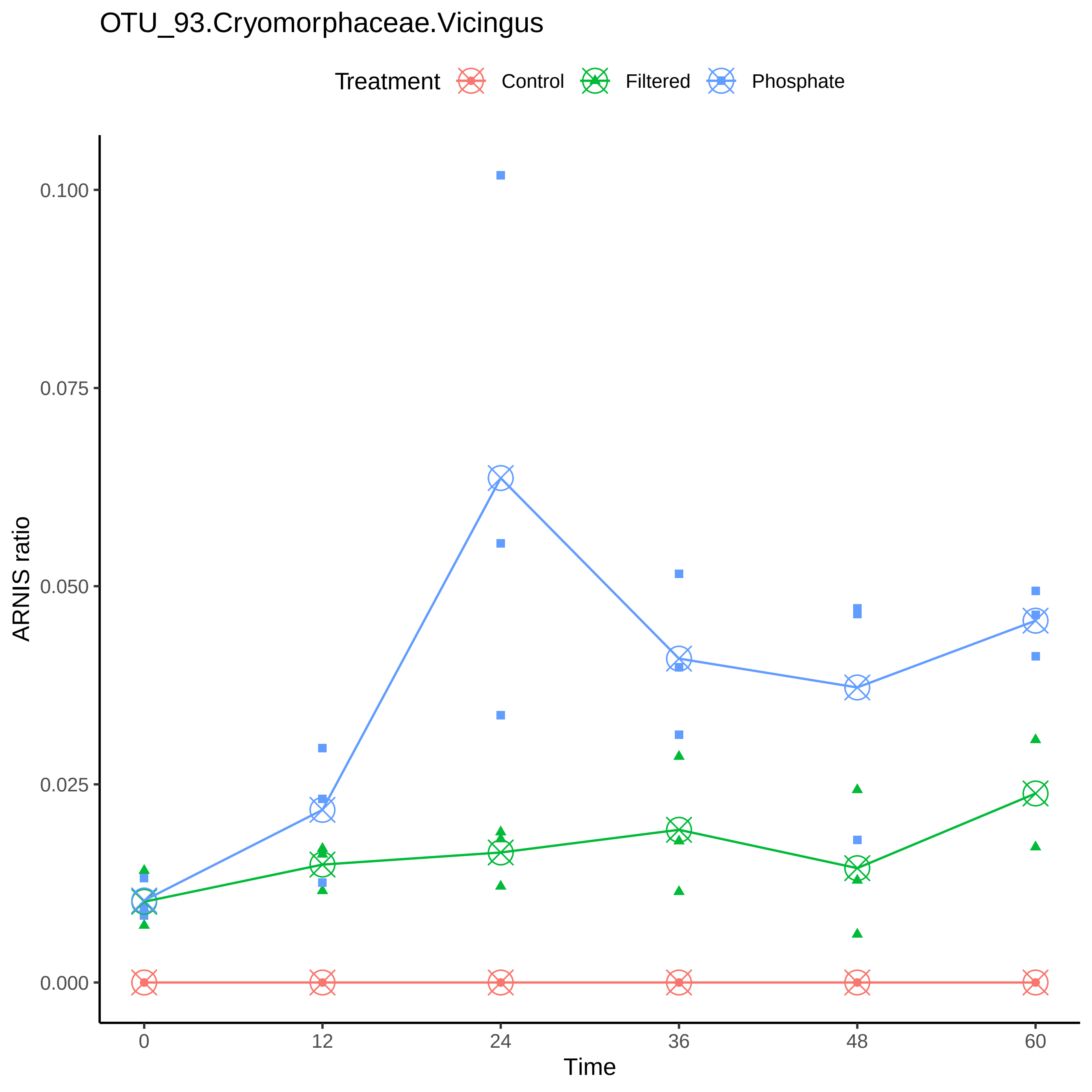

OTU_93.Cryomorphaceae.Vicingus
Treatment
Control
Filtered
Phosphate
ARNIS ratio
0.100
0.075
0.050
0.025
0.000
Time
0
12
24
36
48
60

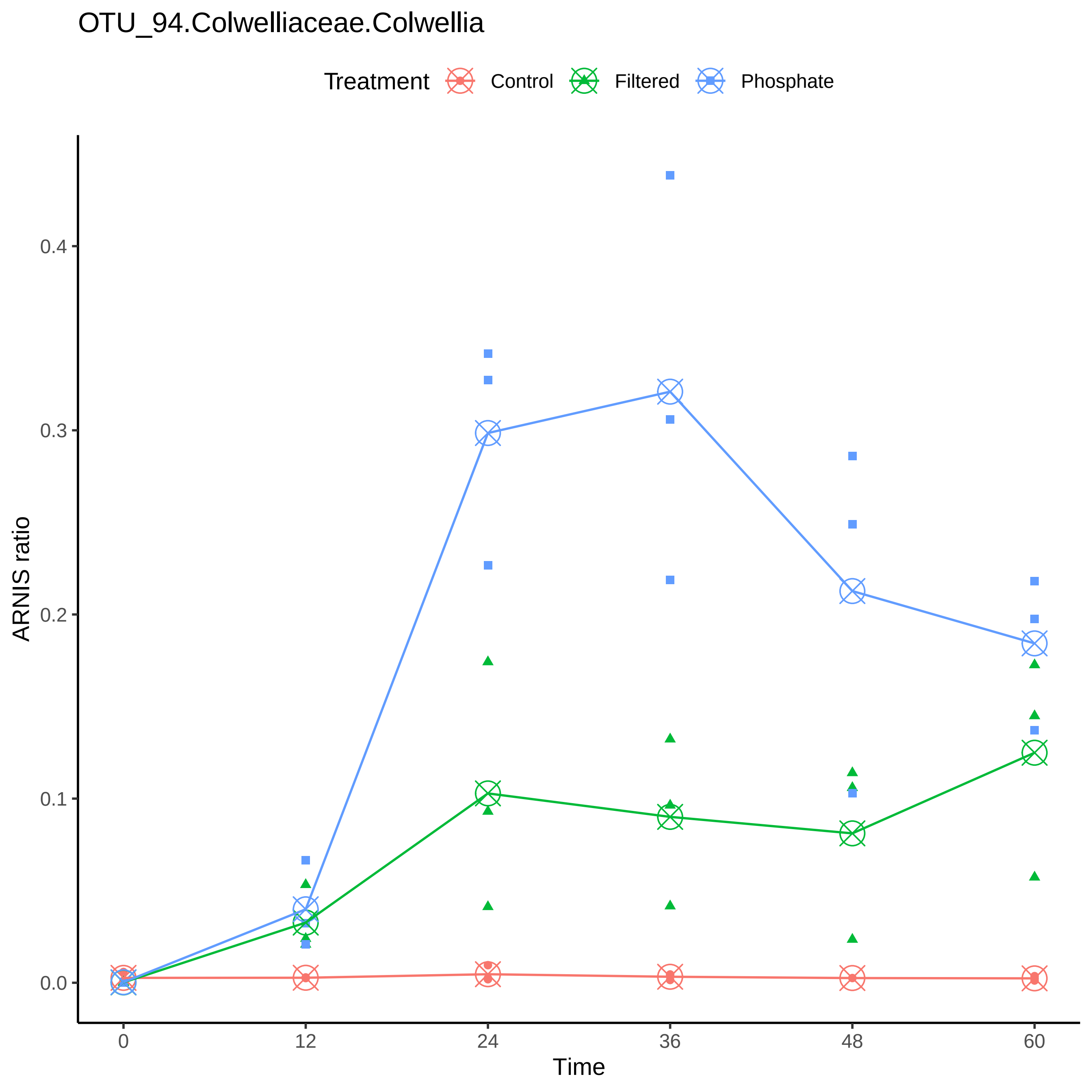
OTU_94.Colwelliaceae.Colwellia
Treatment
Control
Filtered
Phosphate
ARNIS ratio
0.0
0.1
0.2
0.3
0.4
Time
0
12
24
36
48
60

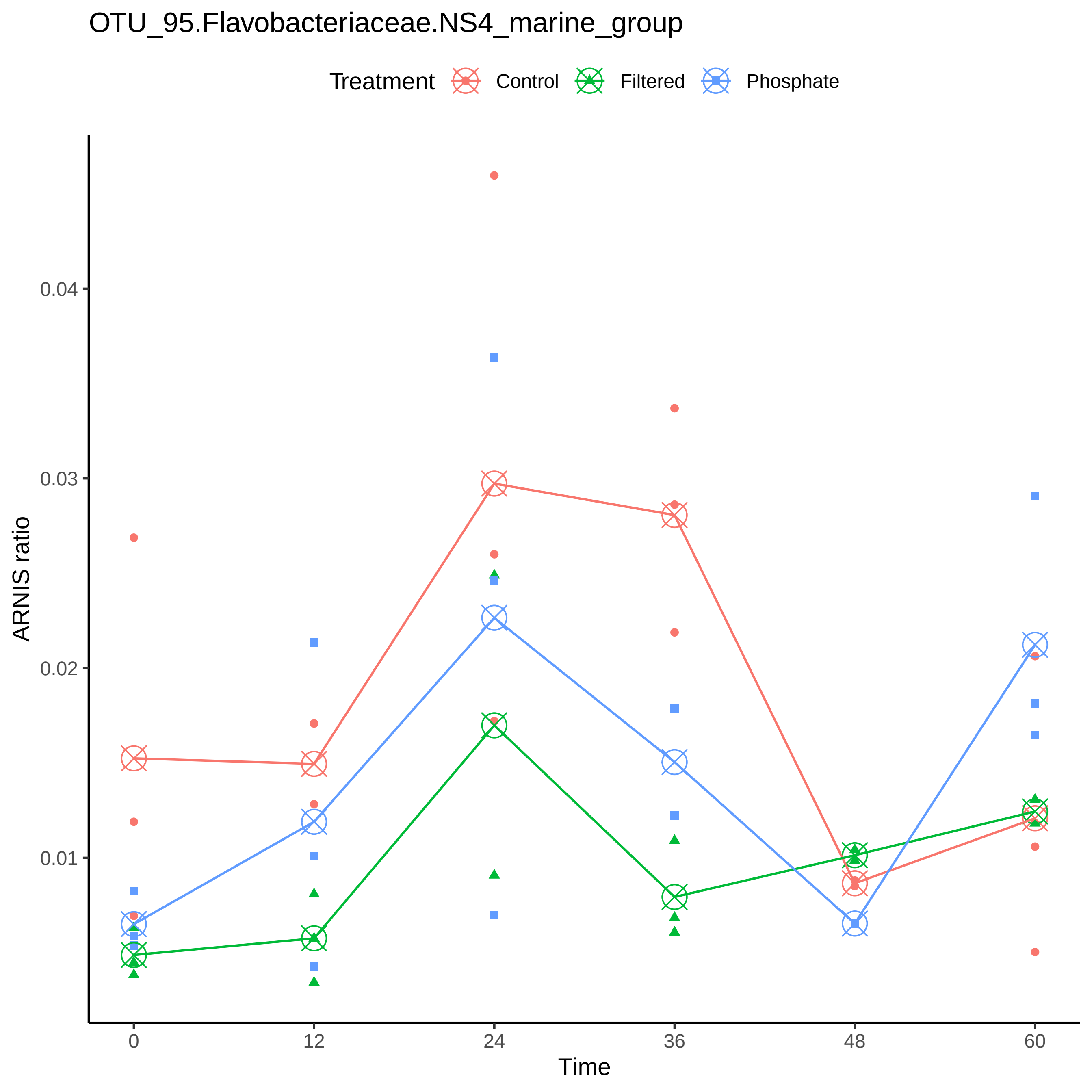
OTU_95.Flavobacteriaceae.NS4_marine_group
Treatment
Control
Filtered
Phosphate
ARNIS ratio
0.04
0.03
0.02
0.01
0
12
24
36
48
60
Time

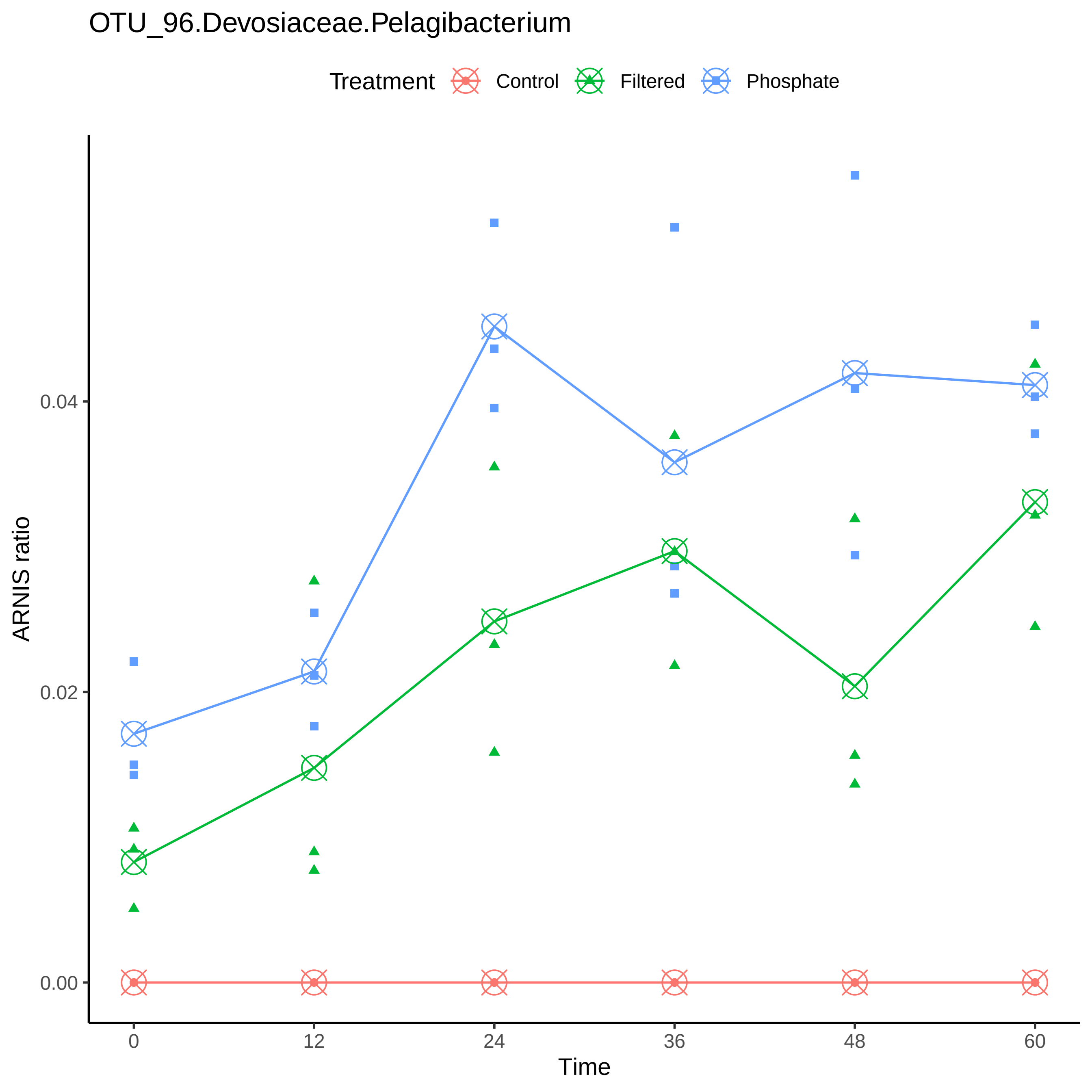

OTU_96.Devosiaceae.Pelagibacterium
Treatment
Control
Filtered
Phosphate
ARNIS ratio
0.00
0.02
0.04
Time
0
12
24
36
48
60

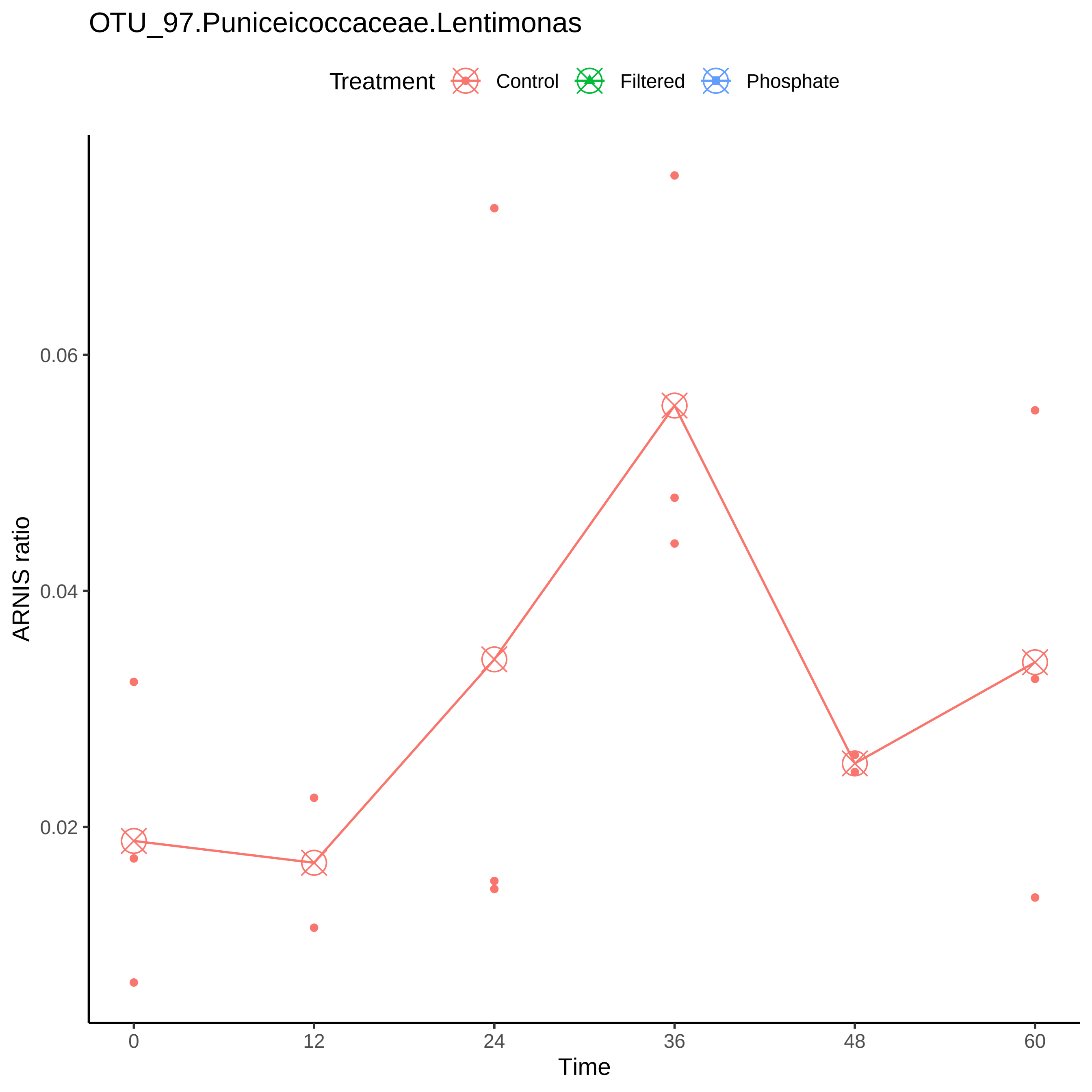
OTU_97.Puniceicoccaceae.Lentimonas
Treatment
Control
Filtered
Phosphate
ARNIS ratio
0.06
0.04
0.02
0
12
24
36
48
60
Time

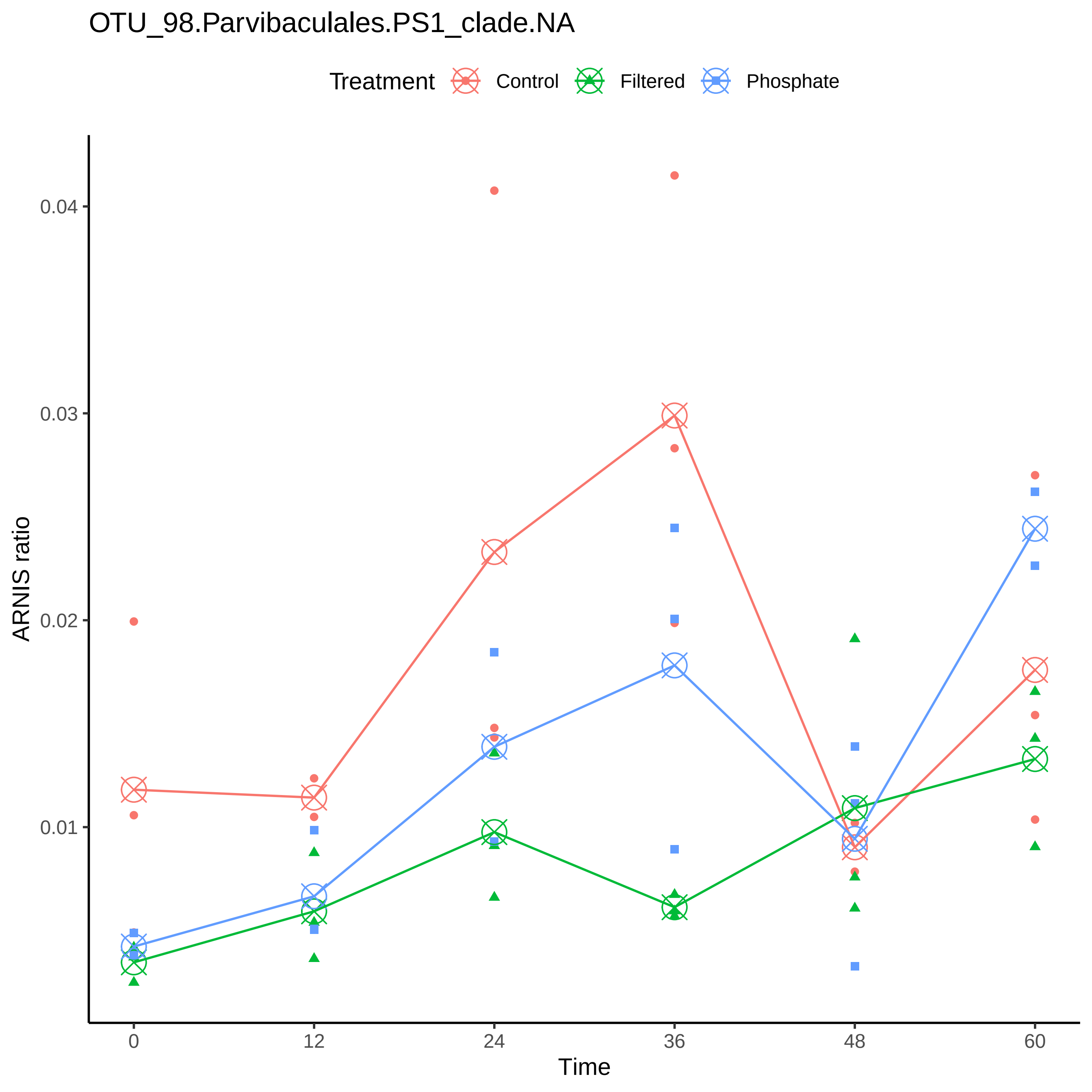

OTU_98.Parvibaculales.PS1_clade.NA
Treatment
Control
Filtered
Phosphate
ARNIS ratio
0.04
0.03
0.02
0.01
0
12
24
36
48
60
Time

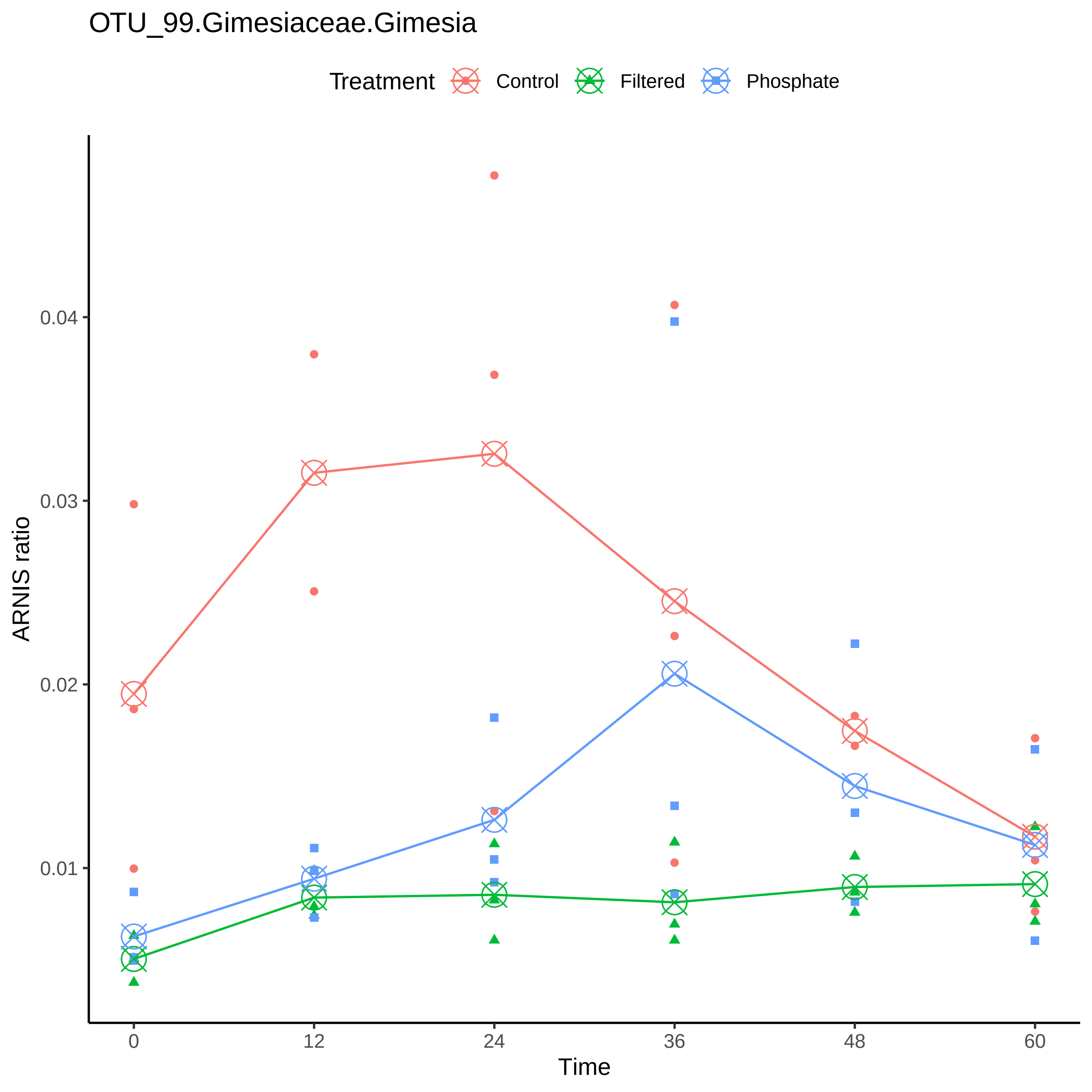
OTU_99.Gimesiaceae.Gimesia
Treatment
Control
Filtered
Phosphate
ARNIS ratio
0.04
0.03
0.02
0.01
0
12
24
36
48
60
Time

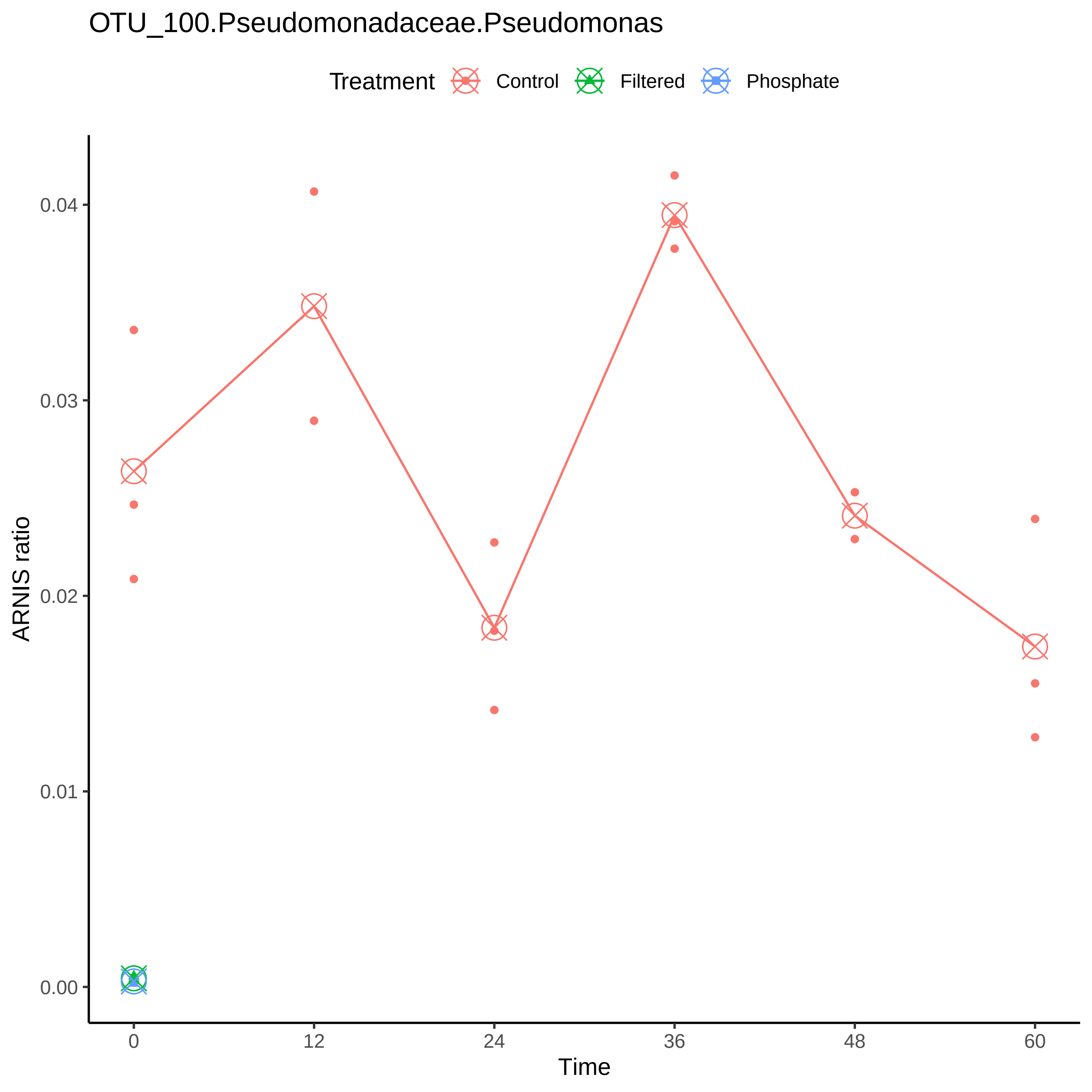

OTU_100.Pseudomonadaceae.Pseudomonas
Treatment
Control
Filtered
Phosphate
ARNIS ratio
0.00
0.01
0.02
0.03
0.04
Time
0
12
24
36
48
60

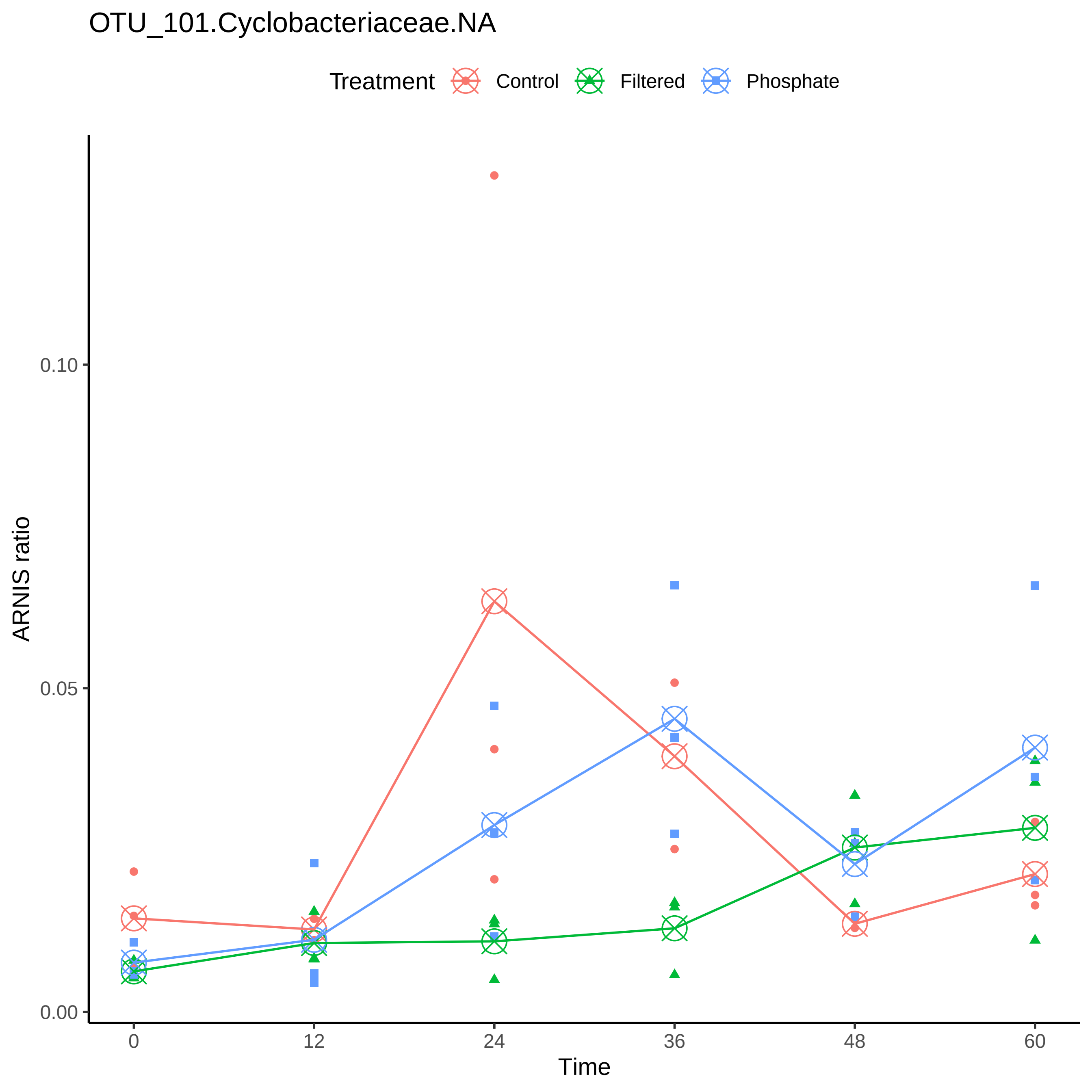
OTU_101.Cyclobacteriaceae.NA
Treatment
Control
Filtered
Phosphate
ARNIS ratio
0.10
0.05
0.00
0
12
24
36
48
60
Time

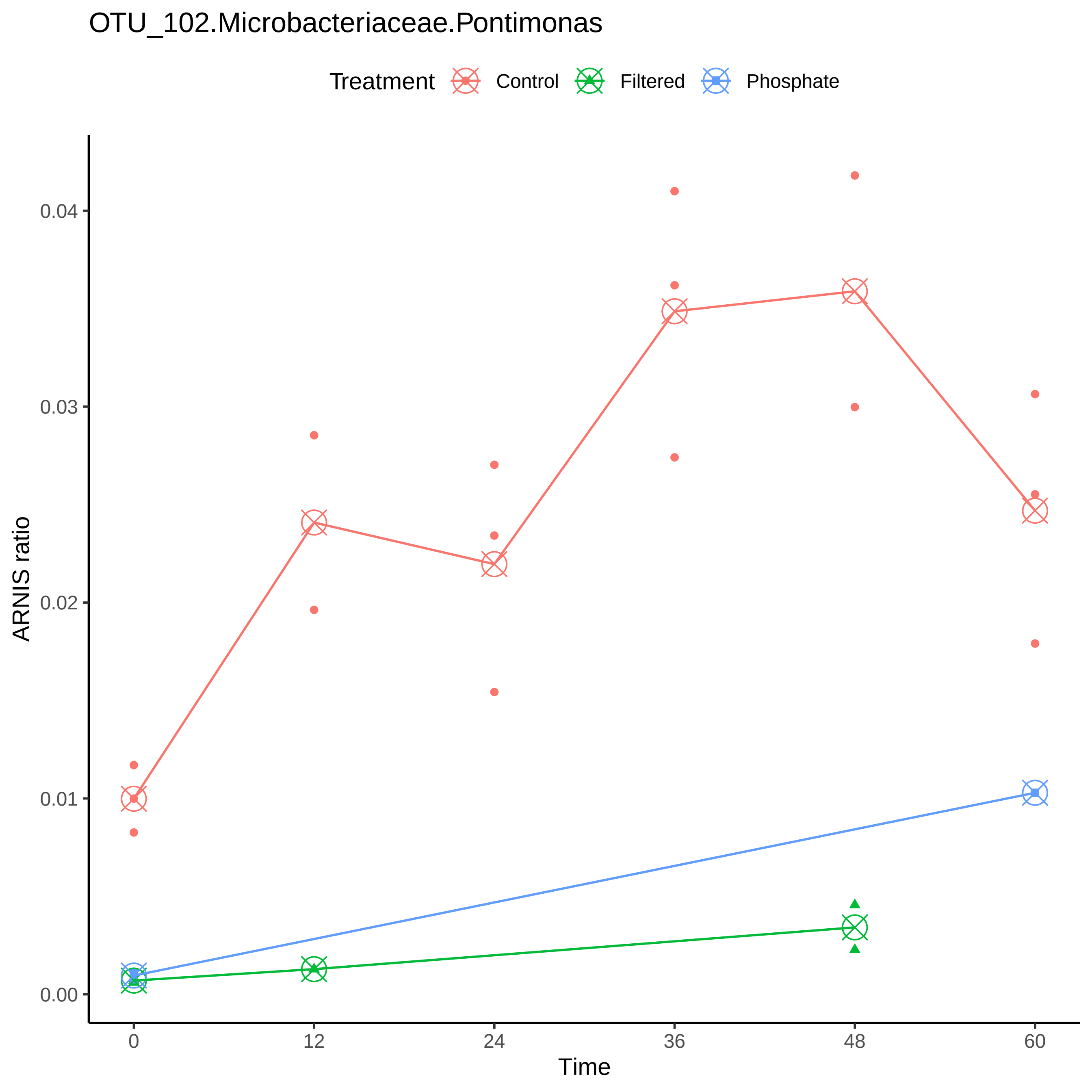

OTU_102.Microbacteriaceae.Pontimonas
Treatment
Control
Filtered
Phosphate
ARNIS ratio
0.00
0.01
0.02
0.03
0.04
Time
0
12
24
36
48
60

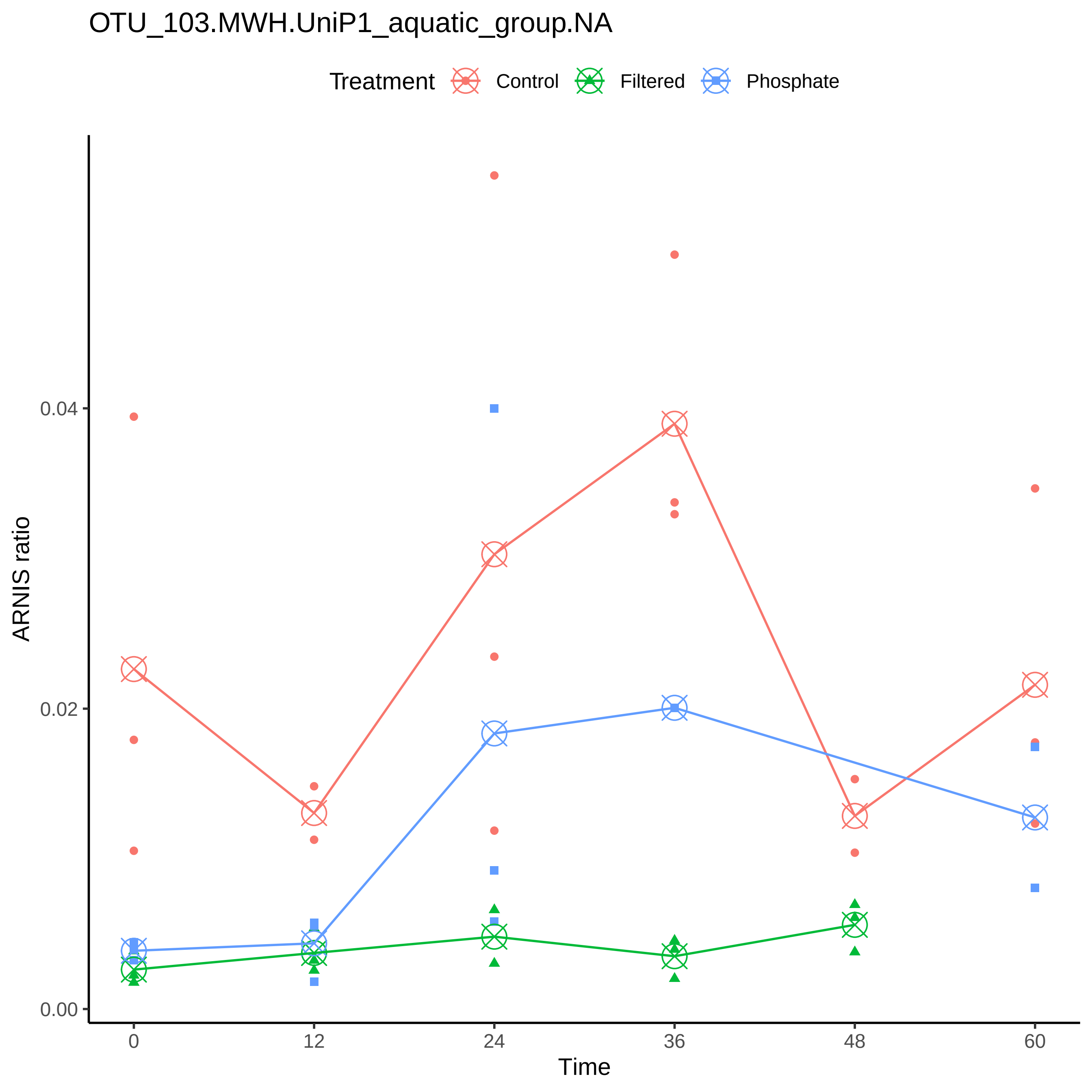

OTU_103.MWH.UniP1_aquatic_group.NA
Treatment
Control
Filtered
Phosphate
ARNIS ratio
0.00
0.02
0.04
Time
0
12
24
36
48
60

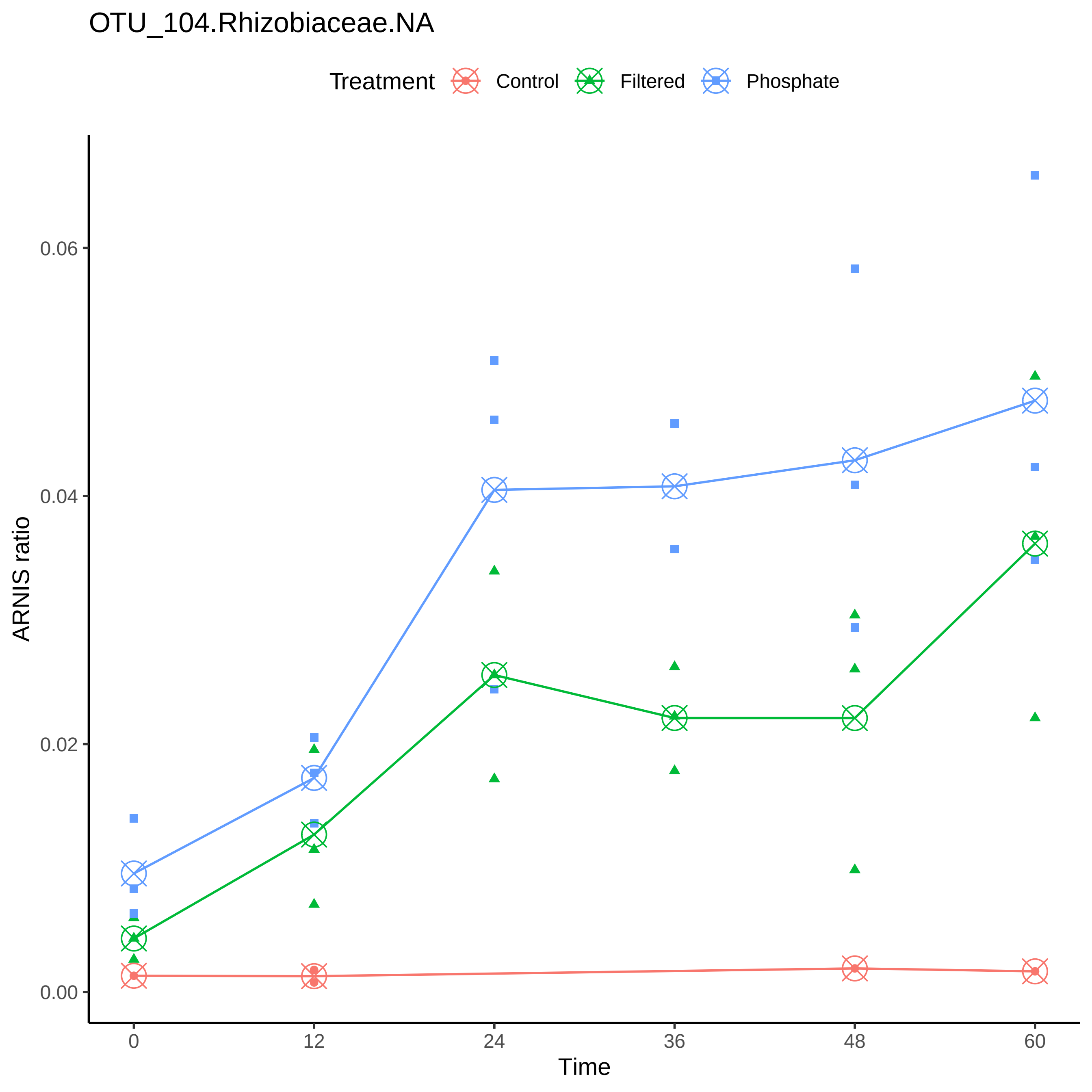
OTU_104.Rhizobiaceae.NA
Treatment
Control
Filtered
Phosphate
ARNIS ratio
0.00
0.02
0.04
0.06
Time
0
12
24
36
48
60

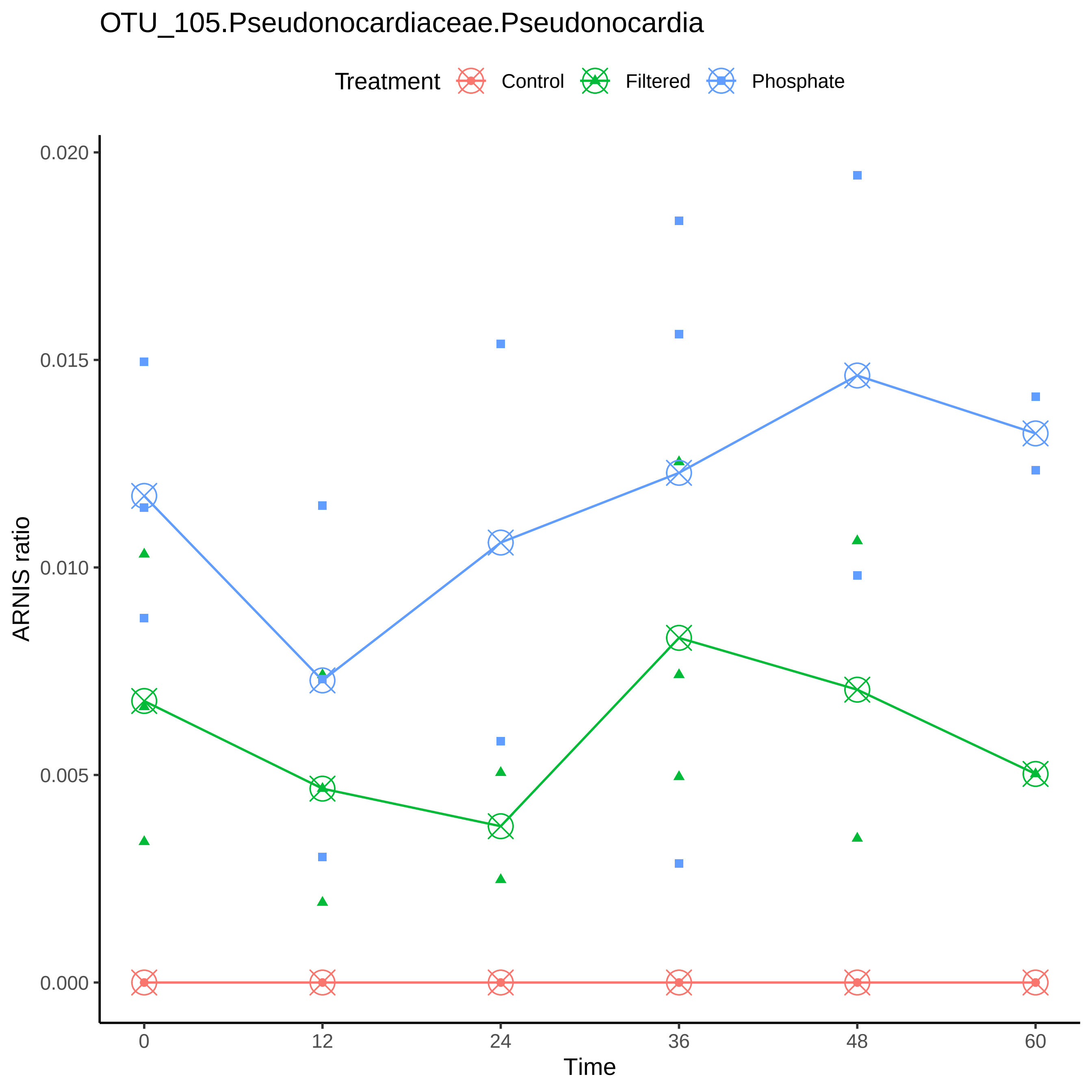
OTU_105.Pseudonocardiaceae.Pseudonocardia
Treatment
Control
Filtered
Phosphate
ARNIS ratio
0.020
0.015
0.010
0.005
0.000
0
12
24
36
48
60
Time

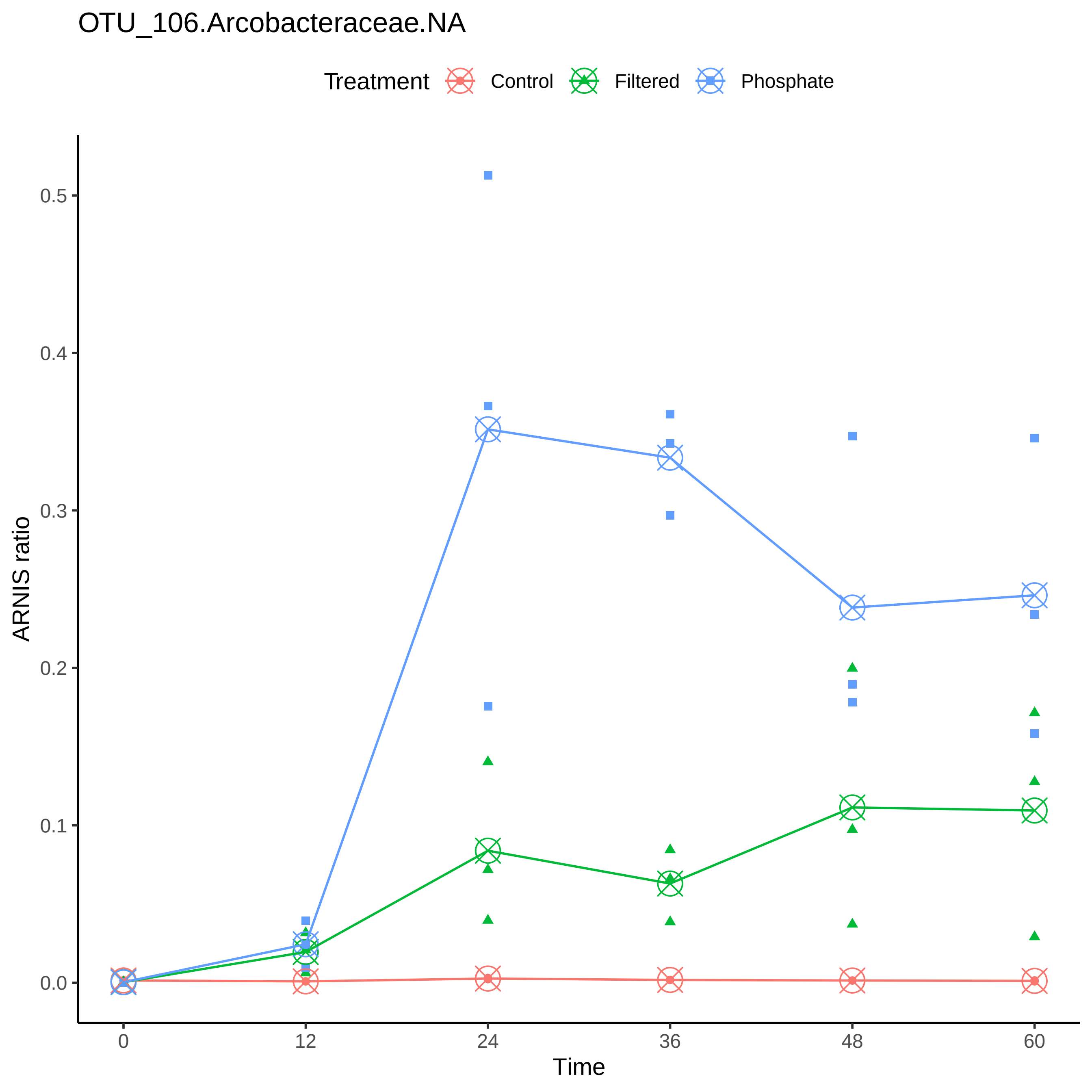

OTU_106.Arcobacteraceae.NA
Treatment
Control
Filtered
Phosphate
ARNIS ratio
0.5
0.4
0.3
0.2
0.1
0.0
0
12
24
36
48
60
Time

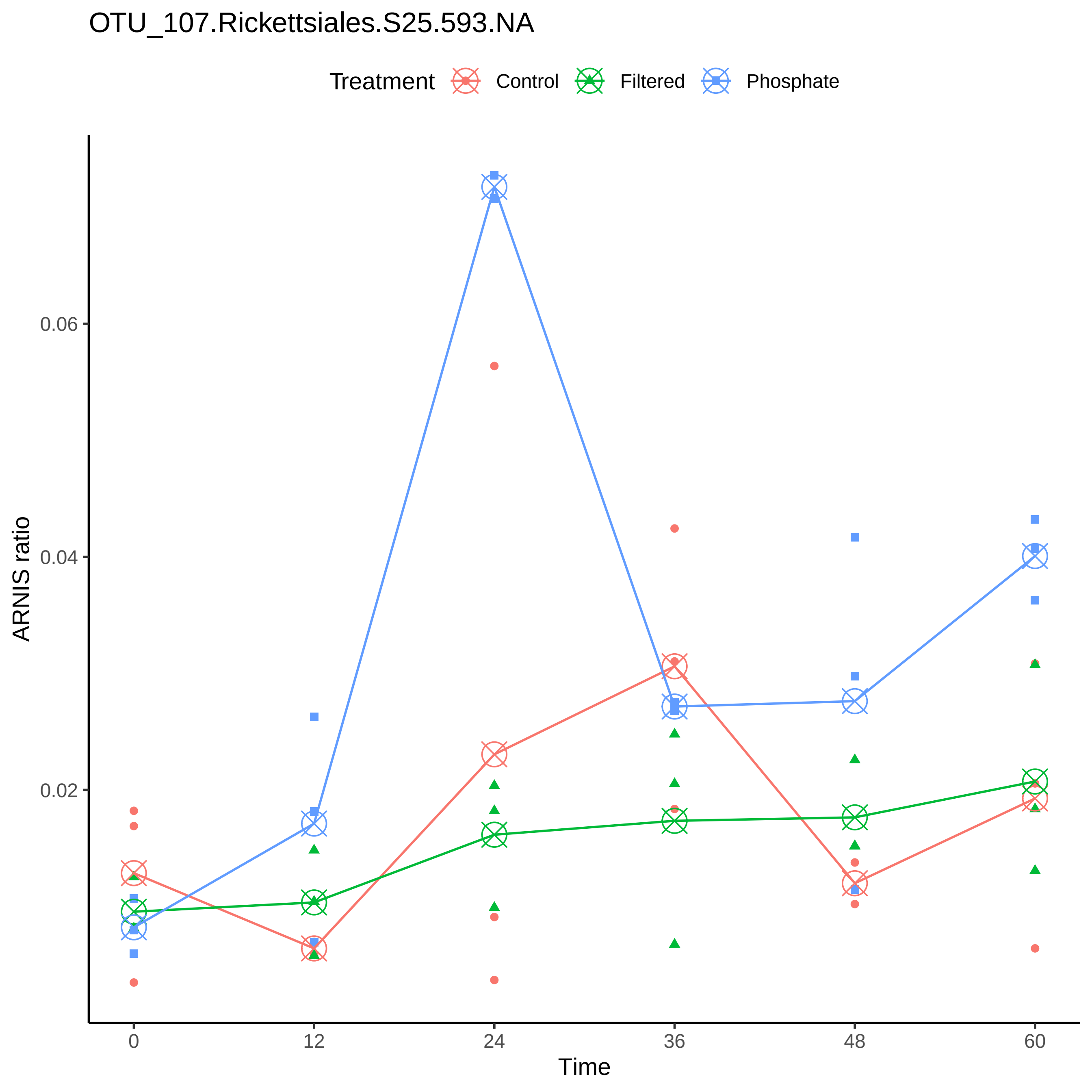
OTU_107.Rickettsiales.S25.593.NA
Treatment
Control
Filtered
Phosphate
ARNIS ratio
0.06
0.04
0.02
0
12
24
36
48
60
Time

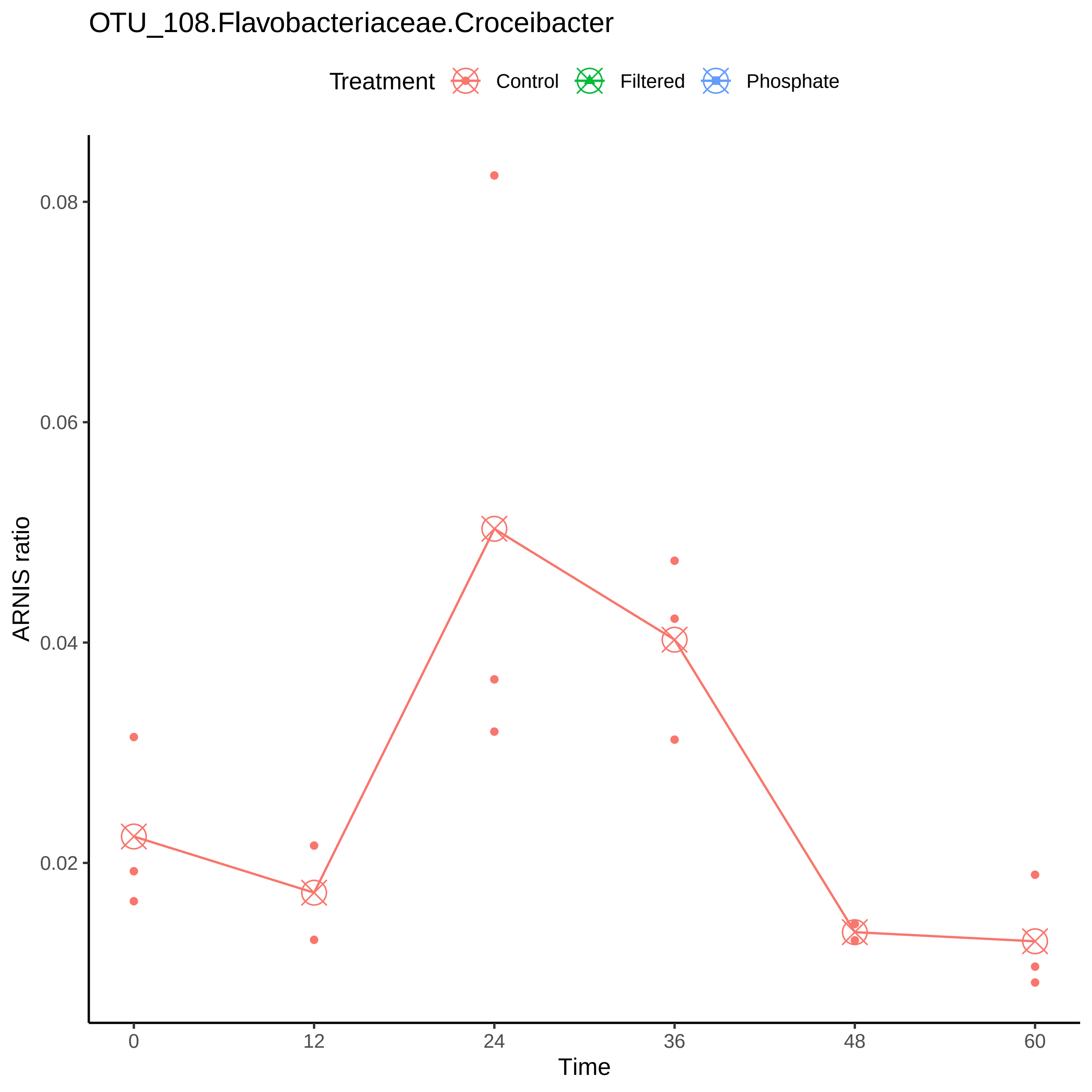

OTU_108.Flavobacteriaceae.Croceibacter
Treatment
Control
Filtered
Phosphate
ARNIS ratio
0.08
0.06
0.04
0.02
0
12
24
36
48
60
Time

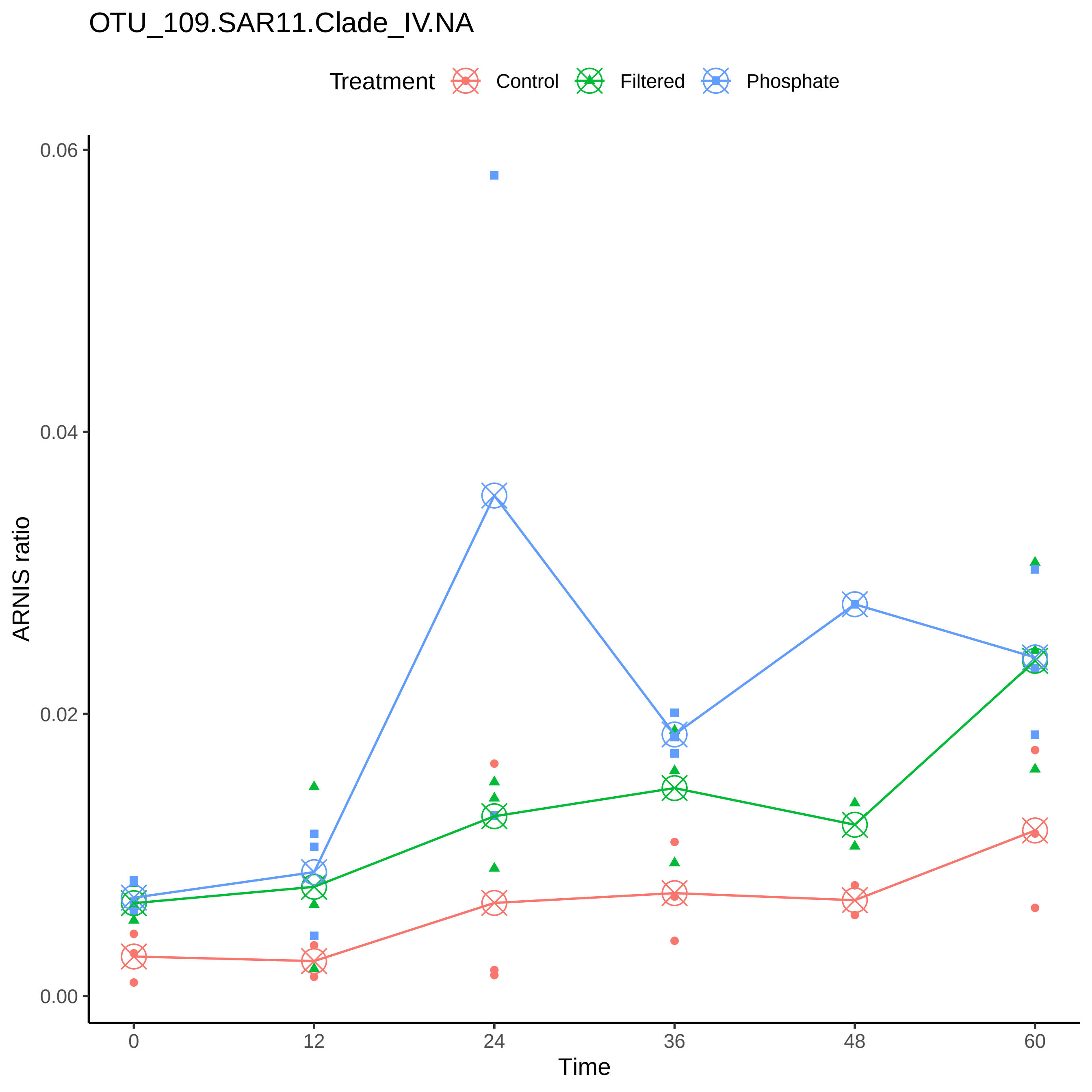
OTU_109.SAR11.Clade_IV.NA
Treatment
Control
Filtered
Phosphate
ARNIS ratio
0.06
0.04
0.02
0.00
Time
0
12
24
36
48
60

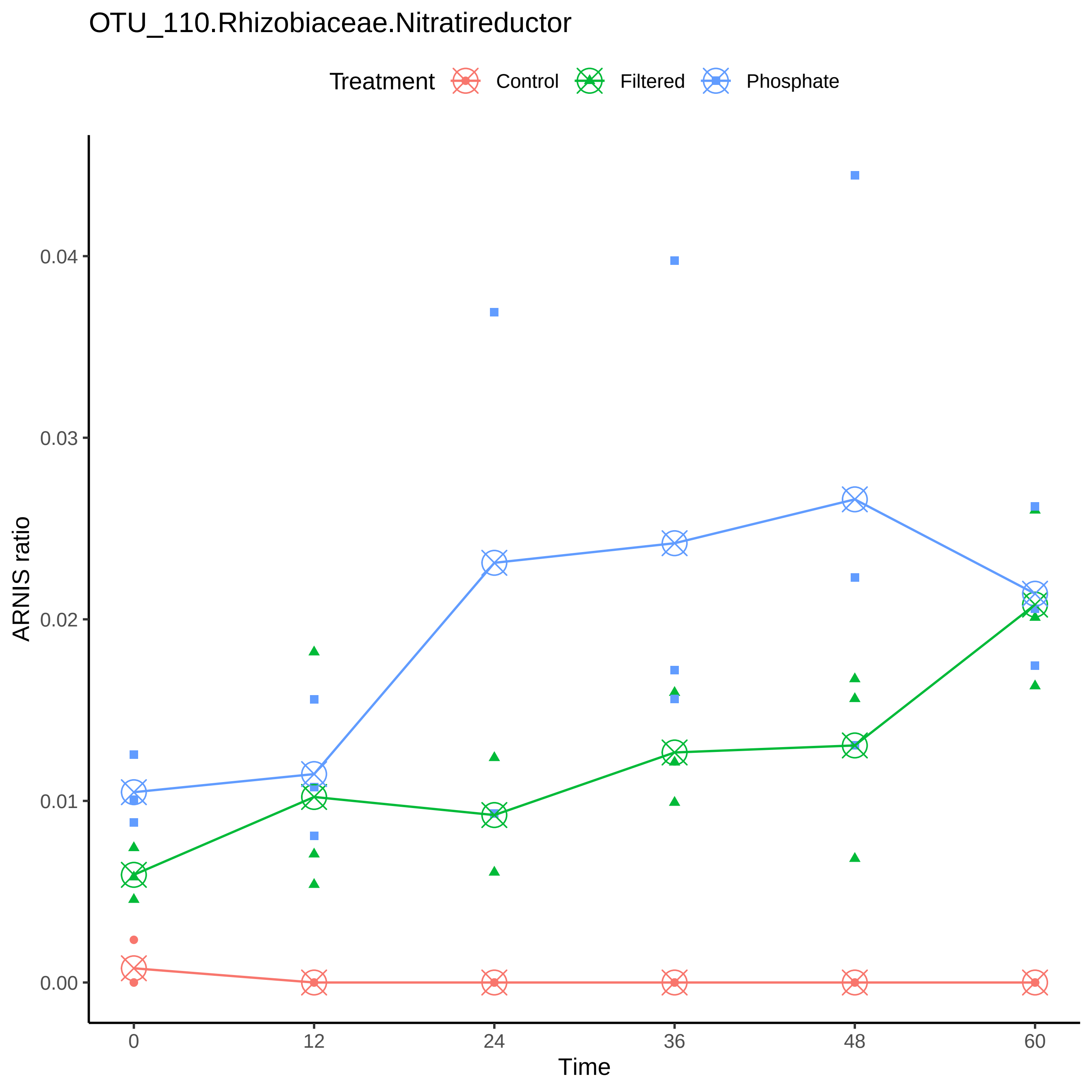

OTU_110.Rhizobiaceae.Nitratireductor
Treatment
Control
Filtered
Phosphate
ARNIS ratio
0.00
0.01
0.02
0.03
0.04
0
12
24
36
48
60
Time

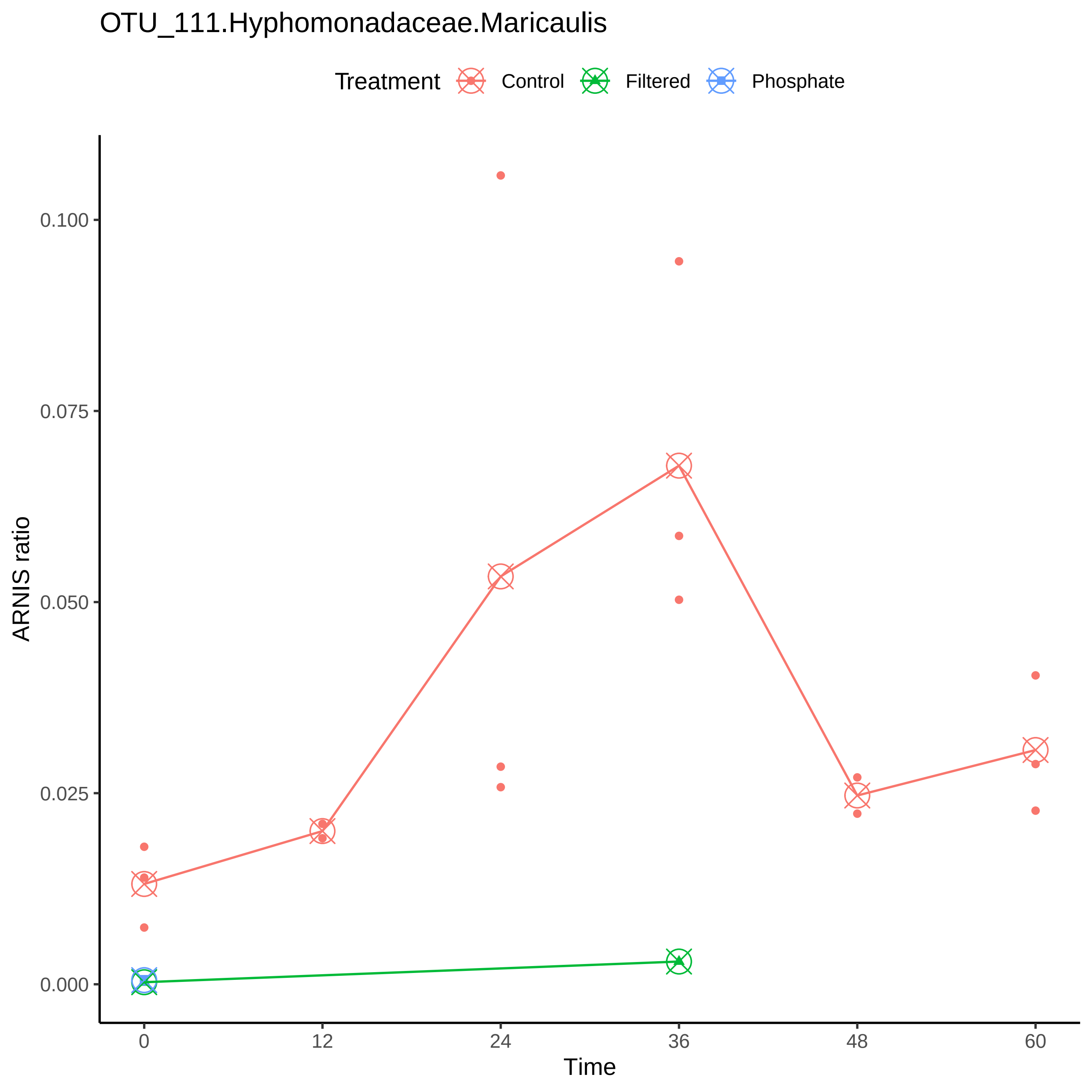
OTU_111.Hyphomonadaceae.Maricaulis
Treatment
Control
Filtered
Phosphate
ARNIS ratio
0.100
0.075
0.050
0.025
0.000
Time
0
12
24
36
48
60

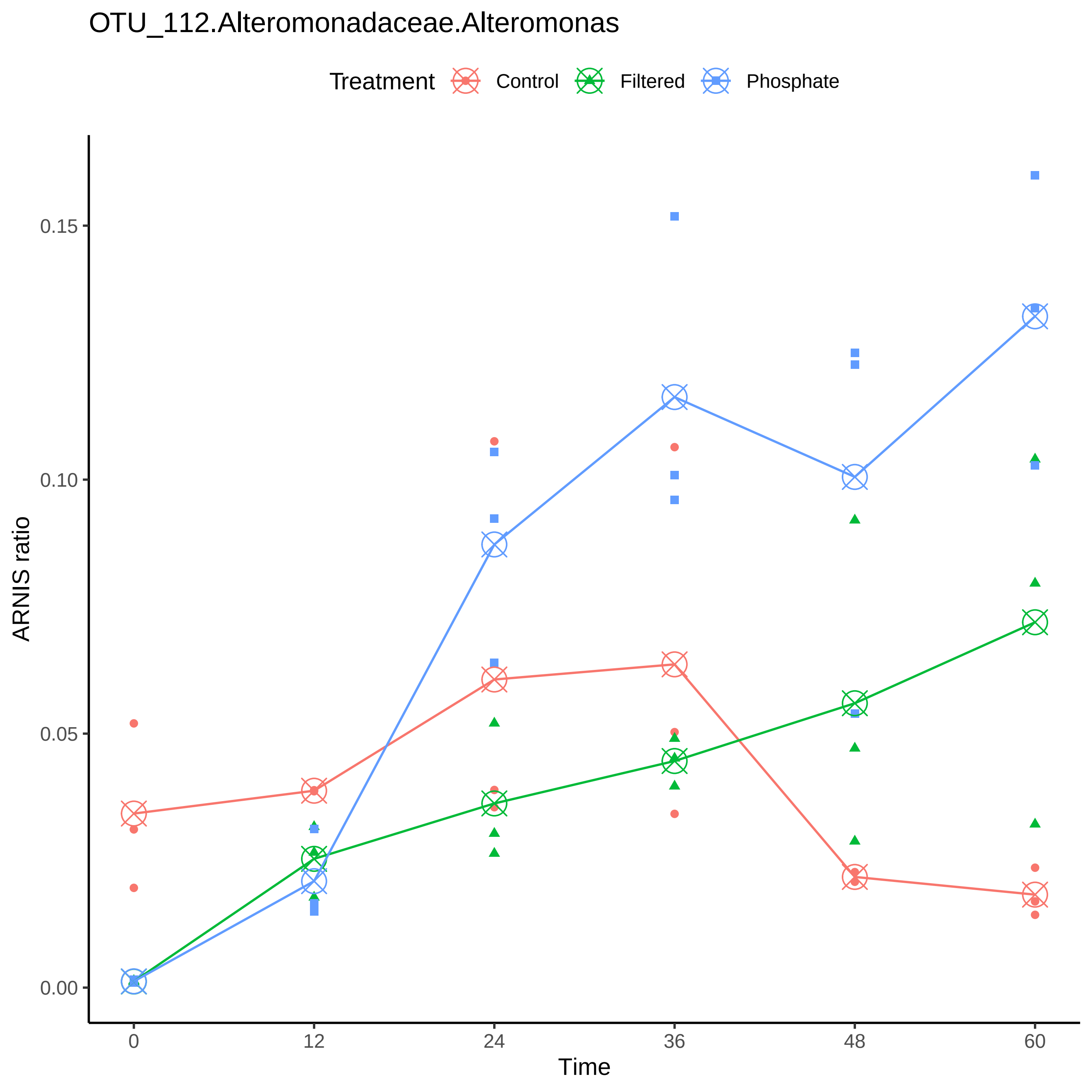

OTU_112.Alteromonadaceae.Alteromonas
Treatment
Control
Filtered
Phosphate
ARNIS ratio
0.15
0.10
0.05
0.00
Time
0
12
24
36
48
60

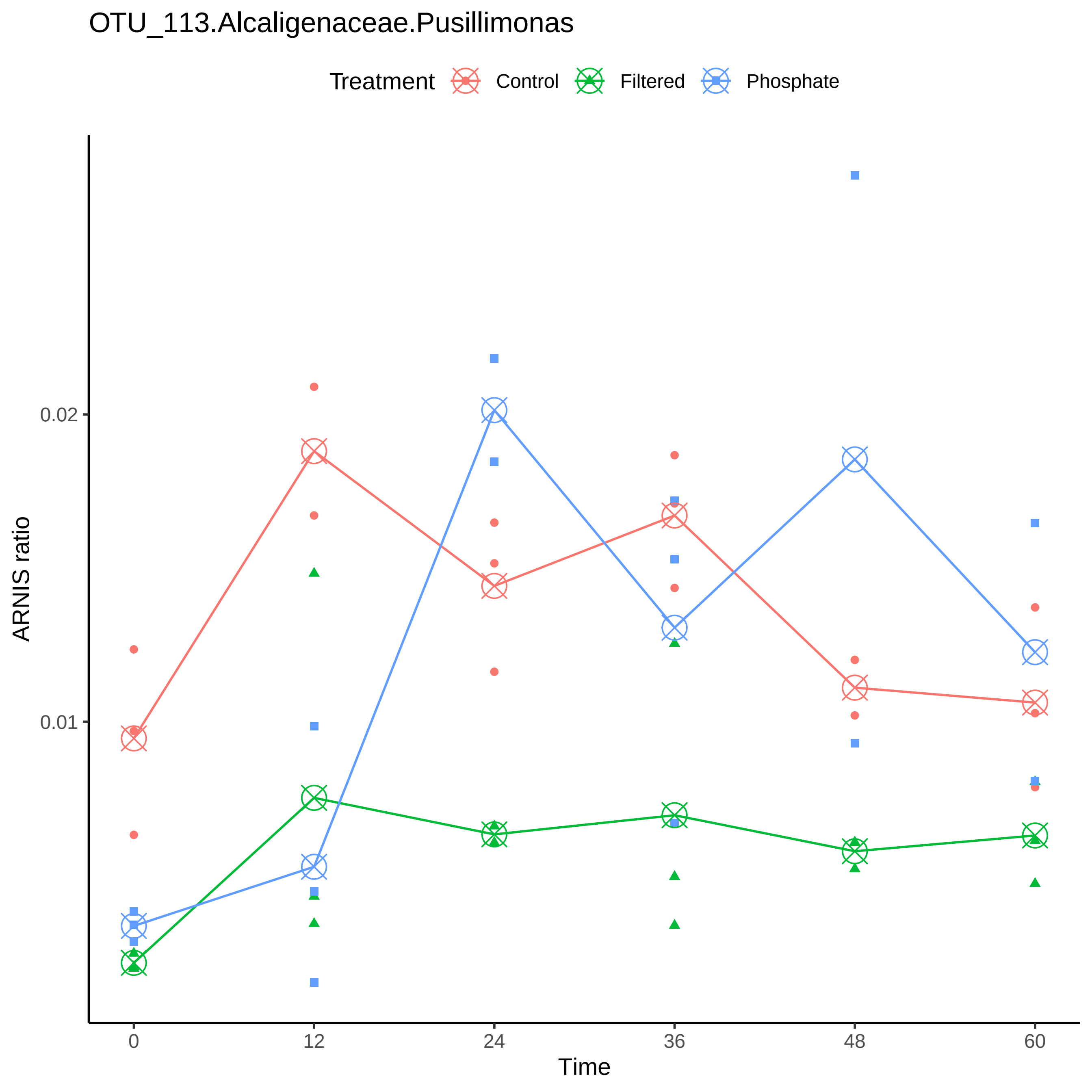
OTU_113.Alcaligenaceae.Pusillimonas
Treatment
Control
Filtered
Phosphate
ARNIS ratio
0.02
0.01
0
12
24
36
48
60
Time

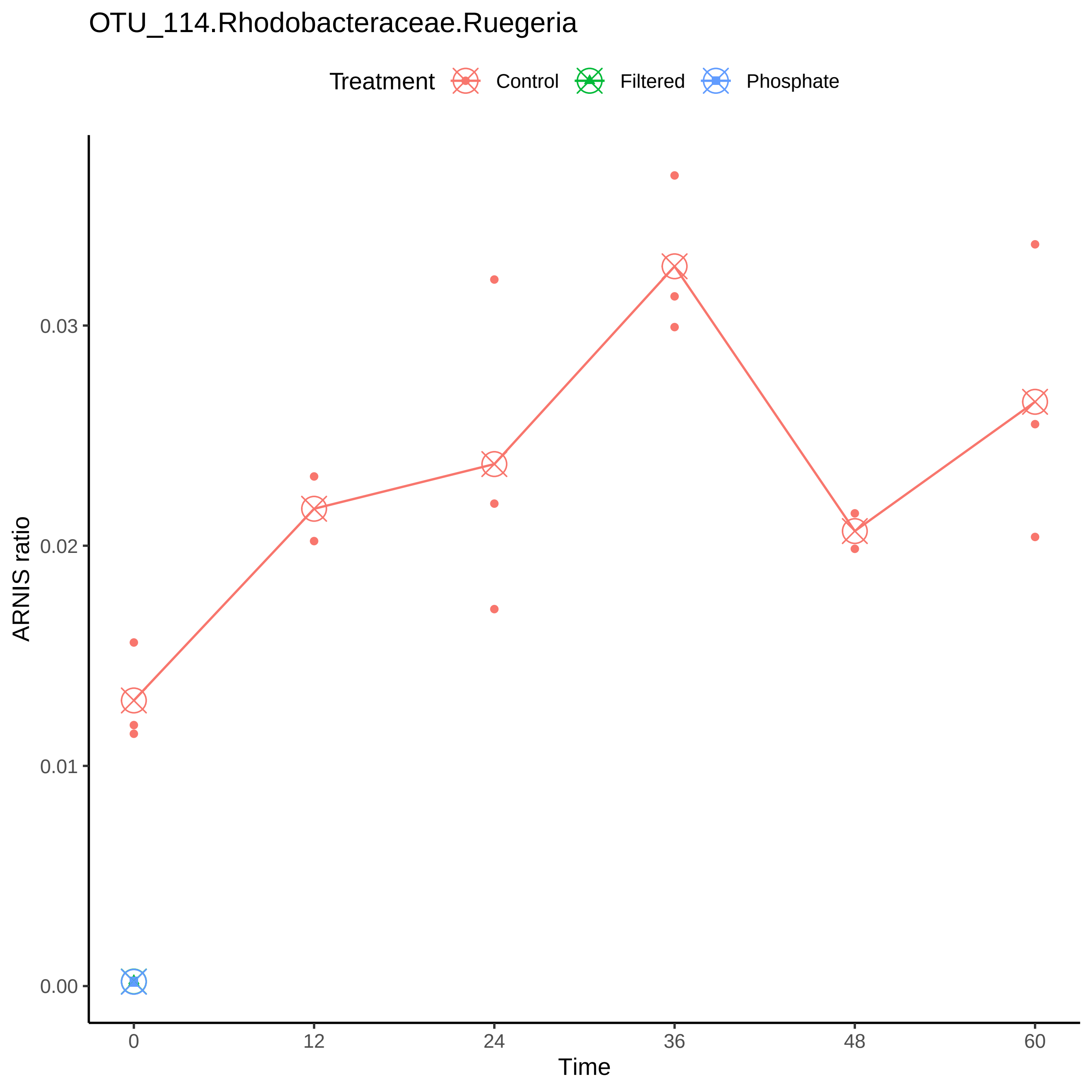

OTU_114.Rhodobacteraceae.Ruegeria
Treatment
Control
Filtered
Phosphate
ARNIS ratio
0.00
0.01
0.02
0.03
Time
0
12
24
36
48
60

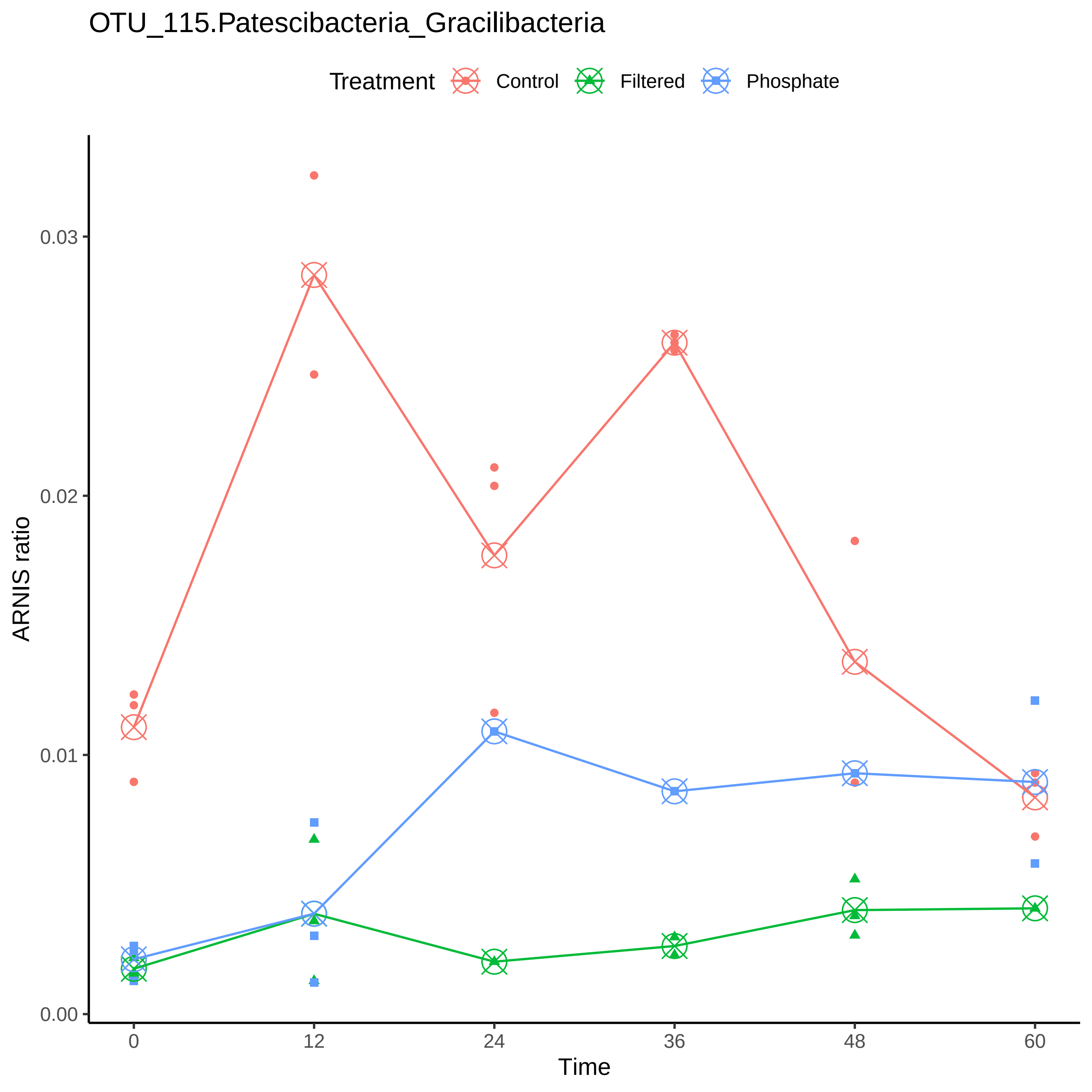

OTU_115.Patescibacteria_Gracilibacteria
Treatment
Control
Filtered
Phosphate
ARNIS ratio
0.00
0.01
0.02
0.03
Time
0
12
24
36
48
60

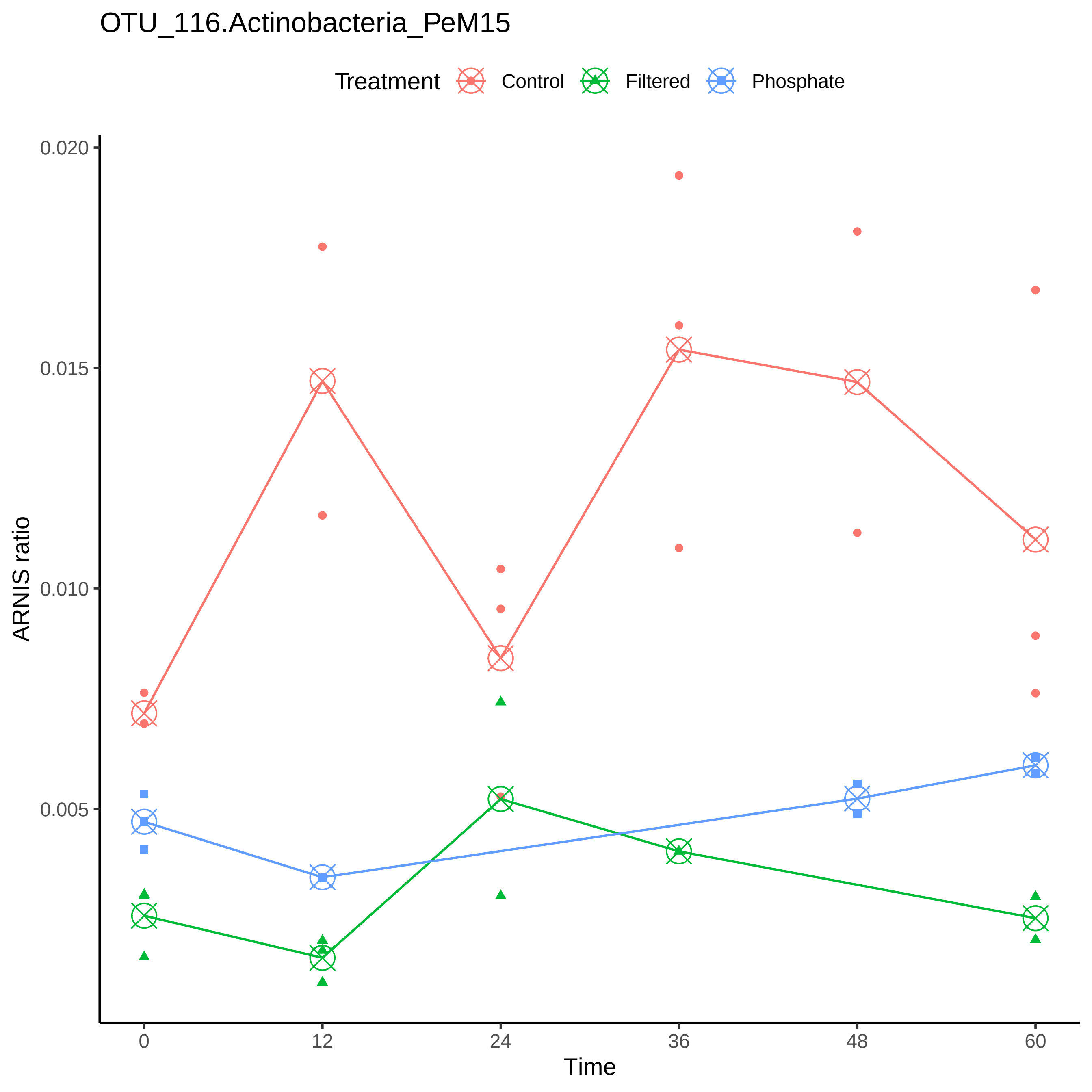
OTU_116.Actinobacteria_PeM15
Treatment
Control
Filtered
Phosphate
ARNIS ratio
0.020
0.015
0.010
0.005
0
12
24
36
48
60
Time

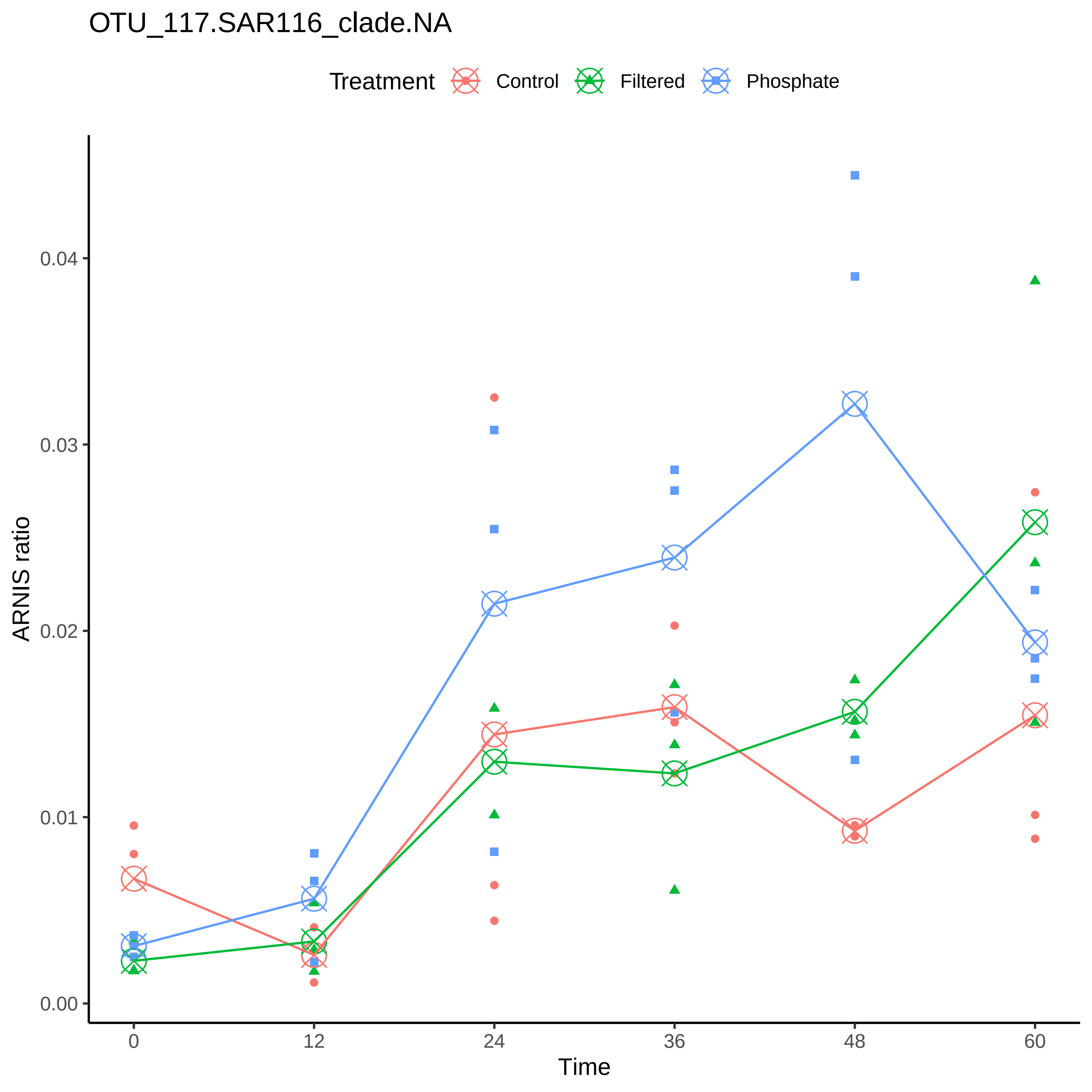

OTU_117.SAR116_clade.NA
Treatment
Control
Filtered
Phosphate
ARNIS ratio
0.00
0.01
0.02
0.03
0.04
0
12
24
36
48
60
Time

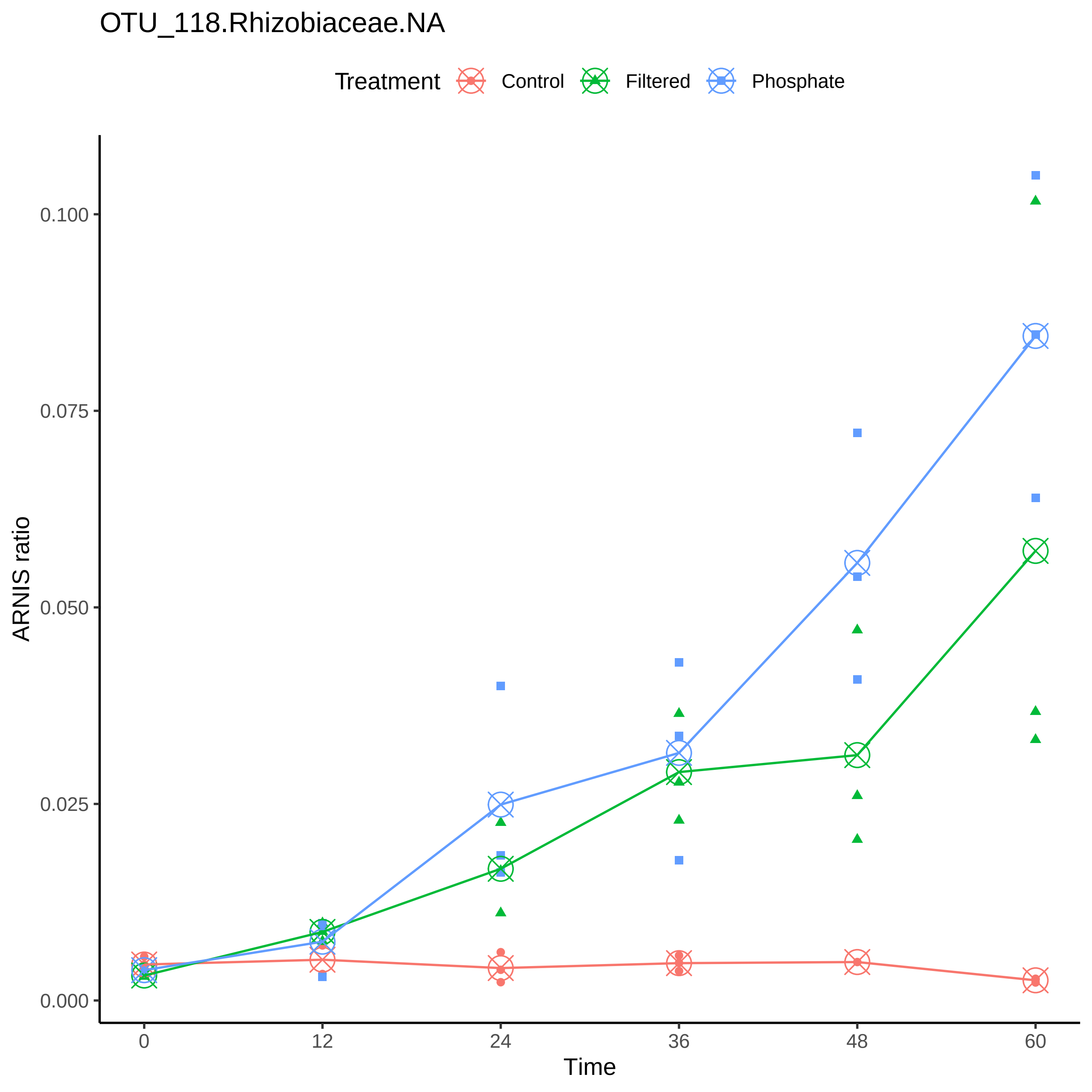

OTU_118.Rhizobiaceae.NA
Treatment
Control
Filtered
Phosphate
ARNIS ratio
0.100
0.075
0.050
0.025
0.000
0
12
24
36
48
60
Time

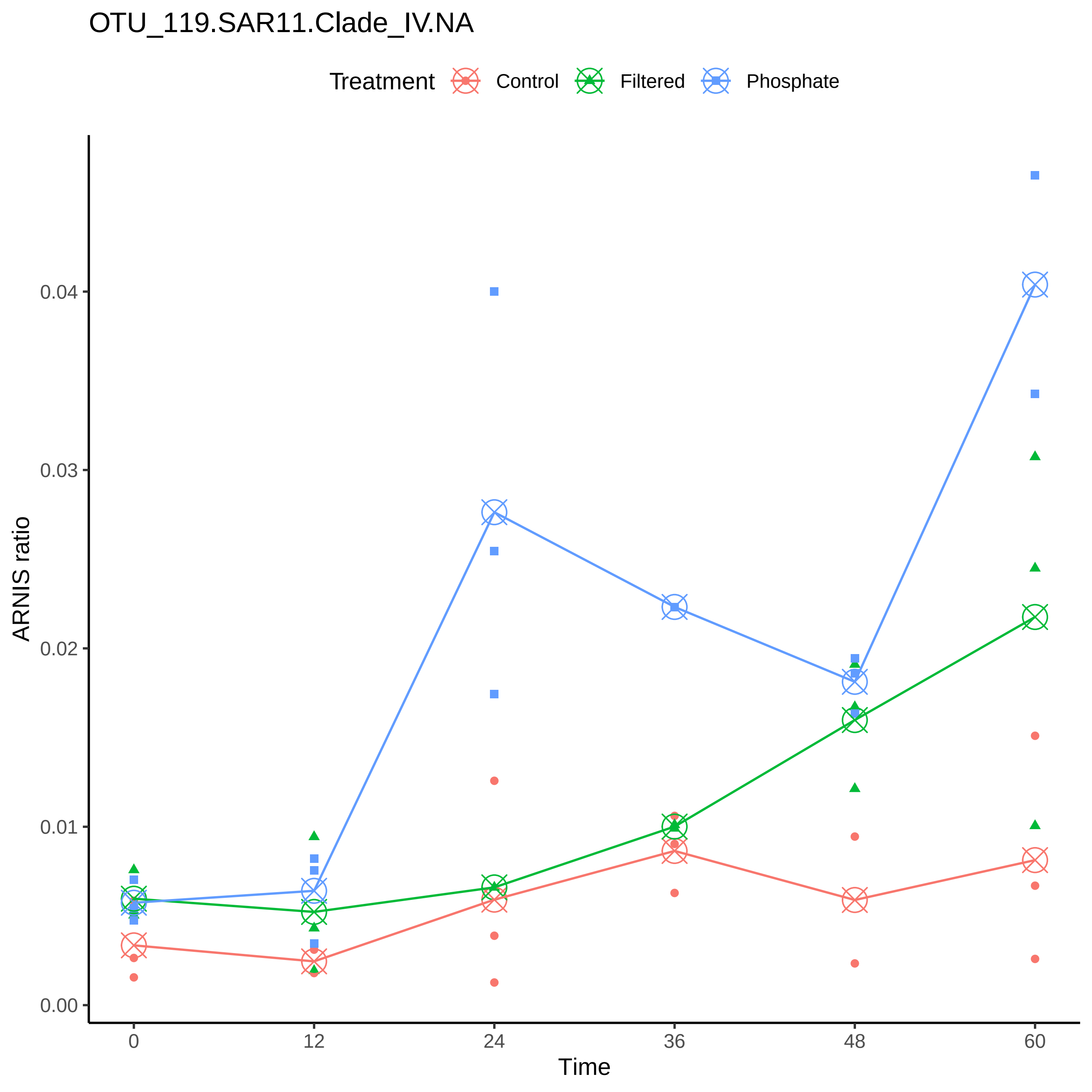
OTU_119.SAR11.Clade_IV.NA
Treatment
Control
Filtered
Phosphate
ARNIS ratio
0.00
0.01
0.02
0.03
0.04
Time
0
12
24
36
48
60

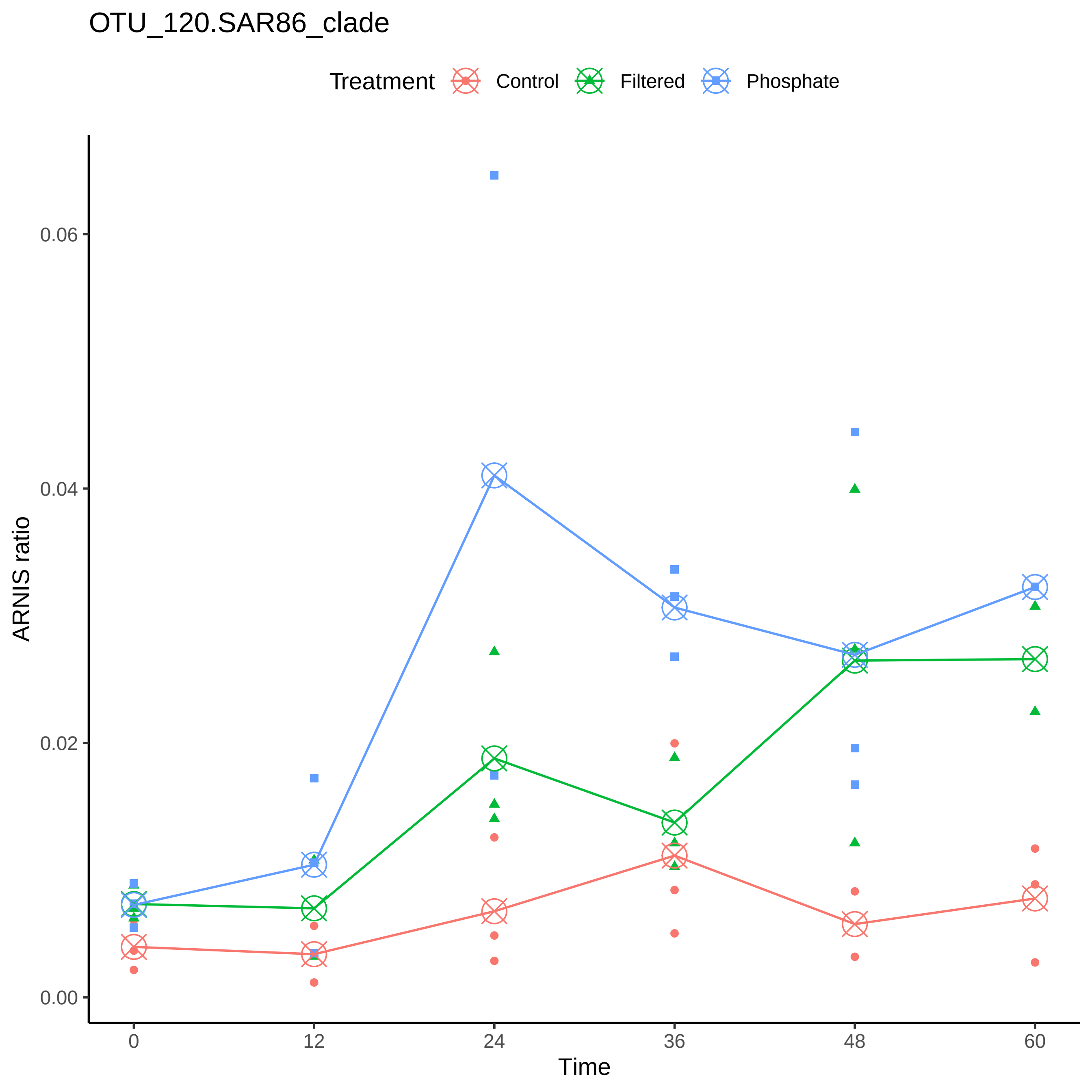
OTU_120.SAR86_clade
Treatment
Control
Filtered
Phosphate
ARNIS ratio
0.00
0.02
0.04
0.06
0
12
24
36
48
60
Time

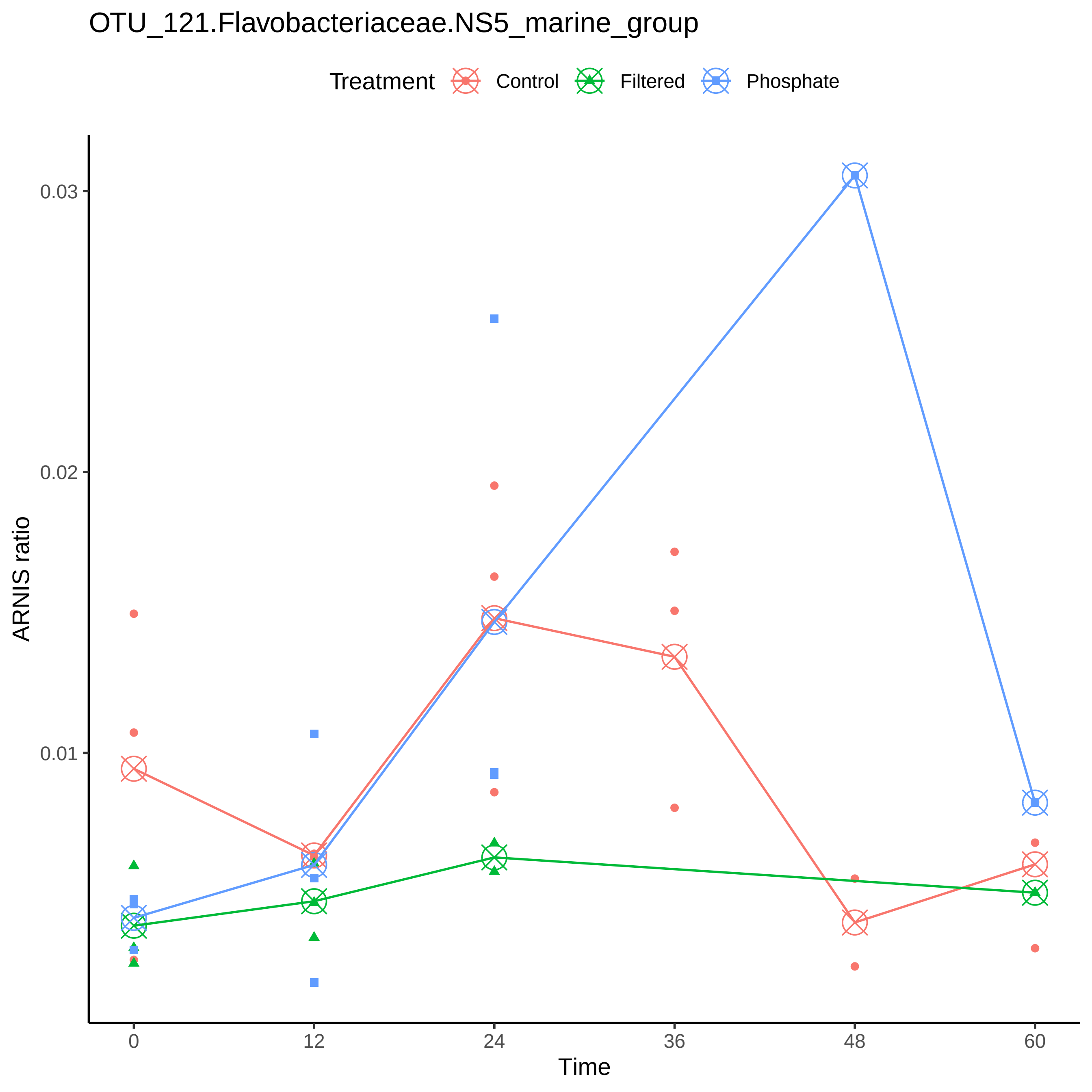
OTU_121.Flavobacteriaceae.NS5_marine_group
Treatment
Control
Filtered
Phosphate
ARNIS ratio
0.03
0.02
0.01
Time
0
12
24
36
48
60

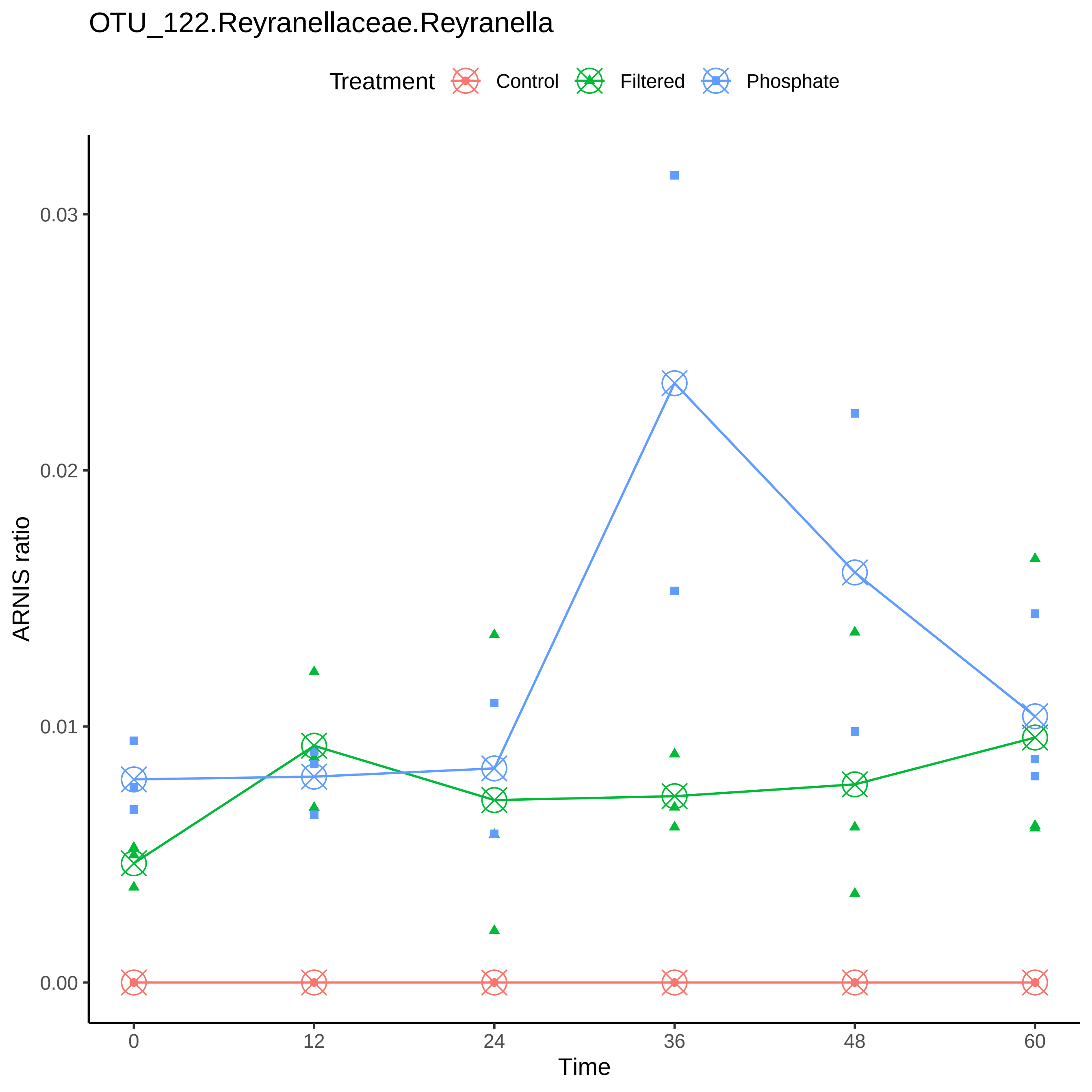
OTU_122.Reyranellaceae.Reyranella
Treatment
Control
Filtered
Phosphate
ARNIS ratio
0.00
0.01
0.02
0.03
Time
0
12
24
36
48
60

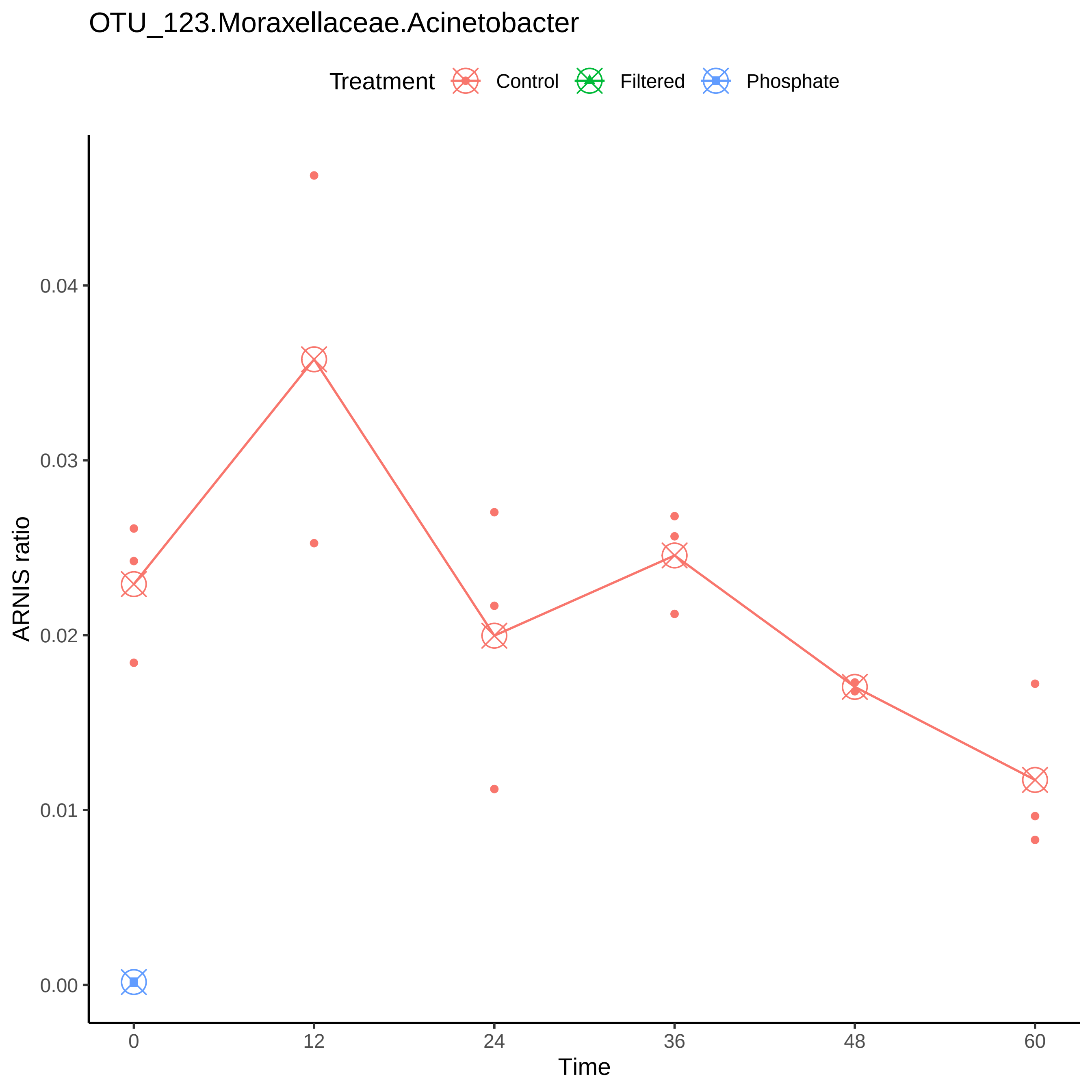
OTU_123.Moraxellaceae.Acinetobacter
Treatment
Control
Filtered
Phosphate
ARNIS ratio
0.00
0.01
0.02
0.03
0.04
0
12
24
36
48
60
Time

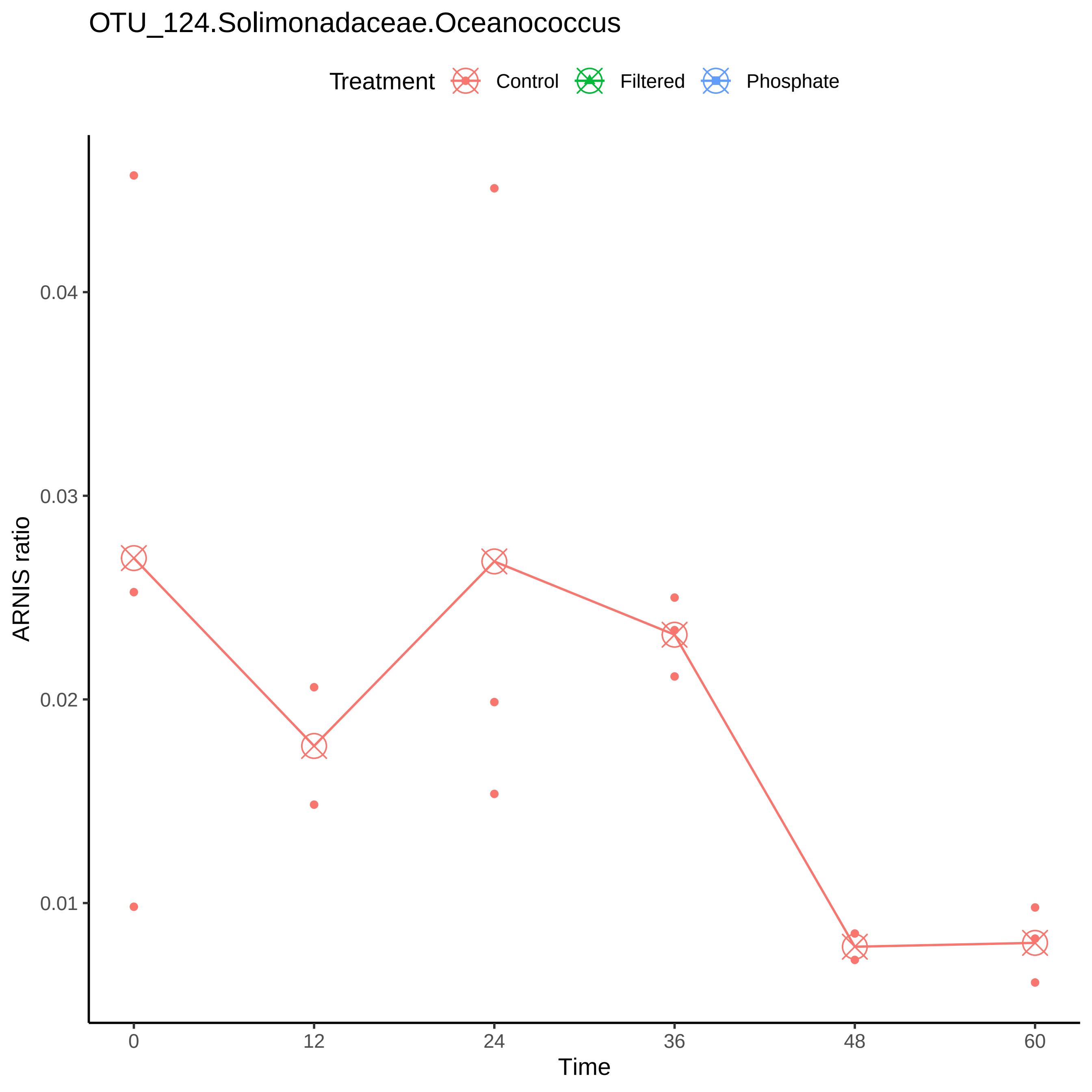

OTU_124.Solimonadaceae.Oceanococcus
Treatment
Control
Filtered
Phosphate
ARNIS ratio
0.04
0.03
0.02
0.01
0
12
24
36
48
60
Time

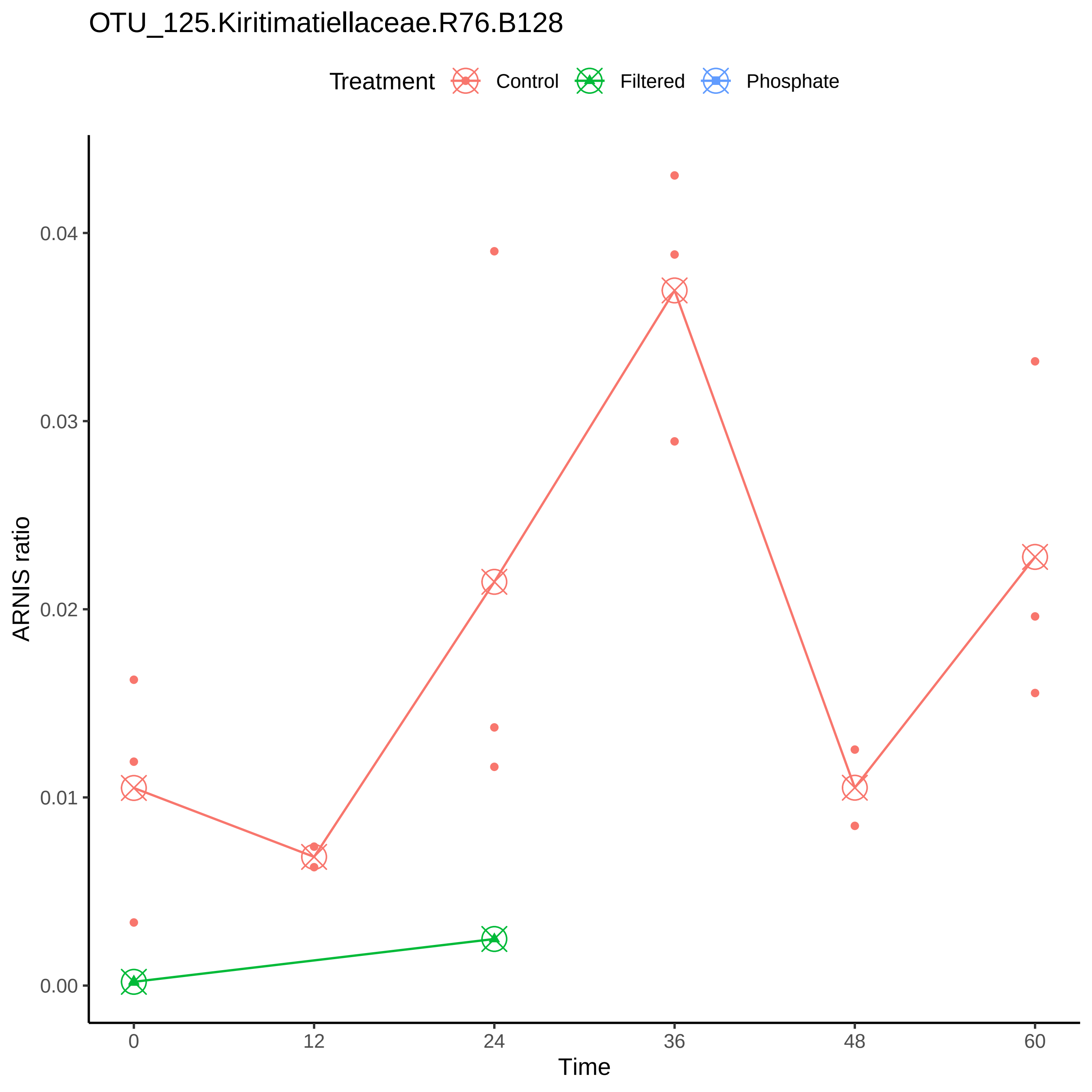

OTU_125.Kiritimatiellaceae.R76.B128
Treatment
Control
Filtered
Phosphate
ARNIS ratio
0.00
0.01
0.02
0.03
0.04
Time
0
12
24
36
48
60

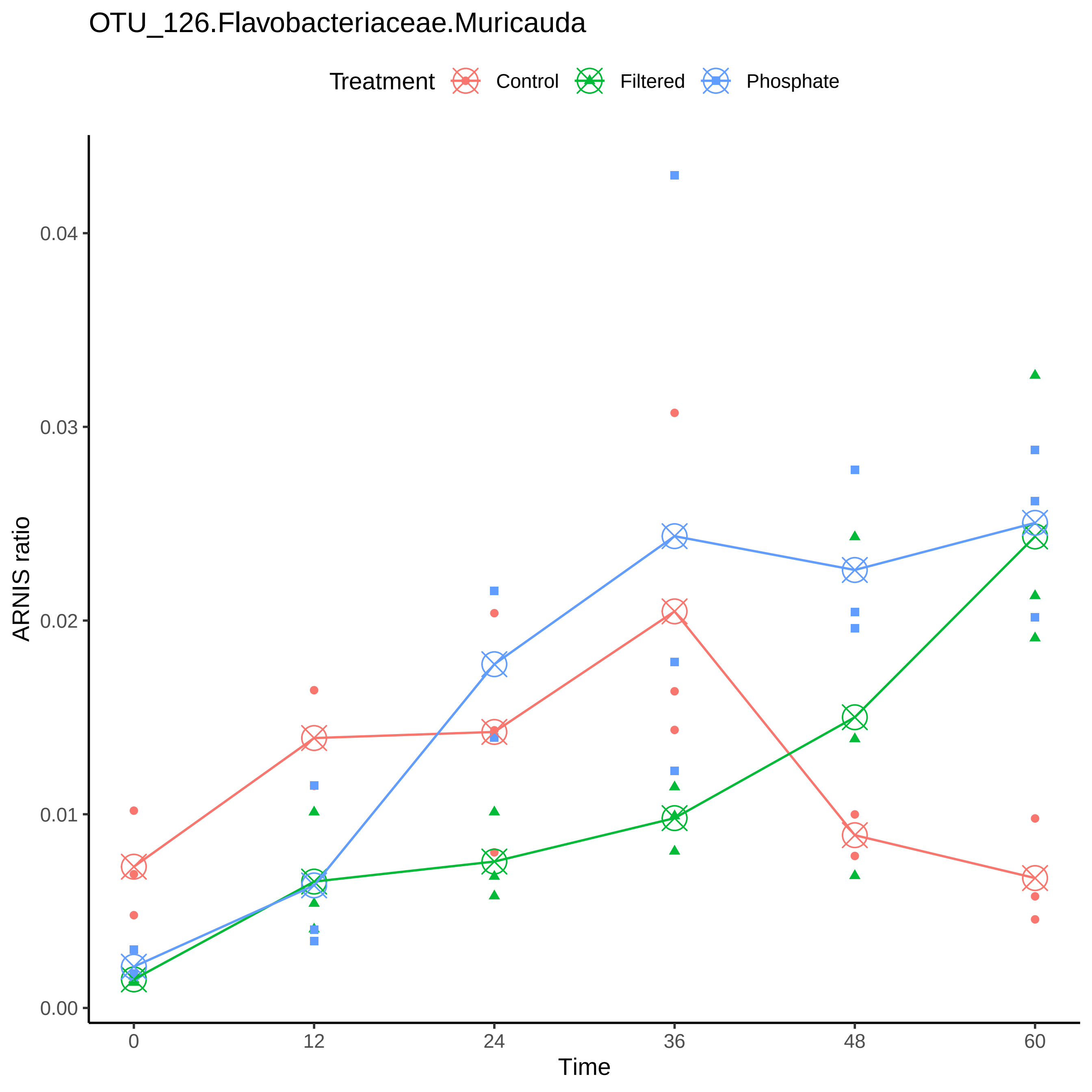
OTU_126.Flavobacteriaceae.Muricauda
Treatment
Control
Filtered
Phosphate
ARNIS ratio
0.00
0.01
0.02
0.03
0.04
0
12
24
36
48
60
Time

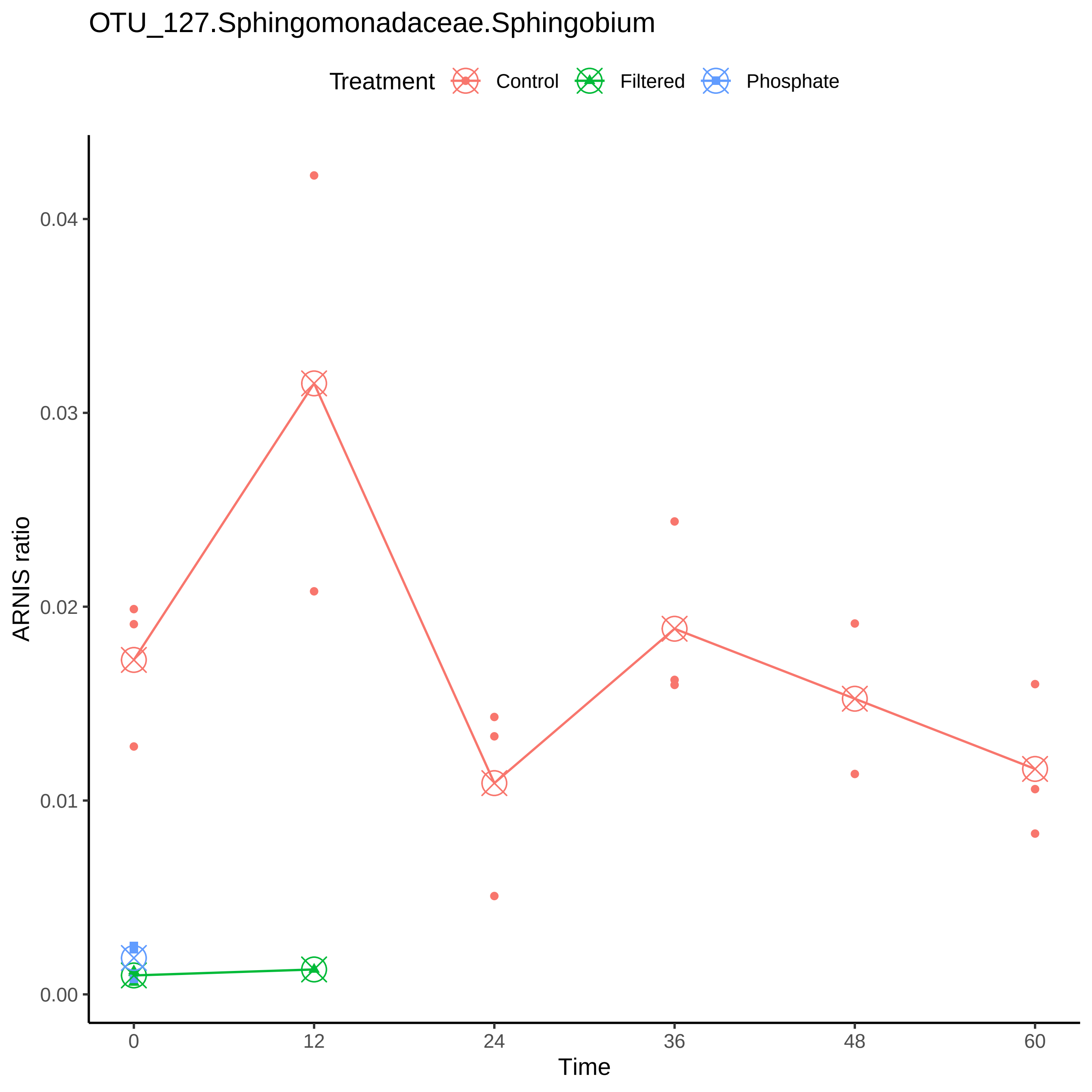
OTU_127.Sphingomonadaceae.Sphingobium
Treatment
Control
Filtered
Phosphate
ARNIS ratio
0.04
0.03
0.02
0.01
0.00
0
12
24
36
48
60
Time

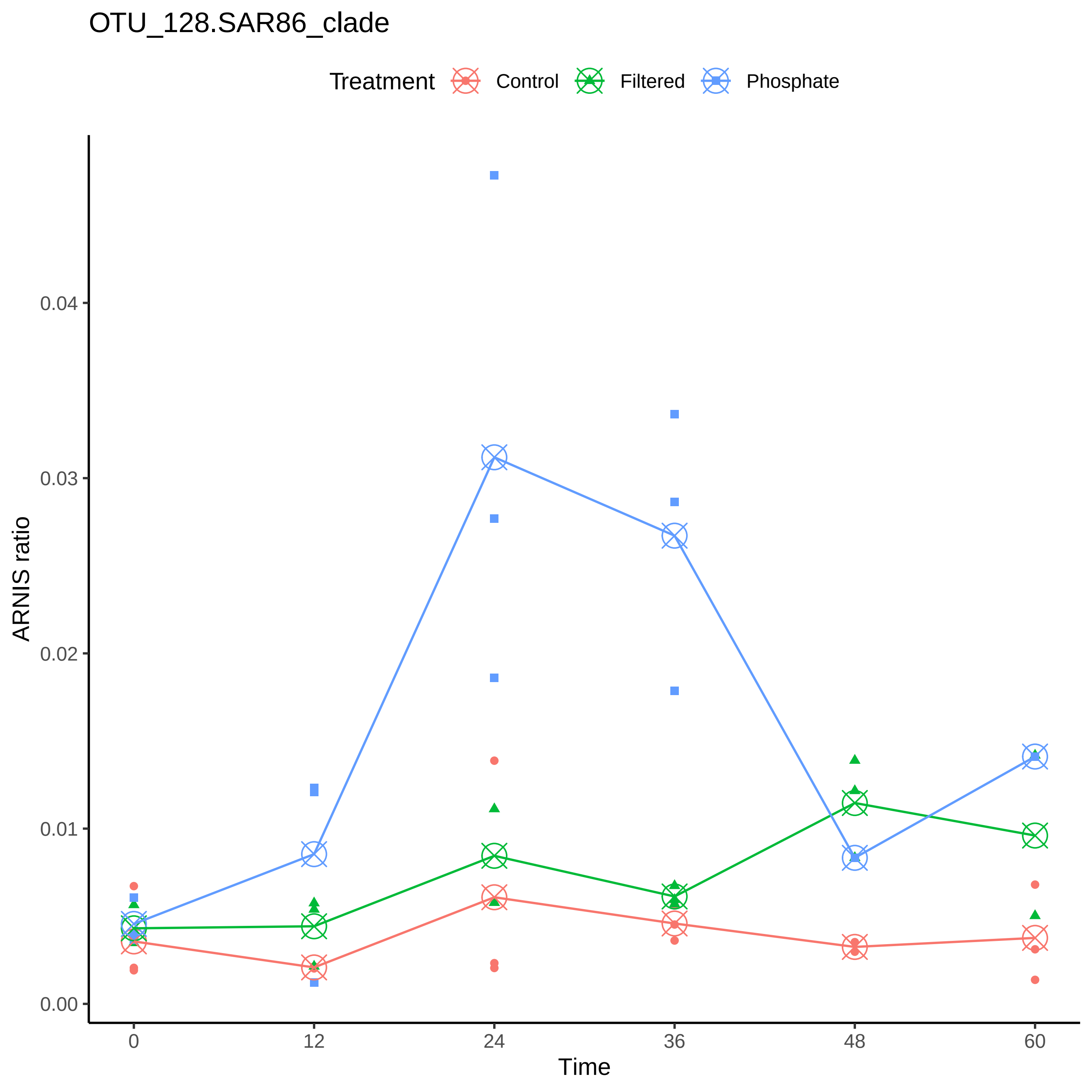

OTU_128.SAR86_clade
Treatment
Control
Filtered
Phosphate
ARNIS ratio
0.00
0.01
0.02
0.03
0.04
0
12
24
36
48
60
Time

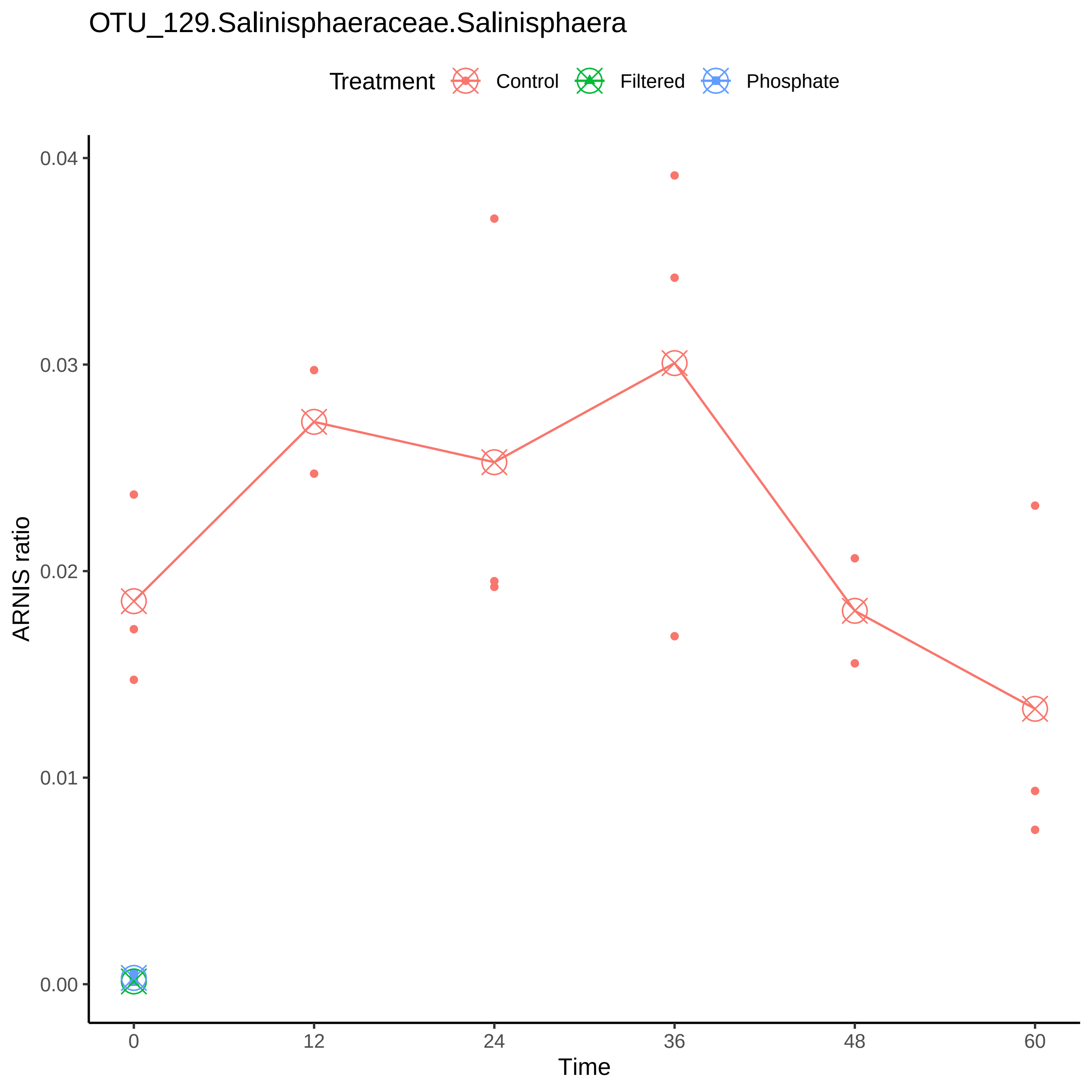
OTU_129.Salinisphaeraceae.Salinisphaera
Treatment
Control
Filtered
Phosphate
ARNIS ratio
0.04
0.03
0.02
0.01
0.00
Time
0
12
24
36
48
60

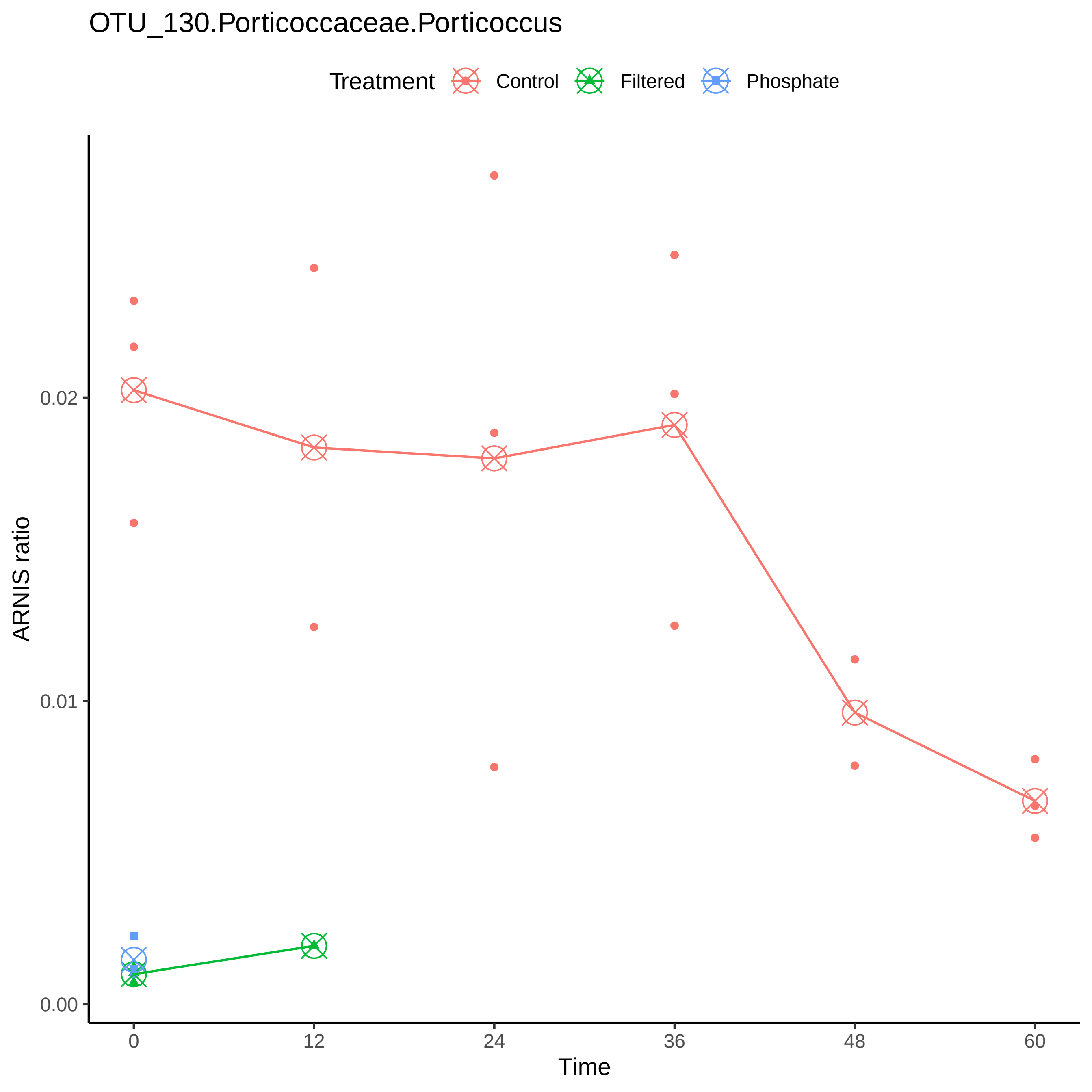

OTU_130.Porticoccaceae.Porticoccus
Treatment
Control
Filtered
Phosphate
ARNIS ratio
0.00
0.01
0.02
0
12
24
36
48
60
Time

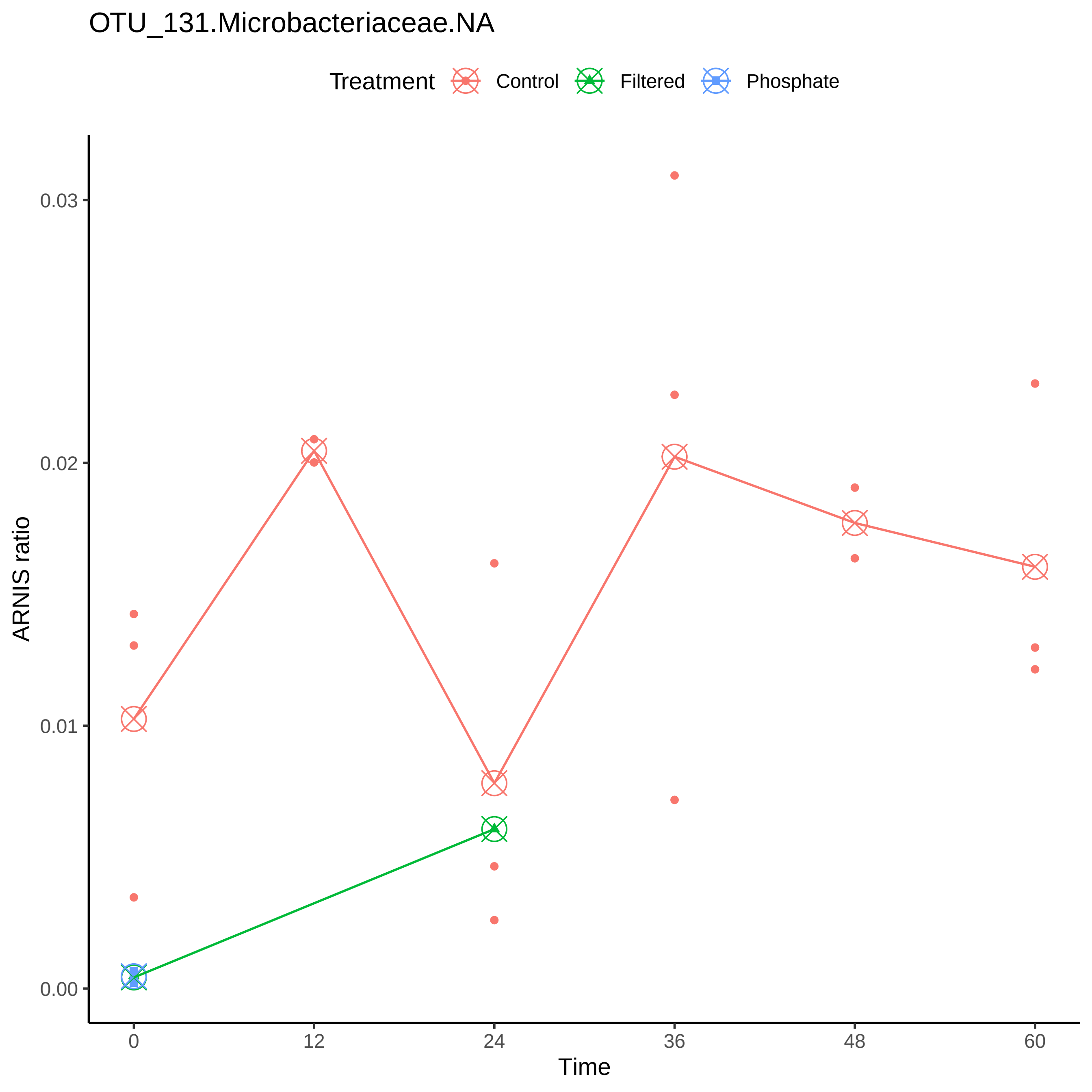
OTU_131.Microbacteriaceae.NA
Treatment
Control
Filtered
Phosphate
ARNIS ratio
0.03
0.02
0.01
0.00
0
12
24
36
48
60
Time

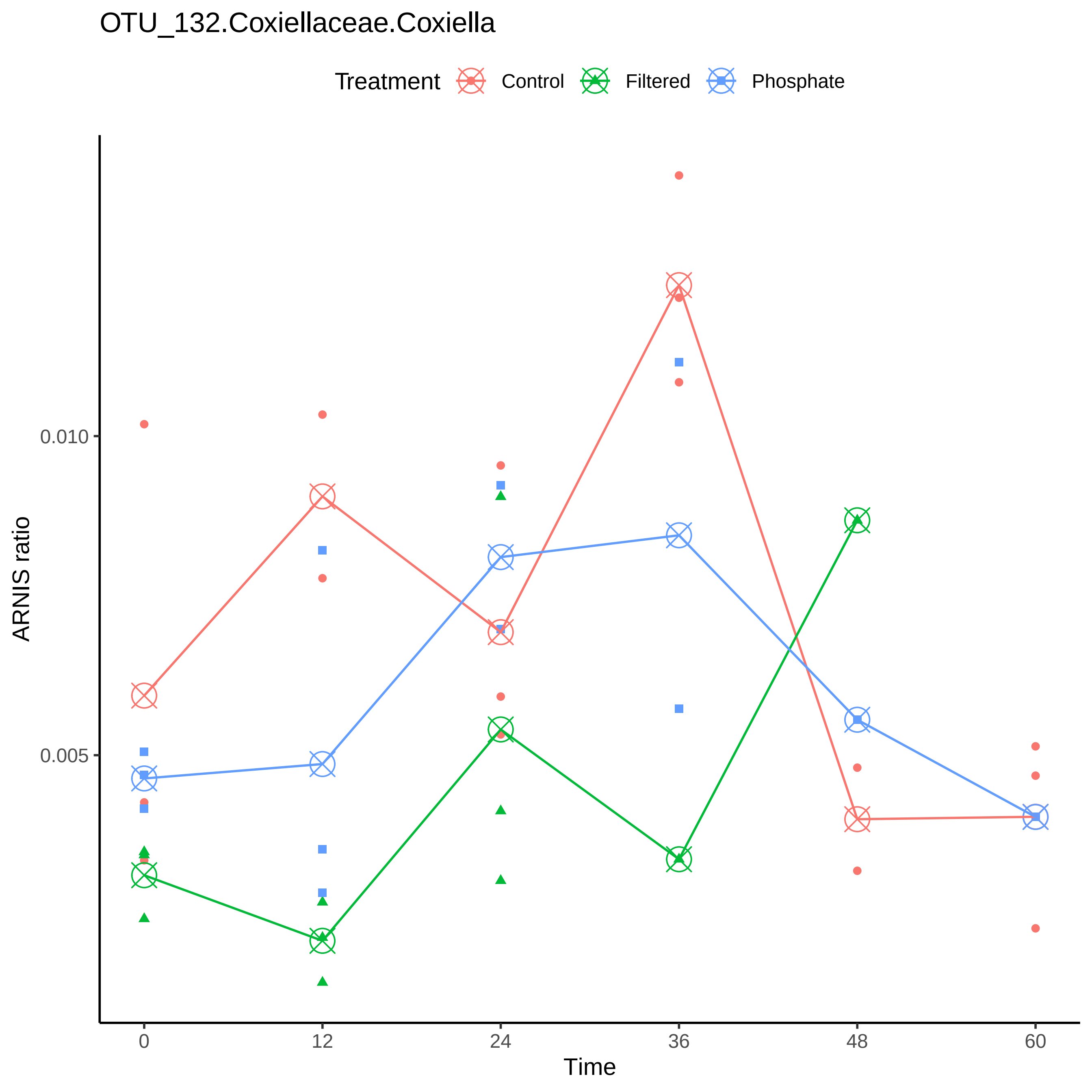
OTU_132.Coxiellaceae.Coxiella
Treatment
Control
Filtered
Phosphate
ARNIS ratio
0.010
0.005
Time
0
12
24
36
48
60

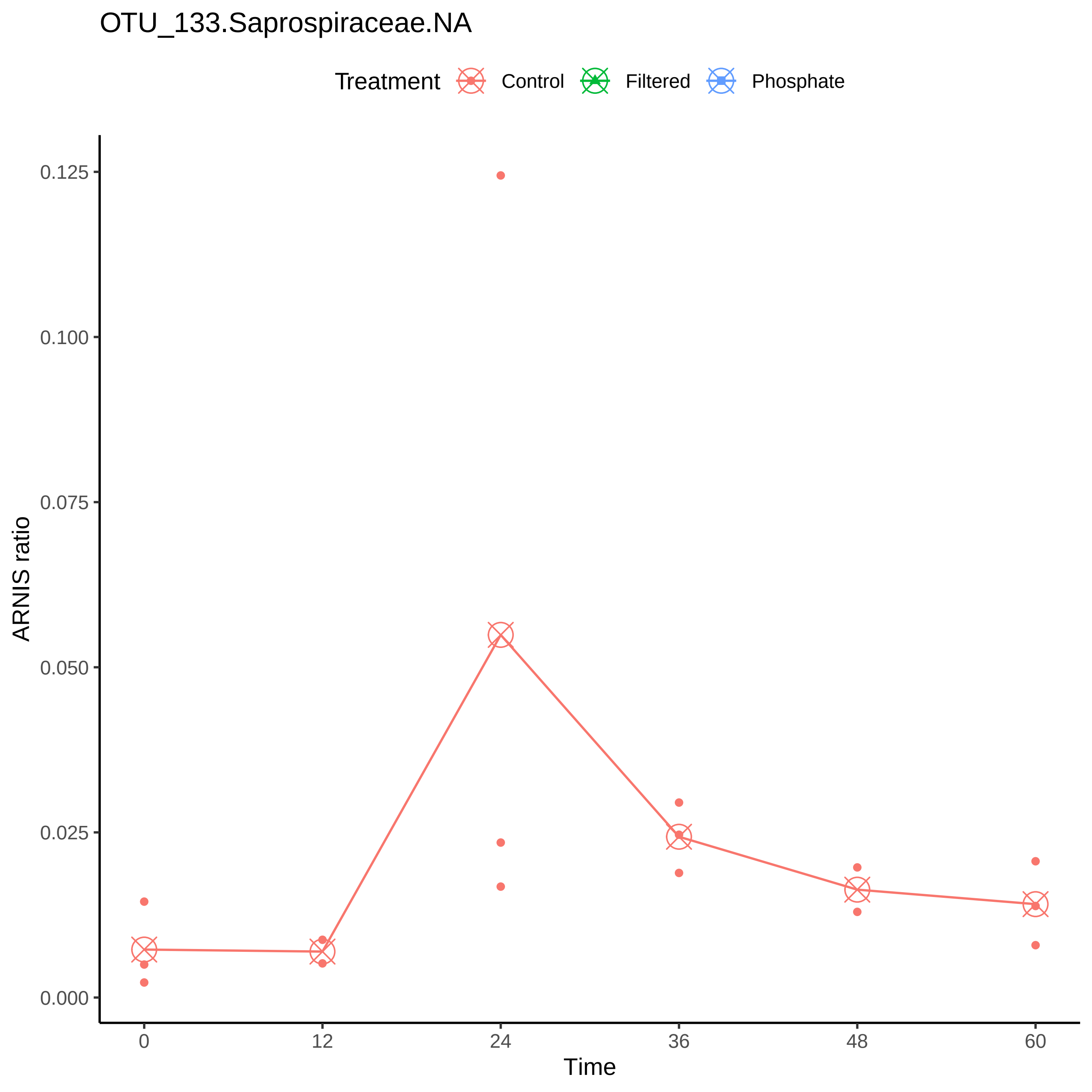
OTU_133.Saprospiraceae.NA
Treatment
Control
Filtered
Phosphate
ARNIS ratio
0.125
0.100
0.075
0.050
0.025
0.000
0
12
24
36
48
60
Time

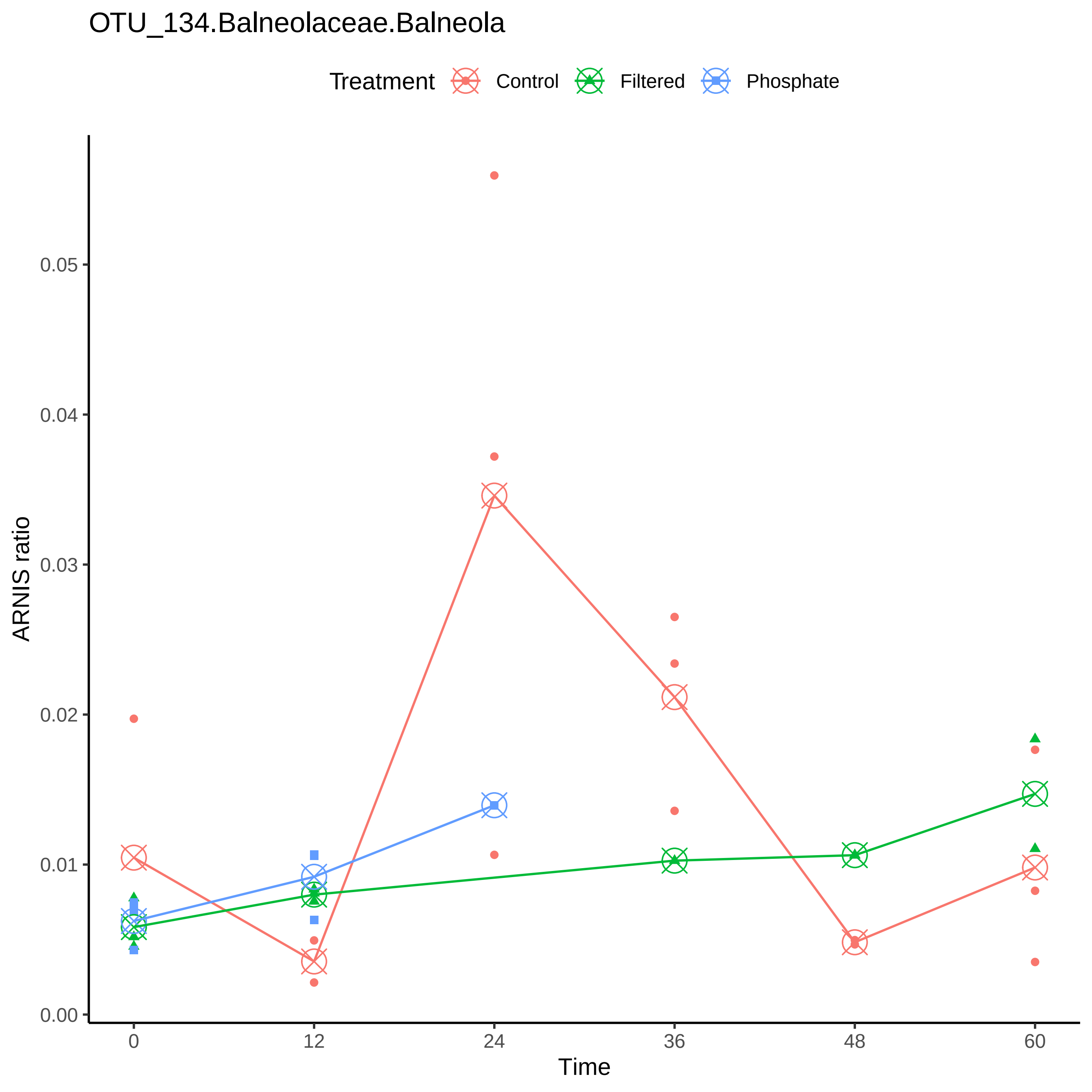
OTU_134.Balneolaceae.Balneola
Treatment
Control
Filtered
Phosphate
ARNIS ratio
0.00
0.01
0.02
0.03
0.04
0.05
0
12
24
36
48
60
Time

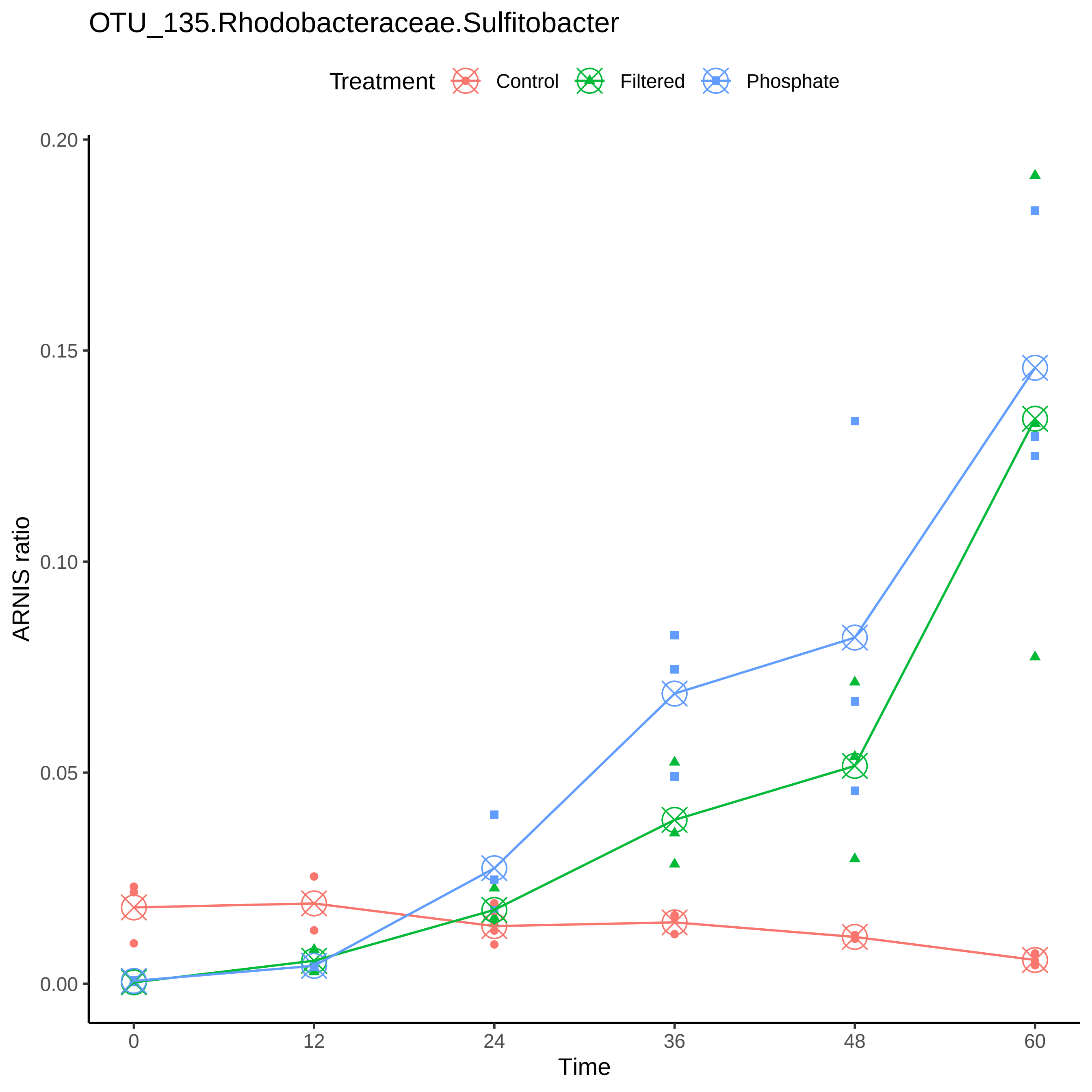
OTU_135.Rhodobacteraceae.Sulfitobacter
Treatment
Control
Filtered
Phosphate
0.20
0.15
0.10
0.05
0.00
ARNIS ratio
0
12
24
36
48
60
Time

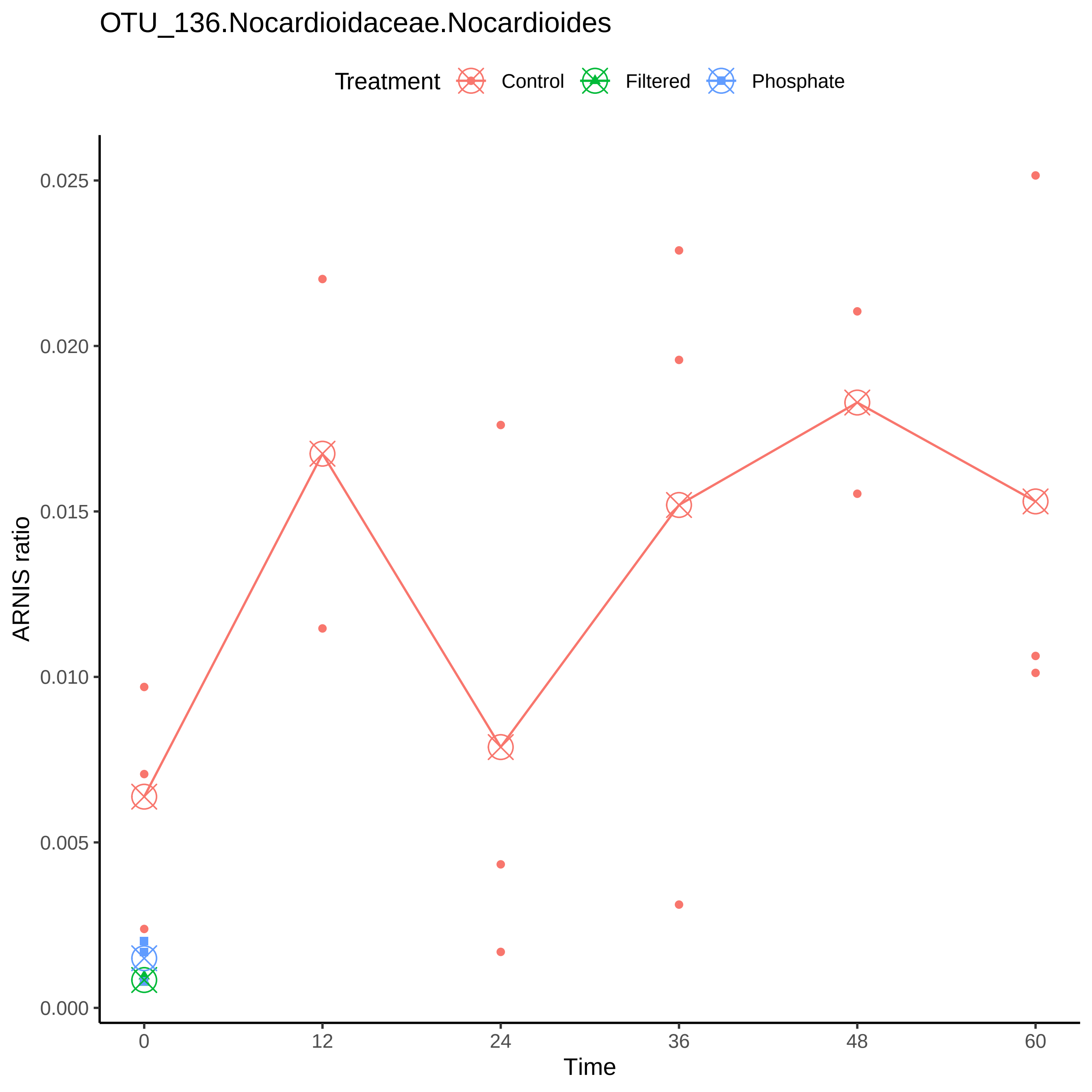

OTU_136.Nocardioidaceae.Nocardioides
Treatment
Control
Filtered
Phosphate
ARNIS ratio
0.025
0.020
0.015
0.010
0.005
0.000
0
12
24
36
48
60
Time

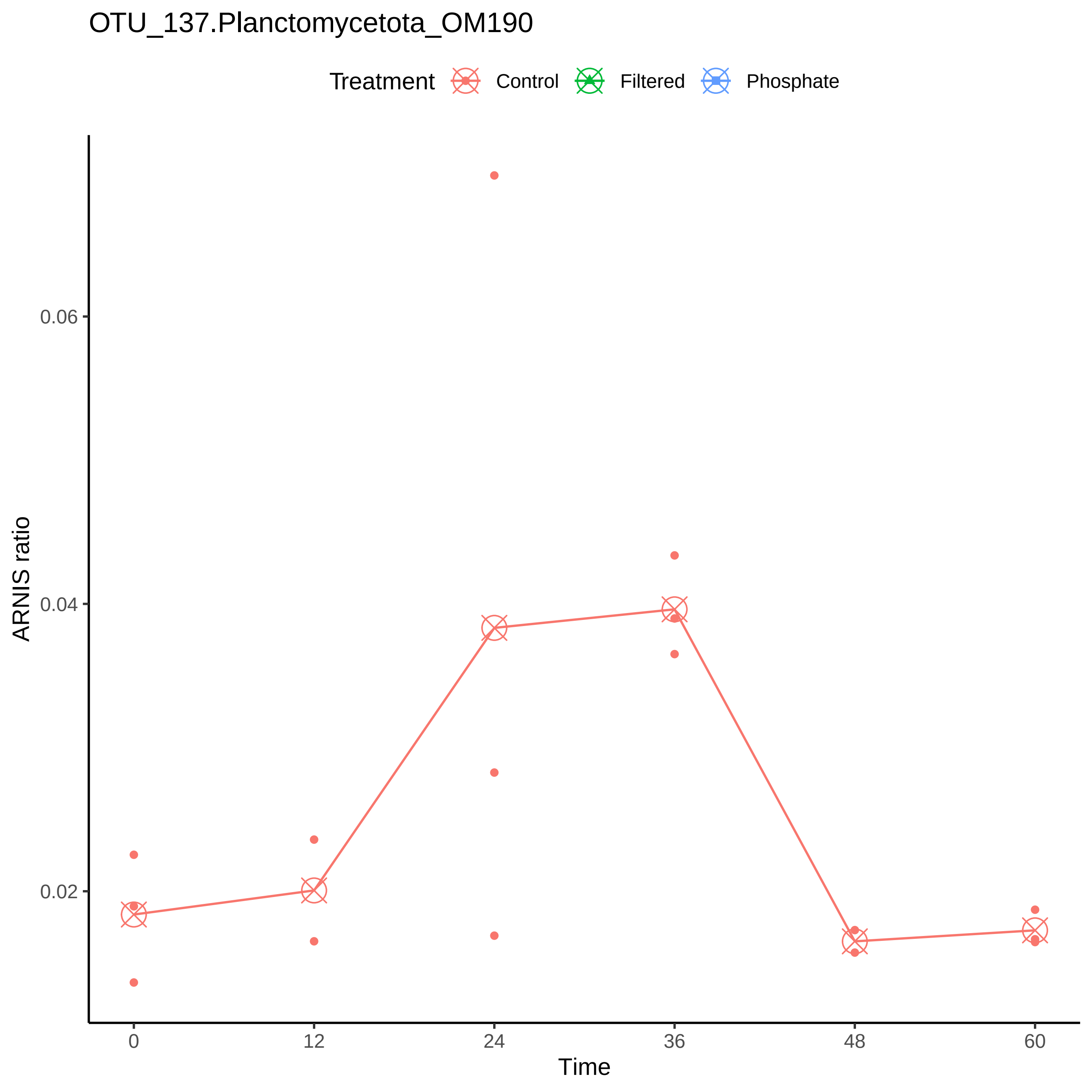

OTU_137.Planctomycetota_OM190
Treatment
Control
Filtered
Phosphate
ARNIS ratio
0.06
0.04
0.02
0
12
24
36
48
60
Time

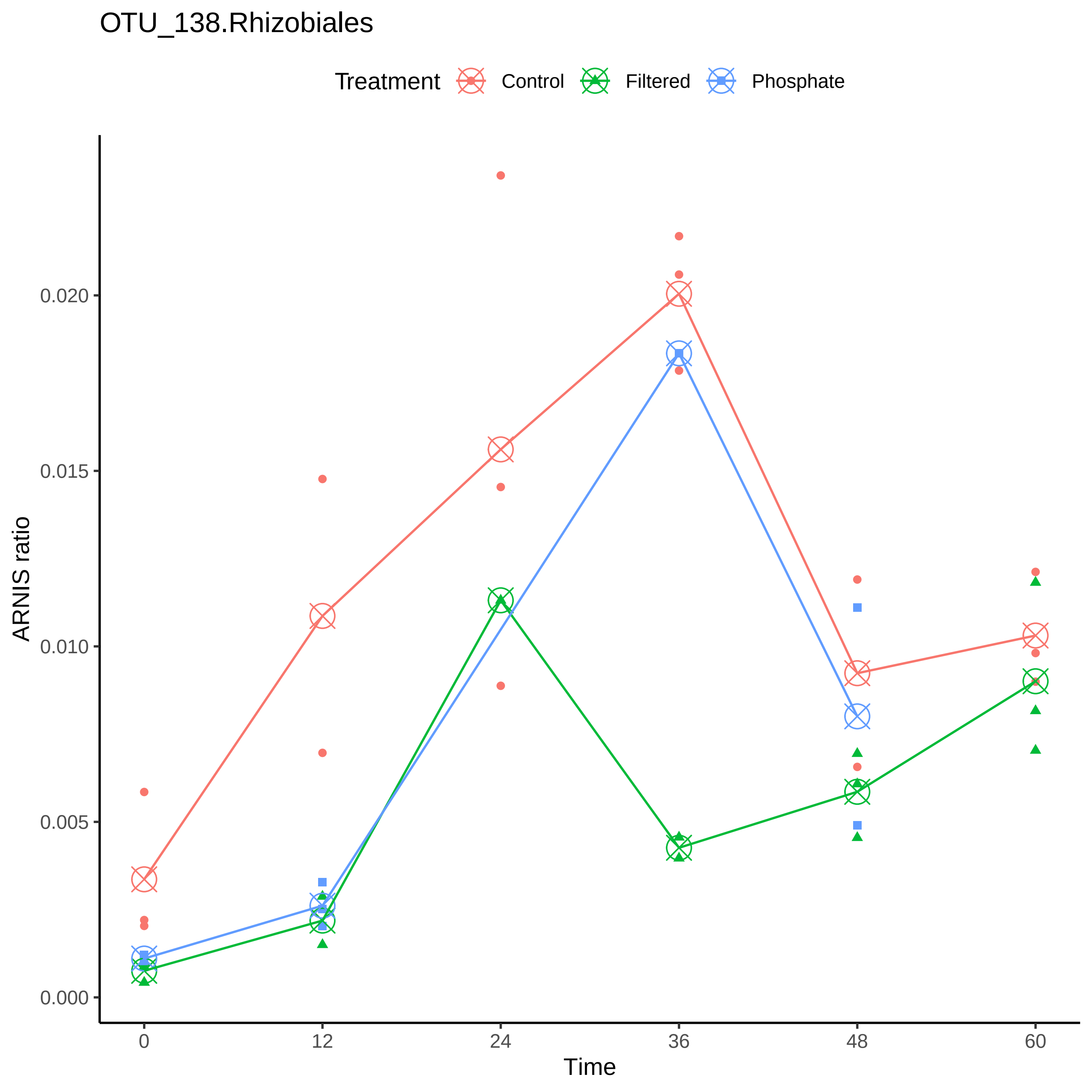
OTU_138.Rhizobiales
Treatment
Control
Filtered
Phosphate
ARNIS ratio
0.000
0.005
0.010
0.015
0.020
0
12
24
36
48
60
Time

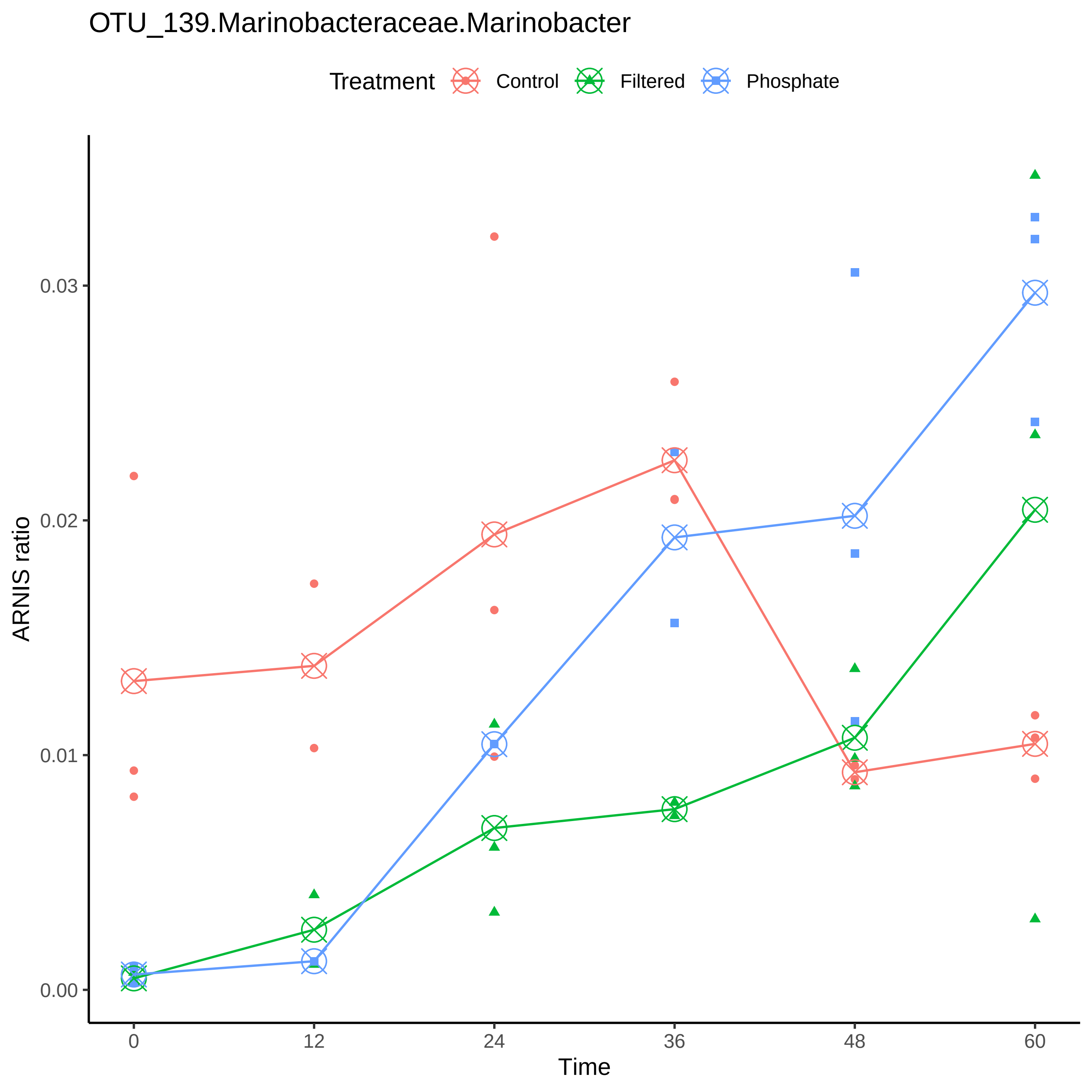
OTU_139.Marinobacteraceae.Marinobacter
Treatment
Control
Filtered
Phosphate
ARNIS ratio
0.00
0.01
0.02
0.03
Time
0
12
24
36
48
60

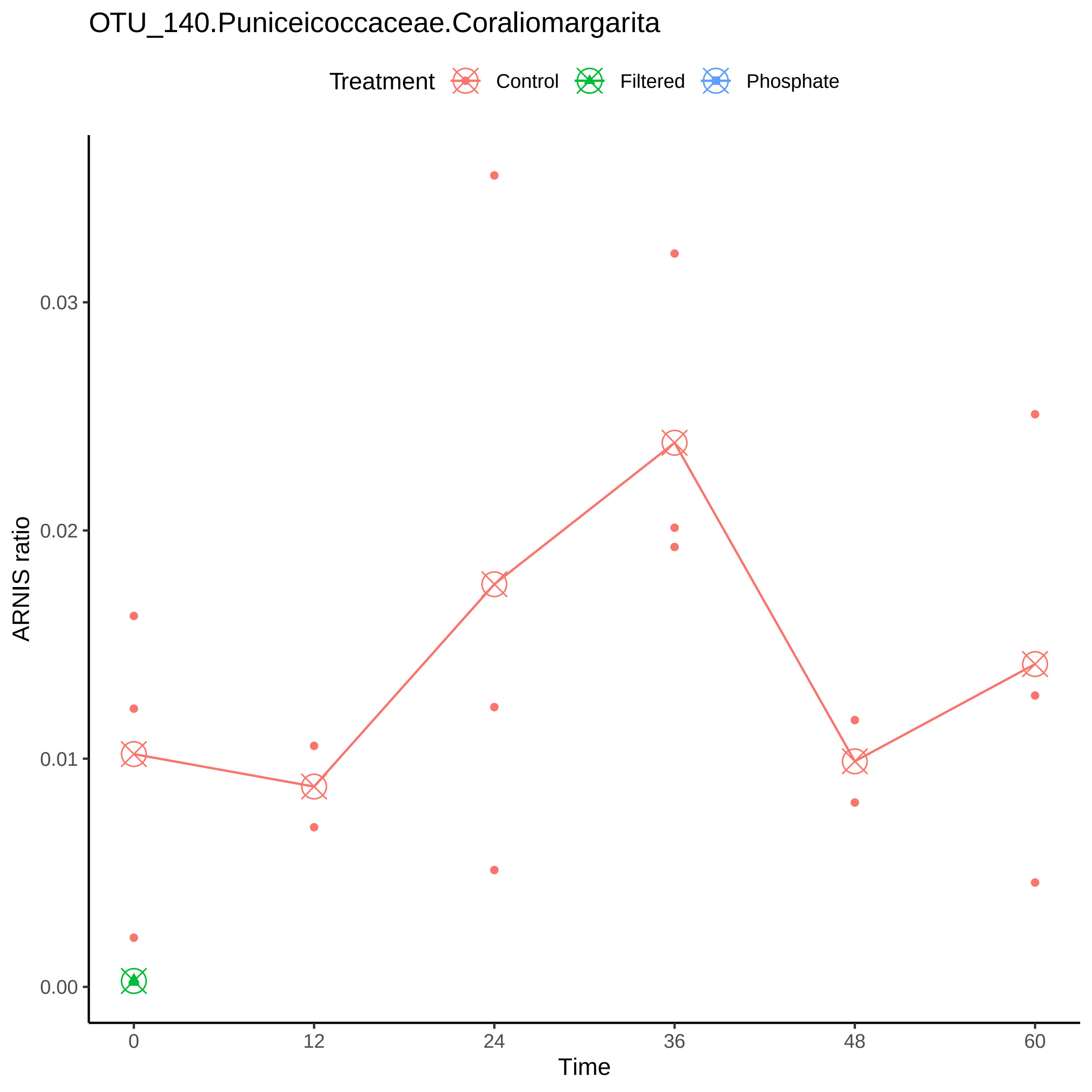

OTU_140.Puniceicoccaceae.Coraliomargarita
Treatment
Control
Filtered
Phosphate
ARNIS ratio
0.00
0.01
0.02
0.03
Time
0
12
24
36
48
60

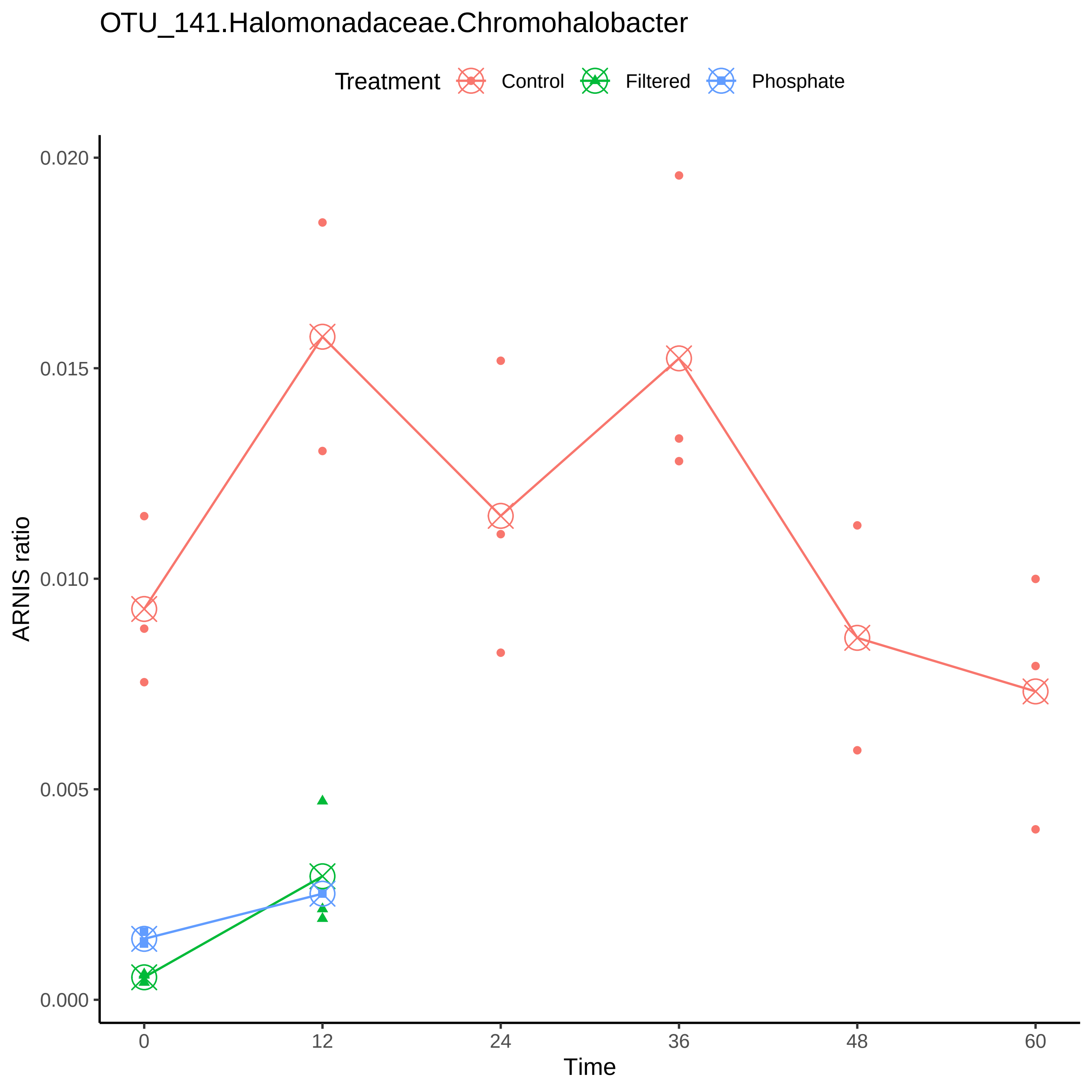

OTU_141.Halomonadaceae.Chromohalobacter
Treatment
Control
Filtered
Phosphate
ARNIS ratio
0.020
0.015
0.010
0.005
0.000
0
12
24
36
48
60
Time

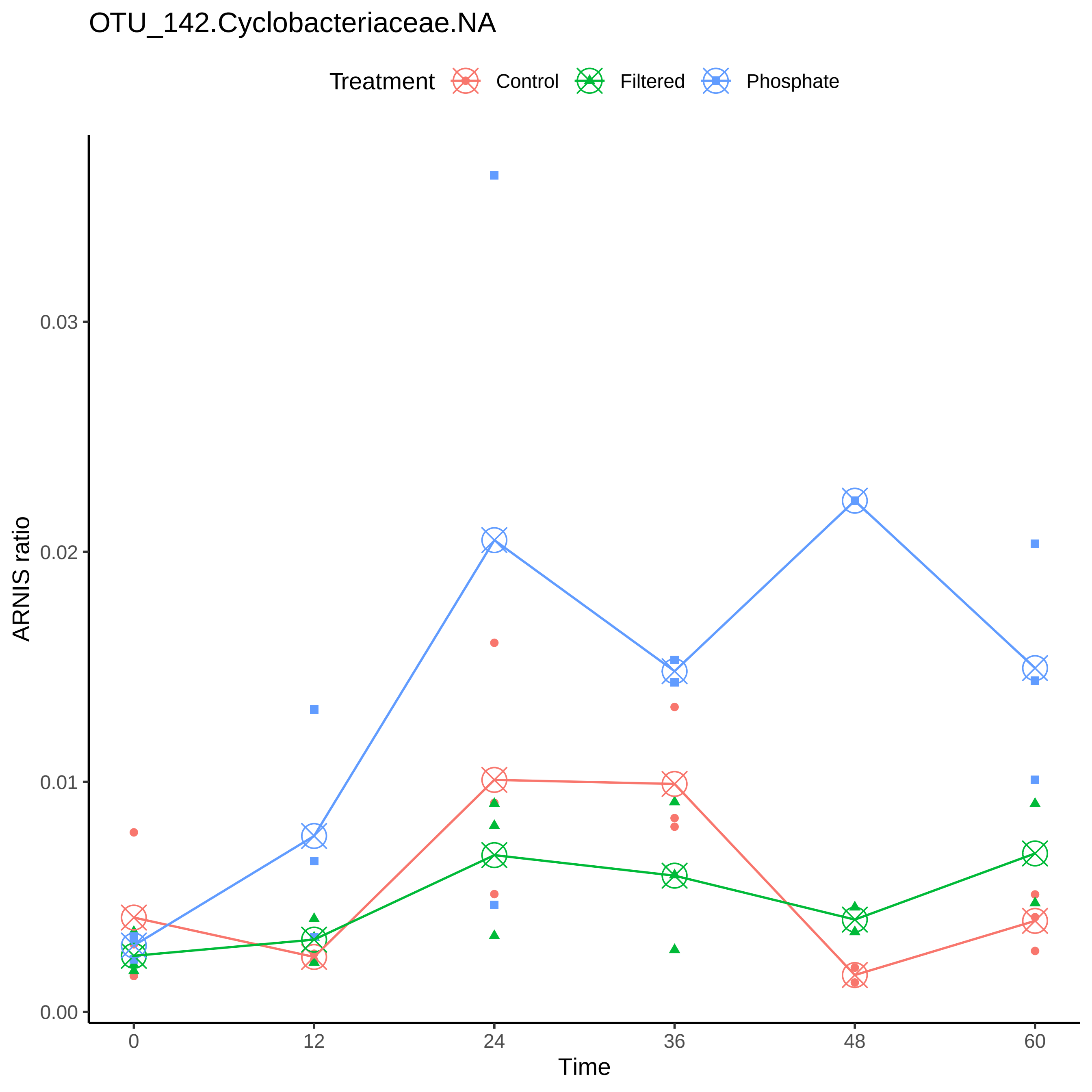

OTU_142.Cyclobacteriaceae.NA
Treatment
Control
Filtered
Phosphate
ARNIS ratio
0.00
0.01
0.02
0.03
0
12
24
36
48
60
Time

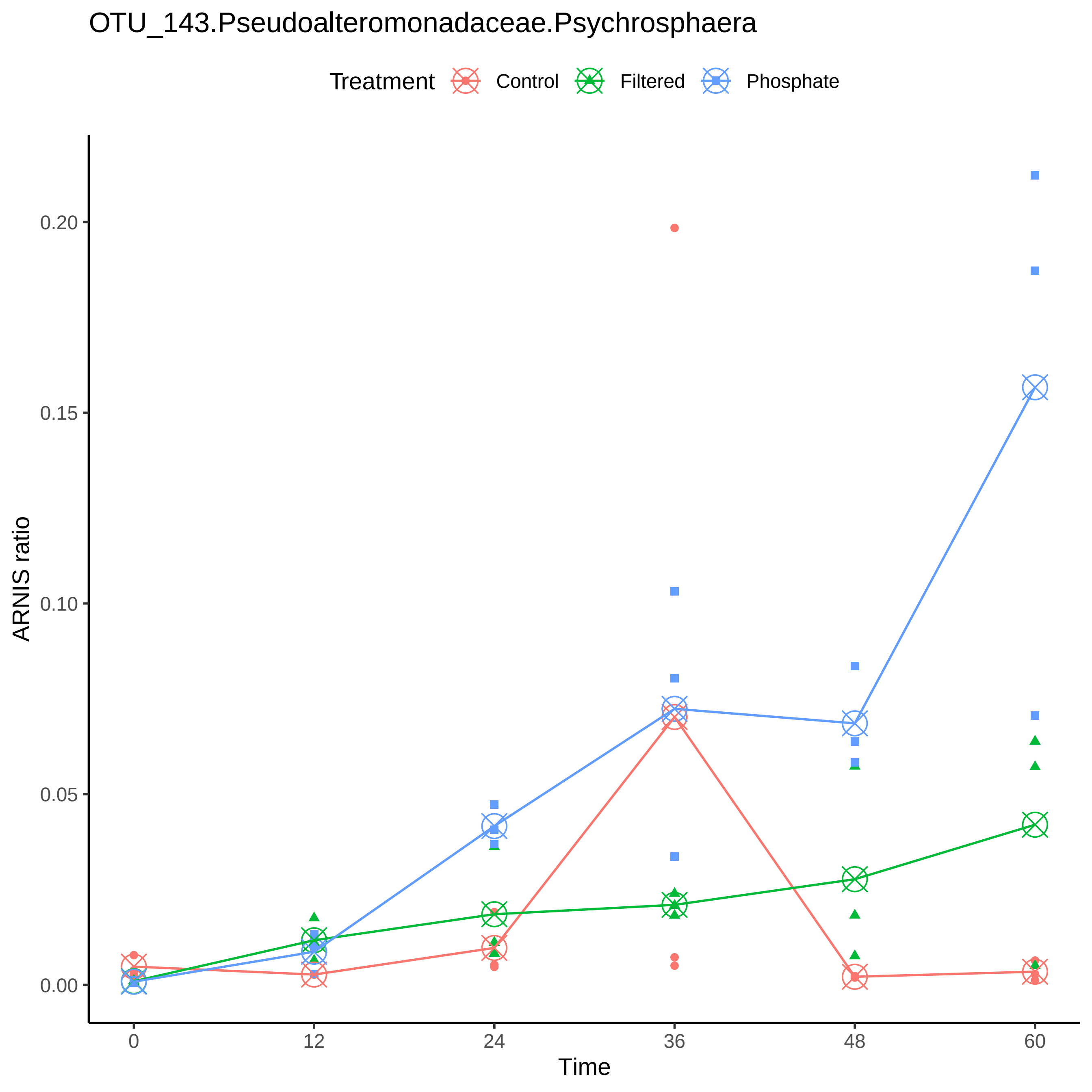

OTU_143.Pseudoalteromonadaceae.Psychrosphaera
Treatment
Control
Filtered
Phosphate
ARNIS ratio
0.00
0.05
0.10
0.15
0.20
Time
0
12
24
36
48
60

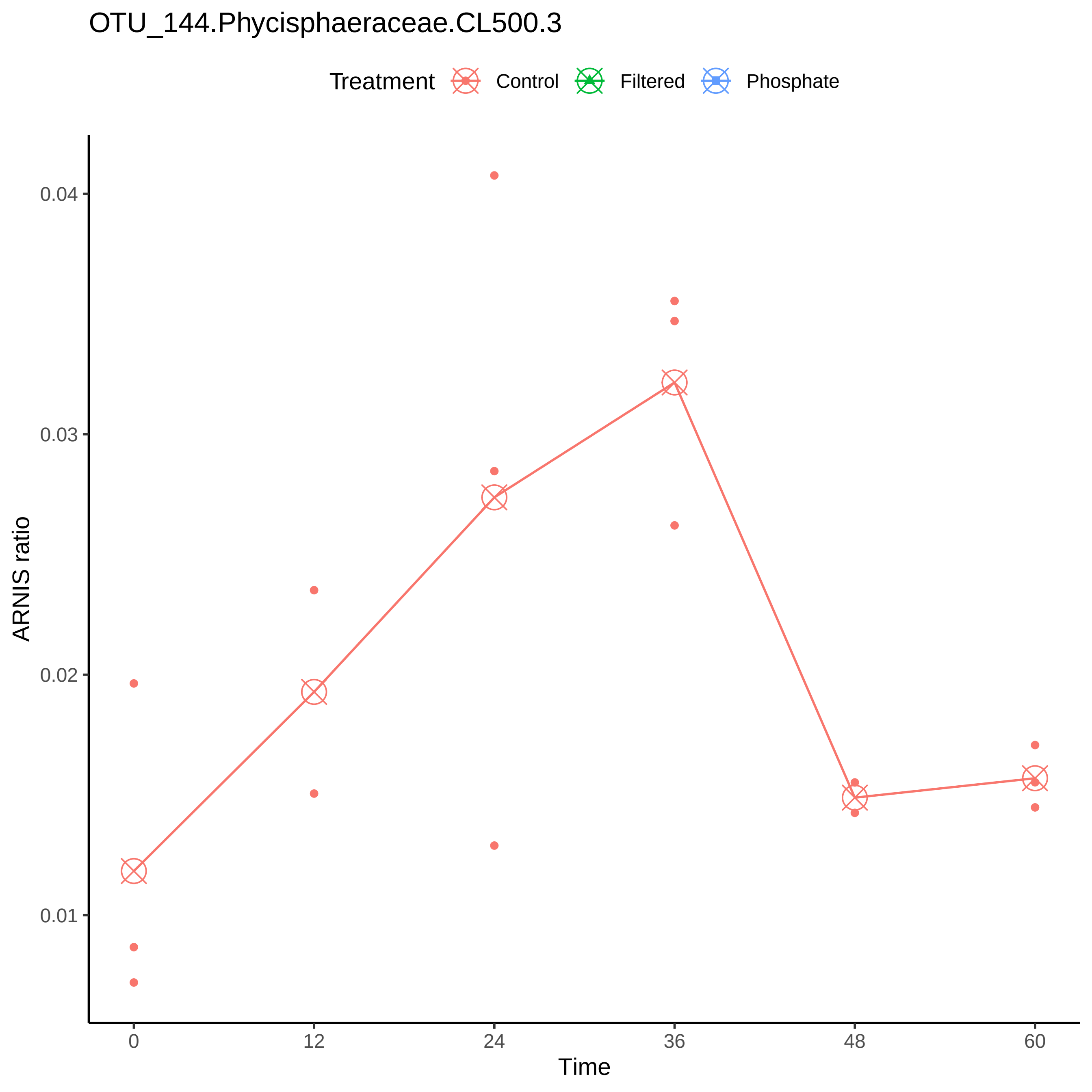
OTU_144.Phycisphaeraceae.CL500.3
Treatment
Control
Filtered
Phosphate
ARNIS ratio
0.04
0.03
0.02
0.01
0
12
24
36
48
60
Time

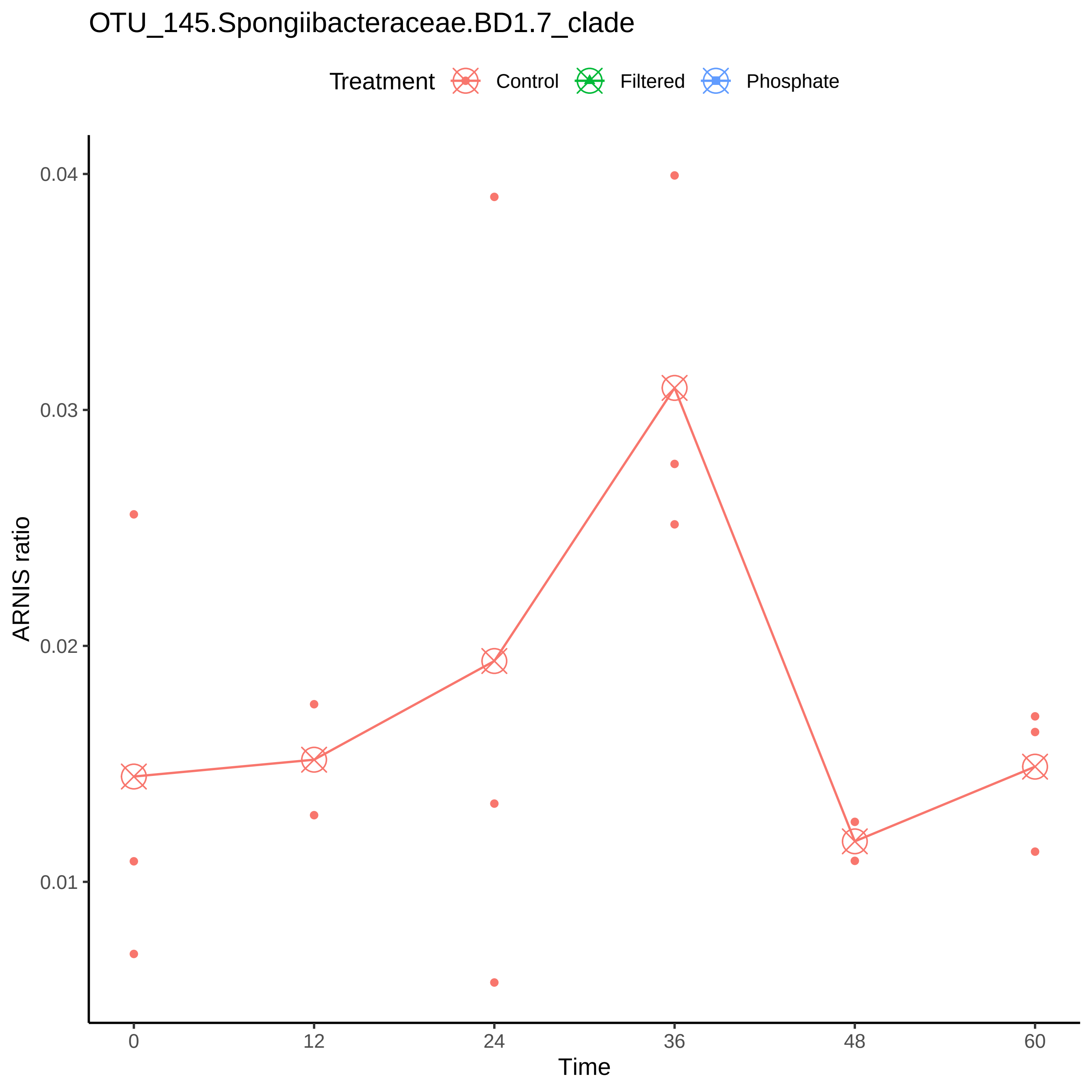
OTU_145.Spongiibacteraceae.BD1.7_clade
Treatment
Control
Filtered
Phosphate
ARNIS ratio
0.04
0.03
0.02
0.01
0
12
24
36
48
60
Time

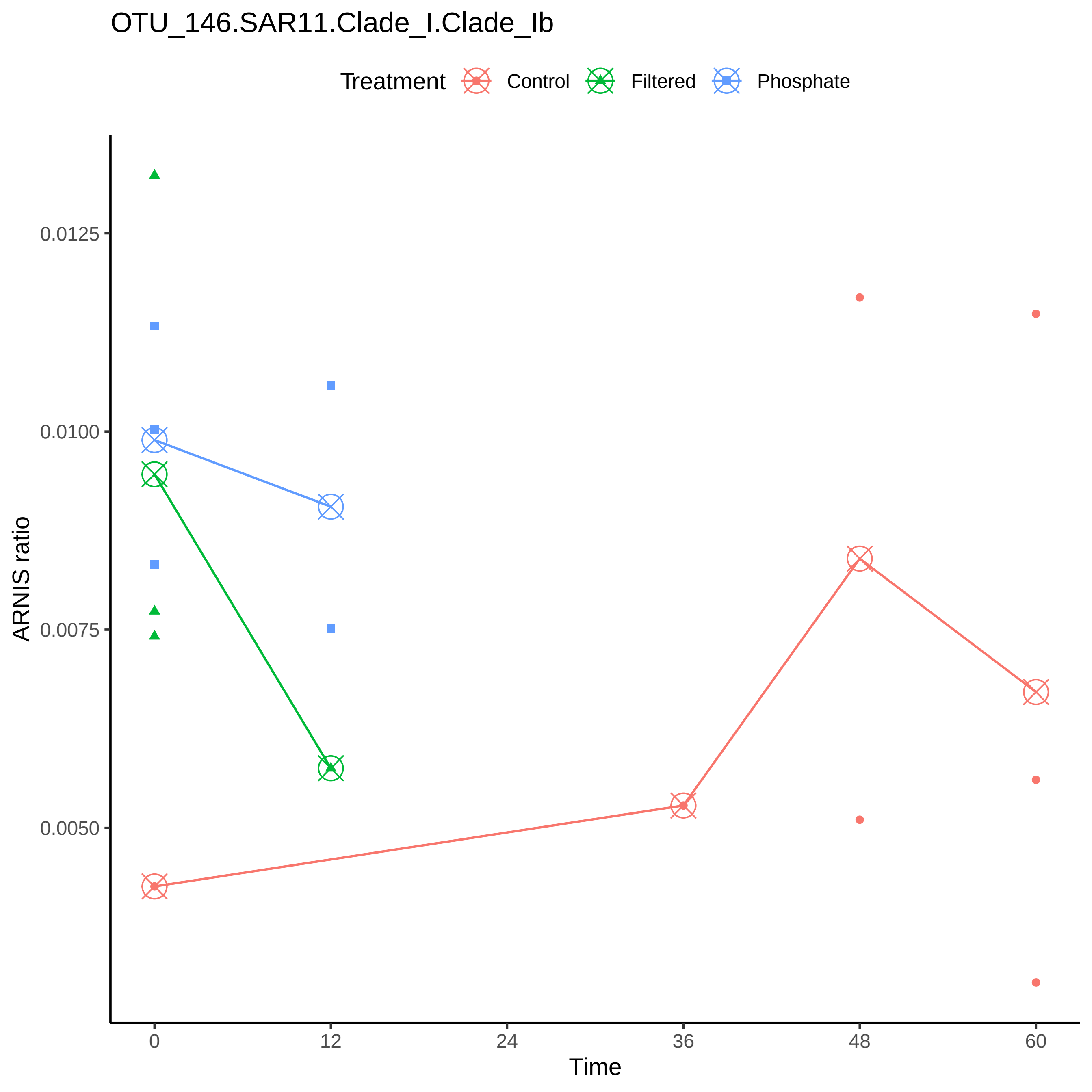
OTU_146.SAR11.Clade_I.Clade_Ib
Treatment
Control
Filtered
Phosphate
0.0125
0.0100
0.0075
0.0050
ARNIS ratio
0
12
24
36
48
60
Time

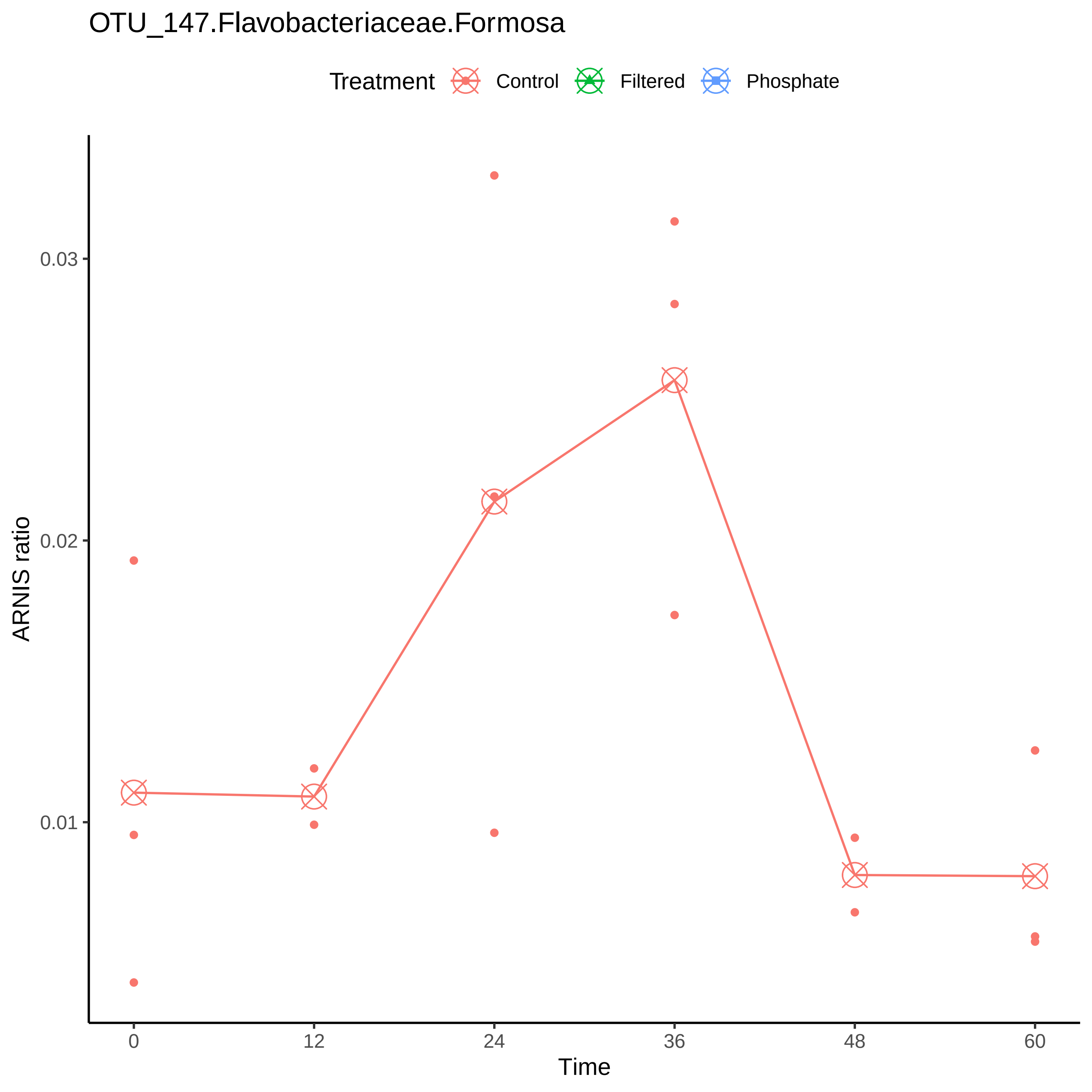
OTU_147.Flavobacteriaceae.Formosa
Treatment
Control
Filtered
Phosphate
ARNIS ratio
0.03
0.02
0.01
Time
0
12
24
36
48
60

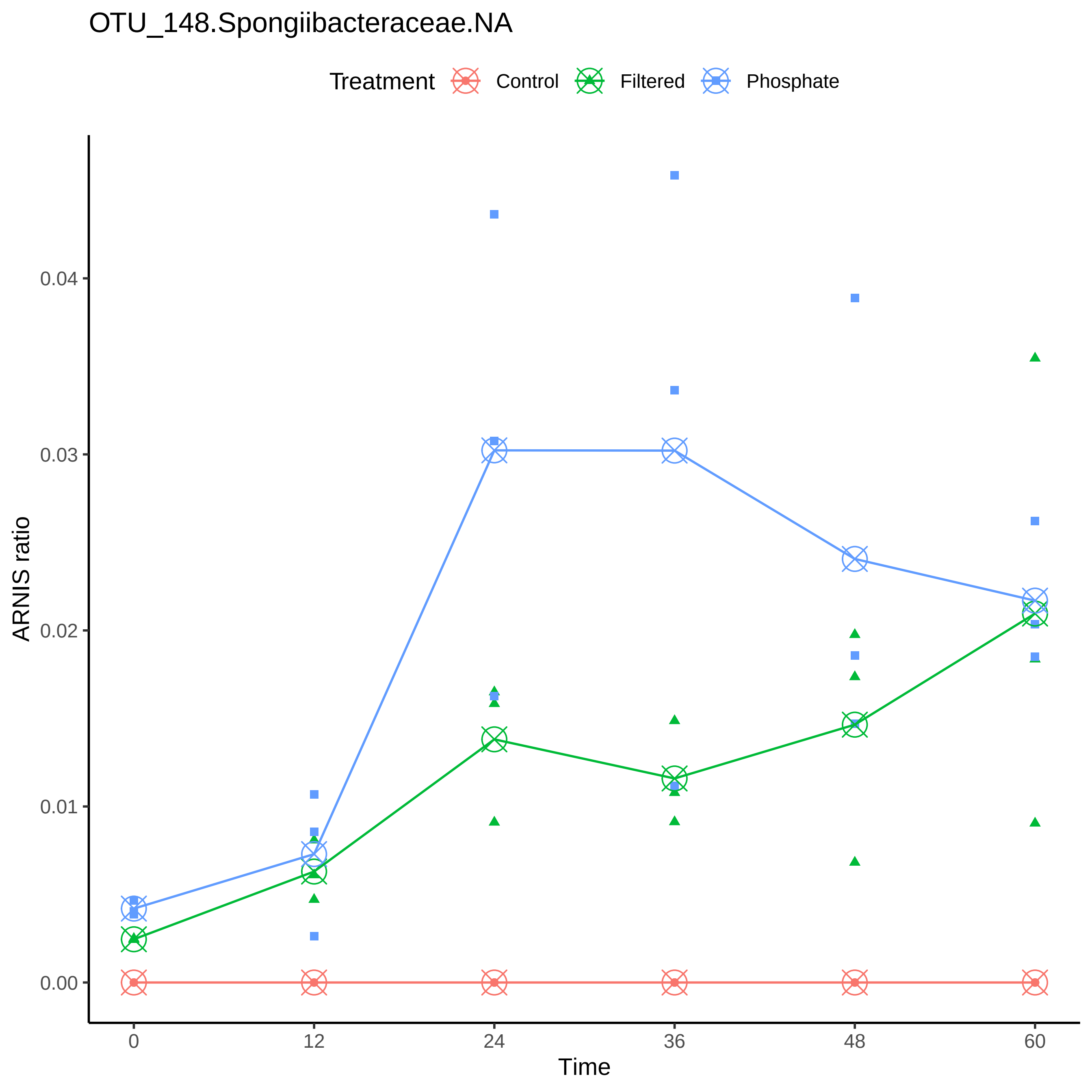
OTU_148.Spongiibacteraceae.NA
Treatment
Control
Filtered
Phosphate
ARNIS ratio
0.00
0.01
0.02
0.03
0.04
Time
0
12
24
36
48
60

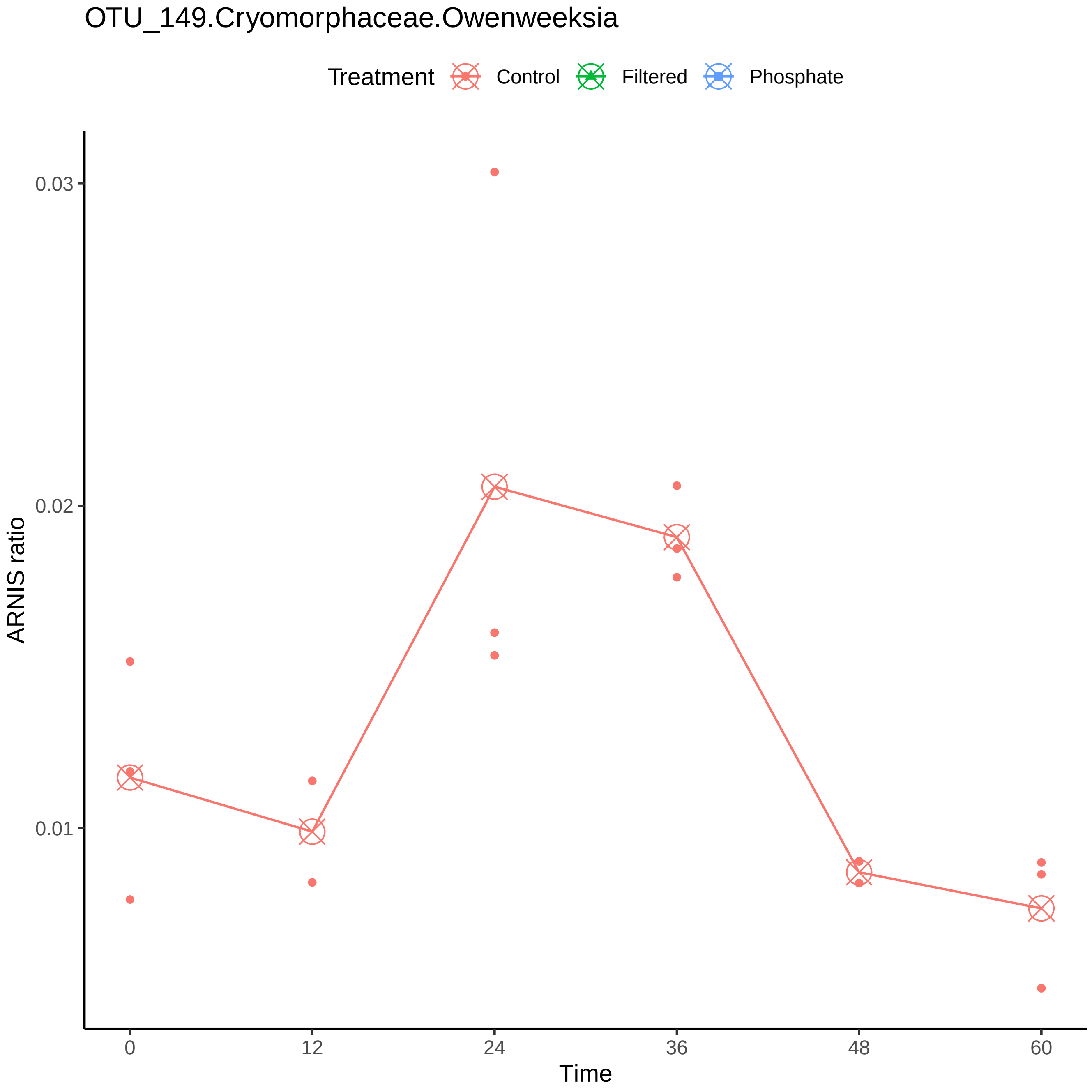
OTU_149.Cryomorphaceae.Owenweeksia
Treatment
Control
Filtered
Phosphate
ARNIS ratio
0.03
0.02
0.01
0
12
24
36
48
60
Time

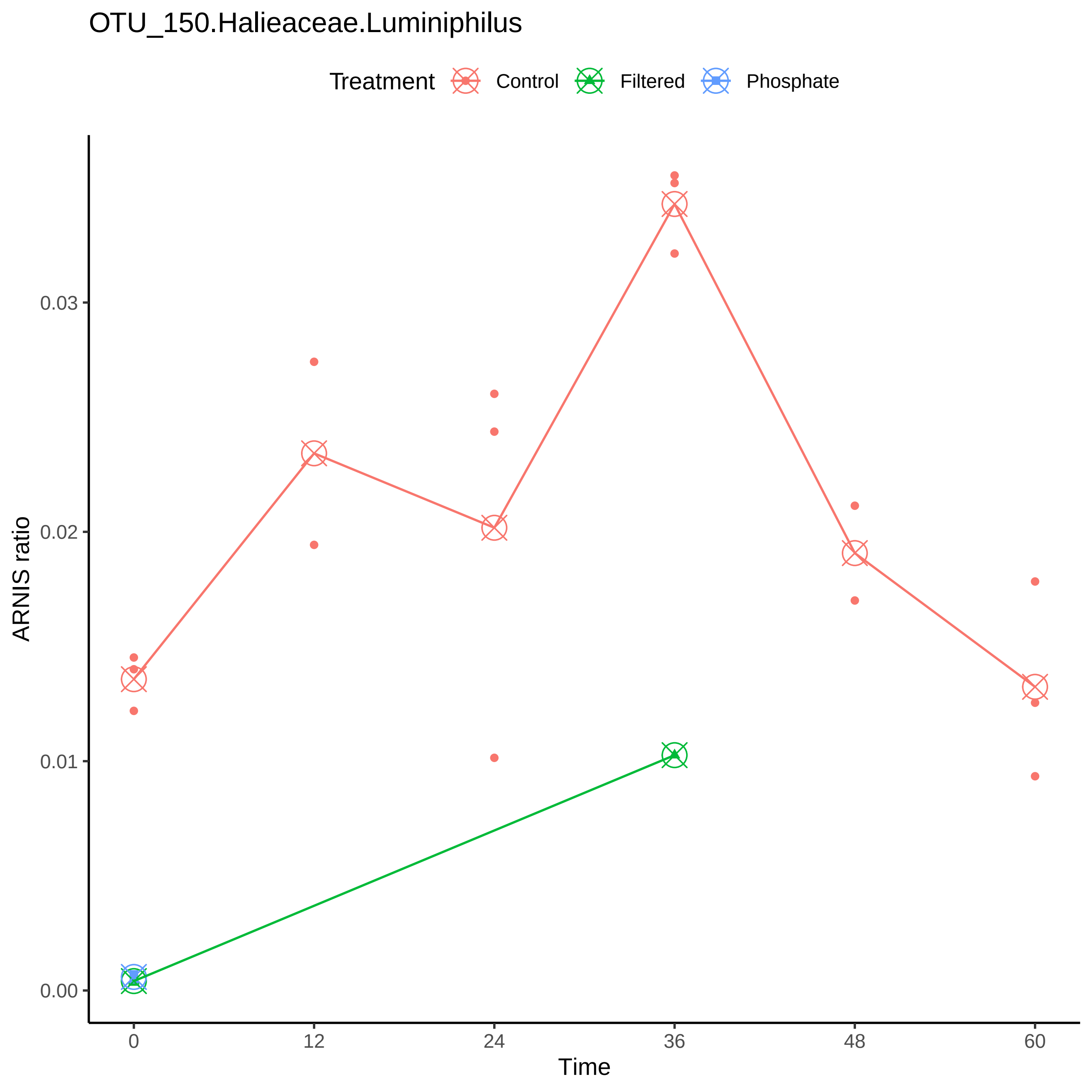

OTU_150.Halieaceae.Luminiphilus
Treatment
Control
Filtered
Phosphate
ARNIS ratio
0.00
0.01
0.02
0.03
0
12
24
36
48
60
Time

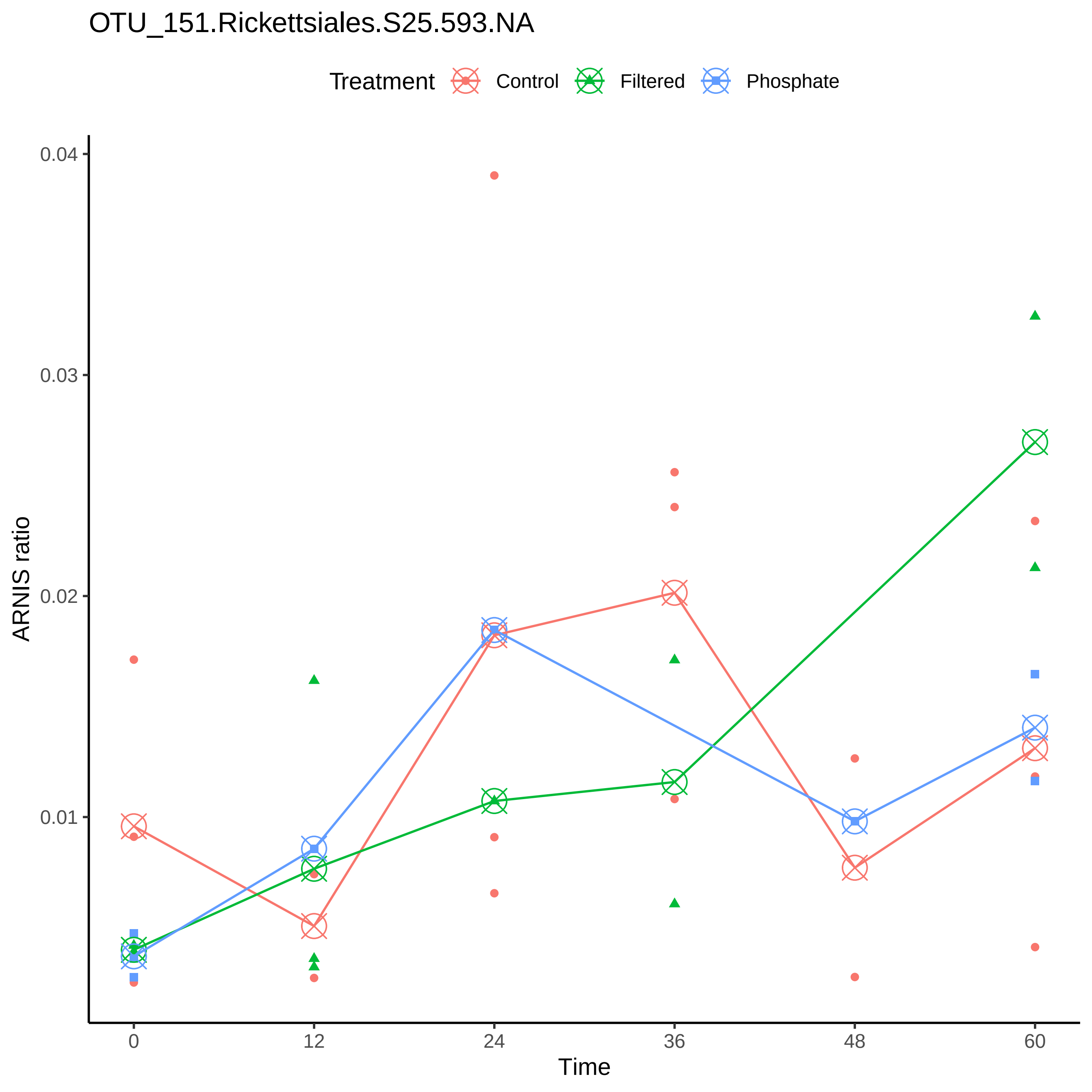

OTU_151.Rickettsiales.S25.593.NA
Treatment
Control
Filtered
Phosphate
ARNIS ratio
0.04
0.03
0.02
0.01
0
12
24
36
48
60
Time

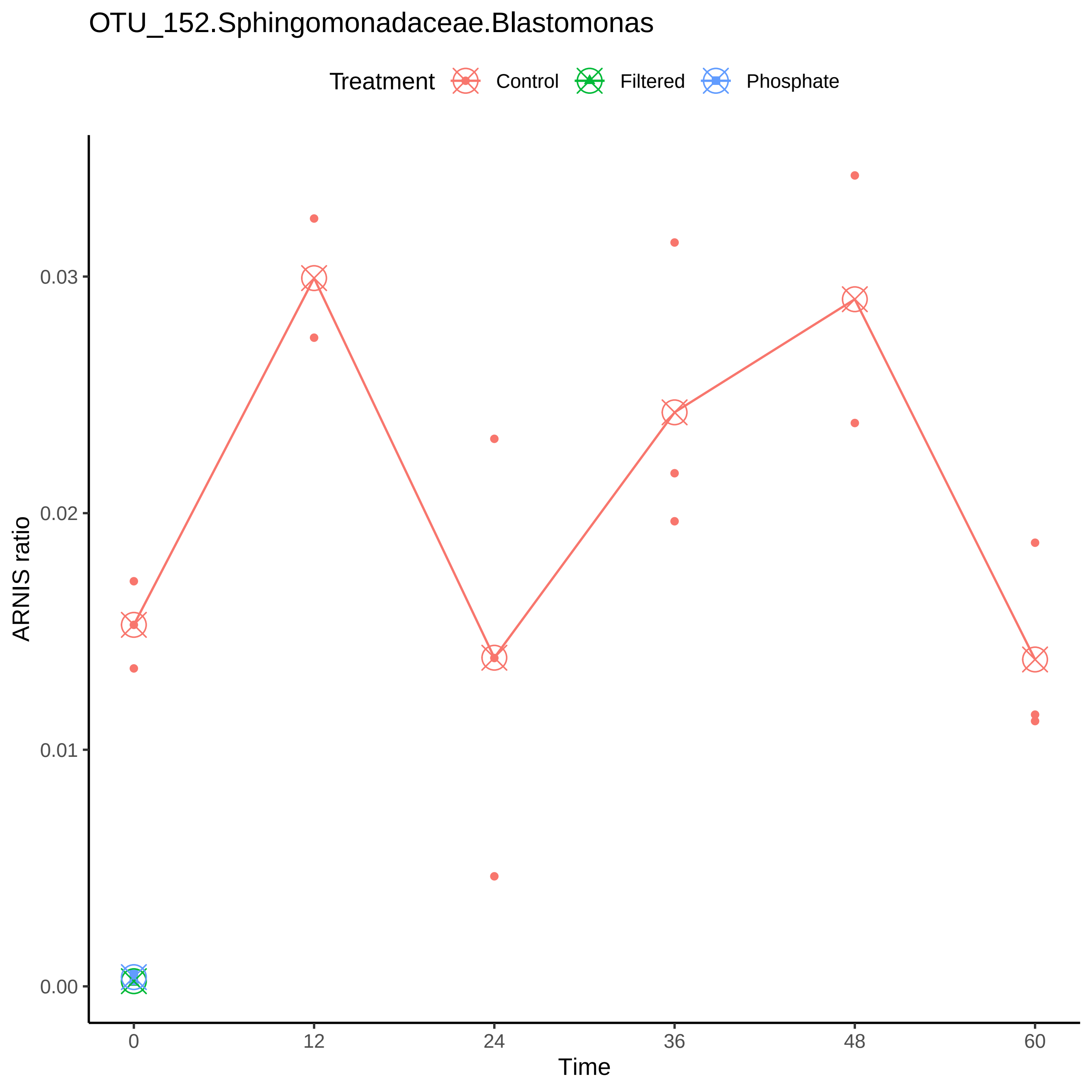

OTU_152.Sphingomonadaceae.Blastomonas
Treatment
Control
Filtered
Phosphate
ARNIS ratio
0.00
0.01
0.02
0.03
Time
0
12
24
36
48
60

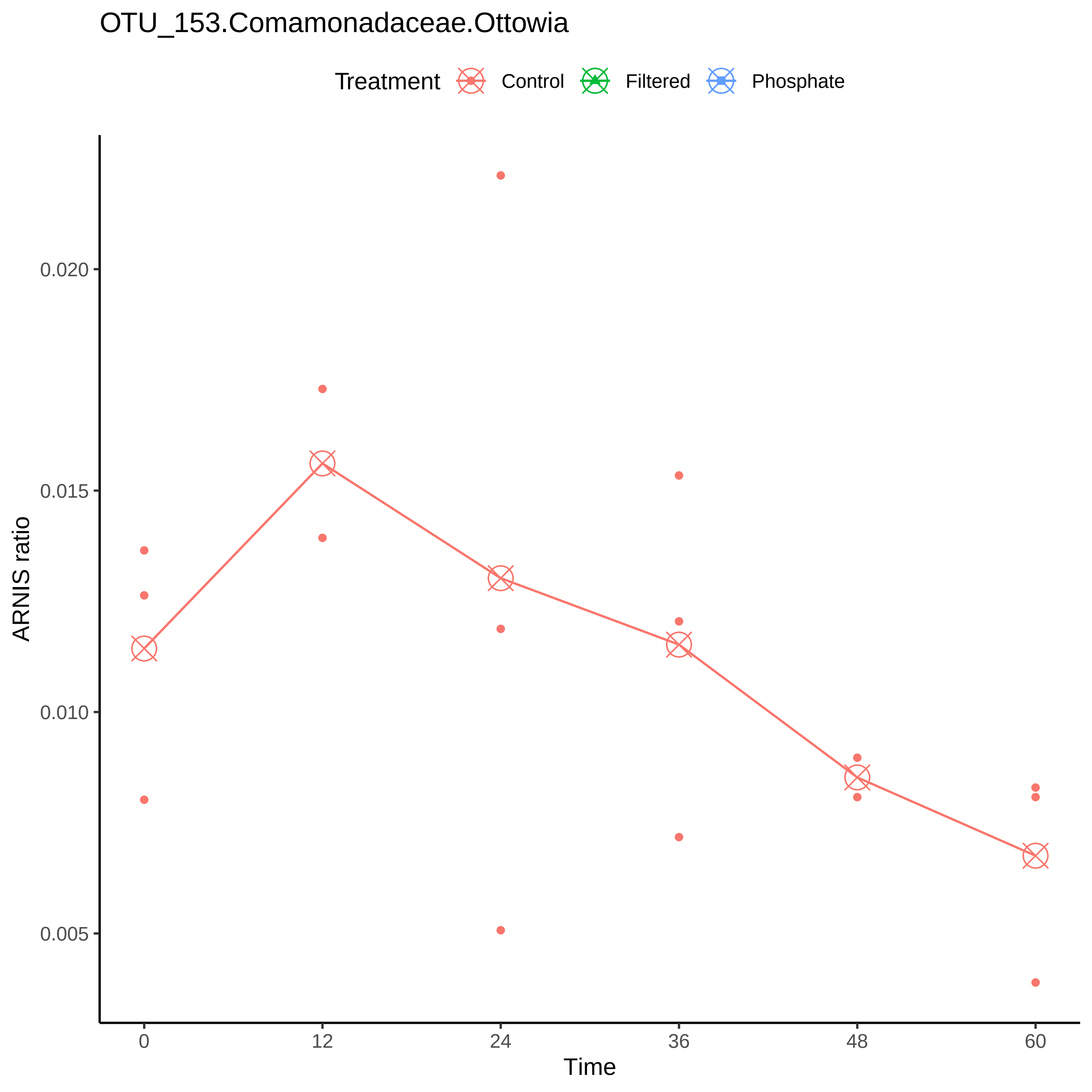

OTU_153.Comamonadaceae.Ottowia
Treatment
Control
Filtered
Phosphate
ARNIS ratio
0.020
0.015
0.010
0.005
Time
0
12
24
36
48
60

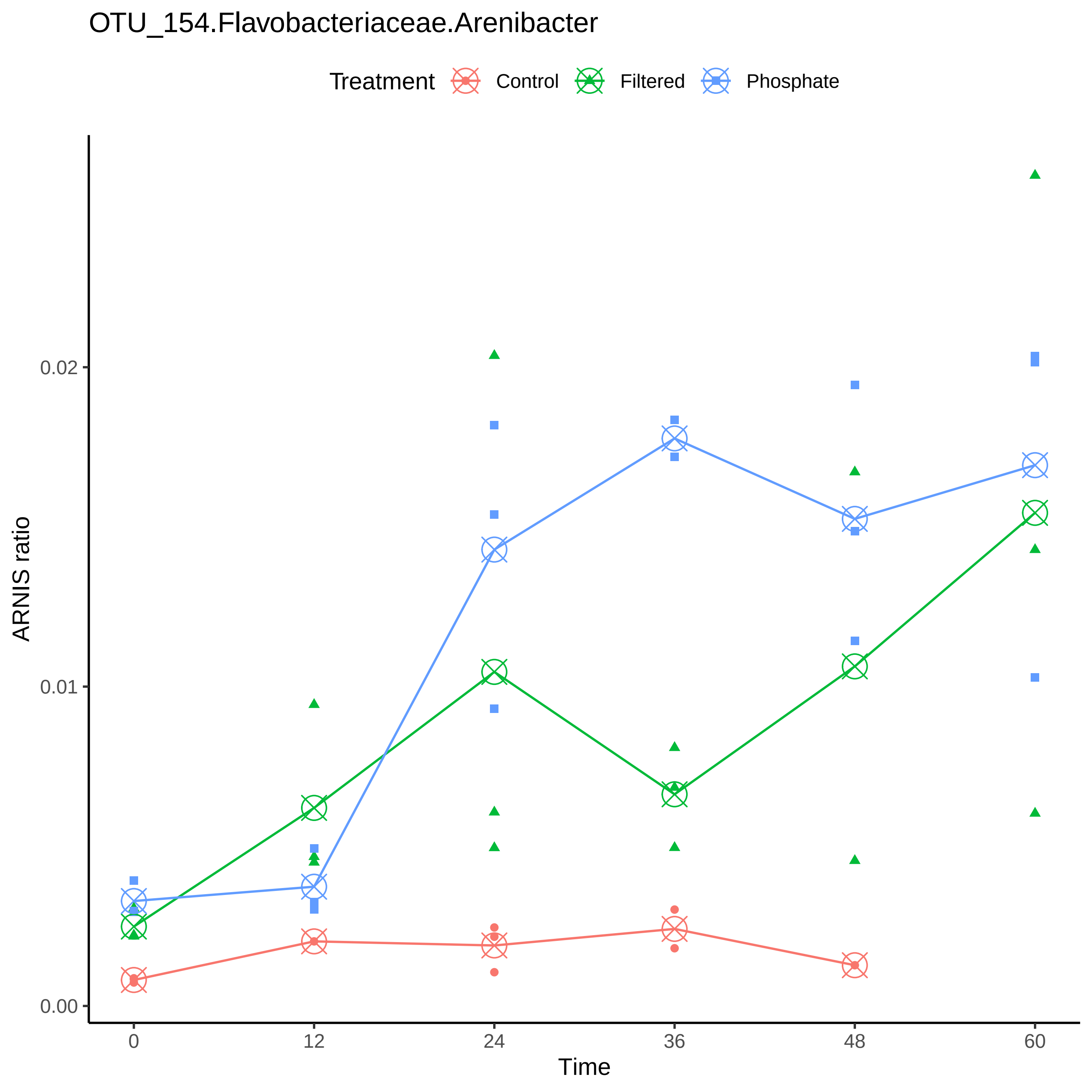

OTU_154.Flavobacteriaceae.Arenibacter
Treatment
Control
Filtered
Phosphate
ARNIS ratio
0.00
0.01
0.02
0
12
24
36
48
60
Time

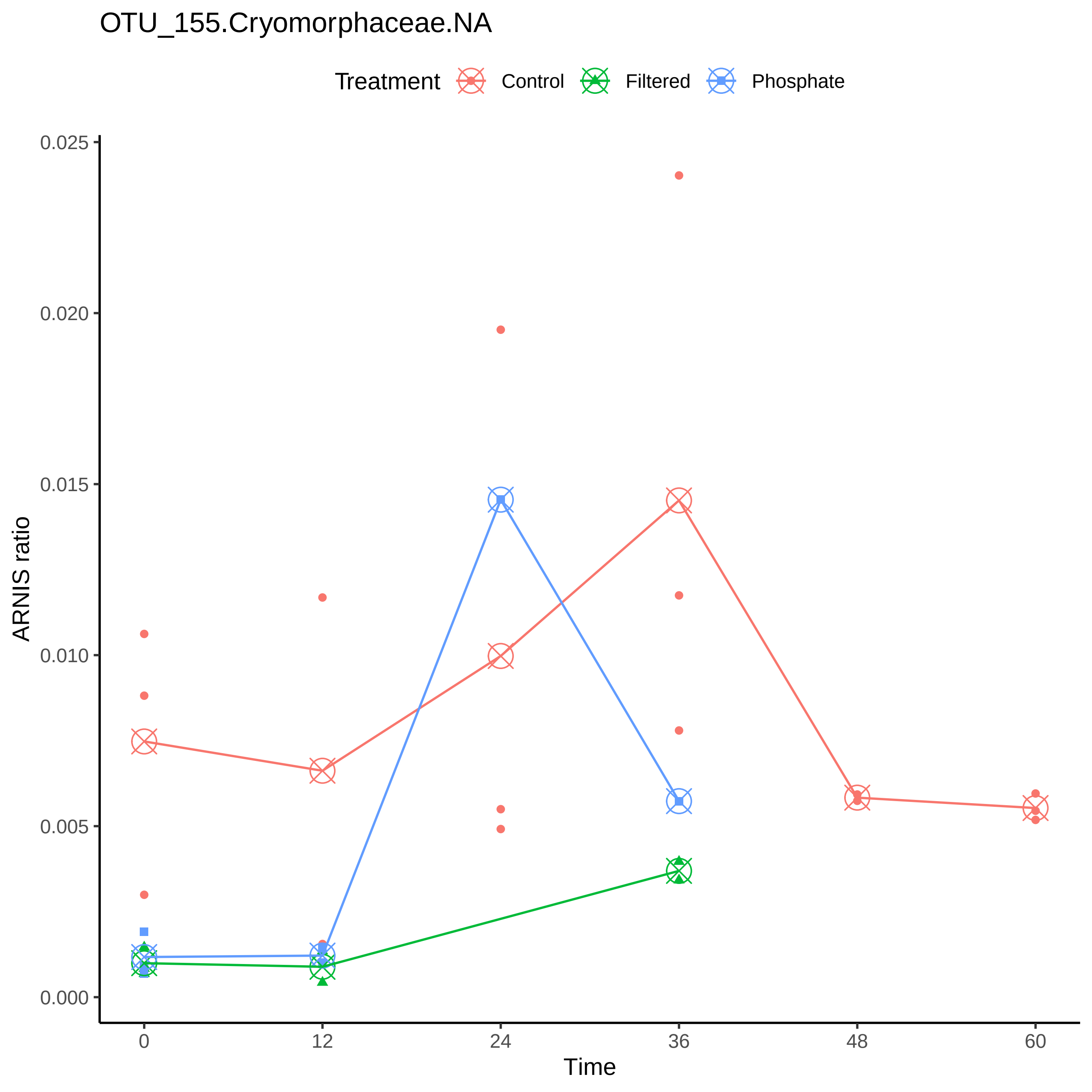
OTU_155.Cryomorphaceae.NA
Treatment
Control
Filtered
Phosphate
ARNIS ratio
0.025
0.020
0.015
0.010
0.005
0.000
0
12
24
36
48
60
Time

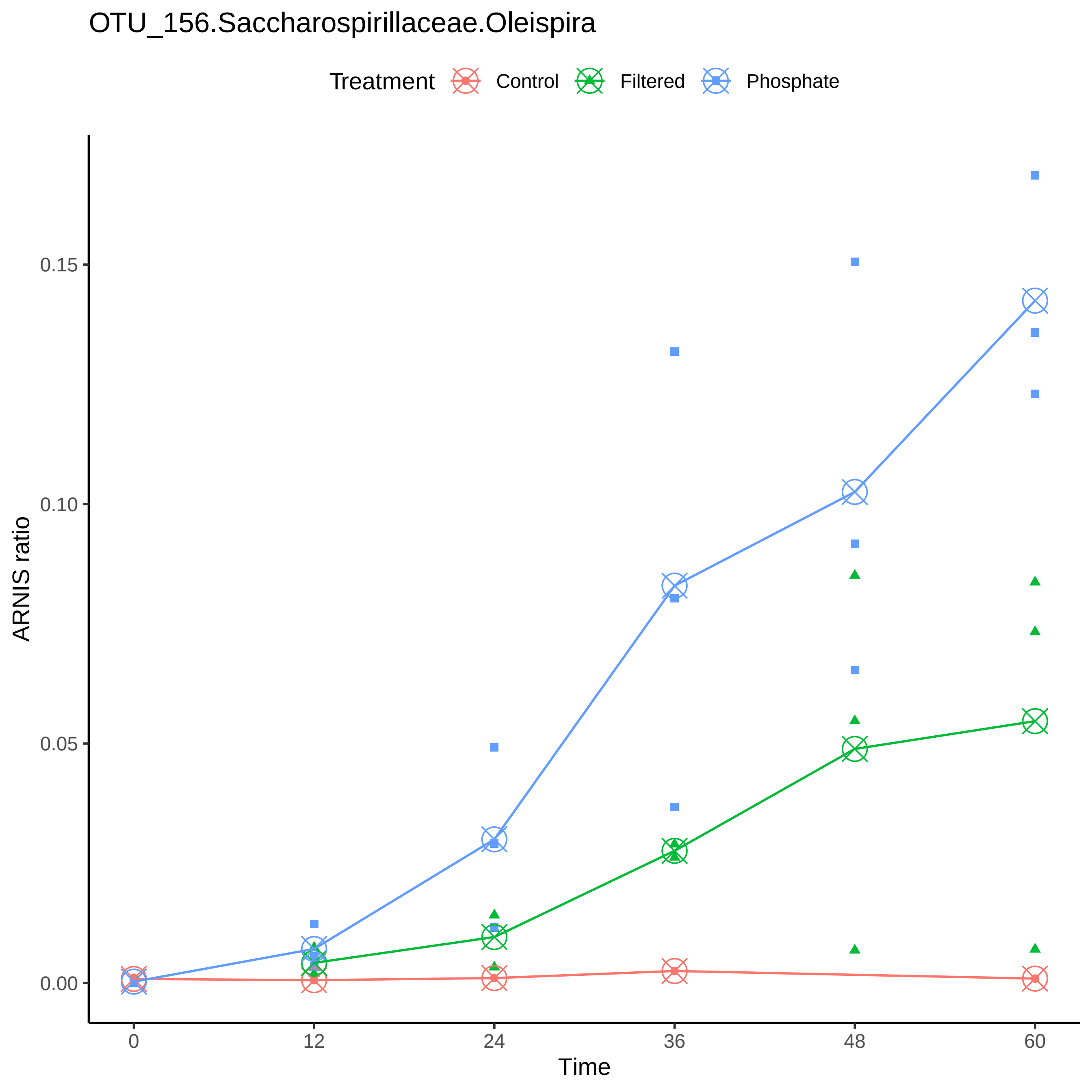
OTU_156.Saccharospirillaceae.Oleispira
Treatment
Control
Filtered
Phosphate
ARNIS ratio
0.00
0.05
0.10
0.15
0
12
24
36
48
60
Time

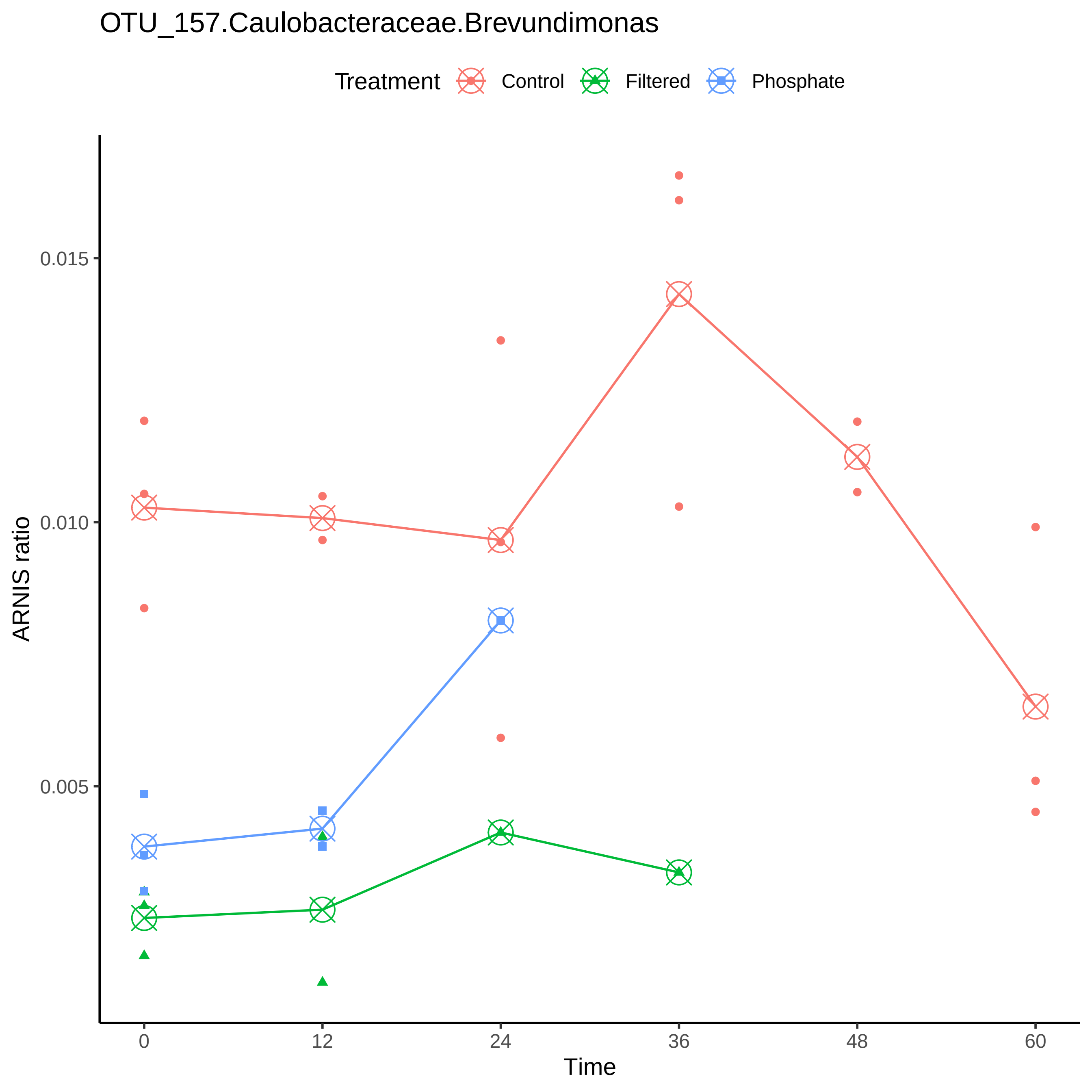

OTU_157.Caulobacteraceae.Brevundimonas
Treatment
Control
Filtered
Phosphate
ARNIS ratio
0.015
0.010
0.005
Time
0
12
24
36
48
60

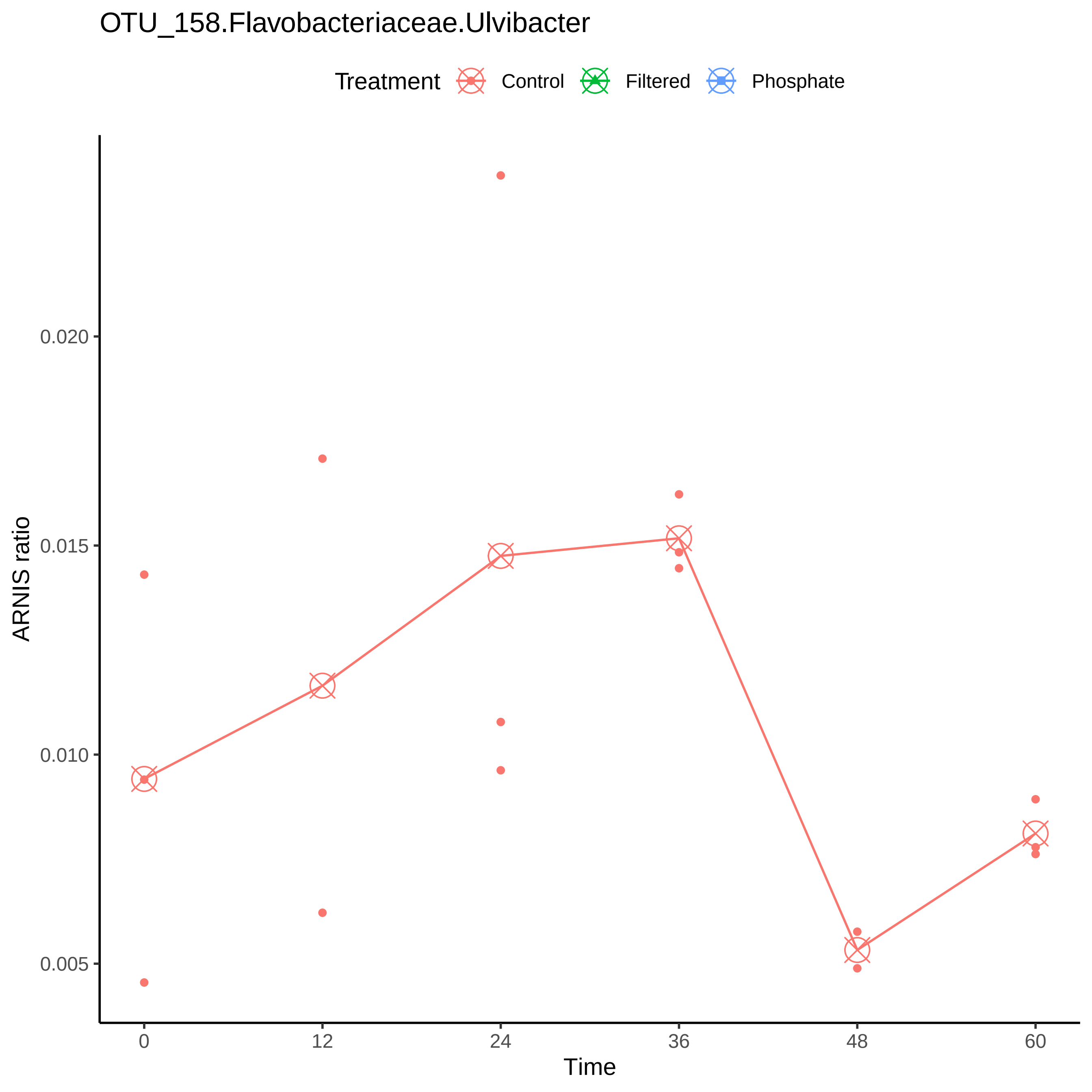

OTU_158.Flavobacteriaceae.Ulvibacter
Treatment
Control
Filtered
Phosphate
ARNIS ratio
0.020
0.015
0.010
0.005
Time
0
12
24
36
48
60

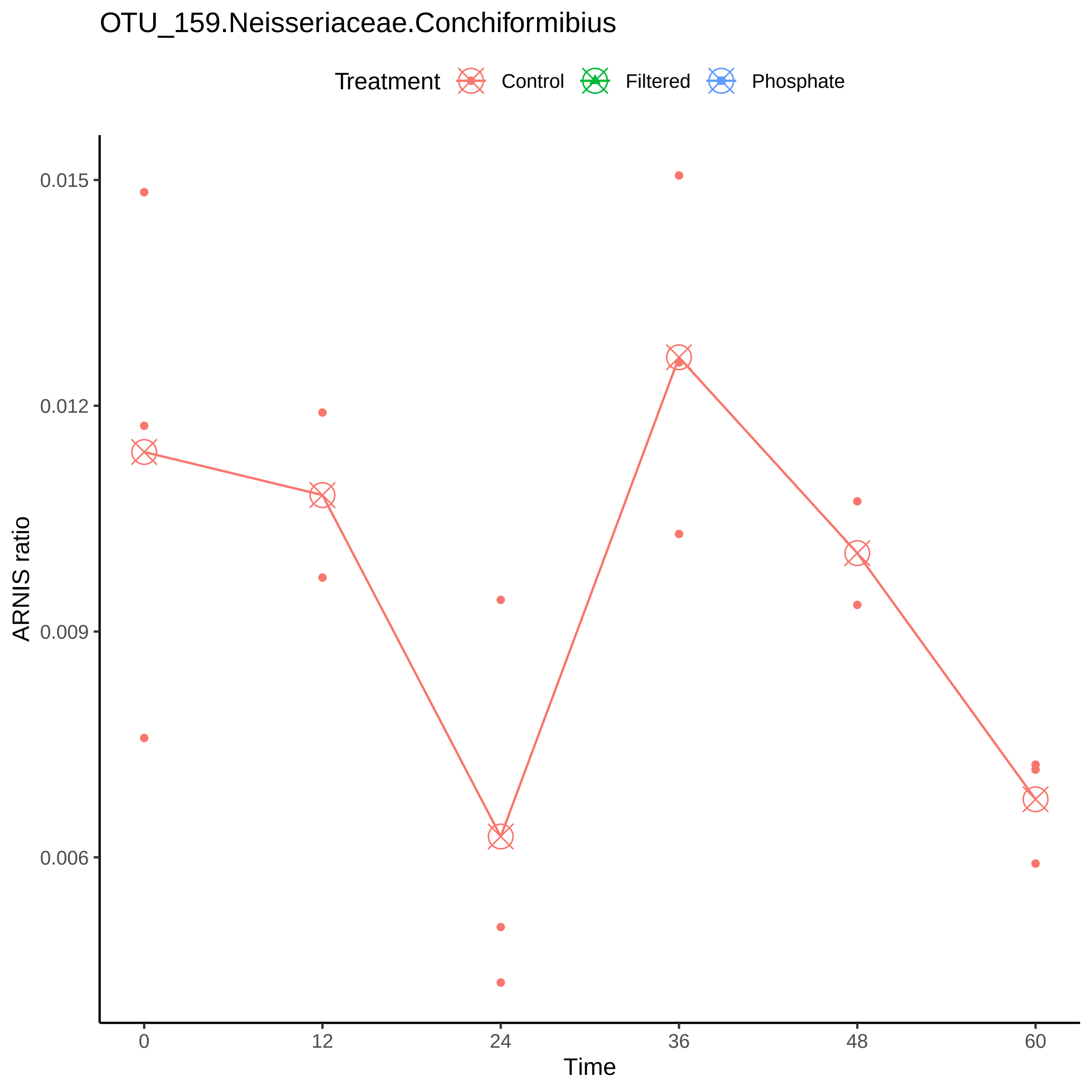

OTU_159.Neisseriaceae.Conchiformibius
Treatment
Control
Filtered
Phosphate
ARNIS ratio
0.015
0.012
0.009
0.006
0
12
24
36
48
60
Time

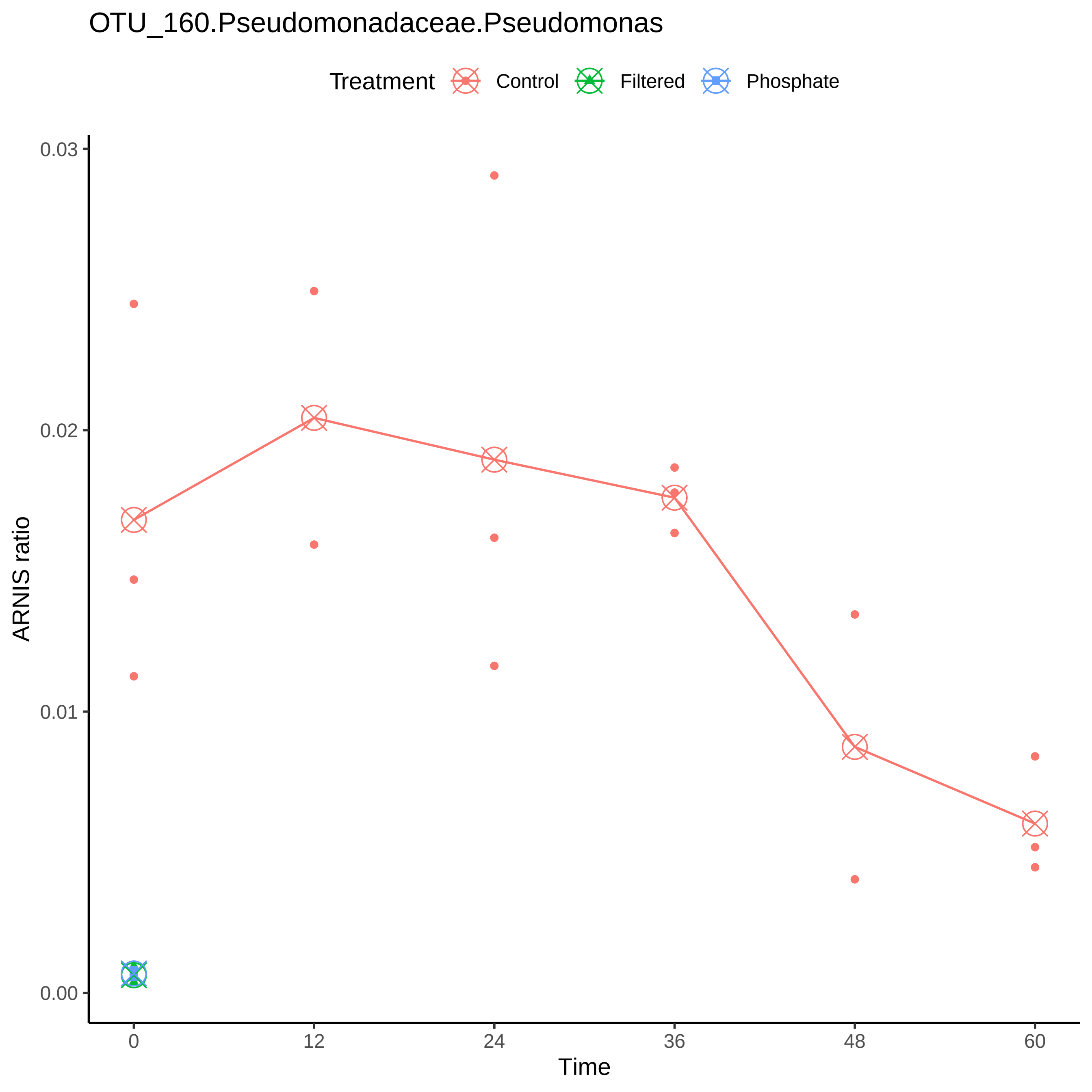

OTU_160.Pseudomonadaceae.Pseudomonas
Treatment
Control
Filtered
Phosphate
0.03
0.02
0.01
0.00
ARNIS ratio
0
12
24
36
48
60
Time

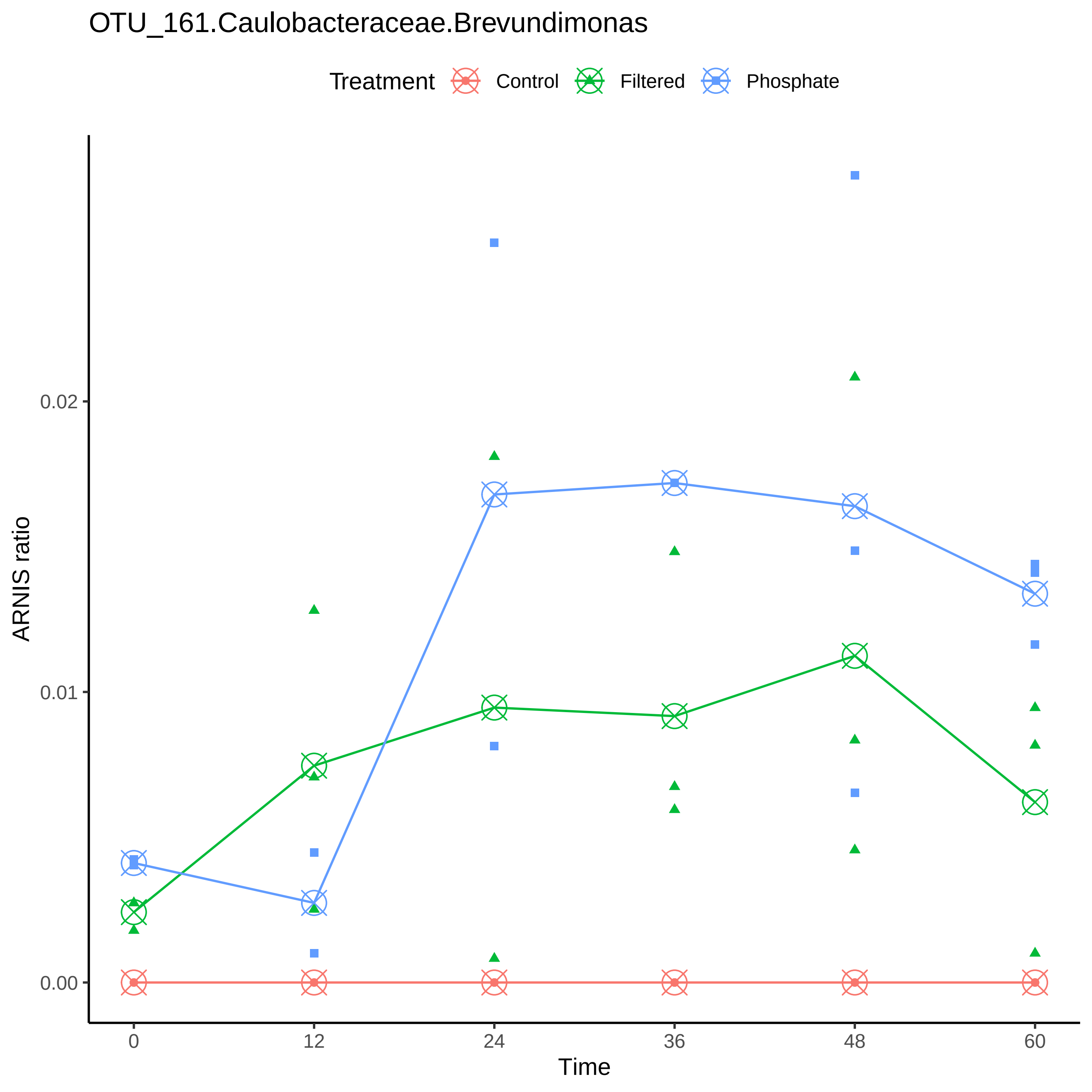
OTU_161.Caulobacteraceae.Brevundimonas
Treatment
Control
Filtered
Phosphate
ARNIS ratio
0.00
0.01
0.02
Time
0
12
24
36
48
60

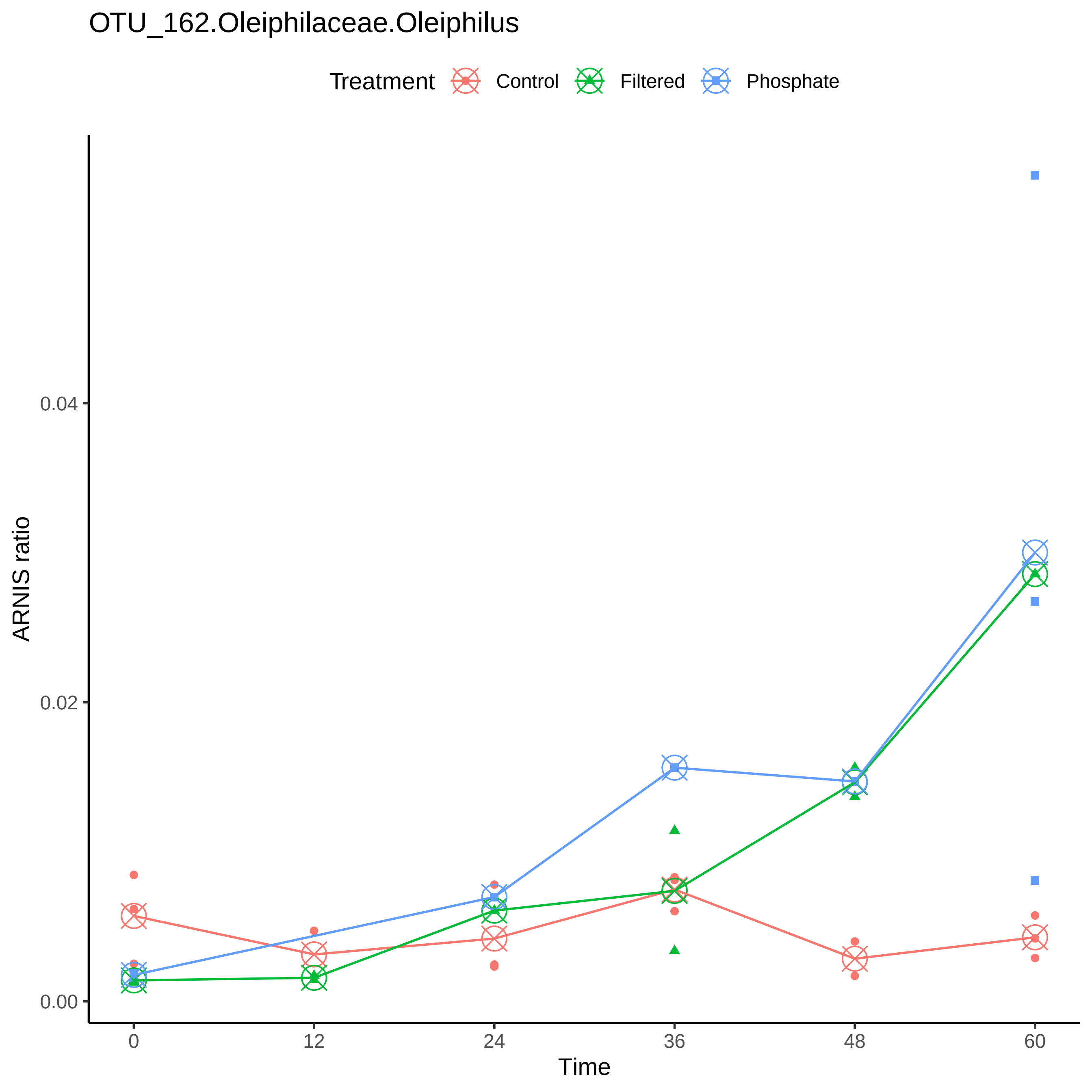

OTU_162.Oleiphilaceae.Oleiphilus
Treatment
Control
Filtered
Phosphate
ARNIS ratio
0.00
0.02
0.04
Time
0
12
24
36
48
60

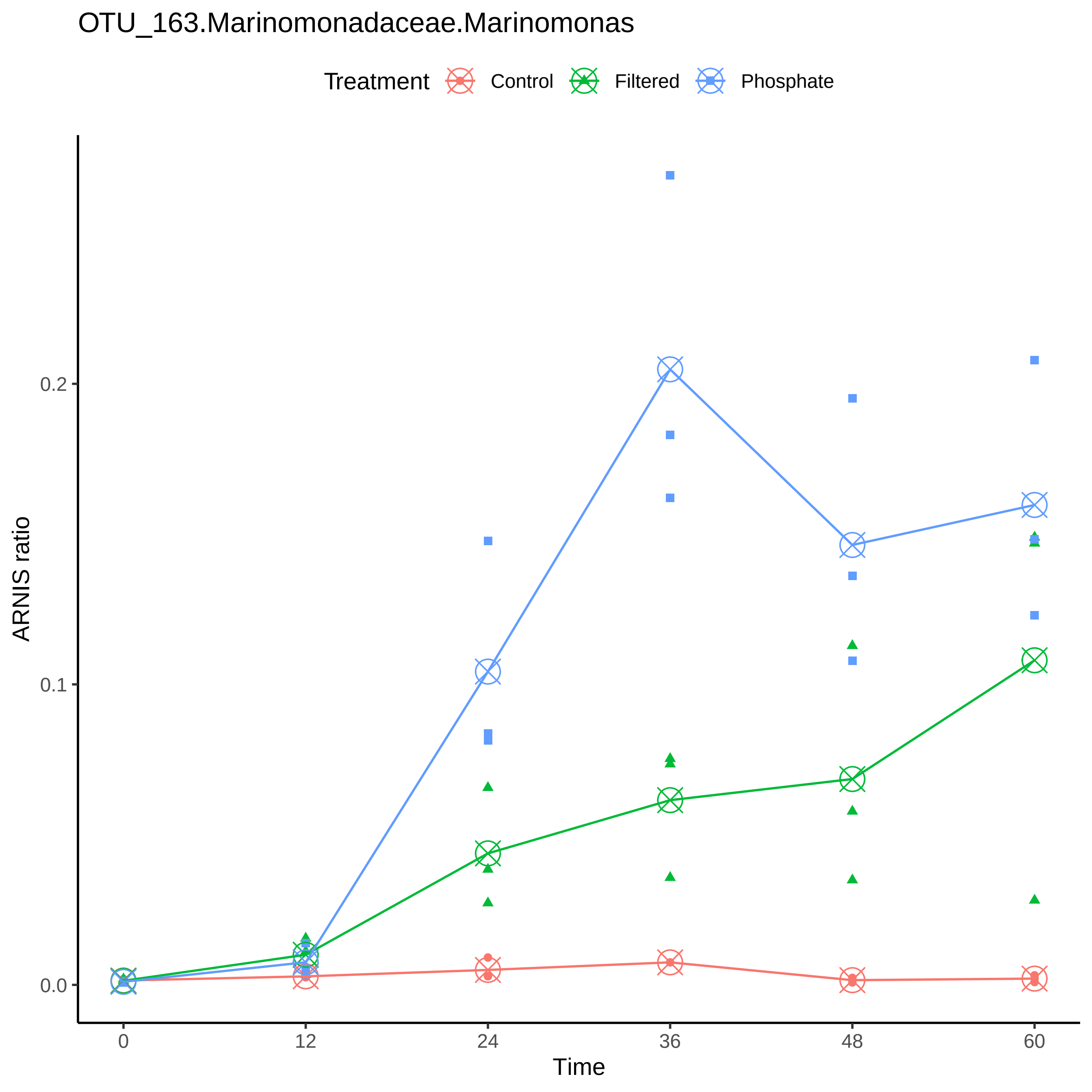
OTU_163.Marinomonadaceae.Marinomonas
Treatment
Control
Filtered
Phosphate
ARNIS ratio
0.0
0.1
0.2
Time
0
12
24
36
48
60

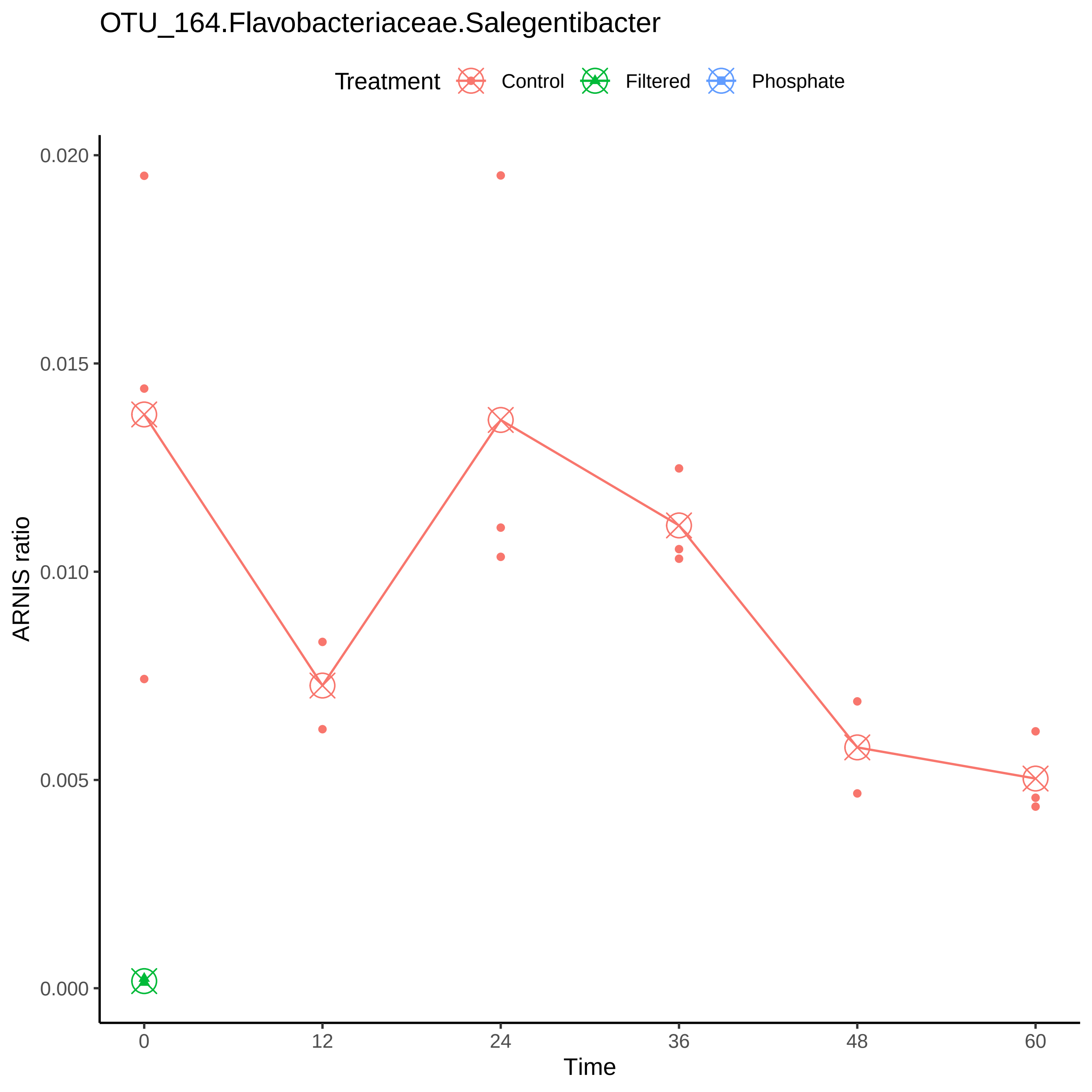

OTU_164.Flavobacteriaceae.Salegentibacter
Treatment
Control
Filtered
Phosphate
ARNIS ratio
0.020
0.015
0.010
0.005
0.000
0
12
24
36
48
60
Time

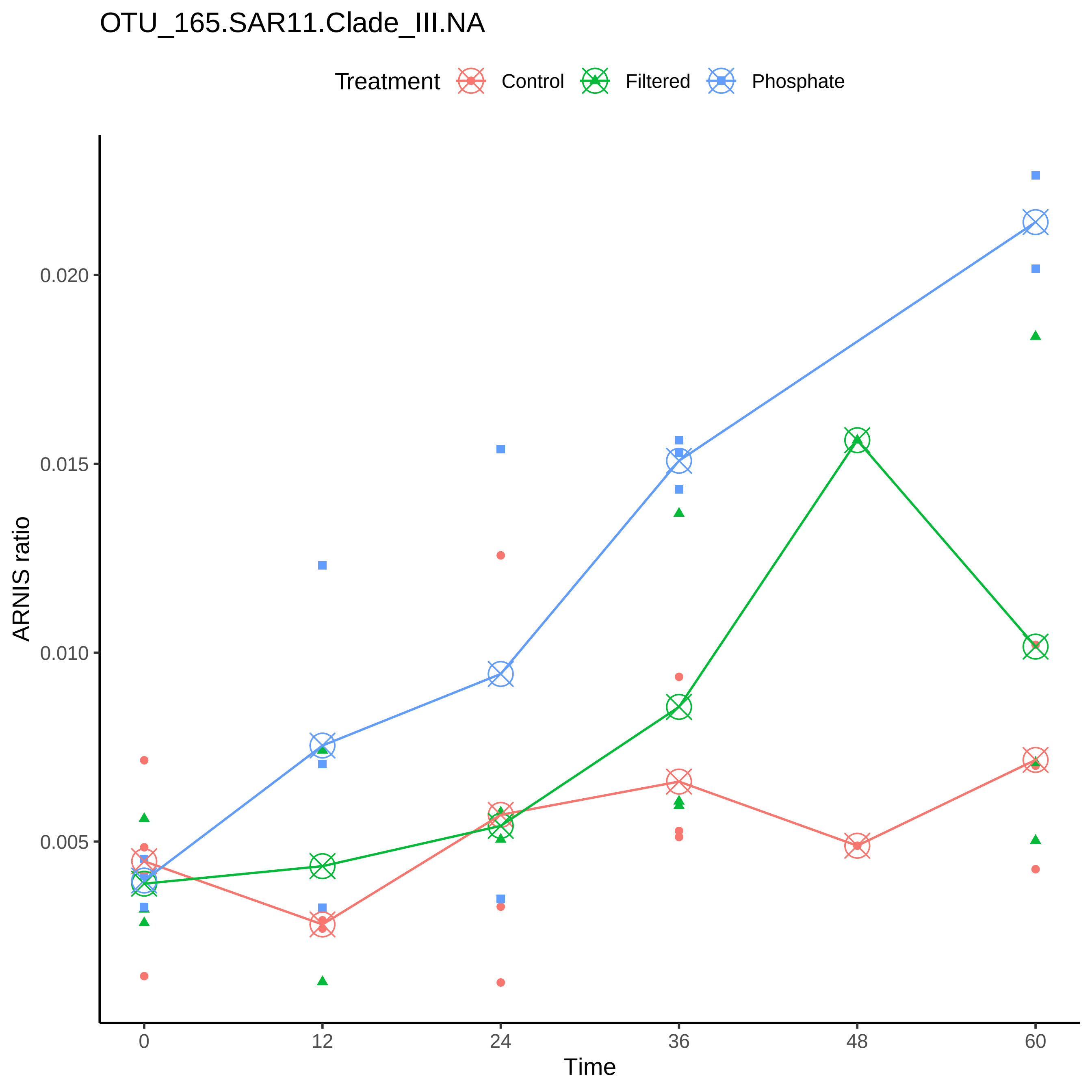
OTU_165.SAR11.Clade_III.NA
Treatment
Control
Filtered
Phosphate
ARNIS ratio
0.005
0.010
0.015
0.020
0
12
24
36
48
60
Time

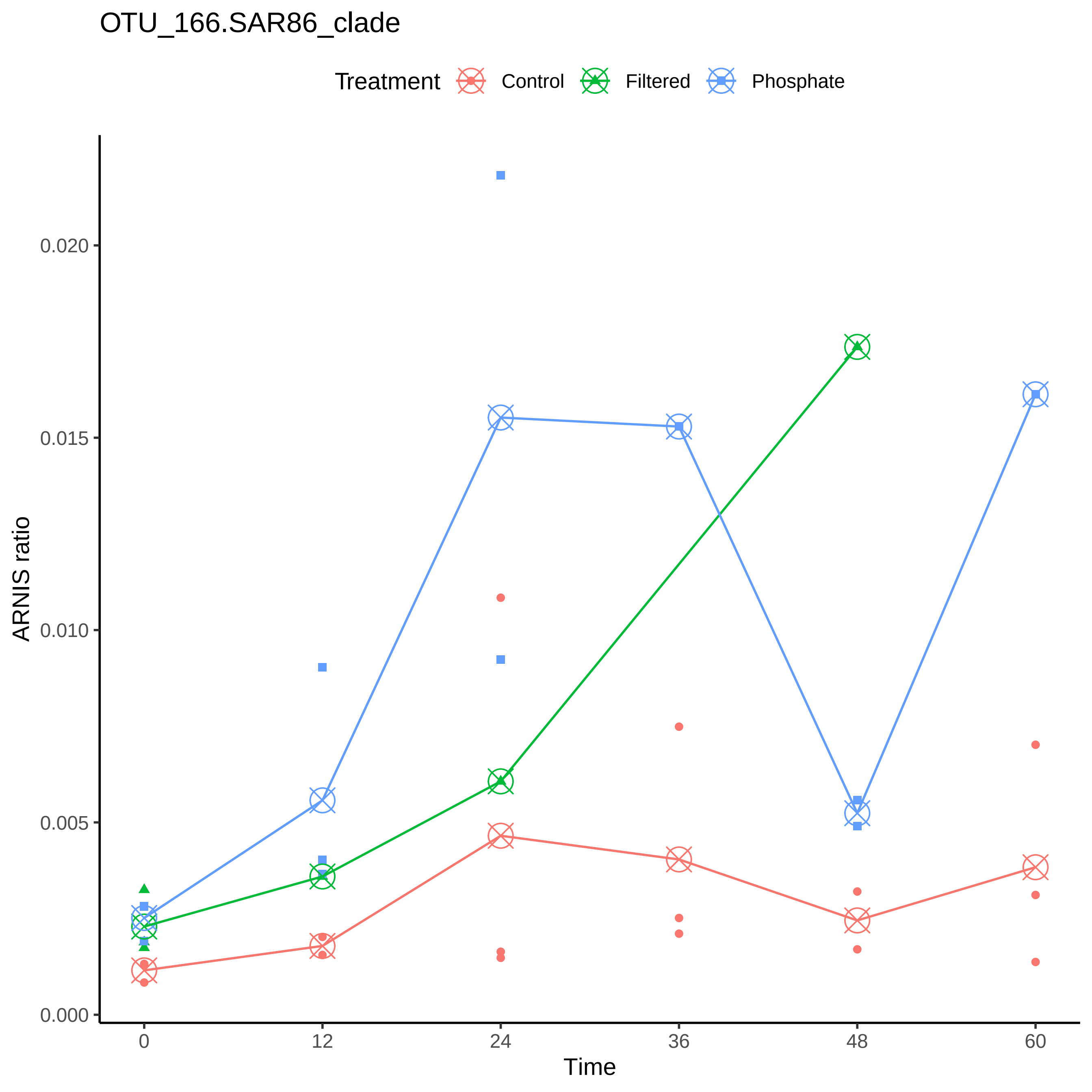
OTU_166.SAR86_clade
Treatment
Control
Filtered
Phosphate
ARNIS ratio
0.000
0.005
0.010
0.015
0.020
0
12
24
36
48
60
Time

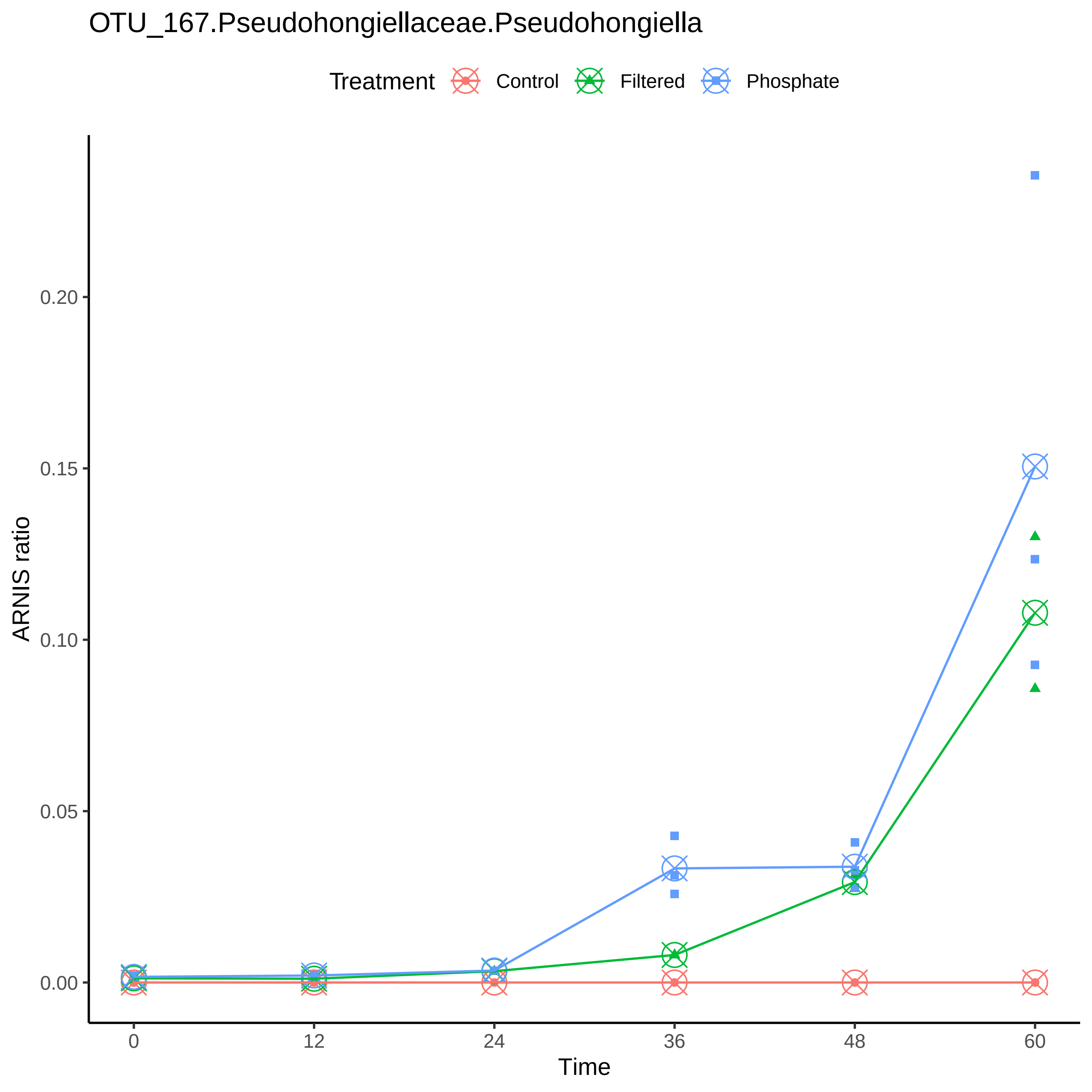

OTU_167.Pseudohongiellaceae.Pseudohongiella
Treatment
Control
Filtered
Phosphate
ARNIS ratio
0.00
0.05
0.10
0.15
0.20
0
12
24
36
48
60
Time

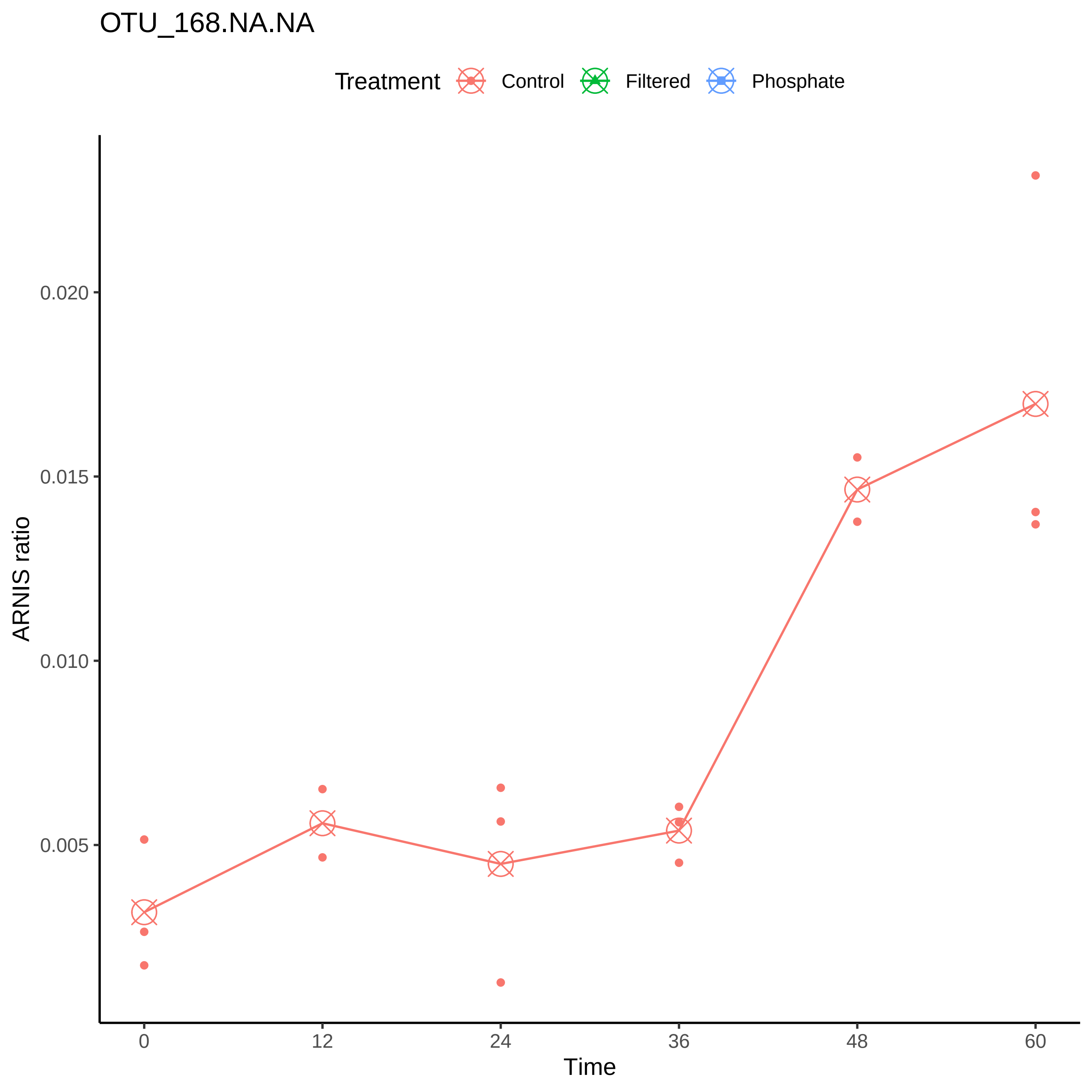
OTU_168.NA.NA
Treatment
Control
Filtered
Phosphate
ARNIS ratio
0.020
0.015
0.010
0.005
Time
0
12
24
36
48
60

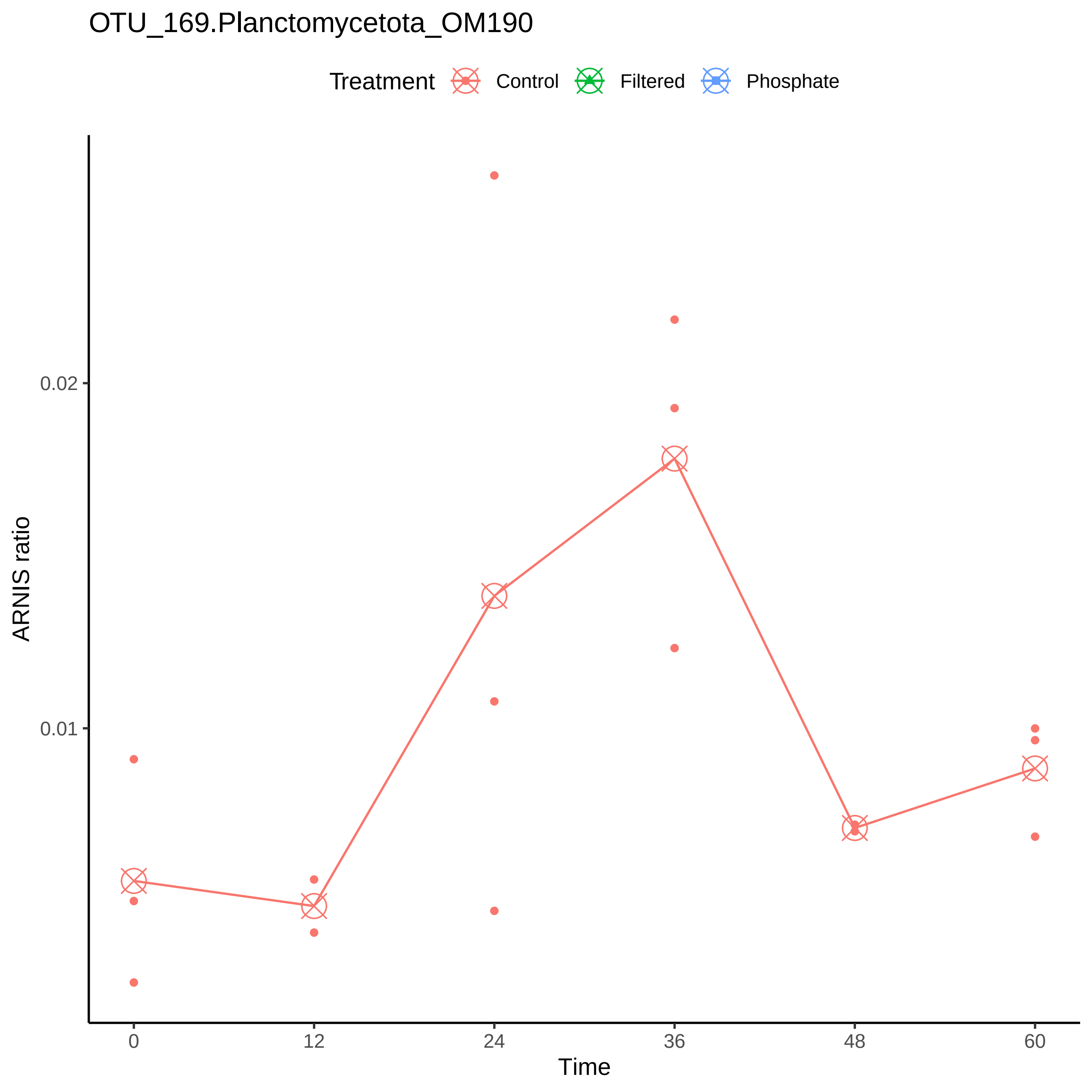

OTU_169.Planctomycetota_OM190
Treatment
Control
Filtered
Phosphate
ARNIS ratio
0.02
0.01
0
12
24
36
48
60
Time

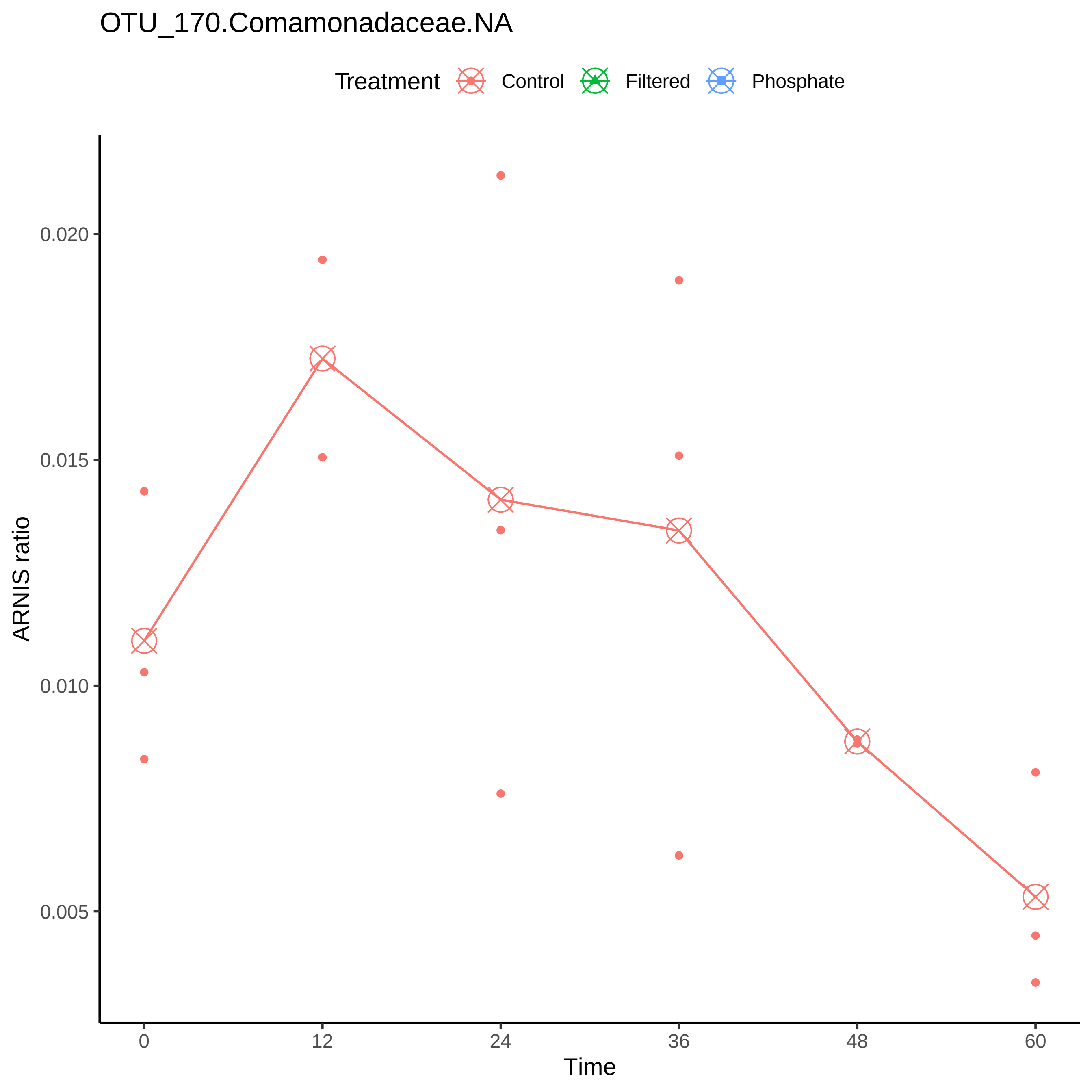
OTU_170.Comamonadaceae.NA
Treatment
Control
Filtered
Phosphate
ARNIS ratio
0.020
0.015
0.010
0.005
0
12
24
36
48
60
Time

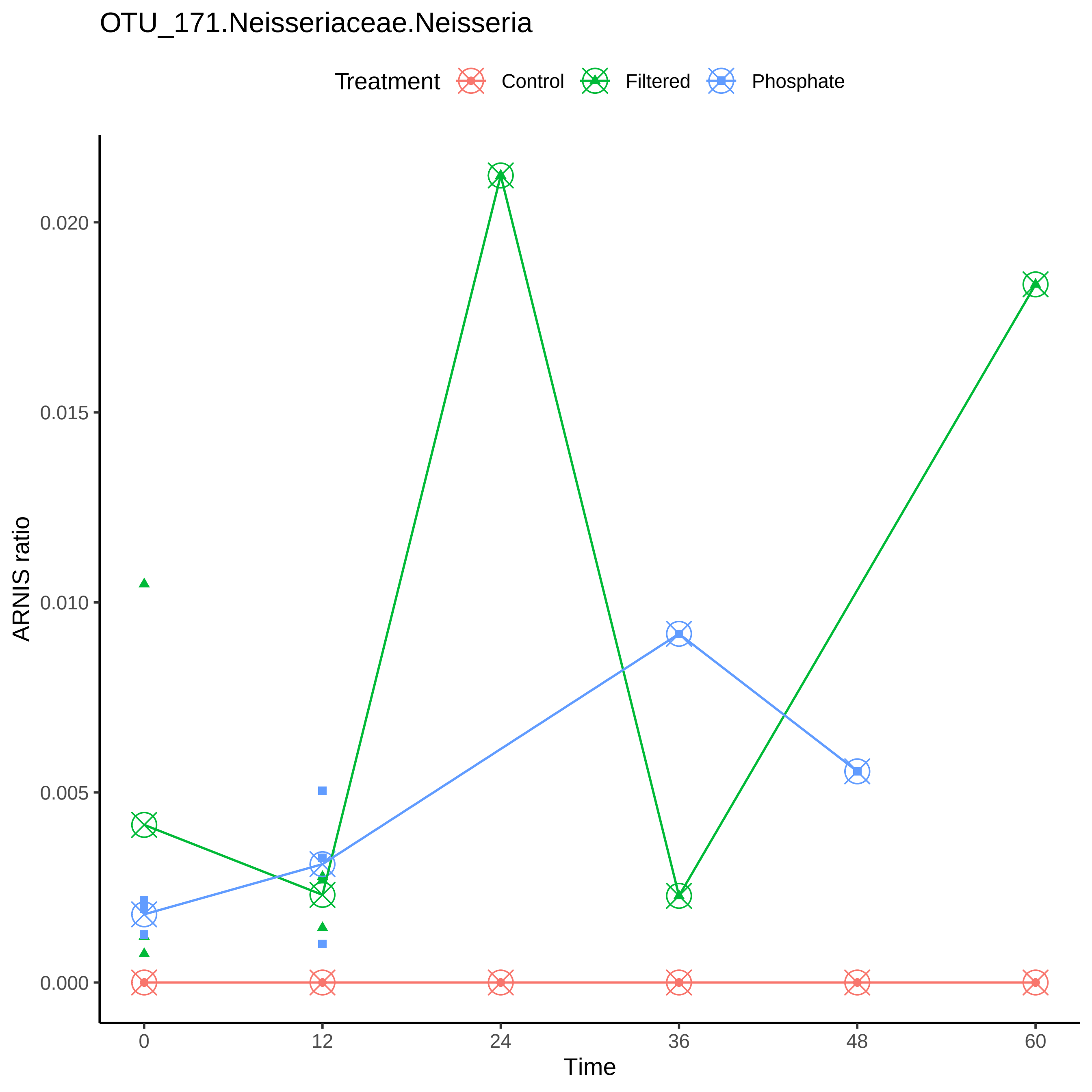
OTU_171.Neisseriaceae.Neisseria
Treatment
Control
Filtered
Phosphate
ARNIS ratio
0.020
0.015
0.010
0.005
0.000
0
12
24
36
48
60
Time

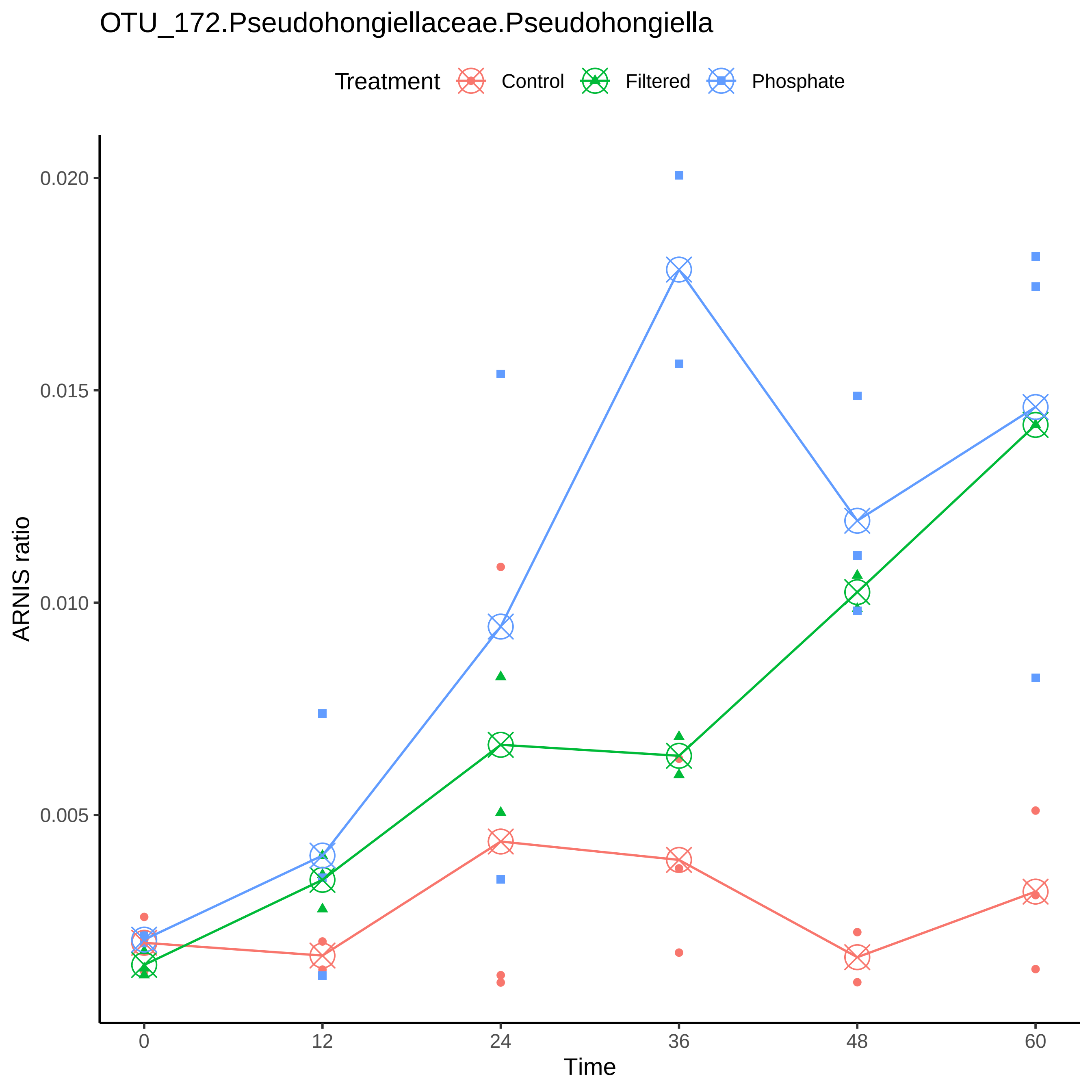

OTU_172.Pseudohongiellaceae.Pseudohongiella
Treatment
Control
Filtered
Phosphate
ARNIS ratio
0.020
0.015
0.010
0.005
0
12
24
36
48
60
Time

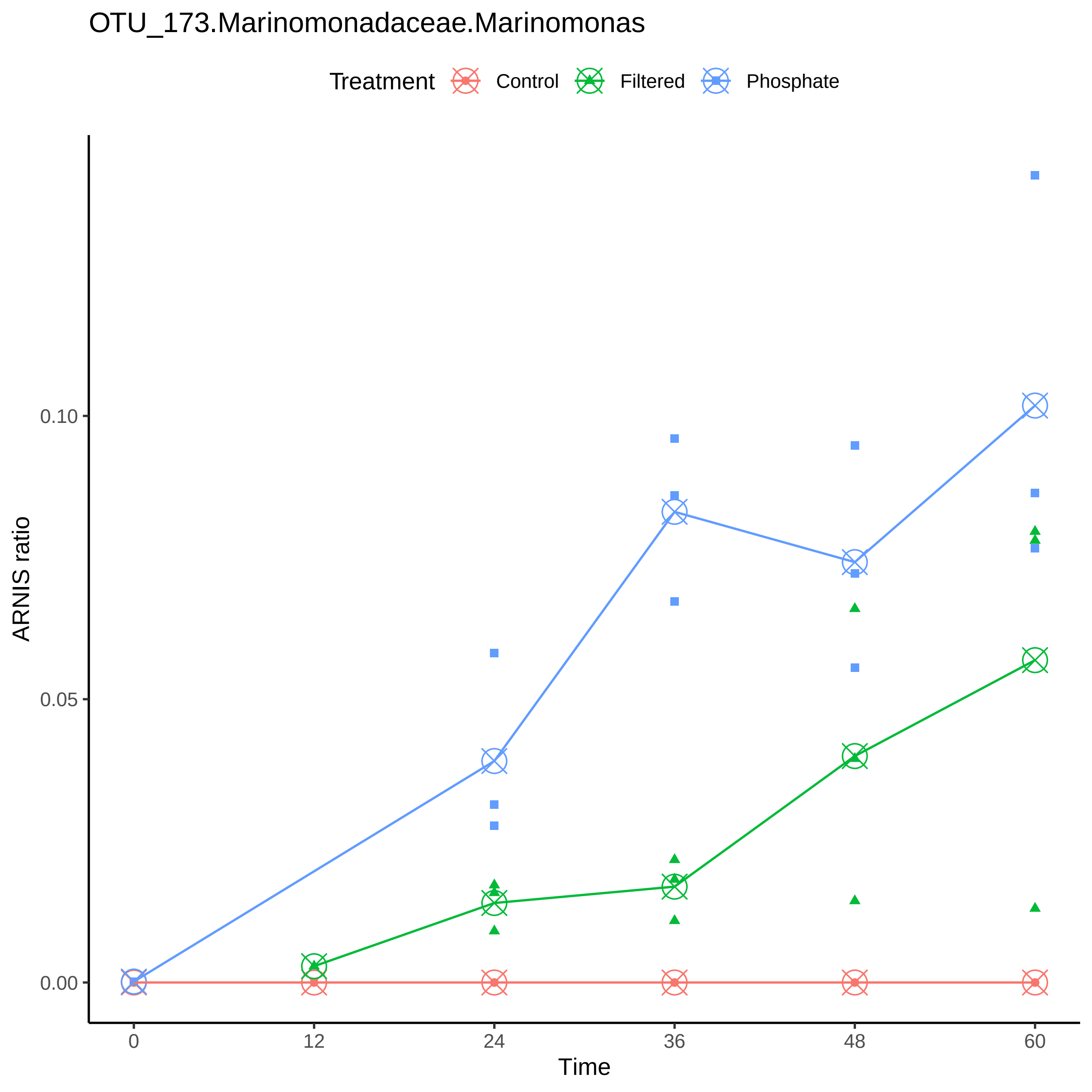
OTU_173.Marinomonadaceae.Marinomonas
Treatment
Control
Filtered
Phosphate
ARNIS ratio
0.00
0.05
0.10
0
12
24
36
48
60
Time

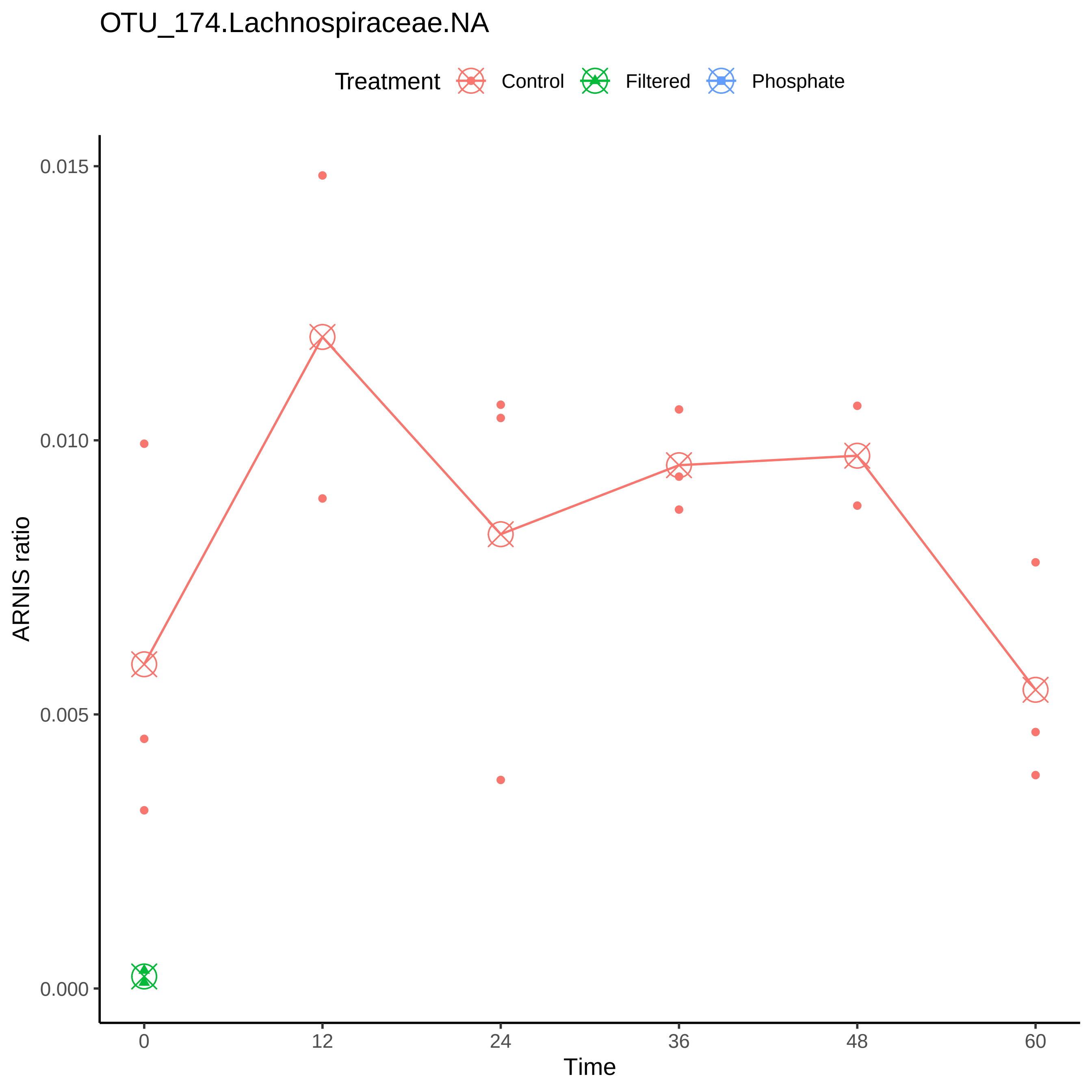

OTU_174.Lachnospiraceae.NA
Treatment
Control
Filtered
Phosphate
ARNIS ratio
0.015
0.010
0.005
0.000
0
12
24
36
48
60
Time

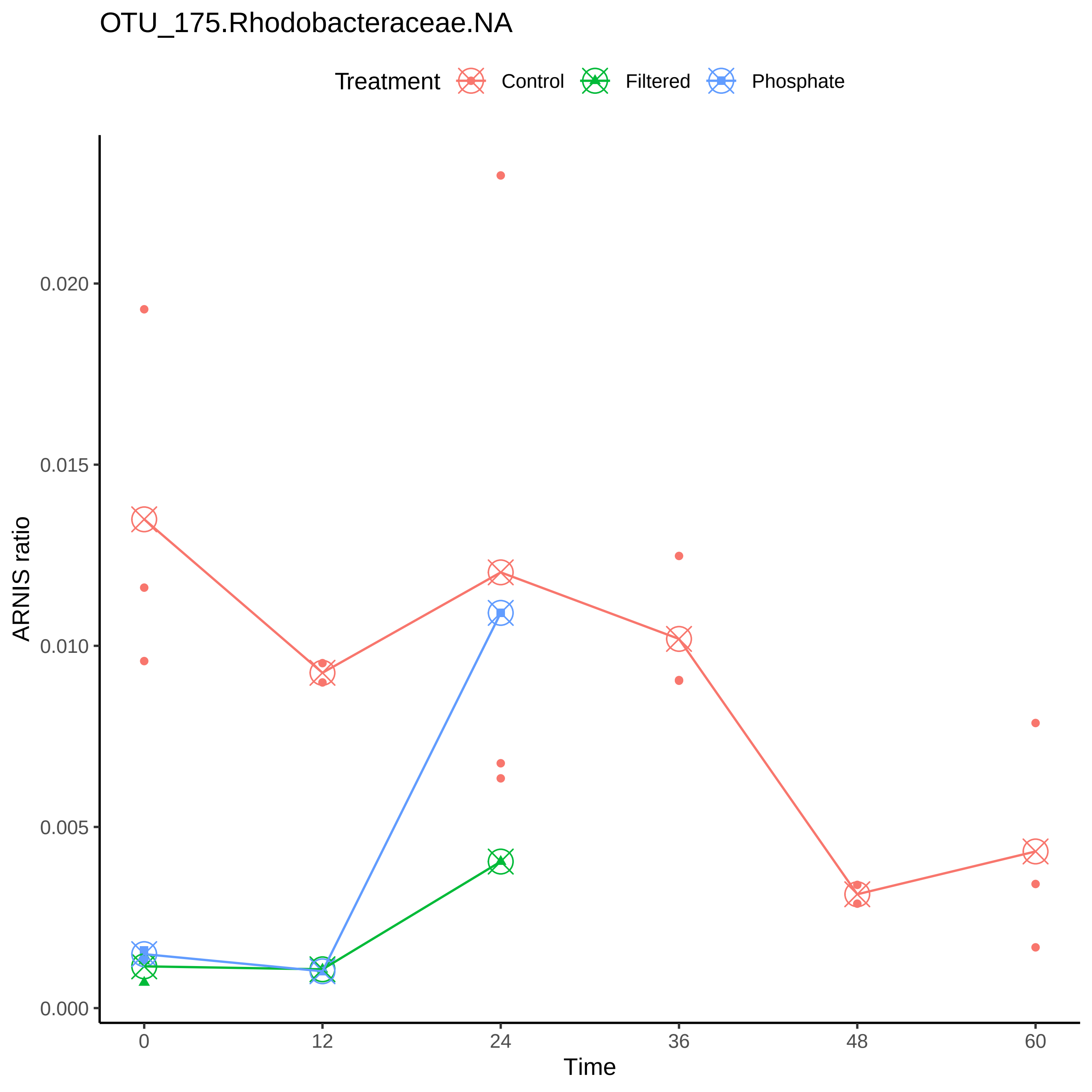

OTU_175.Rhodobacteraceae.NA
Treatment
Control
Filtered
Phosphate
ARNIS ratio
0.020
0.015
0.010
0.005
0.000
0
12
24
36
48
60
Time

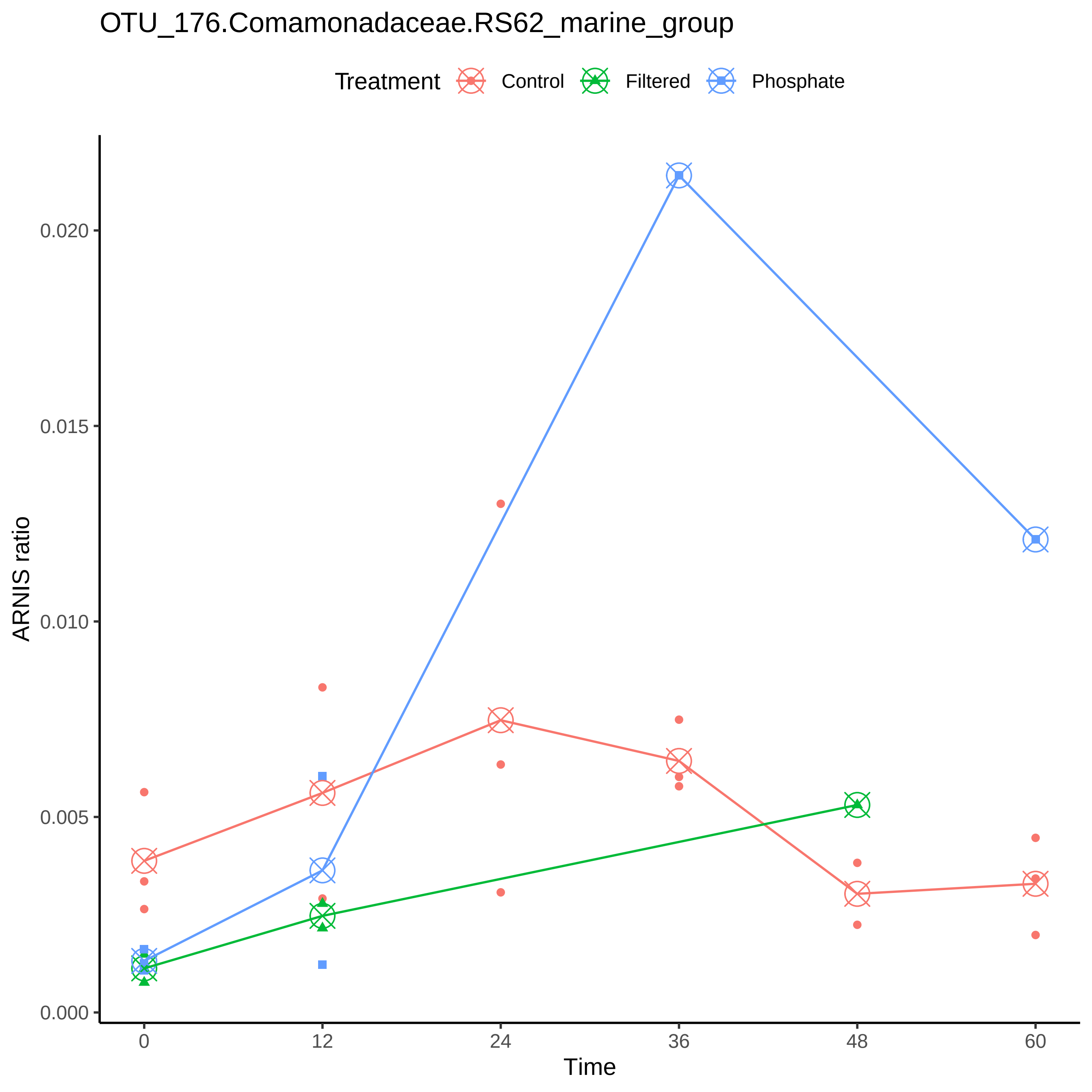

OTU_176.Comamonadaceae.RS62_marine_group
Treatment
Control
Filtered
Phosphate
ARNIS ratio
0.000
0.005
0.010
0.015
0.020
Time
0
12
24
36
48
60

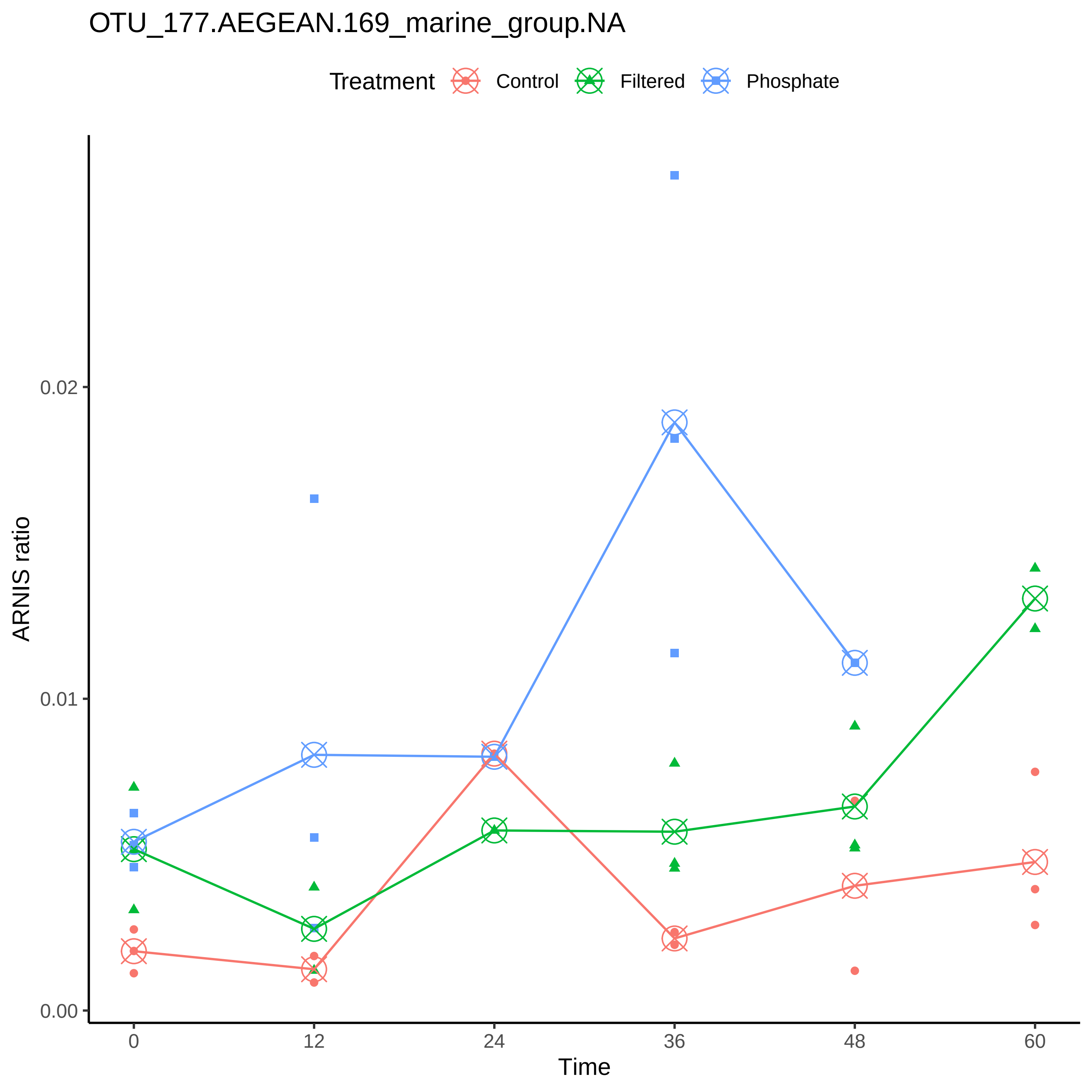

OTU_177.AEGEAN.169_marine_group.NA
Treatment
Control
Filtered
Phosphate
ARNIS ratio
0.00
0.01
0.02
Time
0
12
24
36
48
60

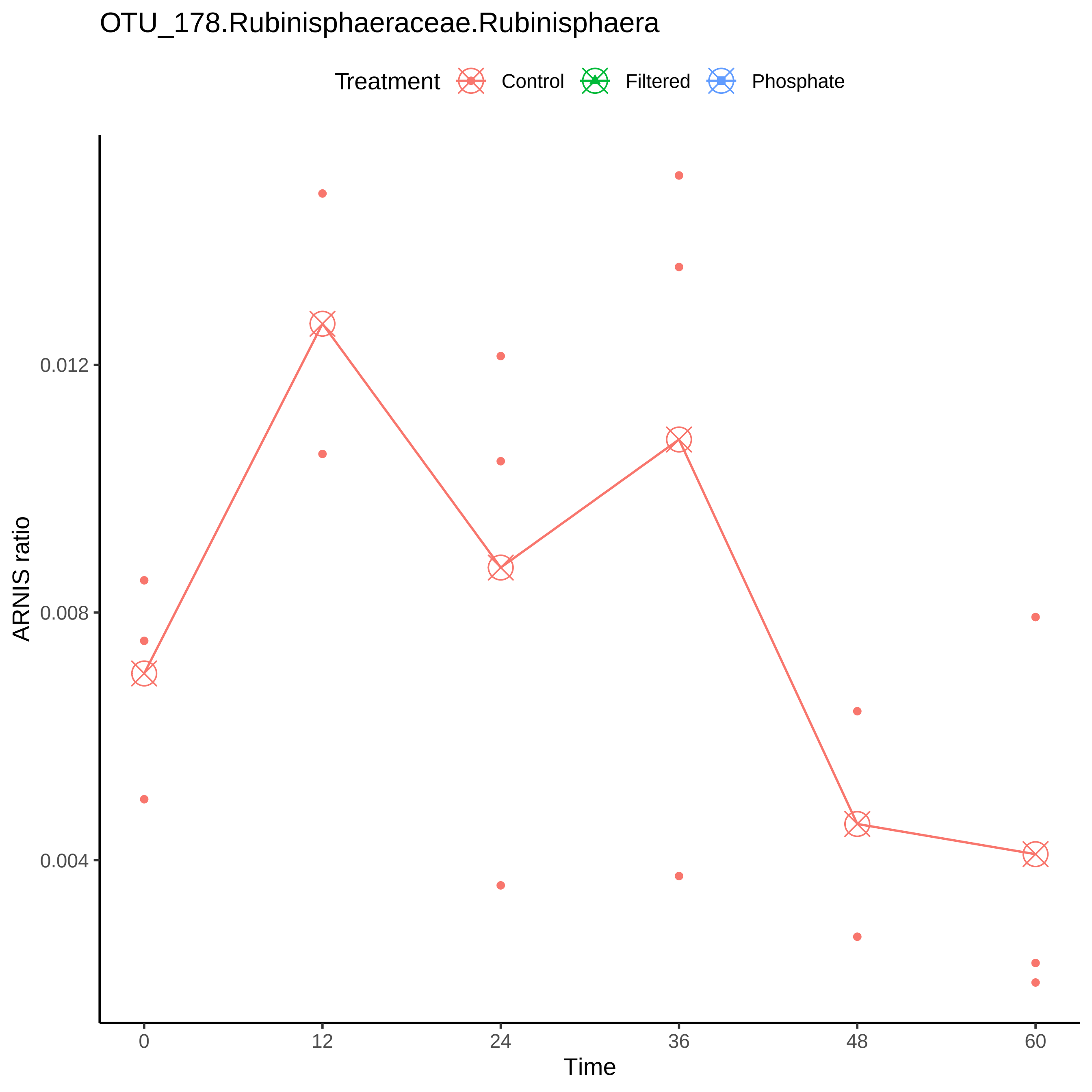

OTU_178.Rubinisphaeraceae.Rubinisphaera
Treatment
Control
Filtered
Phosphate
ARNIS ratio
0.012
0.008
0.004
0
12
24
36
48
60
Time

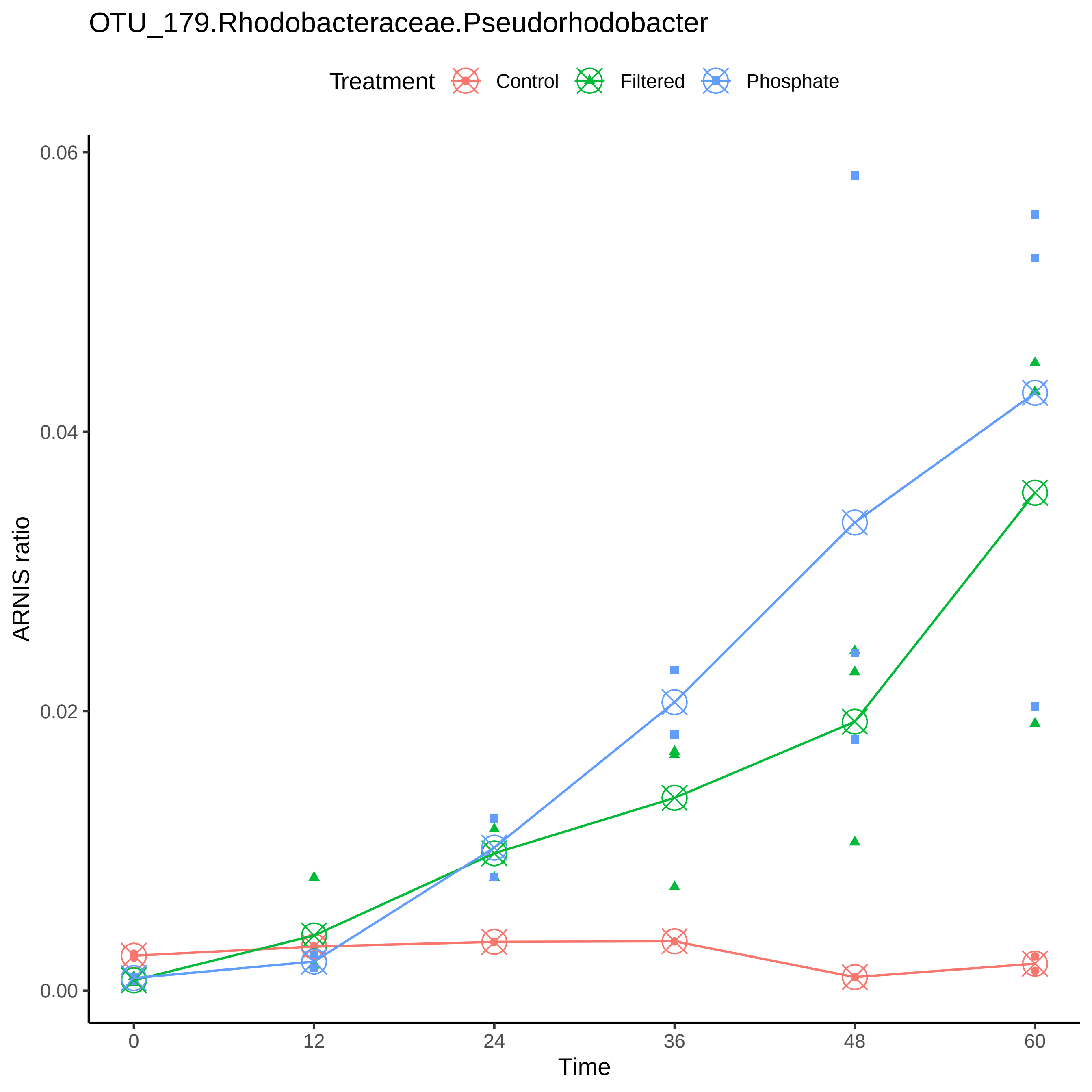
OTU_179.Rhodobacteraceae.Pseudorhodobacter
Treatment
Control
Filtered
Phosphate
ARNIS ratio
0.00
0.02
0.04
0.06
Time
0
12
24
36
48
60

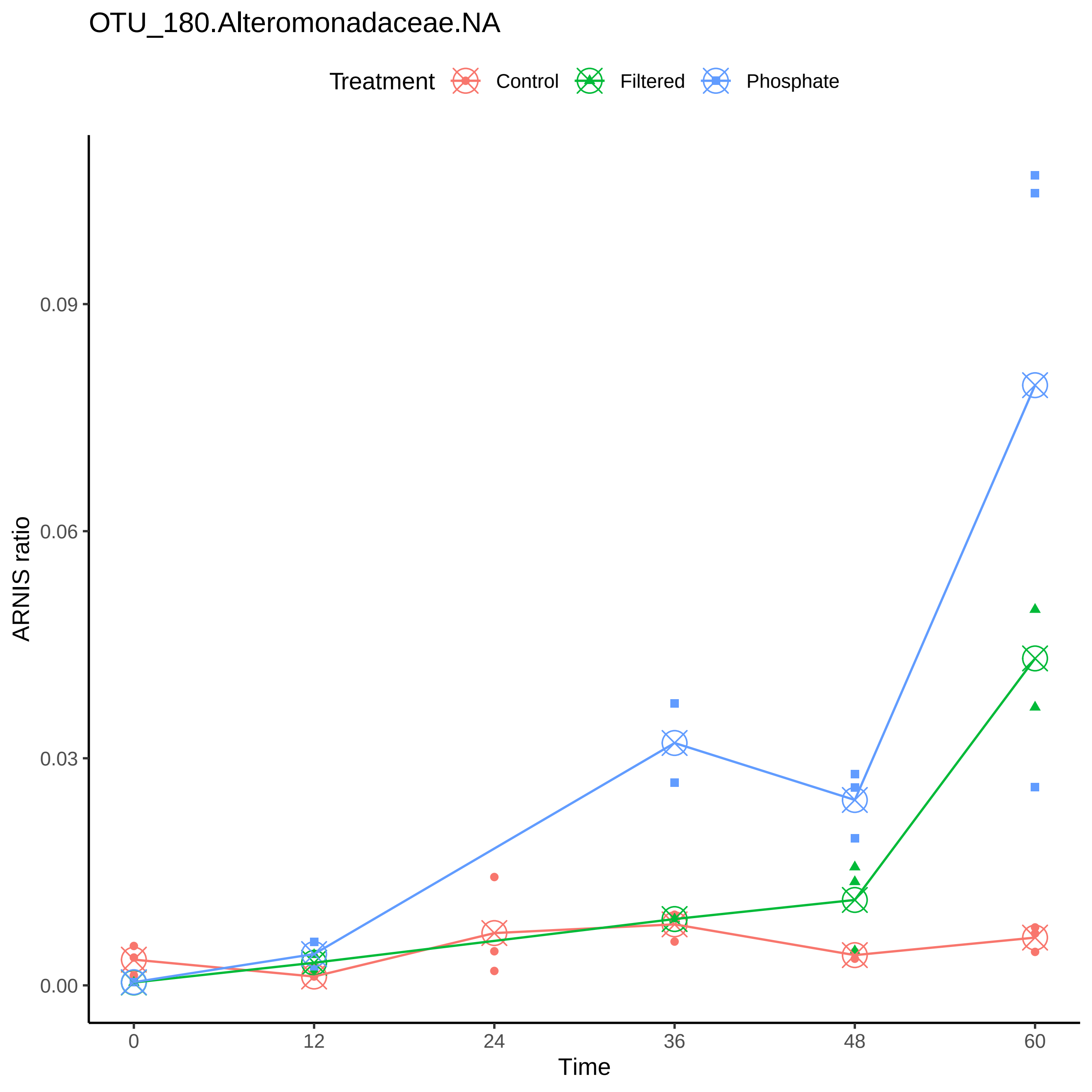
OTU_180.Alteromonadaceae.NA
Treatment
Control
Filtered
Phosphate
ARNIS ratio
0.09
0.06
0.03
0.00
0
12
24
36
48
60
Time

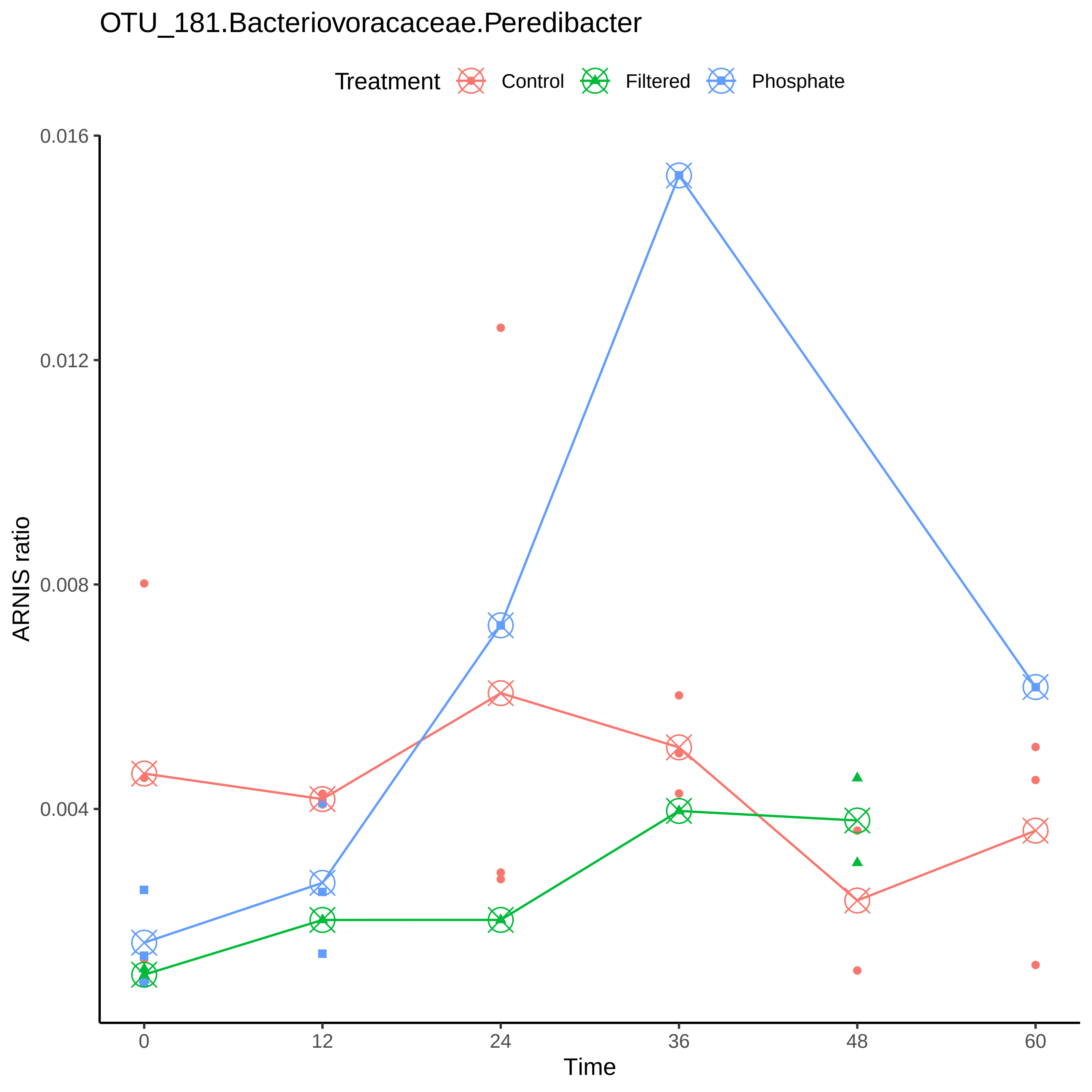
OTU_181.Bacteriovoracaceae.Peredibacter
Treatment
Control
Filtered
Phosphate
0.016
0.012
0.008
0.004
ARNIS ratio
0
12
24
36
48
60
Time

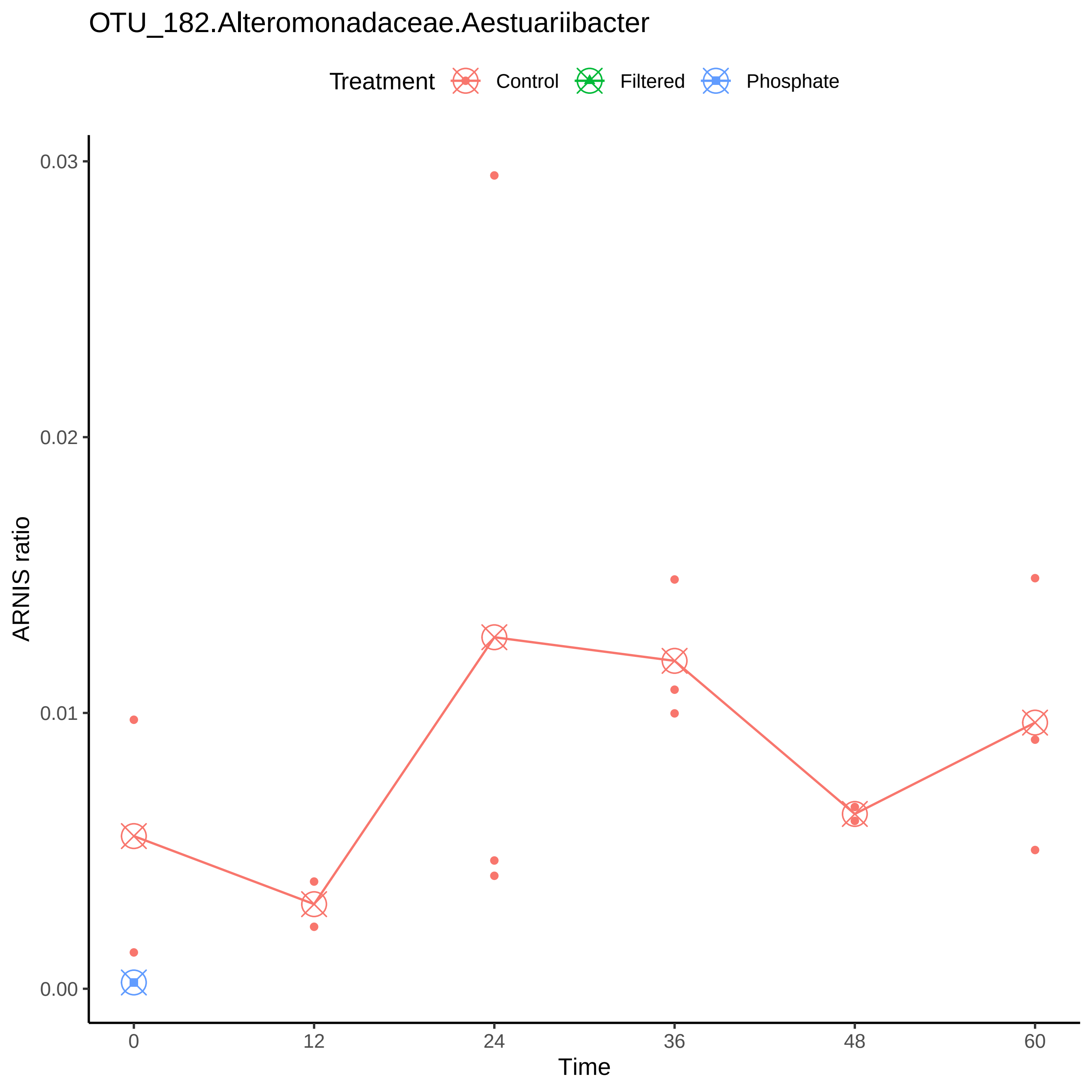

OTU_182.Alteromonadaceae.Aestuariibacter
Treatment
Control
Filtered
Phosphate
ARNIS ratio
0.03
0.02
0.01
0.00
0
12
24
36
48
60
Time

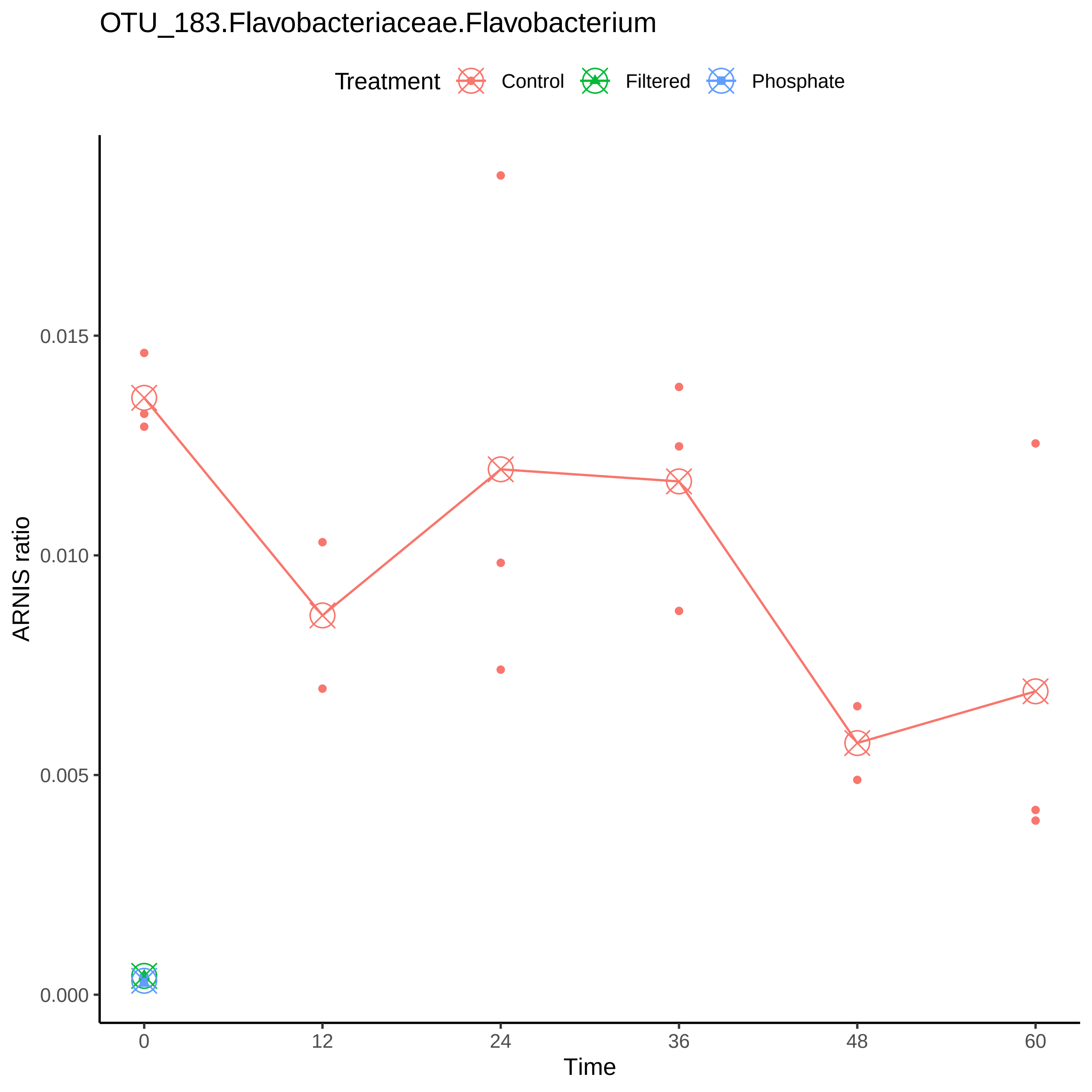
OTU_183.Flavobacteriaceae.Flavobacterium
Treatment
Control
Filtered
Phosphate
ARNIS ratio
0.000
0.005
0.010
0.015
Time
0
12
24
36
48
60

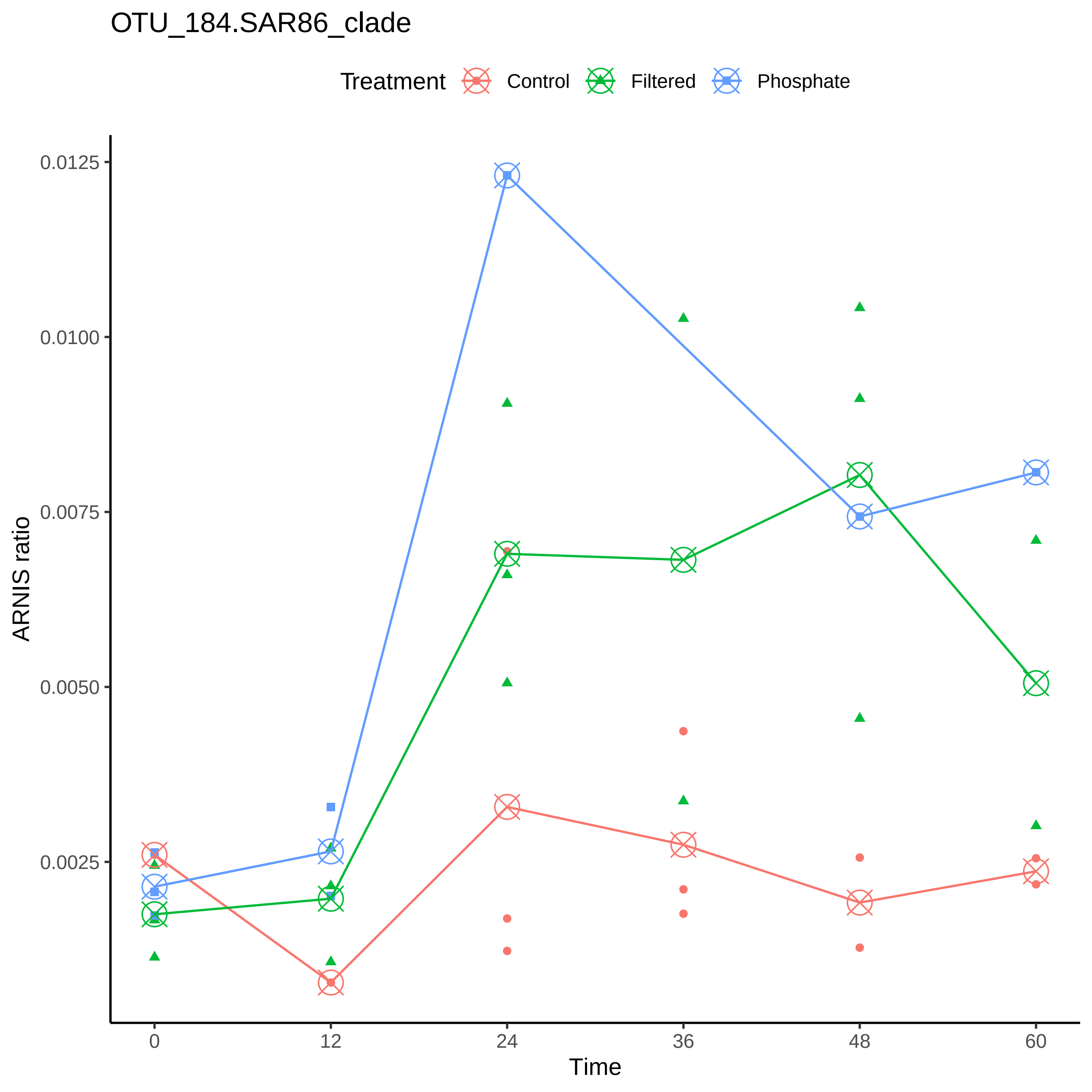
OTU_184.SAR86_clade
Treatment
Control
Filtered
Phosphate
ARNIS ratio
0.0125
0.0100
0.0075
0.0050
0.0025
0
12
24
36
48
60
Time

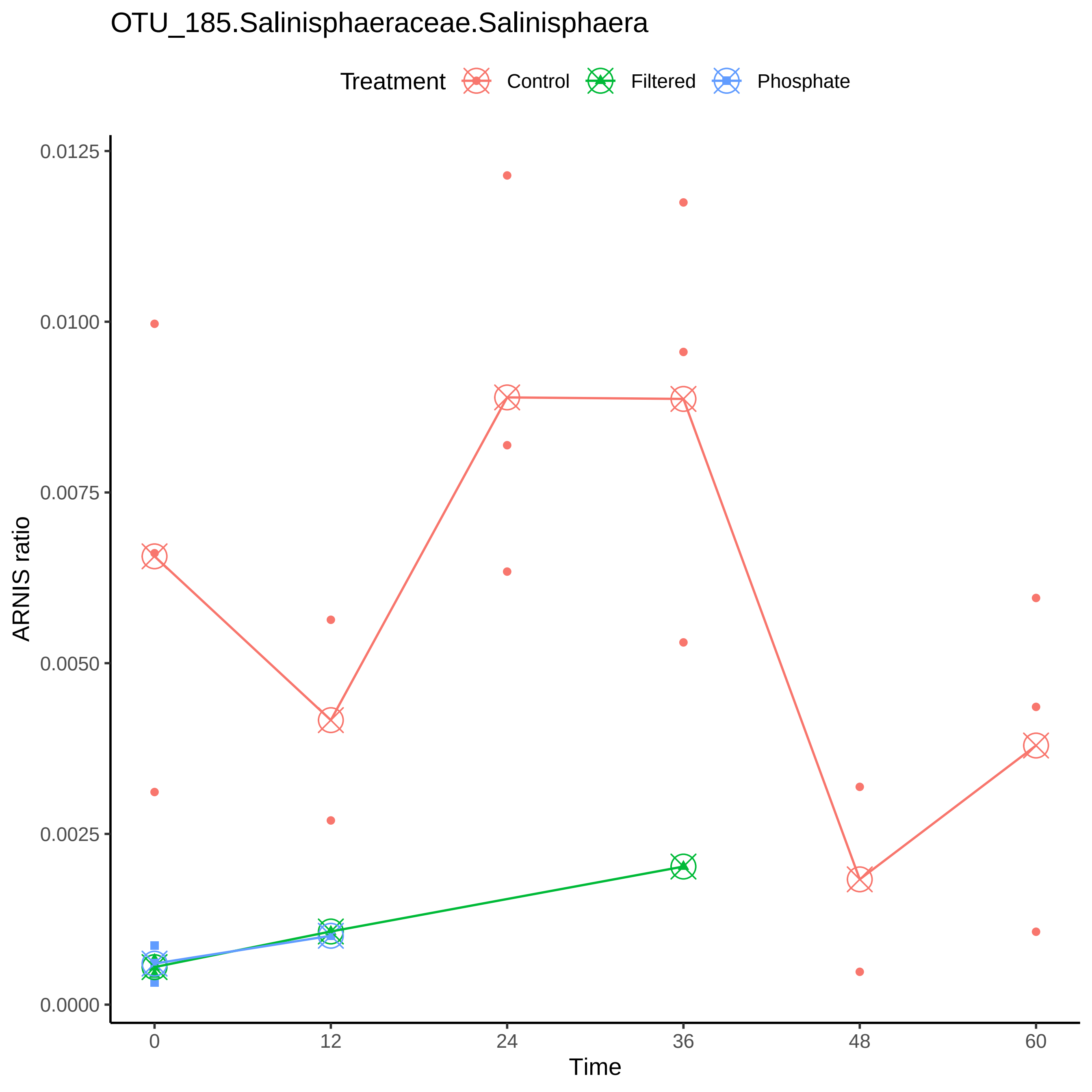
OTU_185.Salinisphaeraceae.Salinisphaera
Treatment
Control
Filtered
Phosphate
ARNIS ratio
0.0125
0.0100
0.0075
0.0050
0.0025
0.0000
0
12
24
36
48
60
Time

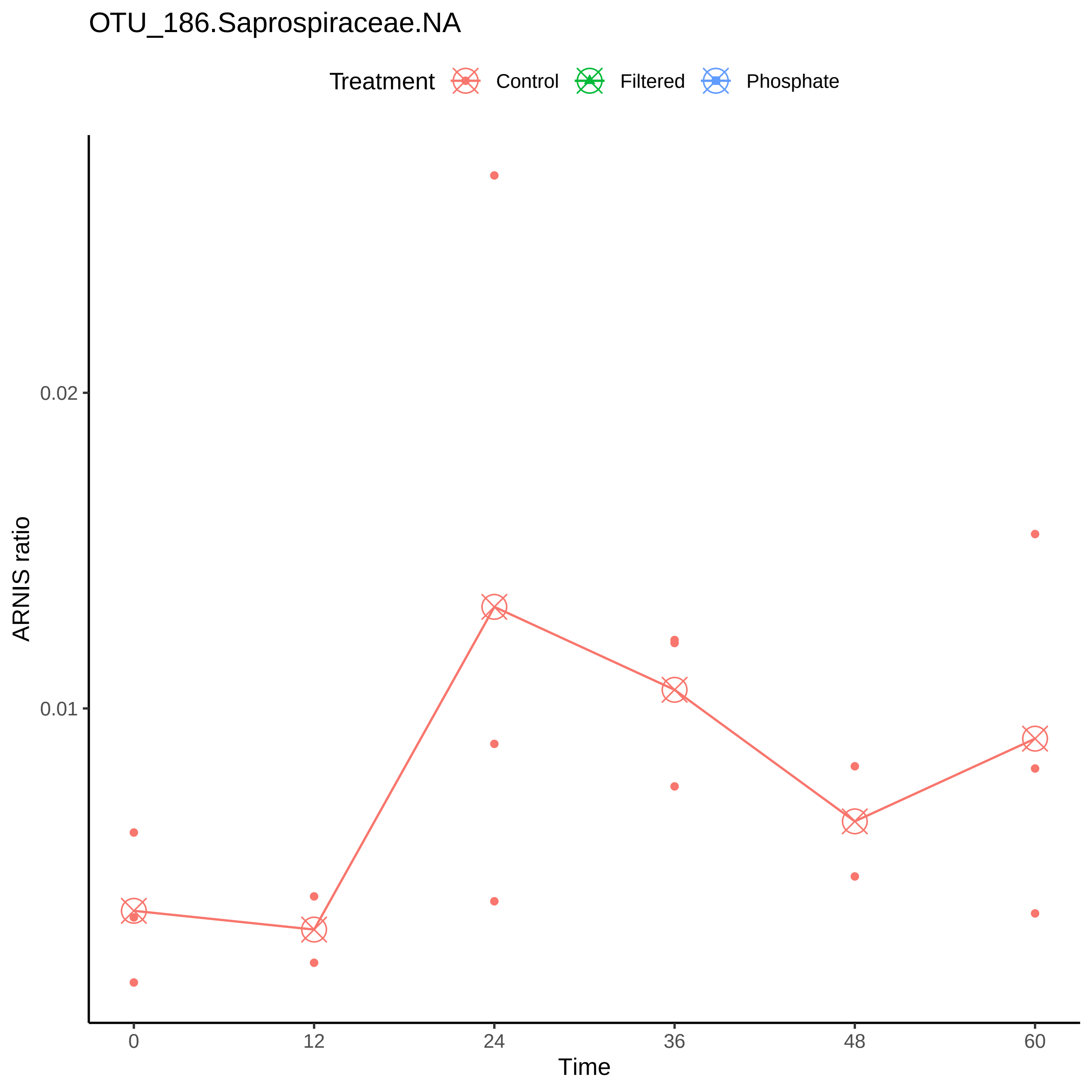

OTU_186.Saprospiraceae.NA
Treatment
Control
Filtered
Phosphate
ARNIS ratio
0.02
0.01
0
12
24
36
48
60
Time

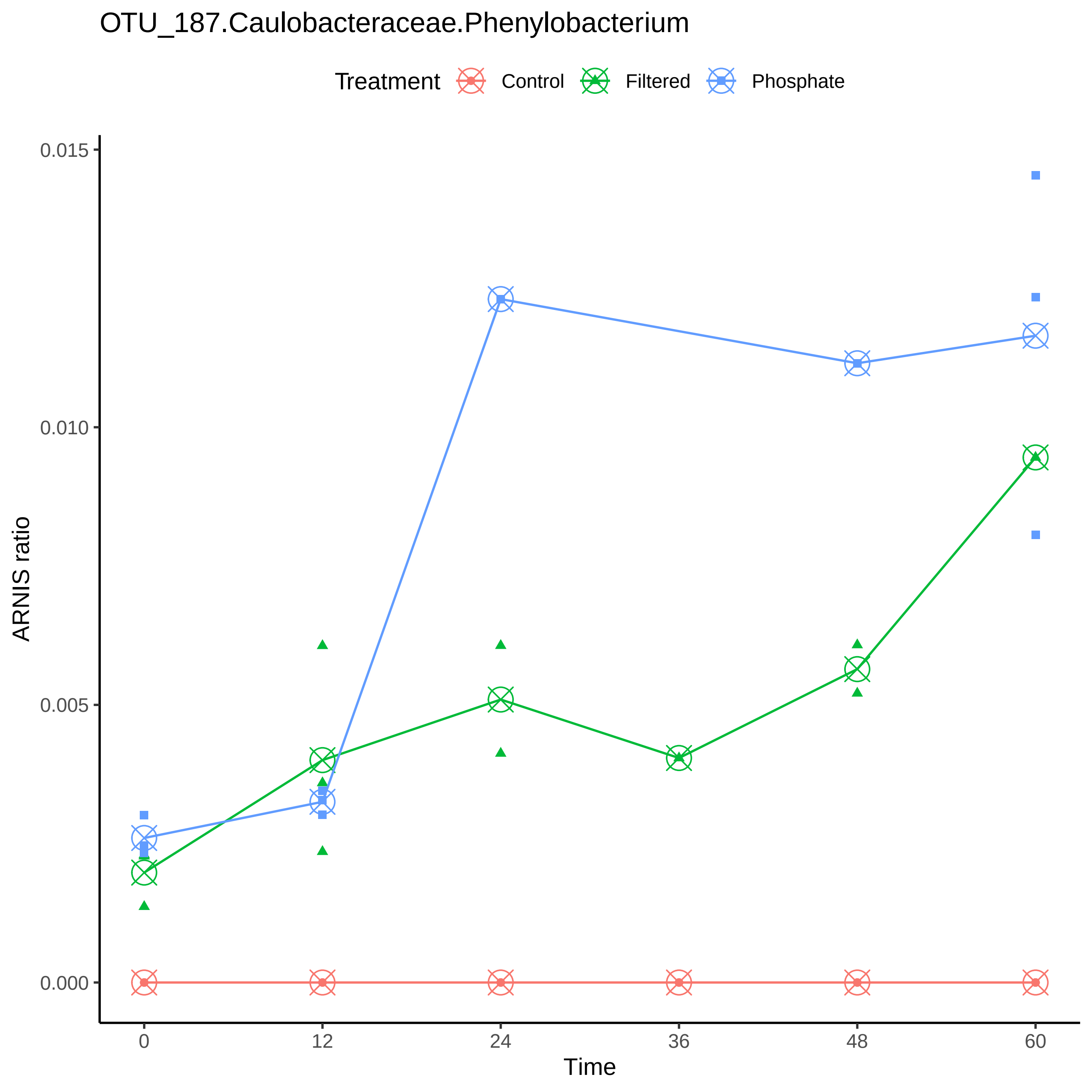

OTU_187.Caulobacteraceae.Phenylobacterium
Treatment
Control
Filtered
Phosphate
ARNIS ratio
0.015
0.010
0.005
0.000
Time
0
12
24
36
48
60

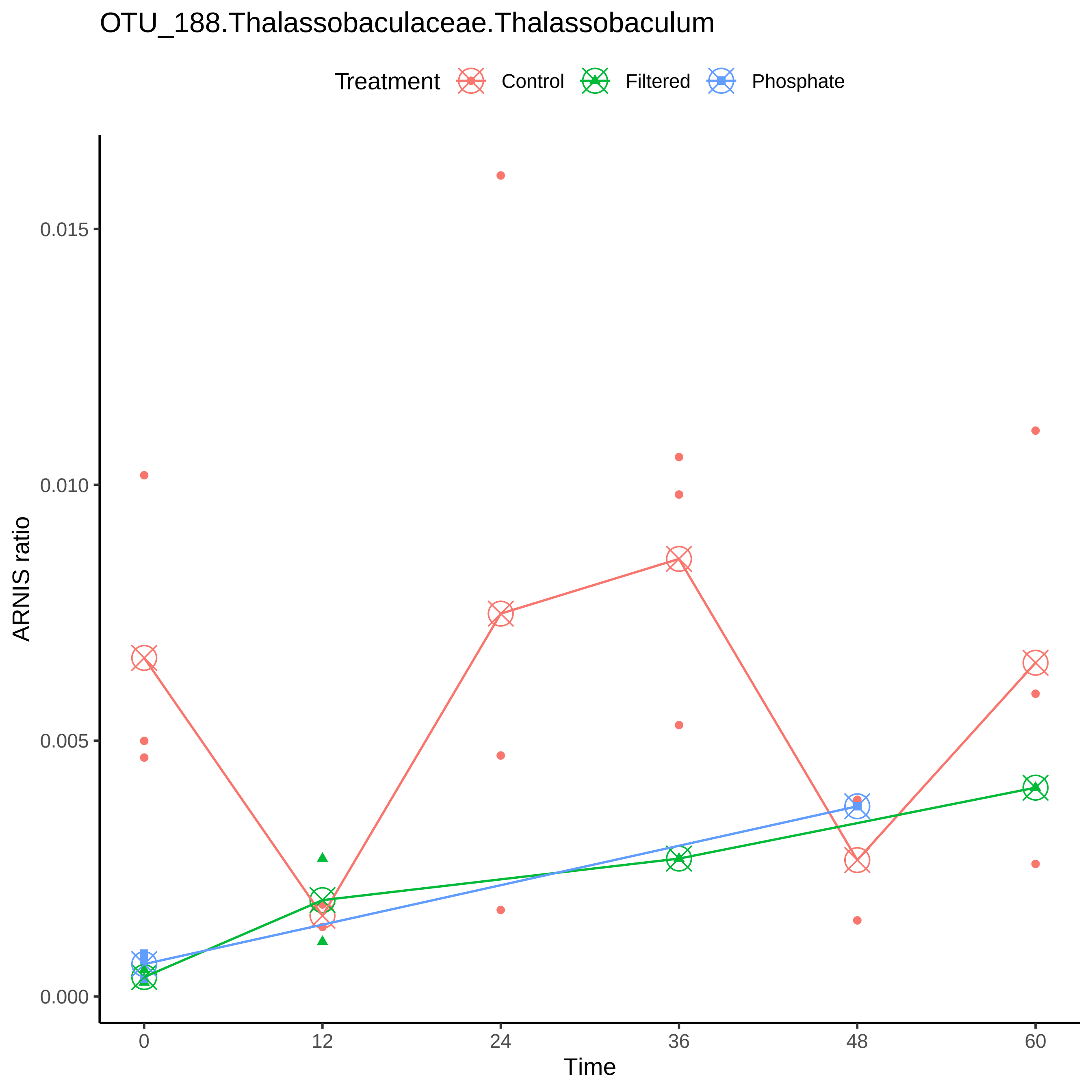

OTU_188.Thalassobaculaceae.Thalassobaculum
Treatment
Control
Filtered
Phosphate
ARNIS ratio
0.015
0.010
0.005
0.000
0
12
24
36
48
60
Time

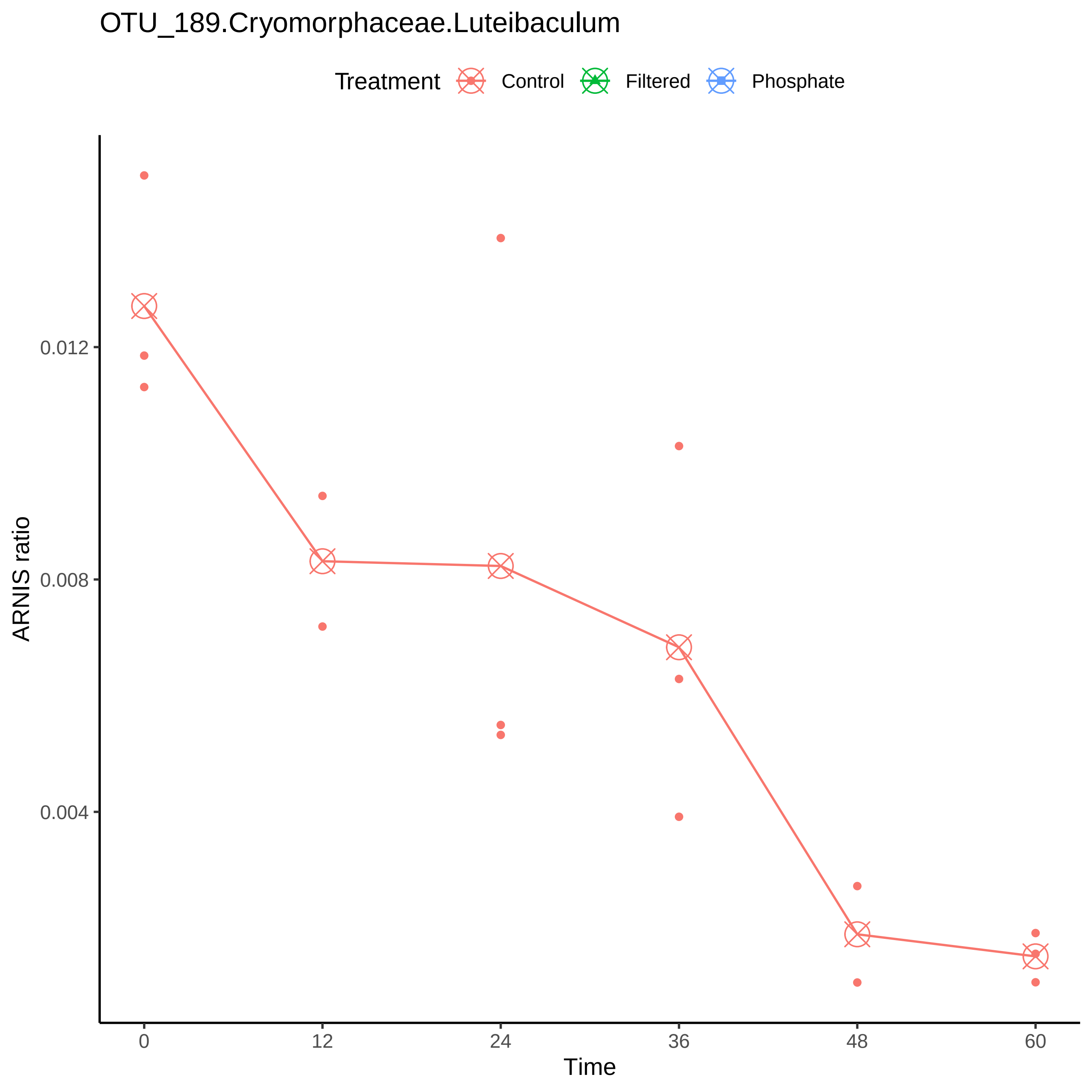
OTU_189.Cryomorphaceae.Luteibaculum
Treatment
Control
Filtered
Phosphate
ARNIS ratio
0.012
0.008
0.004
Time
0
12
24
36
48
60

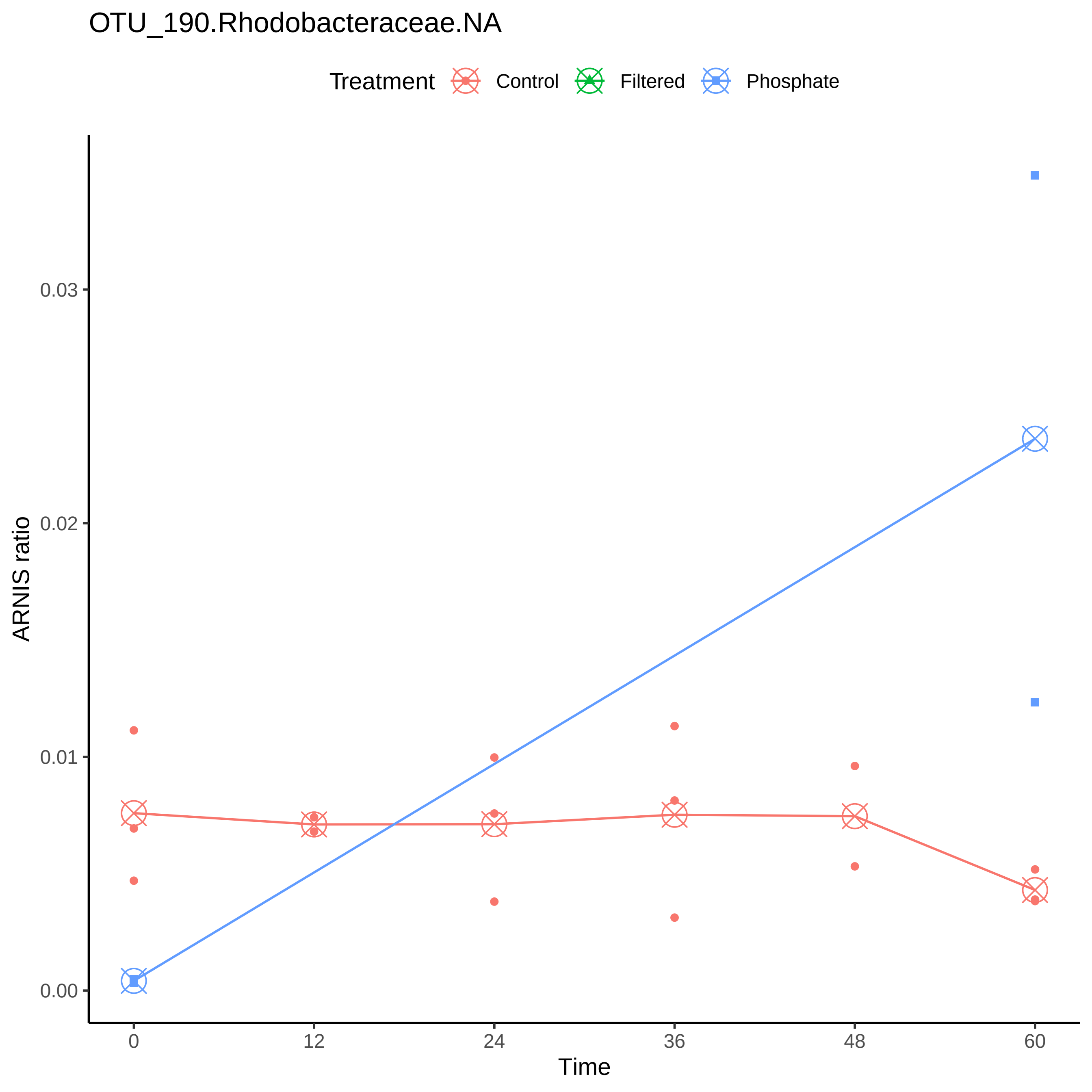

OTU_190.Rhodobacteraceae.NA
Treatment
Control
Filtered
Phosphate
ARNIS ratio
0.00
0.01
0.02
0.03
Time
0
12
24
36
48
60

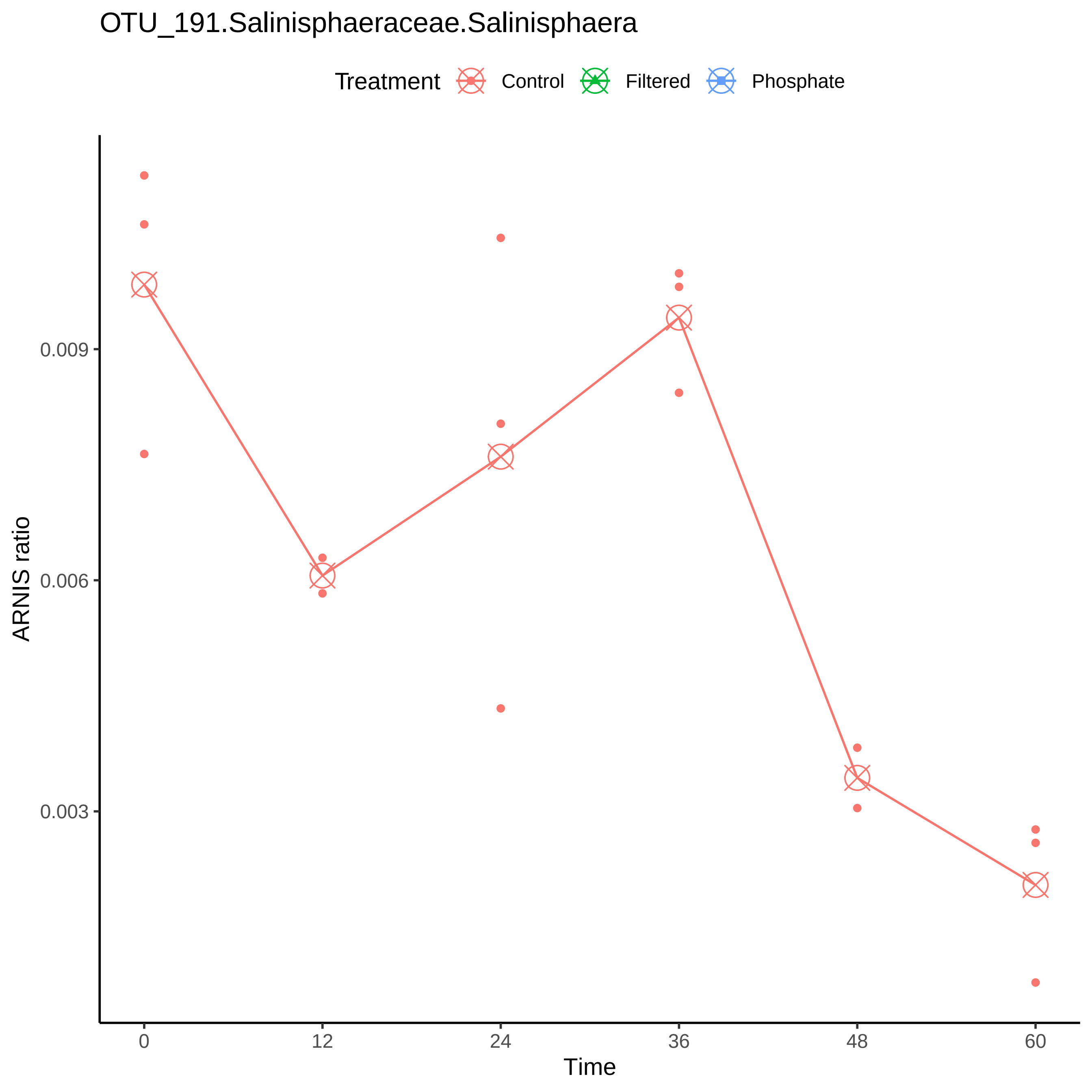
OTU_191.Salinisphaeraceae.Salinisphaera
Treatment
Control
Filtered
Phosphate
ARNIS ratio
0.009
0.006
0.003
Time
0
12
24
36
48
60

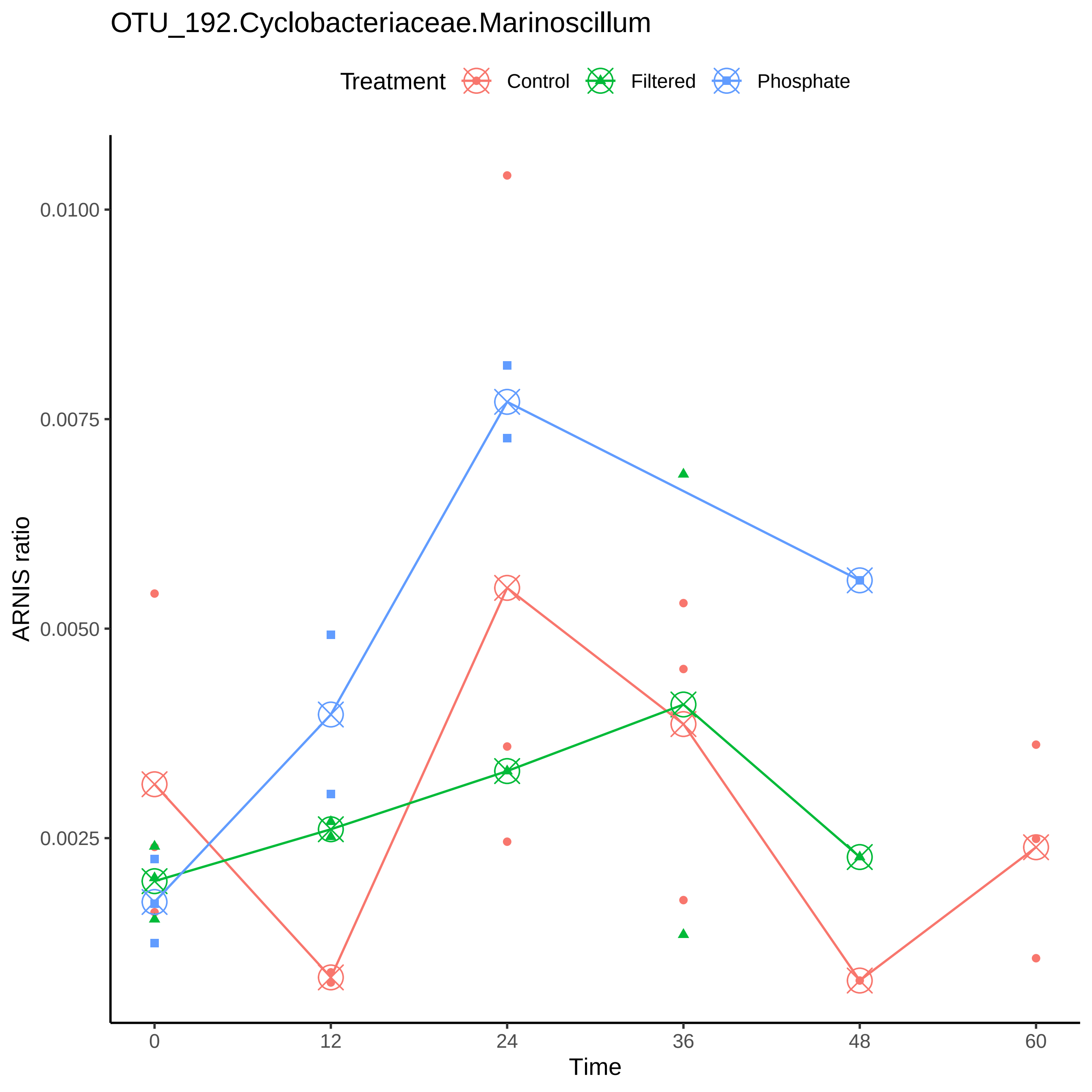
OTU_192.Cyclobacteriaceae.Marinoscillum
Treatment
Control
Filtered
Phosphate
ARNIS ratio
0.0100
0.0075
0.0050
0.0025
0
12
24
36
48
60
Time

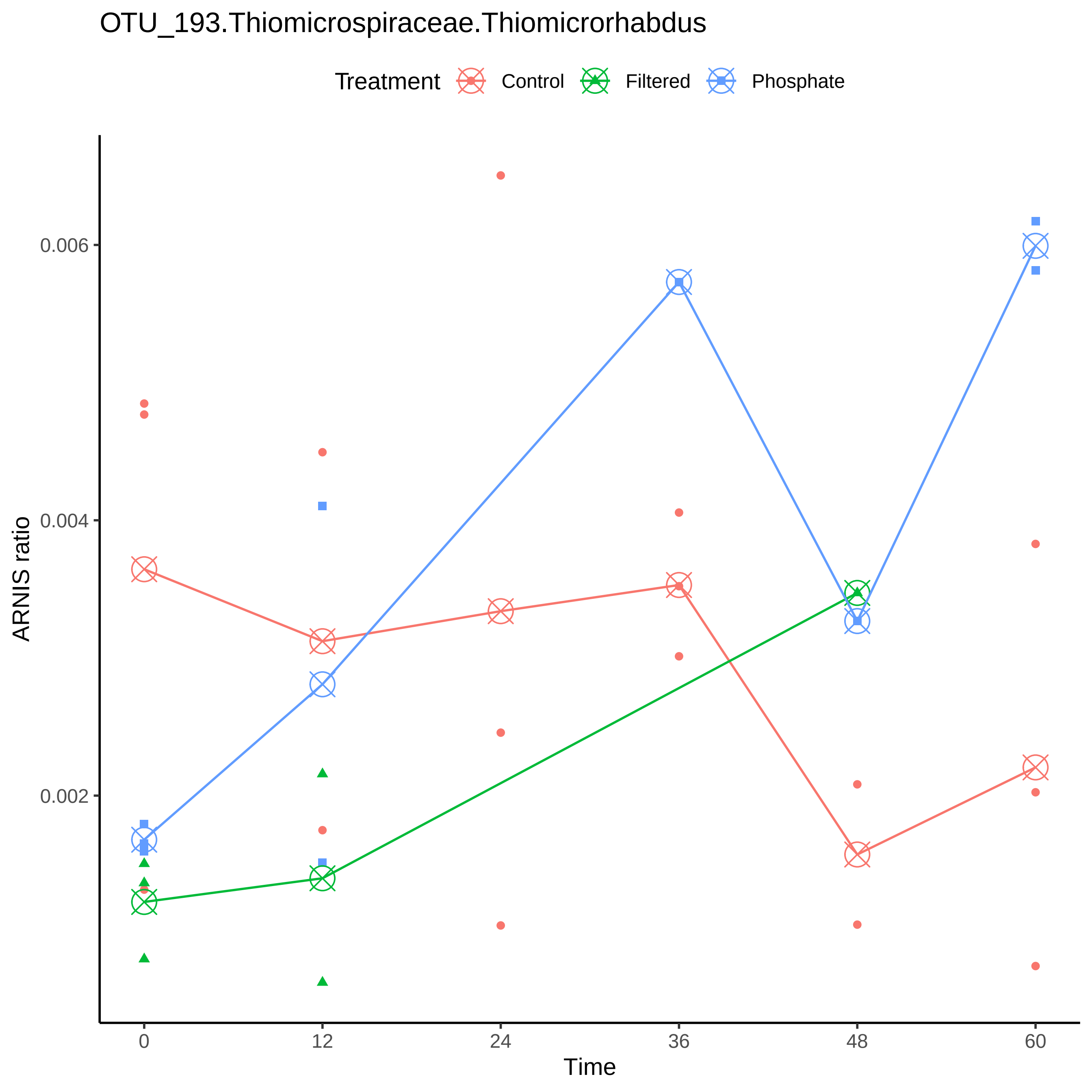

OTU_193.Thiomicrospiraceae.Thiomicrorhabdus
Treatment
Control
Filtered
Phosphate
0.006
0.004
0.002
ARNIS ratio
0
12
24
36
48
60
Time

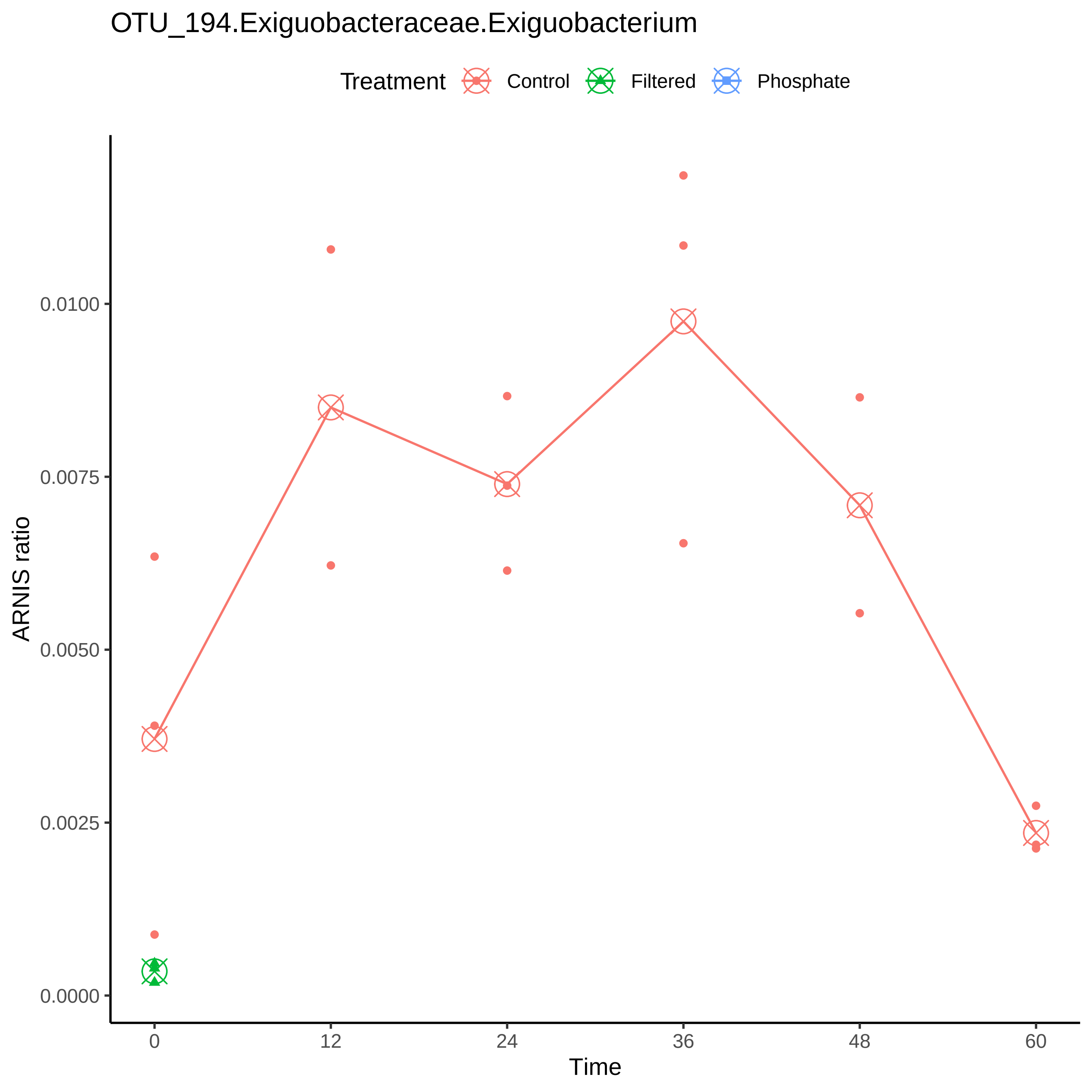

OTU_194.Exiguobacteraceae.Exiguobacterium
Treatment
Control
Filtered
Phosphate
ARNIS ratio
0.0100
0.0075
0.0050
0.0025
0.0000
0
12
24
36
48
60
Time

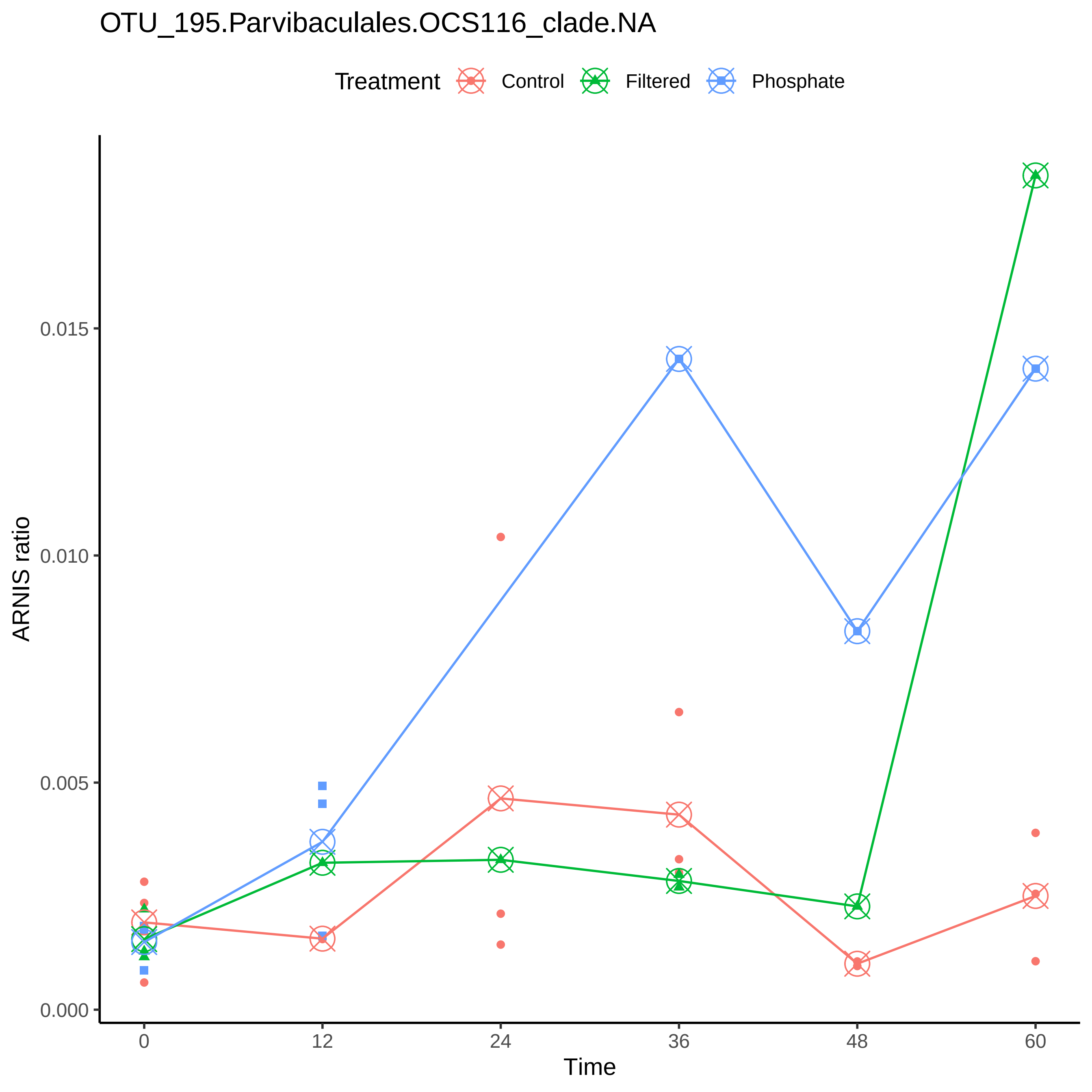
OTU_195.Parvibaculales.OCS116_clade.NA
Treatment
Control
Filtered
Phosphate
ARNIS ratio
0.000
0.005
0.010
0.015
0
12
24
36
48
60
Time

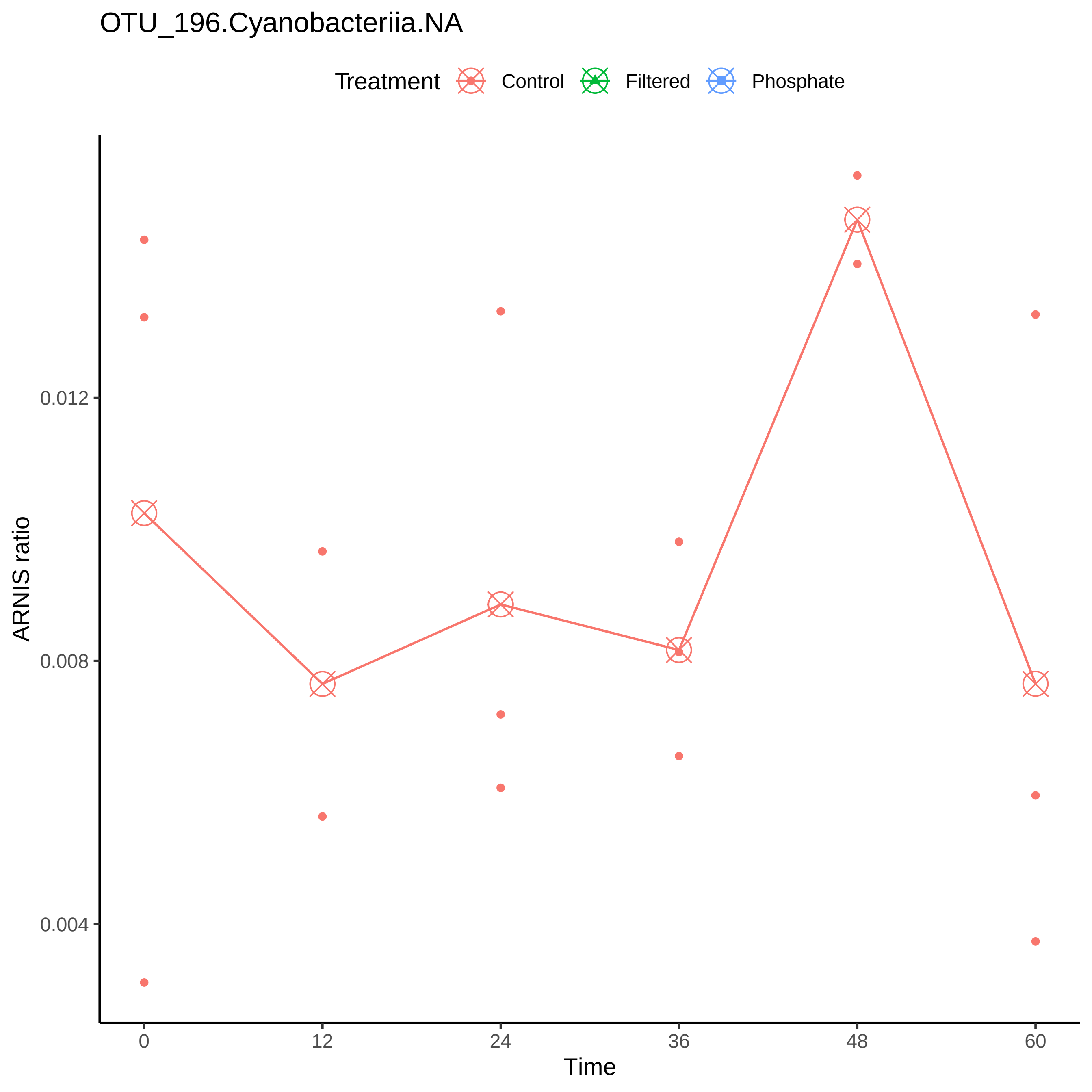
OTU_196.Cyanobacteriia.NA
Treatment
Control
Filtered
Phosphate
ARNIS ratio
0.012
0.008
0.004
Time
0
12
24
36
48
60

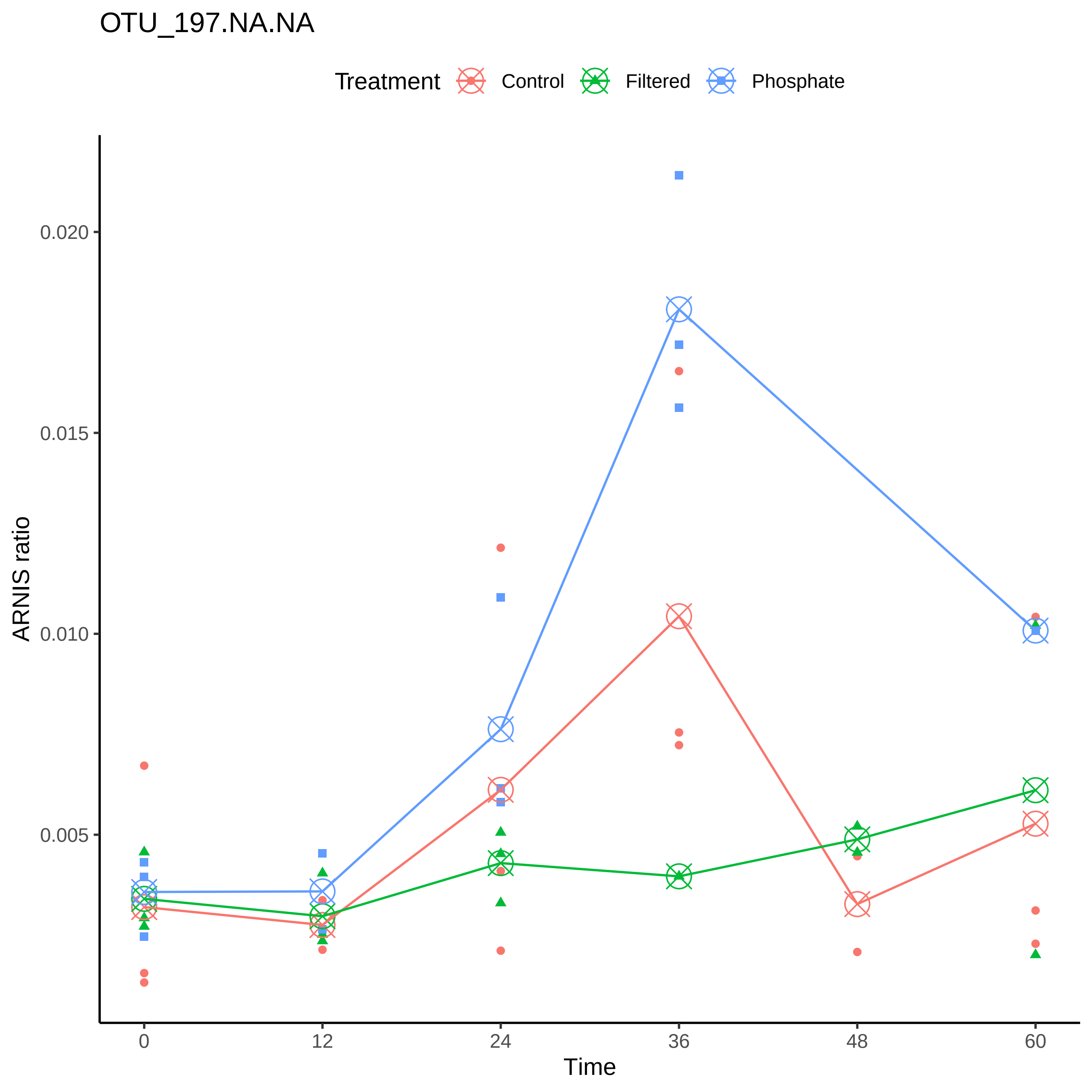

OTU_197.NA.NA
Treatment
Control
Filtered
Phosphate
ARNIS ratio
0.020
0.015
0.010
0.005
0
12
24
36
48
60
Time

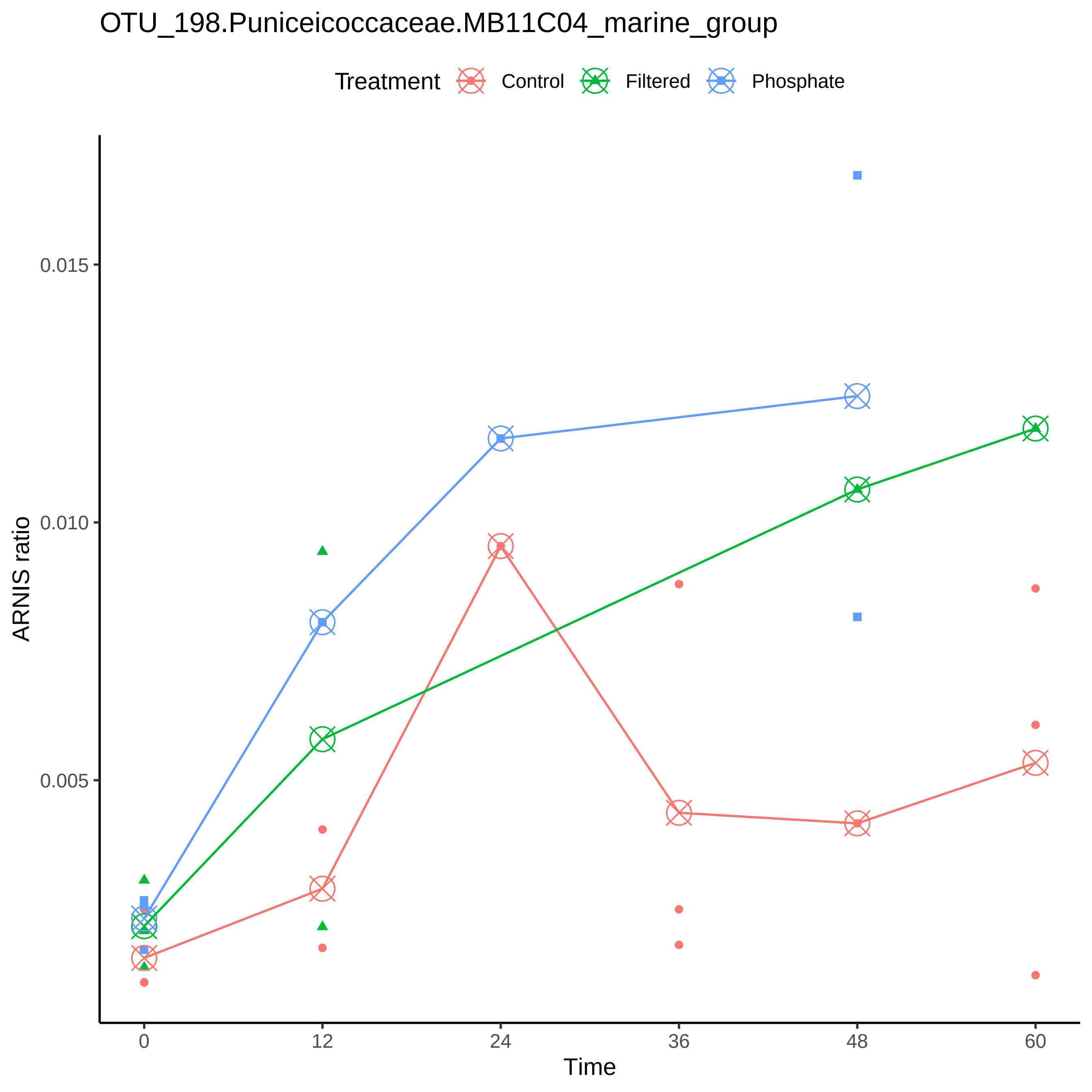

OTU_198.Puniceicoccaceae.MB11C04_marine_group
Treatment
Control
Filtered
Phosphate
ARNIS ratio
0.015
0.010
0.005
0
12
24
36
48
60
Time

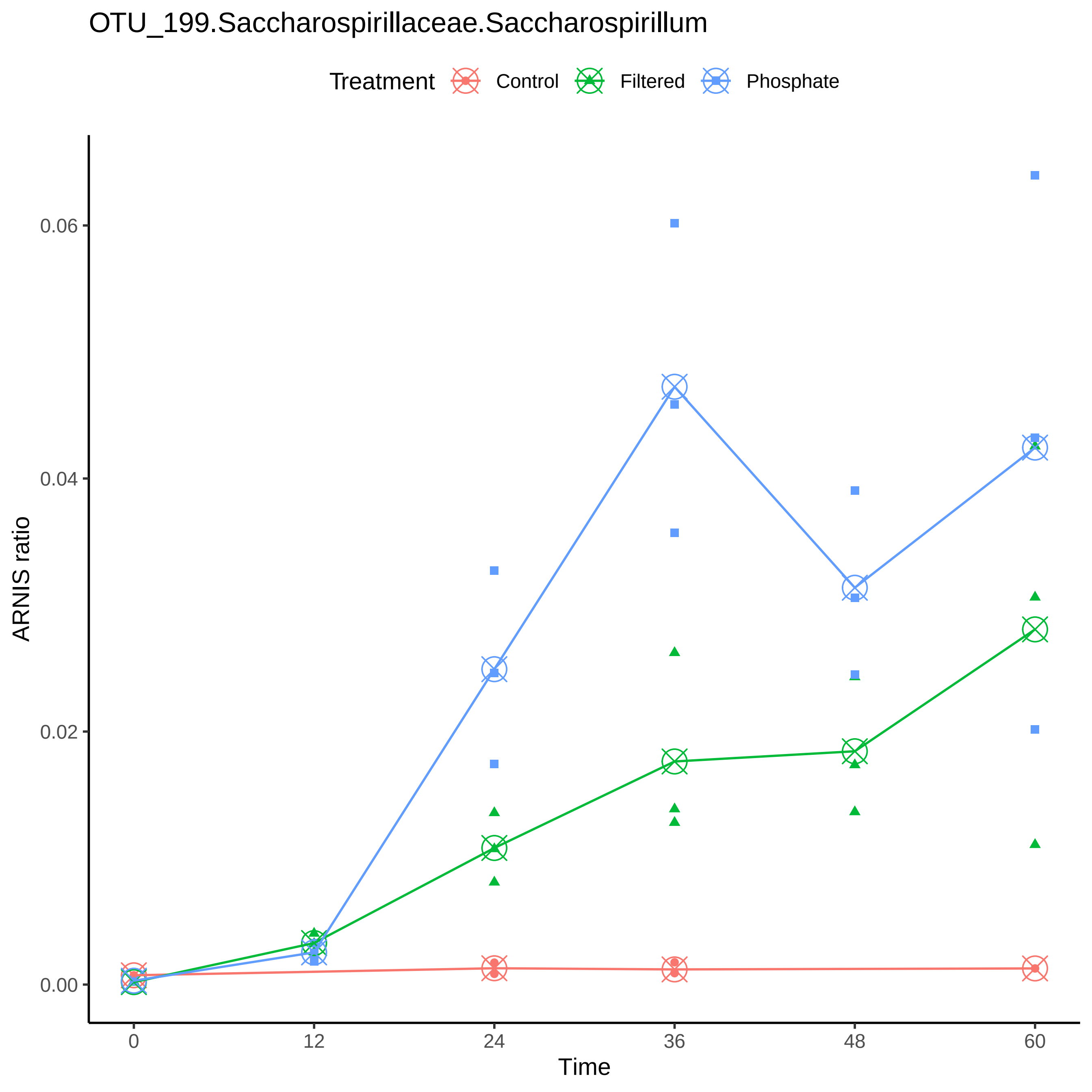
OTU_199.Saccharospirillaceae.Saccharospirillum
Treatment
Control
Filtered
Phosphate
ARNIS ratio
0.00
0.02
0.04
0.06
Time
0
12
24
36
48
60

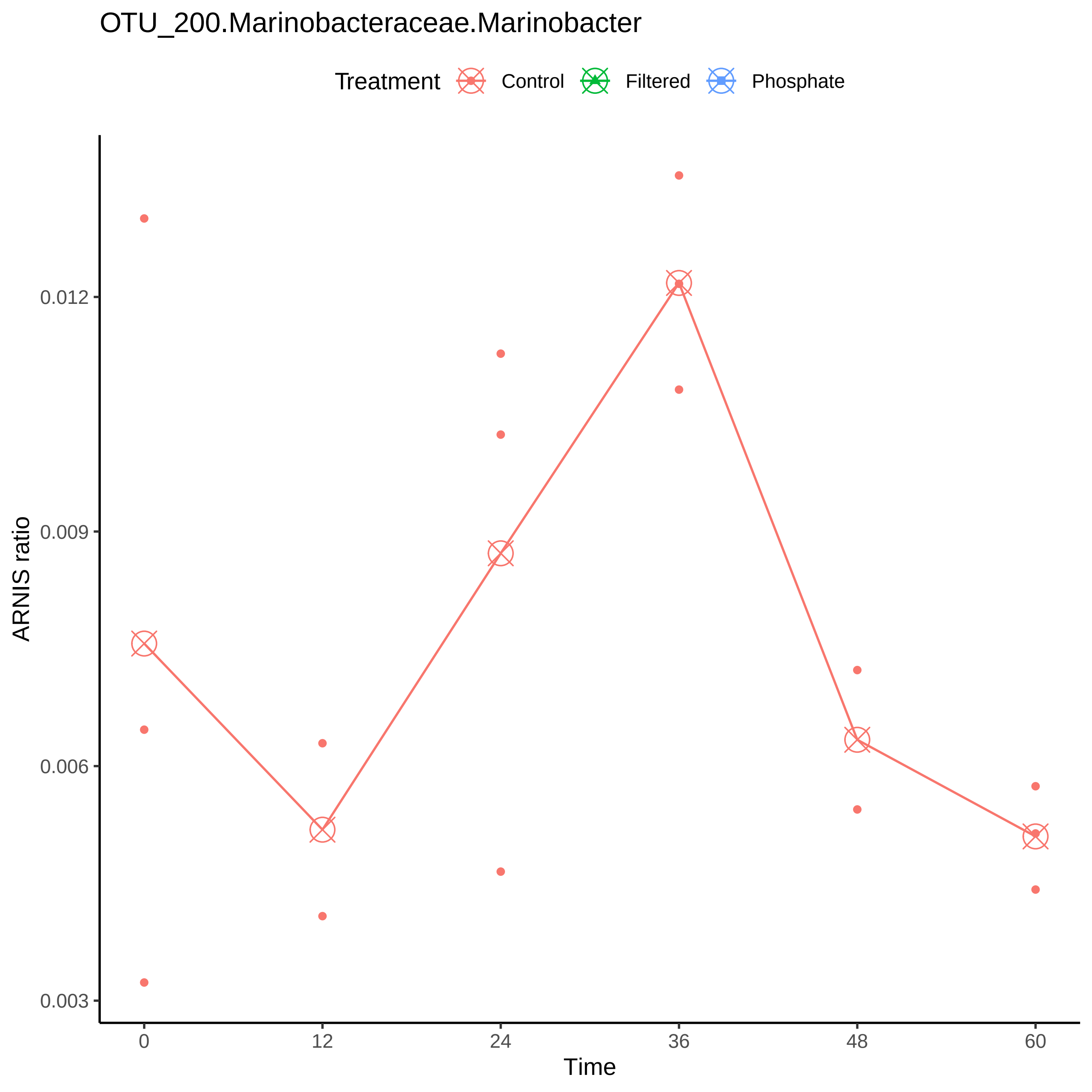

OTU_200.Marinobacteraceae.Marinobacter
Treatment
Control
Filtered
Phosphate
ARNIS ratio
0.012
0.009
0.006
0.003
0
12
24
36
48
60
Time

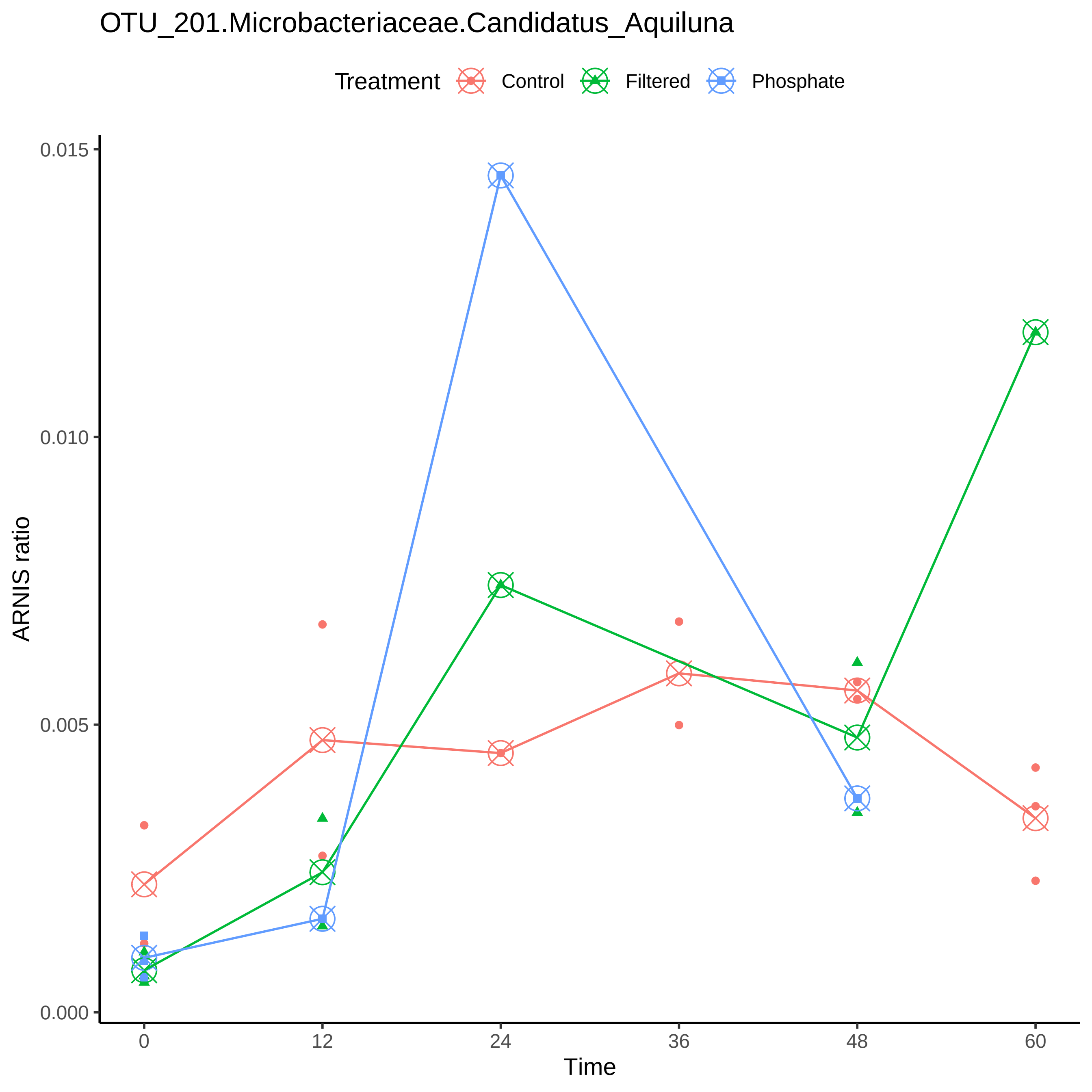
OTU_201.Microbacteriaceae.Candidatus_Aquiluna
Treatment
Control
Filtered
Phosphate
0.015
0.010
0.005
0.000
ARNIS ratio
0
12
24
36
48
60
Time

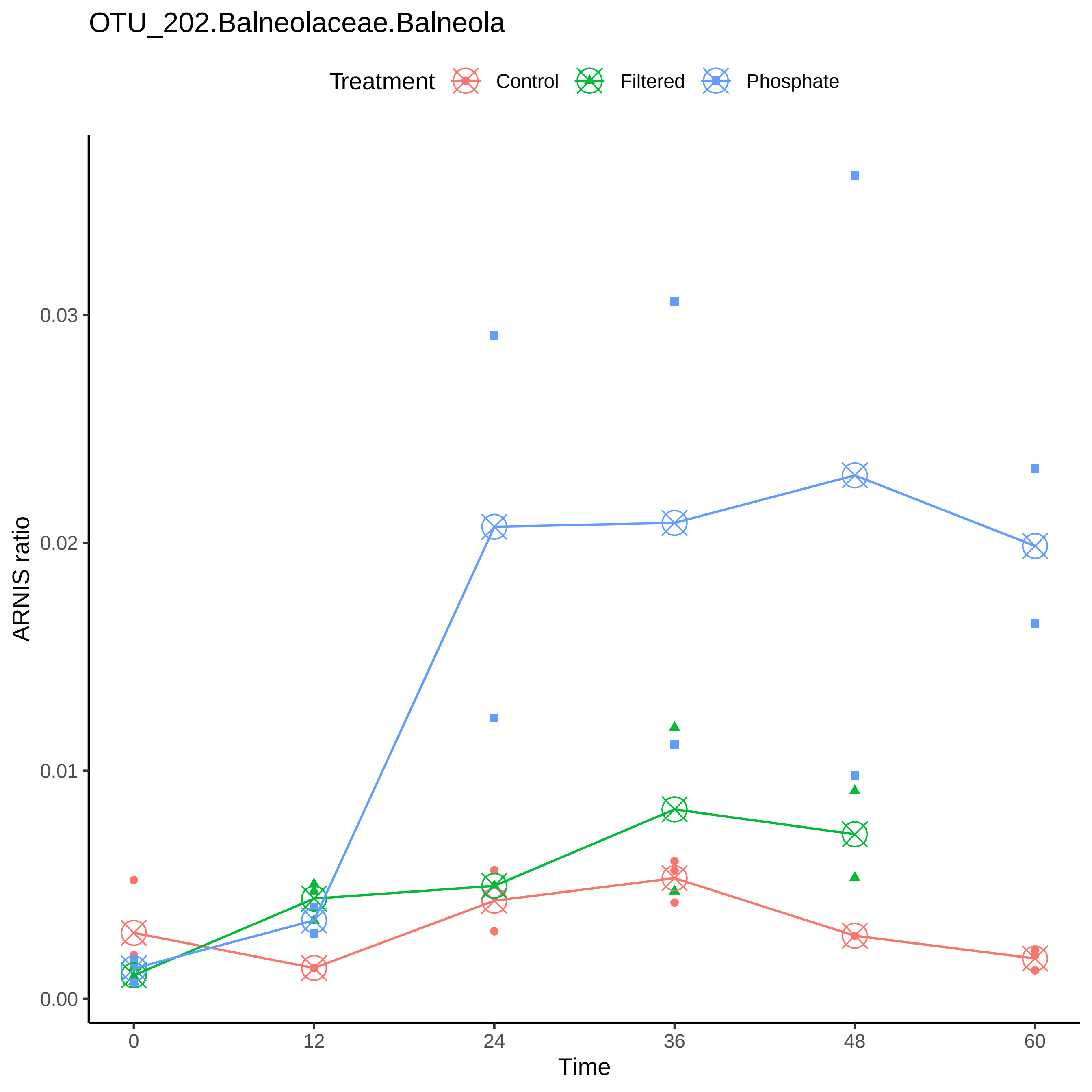

OTU_202.Balneolaceae.Balneola
Treatment
Control
Filtered
Phosphate
ARNIS ratio
0.00
0.01
0.02
0.03
0
12
24
36
48
60
Time

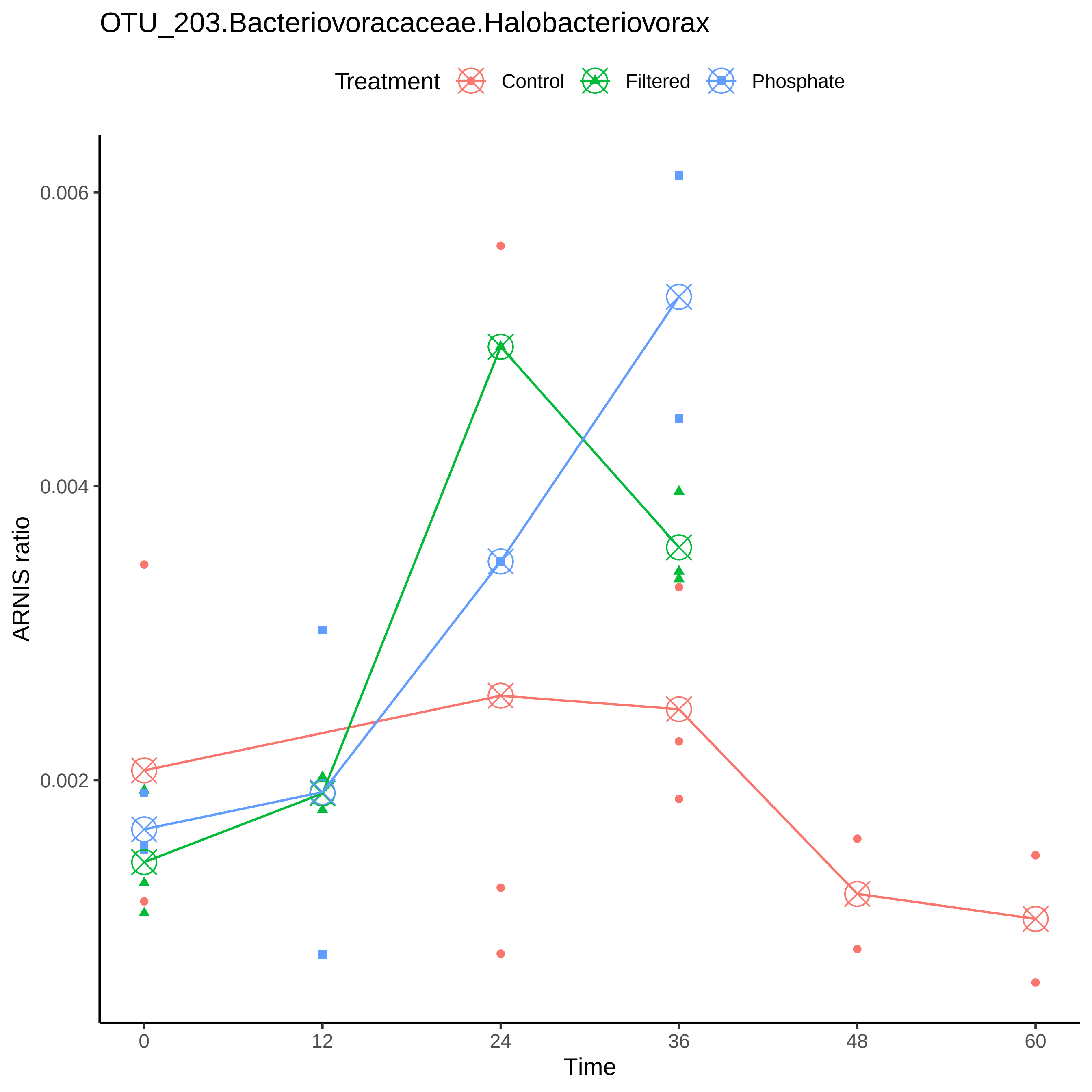

OTU_203.Bacteriovoracaceae.Halobacteriovorax
Treatment
Control
Filtered
Phosphate
0.006
0.004
0.002
ARNIS ratio
0
12
24
36
48
60
Time

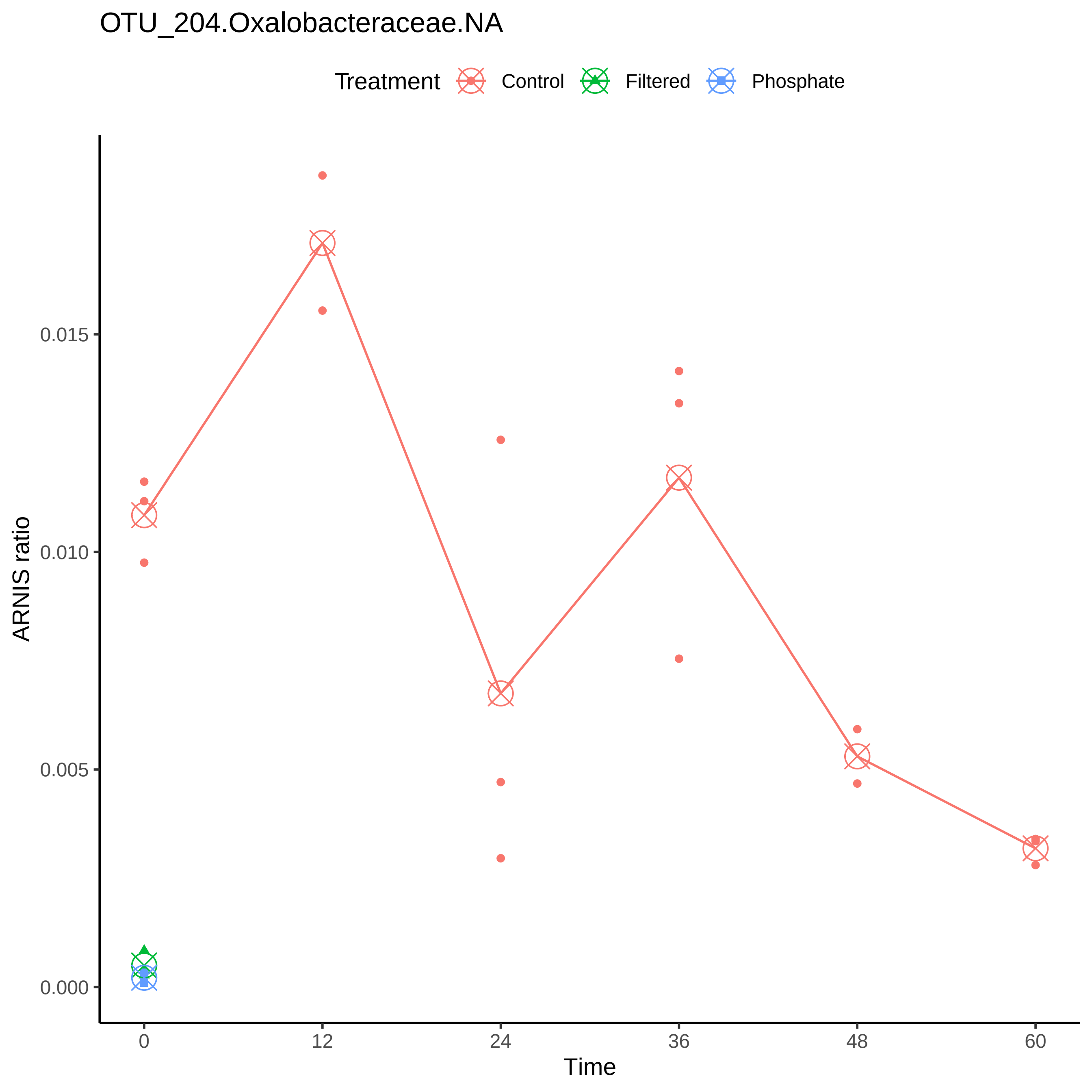

OTU_204.Oxalobacteraceae.NA
Treatment
Control
Filtered
Phosphate
ARNIS ratio
0.015
0.010
0.005
0.000
0
12
24
36
48
60
Time

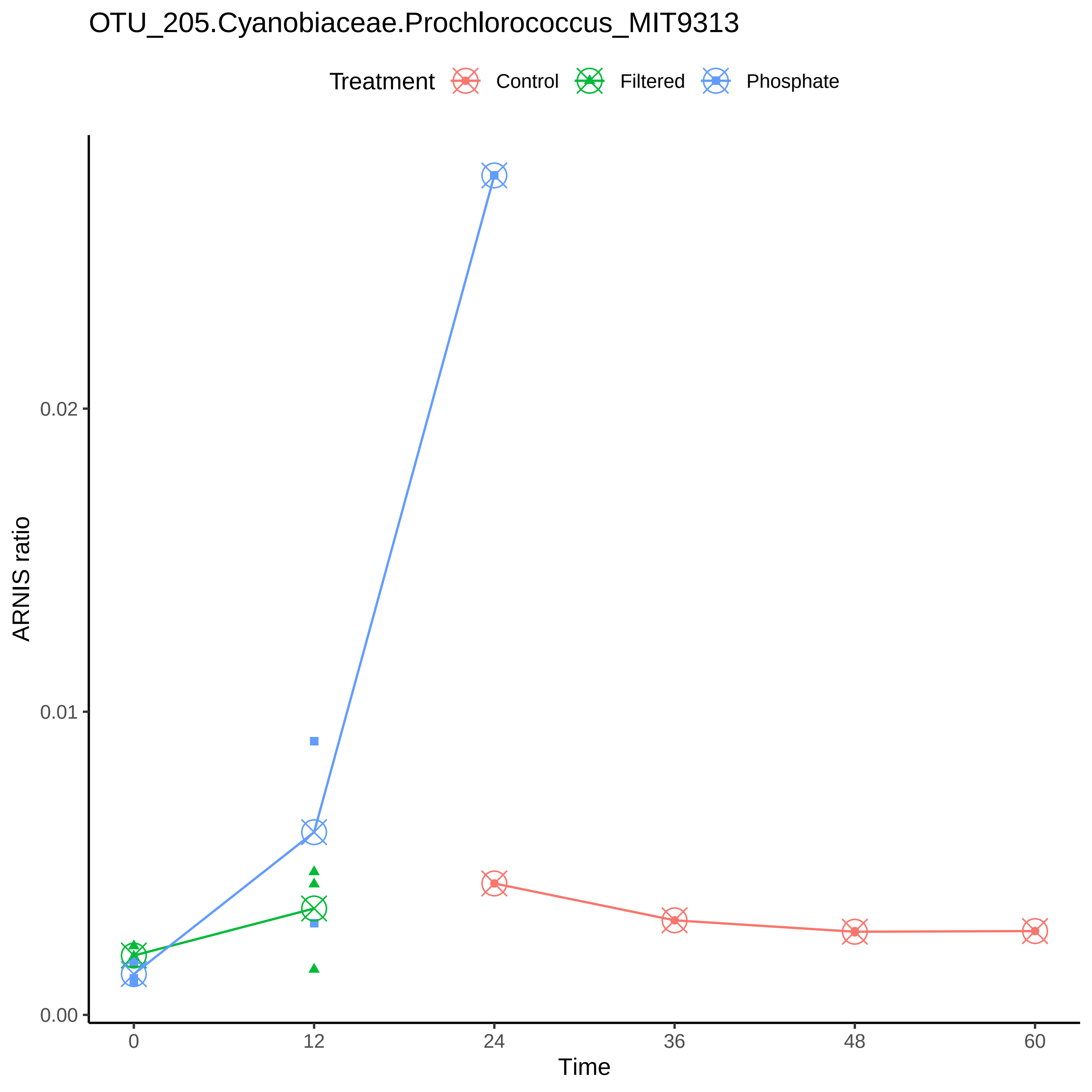

OTU_205.Cyanobiaceae.Prochlorococcus_MIT9313
Treatment
Control
Filtered
Phosphate
ARNIS ratio
0.00
0.01
0.02
Time
0
12
24
36
48
60

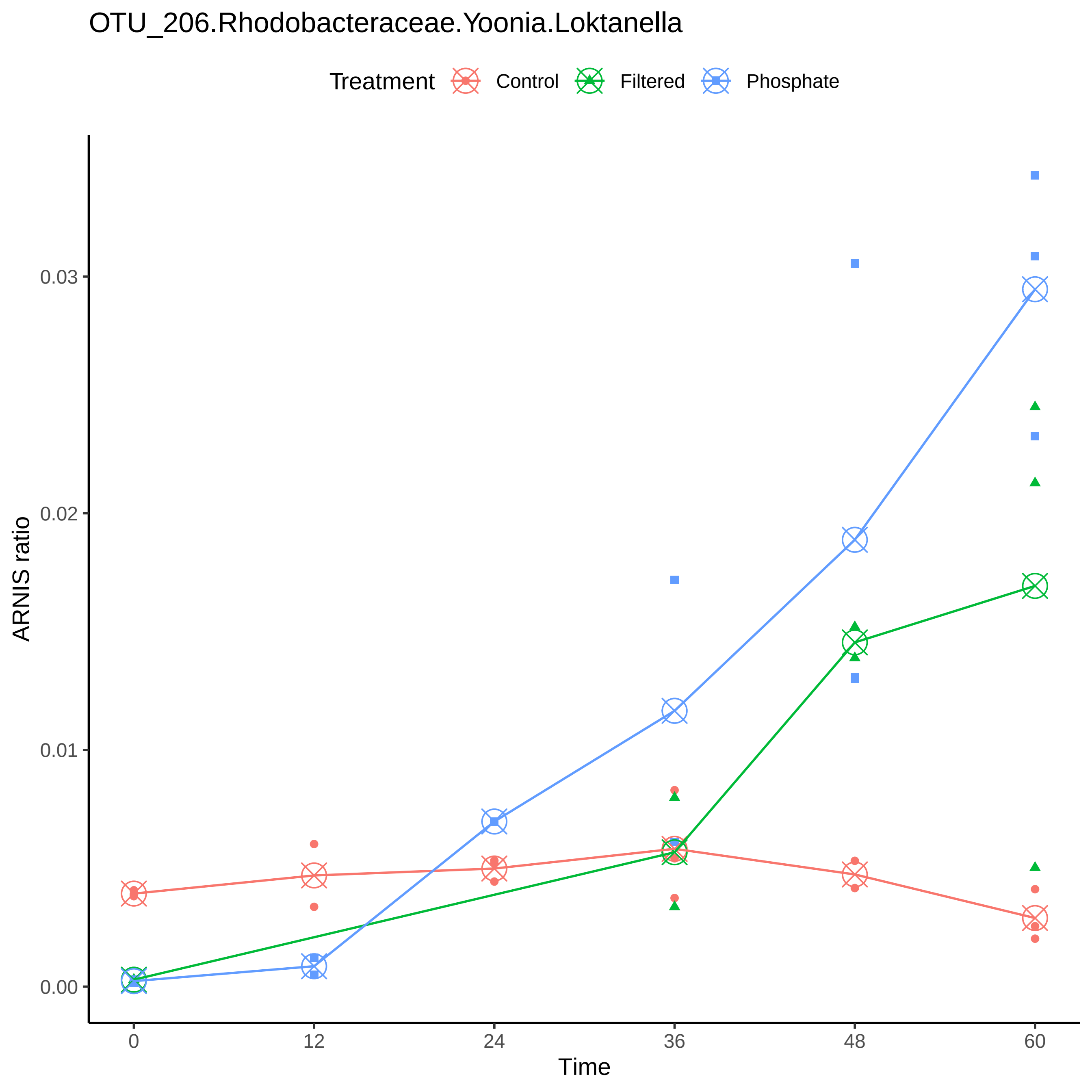

OTU_206.Rhodobacteraceae.Yoonia.Loktanella
Treatment
Control
Filtered
Phosphate
ARNIS ratio
0.00
0.01
0.02
0.03
Time
0
12
24
36
48
60

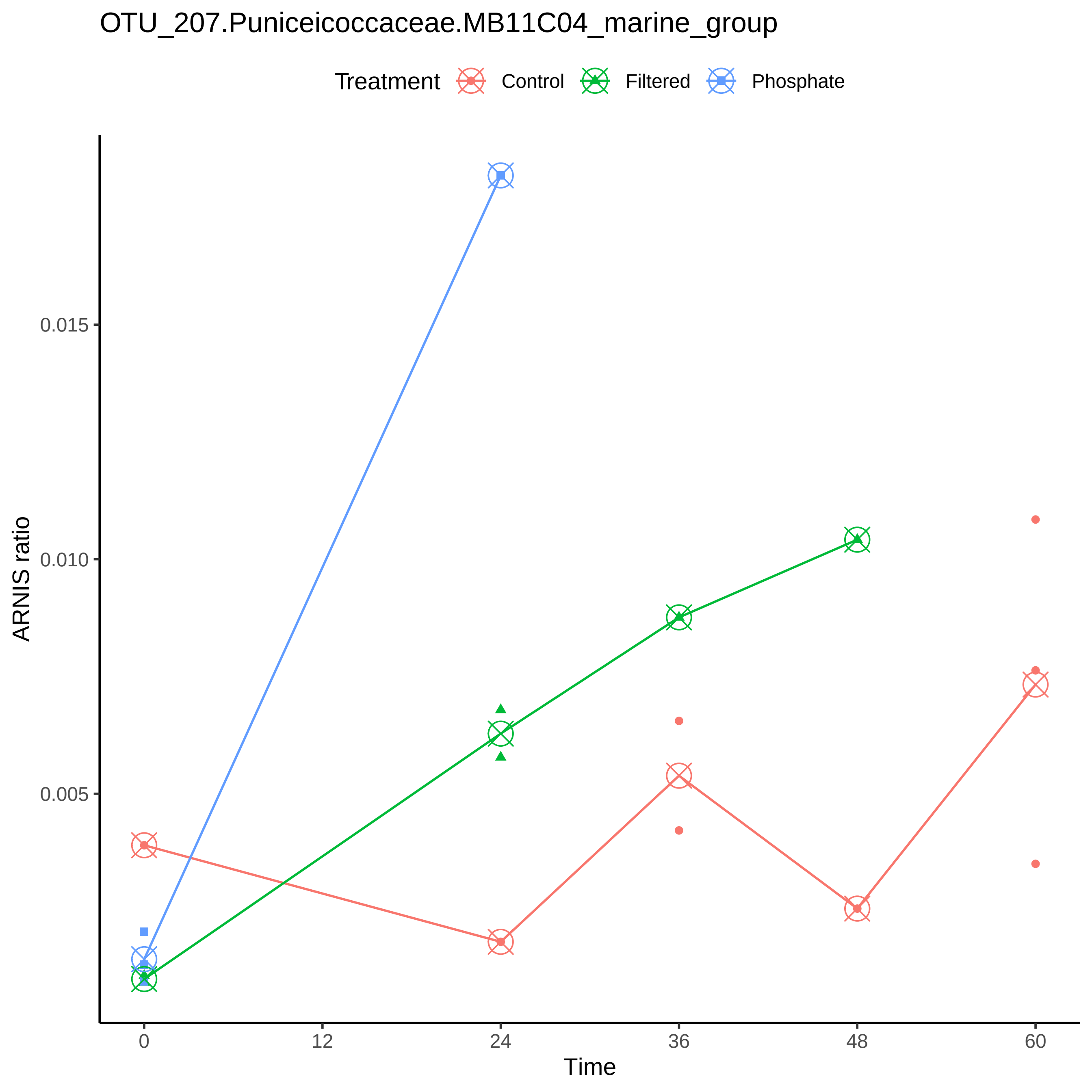

OTU_207.Puniceicoccaceae.MB11C04_marine_group
Treatment
Control
Filtered
Phosphate
ARNIS ratio
0.015
0.010
0.005
0
12
24
36
48
60
Time

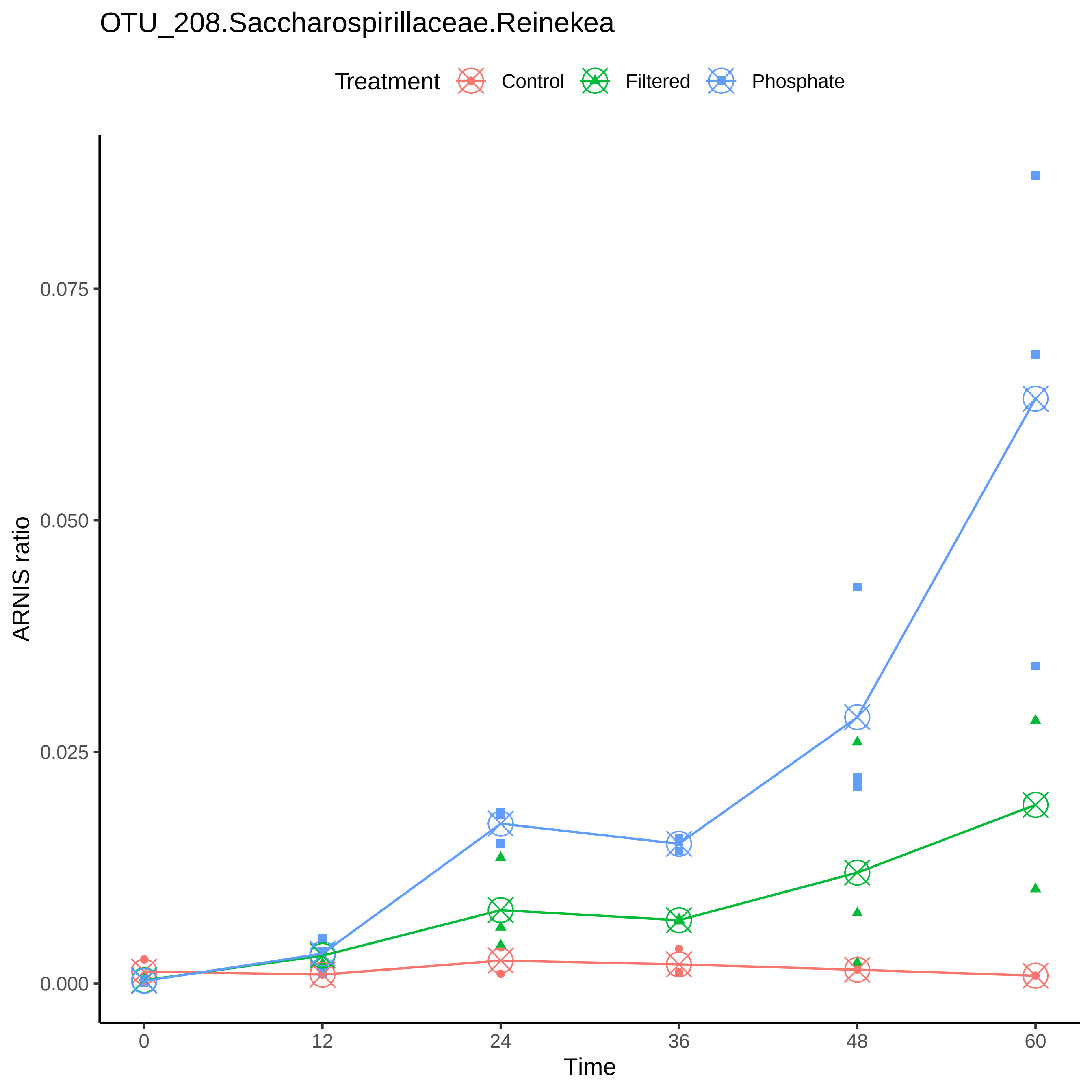
OTU_208.Saccharospirillaceae.Reinekea
Treatment
Control
Filtered
Phosphate
ARNIS ratio
0.000
0.025
0.050
0.075
Time
0
12
24
36
48
60

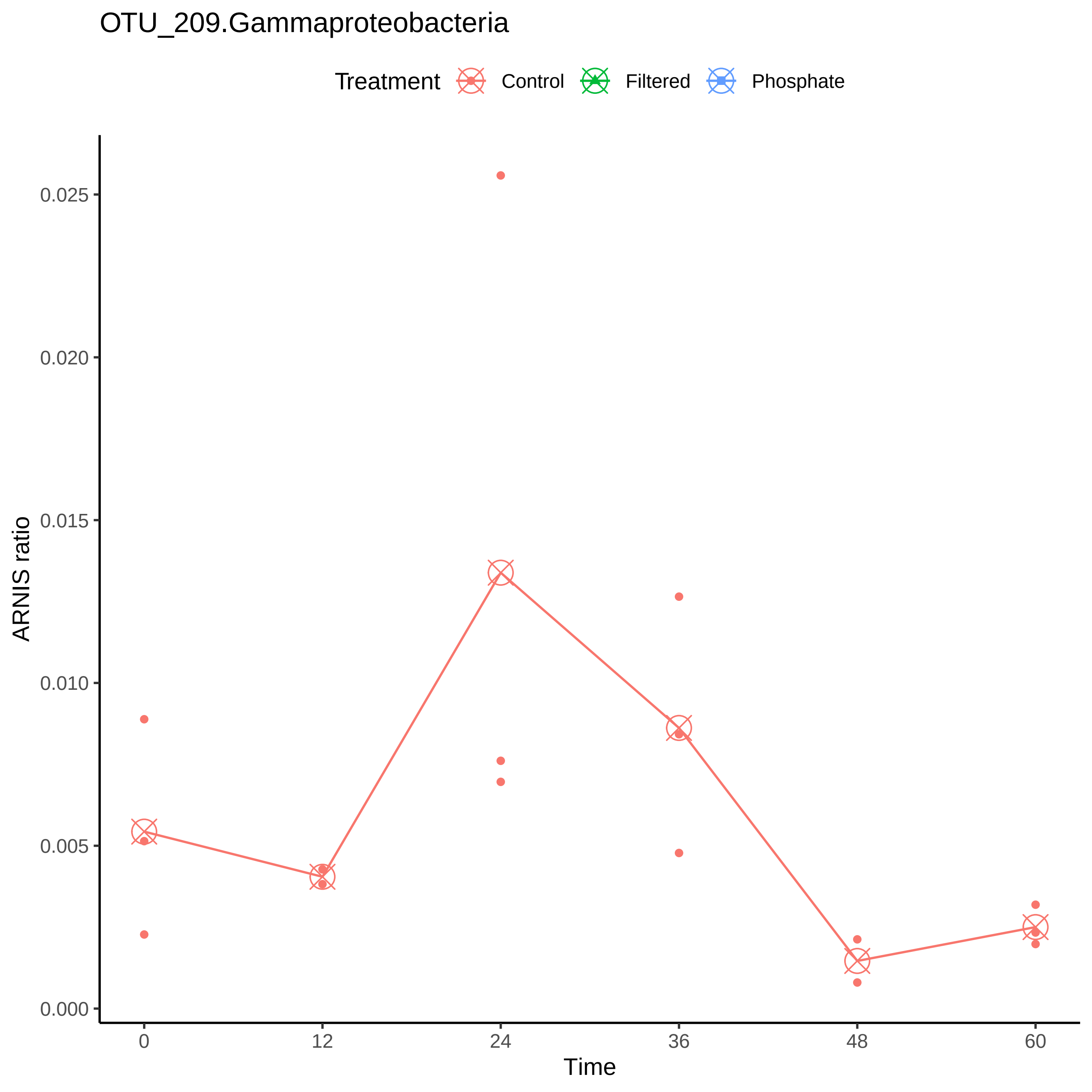
OTU_209.Gammaproteobacteria
Treatment
Control
Filtered
Phosphate
ARNIS ratio
0.000
0.005
0.010
0.015
0.020
0.025
Time
0
12
24
36
48
60

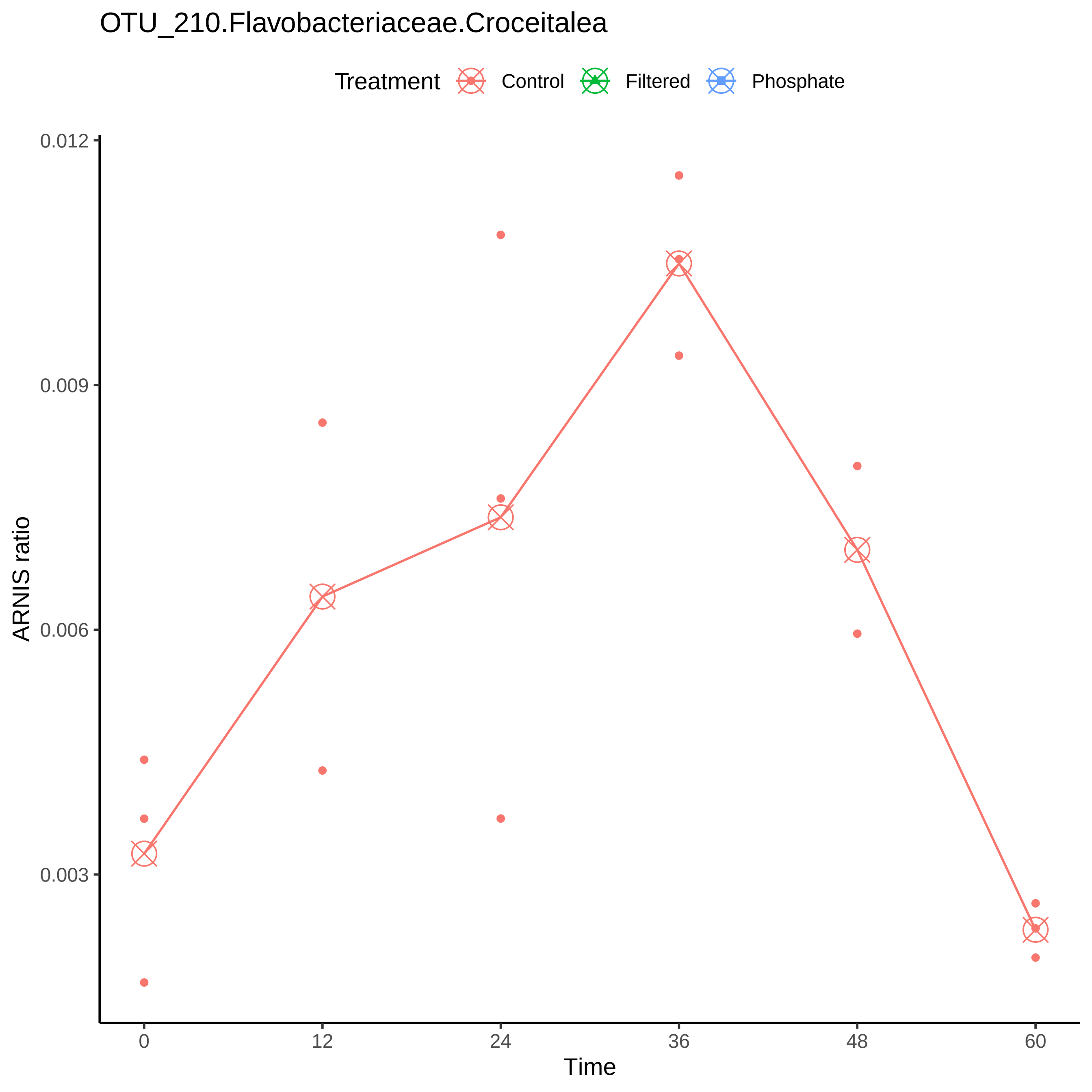

OTU_210.Flavobacteriaceae.Croceitalea
Treatment
Control
Filtered
Phosphate
0.012
0.009
0.006
0.003
ARNIS ratio
0
12
24
36
48
60
Time

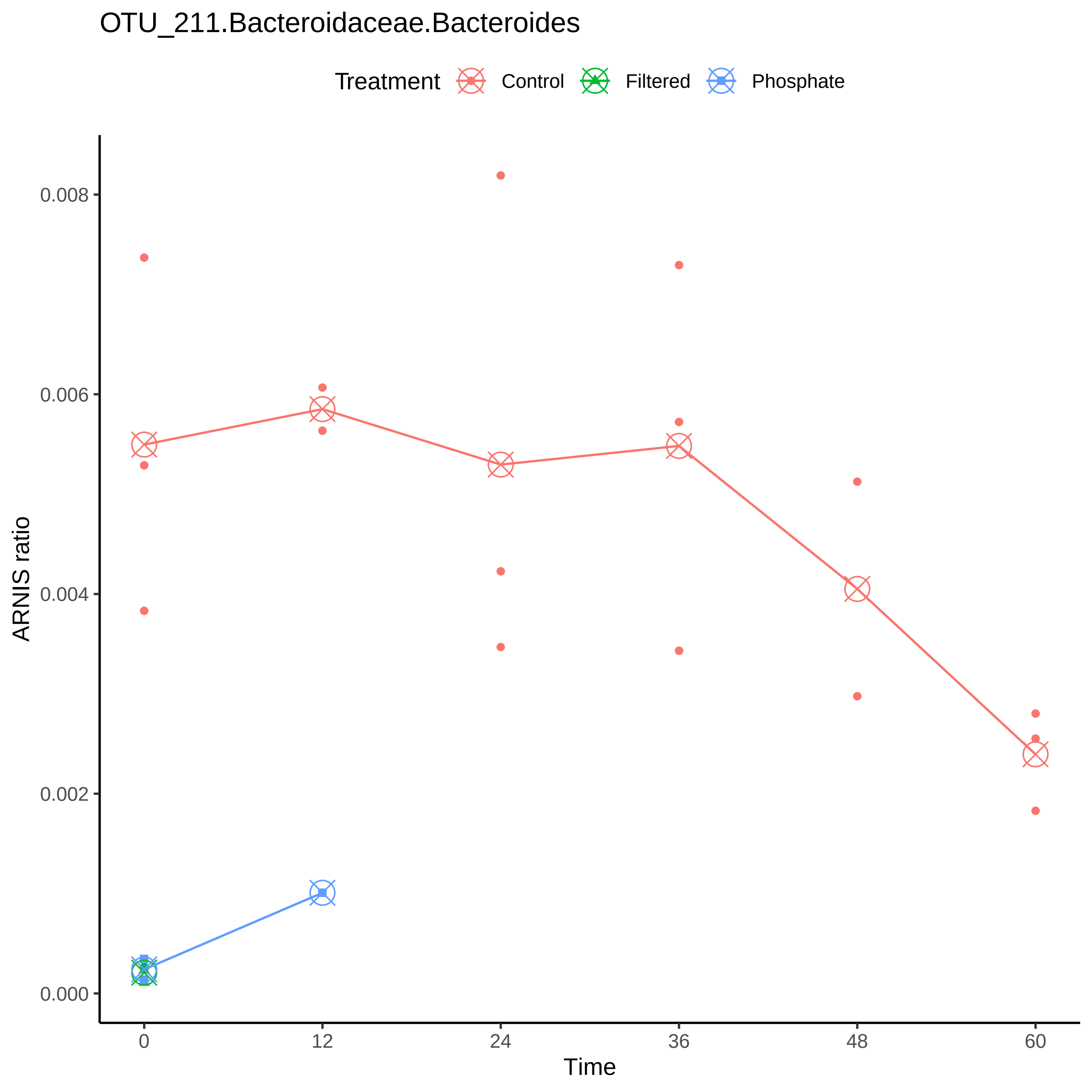
OTU_211.Bacteroidaceae.Bacteroides
Treatment
Control
Filtered
Phosphate
ARNIS ratio
0.008
0.006
0.004
0.002
0.000
Time
0
12
24
36
48
60

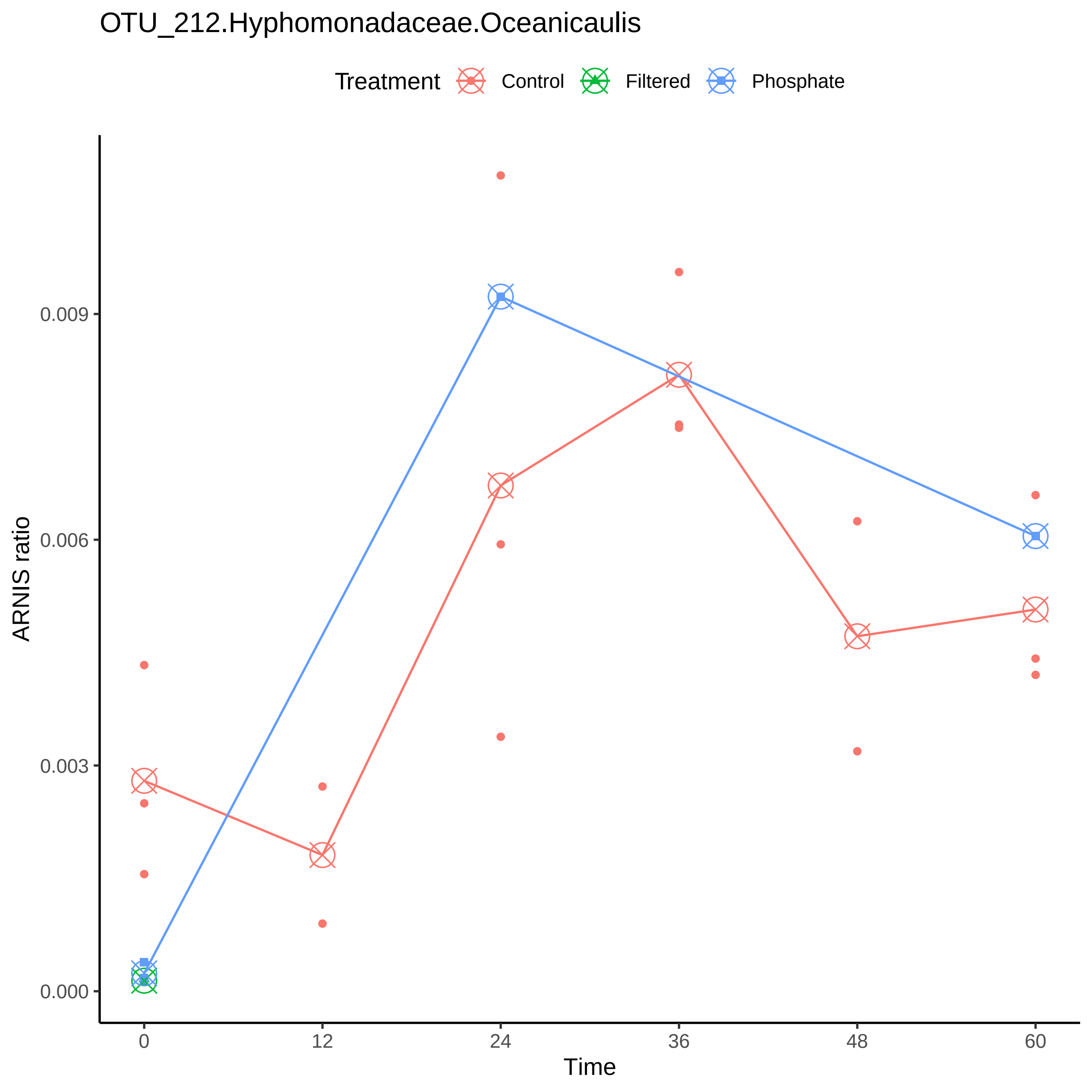
OTU_212.Hyphomonadaceae.Oceanicaulis
Treatment
Control
Filtered
Phosphate
ARNIS ratio
0.000
0.003
0.006
0.009
Time
0
12
24
36
48
60

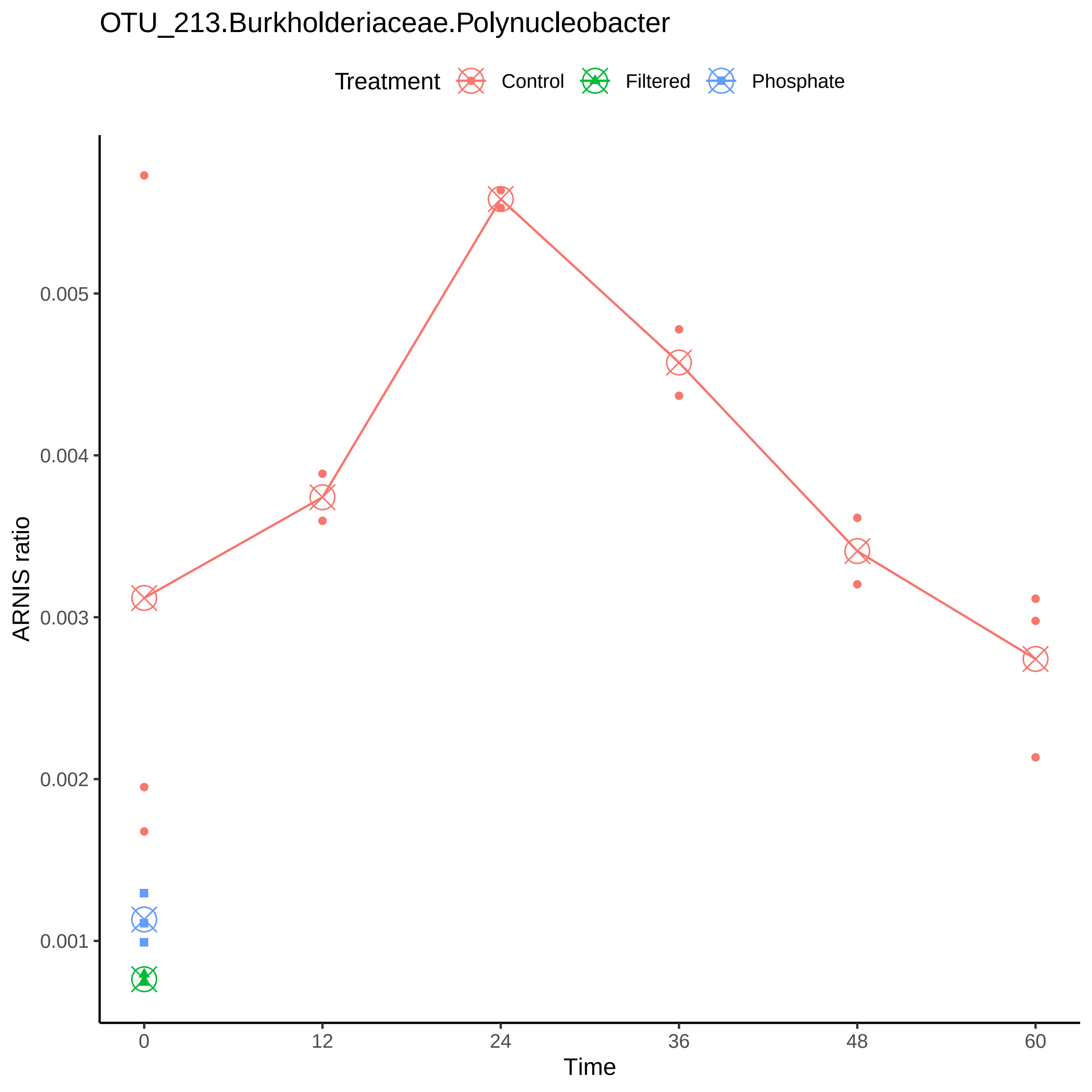

OTU_213.Burkholderiaceae.Polynucleobacter
Treatment
Control
Filtered
Phosphate
ARNIS ratio
0.005
0.004
0.003
0.002
0.001
0
12
24
36
48
60
Time

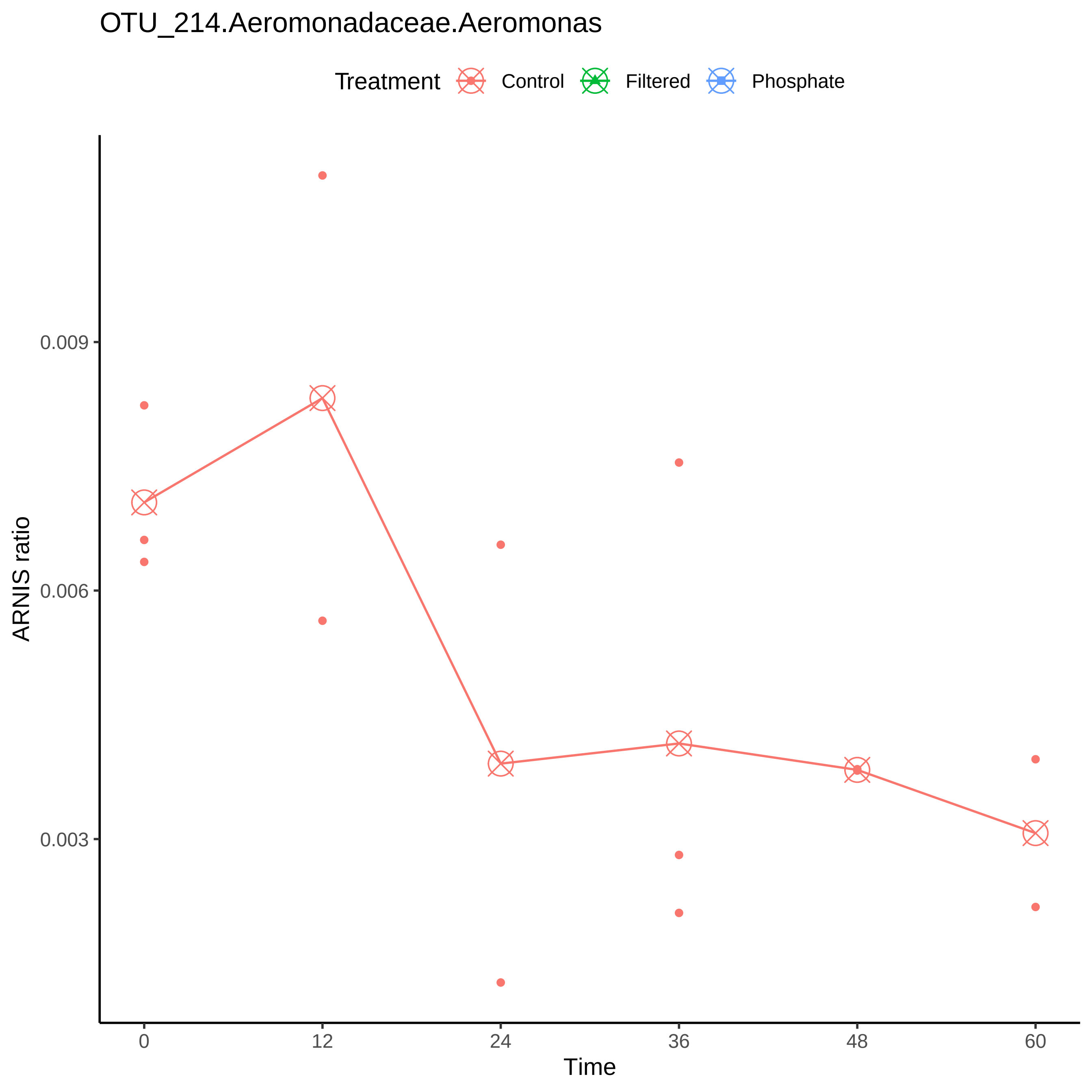
OTU_214.Aeromonadaceae.Aeromonas
Treatment
Control
Filtered
Phosphate
ARNIS ratio
0.009
0.006
0.003
Time
0
12
24
36
48
60

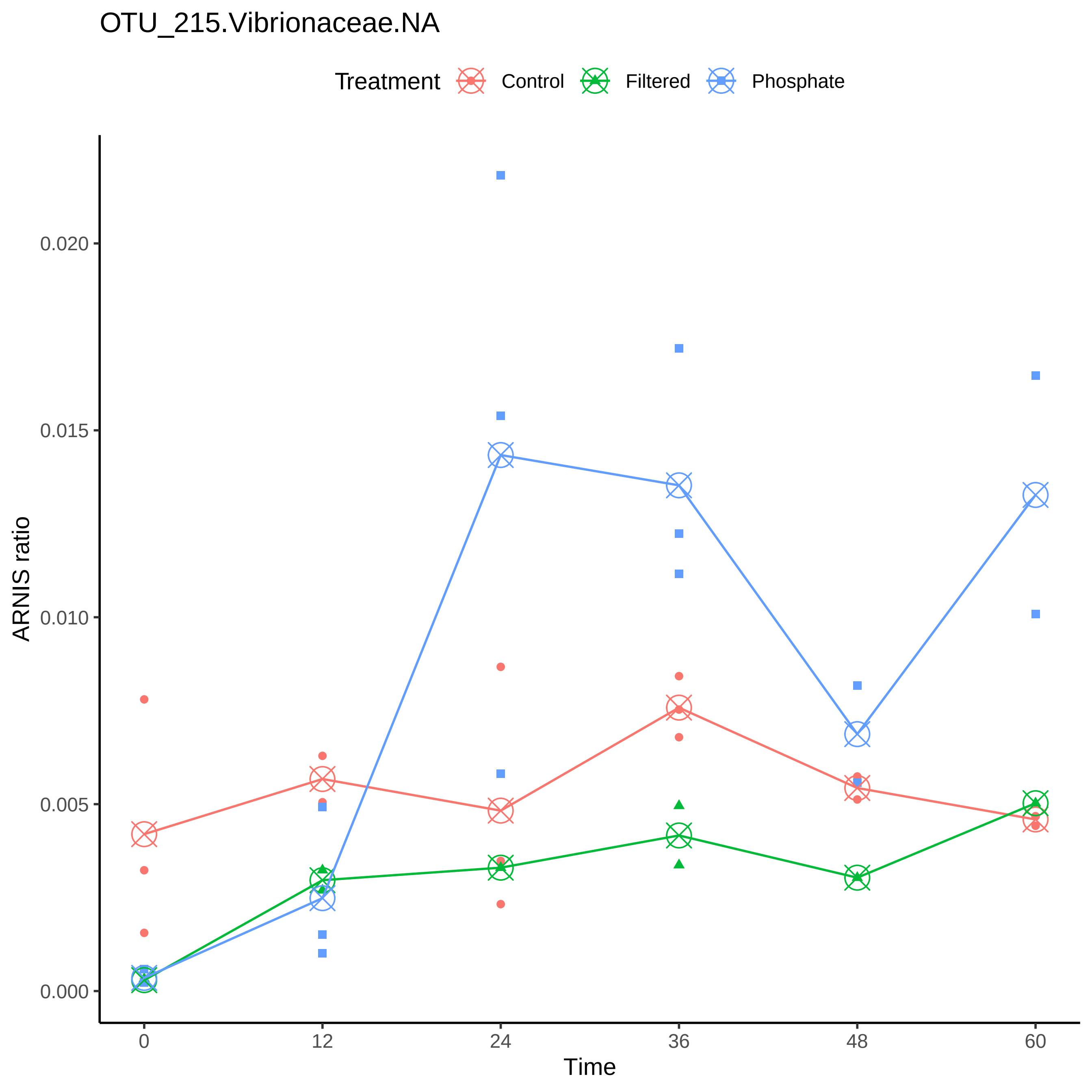

OTU_215.Vibrionaceae.NA
Treatment
Control
Filtered
Phosphate
ARNIS ratio
0.000
0.005
0.010
0.015
0.020
0
12
24
36
48
60
Time

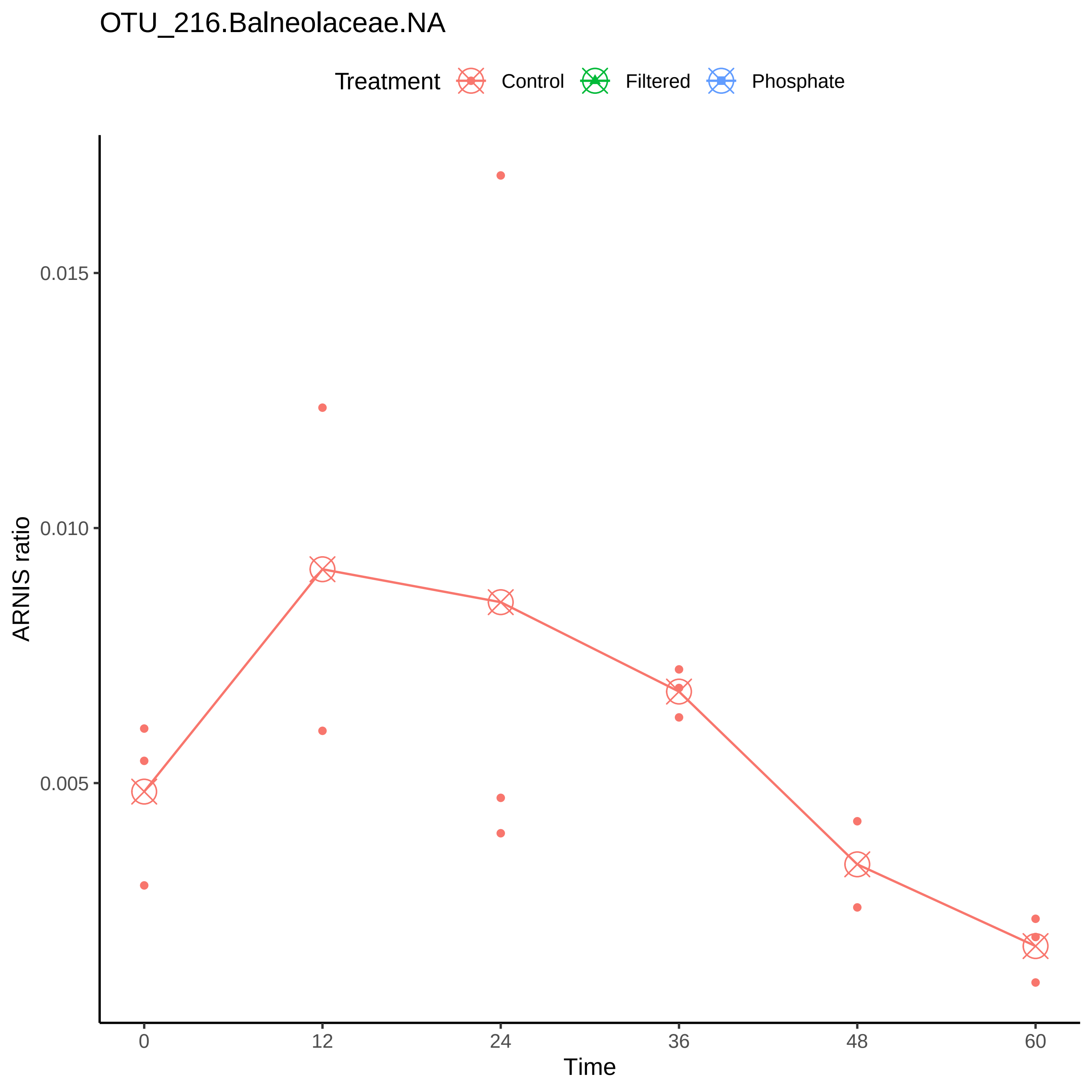

OTU_216.Balneolaceae.NA
Treatment
Control
Filtered
Phosphate
ARNIS ratio
0.015
0.010
0.005
0
12
24
36
48
60
Time

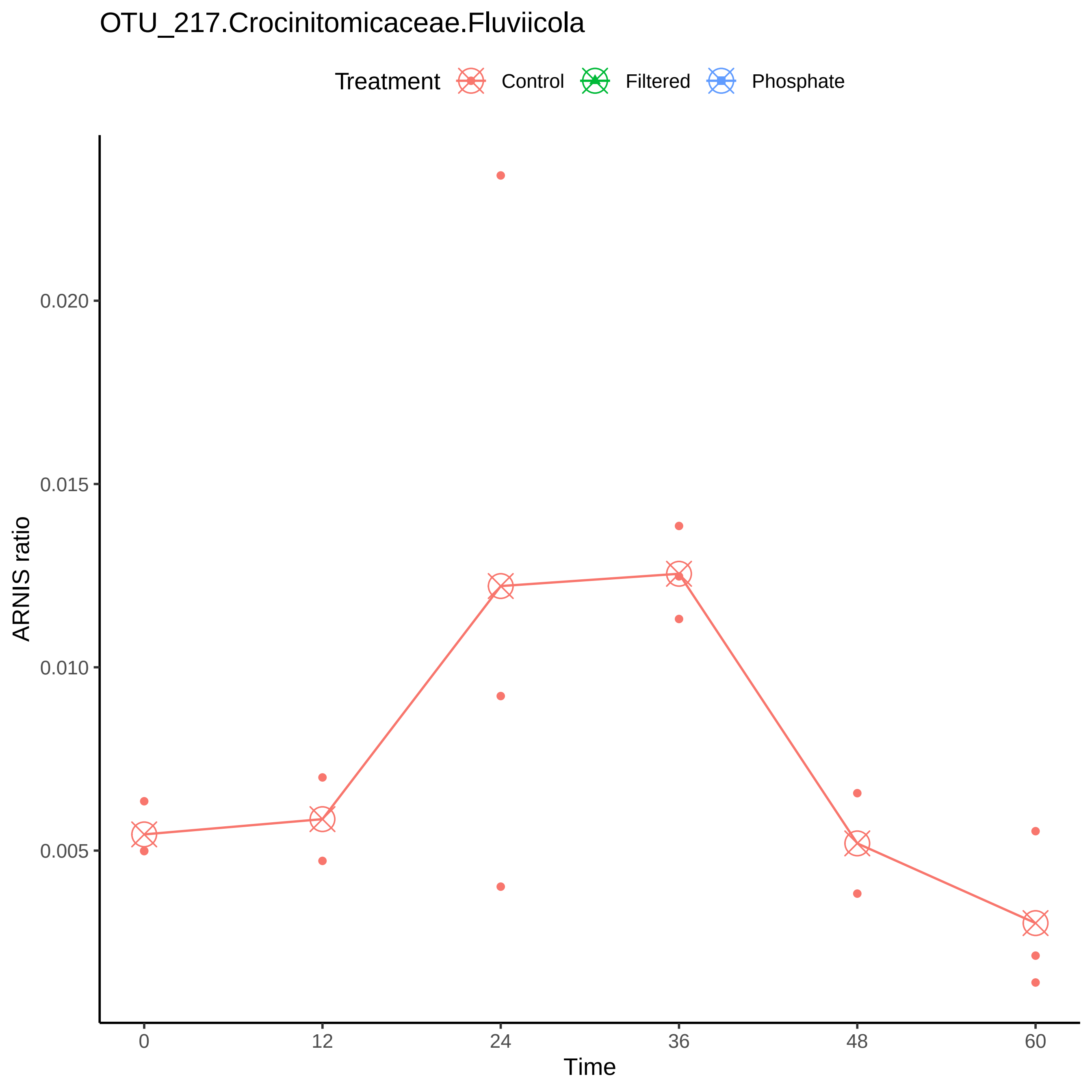
OTU_217.Crocinitomicaceae.Fluviicola
Treatment
Control
Filtered
Phosphate
ARNIS ratio
0.020
0.015
0.010
0.005
0
12
24
36
48
60
Time

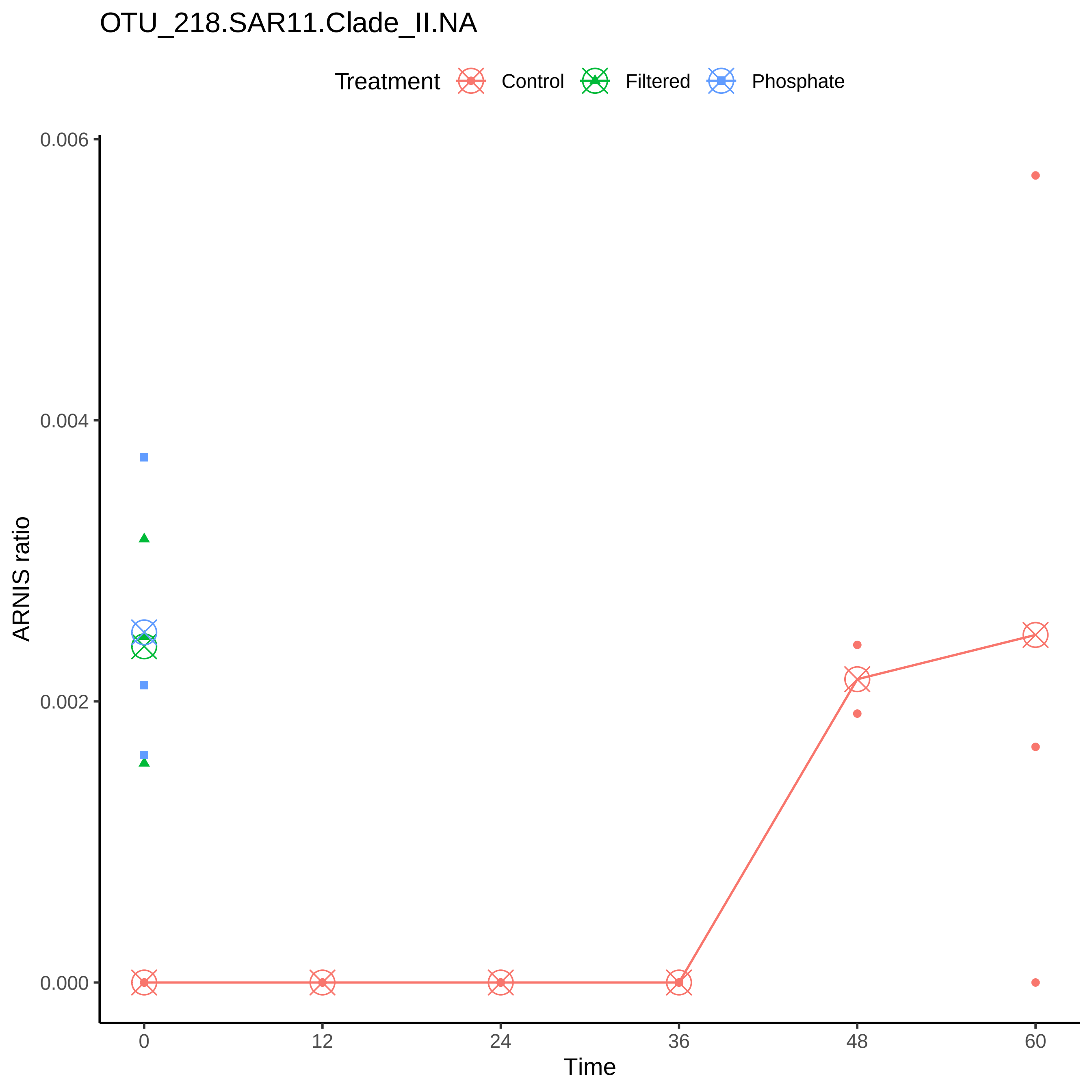

OTU_218.SAR11.Clade_II.NA
Treatment
Control
Filtered
Phosphate
ARNIS ratio
0.006
0.004
0.002
0.000
0
12
24
36
48
60
Time

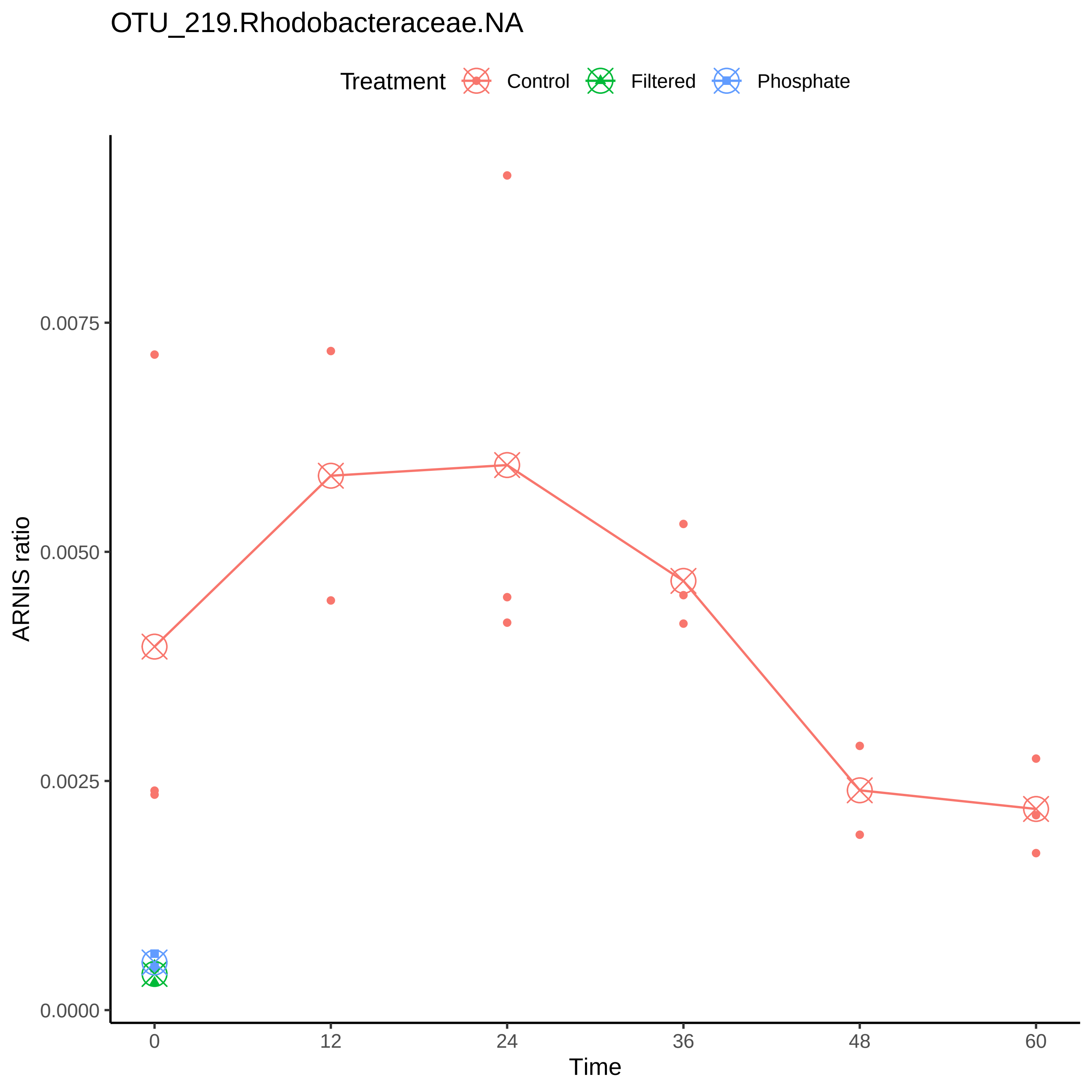

OTU_219.Rhodobacteraceae.NA
Treatment
Control
Filtered
Phosphate
ARNIS ratio
0.0000
0.0025
0.0050
0.0075
Time
0
12
24
36
48
60

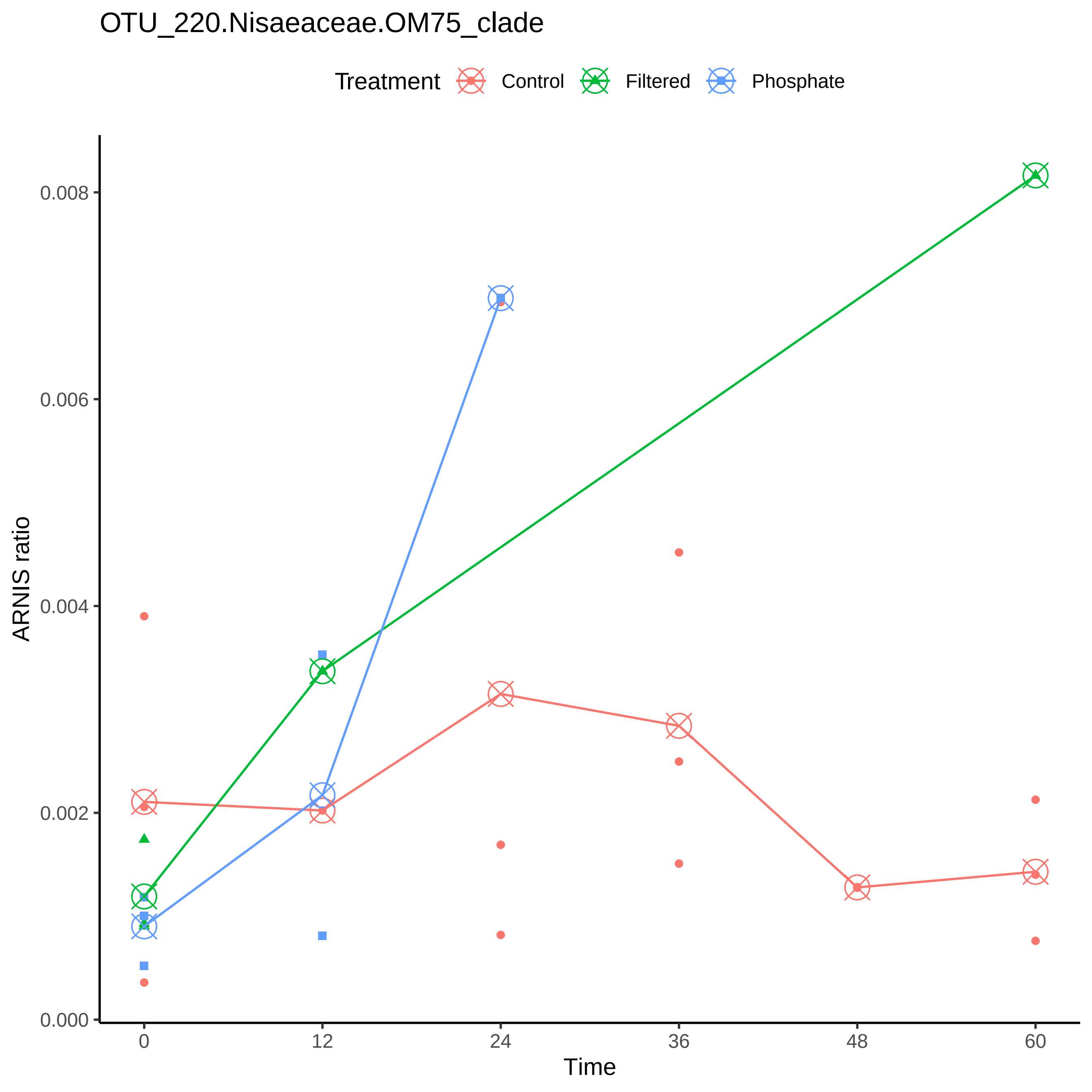

OTU_220.Nisaeaceae.OM75_clade
Treatment
Control
Filtered
Phosphate
ARNIS ratio
0.000
0.002
0.004
0.006
0.008
Time
0
12
24
36
48
60

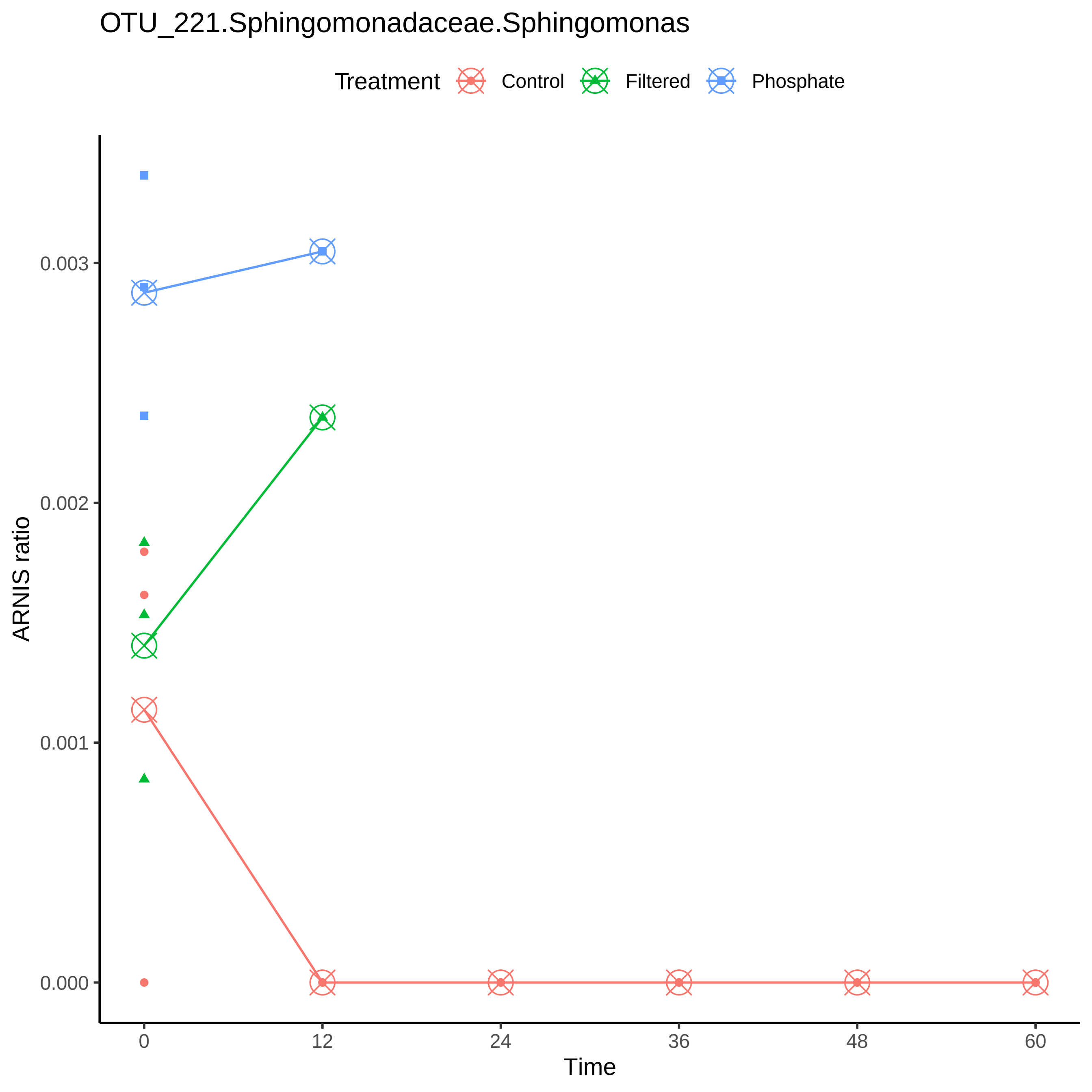
OTU_221.Sphingomonadaceae.Sphingomonas
Treatment
Control
Filtered
Phosphate
ARNIS ratio
0.003
0.002
0.001
0.000
Time
0
12
24
36
48
60

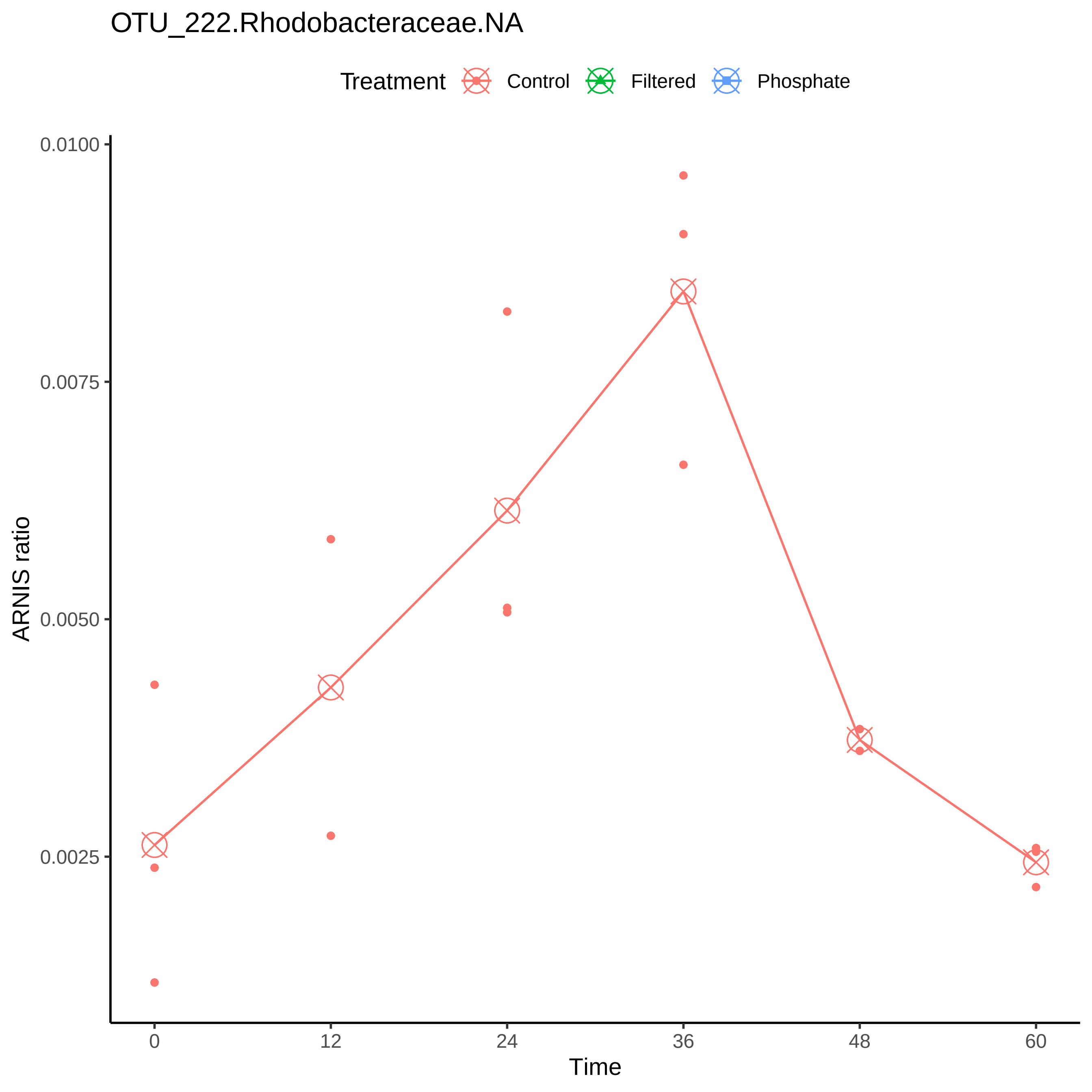
OTU_222.Rhodobacteraceae.NA
Treatment
Control
Filtered
Phosphate
0.0100
0.0075
0.0050
0.0025
ARNIS ratio
0
12
24
36
48
60
Time

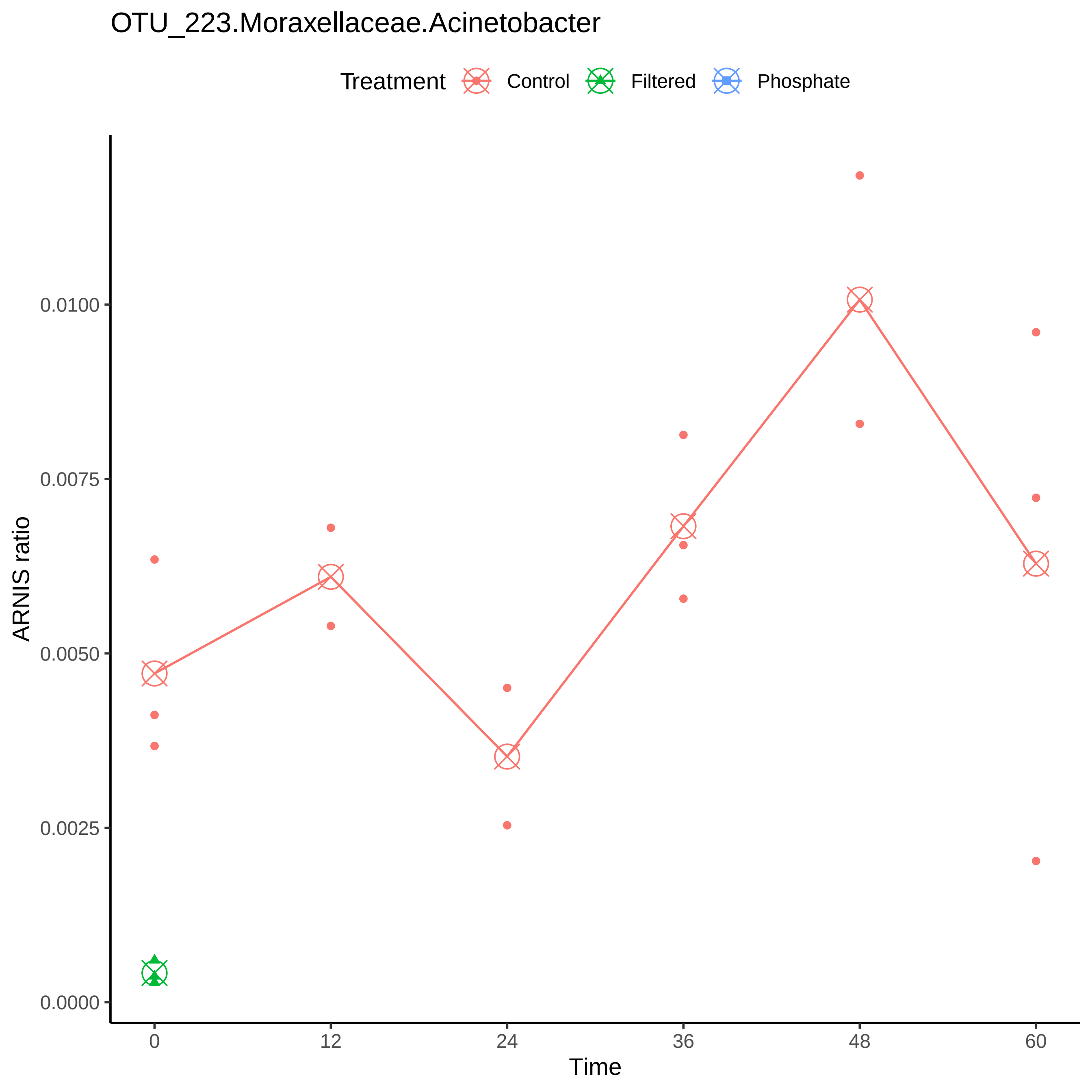

OTU_223.Moraxellaceae.Acinetobacter
Treatment
Control
Filtered
Phosphate
ARNIS ratio
0.0100
0.0075
0.0050
0.0025
0.0000
0
12
24
36
48
60
Time

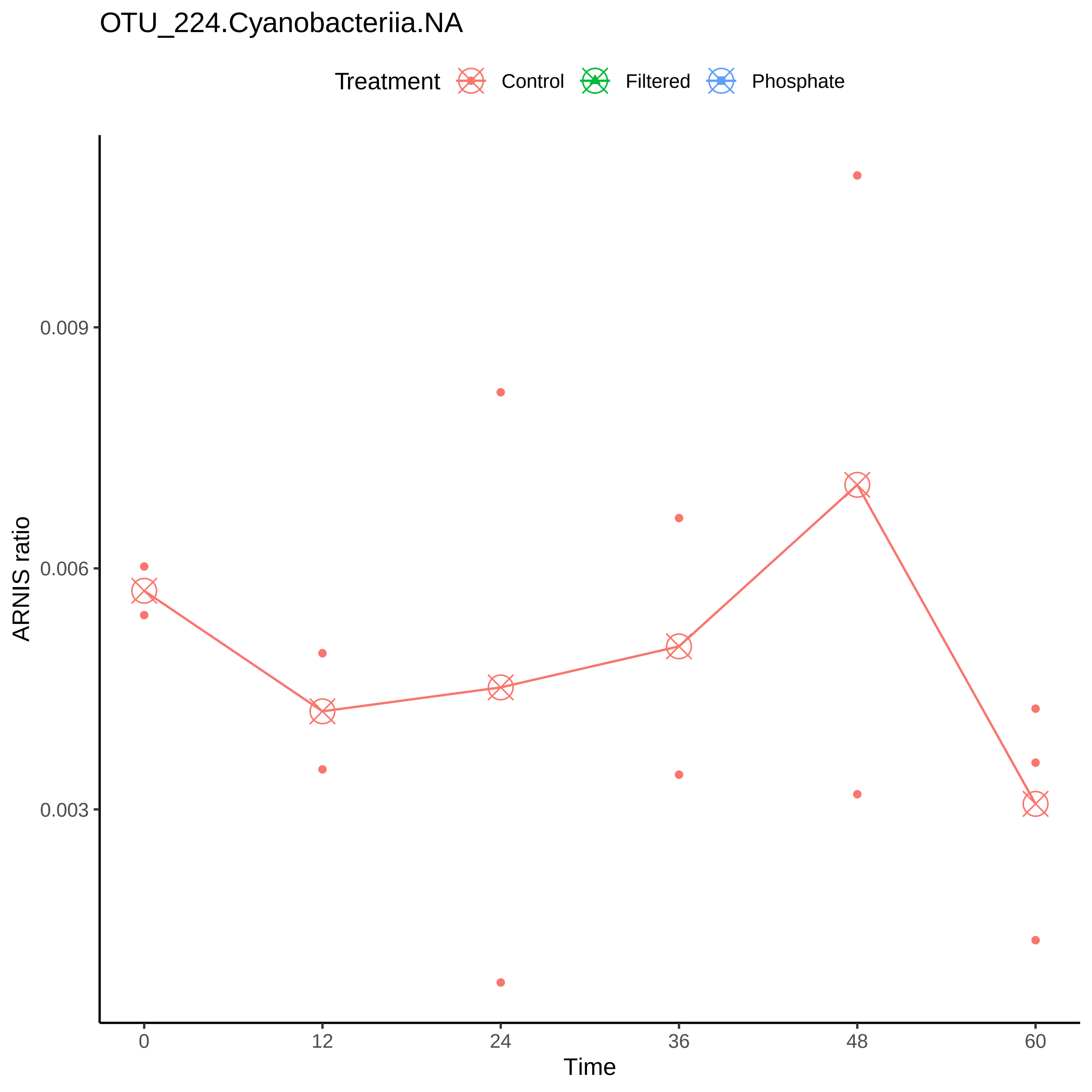
OTU_224.Cyanobacteriia.NA
Treatment
Control
Filtered
Phosphate
0.009
0.006
0.003
ARNIS ratio
0
12
24
36
48
60
Time

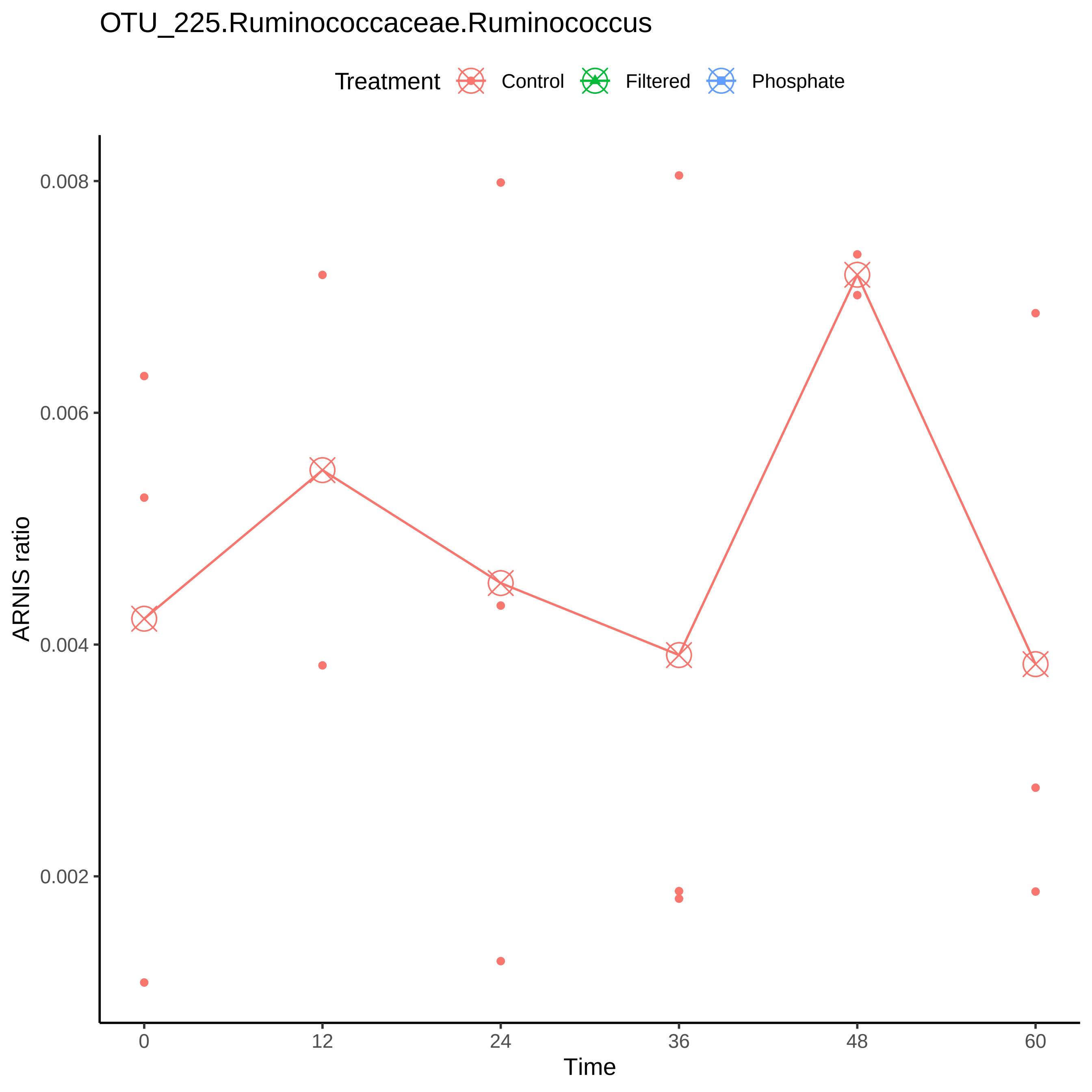

OTU_225.Ruminococcaceae.Ruminococcus
Treatment
Control
Filtered
Phosphate
ARNIS ratio
0.008
0.006
0.004
0.002
0
12
24
36
48
60
Time

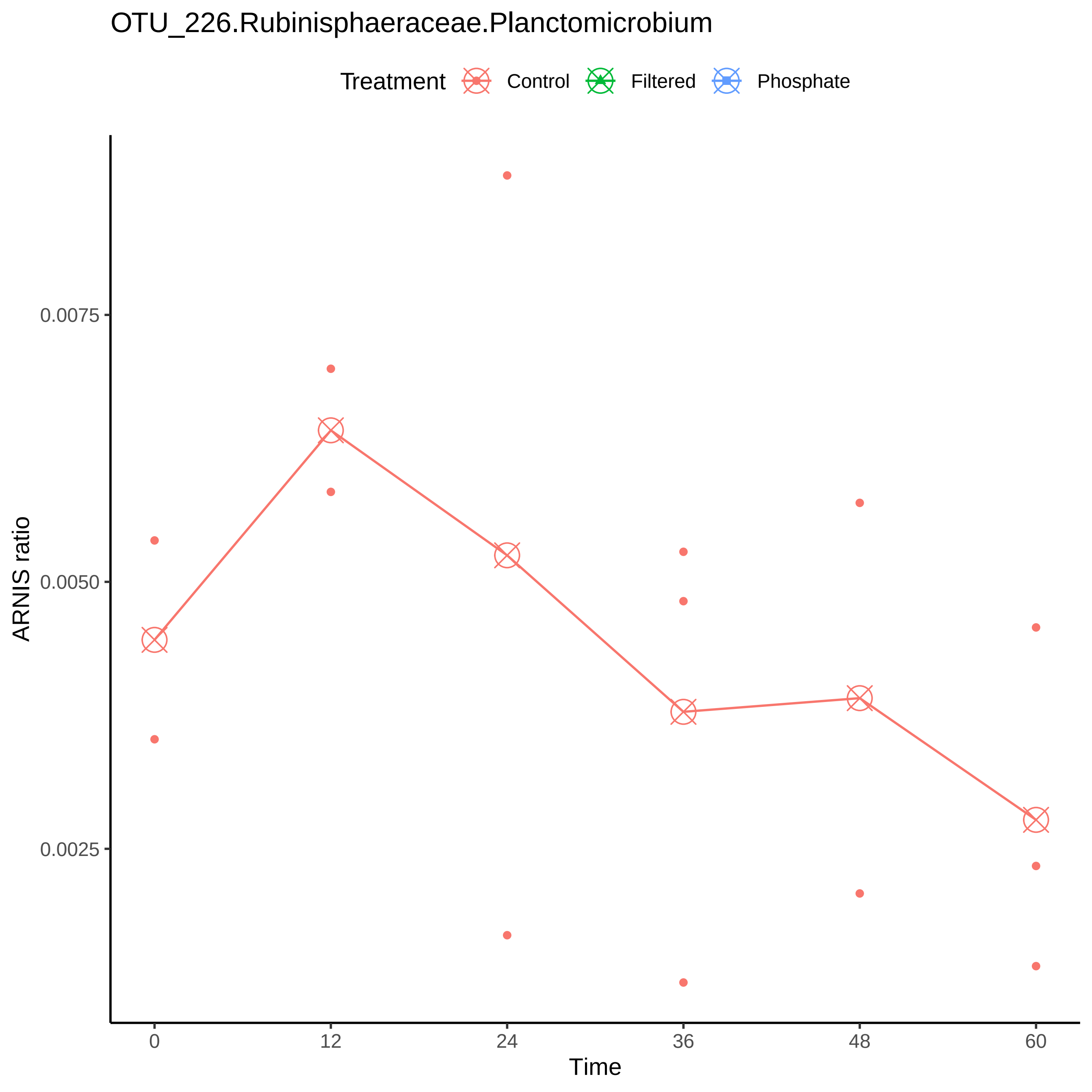
OTU_226.Rubinisphaeraceae.Planctomicrobium
Treatment
Control
Filtered
Phosphate
ARNIS ratio
0.0075
0.0050
0.0025
0
12
24
36
48
60
Time

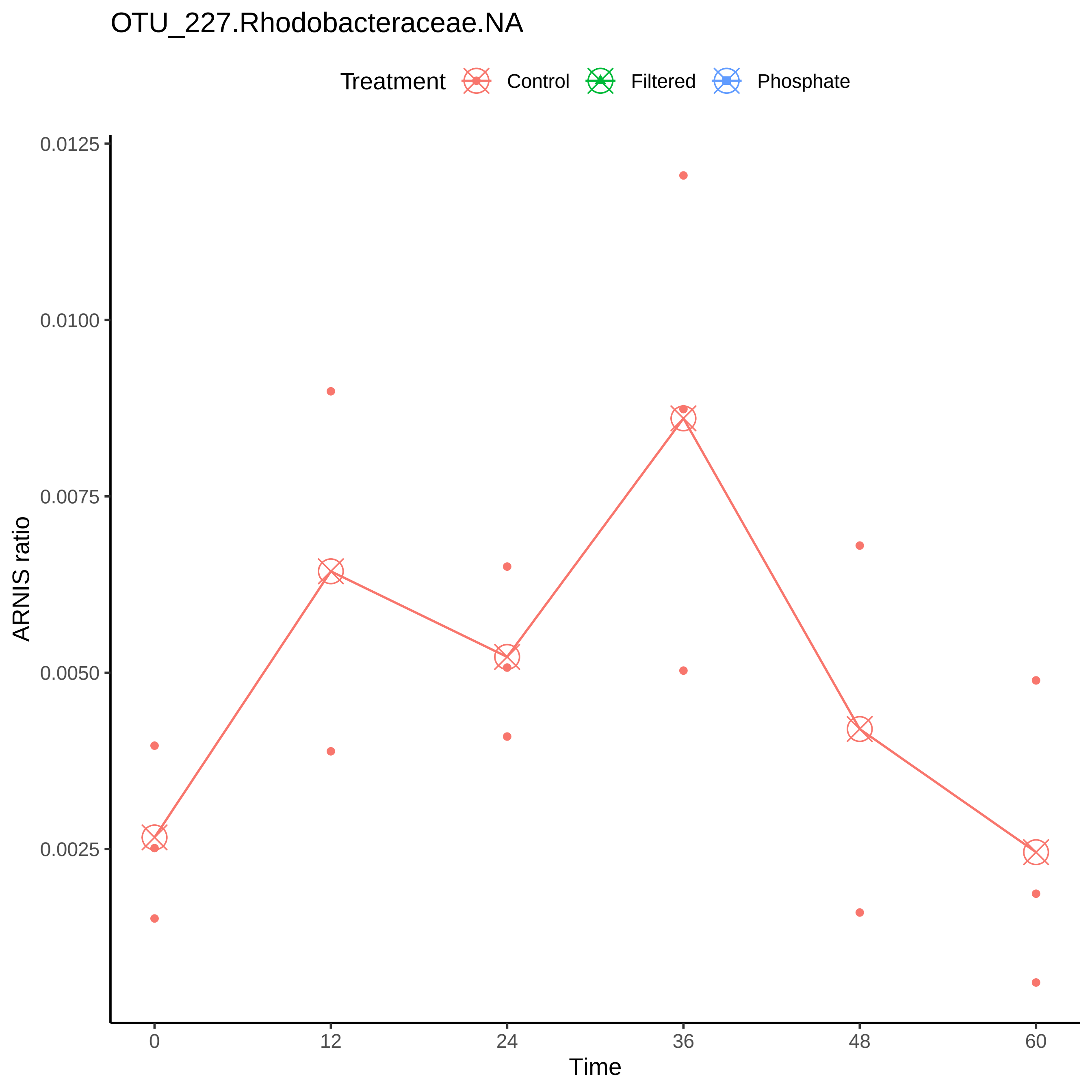

OTU_227.Rhodobacteraceae.NA
Treatment
Control
Filtered
Phosphate
ARNIS ratio
0.0125
0.0100
0.0075
0.0050
0.0025
0
12
24
36
48
60
Time

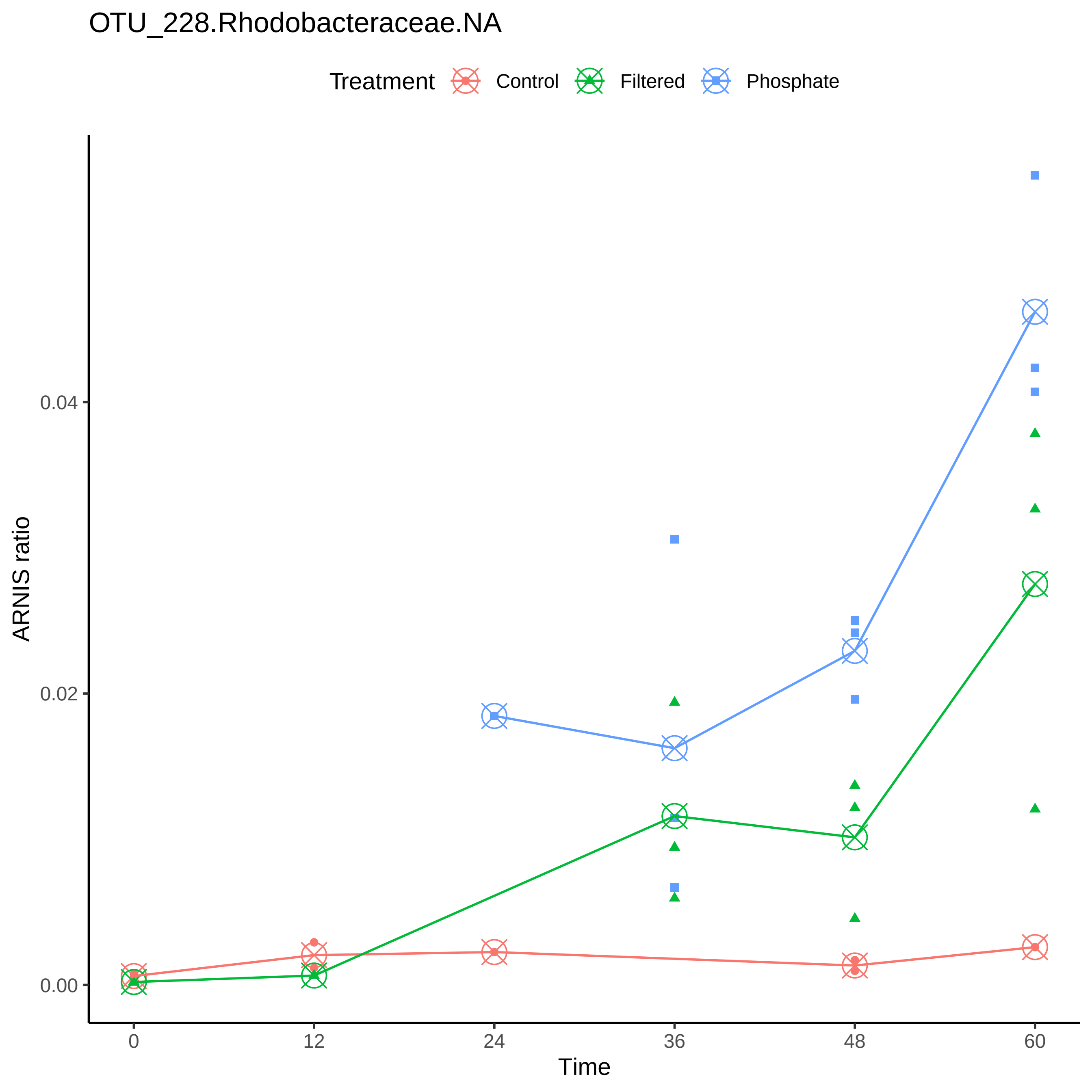

OTU_228.Rhodobacteraceae.NA
Treatment
Control
Filtered
Phosphate
ARNIS ratio
0.00
0.02
0.04
0
12
24
36
48
60
Time

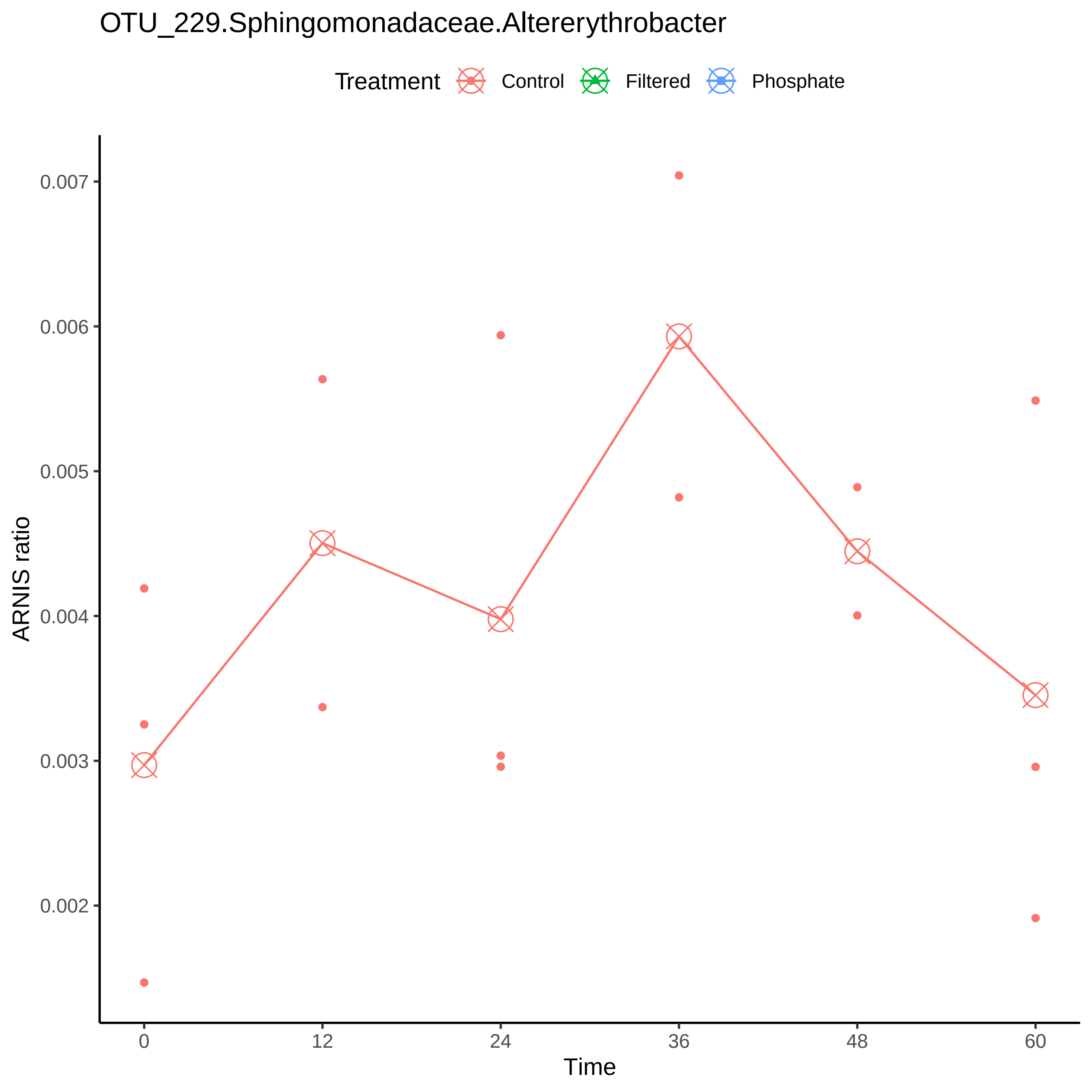
OTU_229.Sphingomonadaceae.Altererythrobacter
Treatment
Control
Filtered
Phosphate
ARNIS ratio
0.007
0.006
0.005
0.004
0.003
0.002
Time
0
12
24
36
48
60

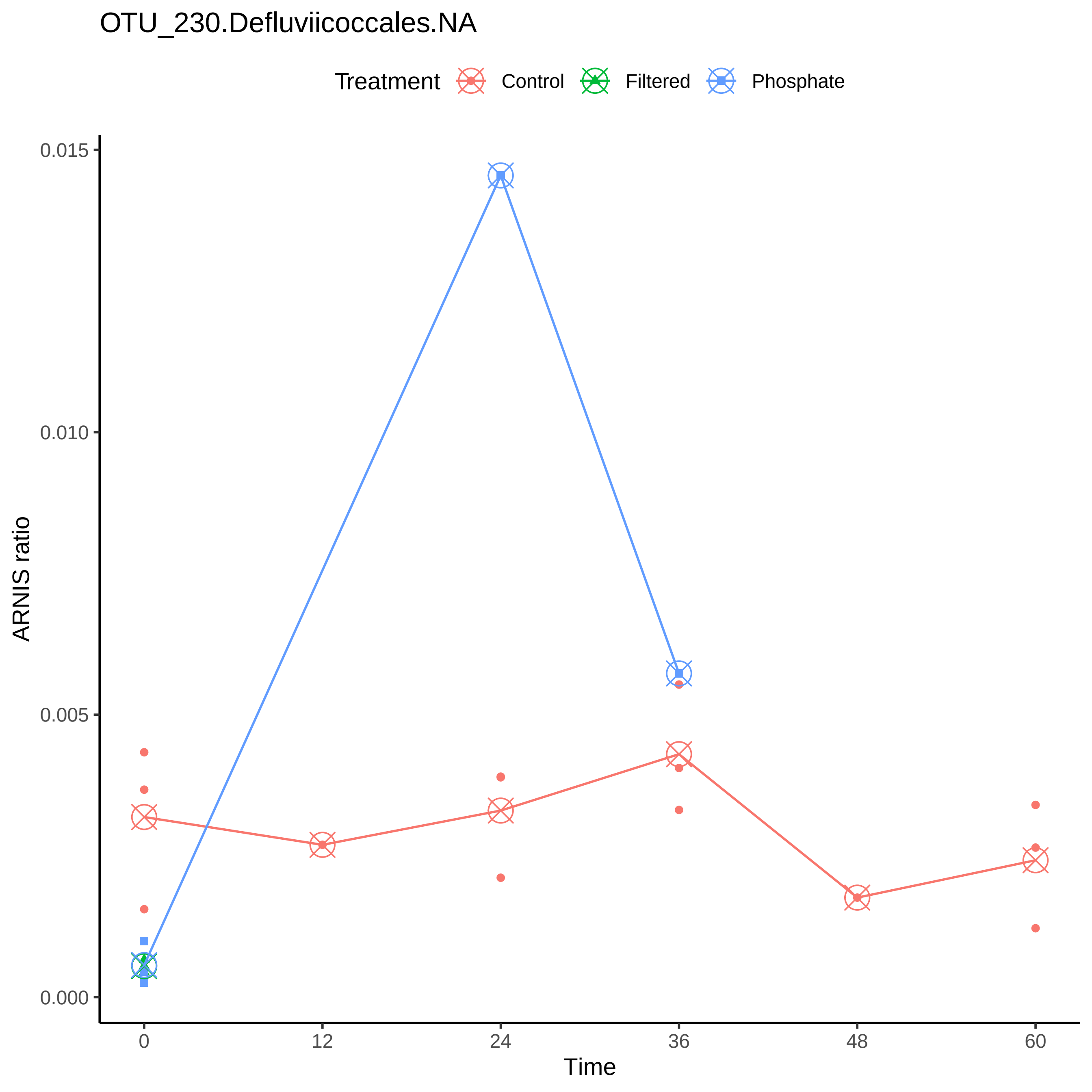

OTU_230.Defluviicoccales.NA
Treatment
Control
Filtered
Phosphate
ARNIS ratio
0.015
0.010
0.005
0.000
Time
0
12
24
36
48
60

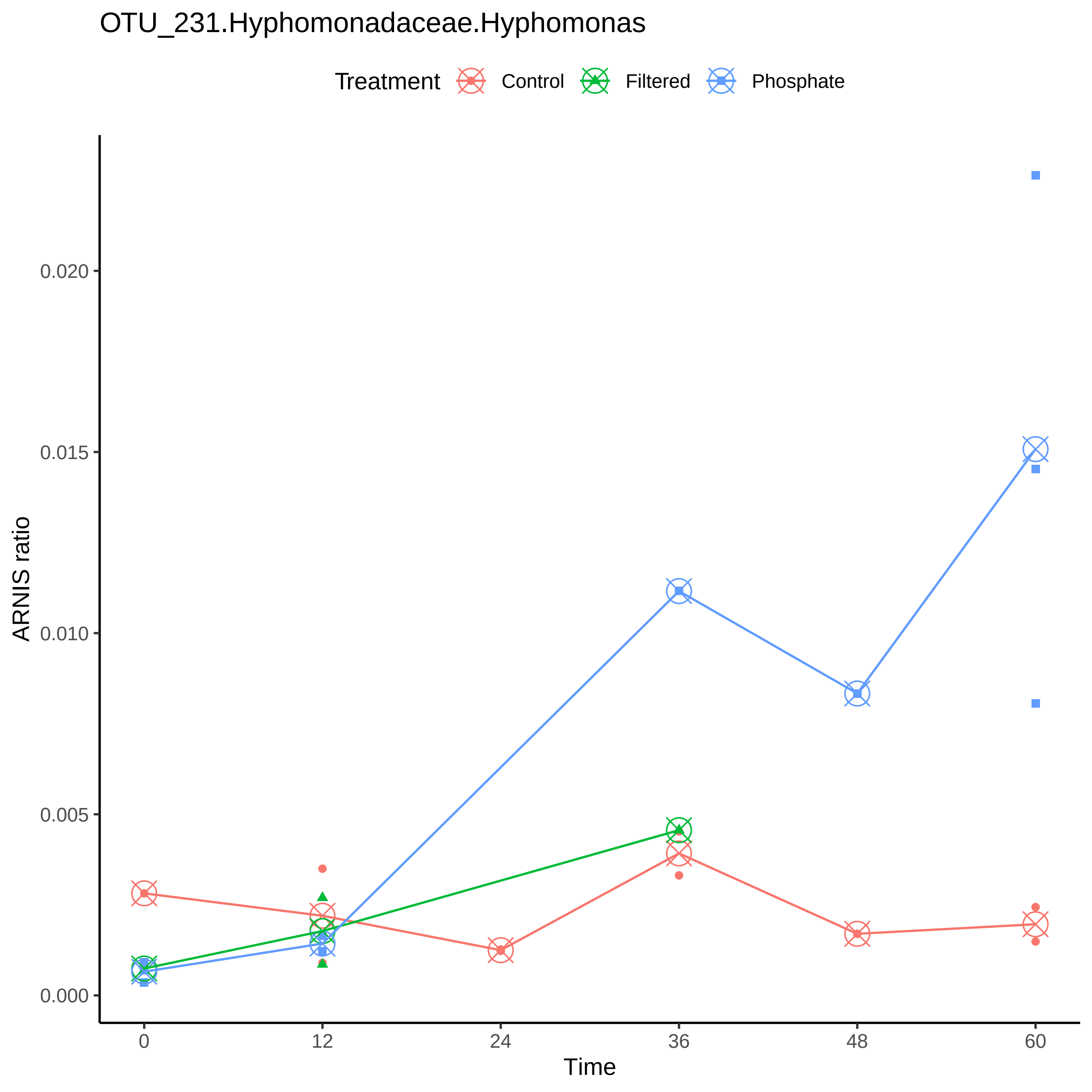
OTU_231.Hyphomonadaceae.Hyphomonas
Treatment
Control
Filtered
Phosphate
ARNIS ratio
0.000
0.005
0.010
0.015
0.020
0
12
24
36
48
60
Time

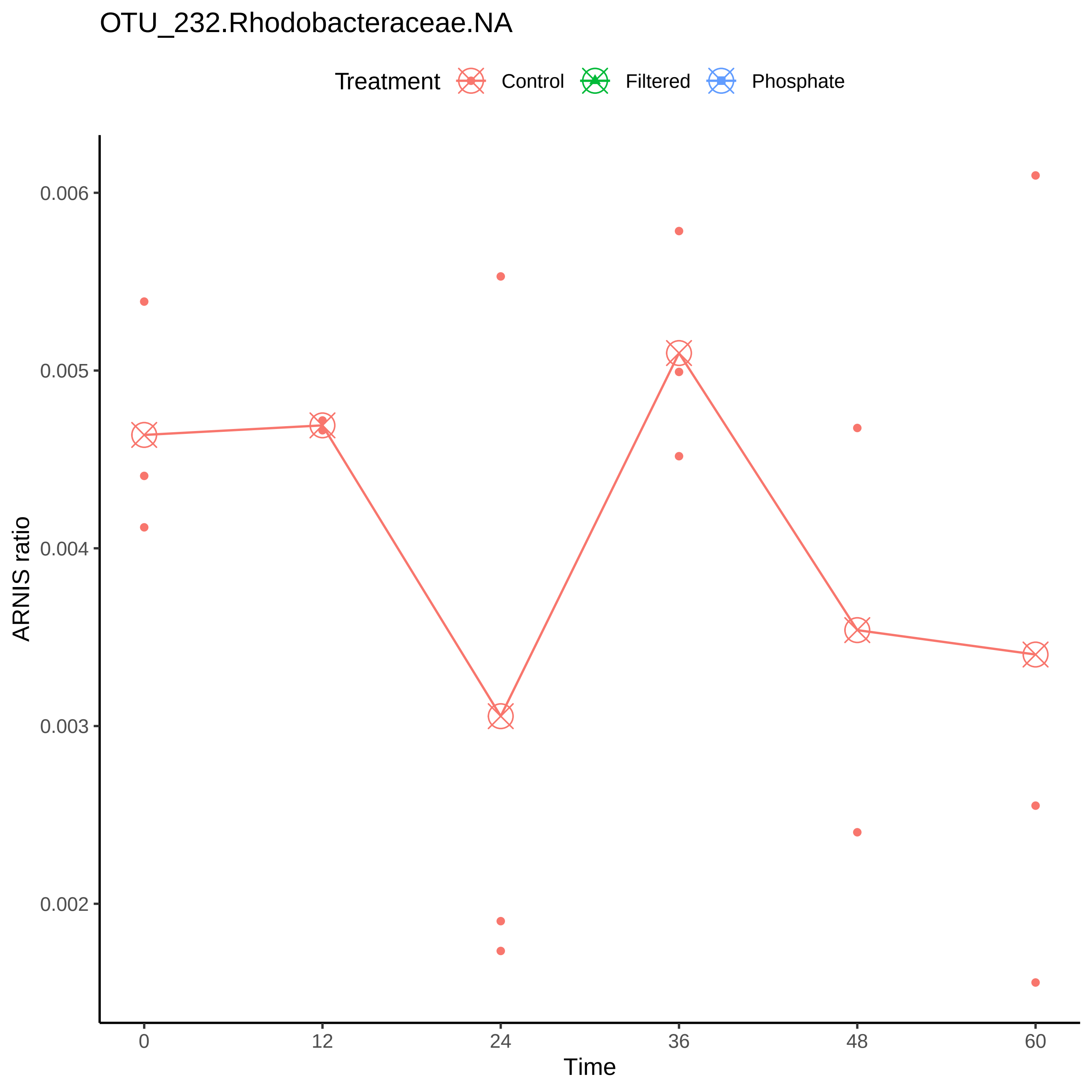
OTU_232.Rhodobacteraceae.NA
Treatment
Control
Filtered
Phosphate
ARNIS ratio
0.006
0.005
0.004
0.003
0.002
0
12
24
36
48
60
Time

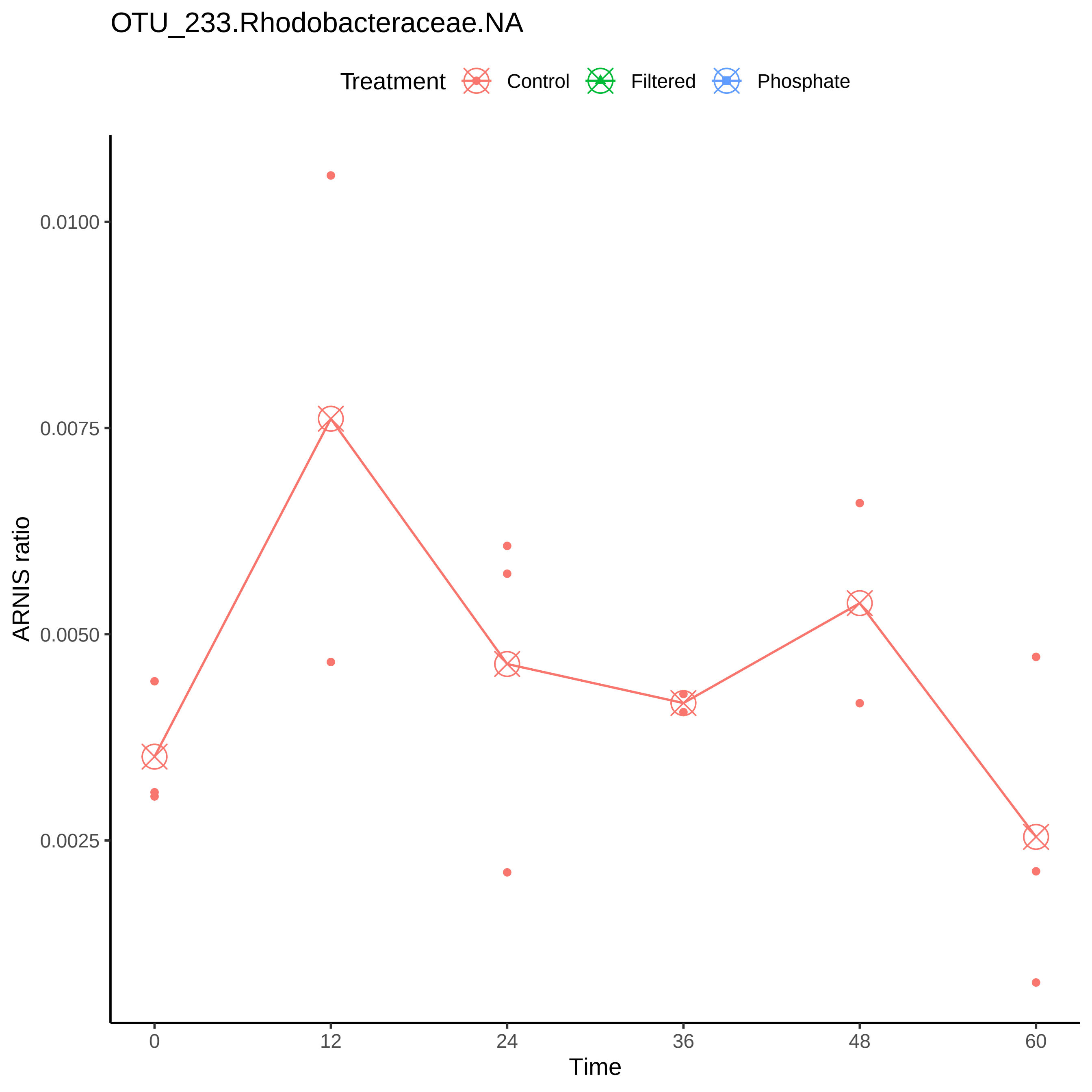

OTU_233.Rhodobacteraceae.NA
Treatment
Control
Filtered
Phosphate
0.0100
0.0075
0.0050
0.0025
ARNIS ratio
0
12
24
36
48
60
Time

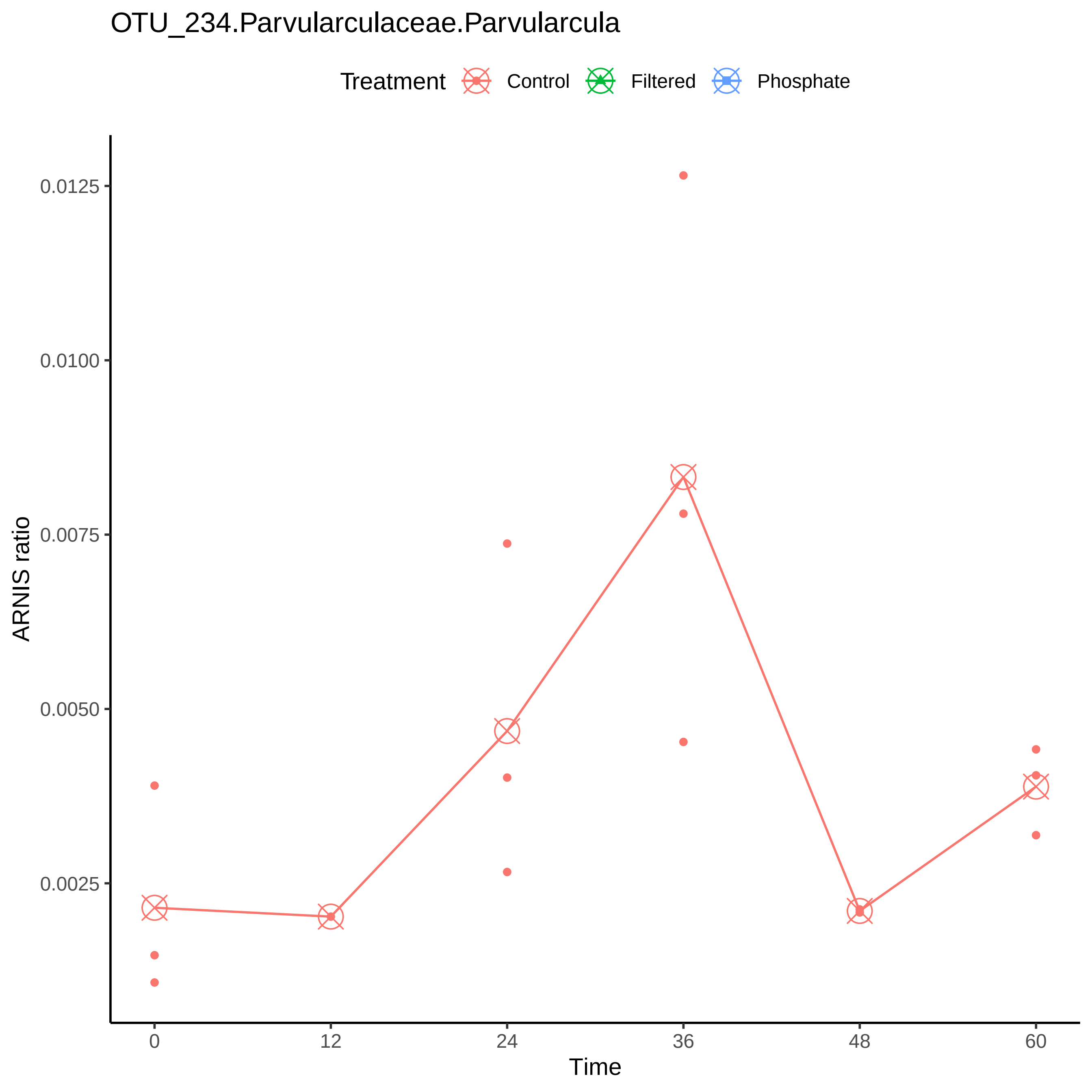
OTU_234.Parvularculaceae.Parvularcula
Treatment
Control
Filtered
Phosphate
ARNIS ratio
0.0125
0.0100
0.0075
0.0050
0.0025
0
12
24
36
48
60
Time

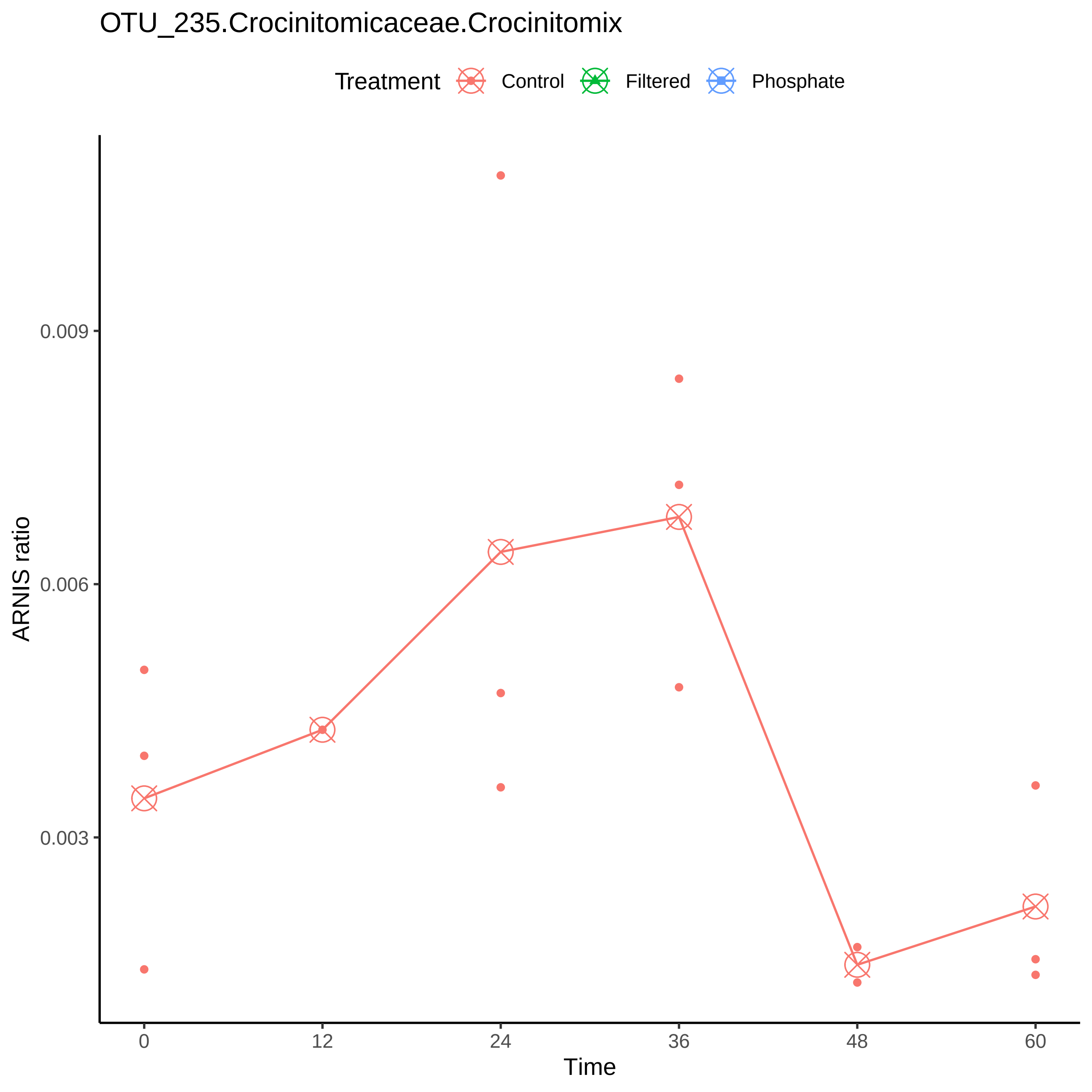

OTU_235.Crocinitomicaceae.Crocinitomix
Treatment
Control
Filtered
Phosphate
ARNIS ratio
0.009
0.006
0.003
0
12
24
36
48
60
Time

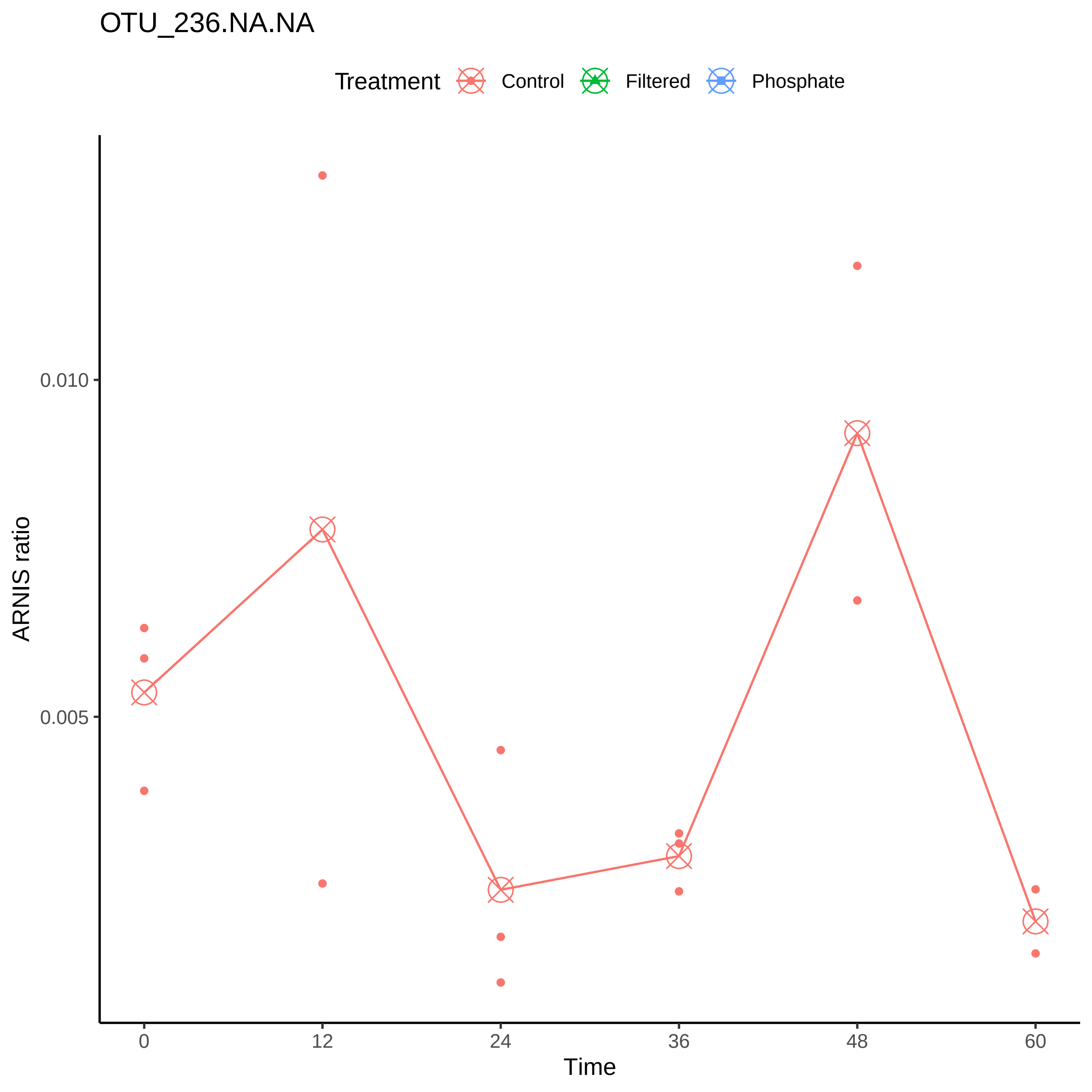

OTU_236.NA.NA
Treatment
Control
Filtered
Phosphate
ARNIS ratio
0.010
0.005
0
12
24
36
48
60
Time

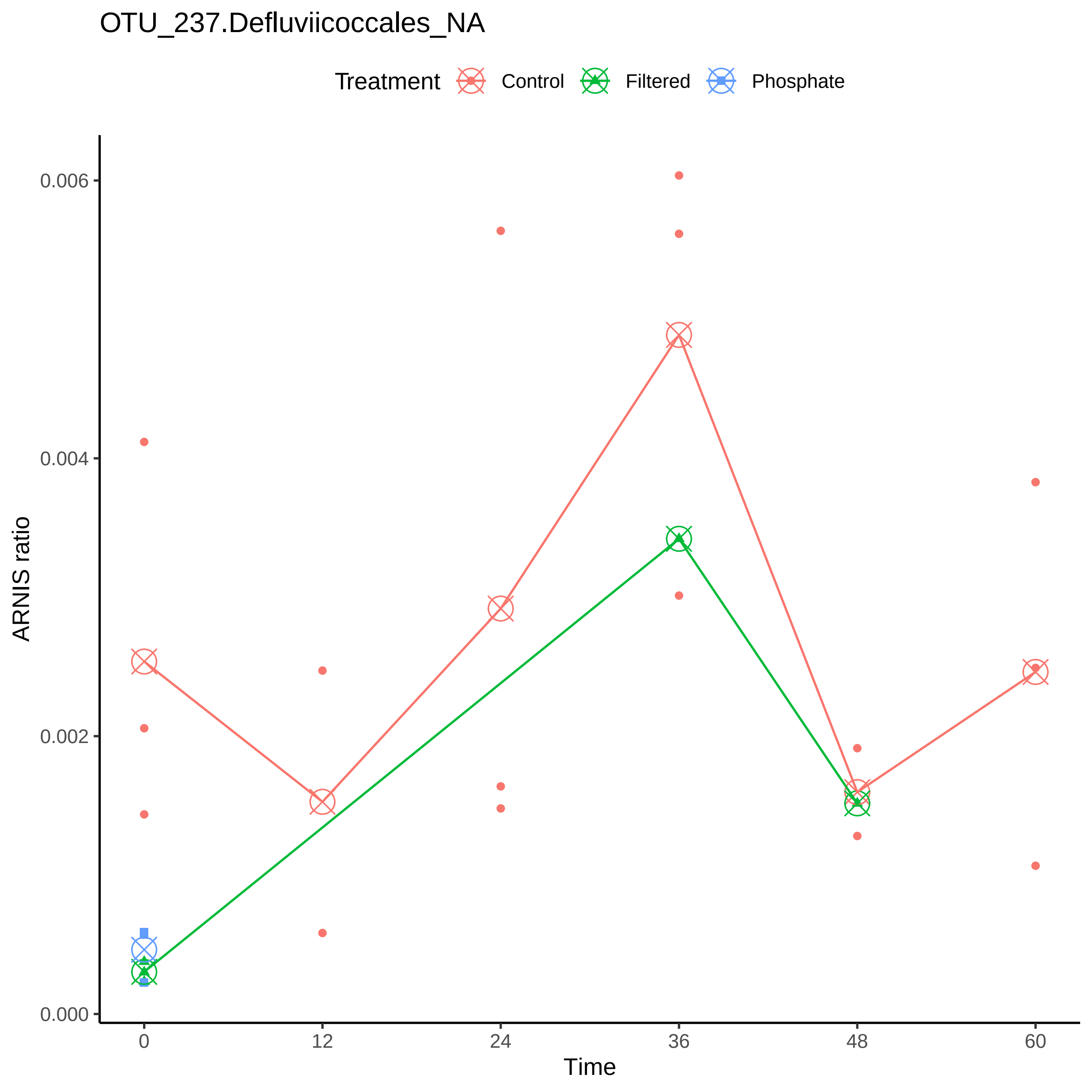
OTU_237.Defluviicoccales_NA
Treatment
Control
Filtered
Phosphate
ARNIS ratio
0.006
0.004
0.002
0.000
Time
0
12
24
36
48
60

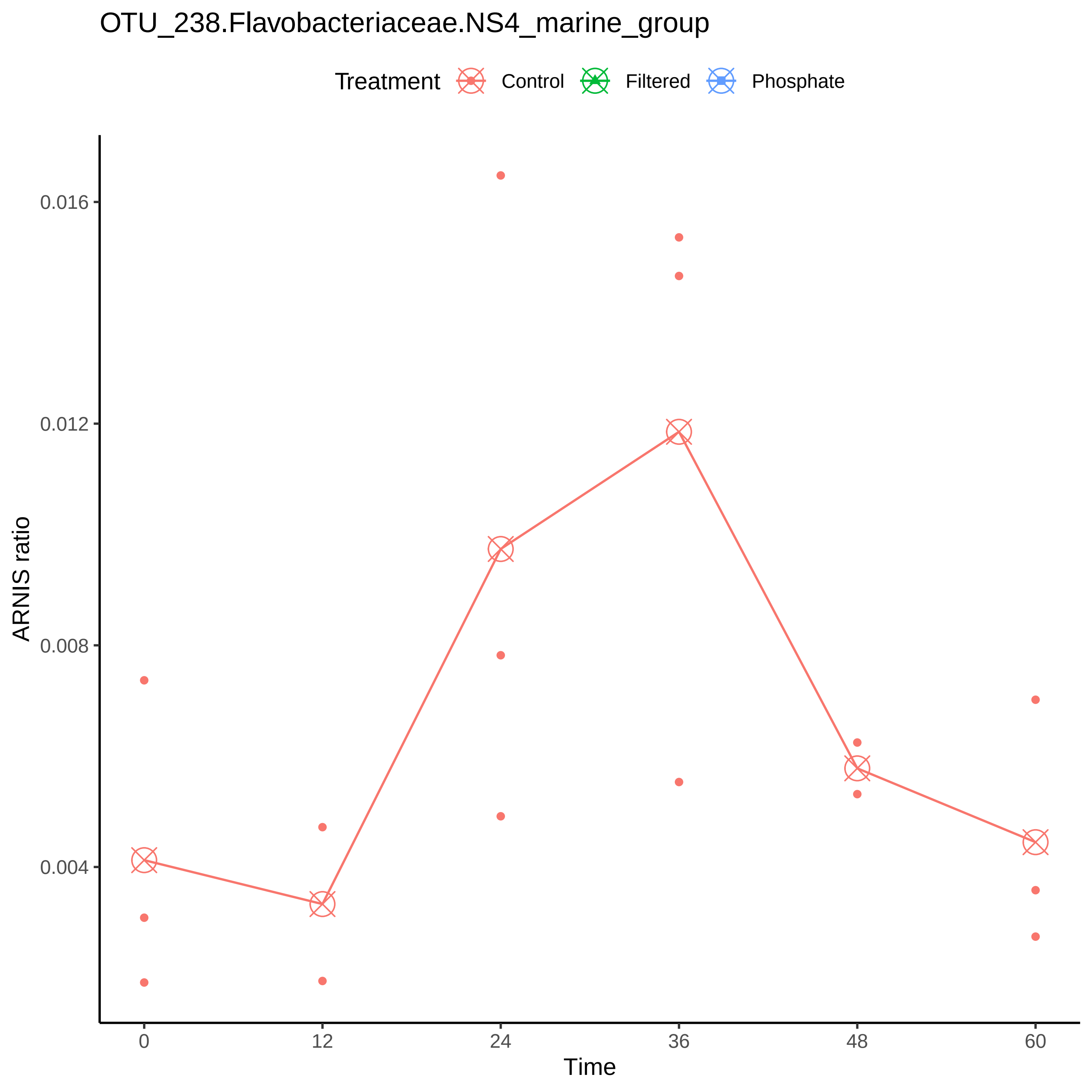

OTU_238.Flavobacteriaceae.NS4_marine_group
Treatment
Control
Filtered
Phosphate
0.016
0.012
0.008
0.004
ARNIS ratio
0
12
24
36
48
60
Time

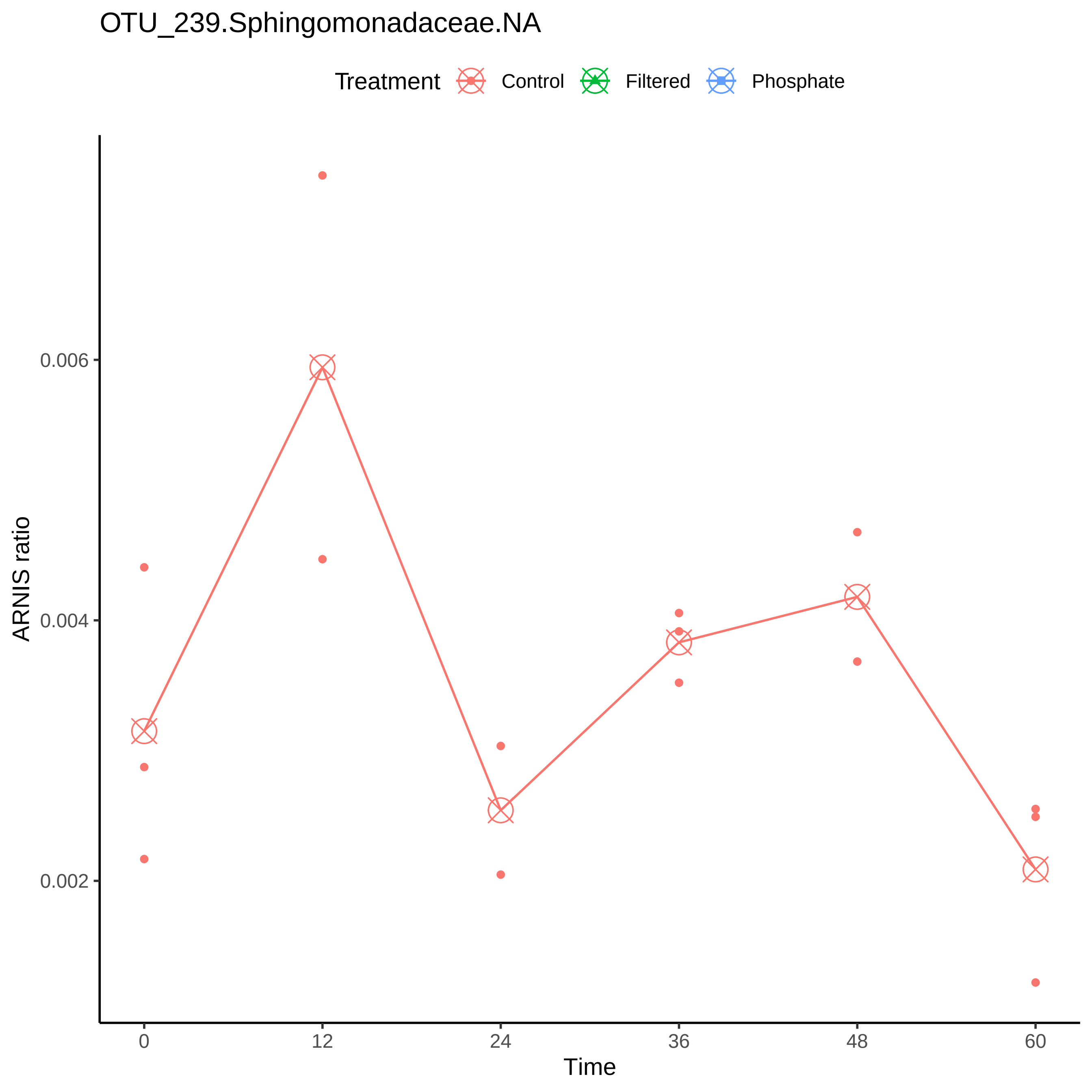
OTU_239.Sphingomonadaceae.NA
Treatment
Control
Filtered
Phosphate
ARNIS ratio
0.006
0.004
0.002
0
12
24
36
48
60
Time

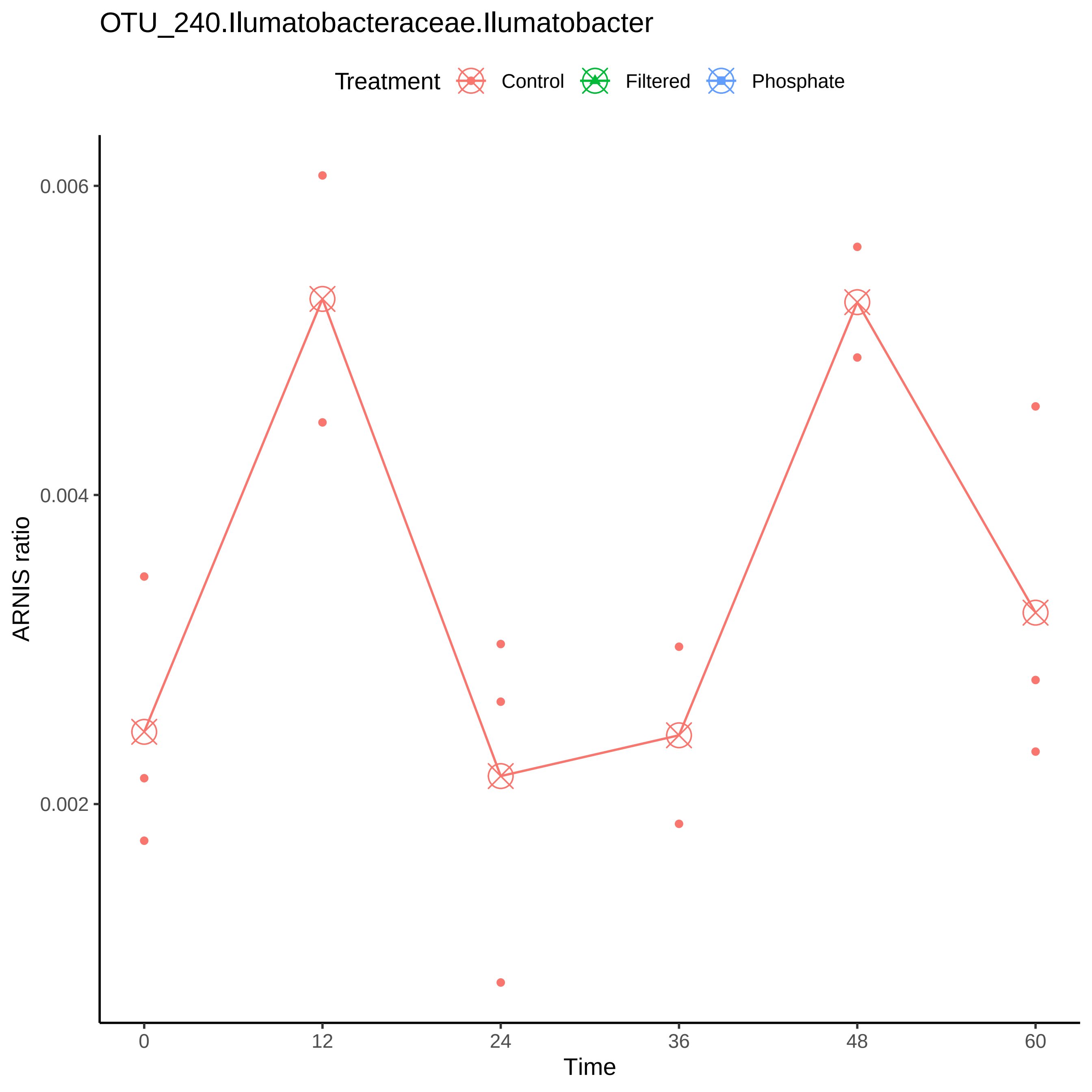
OTU_240.Ilumatobacteraceae.Ilumatobacter
Treatment
Control
Filtered
Phosphate
ARNIS ratio
0.006
0.004
0.002
Time
0
12
24
36
48
60

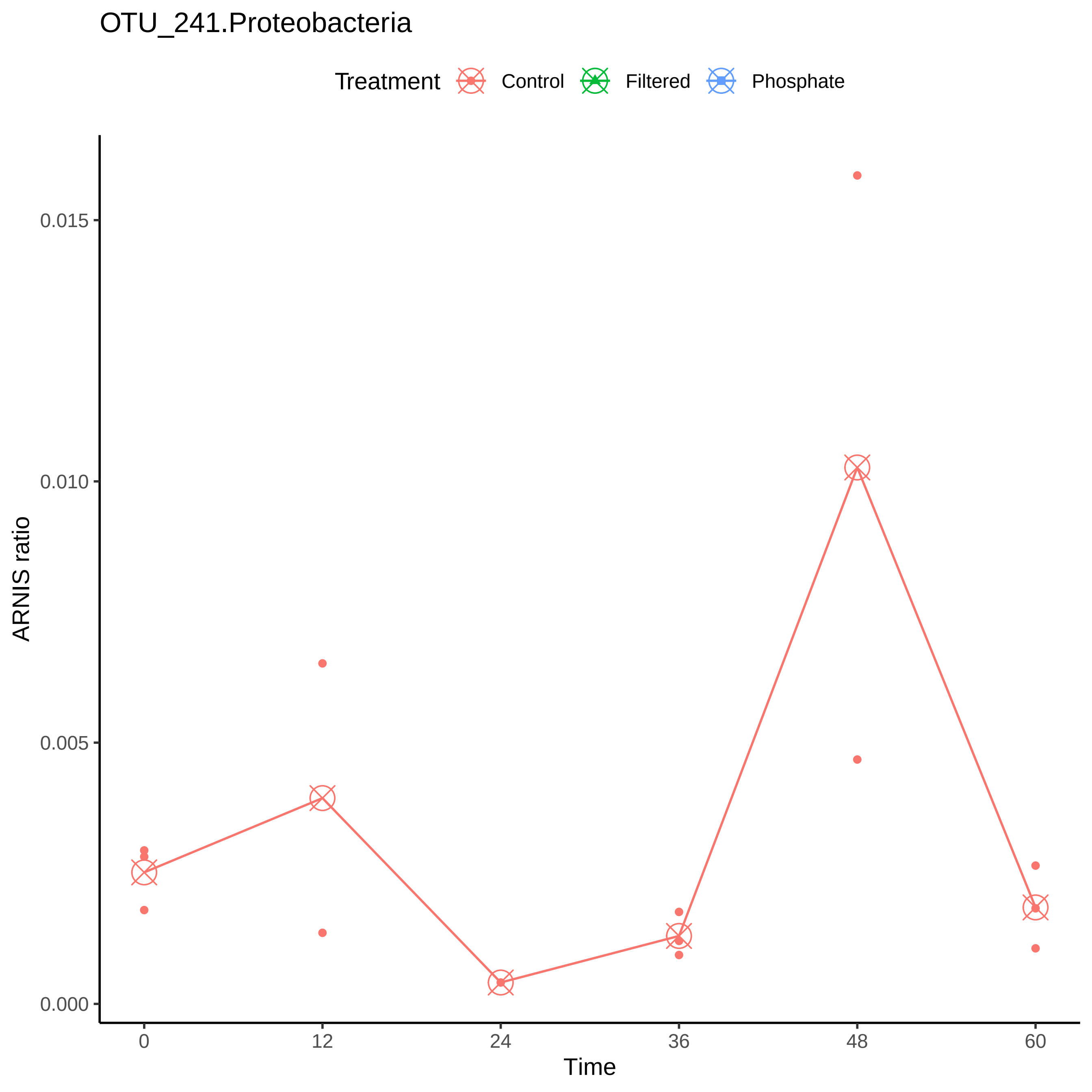
OTU_241.Proteobacteria
Treatment
Control
Filtered
Phosphate
ARNIS ratio
0.015
0.010
0.005
0.000
Time
0
12
24
36
48
60

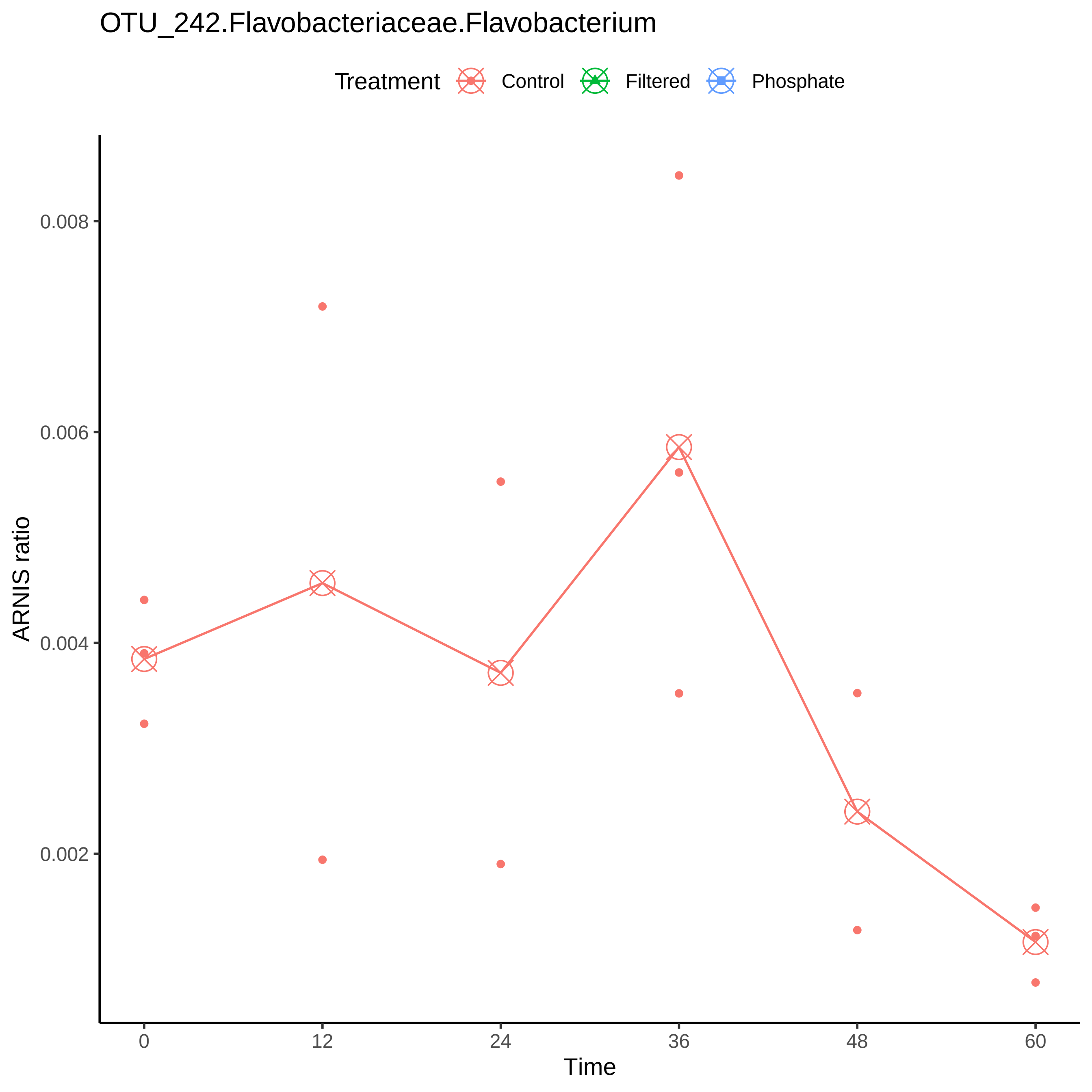
OTU_242.Flavobacteriaceae.Flavobacterium
Treatment
Control
Filtered
Phosphate
ARNIS ratio
0.008
0.006
0.004
0.002
Time
0
12
24
36
48
60

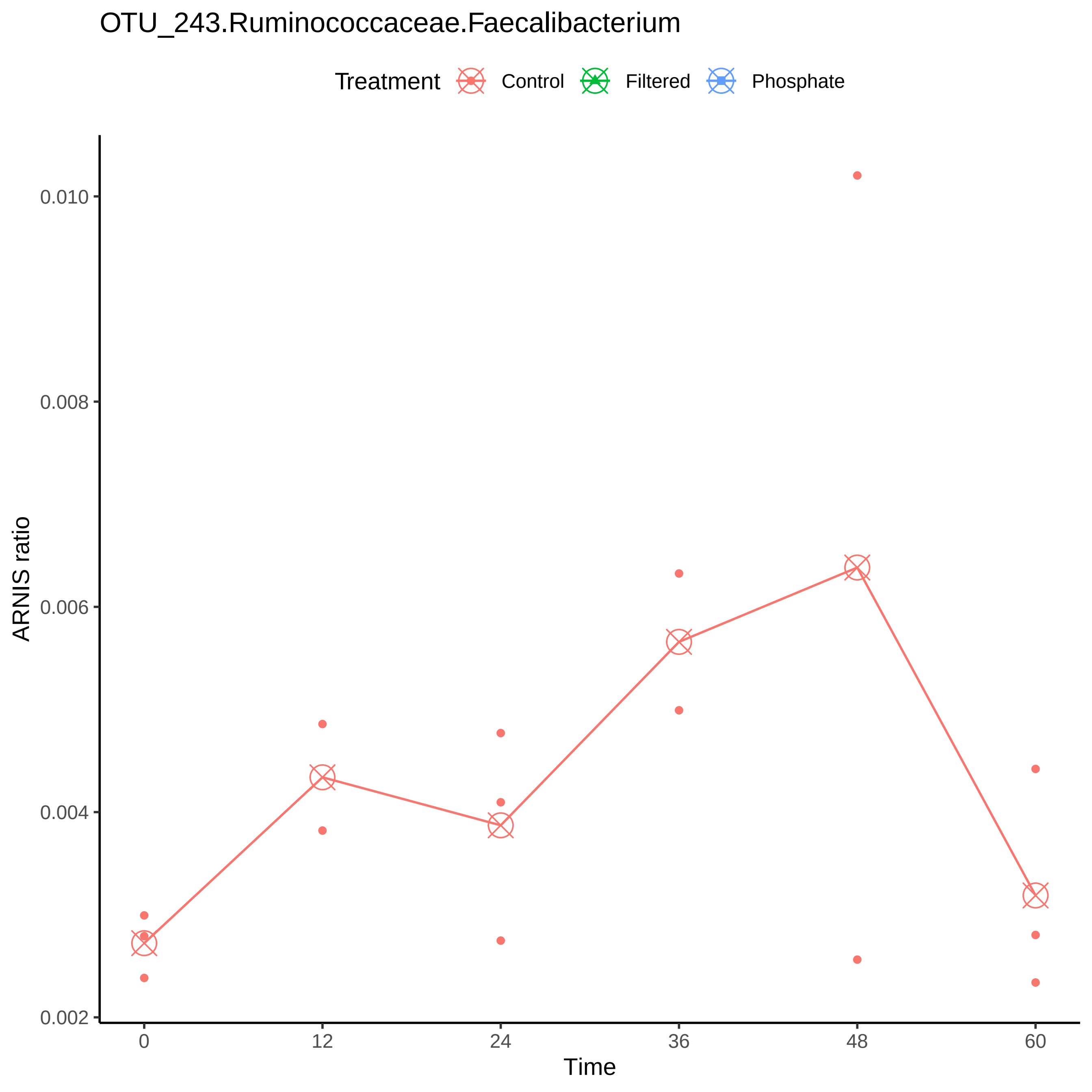

OTU_243.Ruminococcaceae.Faecalibacterium
Treatment
Control
Filtered
Phosphate
ARNIS ratio
0.010
0.008
0.006
0.004
0.002
0
12
24
36
48
60
Time

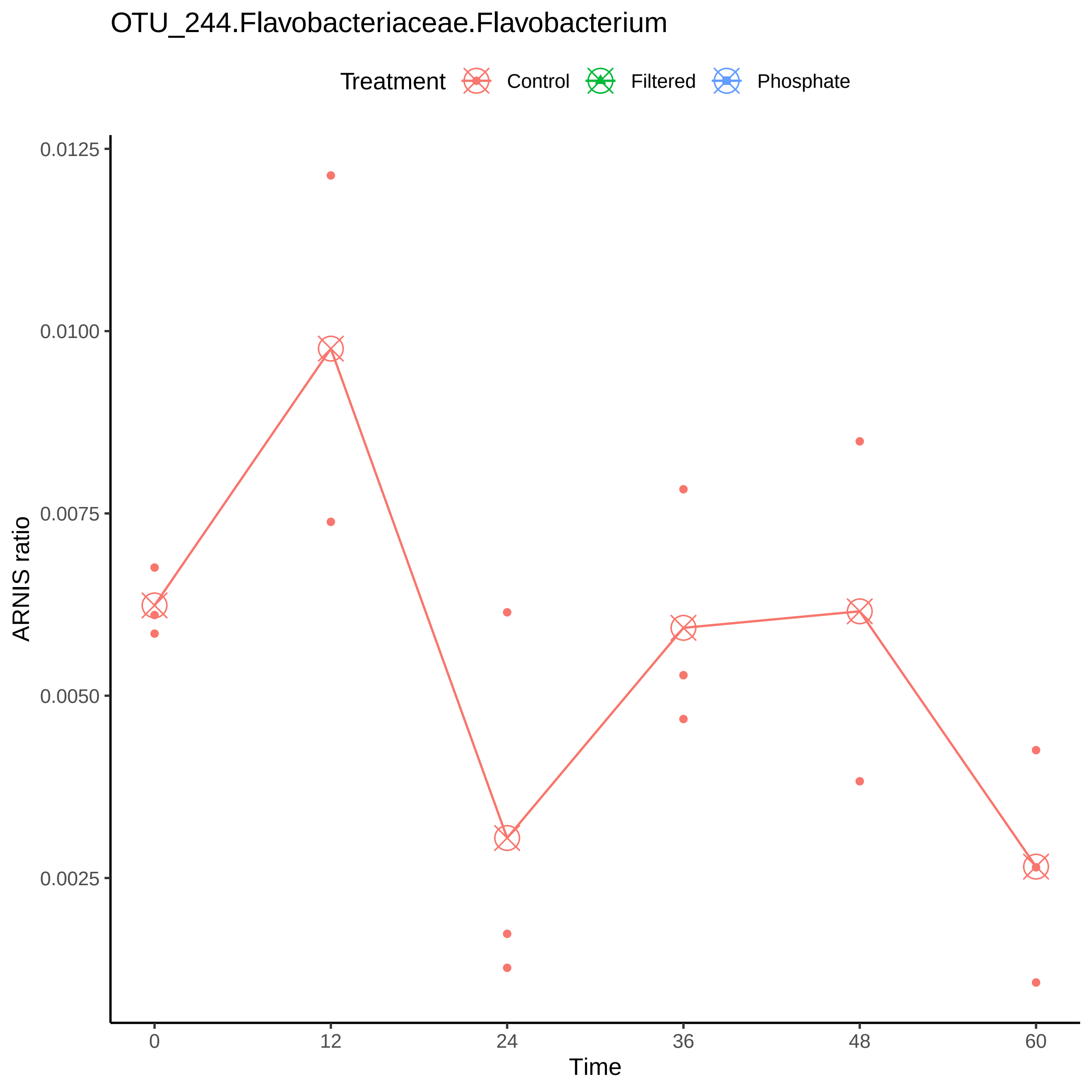
OTU_244.Flavobacteriaceae.Flavobacterium
Treatment
Control
Filtered
Phosphate
ARNIS ratio
0.0125
0.0100
0.0075
0.0050
0.0025
Time
0
12
24
36
48
60

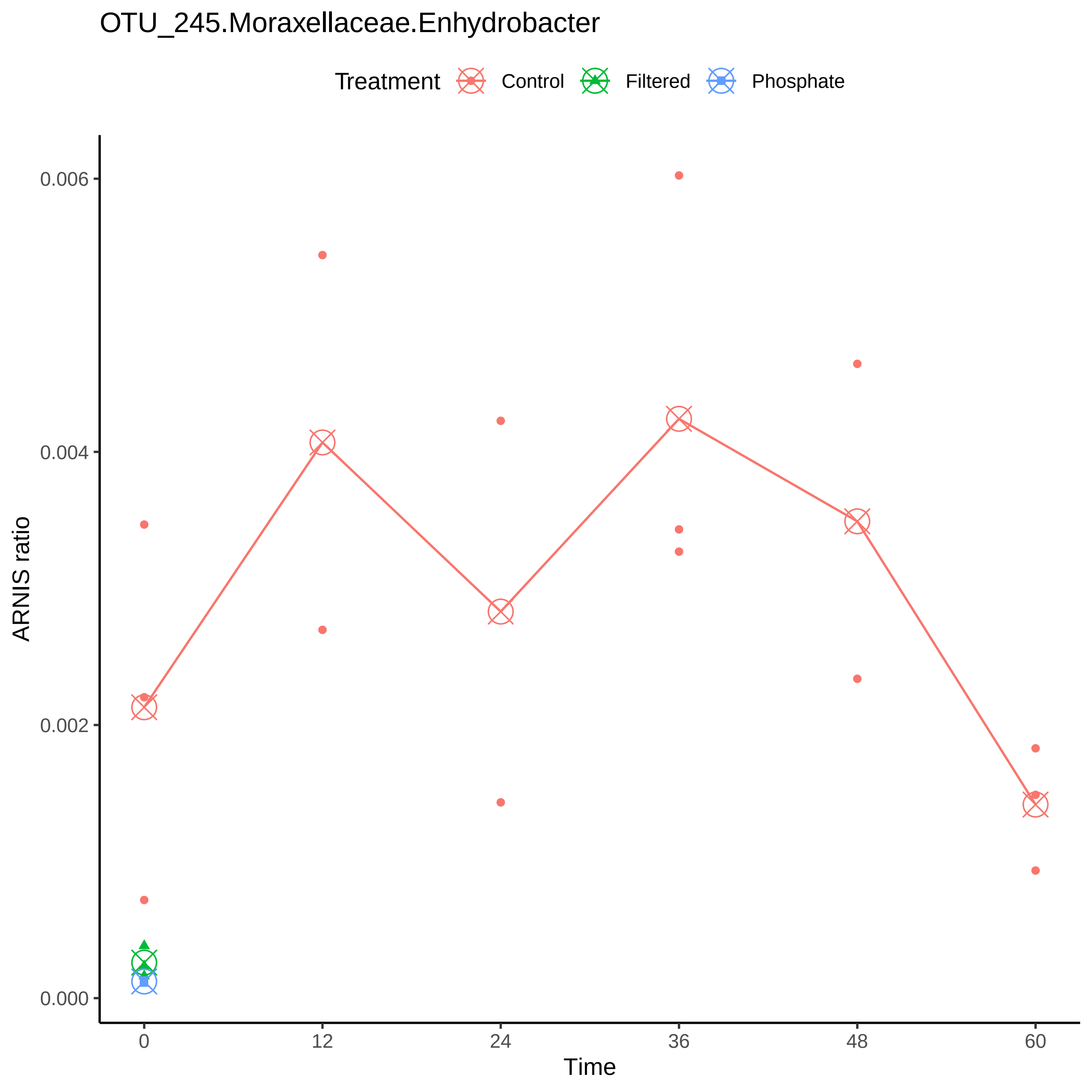

OTU_245.Moraxellaceae.Enhydrobacter
Treatment
Control
Filtered
Phosphate
ARNIS ratio
0.006
0.004
0.002
0.000
Time
0
12
24
36
48
60

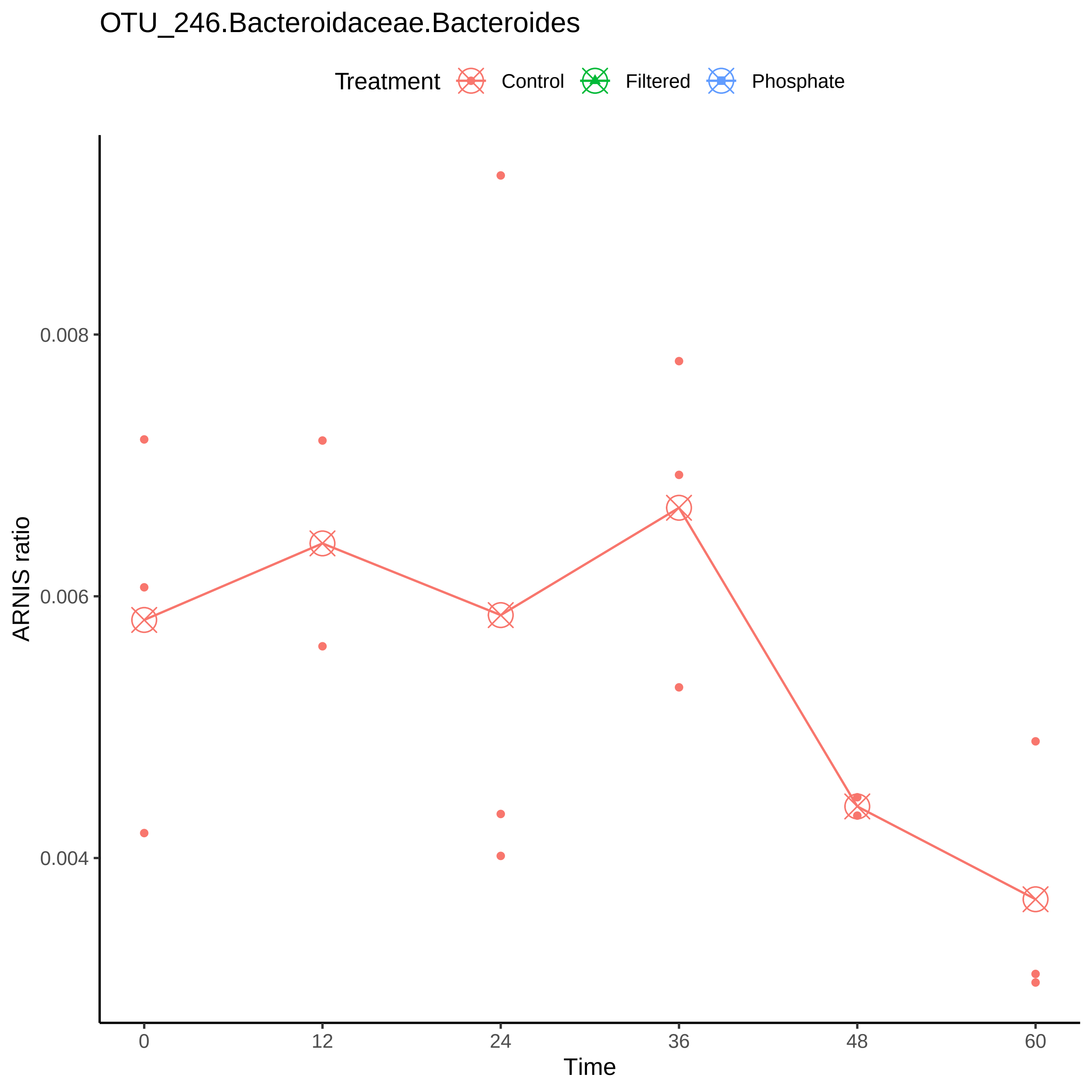

OTU_246.Bacteroidaceae.Bacteroides
Treatment
Control
Filtered
Phosphate
ARNIS ratio
0.008
0.006
0.004
0
12
24
36
48
60
Time

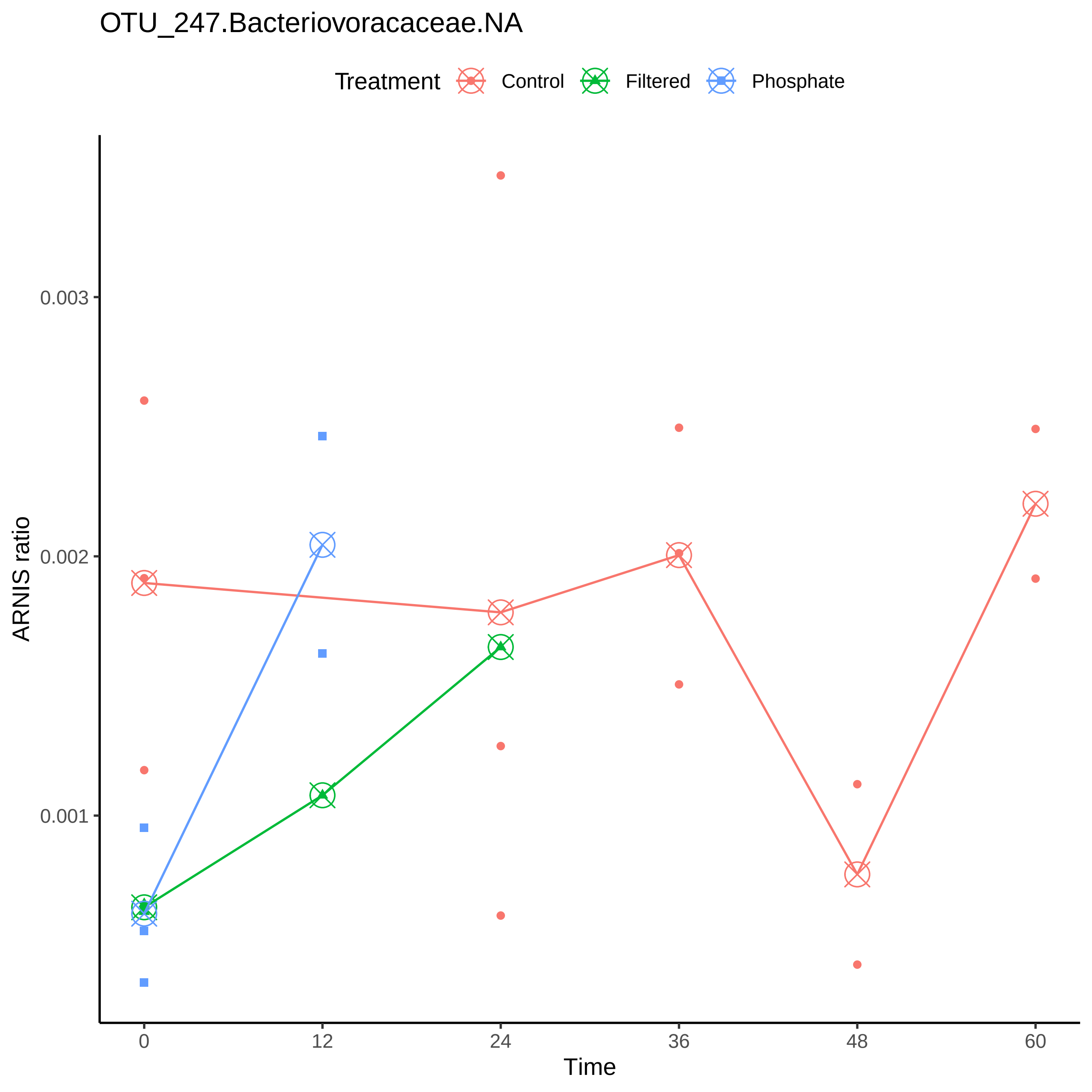

OTU_247.Bacteriovoracaceae.NA
Treatment
Control
Filtered
Phosphate
ARNIS ratio
0.003
0.002
0.001
0
12
24
36
48
60
Time

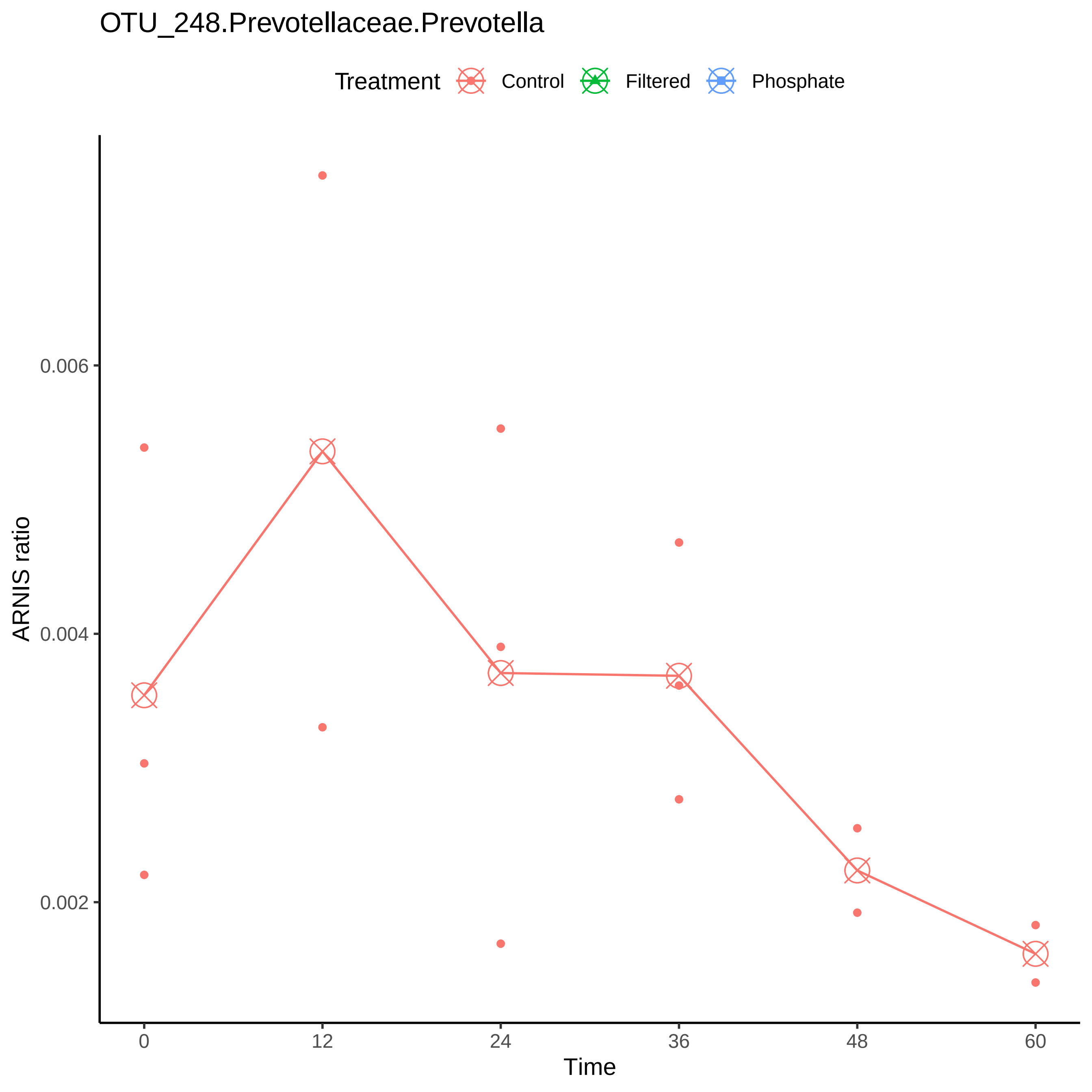

OTU_248.Prevotellaceae.Prevotella
Treatment
Control
Filtered
Phosphate
ARNIS ratio
0.006
0.004
0.002
Time
0
12
24
36
48
60

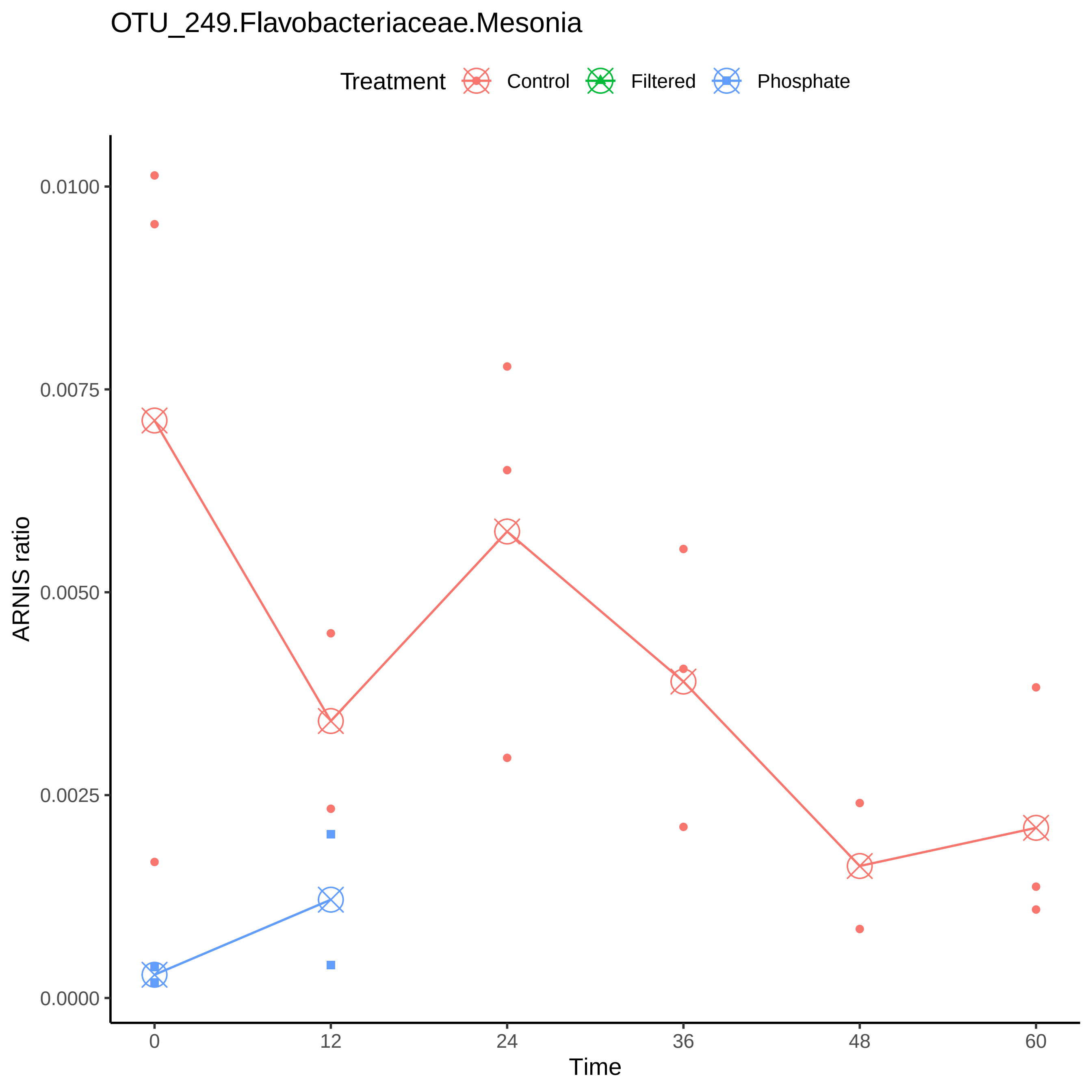

OTU_249.Flavobacteriaceae.Mesonia
Treatment
Control
Filtered
Phosphate
0.0100
0.0075
0.0050
0.0025
0.0000
ARNIS ratio
0
12
24
36
48
60
Time

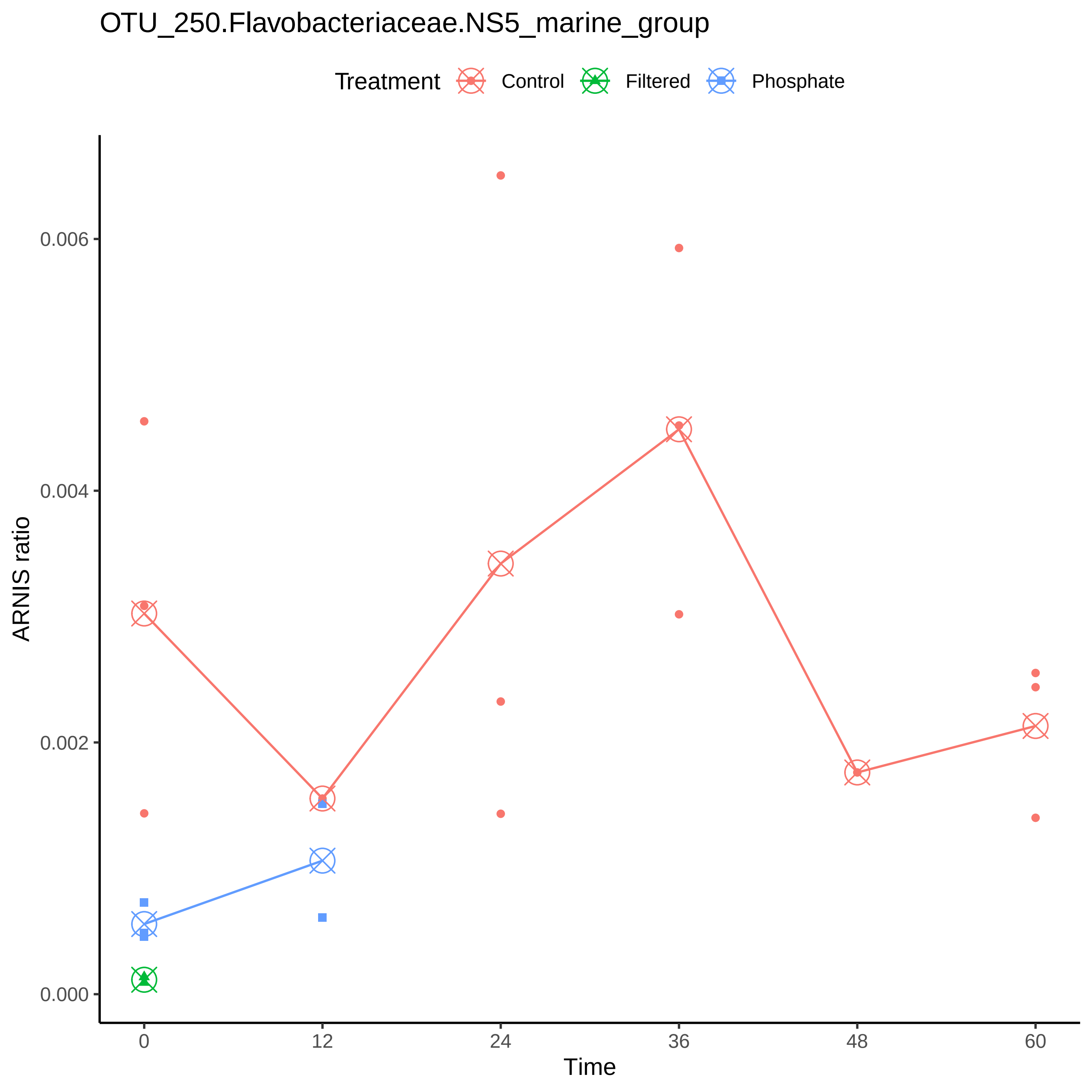

OTU_250.Flavobacteriaceae.NS5_marine_group
Treatment
Control
Filtered
Phosphate
ARNIS ratio
0.000
0.002
0.004
0.006
Time
0
12
24
36
48
60

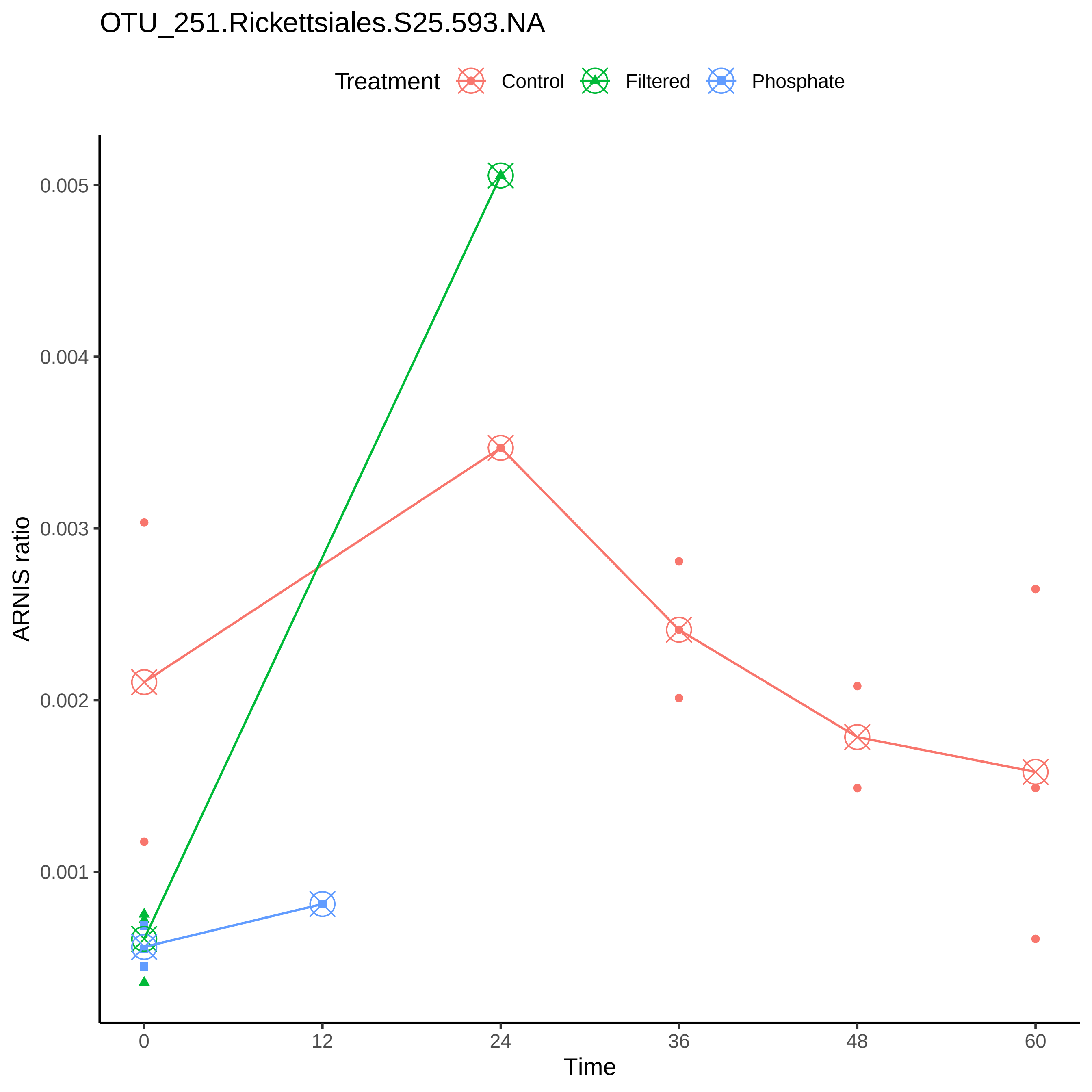
OTU_251.Rickettsiales.S25.593.NA
Treatment
Control
Filtered
Phosphate
ARNIS ratio
0.005
0.004
0.003
0.002
0.001
0
12
24
36
48
60
Time

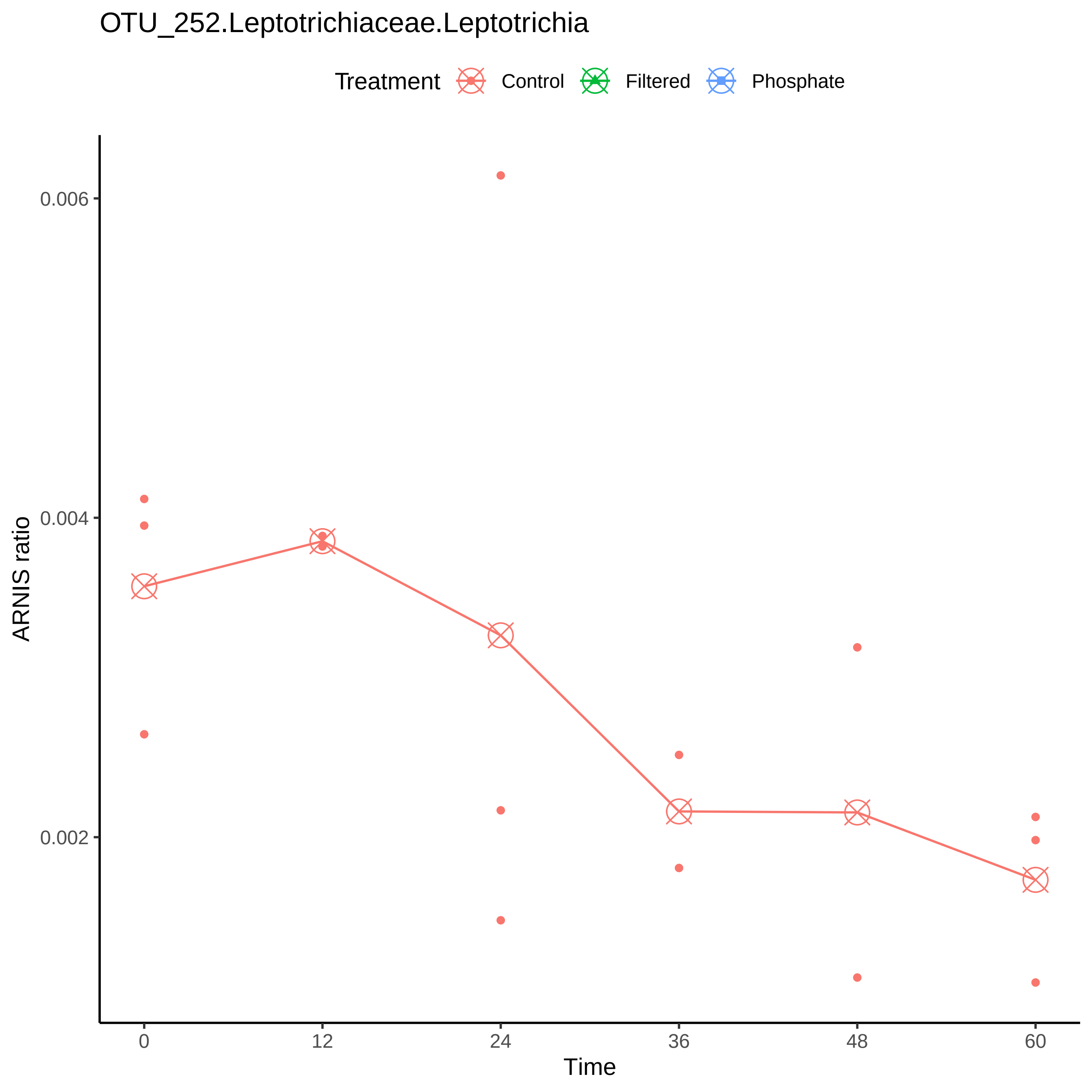

OTU_252.Leptotrichiaceae.Leptotrichia
Treatment
Control
Filtered
Phosphate
ARNIS ratio
0.006
0.004
0.002
0
12
24
36
48
60
Time

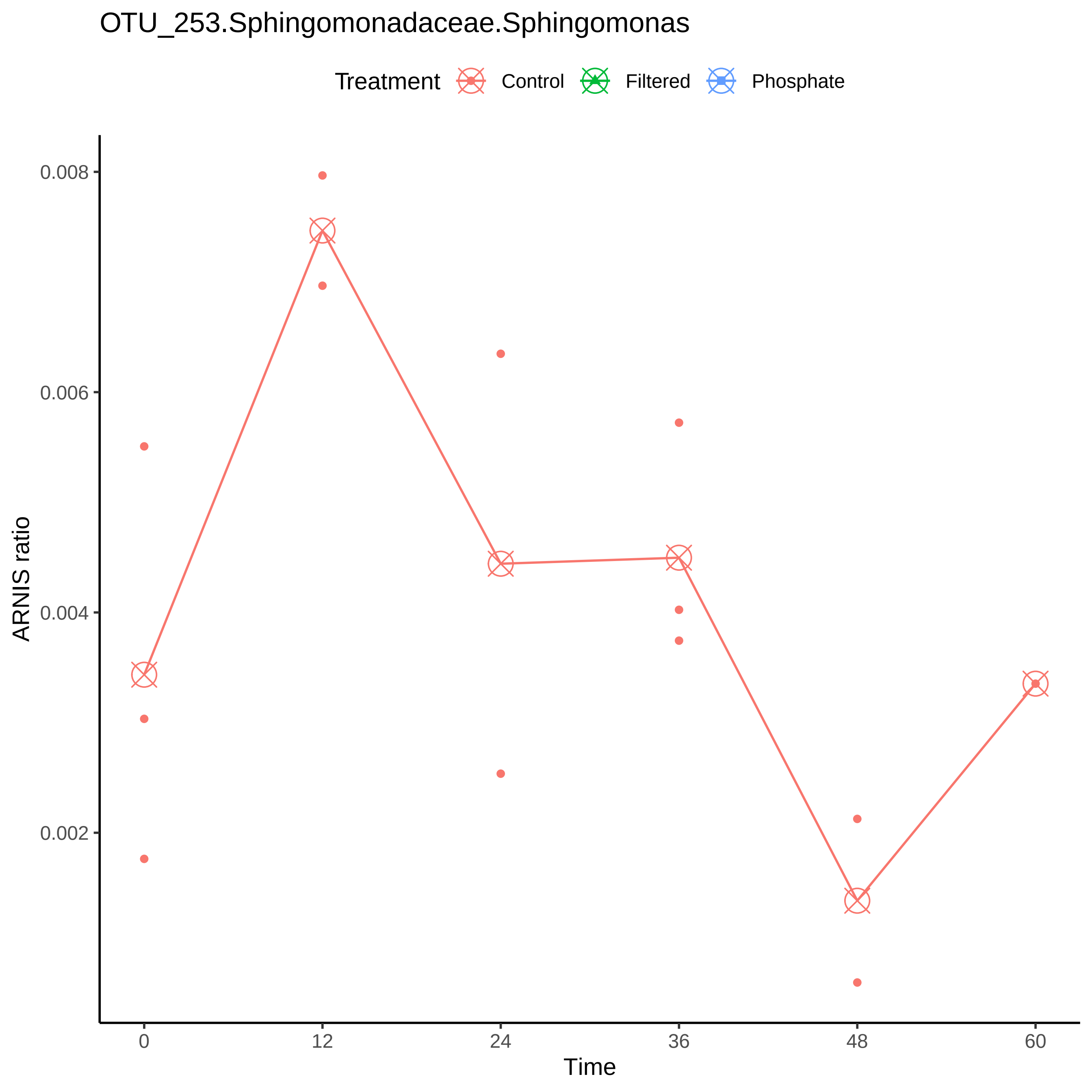
OTU_253.Sphingomonadaceae.Sphingomonas
Treatment
Control
Filtered
Phosphate
ARNIS ratio
0.008
0.006
0.004
0.002
Time
0
12
24
36
48
60

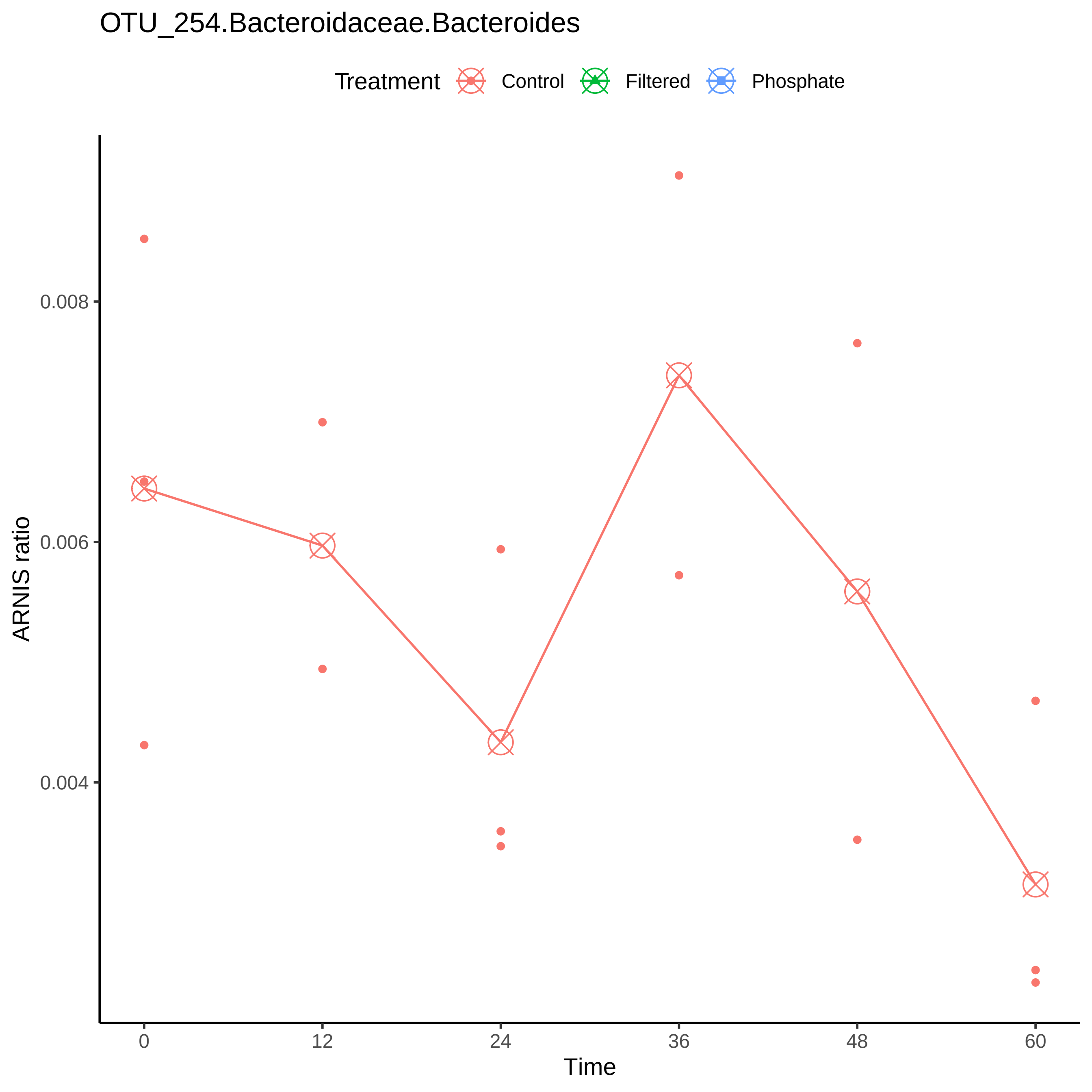

OTU_254.Bacteroidaceae.Bacteroides
Treatment
Control
Filtered
Phosphate
ARNIS ratio
0.008
0.006
0.004
0
12
24
36
48
60
Time

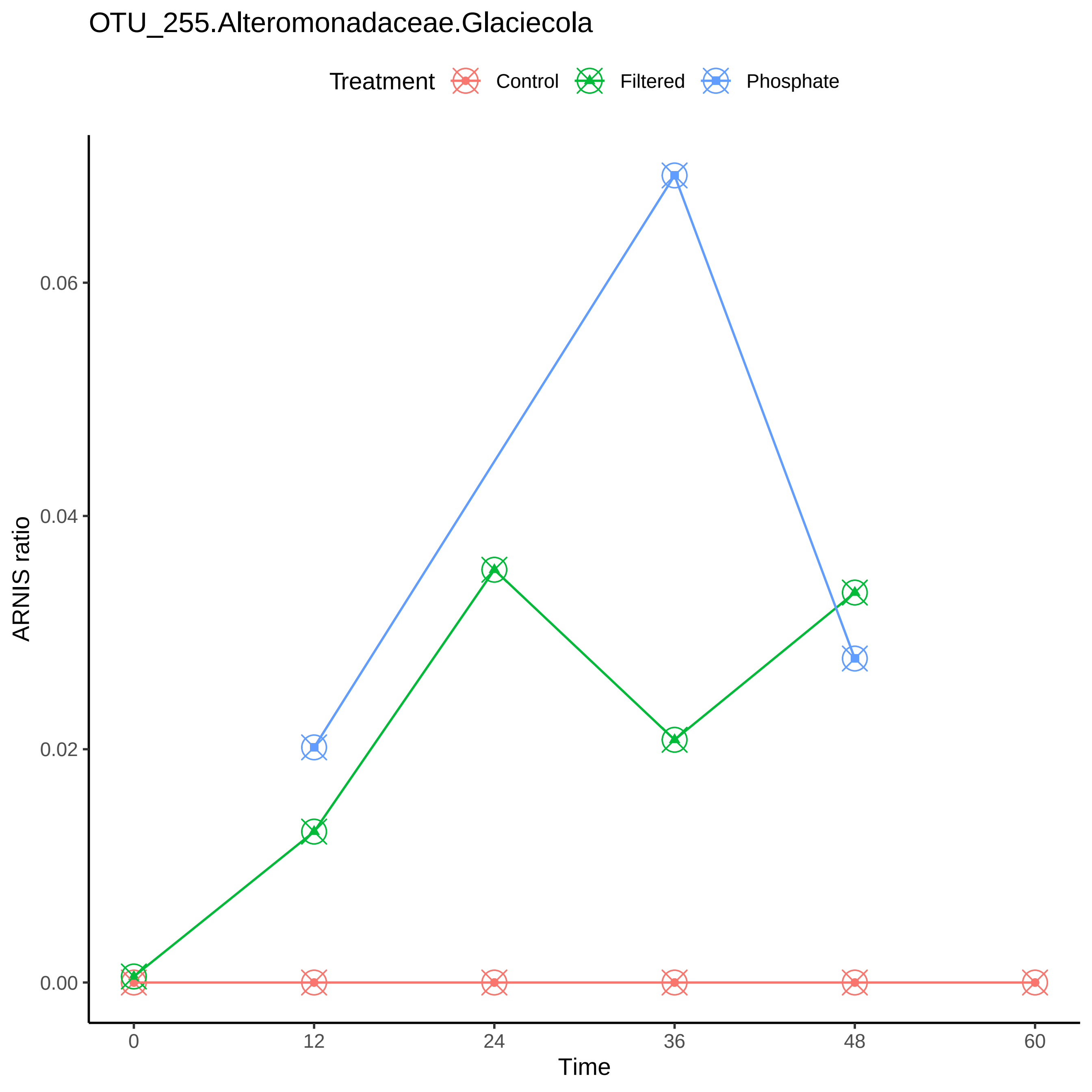
OTU_255.Alteromonadaceae.Glaciecola
Treatment
Control
Filtered
Phosphate
ARNIS ratio
0.00
0.02
0.04
0.06
Time
0
12
24
36
48
60

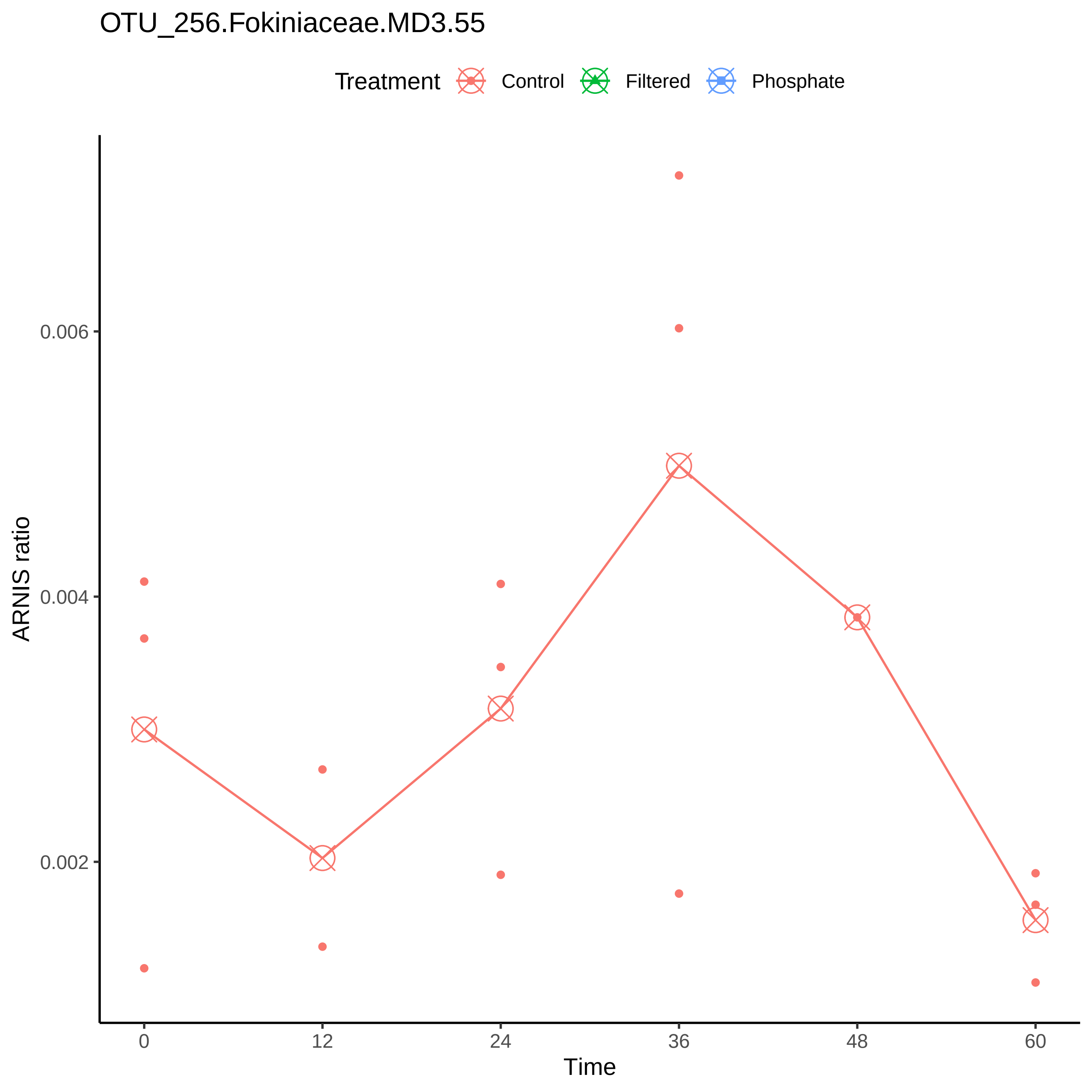
OTU_256.Fokiniaceae.MD3.55
Treatment
Control
Filtered
Phosphate
ARNIS ratio
0.006
0.004
0.002
0
12
24
36
48
60
Time

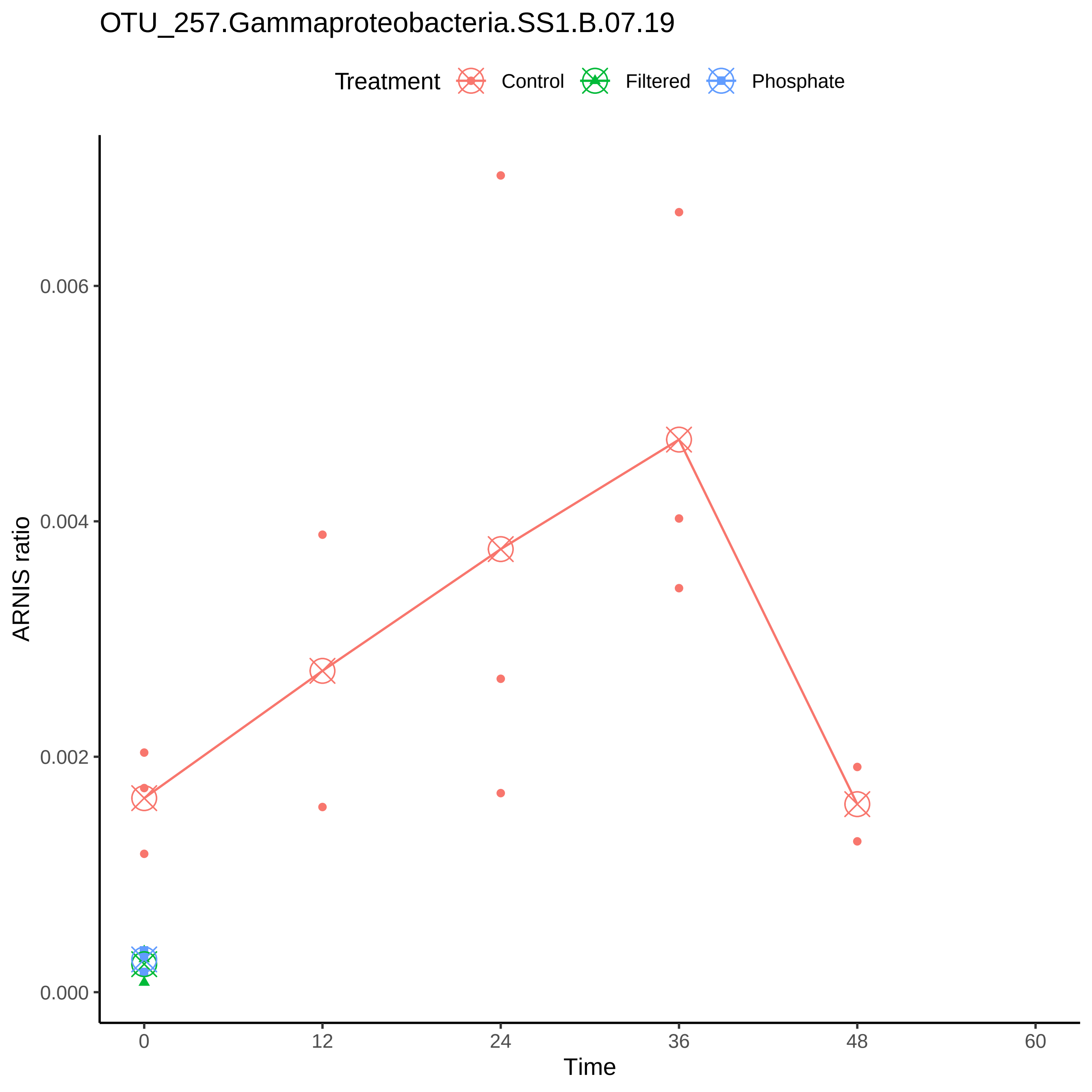

OTU_257.Gammaproteobacteria.SS1.B.07.19
Treatment
Control
Filtered
Phosphate
ARNIS ratio
0.006
0.004
0.002
0.000
Time
0
12
24
36
48
60

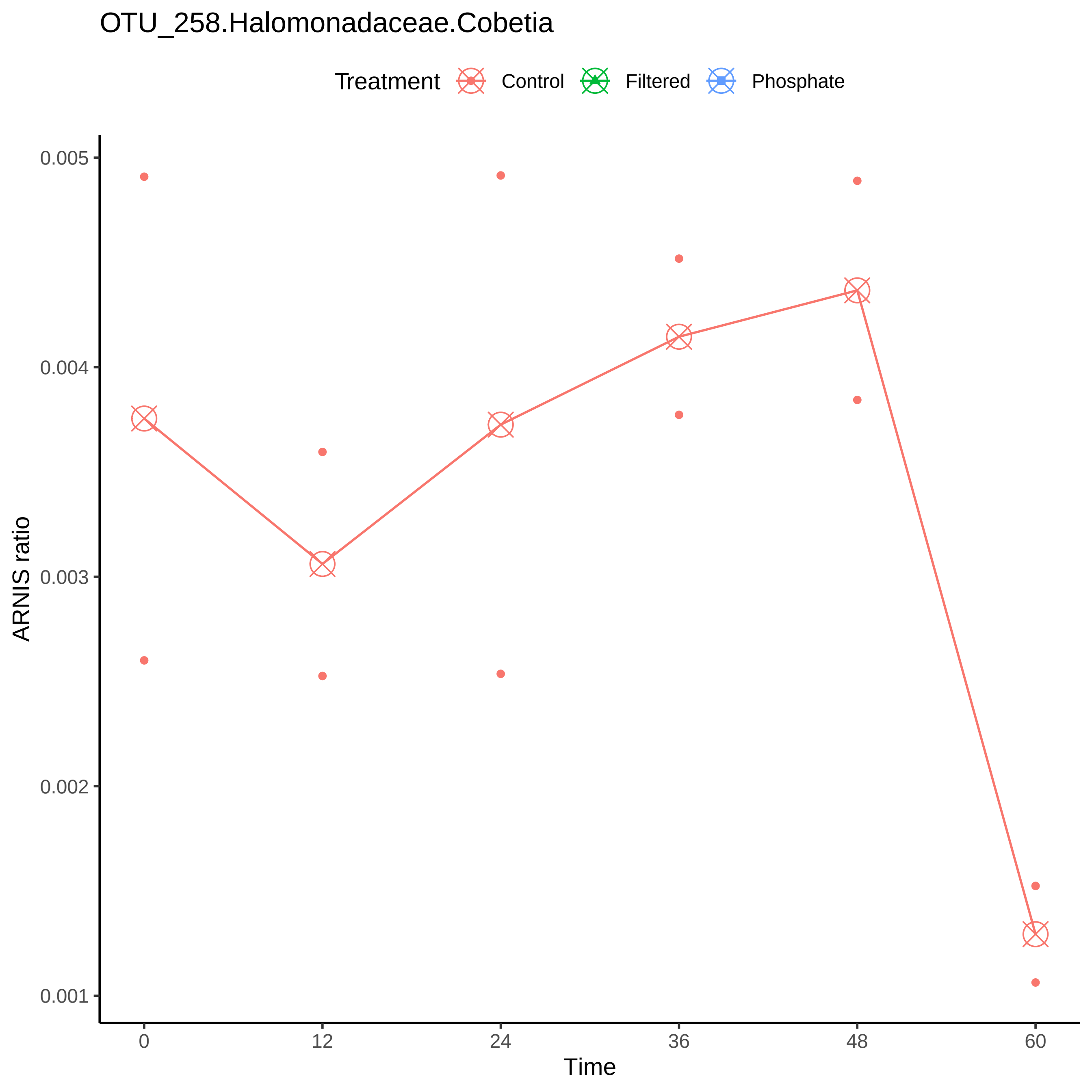

OTU_258.Halomonadaceae.Cobetia
Treatment
Control
Filtered
Phosphate
0.005
0.004
0.003
0.002
0.001
ARNIS ratio
0
12
24
36
48
60
Time

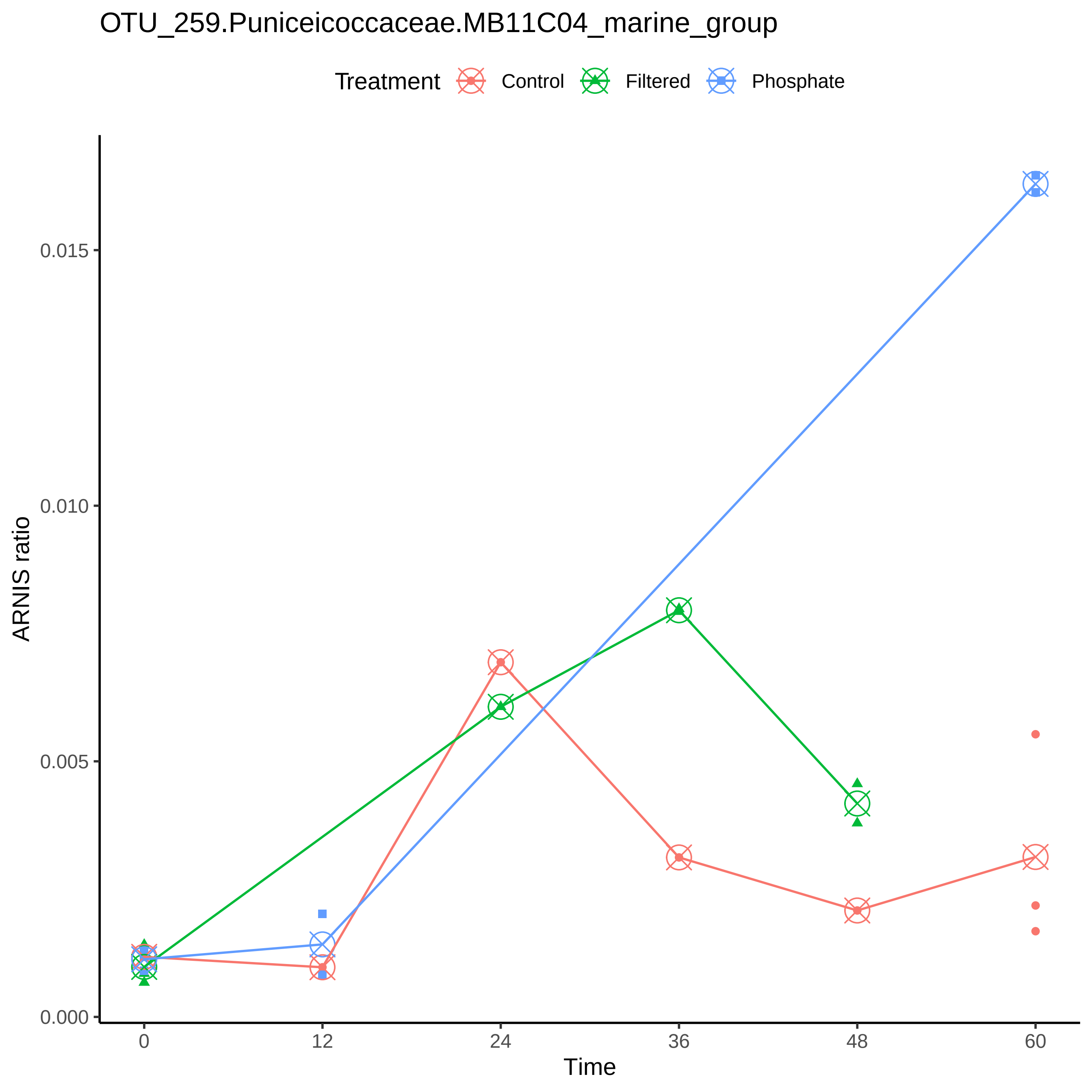

OTU_259.Puniceicoccaceae.MB11C04_marine_group
Treatment
Control
Filtered
Phosphate
ARNIS ratio
0.015
0.010
0.005
0.000
0
12
24
36
48
60
Time

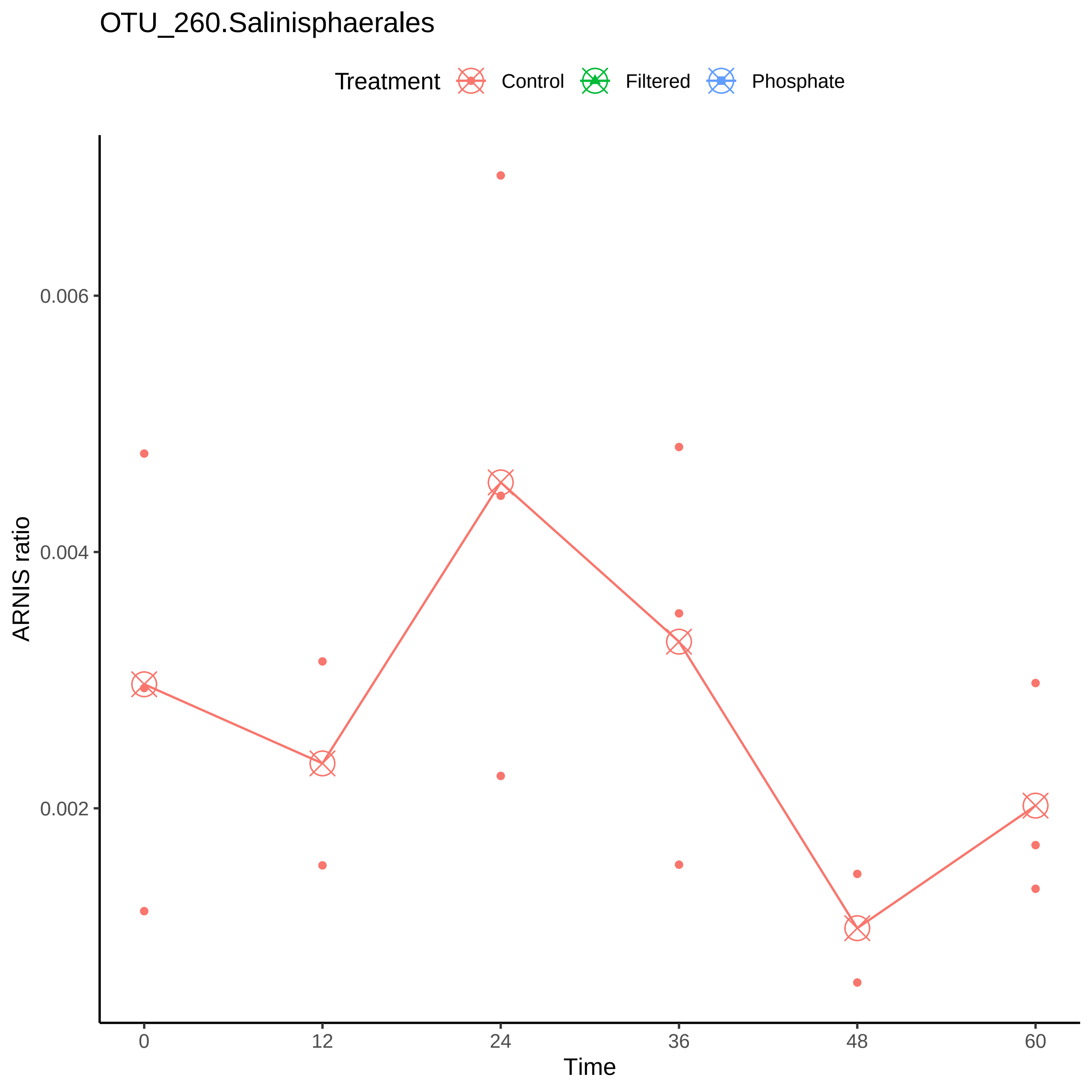
OTU_260.Salinisphaerales
Treatment
Control
Filtered
Phosphate
ARNIS ratio
0.006
0.004
0.002
Time
0
12
24
36
48
60

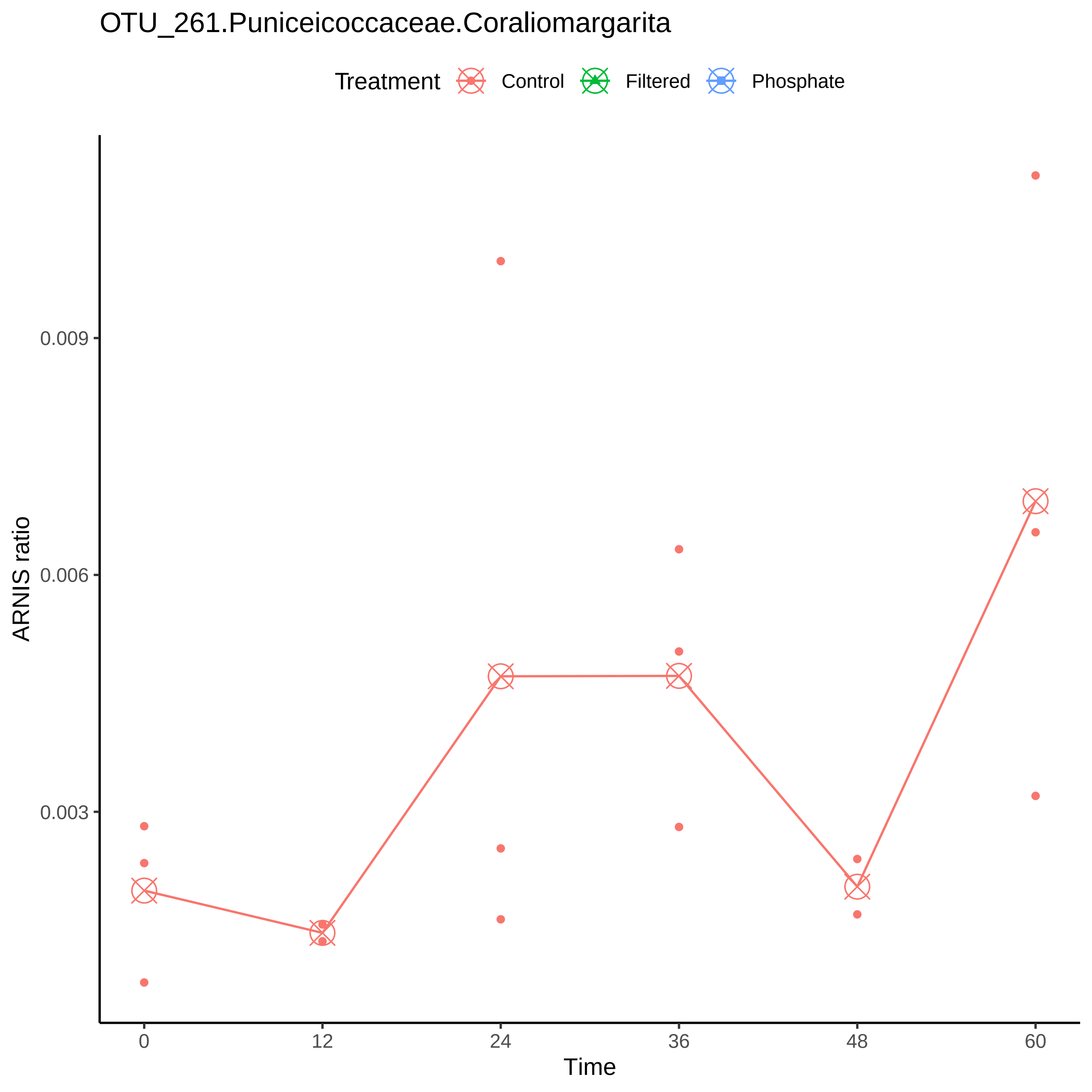
OTU_261.Puniceicoccaceae.Coraliomargarita
Treatment
Control
Filtered
Phosphate
ARNIS ratio
0.009
0.006
0.003
0
12
24
36
48
60
Time

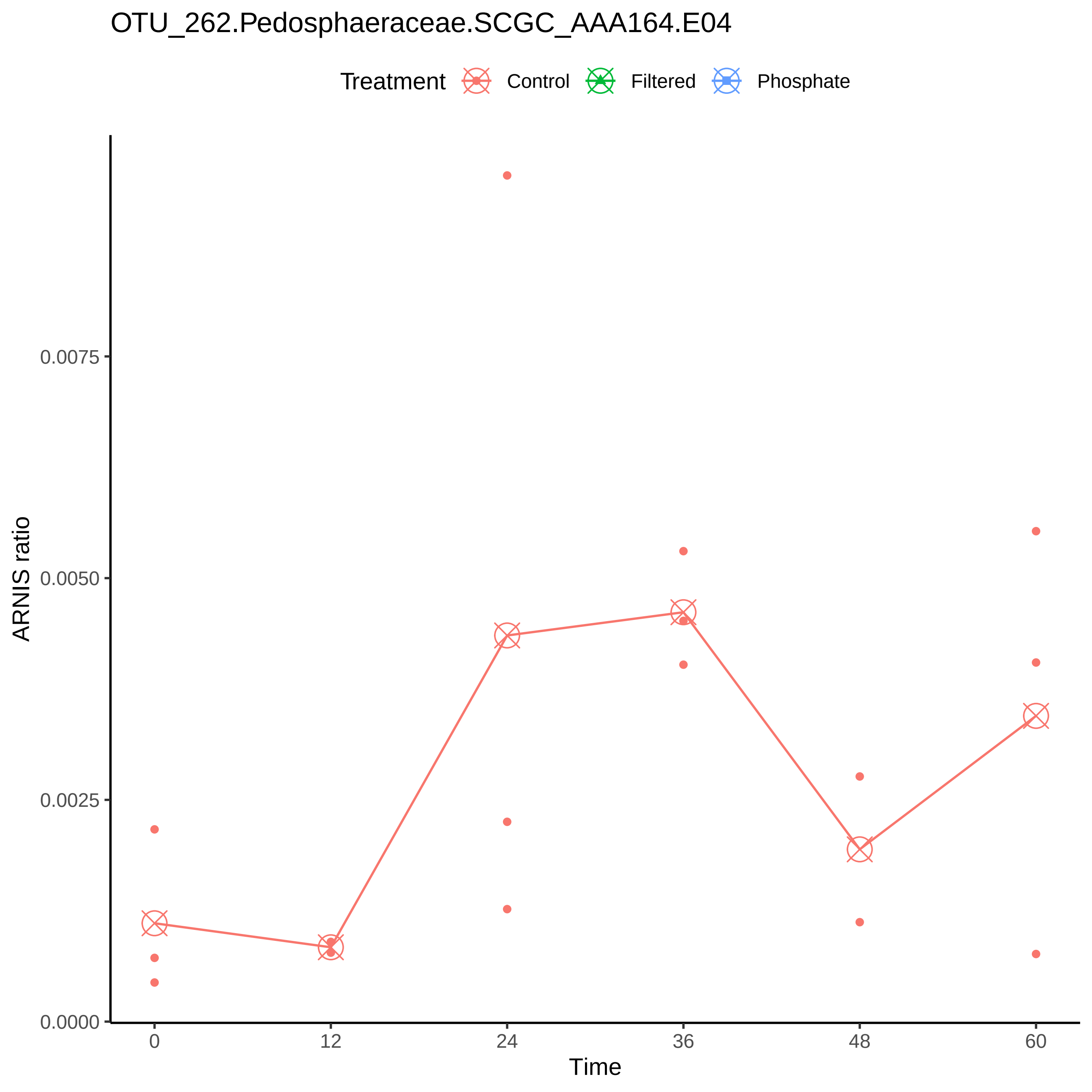

OTU_262.Pedosphaeraceae.SCGC_AAA164.E04
Treatment
Control
Filtered
Phosphate
ARNIS ratio
0.0000
0.0025
0.0050
0.0075
Time
0
12
24
36
48
60

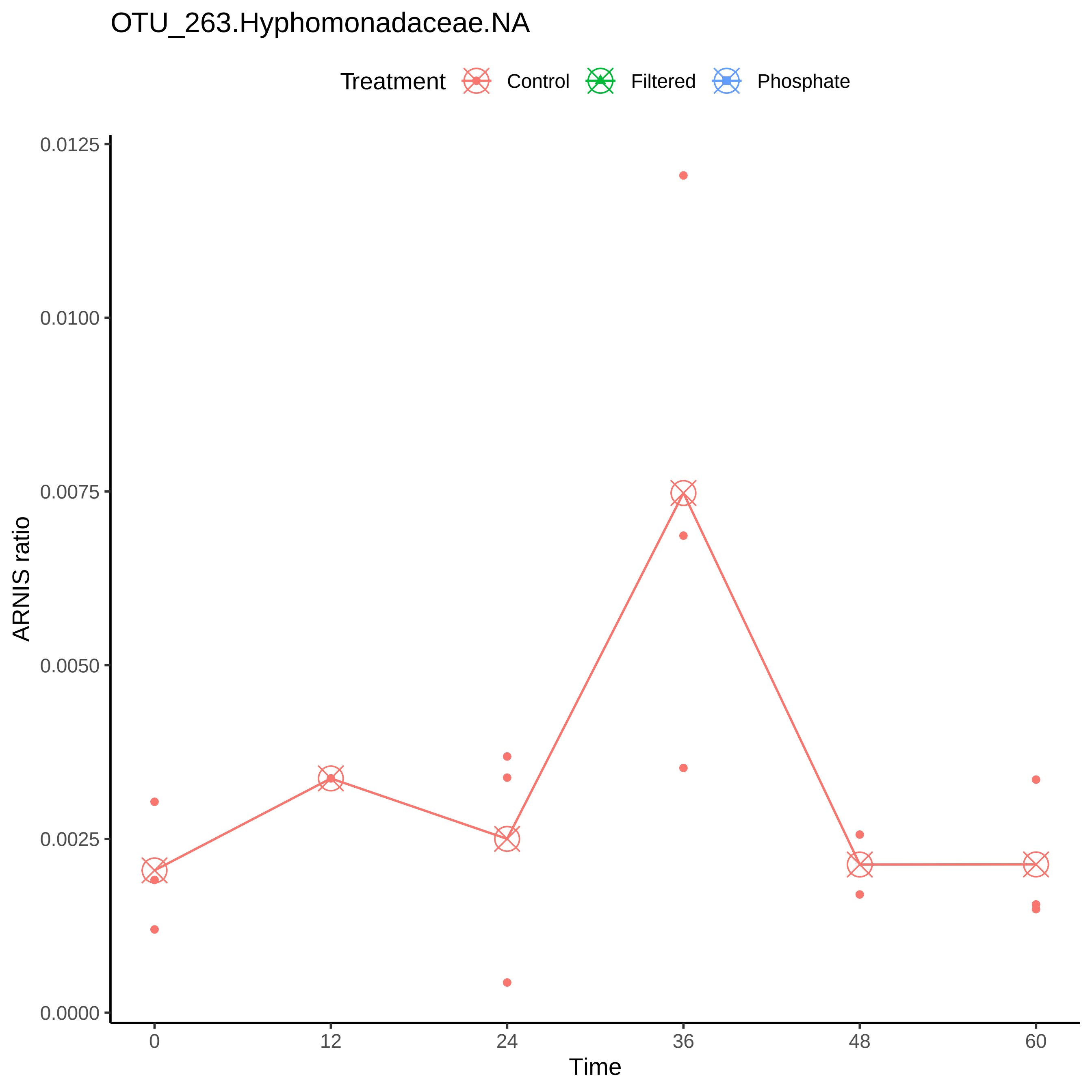

OTU_263.Hyphomonadaceae.NA
Treatment
Control
Filtered
Phosphate
0.0125
0.0100
0.0075
0.0050
0.0025
0.0000
ARNIS ratio
0
12
24
36
48
60
Time

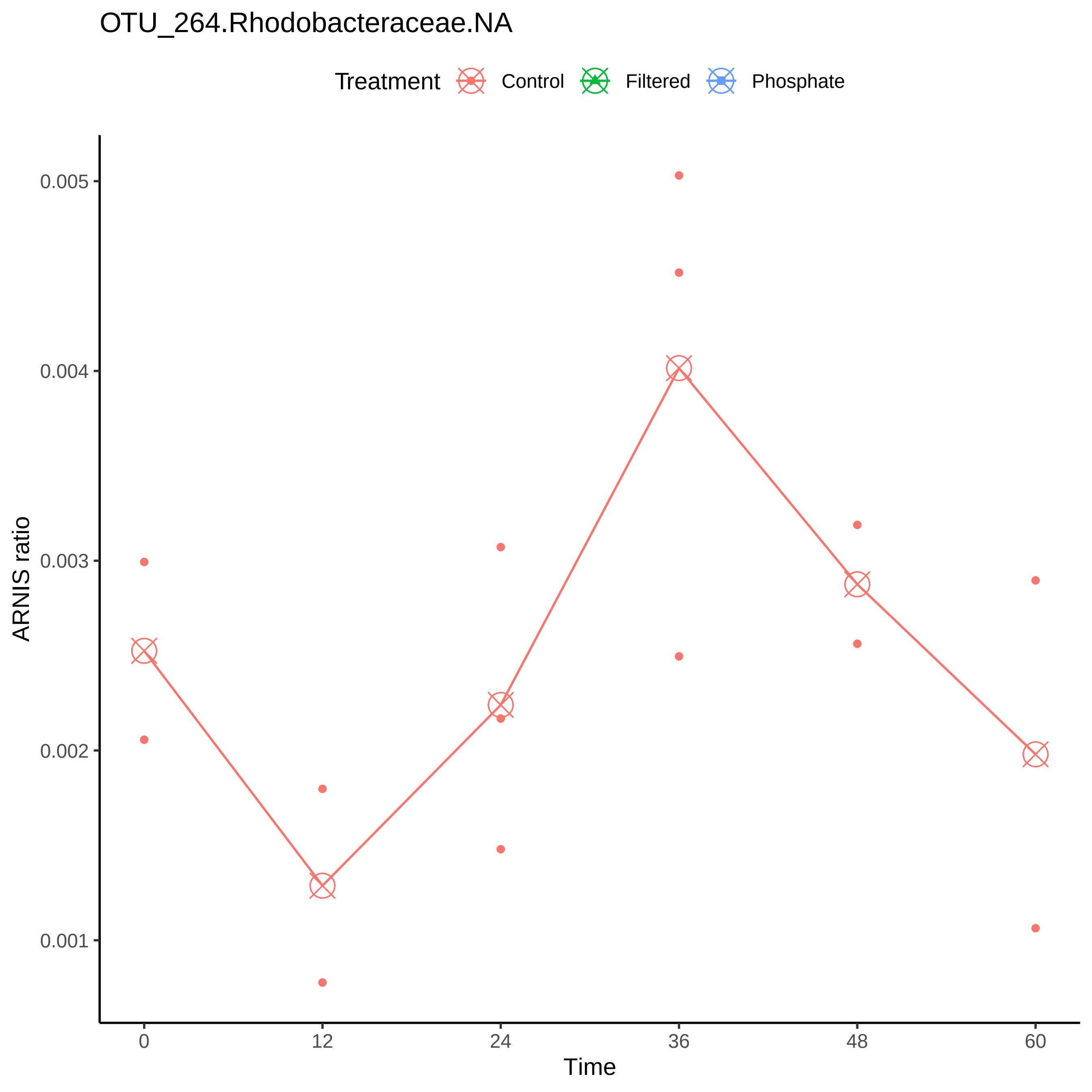

OTU_264.Rhodobacteraceae.NA
Treatment
Control
Filtered
Phosphate
0.005
0.004
0.003
0.002
0.001
ARNIS ratio
0
12
24
36
48
60
Time

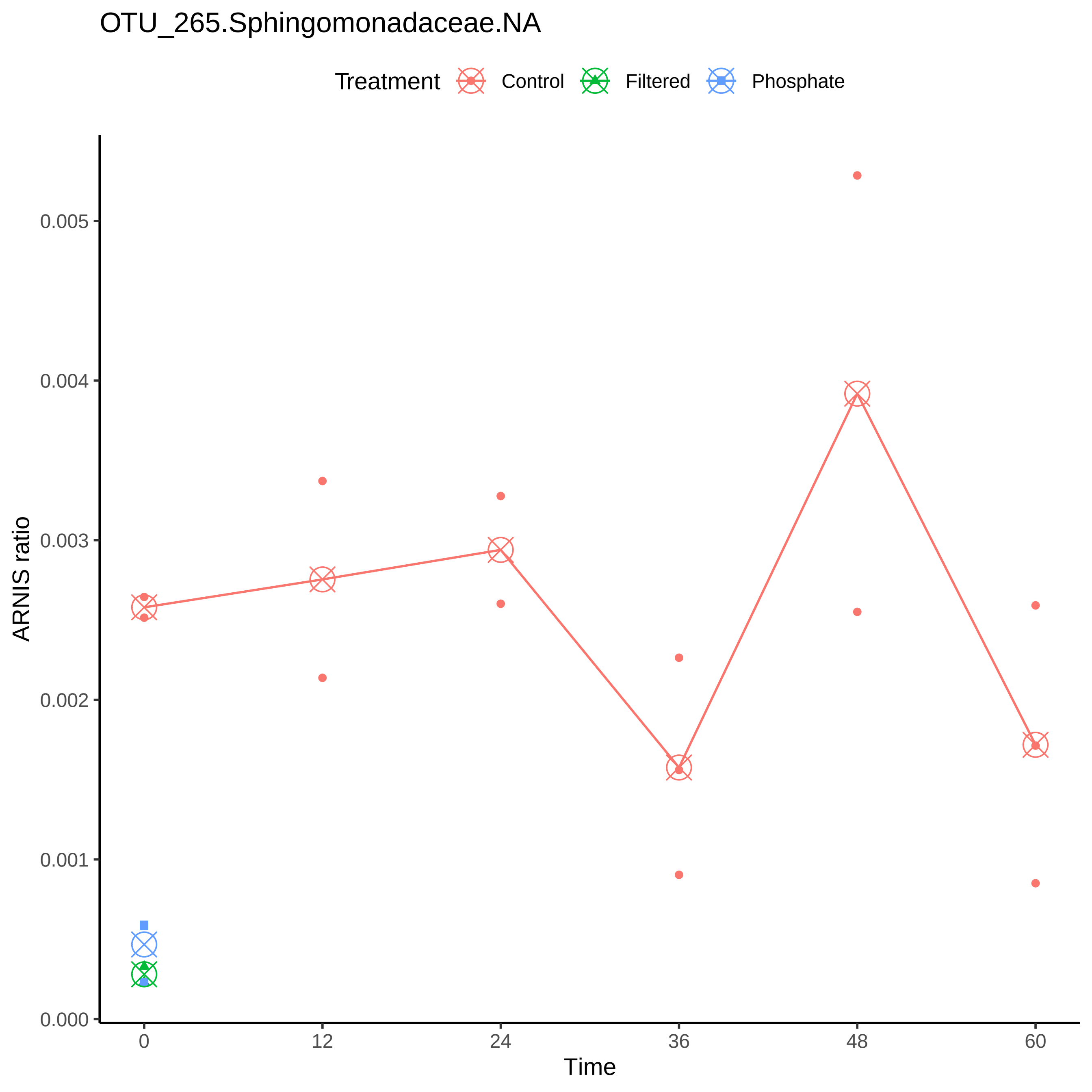
OTU_265.Sphingomonadaceae.NA
Treatment
Control
Filtered
Phosphate
ARNIS ratio
0.000
0.001
0.002
0.003
0.004
0.005
Time
0
12
24
36
48
60

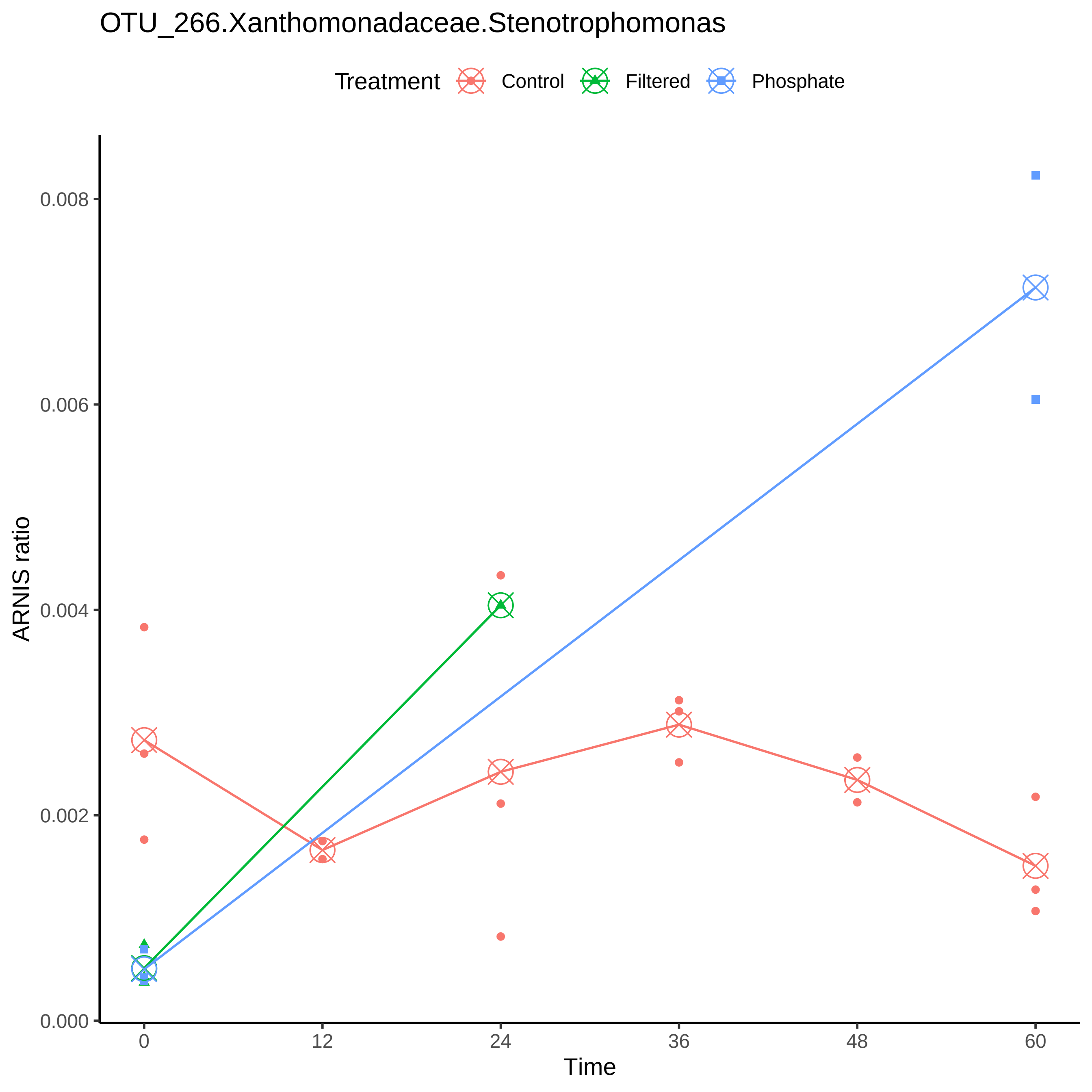

OTU_266.Xanthomonadaceae.Stenotrophomonas
Treatment
Control
Filtered
Phosphate
ARNIS ratio
0.000
0.002
0.004
0.006
0.008
0
12
24
36
48
60
Time

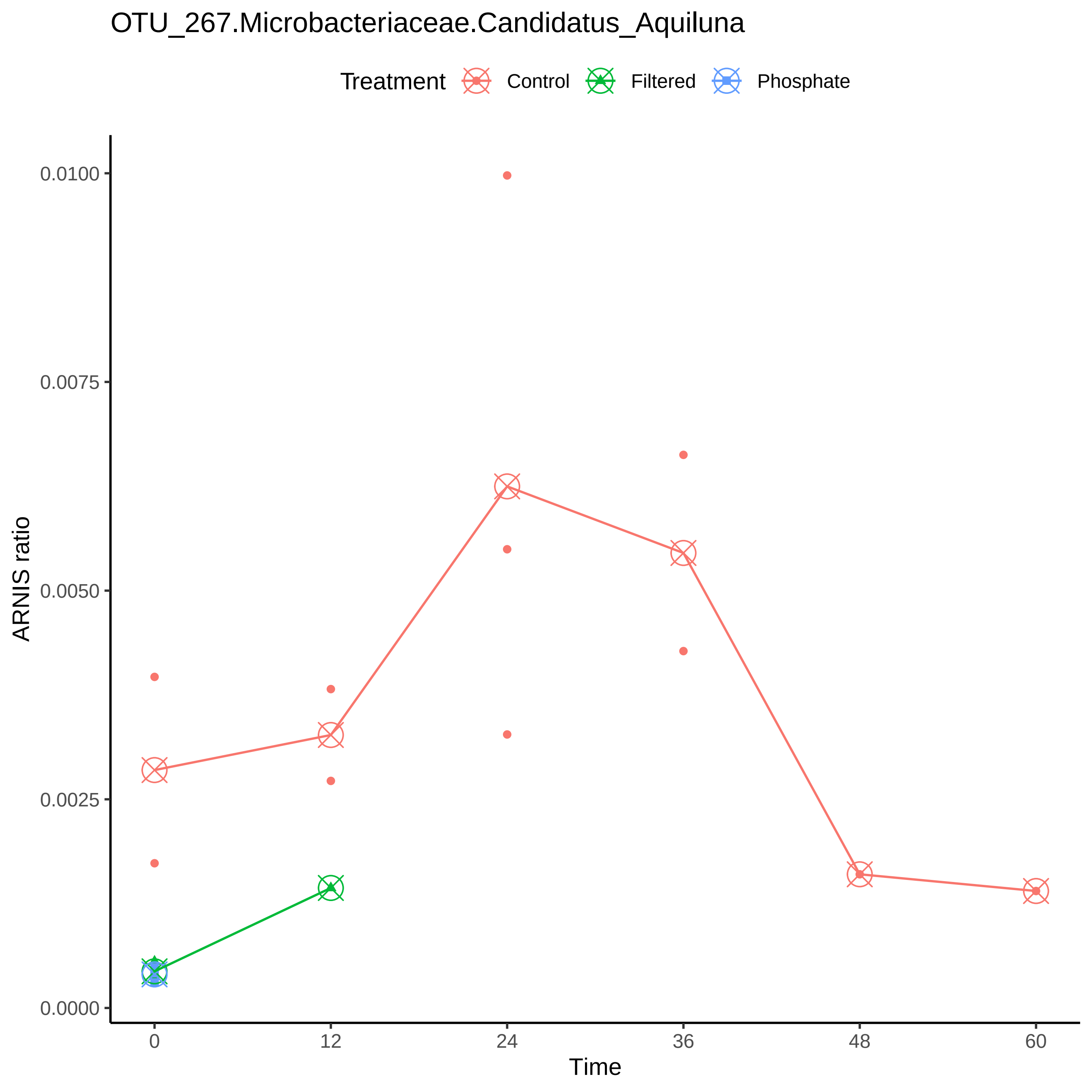

OTU_267.Microbacteriaceae.Candidatus_Aquiluna
Treatment
Control
Filtered
Phosphate
0.0100
0.0075
0.0050
0.0025
0.0000
ARNIS ratio
0
12
24
36
48
60
Time

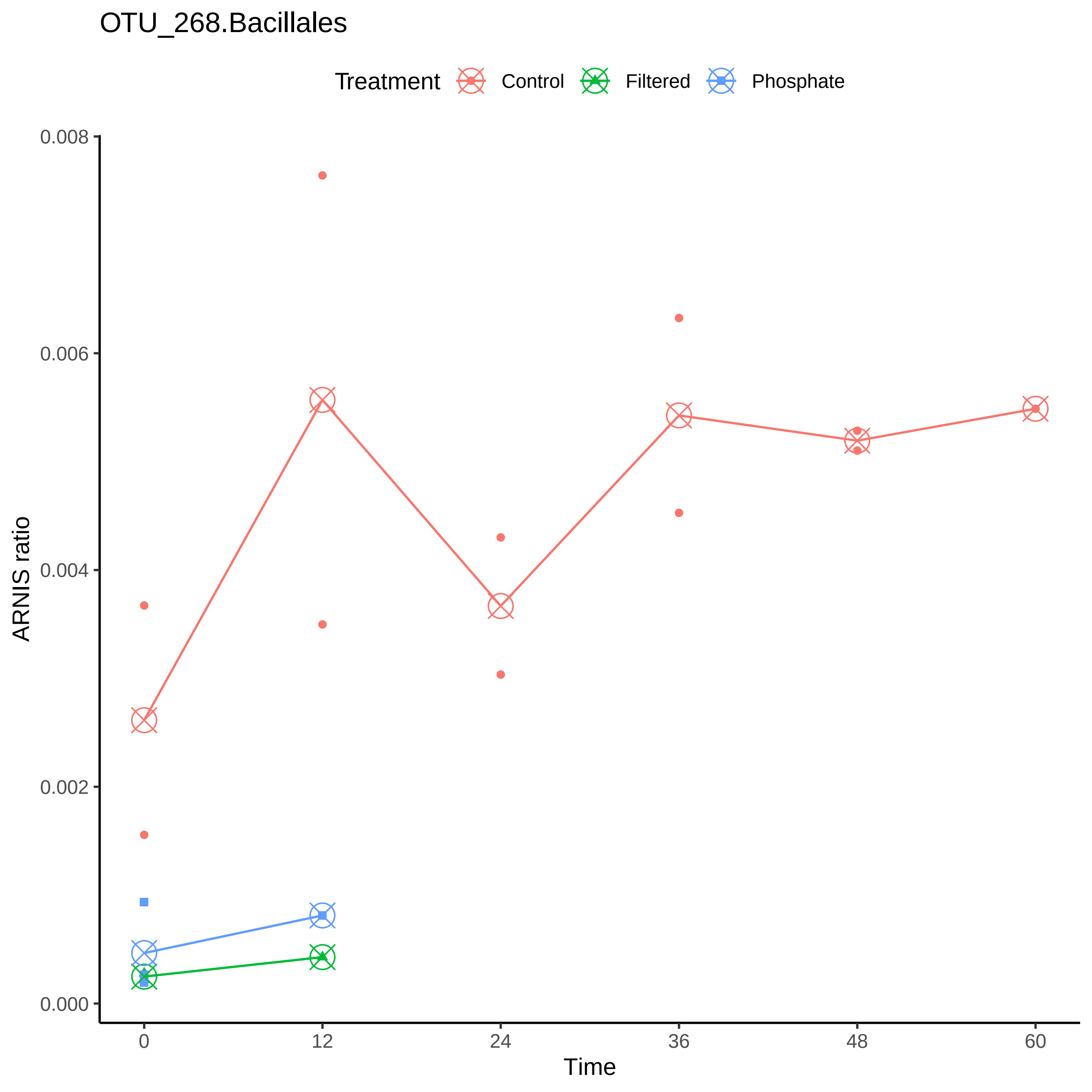
OTU_268.Bacillales
Treatment
Control
Filtered
Phosphate
0.008
0.006
0.004
0.002
0.000
ARNIS ratio
0
12
24
36
48
60
Time

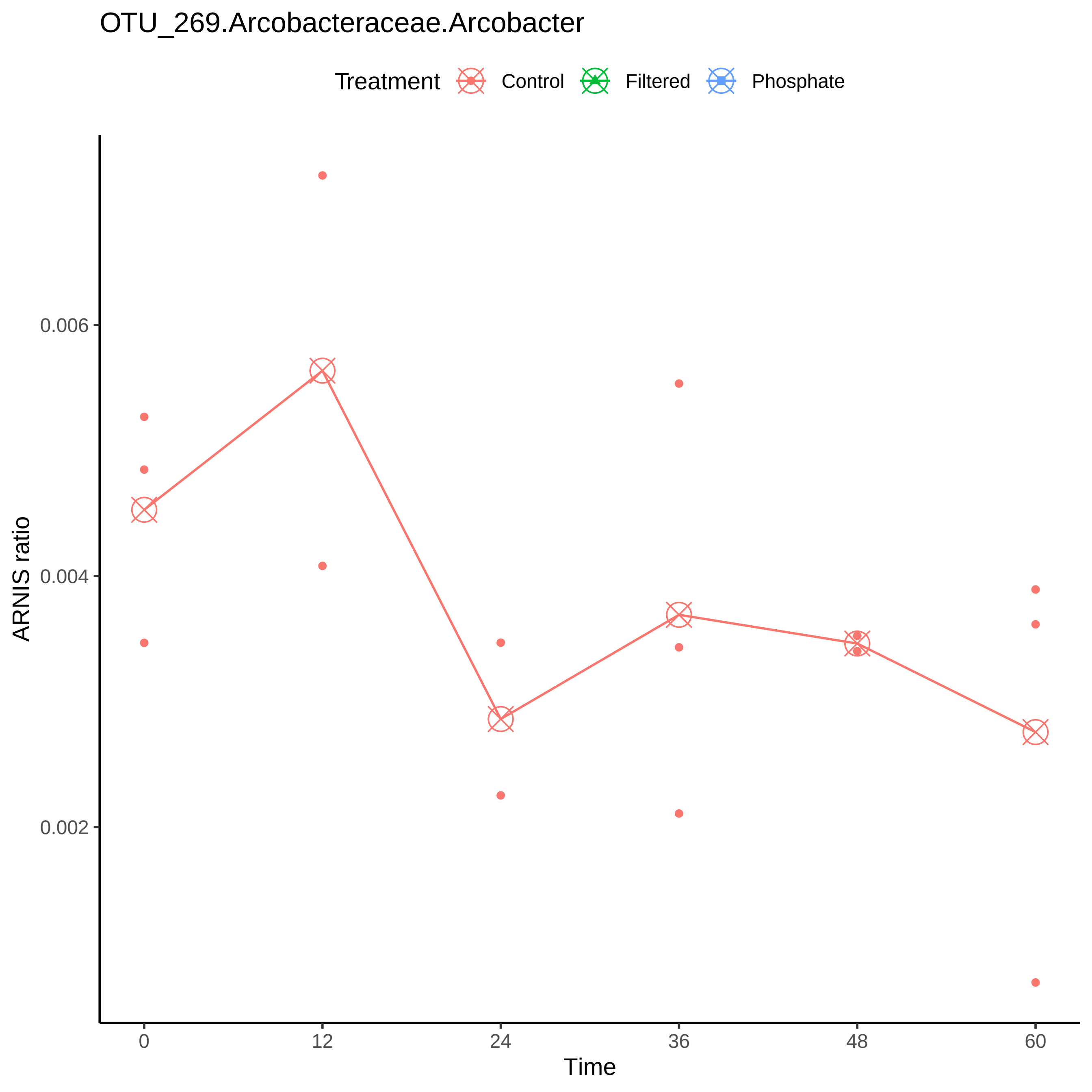

OTU_269.Arcobacteraceae.Arcobacter
Treatment
Control
Filtered
Phosphate
ARNIS ratio
0.006
0.004
0.002
0
12
24
36
48
60
Time

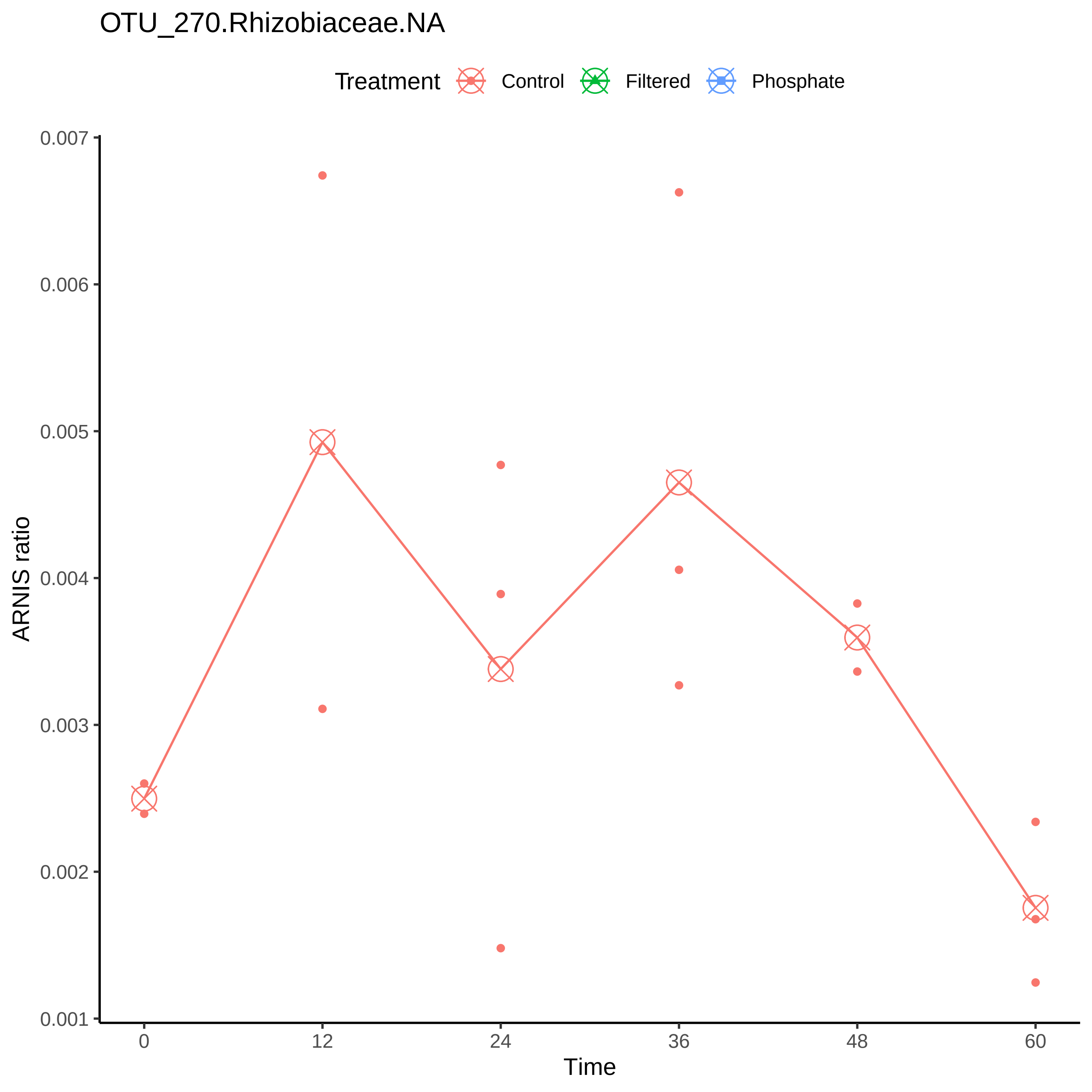

OTU_270.Rhizobiaceae.NA
Treatment
Control
Filtered
Phosphate
0.007
0.006
0.005
0.004
0.003
0.002
0.001
ARNIS ratio
0
12
24
36
48
60
Time

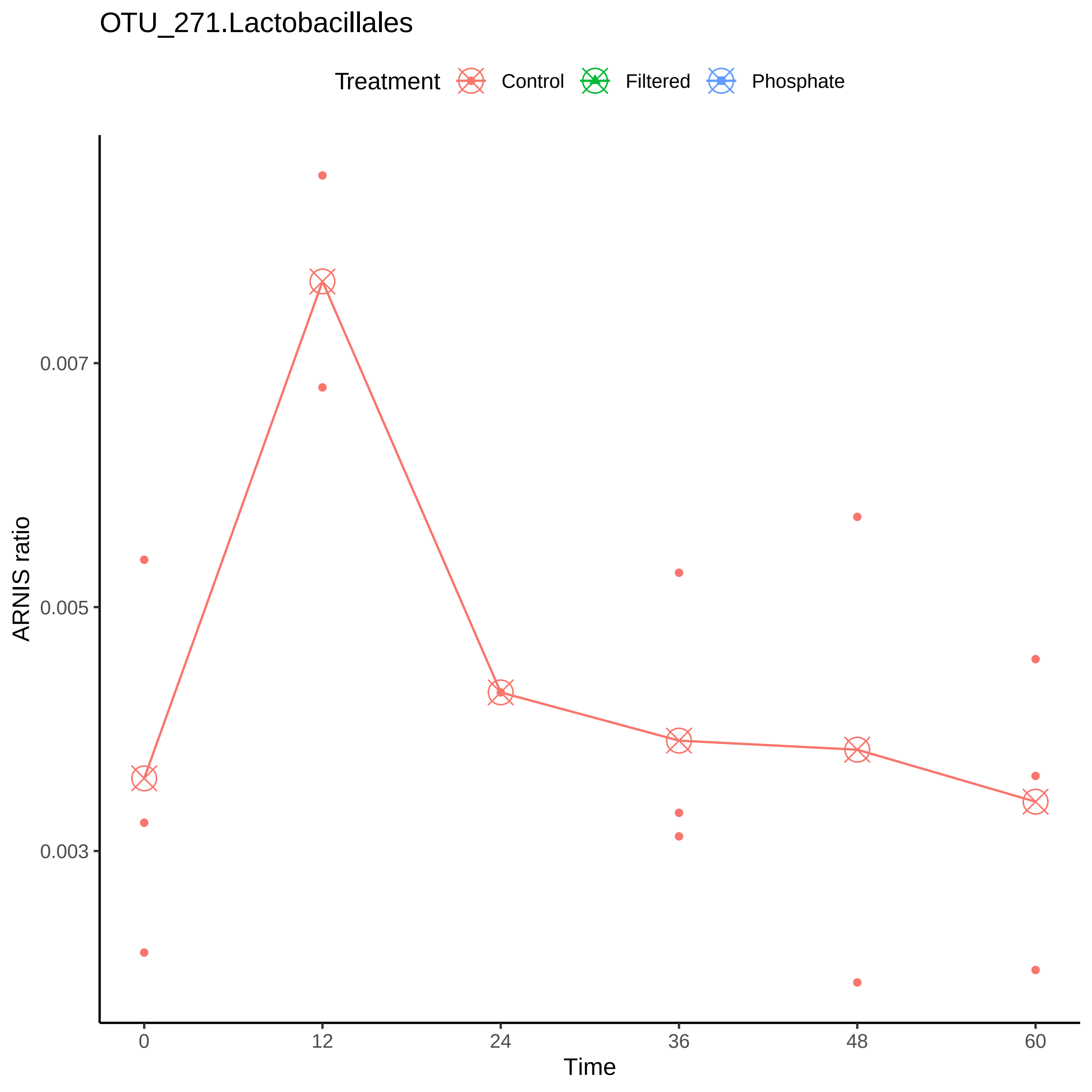
OTU_271.Lactobacillales
Treatment
Control
Filtered
Phosphate
ARNIS ratio
0.007
0.005
0.003
0
12
24
36
48
60
Time

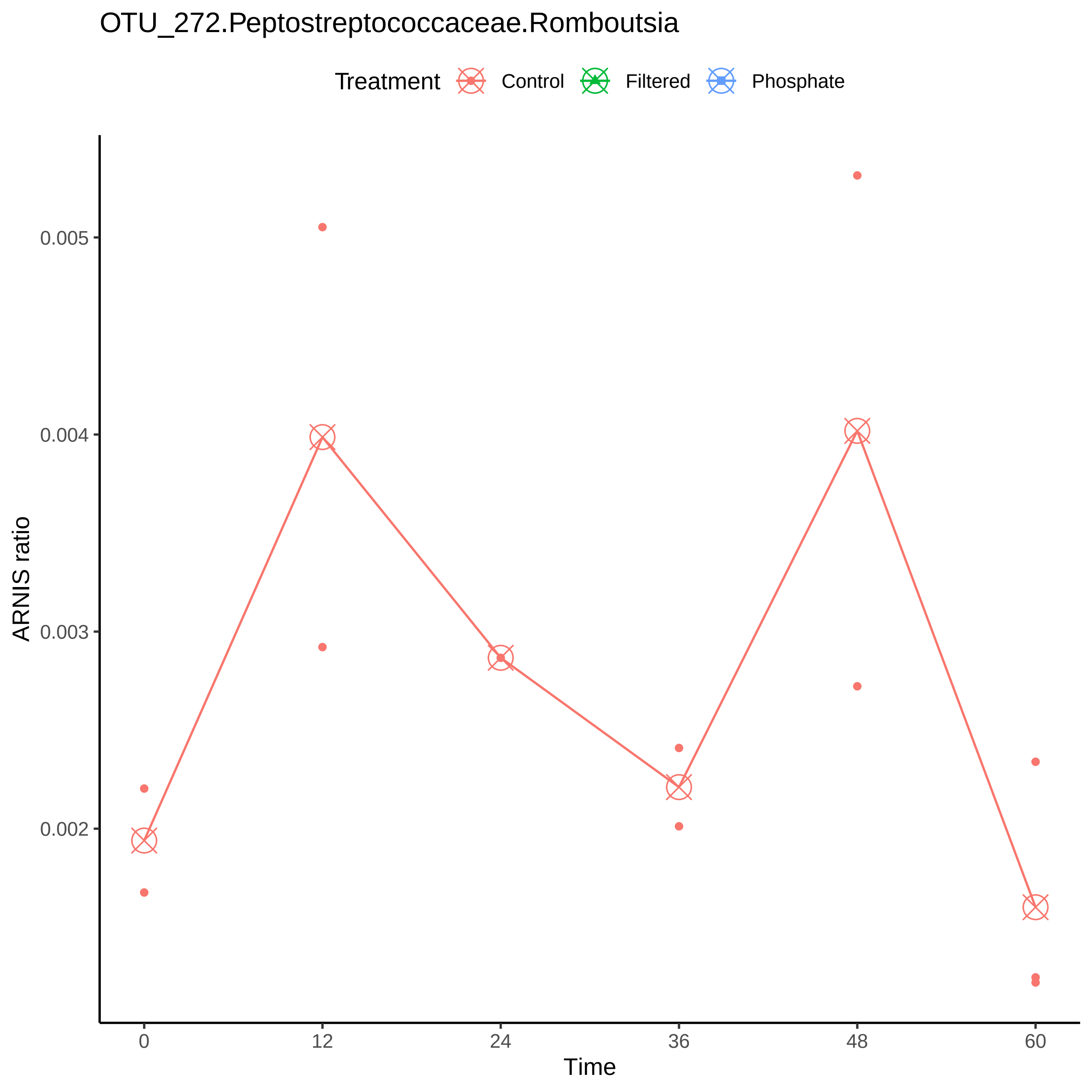

OTU_272.Peptostreptococcaceae.Romboutsia
Treatment
Control
Filtered
Phosphate
ARNIS ratio
0.005
0.004
0.003
0.002
0
12
24
36
48
60
Time

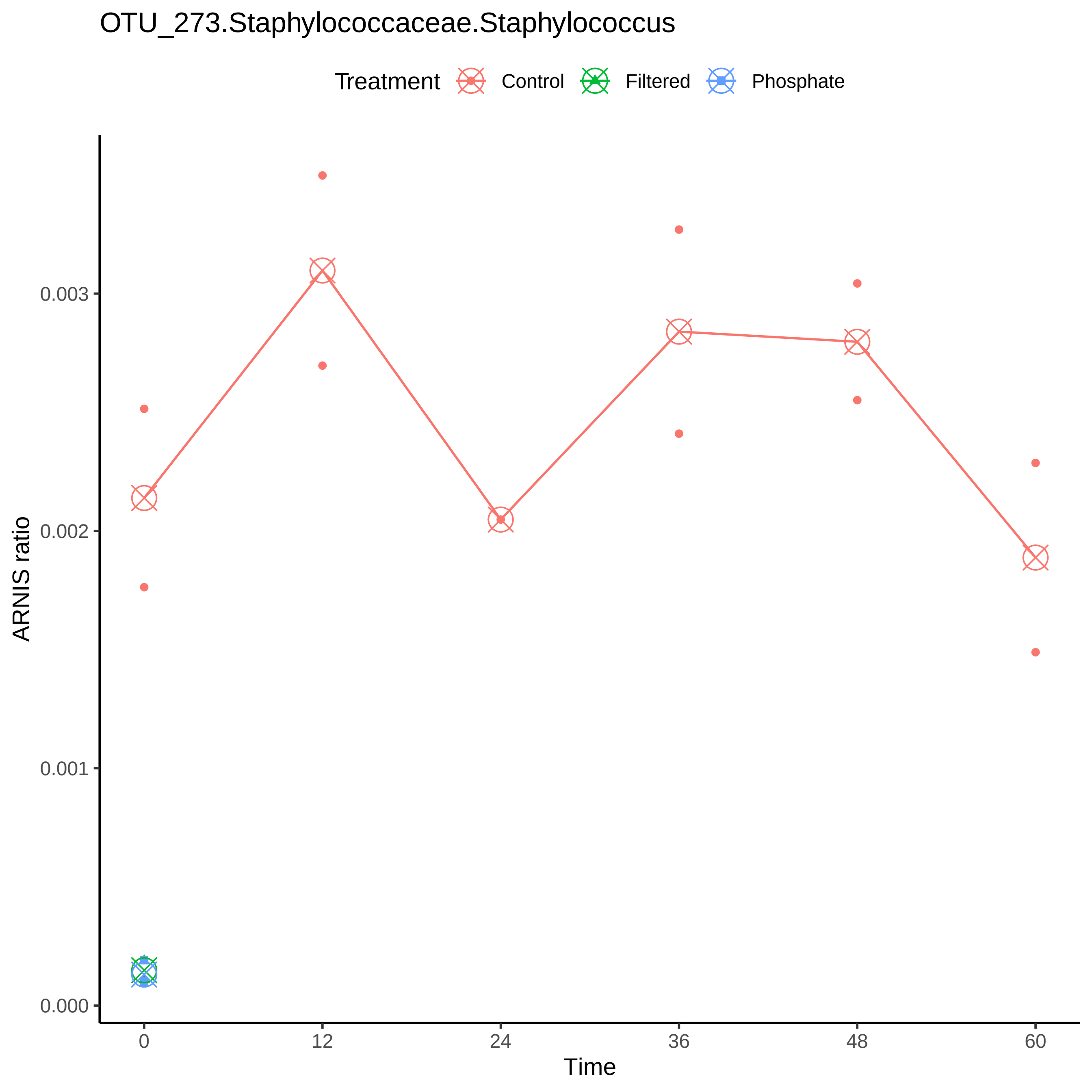

OTU_273.Staphylococcaceae.Staphylococcus
Treatment
Control
Filtered
Phosphate
ARNIS ratio
0.003
0.002
0.001
0.000
Time
0
12
24
36
48
60

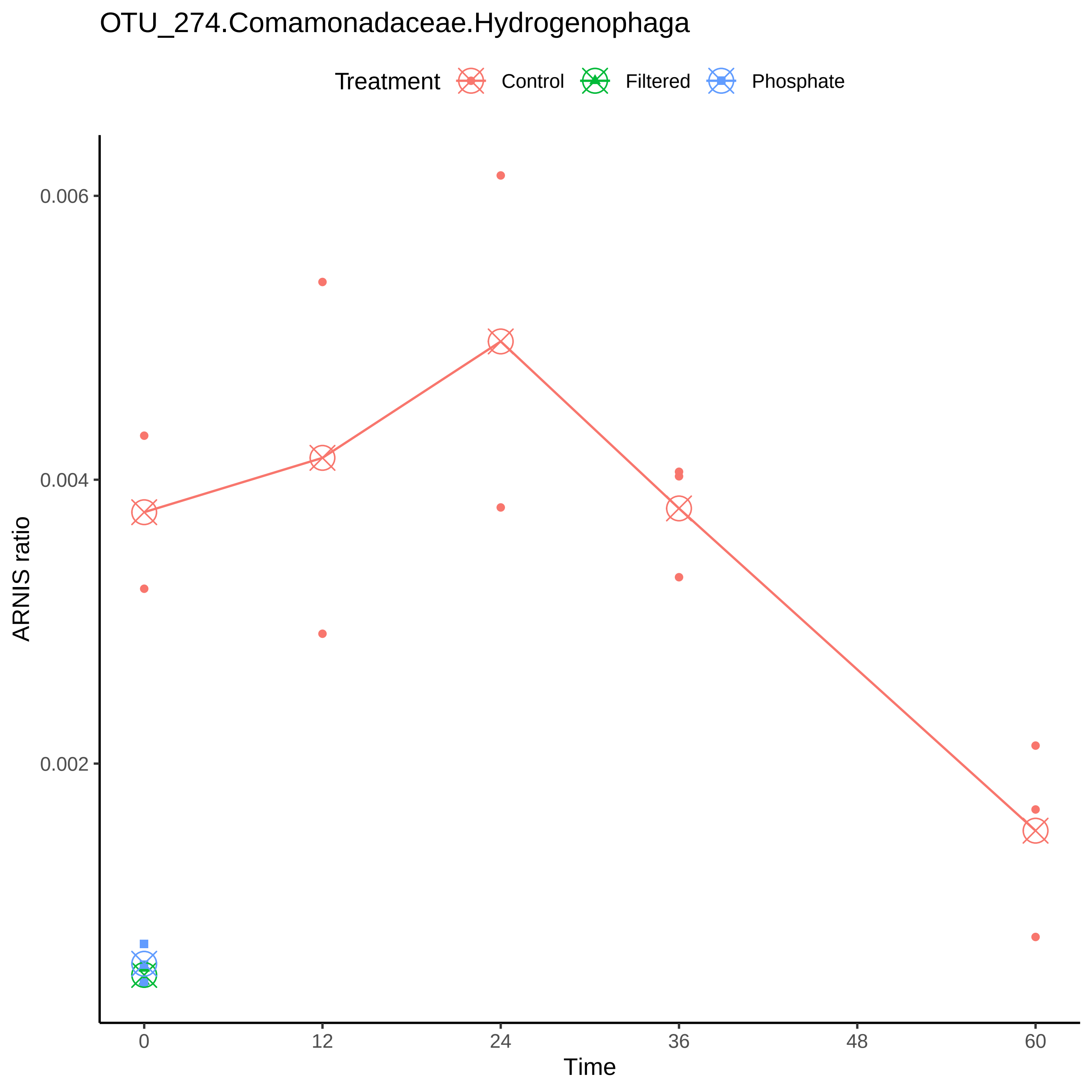
OTU_274.Comamonadaceae.Hydrogenophaga
Treatment
Control
Filtered
Phosphate
ARNIS ratio
0.006
0.004
0.002
0
12
24
36
48
60
Time

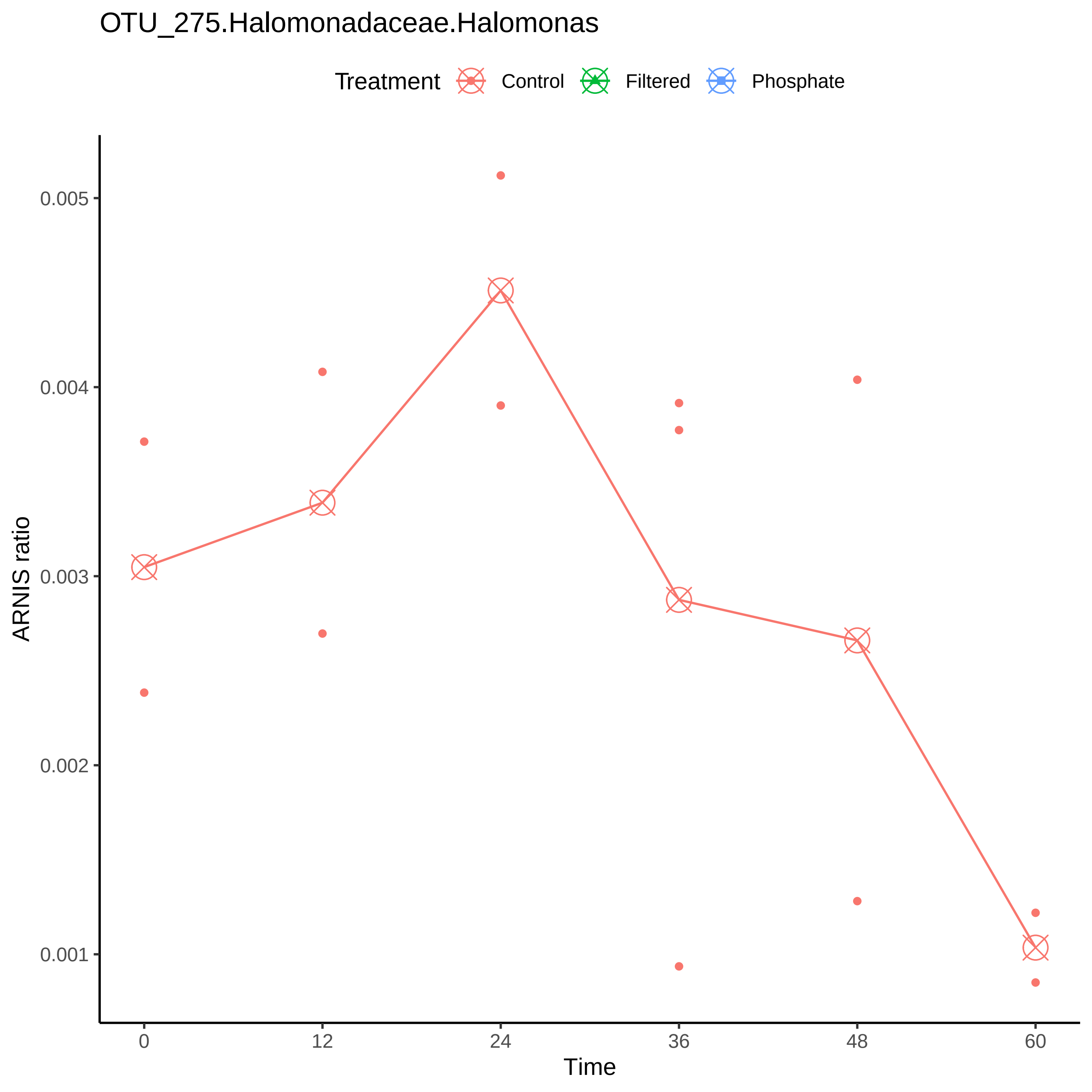
OTU_275.Halomonadaceae.Halomonas
Treatment
Control
Filtered
Phosphate
0.005
0.004
0.003
0.002
0.001
ARNIS ratio
0
12
24
36
48
60
Time

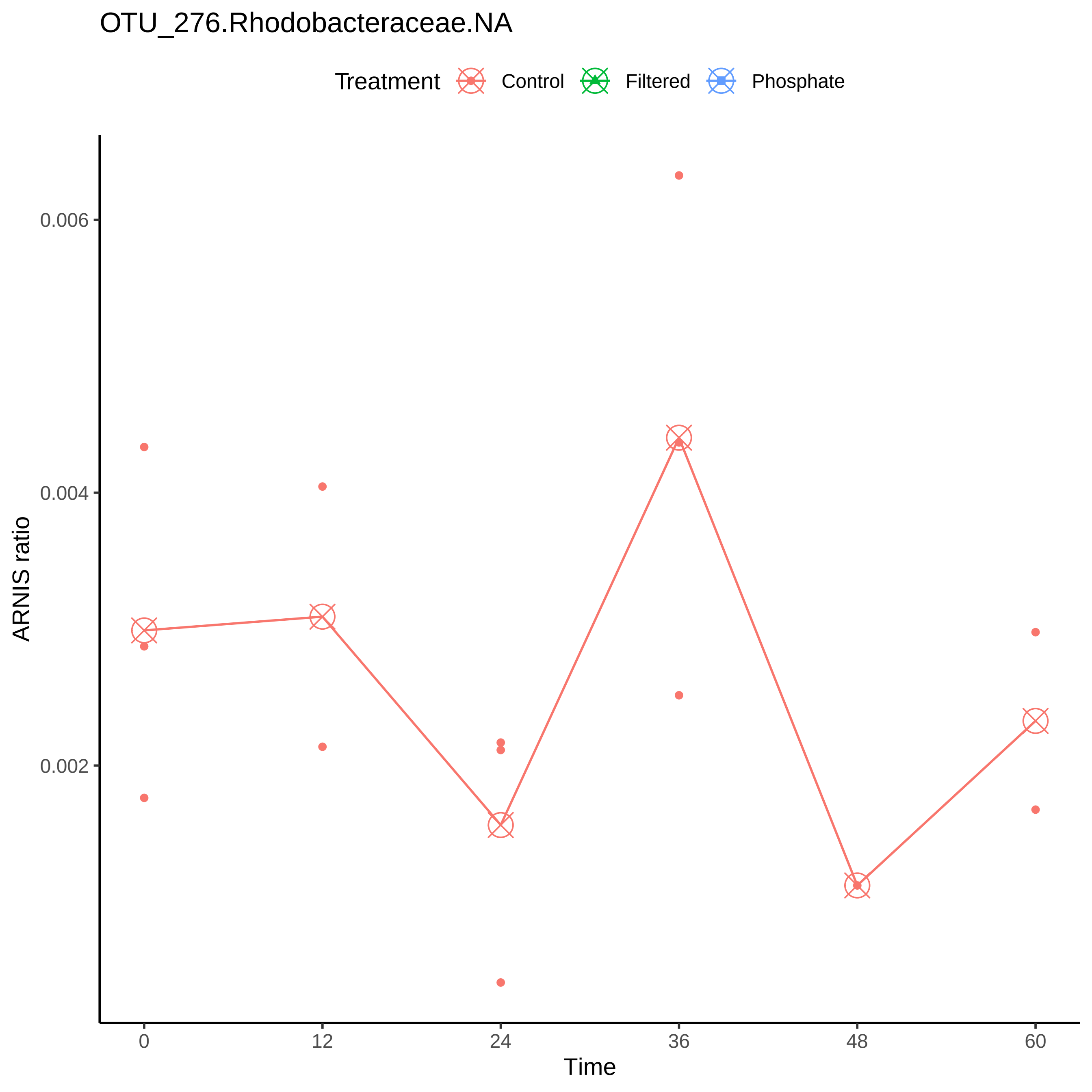
OTU_276.Rhodobacteraceae.NA
Treatment
Control
Filtered
Phosphate
0.006
0.004
0.002
ARNIS ratio
0
12
24
36
48
60
Time

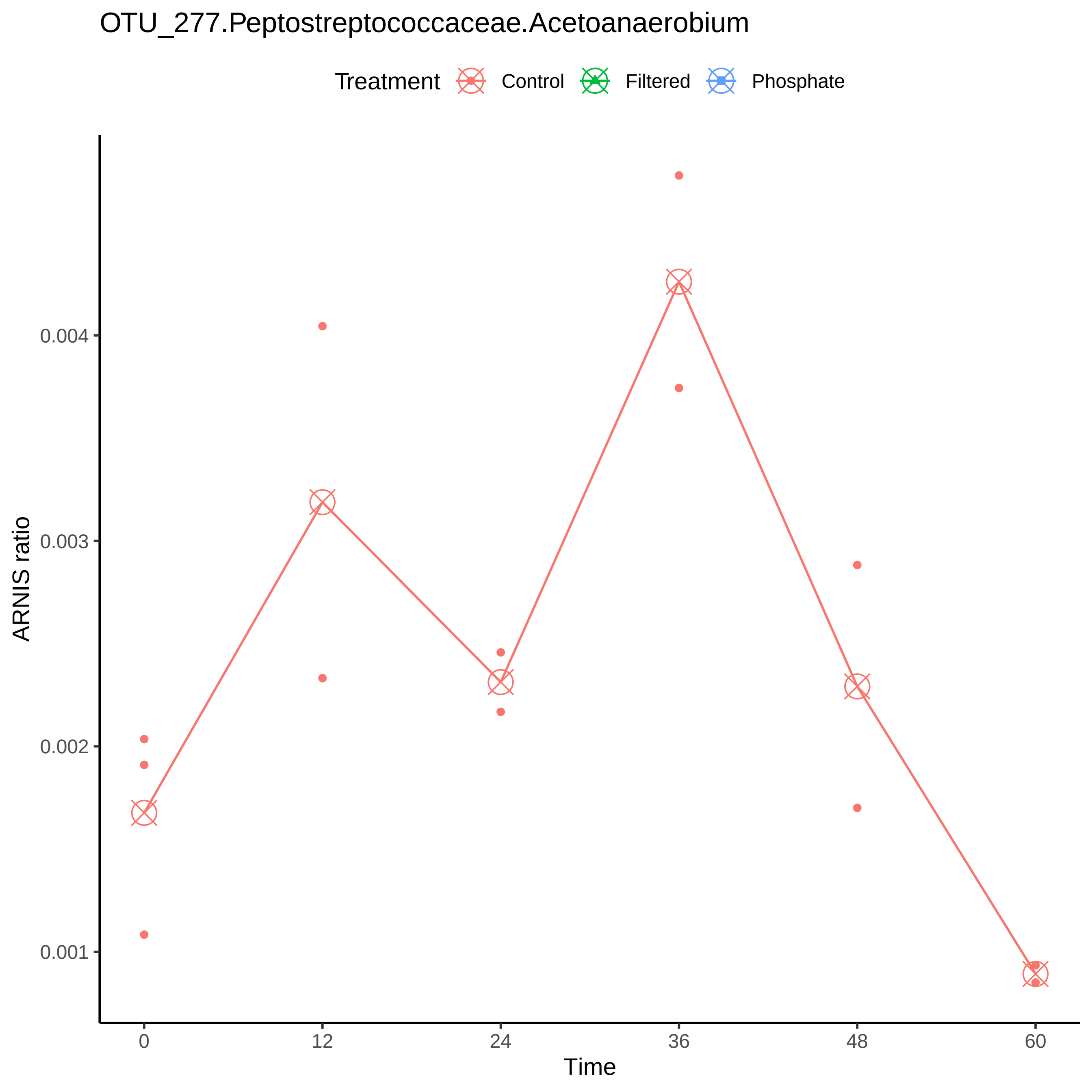
OTU_277.Peptostreptococcaceae.Acetoanaerobium
Treatment
Control
Filtered
Phosphate
ARNIS ratio
0.004
0.003
0.002
0.001
0
12
24
36
48
60
Time

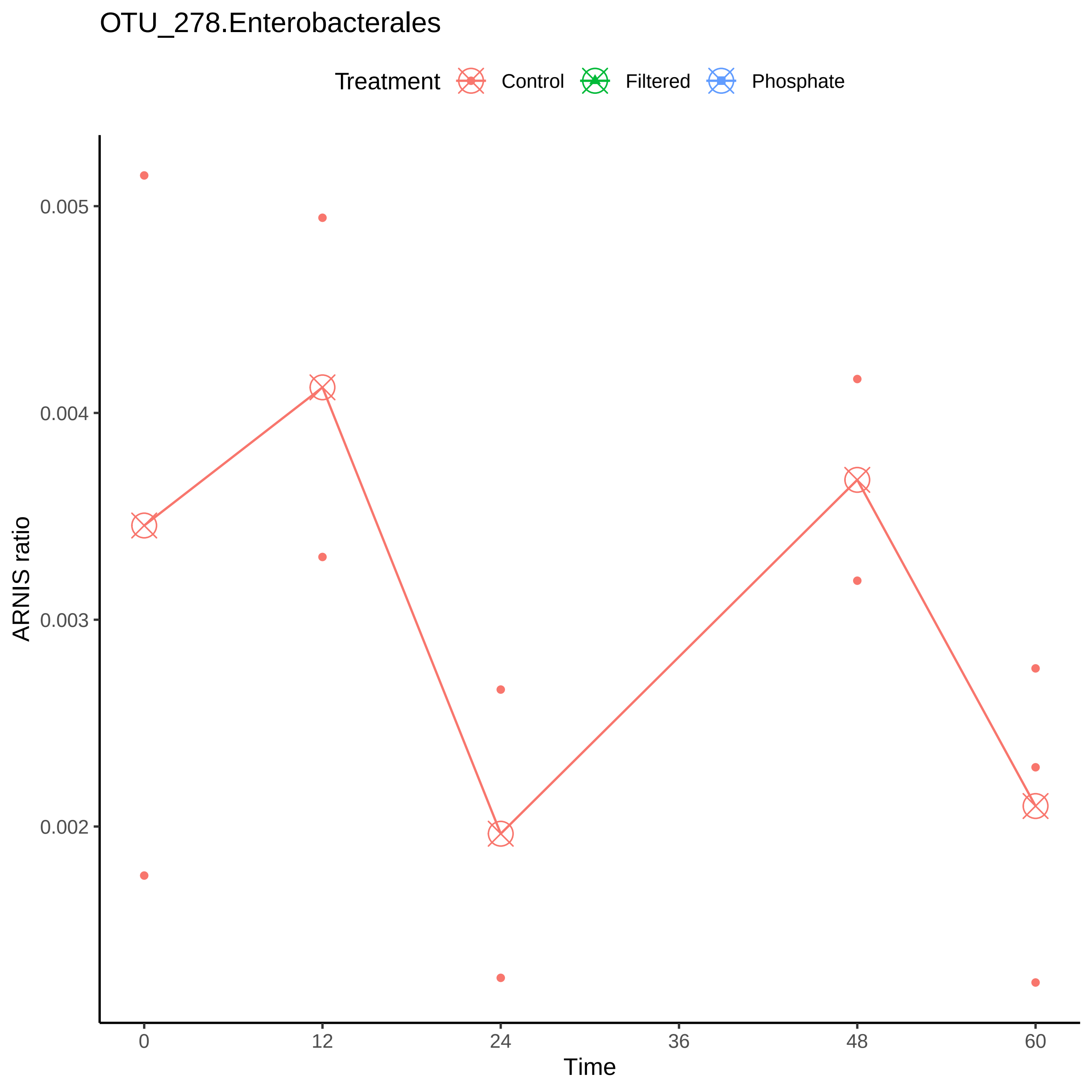

OTU_278.Enterobacterales
Treatment
Control
Filtered
Phosphate
ARNIS ratio
0.005
0.004
0.003
0.002
Time
0
12
24
36
48
60

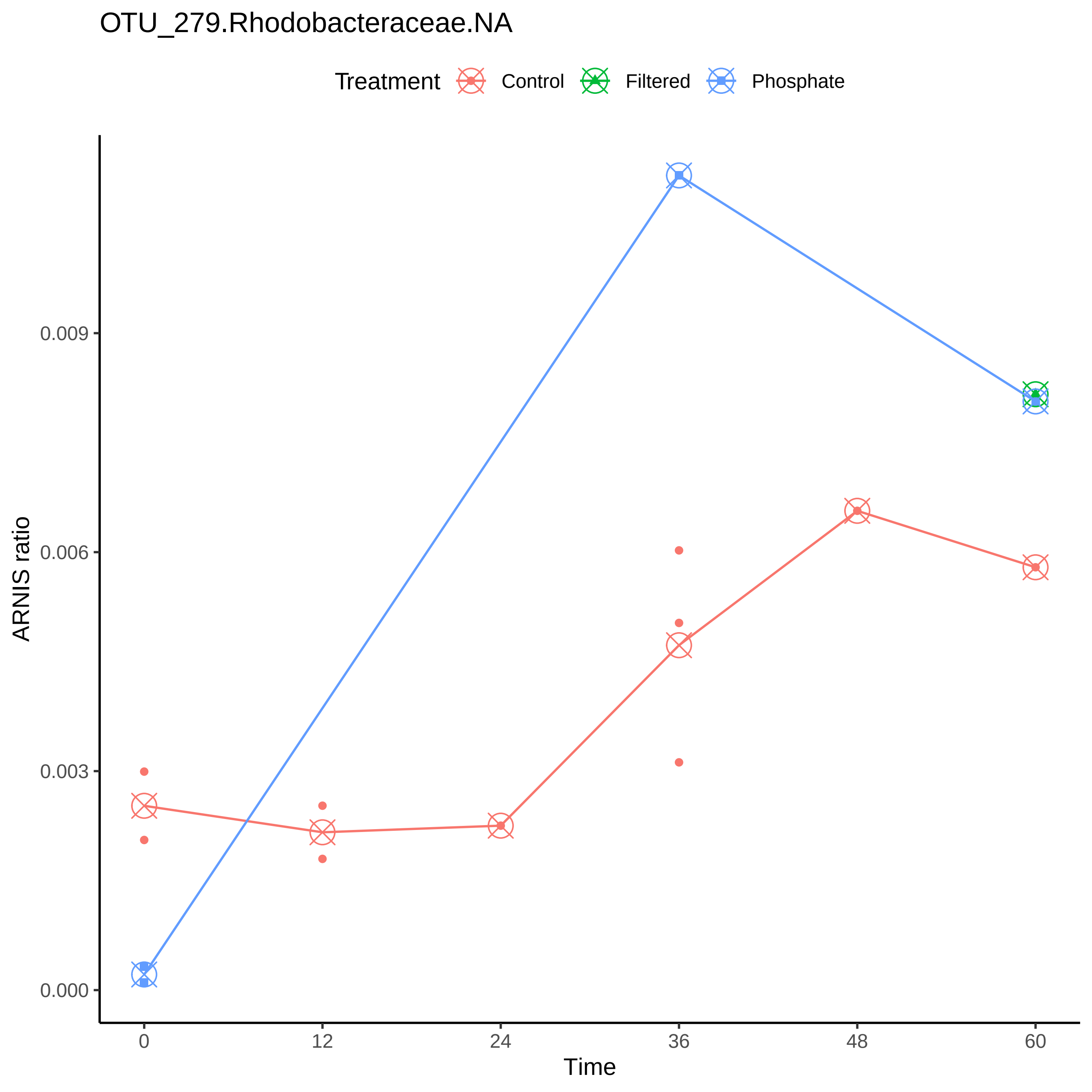
OTU_279.Rhodobacteraceae.NA
Treatment
Control
Filtered
Phosphate
ARNIS ratio
0.009
0.006
0.003
0.000
0
12
24
36
48
60
Time

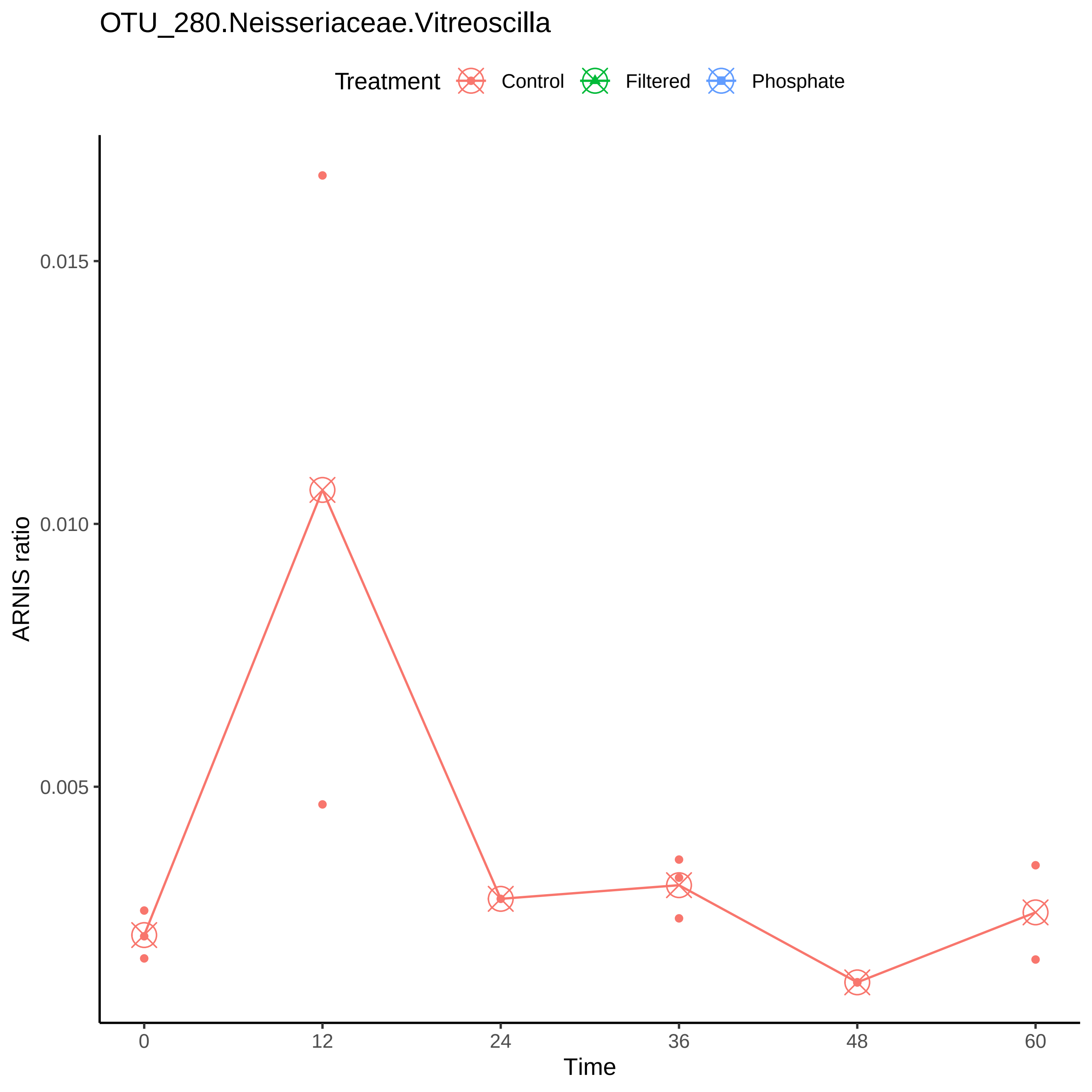
OTU_280.Neisseriaceae.Vitreoscilla
Treatment
Control
Filtered
Phosphate
ARNIS ratio
0.015
0.010
0.005
0
12
24
36
48
60
Time

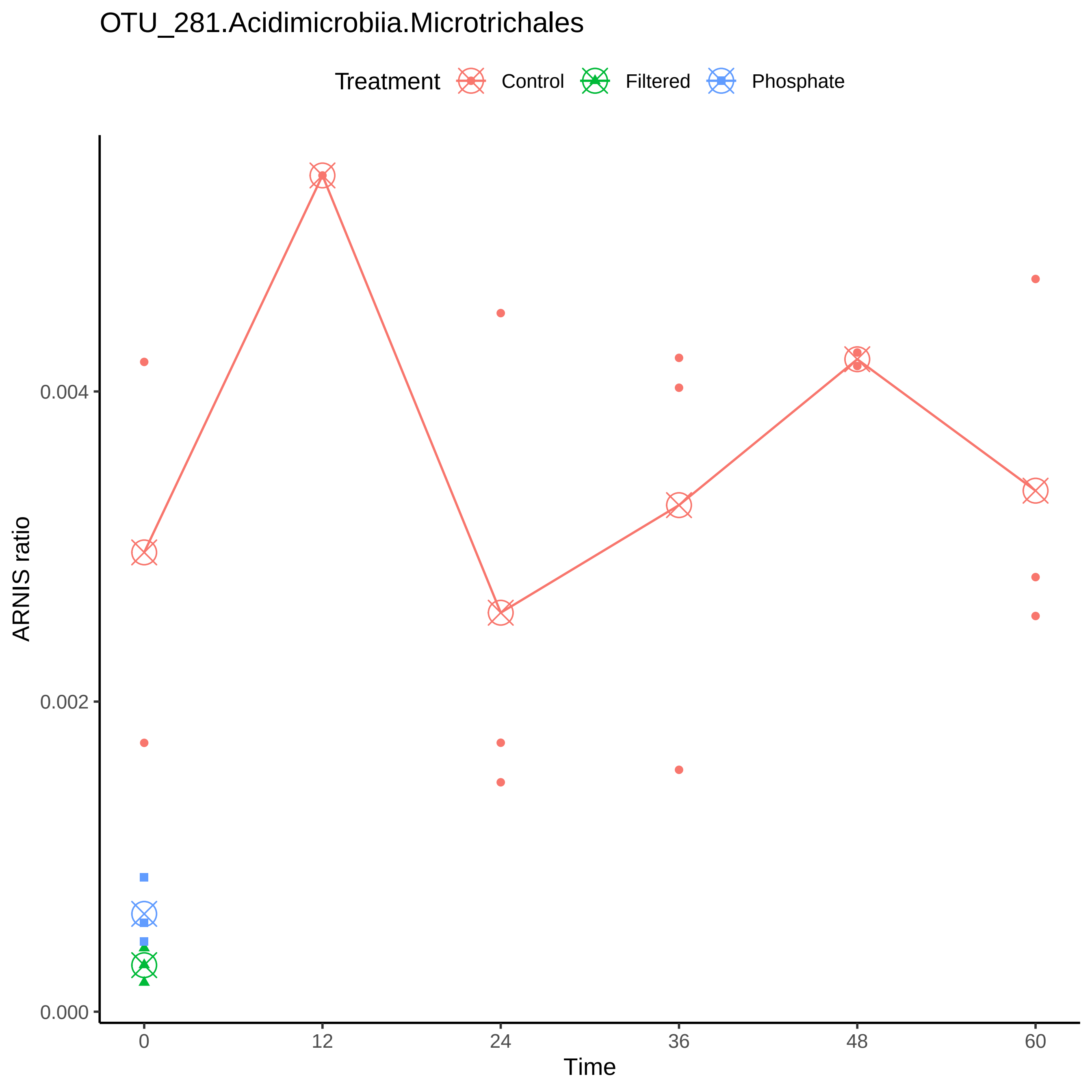

OTU_281.Acidimicrobiia.Microtrichales
Treatment
Control
Filtered
Phosphate
ARNIS ratio
0.004
0.002
0.000
0
12
24
36
48
60
Time

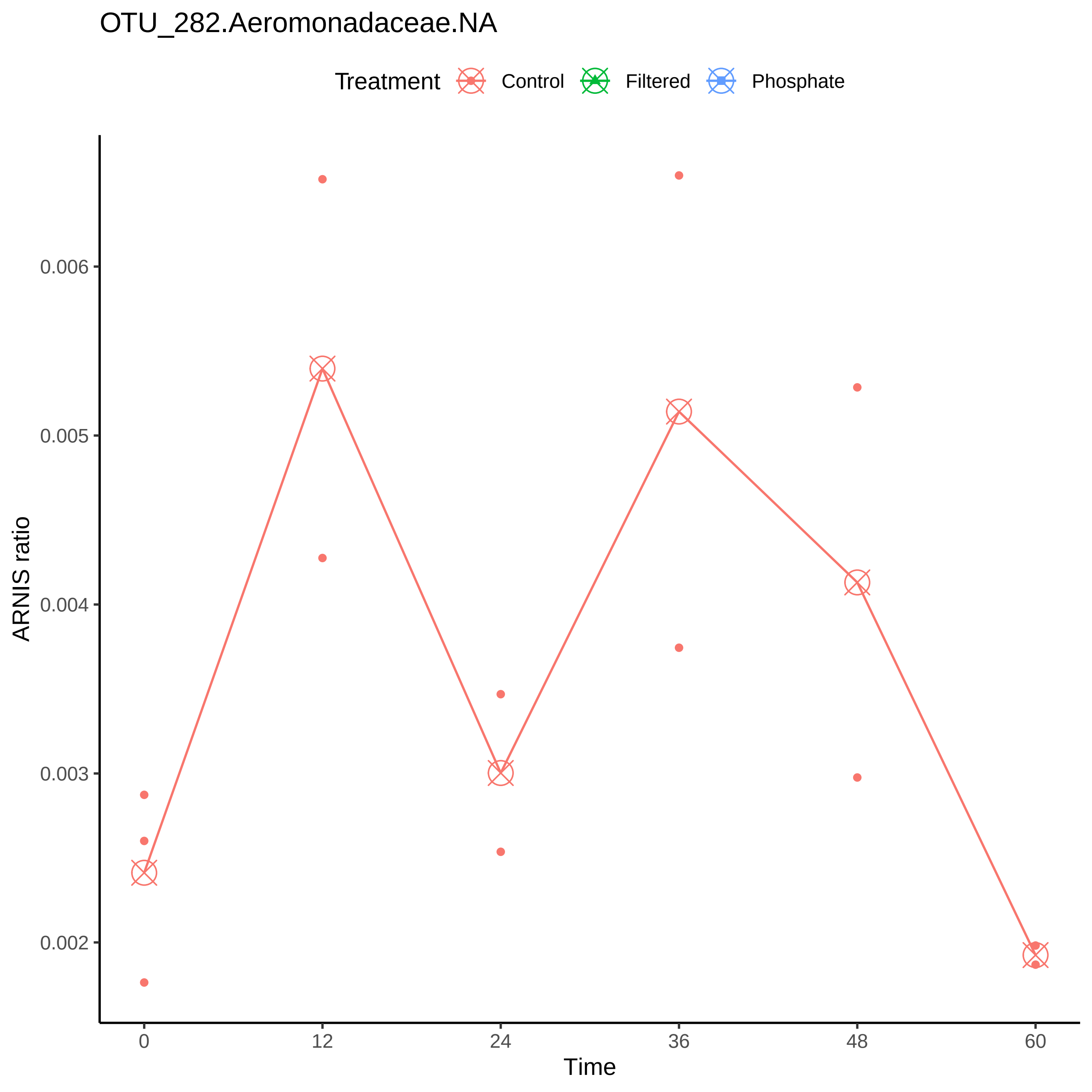

OTU_282.Aeromonadaceae.NA
Treatment
Control
Filtered
Phosphate
ARNIS ratio
0.006
0.005
0.004
0.003
0.002
0
12
24
36
48
60
Time

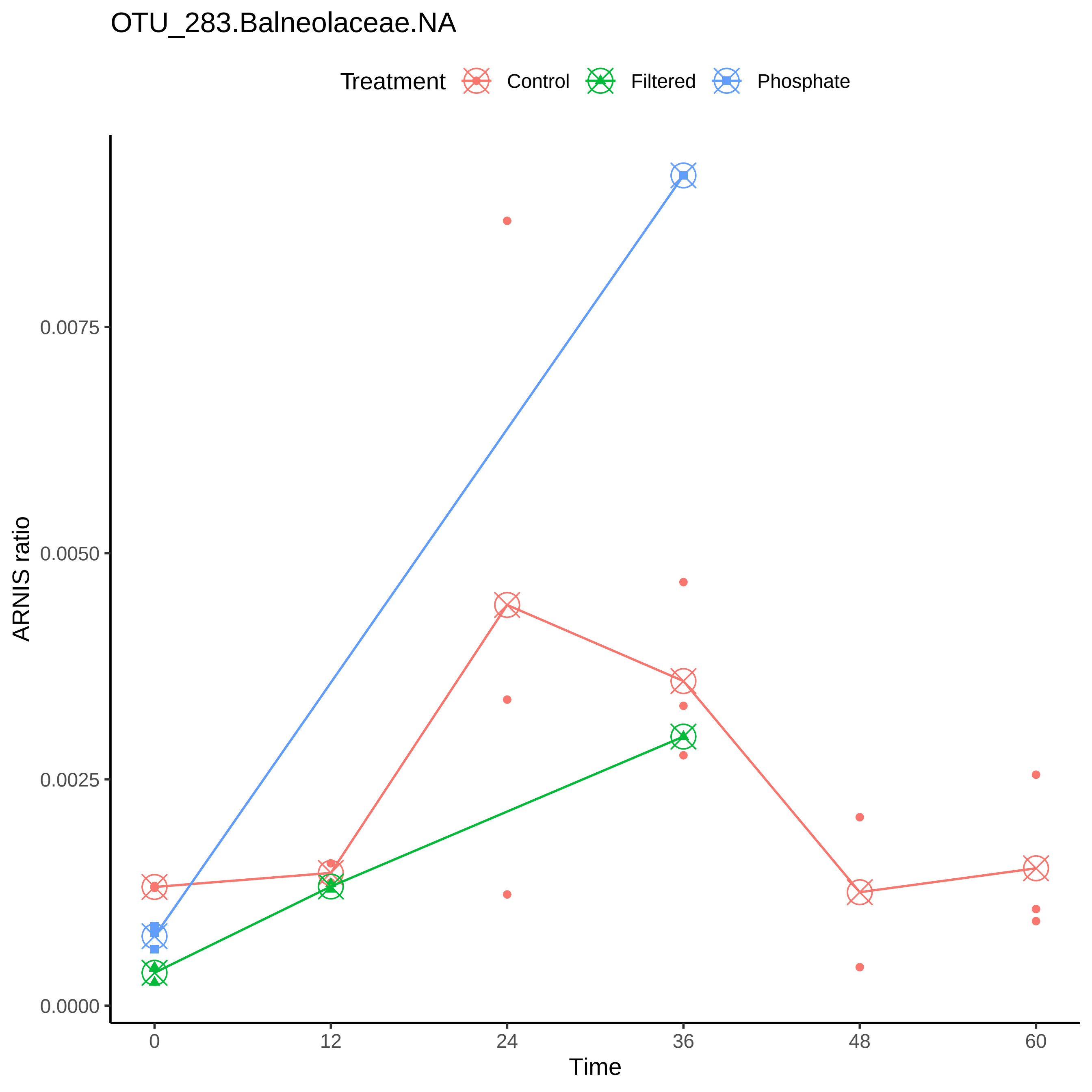
OTU_283.Balneolaceae.NA
Treatment
Control
Filtered
Phosphate
ARNIS ratio
0.0000
0.0025
0.0050
0.0075
0
12
24
36
48
60
Time

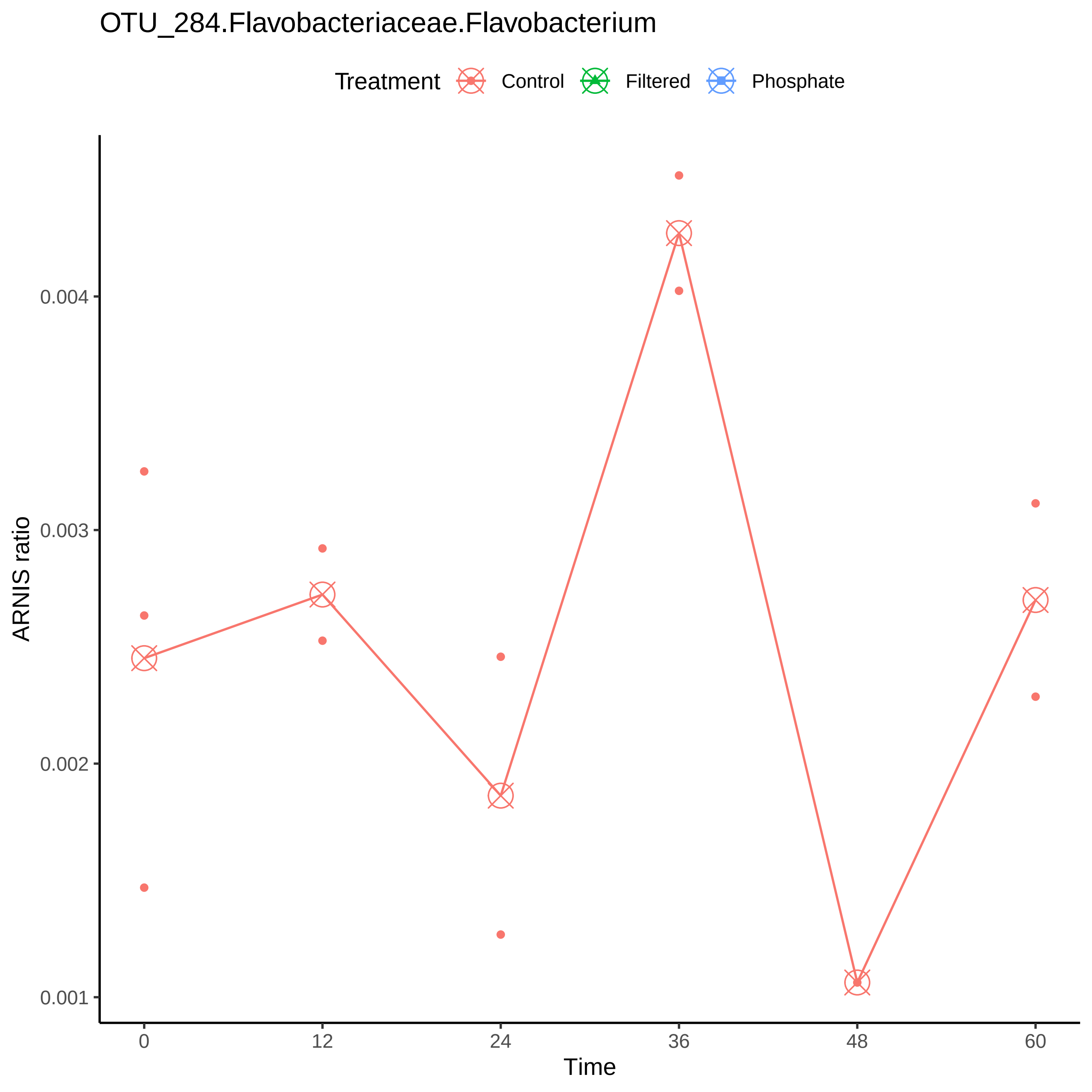

OTU_284.Flavobacteriaceae.Flavobacterium
Treatment
Control
Filtered
Phosphate
ARNIS ratio
0.004
0.003
0.002
0.001
0
12
24
36
48
60
Time

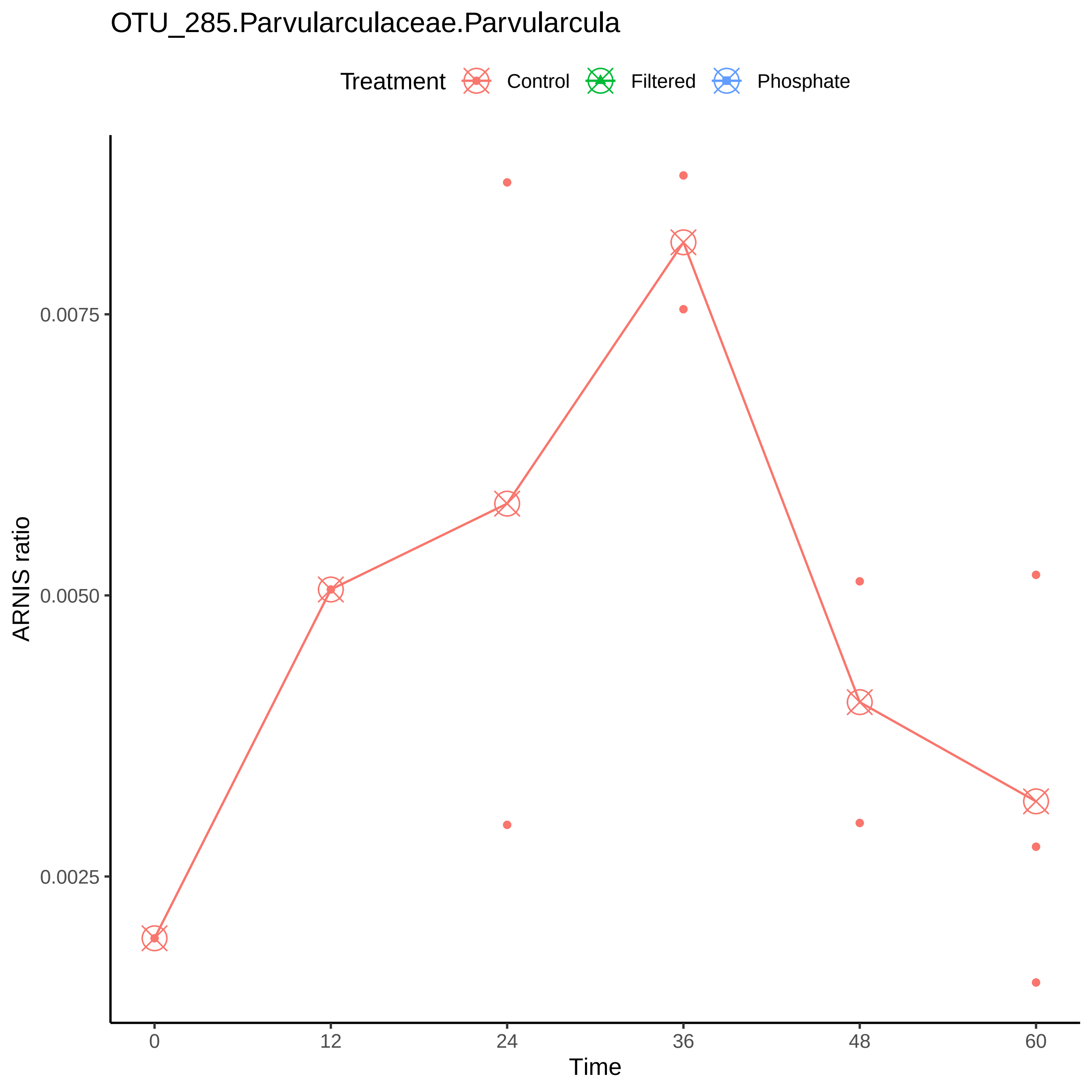
OTU_285.Parvularculaceae.Parvularcula
Treatment
Control
Filtered
Phosphate
ARNIS ratio
0.0075
0.0050
0.0025
Time
0
12
24
36
48
60

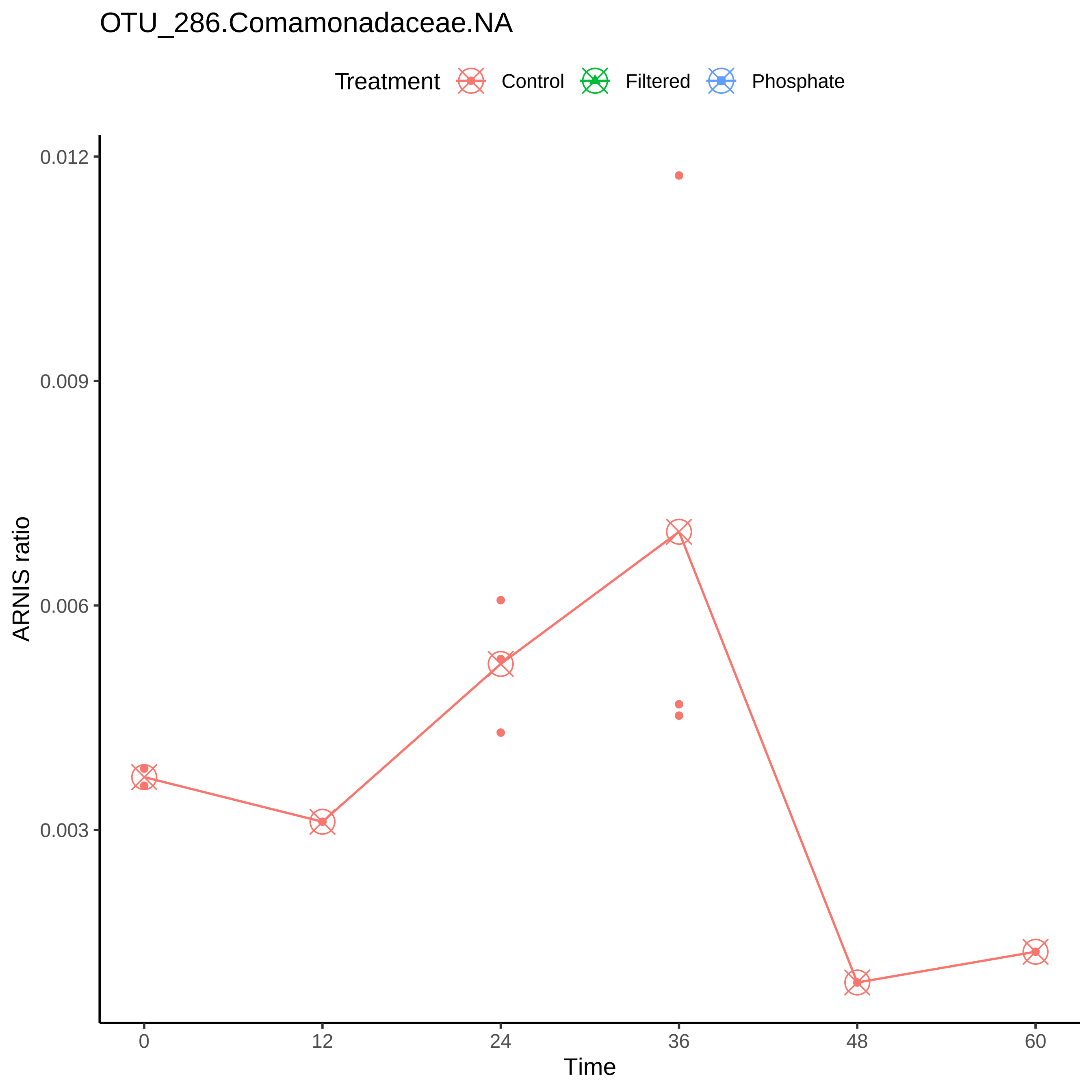
OTU_286.Comamonadaceae.NA
Treatment
Control
Filtered
Phosphate
0.012
0.009
0.006
0.003
ARNIS ratio
0
12
24
36
48
60
Time

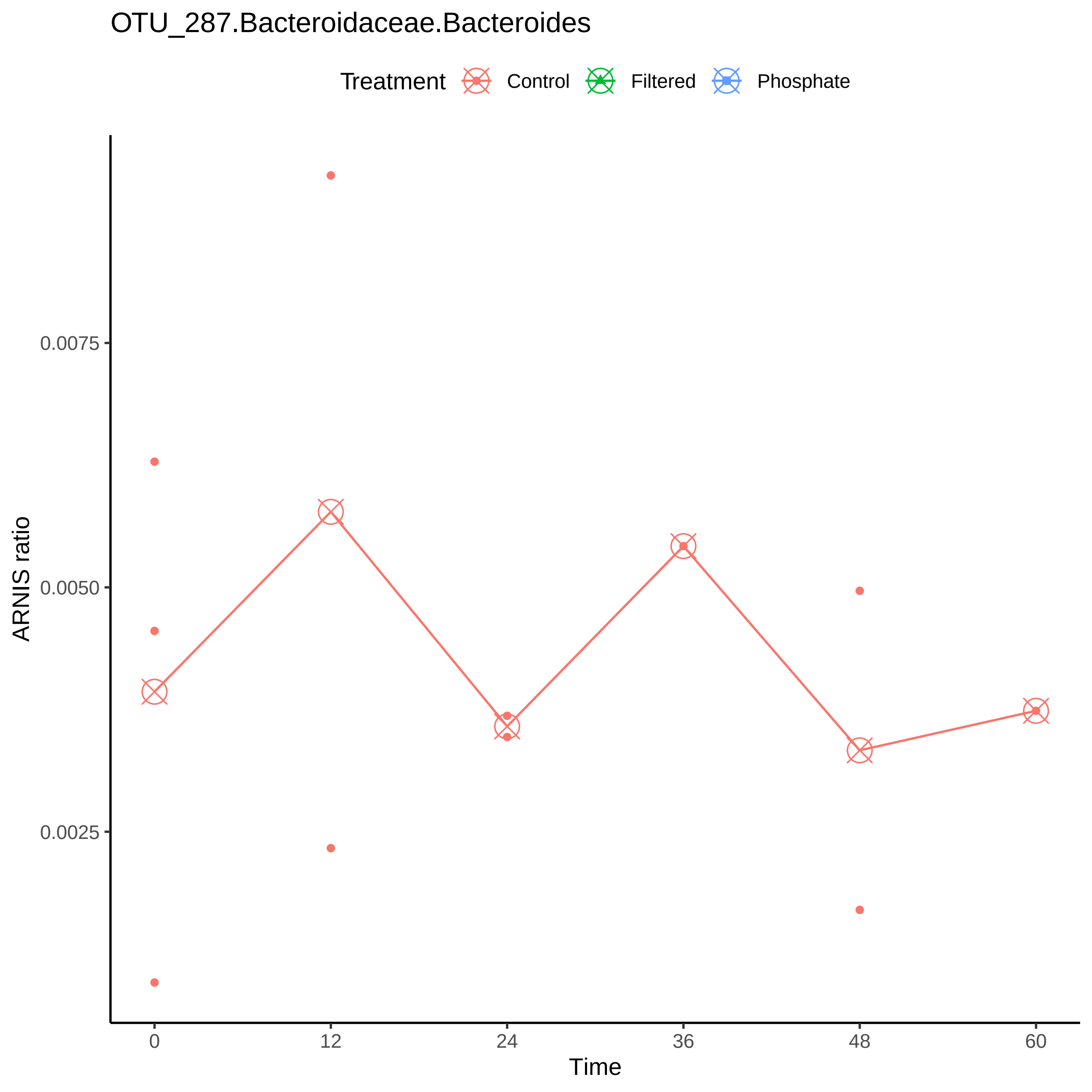
OTU_287.Bacteroidaceae.Bacteroides
Treatment
Control
Filtered
Phosphate
ARNIS ratio
0.0075
0.0050
0.0025
0
12
24
36
48
60
Time

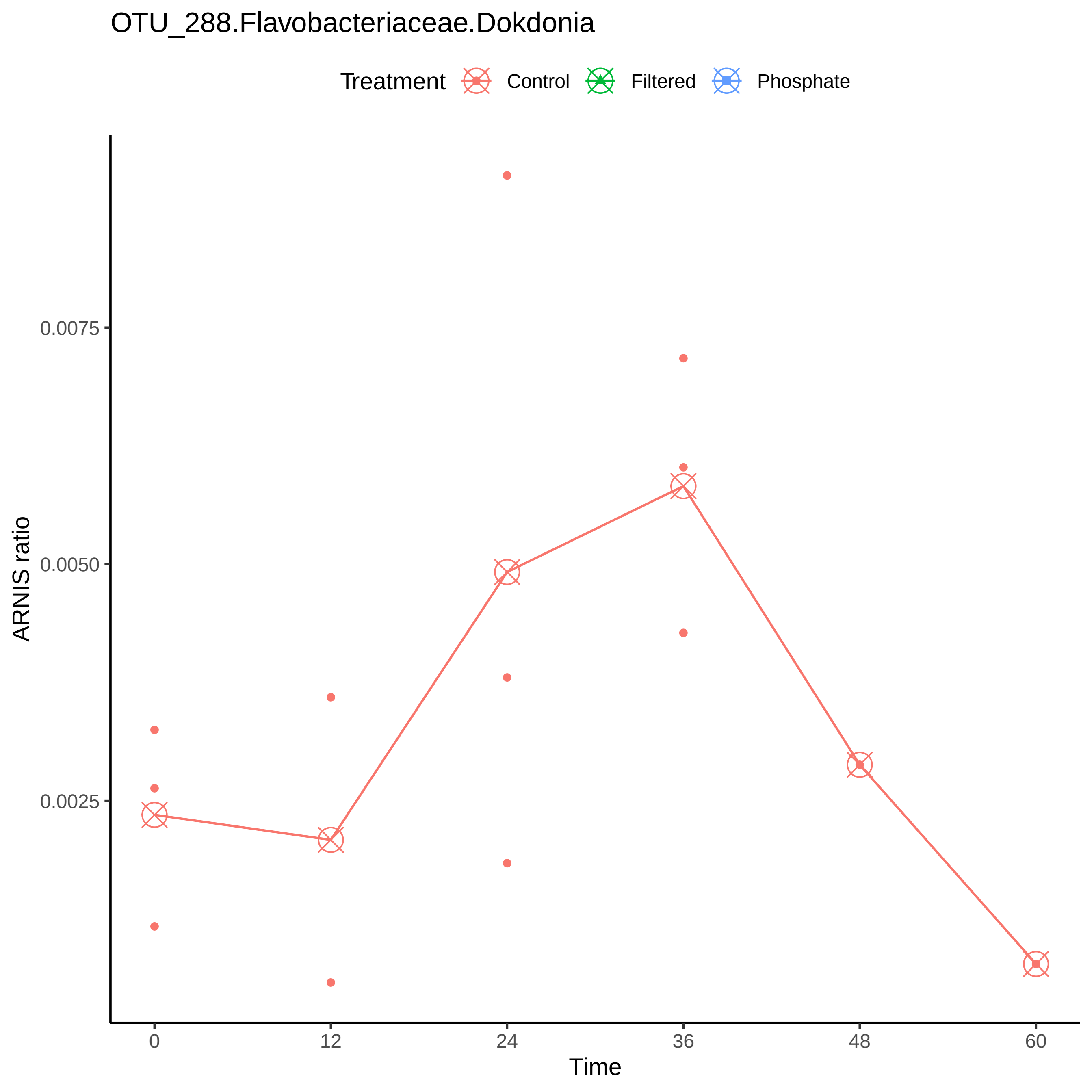

OTU_288.Flavobacteriaceae.Dokdonia
Treatment
Control
Filtered
Phosphate
ARNIS ratio
0.0075
0.0050
0.0025
0
12
24
36
48
60
Time

OTU_289.Halieaceae.Halioglobus

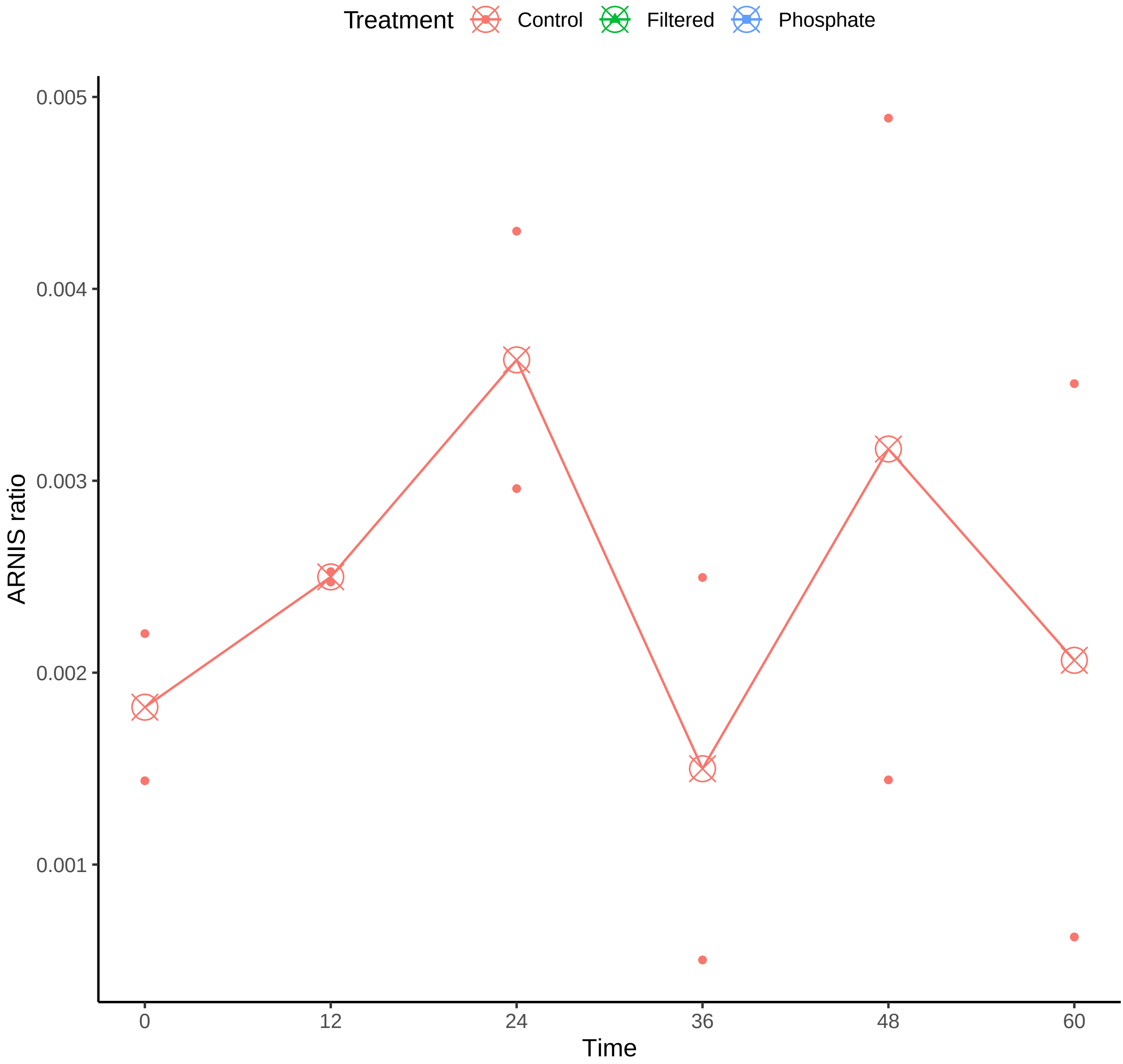

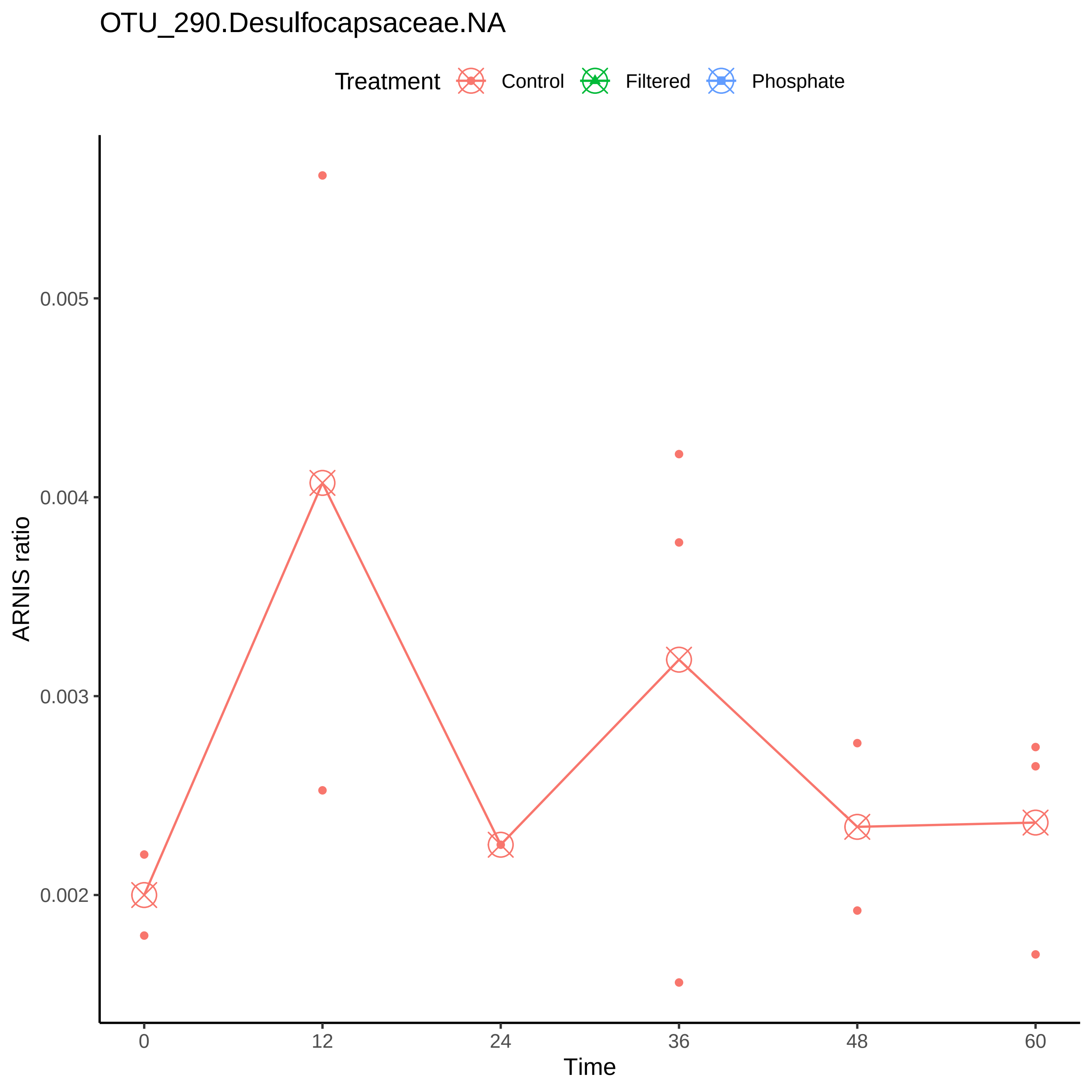
OTU_290.Desulfocapsaceae.NA
Treatment
Control
Filtered
Phosphate
ARNIS ratio
0.005
0.004
0.003
0.002
Time
0
12
24
36
48
60
