# Supplementary material for: Lineage-Specific Growth Curves Document Large Differences in Response of Individual Groups of Marine Bacteria to the Top-Down and Bottom-Up Controls
Source: mSystems. 2021 Sep 28;6(5):e00934-21. doi: 10.1128/mSystems.00934-21 (PMC8547455; doi:10.1128/mSystems.00934-21)
Supplement: TABLE S2 [file msystems.00934-21-st002.pdf]

Supplemental Table S2. Sequences of PCR primers and coverage of the 16S rRNA primers of selected groups.

(Primer coverage of selected target groups is based on SILVA138 accessed in September 2020.)

**16S rRNA primers and 5' -> 3' sequence**	**Target and coverage of both primers**	**PCR conditions**	**Reference**
**515F-Y** GTGYCAGCMGCCGCGGTAA **926 R** CCGYCAATTYMTTTRAGTTT	*Alteromonadaceae* 92% *Colwelliaceae* 97%	50°C annealing, 25 cycles	[S1]
	SAR11 clade 98%		
	Rhodobacteraceae 98%		
**pufM UniF** GGNAAYYTNTWYTAYAAYCCNTTYCA **pufM WAWR** AYNGCRAACCACCANGCCCA	NA	58°C annealing, 27 cycles	[S2]
			[S3]

S1. Parada AE, Needham DM, Fuhrman, JA. 2016. Environ Microbiol 18:1403-1414. doi: 10.1111/1462-2920.13023.

S2. Béjà O, Suzuki MT, Heidelberg JF, Nelson WC, Preston CM, Hamada T, Eisen JA, Fraser CA, DeLong EF. 2002. Nature 4–633. doi: 10.1038/415630a.

S3. Yutin N, Suzuki MT, Be O. 2005. Appl Environ Microbiol 71:8958–8962. doi: 10.1128/AEM.71.12.8958-8962.2005.
