# Supplementary material for: Lineage-Specific Growth Curves Document Large Differences in Response of Individual Groups of Marine Bacteria to the Top-Down and Bottom-Up Controls
Source: mSystems. 2021 Sep 28;6(5):e00934-21. doi: 10.1128/mSystems.00934-21 (PMC8547455; doi:10.1128/mSystems.00934-21)
Supplement: TABLE S3 [file msystems.00934-21-st003.pdf]

Supplemental Table S3. Sequences of CARD-FISH probes used in the study and their targets. (Probe coverage of the target group and outgroup hits based on SILVA138 accessed in September 2020.)

**Probe name and 5’ -> 3’ sequence**	**Target group (coverage)**	**OH***	**HB (%)****	**Reference**
ALT1413<br>TTTGCATCC CAC TCC CAT	*Alteromonadaceae* 57%<br>*Colwelliaceae* 40%	82	60	[S4]
SAR11-441R<br>TACAGTCATTTTCTTCCCCGAC	SAR11 clade I 91%<br>SAR11 clade II 90%	12	45	[S5]
ROS537<br>CAACGCTAACCCCCTCC	*Roseobacter* clade 88%	224	55	[S6]

*OH – outgroup hits

**HB – concentration of formamid in the hybridization buffer.

S4. Eilers H, Pernthaler J, Amann R. 2000. Appl Environ Microbiol 66:4634-4640. doi: 10.1128/aem.66.11.4634-4640.2000

S5. Morris RM, Rappé MS, Connon SA, Vergin KL Slebold WA, Carlson CA, Giovannoni SJ. 2002. Nature 420:806–810. doi: 10.1038/nature01240.

S6. Eilers H, Pernthaler J, Peplies J, Glöckner FO, Gerdts G, Amann R. 2001. doi:10.1128/AEM.67.11.5134-5142.2001. Appl Environ Microbiol 67:5134-5142.
